# Cumulative Weighing of Time in Intertemporal Tradeoffs

**DOI:** 10.1037/xge0000198

**Published:** 2016-09

**Authors:** Marc Scholten, Daniel Read, Adam Sanborn

**Affiliations:** 1 Department of Marketing, Universidade Europeia; 2 Department of Behavioural Science, Warwick Business School; 3 Department of Psychology, University of Warwick

**Keywords:** discounting, intertemporal choice, sequences, tradeoff, utility

## Abstract

We examine preferences for sequences of delayed monetary gains. In the experimental literature, two prominent models have been advanced as psychological descriptions of preferences for sequences. In one model, the instantaneous utilities of the outcomes in a sequence are discounted as a function of their delays, and assembled into a discounted utility of the sequence. In the other model, the accumulated utility of the outcomes in a sequence is considered along with utility or disutility from improvement in outcome utilities and utility or disutility from the spreading of outcome utilities. Drawing on three threads of evidence concerning preferences for sequences of monetary gains, we propose that the accumulated utility of the outcomes in a sequence is traded off against the *duration* of utility accumulation. In our first experiment, aggregate choice behavior provides qualitative support for the tradeoff model. In three subsequent experiments, one of which incentivized, disaggregate choice behavior provides quantitative support for the tradeoff model in Bayesian model contests. One thread of evidence motivating the tradeoff model is that, when, in the choice between two single dated outcomes, it is conveyed that receiving less sooner means receiving nothing later, preference for receiving more later increases, but when it is conveyed that receiving more later means receiving nothing sooner, preference is left unchanged. Our results show that this *asymmetric hidden-zero effect* is indeed driven by those supporting the tradeoff model. The tradeoff model also accommodates all remaining evidence on preferences for sequences of monetary gains.

Intertemporal choices are those in which the outcomes of available options are distributed over time, and tradeoffs must be made between *what* will be experienced and *when*. Examples of choices with intertemporal features are those between professional careers, medical treatments, and home mortgages. In these situations, the options of choice are well characterized as streams, or *sequences*, of outcomes.

Most laboratory studies on the psychology of intertemporal choices restrict their scope to choices between two *single* dated outcomes, as that between $100 in one year and $150 in two years. Here, the outcomes are “distributed over time,” but only *between* the available options. Some studies broaden the scope to choices between *sequences* of dated outcomes, as that between a rising and a falling profile of monetary gains, for example, {$100 in one year, $150 in two years} and {$150 in one year, $100 in two years}. Here, the outcomes are also “distributed over time,” but now *within*, as well as between, options. This comes closer to the real-life examples given above. The purpose of this paper is to investigate preferences for sequences of monetary gains. In doing so, we introduce a substantive innovation in the psychological modeling of individuals’ choices between sequences, and present both qualitative and quantitative support for this new approach.

We consider three candidate models for describing preferences for sequences: Two prominent models from the experimental literature, and a third model that we present as a challenger. In what we call the *discounted instantaneous utility model* (Abdellaoui, Attema, & Bleichrodt, 2010), instantaneous utilities are assigned to the dated outcomes in a sequence, each utility is discounted as a function of the delay to the outcome, the discounted utilities of the outcomes are assembled into the discounted utility of the sequence, and the sequence with the highest discounted utility is chosen. In what we call the *sequences model* (Loewenstein & Prelec, 1993), the accumulated utility of the outcomes in a sequence is considered along with utility or disutility from improvement in outcome utilities and utility or disutility from the spreading of outcome utilities, and the sequence with the highest utility is chosen. As a challenger, we propose a modification of the *tradeoff model* (Read & Scholten, 2012; Scholten & Read, 2010), in which the accumulated utility of the outcomes in a sequence is traded off against the *duration* of utility accumulation, and the sequence favored by this tradeoff is chosen. Qualitative and quantitative evidence from four experiments provides credence to the challenger.

Our model development is motivated by three threads of evidence concerning preferences for sequences of *unlabeled* monetary gains, meaning money that is not attributed to a specific origin, such as wage payments or rental income (e.g., Loewenstein & Sicherman, 1991). That evidence shows deviations from the *Net Present Value* (NPV) model, the standard of economic rationality found in textbooks on finance and economics (e.g., Brealey et al., 2012; Hey, 2003), and originally due to Fisher (1930). In the NPV model, each outcome in a sequence is discounted as a function of the delay to the outcome, at an interest rate determined by the opportunity cost of money, and the discounted outcomes are assembled into the NPV of the sequence. Psychologists often cite a variant of the NPV model, referred to as the exponential-discounting model, as the normative standard. In their variant, *r* is any *personal* interest rate that may differ from the market interest rate or opportunity cost of money (Read, Frederick, & Scholten, 2013; Scholten & Read, 2013). To illustrate the NPV model, consider the following choice pair, in which outcomes being distributed over three consecutive years:

**Table:** 

	Delay to outcome			Exponentially discounted outcome		
Option	1	2	3	(*x*_1_, 1)	(*x*_2_, 2)	(*x*_3_, 3)
Low NPV		$1,000			${\left(\frac{1}{1+r}\right)}^{2}1,000$	
High NPV	$500		$500	$\left(\frac{1}{1+r}\right)500$		${\left(\frac{1}{1+r}\right)}^{3}500$

The term between brackets is the *per-period discount factor*, and, within it, *r* is the interest rate. By *any* nonzero interest rate, that is, either positive (*r* > 0) or, which is nonstandard in economics, negative (−1 < *r* < 0), the “high NPV option” has a higher Net Present Value than the “low NPV option,” that is, 500/(1 + *r*) + 500/(1 + *r*)^3^ > 1,000/(1 + *r*)^2^, which solves for *r*^2^ > 0. In case of a zero interest rate, the NPV of each sequence is simply the sum of its outcomes, which, in this illustration, and in most research on preferences for sequences, is the same for both sequences. The three threads of evidence are as follows.

## Three Threads of Evidence

### Thread 1: Undecided Preference Between Decreasing and Constant Sequences

In incentivized studies, Manzini, Mariotti, and Mittone (2010) and Gigliotti and Sopher (1997) examined preferences between decreasing, constant, and increasing sequences. Sequences totaled the same amount of money, for example, {$32, $16} for a decreasing sequence, {$24, $24} for a constant sequence, and {$16, $32} for an increasing sequence. Majorities preferred decreasing and constant sequences to increasing ones (80% and 93%, respectively, in Manzini et al., 2010; 62% and 82%, respectively, in Gigliotti & Sopher, 1997). This is consistent with the NPV model, because a given amount of money sooner is always better than the same amount later. By the same logic, however, decreasing sequences are better than constant ones, and, yet, no consensus emerged, with only about half of the participants preferring decreasing sequences over constant ones (66% in Manzini et al., 2010, and 40% in Gigliotti & Sopher, 1997). Our experiments confirm that preference between decreasing and constant sequences is basically undecided (i.e., close to chance level).

### Thread 2: The Asymmetric Hidden-Zero Effect

The *hidden-zero effect* (Magen, Dweck, & Gross, 2008) is that people are less patient when choosing between single dated outcomes, for example, “$100 today” (*SS*, for Smaller-Sooner) and “$150 in one year” (*LL*, for Larger-Later), than when choosing between the outcome sequences “$100 today and $0 in one year” (*SS*_0_) and “$0 today and $150 in one year” (*LL*_0_). That is, choosing *LL* rather than *SS* is less likely than choosing *LL*_0_ rather than *SS*_0_. This is incompatible with the NPV model, because zero outcomes have zero value, and should therefore not affect preference. Subsequent research (Read, Olivola, & Hardisty, in press; Wu & He, 2012) has shown, however, that the zeroes are not equal: Adding a later zero (changing *SS* into *SS*_0_) increases patience, but adding a sooner zero (changing *LL* into *LL*_0_) has no effect. We will show that this *asymmetric hidden-zero effect* is driven by those individuals whose preferences are best described by the tradeoff model.

### Thread 3: Preference for Faster Accumulation

The third thread of evidence originally came from informal observations, which we then confirmed experimentally. It concerns the preference between the low and high NPV options discussed earlier. Given *any* time preference, the NPV model predicts a preference for the high NPV option over the low NPV option. We conducted informal surveys of colleagues and students, and, without any exception, large majorities preferred the *low* NPV option, arguing that, with the low NPV option, they would *receive the full amount sooner*. That is, they would receive the full $1,000 by the second period, whereas, with the high NPV option, they would have to wait until the third period to get that full amount. Confirming these informal observations, 71% chose the low NPV option in an MTurk survey of 429 American residents, and 79% did so in a survey of 321 British residents.^1^ We will show that, by holding on to the logic of our colleagues and students, which is that the low NPV option *accumulates faster* to $1,000 than the high NPV option, we arrive at a model of intertemporal choice that offers the best account of preferences for monetary sequences.

## Models of Preferences for Sequences

Below, we describe the three candidate models of preferences for sequences, and discuss how each deals with the three threads of evidence discussed in the introduction. It turns out that the discounted instantaneous utility model and the sequences model fail to offer a coherent account of the evidence, whereas the tradeoff model does, and in a parsimonious way. This will set the stage for four experiments, in which we examine whether the tradeoff model outperforms the other candidate models in accounting for fresh evidence.

### The Discounted Instantaneous Utility Model

We earlier described the NPV model, in which *outcomes* are discounted at a constant rate as a function of their delays. An alternative to the NPV model is the discounted instantaneous utility model, in which utilities are assigned to the outcomes (by means of a utility function that may not be linear), and the utilities are discounted at a personal rate (which may deviate from the rate deriving from the economically appropriate interest rate). Formally, the discounted utility of a sequence is given as:

$$U\left(x_{1},t_{1}\text{;}\text{ . . . }\text{; }x_{n},t_{n}\right)=\overset{n}{\underset{i=1}{\sum }}\delta^{t_{i}}u(x_{i}),$$ 1

where *x*_*i*_ is the *i*th outcome in the sequence, *t*_*i*_ is its delay, *n* is the number of outcomes in the sequence, and *u* is an instantaneous utility function satisfying *u*(0) = 0 (Abdellaoui et al., 2010; Wakker, 2010). Even though *u* might be any monotonically increasing function, it is commonly assumed to be concave over gains, meaning that it exhibits *diminishing sensitivity* (Kahneman & Tversky, 1979; Loewenstein & Prelec, 1992). Diminishing sensitivity is that the marginal impact of an outcome decreases with its absolute magnitude; for example, adding $1 to $100 has psychologically less impact than adding $1 to $10.

Table 1 summarizes how the discounted instantaneous utility model (and the models yet to be discussed) deals with the three threads of evidence. We begin with Thread 1, or the undecided preference between decreasing and constant sequences. Consider the decreasing sequence {$400, $200} and the constant sequence {$300, $300}. By constant discounting of outcomes, or computation of Net Present Value, we have δ400 + δ^2^200 > δ300 + δ^2^300, or 100 > δ100, so that the decreasing sequence would be preferred to the constant one. The discounted instantaneous utility model, however, posits discounting of outcome *utilities*. Concave utility countervails discounting, in that *u*(400) + *u*(200) < 2*u*(300), which favors the constant sequence. When concave utility *outweighs* discounting, we have δ*u*(400) + δ^2^*u*(200) < δ*u*(300) + δ^2^*u*(300), or *u*(400) – *u*(300) < δ[*u*(300) – *u*(200)], so that the constant sequence would be preferred to the decreasing one. With discounting and concave utility working in opposite directions, there may be no consensus on the preference between decreasing and constant sequences, which is Thread 1. The discounted instantaneous utility model further predicts that the increasing sequence {$200, $400} will lose from the decreasing sequence {$400, $200}, by discounting, and from the constant sequence {$300, $300}, by both discounting and concave utility, consistent with the results from Manzini et al. (2010) and Gigliotti and Sopher (1997).[Table-anchor tbl1]

Importantly, any preference for a constant sequence over a decreasing one is a violation of *dominance*, a cornerstone in the rational choice paradigm (Tversky & Kahneman, 1986), which, in the domain of intertemporal choice, affirms that, when the cumulative outcome of sequence *X* in the successive time periods is never worse than that of sequences *Y*, and better in at least one time period, then *X* dominates *Y*, and *should* be chosen (Scholten & Read, 2014). This definition of dominance assumes that more money is better than less (*monotonicity*), and that money is better sooner than later (*positive time preference*; Fisher, 1930). Thus, holding the total amount earned constant, a decreasing sequence dominates a constant one, and a constant sequence dominates an increasing one. By dominance, people should indeed prefer decreasing and constant sequences to increasing ones, as observed, but they should *also* prefer decreasing sequences to constant ones, while no consensus emerged. The discounted instantaneous utility model offers an explanation for this, by suggesting that concave utility countervails discounting, and is therefore a *nonnormative* model.

The discounted instantaneous utility model can further accommodate the preference for faster accumulation (Thread 3) when assuming that discounting is outweighed by *convex* utility, or *augmenting sensitivity*, in which case we have δ*u*(500) + δ^3^*u*(500) < δ^2^*u*(1,000), or δ^−1^*u*(500) + δ*u*(500) < *u*(1,000). Concave utility over gains is a nonstandard assumption, and, in any case, the discounted instantaneous utility model needs *concave* utility to accommodate the undecided preference between decreasing and constant sequences (Thread 1). It thus cannot offer a coherent account of the prior evidence.

The discounted instantaneous utility model cannot account for the (asymmetric) hidden-zero effect (Thread 2) either, because zero outcomes have zero utility, and should therefore not affect preference.

### The Sequences Model

Loewenstein and Prelec (1993) developed the sequences model. “The guiding idea behind the model,” according to the authors, “is that evaluation of sequences reflects the interaction between two motives: a basic preference for improvement tempered by a desire to spread the better outcomes more or less uniformly over the entire interval” (p. 92). In proposing a preference for improvement, they were partly motivated by Loewenstein and Sicherman’s (1991) finding that people preferred increasing over decreasing sequences of monetary gains. As mentioned earlier, Manzini et al. (2010) and Gigliotti and Sopher (1997) found a strong preference for decreasing over increasing sequences. Loewenstein and Sicherman (1991) used large amounts of money, labeled the money, that is, specified its origin (wage payments or rental income), and presented the sequences using column charts to museum visitors, whereas Manzini et al. (2010) and Gigliotti and Sopher (1997) used small amounts of money, did not label the money, and presented sequences using delay-by-payoff matrices to college students. It is not entirely clear which methodological differences are responsible for the opposite results, but preference for increasing sequences is more pronounced for larger amounts of money (Duffy & Smith, 2013), more pronounced for income from labor than for income from rent (Loewenstein & Sicherman, 1991) and income from lotteries (Duffy & Smith, 2013; Duffy, Smith, & Woods, 2015), perhaps because people *expect* wages to increase over time (Chapman, 1996), and more pronounced among naïve people than among (financially) sophisticated people (Guyse, Keller, & Eppel, 2002; Loewenstein & Sicherman, 1991; Matsumoto, Peecher, & Rich, 2000). In any case, the sequences model is very general, in that it allows individuals to either like or dislike improvement, and either like or dislike spreading, so we will give it maximum latitude.

As applied to sequences of future outcomes distributed over contiguous time periods (e.g., three years, as in the choice between the high and low NPV options), the sequences model has three components: One is the sum of instantaneous utilities, and the others are the “‘gestalt’ properties of sequences” (Loewenstein & Prelec, 1993, p. 94): Improvement and spreading.

The sequences model draws on the concept of *cumulative* instantaneous utilities. Imagine that, in each period, some instantaneous utility is received, and a running total of that utility is kept. This is the cumulative instantaneous utility. This “real” running total is compared with an “imaginary” running total from a constant utility stream across the periods. More precisely, in each period, the utility that has accumulated until that period is compared with the utility that would have accumulated until that period had the same sum of utility been distributed uniformly over time:

$$d_{i}=\frac{i}{n}\sum_{j=1}^{n}u\left(x_{j}\right)-\sum_{j=1}^{i}u(x_{j}).$$ 2a

where the second term on the right is the utility that has accumulated until each period *i*, and the first term on the right is the utility that would have accumulated until that period had the same sum of utility been distributed uniformly over time. To see how this works, assume linear utility, that is, *u*(*x*) = *x*, and consider the sequence *S* = {$100, $200, $300}, the cumulative outcomes of which are *C*_*S*_ = {$100, $300, $600}. Had the outcomes been distributed evenly over time, we would have had the (flat) sequence *F* = {$200, $200, $200}, the cumulative outcomes of which are *C*_*F*_ = {$200, $400, $600}. We see that *C*_*F*_ “runs ahead” of *C*_*S*_ by $200 – $100 = $100 in period 1, and by $400 – $300 = $100 in period 2. The *improvement score* of the sequence *S* is the sum of these differences, that is, Σ*d*_*i*_, or $100 + $100 = $200, which is a positive number, and therefore an indication of improvement rather than *decline* (Loewenstein & Prelec, 1993). It is no coincidence that the improvement score equals the difference between the outcome afforded by *S* in period 3 and the outcome afforded in period 1, that is, $300 – $100 = $200, because, for any three-outcome sequence {*x*_1_, *x*_2_, *x*_3_}, the improvement score is *u*(*x*_3_) – *u*(*x*_1_), which is the difference between the utility of the last outcome and the utility of the first outcome in the sequence. For an increasing sequence, the improvement score is positive; for a constant sequence, it is zero; and, for a decreasing sequence, it is negative. To capture an individual’s attitude toward improvement, the improvement score is weighted by β, where β > 0 is a desire for improvement, β = 0 is neutrality toward improvement, and β < 0 is distaste for improvement.^2^

The *spreading score* of a sequence is the sum of the *absolute* differences, that is, Σ|*d*_*i*_|, which indicates how much the sequence deviates from a uniform distribution. For the sequence *S*, all differences are positive, so that its spreading score equals the improvement score. More generally, for a three-outcome sequence {*x*_1_, *x*_2_, *x*_3_}, the spreading score is 1/3(|*u*(*x*_3_) + *u*(*x*_2_) – 2*u*(*x*_1_)| + |2*u*(*x*_3_) – *u*(*x*_2_) – *u*(*x*_1_)|), which contrasts the utilities in periods 3 and 2 with the utility in period 1, and contrasts the utility in period 3 with the utilities in periods 2 and 1. For a constant sequence, the spreading score is zero; for an increasing or a decreasing sequence, it is positive. To capture an individual’s attitude toward spreading, the spreading score is weighted by −σ, where σ > 0 is a desire for spreading (meaning that the utility of the sequence *decreases* more with a larger deviation from a uniform distribution), σ = 0 is neutrality toward spreading, and σ < 0 is distaste for spreading (meaning that the utility of the sequence *increases* more with a larger deviation from a uniform distribution).^3^ Formally, the utility of a sequence is given as follows:

$$U\left(x_{1},t_{1}\text{;}\text{ . . . }\text{; }x_{n},t_{n}\right)=\overset{n}{\underset{i=1}{\sum }}u\left(x_{i}\right)+\beta \overset{n}{\underset{i=1}{\sum }}d_{i}-\sigma \overset{n}{\underset{i=1}{\sum }}\left|d_{i}\right|.$$ 2b

Loewenstein and Prelec (1993) applied the model in Equations (2a) and (2b) to decisions about consumption experiences, for example, weekends differing in their pleasantness, and observed a preference for improvement tempered by a desire for spreading. As mentioned, however, the model is very general, and allows for any attitude toward improvement and spreading. Moreover, its flexibility can be enhanced even further by alternative treatments of concealed zero outcomes.

In one variant of the sequences model, which we call the *no-zero sequences model*, concealed zeroes are *not* inferred. The no-zero sequences model can accommodate the asymmetric hidden-zero effect (Thread 2) if it invokes a desire for improvement and a desire for spreading, consistent with Loewenstein and Prelec’s (1993) findings. Either zero creates a sequence that deviates from a uniform distribution, which, under a desire for spreading, is evaluated negatively. The later zero (changing *SS* into *SS*_0_) creates a decreasing sequence, which, under a desire for improvement, is evaluated negatively as well. With the desire for improvement reinforcing the desire for spreading, the later zero will increase preference for *LL*. In contrast, the sooner zero (changing *LL* into *LL*_0_) creates an increasing sequence, which, under a desire for improvement, is evaluated positively. With the desire for improvement countervailing the desire for spreading, the sooner zero may not affect preference between *SS* and *LL*_0_, and it will not do so if the desire for improvement precisely offsets the desire for spreading. Thus, to accommodate the asymmetric hidden-zero effect, the no-zero sequences model can preserve a desire for improvement and a desire for spreading. However, to accommodate preference for decreasing over increasing sequences, which accompanies the undecided preference between decreasing and constant sequences (Thread 1) and preference for constant over increasing sequences, the nonzero sequences model needs *distaste* for improvement. It therefore fails to offer a coherent account of the prior evidence.

In the other variant of the sequences model, which we call the *zero sequences model*, concealed zeroes *are* inferred. For this variant, the asymmetric hidden-zero effect (Thread 2) is insurmountable. When the later zero is revealed by the experimenter (changing *SS* into *SS*_0_), the sooner zero is inferred by the decision maker (changing *LL* into *LL*_0_), and, when the sooner zero is revealed by the experimenter (changing *LL* into *LL*_0_), the later zero is inferred by the decision maker (changing *SS* into *SS*_0_). Because either zero changes the choice into one between *SS*_0_ and *LL*_0_, it cannot be that one zero has an effect, whereas the other has not. Thus, the zero sequences model also fails to accommodate the prior evidence. Table 1 provides additional details on how the sequences model can enforce its flexibility to address the isolated pieces of prior evidence.

### The Tradeoff Model

Whereas, in the sequences model, the accumulated utility of the outcomes is combined with the utility deriving from a desire or distaste for improvement and the utility deriving from a desire or distaste for spreading, we propose an instantiation of the intertemporal tradeoff model in which the accumulated utility is traded off against the *duration* of utility accumulation. Generally, the duration of a sequence is some average of the constituent delays:

t^=∑j=1najtj, where ∑j=1naj=1.

In the instantiation of the intertemporal tradeoff model proposed by Read and Scholten (2012), duration lies between two limits. One is the unweighted average of constituent delays, *a*_*j*_ = 1/*n*, and the other is the weighted average of constituent delays, with each delay being weighted by the relative magnitude of the outcome received following that delay^4^:

$$a_{j}=\frac{x_{j}}{\sum_{i=1}^{n}x_{i}}.$$

Whatever the computation, the duration of a sequence lies between the delay to the first outcome (*t*_1_) and the delay to the last outcome (*t*_*n*_), because money is received after *t*_1_, meaning that the duration will be longer than *t*_1_, and money is received before *t*_*n*_, meaning that the duration will be shorter than *t*_*n*_: The duration thus lies “somewhere in between.” In light of Thread 3 (preference for faster accumulation), we propose to preserve the concept of duration, but, in its computation, to weigh individual delays by *cumulative outcome utilities*, meaning that *a*_*j*_ is given as follows:

$$a_{j}=\frac{\overset{j}{\underset{k=1}{\sum }}u\left(x_{k}\right)}{\overset{n}{\underset{i=1}{\sum }}\left(\overset{i}{\underset{k=1}{\sum }}u\left(x_{k}\right)\right)}.$$

When *a*_*j*_ is specified in this way, *t̂* becomes the *duration of utility accumulation*, obtained through a *cumulative weighing of time*, with each *t*_*j*_ being weighted by the utility accumulating until period *j*, normalized by the utility accumulating until all time periods:

t^=∑j=1n∑k=1ju(xk)∑i=1n(∑k=1iu(xk))tj. 3a

Consider the high NPV option {$500 in one year, $500 in three years}, with which the utility accumulating to period 1 is *u*(500), the utility accumulating until period 3 is *u*(500) + *u*(500), and the utility accumulating until both time periods is *u*(500) + [*u*(500) + *u*(500)]. Thus, the duration of the high NPV option is as follows:

t^H=u(500)u(500)+[u(500)+u(500)]×1+u(500)+u(500)u(500)+[u(500)+u(500)]×3 = 2.33.

In contrast, the low NPV option ($1,000 in two years) is a single dated outcome, which has no duration, and its delay is *t*_*L*_ = 2. Because the duration of the high NPV option is longer than the delay of the low NPV option, that is, *t̂*_*H*_ = 2.33 > 2 = *t*_*L*_, the cumulative weighing of time contributes to a preference for the low NPV option.

To complete the analysis, we must specify the tradeoff model in its full form. The tradeoff model is an *attribute-based* model, in that outcome and time advantages of the options are traded off against one another, and the option favored by the tradeoff is chosen. In contrast, the previous models are *alternative-based* models, in that each option receives a utility, independently of the other option, and the option with the highest utility is chosen. According to the tradeoff model, the decision maker will be indifferent between the high NPV option (*H*) and the low NPV option (*L*) when:

∑i=1nu(xHi)−∑i=1nu(xLi)=κ[w(t^H)−w(t^L)], 3b

where, analogous to δ < 1 in discounting models, κ > 0 captures impatience, with higher values benefiting the sequence with the shortest duration, and *w* is a (concave) time-weighing function (thus exhibiting diminishing sensitivity).^5^ Furthermore, the difference between the accumulated utilities is an advantage of *H* when positive and an advantage of *L* when negative, whereas the difference between weighted durations is an advantage of *L* when positive and an advantage of *H* when negative. In the above choice between the high NPV option and the low NPV option, the low NPV option is a single dated outcome, so that *t̂*_*L*_ – *t*_*L*_, and this delay is shorter than the duration of the high NPV option, that is, *t*_*L*_ < *t̂*_*H*_, which contributes to preference for the low NPV option. By diminishing sensitivity to outcomes (concavity of the utility function *u*), however, the accumulated utility of the high NPV option will be higher than the utility of the low NPV option, that is, *u*(500) + *u*(500) > *u*(1,000), contributing to a preference for the high NPV option. Preference for the low NPV option (Thread 3) occurs when concave utility is outweighed by the cumulative weighing of time.

The tradeoff model in Equations (3a) and (3b) also accounts for the asymmetric hidden-zero effect (Thread 2). The smaller-sooner outcome (*SS*) is “$100 today.” This option has no duration, and its delay is *t*_*SS*_ = 0. Revealing the later zero outcome, that is, “$100 today and $0 in one year” (*SS*_0_), creates a sequence, which *has* duration. Specifically, the duration of *SS*_0_ is:

t^SS0=u(100)u(100)+u(100)+u(0)×0+u(100)+u(0)u(100)+u(100)+u(0)×1 = 12.

Because *t̂*_*SS*_0__ = 1/2 > 0 = *t*_*SS*_, revealing the later zero increases preference for *LL*. Conversely, the larger-later outcome (*LL*) is “$150 in one year,” the delay of which is *t*_*LL*_. Revealing the sooner zero outcome, that is, “$0 today and $150 in one year” (*LL*_0_), creates a sequence, the duration of which is:

t^LL0=u(0)u(0)+u(0)+u(150)×0+u(0)+u(150)u(0)+u(0)+u(150)×1=1.

Because *t̂*_*LL*__0_ = 1 = *t*_*LL*_, revealing the sooner zero does not affect preference between *SS* and *LL*. Therefore, a cumulative weighing of time naturally produces the asymmetric hidden-zero effect.

Finally, the tradeoff model in Equations (3a) and (3b) agrees with the one proposed by Read and Scholten (2012) that duration is longer for increasing than for constant sequences, and longer for constant than for decreasing sequences. Increasing sequences lose from decreasing sequences because of their longer duration, and lose from constant sequences not only because of their longer duration, but also because of concave utility. The undecided preference between decreasing and constant sequences (Thread 1) occurs because duration (favoring the decreasing sequence) is countervailed by concave utility (favoring the constant sequence). Overall, and as summarized in Table 1, a tradeoff model in which the accumulated utility of a sequence is traded off against the duration of utility accumulation offers a coherent and parsimonious account of all prior evidence.

As mentioned in Footnote 5, Equation (3b) may be rearranged into an (arithmetically) “discounted utility” model, by which Σ*u*_*H*_ − κ*t̂*_*H*_ = Σ*u*_*L*_ − κ*t̂*_L_. The current specification of the tradeoff model thus seems to blur the distinction between attribute-based choice and alternative-based choice. Our application of the tradeoff model would seem to do so as well, because choice will be assumed to follow a stochastic difference rule, so that the probability of choosing the high NPV option is *F*[(Σ*u*_*H*_ − Σ*u*_*L*_) − κ(*t̂*_*H*_ − *t̂*_*L*_)], which is equivalent to *F*[(Σ*u*_*H*_ − κ*t̂*_*H*_) − (Σ*u*_*L*_ − κ*t̂*_*L*_)]. However, if the tradeoff model were an alternative-based choice model, a sequence of *positive* outcomes might receive a *negative* “discounted utility,” which is formally unacceptable and psychologically implausible. This constitutes a compelling argument for maintaining that the tradeoff model *is* an attribute-based choice model.

## Candidate Models

We identified three candidate models for describing preferences for sequences: The Discounted Instantaneous Utility Model (DIUM), in Equation (1), the Sequences Model (SM), in Equations (2a) and (2b), as previously applied by Loewenstein and Prelec (1993) and Guyse et al. (2002), and the Tradeoff Model (TM), in Equations (3a) and (3b). Although Loewenstein and Prelec (1993) applied Equations (2a) and (2b) in their empirical test of SM, they also showed how discounting of individual outcome utilities could be incorporated into their model. As evident from their simulations (Loewenstein & Prelec, 1993, Figure 4), discounting interacts with the “gestalt” properties of sequence when a sequence starts in (or close to) the present (e.g., 0, 1, and 2) or when time periods are noncontiguous (e.g., 1, 2, and 10). Similar to Loewenstein and Prelec (1993), who used contiguous 1-week intervals starting in one week, we use contiguous 1-year intervals starting in one year (i.e., 1, 2, and 3). And, as Loewenstein and Prelec (1993) did, we compare the performance of SM with that of DIUM. The novelty is that we introduce TM, with its cumulative weighing of time, as a candidate model, and that we apply the candidate models to sequences of unlabeled monetary gains rather than consumption experiences.

In Experiment 1, we compare the *qualitative* predictions of the candidate models in an aggregate analysis, and, in Experiments 2 through 4, we compare their *quantitative* predictions in a Bayesian model contest in disaggregate analyses. We find that TM best describes both the “representative agent” and the majority of agents individually. In Experiment 3, we validate the Bayesian model contest against the asymmetric hidden-zero effect, which we show to be indeed driven by those participants best described by TM. We conclude with an assessment of how TM deals with remaining evidence on preferences for sequences of unlabeled monetary gains.

## Experiment 1: Concealed Zeroes Revealed

**Figure 1 fig1:**
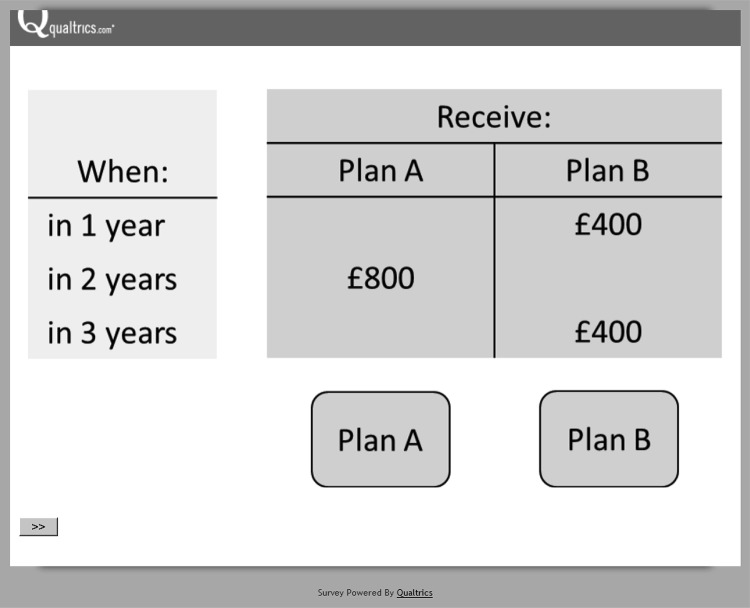
Screen shot from choice pair #11.

In Experiment 1, we examine, within the “hidden-zero paradigm,” how the preference between low and high NPV sequences is affected when the zero outcomes are revealed. We introduce two changes to elicitation of preference between the low and high NPV options. First, we use multiples of £100 below the £1,000 mark. Indeed, a partial explanation of the preference for the low NPV option might be that the number “1,000” acquires special significance among numbers that are merely multiples of “100.” We therefore use tasks in which the numbers are “300” and “600,” or “400” and “800.” Second, we present the choice pairs in delay-by-payoff matrices, with the options designated as “plans,” as shown in Figure 1 (see also Manzini et al., 2010; Gigliotti & Sopher, 1997). In our informal inquiries and initial surveys, the choice pairs were presented as follows:

Which option do you prefer?[Fig-anchor fig1]

○ Receive $500 in one year and receive $500 in three years.

○ Receive $1,000 in two years.

A partial explanation of the preference for the low NPV option might be that it “ended earlier” in a reading from left to right. Delay-by-payoff matrices resolve this problem, and ensure that, when zero outcomes are revealed, nothing changes in the presentation format, except for the appearance of the zeroes.

So the question is how preference changes when, for instance, the choice between £800 in two years and {£400 in one year, £400 in three years} is changed into a choice between {£0 in one year, £800 in two years, £0 in three years} and {£400 in one year, £0 in two years, £400 in three years}. According to the Tradeoff Model (TM), concealed zeroes are not inferred. With zero outcomes concealed, TM produces a preference for the low NPV option (£800 in two years) when duration outweighs concave utility. When, however, the low NPV option is changed into {£0 in one year, £800 in two years, £0 in three years}, year one has no weight, because zero utility accumulates until year one, year two is weighted by the accumulated utility *u*(0) + *u*(800) = *u*(800), *and so is year three*, that is, *u*(0) + *u*(800) + *u*(0) = *u*(800). Thus, compared with a situation in which zeroes are concealed, year three has weight, which increases the duration of the low NPV option. In addition, when the high NPV option is changed into {£400 in one year, £0 in two years, £400 in three years}, year one is weighted by the accumulated utility *u*(400), *year two as well*, that is, *u*(400) + *u*(0) = *u*(400), and year three is weighted by the accumulated utility *u*(400) + *u*(0) + *u*(400) = 2*u*(400). Thus, compared with a situation in which zeroes are concealed, year two has weight, which decreases the duration of the high NPV option. In sum, revealing zeroes increases the duration of the low NPV option, and decreases the duration of the high NPV option, and should thus increase preference for the high NPV option.

The Discounted Instantaneous Utility Model (DIUM) can produce a preference for the low NPV option if concave utility, or diminishing sensitivity, is replaced by convex utility, which would have to outweigh discounting. However, because zero outcomes have zero utility, and therefore do not affect the discounted utilities of the options, DIUM predicts that preference for the low NPV option should be unaffected by revealing zero outcomes.

The Sequences Model (SM) shares its prediction with either TM or DIUM, depending on the specific assumptions invoked. To reach the *TM prediction*, SM may assume that concealed zeroes are *not* inferred (the No-Zero Sequences Model, or SMNZ). It can produce a preference for the low NPV option if concave utility is replaced by convex utility (see Table 1). When the zeroes are revealed, both options become sequences that deviate from a uniform distribution, but the high NPV option deviates less from a uniform distribution than the low NPV option. Thus, if SMNZ assumes a desire for spreading, it predicts that revealing zeroes increases preference for the high NPV option, as TM does on other grounds.

To reach the *DIUM prediction*, SM may assume that concealed zeroes *are* inferred (the Zero Sequences Model, or SMZ). Both options are sequences that deviate from a uniform distribution, but the high NPV option deviates less from a uniform distribution than the low NPV option. SMZ can then produce a preference for the low NPV option if convex utility reinforces distaste for spreading or outweighs a desire for spreading, or if distaste for spreading outweighs concave utility (see Table 1). However, because zero outcomes, revealed *or* concealed, are considered anyway, SMZ predicts that preference for the low NPV option should be unaffected by revealing zero outcomes, as DIUM does on other grounds.

It thus appears that SM can have it both ways. However, this is more apparent than real, because both SMNZ and SMZ fail to accommodate the prior evidence, as discussed earlier. On the one hand, SMNZ cannot reconcile the asymmetric hidden-zero effect with the preference for decreasing over increasing sequences; on the other hand, SMZ cannot produce the asymmetric hidden-zero effect whatsoever. Because there is no parameterization of SM that accommodates prior evidence, SM fails to produce an a priori prediction for the present experiment.

### Method

#### Participants

Participants were 703 British residents, recruited through Maximiles (now rebranded to Bilendi), an Internet service that awards its members points, tradeable for goods and services, for completing surveys. The sample was 57% female, with an average age of 39. Most (86%) were employed (61% full time), and a majority (59%) had an academic degree (41% bachelors, 15% masters, 3% PhD). For 50% of the participants, the total combined income of all members of the family was between £20,000 and £59,999, for 15% it was £60,000 or more, and for 24% it was less than £20,000, with 11% preferring not to report their income.

The samples of all our experiments, including this one, were recruited in “one go,” and no participants were ever removed from the samples (except for three participants in Experiment 4, who were dropped from further analysis due to a programming error). We recruited rather large samples, because, in the subsequent modeling experiments, participants would disperse across candidate models as “their” model, and, in the present experiment, they disperse across two between-participants conditions. The largest sample is collected in Experiment 3, which is a modeling experiment with three between-participants conditions.

#### Stimuli

There were four choice pairs, as displayed in Table 2. Two pairs were compromised of a “low NPV option” and a “high NPV option” as discussed throughout the theoretical introduction, or a single dated outcome in period 2, and a sequence of two equal outcomes in periods 1 and 3 affording the same total amount. In two other pairs, the sequence was changed by increasing the outcome in period 1 and correspondingly decreasing the outcome in period 3. According to each candidate model, this should increase the preference for the sequence (and so it did).[Table-anchor tbl2]

#### Procedure

Participants were randomly assigned to either the zero condition or the no-zero condition. As mentioned earlier, choice pairs were presented in delay-by-payoff matrices, with the options designated as “plans.” The participant selected either the Plan A button or the Plan B button at the bottom of the screen, and could change his or her selection before confirming it by clicking on the arrow in the bottom-left corner of the screen. The order of the choice pairs, and the left–right order of the options in each choice pair, was randomized across participants.

### Results

The results are reported in Table 2. Majorities preferred the low NPV option to the high NPV option in the no-zero condition, and, in the zero condition, preference for the high NPV option increased, such that, now, majorities preferred *that* option. As suspected, the majorities preferring the low NPV option to the high NPV option in the no-zero condition were smaller than in our informal inquiries and initial surveys.

### Discussion

The results favored the Tradeoff Model (TM) over the Discounted Instantaneous Utility Model (DIUM), because TM correctly predicted an increased preference for the high NPV option in the zero condition, whereas DIUM predicted that preference between the high and low NPV options would not change. The evidence also favors TM over the Sequences Model (SM), because TM provides an a priori prediction of the observed change in preference, whereas SM does not; a posteriori, the results would favor SMNZ over SMZ.

## Experiment 2: No Size Fits All

The purpose of Experiment 2 is to conduct a first Bayesian contest between our candidate models. In the previous experiment, we tested the models in aggregate choice behavior, basically assuming that the average participants is representative of all participants; in this experiment, we recognize that the “representative agent” may not exist, or that the average participant is perhaps representative of many, but not all.

The models differ in their policy on zero outcomes. The Discounted Instantaneous Utility Model (DIUM) holds that it does not matter whether revealed or concealed zeroes are ignored or considered, because zero outcomes have zero utility, and therefore do not affect discounted utility. The Tradeoff Model (TM) holds that it *does* matter how zeroes are treated, and proposes that concealed zeroes are ignored, whereas revealed zeroes are considered. Finally, the Sequences Model (SM) also holds that it matters how zeroes are treated, but is silent on whether concealed zeroes are, as in the No-Zero Sequences Model (SMNZ), ignored or, as in the Zero Sequences Model (SMZ), considered. We therefore include both variants of SM as candidate models in our Bayesian contest.

### Method

#### Participants

Participants were 520 British residents, recruited through Maximiles. The sample was 61% female, with an average age of 37. The great majority (83%) were employed (61% full time), and a large majority (94%) had an academic degree (63% bachelors, 27% masters, 5% PhD). For 52% of the participants, the total combined income of all members of the family was between £20,000 and £59,999, for 20% it was £60,000 or more, and for 16% it was less than £20,000, with 13% preferring not to report their income.

#### Stimuli

Stimuli were selected from a space spanned by two options and three time periods, making for six slots. At each stimulus construction trial, each slot was filled with an amount randomly selected from among £800, £600, £400, and £200, along with a “blank” (zero) outcome, so that the outcomes were equally spaced and below the £1,000 mark. Prior criteria were that the options should not be identical, that at least one option should be a sequence, that, across options, at least one nonzero outcome occurred in each time period, that, across options and time periods, at least one zero outcome occurred, that, in no time period, the options had identical outcomes (avoiding *common consequences*), and that the options afforded the same total amount.

Selection of choice tasks took place in two runs, one searching for nondominance relations (ND), the other searching for dominance relations (D), between options affording the same total amount. Choice tasks in which Net Present Value could, depending on the discount rate, yield either preference order of the options were discarded. Choice tasks in which Net Present Value yielded the same preference order of the options as duration (computed with outcomes, rather than unknown outcome utilities) were also discarded. Run ND identified 28 choice tasks, whereas run D identified one choice task. One choice task identified by run ND, and the choice task identified by run D, included options with equal duration.

From the 29 choice tasks identified, seven with nondominance relations were dropped to reduce repetitiveness in the stimulus set, and thus fatigue and boredom on the part of the participants. These were replaced by four tasks with dominance relations: Two included a decreasing sequence and an increasing sequence affording the same total amount (one with a linear progression, the other with a nonlinear progression); the remaining two included a constant sequence and either a linearly decreasing sequence or a linearly increasing sequence affording the same total amount. These four tasks served to relate our results back to those from Manzini et al. (2010) and Gigliotti and Sopher (1997). Table 3 shows the 26 choice tasks used in Experiment 2, with the first five exhibiting dominance relations.[Table-anchor tbl3]

#### Procedure

Participants were informed they would make 26 choices, and that, for each choice, they should assume they were entitled to receive money, which they could receive according to two plans. They were told that all plans spanned a period of three consecutive years, and that they could choose the plan they liked best. Participants started with a rehearsal choice between two options exhibiting a transparent dominance relation, one yielding £200 in one year, £300 in two years, and £400 in three years, and the other yielding £100 less in each consecutive year. After making their choice, we told participants they probably noticed that the choice was very easy: One option yielded more money in each year, so surely they wanted that. We added, however, that the remaining choices would not be so easy, because the options would yield the same total amount.

#### Model estimation and evaluation

We investigated the support for each model from each individual participant using a Bayesian analysis. A Bayesian analysis allows the user to make clear and coherent statements about the direction and magnitude of the evidence in favor of each model for each individual participant. It automatically adjusts for each model’s complexity using the priors assigned to the parameters. The key measure of a Bayesian analysis is the result of the prior probability of the parameters multiplied by the likelihood of the data given those parameters, and the logic of this measure can be conveyed with a stock-market metaphor. Each model can be thought of as a stock portfolio, which starts with the same amount of money (the prior) that can be split among its companies (the parameter settings). The monetary gain from a company is the product of how much money is invested in that company and its performance, which is its share price at sale divided by the share price at purchase (the likelihood of the data given that parameter setting). The overall monetary gain from the portfolio is the sum of the results from each company in the portfolio. The narrower the range of companies in a portfolio, the more that can be invested in each company, and the larger the amount that will be gained if those companies do well. It thus becomes intuitive why, for a given model, a narrower range of prior parameter settings is better than a wider range if that those parameters perform equally well.

We present the evidence in the form of Bayes Factors (Kass & Raftery, 1995), which are statements of the relative likelihood of obtaining the data, *D*, from a pair of models, *M*_1_ and *M*_2_,

$$BF=\frac{p\left(D|M_{1}\right)}{p\left(D|M_{2}\right)},$$

where *p*(*D* | *M*) is the probability of the data given model *M.* Using Bayes Factors as a measure of the evidence easily allows readers to integrate their personal opinions about the plausibility of each model with that evidence. What a reader should believe about each model after observing the data is just their relative prior beliefs in two models *p*(*M*_1_)/*p*(*M*_*2*_) multiplied by the Bayes Factor,

$$\frac{p\left(M_{1}|D\right)}{p\left(M_{2}|D\right)}=BF\frac{p\left(M_{1}\right)}{p\left(M_{2}\right)},$$

and, when we report the posterior probabilities of models, we assume that the priors over models are always equal.

Bayes Factors are calculated by essentially finding the likelihood of the data for every possible setting of each model’s parameters, with each set of parameters weighted by its prior belief in that set of parameters, which is known as the marginal likelihood

p(D|M)=∫p(D|M,θ)p(θ|Μ)dθ,

where θ is a set of parameters for model *M*. We did not discover an analytic solution to this equation, but on visual inspection we noticed that the posterior distributions were not particularly narrow compared to the prior distributions for individual participants. This potentially allows us to sidestep complex simulation methods such as Markov chain Monte Carlo and instead use a simple Monte Carlo approximation for the marginal likelihood:

$$p\left(D|M\right)=\frac{1}{n}\overset{n}{\underset{j=1}{\sum }}p(D|M,\theta_{j}),$$

where the θ_*j*_ are samples from the prior over parameters *p*(θ | *M*). When the posterior is not too narrow compared to the prior distribution, samples drawn from the prior will often fall in regions of high posterior probability, yielding a stable estimate of the marginal likelihood. We assessed the stability of our marginal likelihoods by running our main analysis twice. The same model was found to have the highest marginal likelihood for 99.2% of the participants, and, as an absolute measure of stability, the average deviation in model posterior probability between the two runs, assuming equal priors for all models, was 0.001 across all participants and models.

Unpacking the Bayesian analysis even further, the likelihood of the data was simply the likelihood of the model producing the same set of binary choices as a participant, which can be expressed as:

log⁡[p(D|M,θ)]=∑i=1m[xilog⁡(P^i)+(1−xi)log⁡(1−P^i)],

where *x*_*i*_ acquires the value of 1 if the high NPV option is chosen from choice pair *i*, and the value of 0 otherwise, *P̂*_*i*_ is the predicted probability of choosing the high NPV option from choice pair *i* using model *M*, and *m* is the number of choice pairs. The predicted probability of choosing the high NPV option is given by the logistic distribution function:

P^i=11+exp⁡[−ε(UHNPVi−ULNPVi)],

where ε regulates the degree to which choice probabilities are determined by the model, with a value of zero indicating that choice is totally random (only “noise”). For the choice pairs in which one option dominates the other (e.g., the choice between a decreasing sequence and an increasing one yielding the same payoff), we introduce an adjustment 0 ≤ ϑ ≤ 1 for dominance detection. Imagine an avid dominance detector. If a substantive model, by its proper psychology, already predicts high probabilities that dominant options will be chosen, then the adjustment is small (ϑ close to zero); conversely, if the model predicts low probabilities that dominant options will be chosen, then the adjustment is large (ϑ close to one). The predicted probability of choosing the dominant over the dominated option is given as follows:

P^i=ϑ+(1−ϑ)11+exp⁡[−ε(UHNPVi−ULNPVi)],

where *P̂*_*i*_ is the probability of choosing the dominant option, as the combined result of the model and dominance detection.

The weighing parameters of TM and SM were transformed into relative weights, thus encouraging an independent role of ε in the distribution function and making values easier to interpret. For TM, the accumulated utilities were weighted by 1/(1 + κ), and duration by κ* = κ/(1 + κ). Analogously, for SM, the accumulated utilities were weighted by 1/(1 + |β| + |σ|), the improvement scores by β* = β/(1 + |β| + |σ|), and the spreading scores by σ* = σ/(1 + |β| + |σ|).

For all three models, we specified the utility function as the power function *u*(*x*) = *x*^γ^ (e.g., Abdellaoui et al., 2010; Dai & Busemeyer, 2014) with 0 ≤ γ ≤ 5, and assumed a uniform prior distribution over its entire range.^6^ For δ and ϑ, we assumed the same uniform prior between zero and one. For the regulation parameter ε, we assumed a uniform prior over the range of 0 to 100. For TM, we specified, as mentioned in Footnote 5, the time-weighing function as the identity function *w*(*t̂*) = *t̂*, and let κ have a uniform prior from 0 to 100. For SM, we allowed participants to either like (β > 0) or dislike (β < 0) improvement, and to either like (σ > 0) or dislike (σ < 0) spreading. Both parameters were given uniform prior distributions from −100 to 100. The priors for κ, β, and σ cover the entire range from exclusive weight of accumulated utilities and (practically) exclusive weight of the model components associated with κ, β, and σ. Additional analyses with different sets of prior distributions produced qualitatively the same headline results.^7^

#### Model recovery

We will estimate models with four to five parameters from individual choice behavior in 26 binary choice tasks. Because information is relatively scarce, one might wonder whether the information is *sufficient* at all to identify the “right” model. We therefore undertook a model-recovery exercise. At each step in the exercise, a parameter set was drawn from the prior of the *generating model*, and one individual’s worth of data were generated from those parameters. The marginal likelihoods were found for the data, and the winning model was selected, thus treated as the *recovered model*. This procedure was repeated 1,000 times for each of the models taking a turn as the generating model. The results are reported in Table 4. The rate of model recovery is high: On more than 87% of the 4,000 occasions, the generating model was successfully recovered. We therefore conclude that the information provided by our experimental paradigm is sufficient to identify the “right” model.[Table-anchor tbl4]

#### Parameter estimation

Although our model recovery exercise demonstrated that we could identify the best model using individual data, the best parameters for individuals were not as easily identifiable. As shown in the supplemental material, the posterior distributions over parameters for each individual were broad and sometimes multimodal, so any single point does not provide a good summary of these distributions.^8^ Instead, we ran a secondary analysis to identify the best parameters. Participants were split into groups according to which model produced the highest marginal likelihood for their individual data. The data of each group were aggregated, and the aggregate data were used to determine the posterior distribution over the parameters for the model associated with that group. Thus, the posterior distribution over parameters for each model was determined using a different group of participants.

We needed a new approximation to calculate the posterior distributions, because the simple approximation we used in the individual-level analyses does not work well when the posterior distributions are narrow. Thus, in the group-level analyses, we used the Metropolis-Hastings algorithm, which is a Markov chain Monte Carlo method that starts in a particular state, and produces a chain of states that are used as samples from the posterior distribution over parameters. We started each chain using a random draw from the prior distribution and collected one million states for each chain, discarding the first half so as to remove the influence of the arbitrary starting state. During this “burn-in period,” we continually recalibrated the proposal distribution, that is, the distribution that determined what the next state could be, by setting the covariance of the Gaussian proposal distribution to a constant multiple of the sample covariance of a recent set of states, plus a small additive diagonal factor, to ensure the covariance matrix did not collapse to zero (Rosenthal, 2011). The samples were summarized by finding both the median of each parameter and the Highest Density Interval (HDI), which was the single shortest continuous interval that contained 95% of the posterior distribution. Across two runs of this analysis using random starting points for the sampler, we found consistent medians and HDIs.

#### Main contest and parallel contest

We run a “main contest” and a “parallel contest” between models. The *main contest* is between complete specifications of the four substantive psychological models, that is, TM, DIUM, SMNZ, and SMZ. The *parallel contest* is between the same models, and reduced forms of these models, either by setting ϑ to zero, or by setting ε to zero, or by setting both ϑ and ε to zero. We next explain the logic behind this.

In the main contest, each candidate model includes the parameter ϑ, an adjustment for dominance detection. This parameter increases the probability of choosing the dominant option; when it is zero, an individual has no stronger proclivity toward the dominant option than is given by the model. The adjustment parameter, however, does make the models less parsimonious, so that, when dominance detection is modest, models without the adjustment parameter might, by their parsimony, fare better. In the Appendix, we specify all four models with and without the adjustment parameter, and report the relative performance of the models accordingly, but we are generally interested in how the models perform regardless of whether increased parsimony pays off or not, just as we are interested, in the main contest, in how the models perform regardless of whether ϑ is high or low.

Another aspect of the main contest is that we only include substantive psychological models as candidates. Each model, however, includes, as in modeling exercises elsewhere, the parameter ε, which regulates the degree to which choice probabilities are determined by the model; when it is zero, choice is totally random. As choice becomes increasingly random, a *Random-choice model* (RM) might, by its parsimony, fare better than any substantive psychological model. In the Appendix, we include RM as a fifth model. When specified without the parameter ϑ, RM holds that *any* choice is random; when specified with this parameter, RM holds that choice is random *unless* one option dominates another, in which case choice of the dominant option may become more likely. In our discussion of the results from the main contest, we will refer to the results from the parallel contest.

#### Descriptive accuracy

Although Bayes factors allow us to assess how well the models perform *relative to* one another, they do not provide an absolute standard against which to measure the performance of each model. So, in addition to the Bayes factors, we calculated Bayesian *p* values, which measure descriptive accuracy by evaluating the posterior predictions of a model against the observed data (see Meng, 1994; Myung, Karabatsos, & Iverson, 2005). In particular, a Bayesian *p* value is the probability that the discrepancy between the model and the posterior predicted data is greater than the discrepancy between the model and the observed data, *p*{*T*(*D*^rep^) > *T*(*D*) | *D*, *M*}, with values below the conventional threshold of .05 meaning that it is unlikely that the observed data would have arisen from a particular model, or that the model does not fit well.

A discrepancy function *T* must be chosen to evaluate the “closeness” of the data to the model. While the Pearson chi-square discrepancy function is a common choice (Myung et al., 2005), the predicted probabilities from our model were often within machine precision of zero or one, which makes that discrepancy function difficult to work with. Instead, we chose the sum-of-squares discrepancy function

$$T\left(D;\;\theta ,M\right)=\underset{i}{\sum }{\left[d_{i}-p\left(d_{i}|\theta ,M\right)\right]}^{2},$$

where *d*_*i*_ are the data from each individual choice *i*. Using the discrepancy function *T*, the formula for the Bayesian *p* value is as follows:

$$p\left\{T\left(D^{rep}\right)>T\left(D\right)|D,M\right\}=\int \frac{1/n\sum_{k}^{n}\left[p\left(D_{k}^{rep}|\theta ,M\right)I\left(T\left(D_{k}^{rep};\;\theta ,M\right)>T\left(D;\theta ,M\right)\right)\right]P\left(D|\theta ,M\right)p\left(\theta |M\right)}{p\left(D|M\right)}d\theta ,$$

where *I* is an indicator function that is 1 if the argument is true and 0 if it is false.

As with the Bayes factors, the Bayesian *p* value needs to be approximated. To do this, we follow a procedure similar to that for approximating the Bayes factors for individual participants. Specifically, we drew samples from *P*(θ | *M*), and, for each sample, we generated a single *D*^rep^ by sampling choices using the choice probabilities predicted by model *M* with parameters θ. We calculated the discrepancies *T*(*D*) and *T*(*D*^rep^) using the predictions generated by model *M* using parameters θ. If, while iterating over the samples, *T*(*D*^rep^) > *T*(*D*), we added *p*(*D* | θ, *M*)/*p*(*D* | *M*) to the Bayesian *p* value, otherwise we added nothing.

### Results

Table 3 shows the percentages of participants choosing the high NPV option among those supporting the respective models in the Bayesian analyses, to be reported shortly, and among all those participating in the experiment. The results for choice pairs #2 to #4 confirm the results from Manzini et al. (2010) and Gigliotti and Sopher (1997): Participants preferred a constant sequence to an increasing one, and they preferred a decreasing sequence to an increasing one, but their preference between a decreasing sequence and a constant one was basically undecided.

**Figure 2 fig2:**
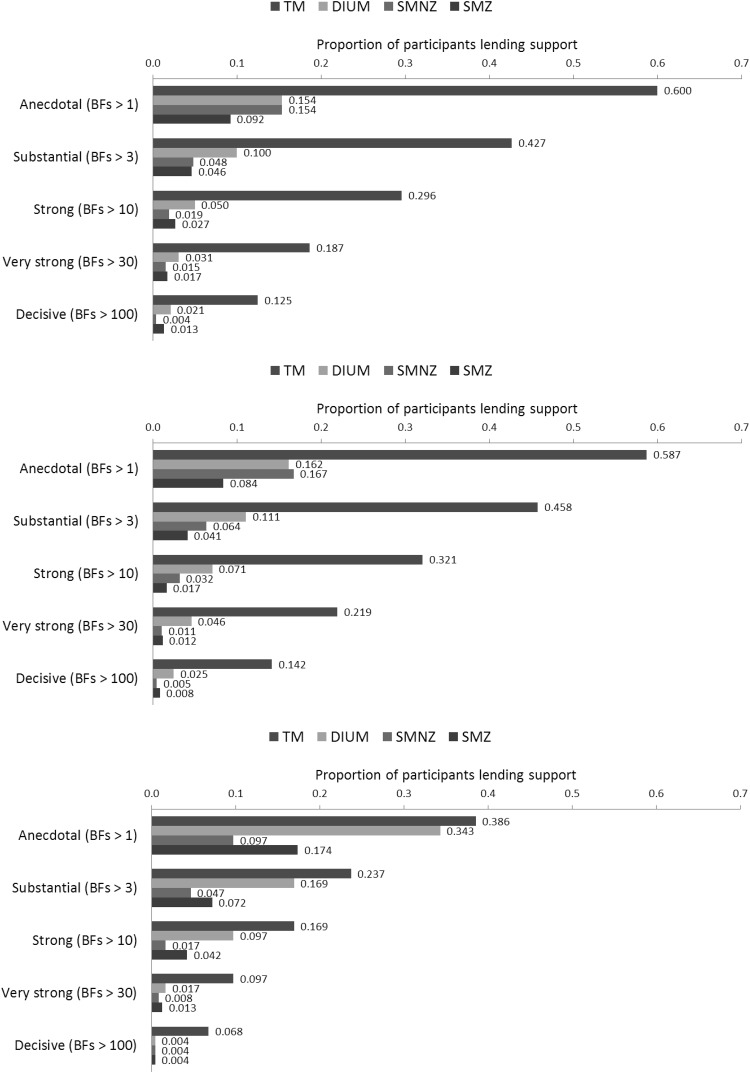
Proportions of participants (above bars) lending increasingly stronger support for candidate models in Experiment 2 (top panel), Experiment 3 (center panel), and Experiment 4 (bottom panel). BFs = Bayes Factors; TM = Tradeoff Model; DIUM = Discounted Instantaneous Utility Model; SMNZ = No-Zero Sequences Model; SMZ = Zero Sequences Model.

Figure 2 depicts the proportions of participants whose support for one model over *all* other candidate models in the main contest is, by informal rules of thumb (Jeffreys, 1961; Wetzels et al., 2011), anecdotal (both Bayes Factors [BFs] greater than one), substantial (both BFs greater than three), strong (both BFs greater than 10), very strong (both BFs greater than 30), and decisive (both BFs greater than 100). TM receives the most support; for example, it receives substantial support from more than 40% of the participants, whereas the other candidate models together receive substantial support from fewer than 20% of the participants.[Fig-anchor fig2]

**Figure A1 fig8:**
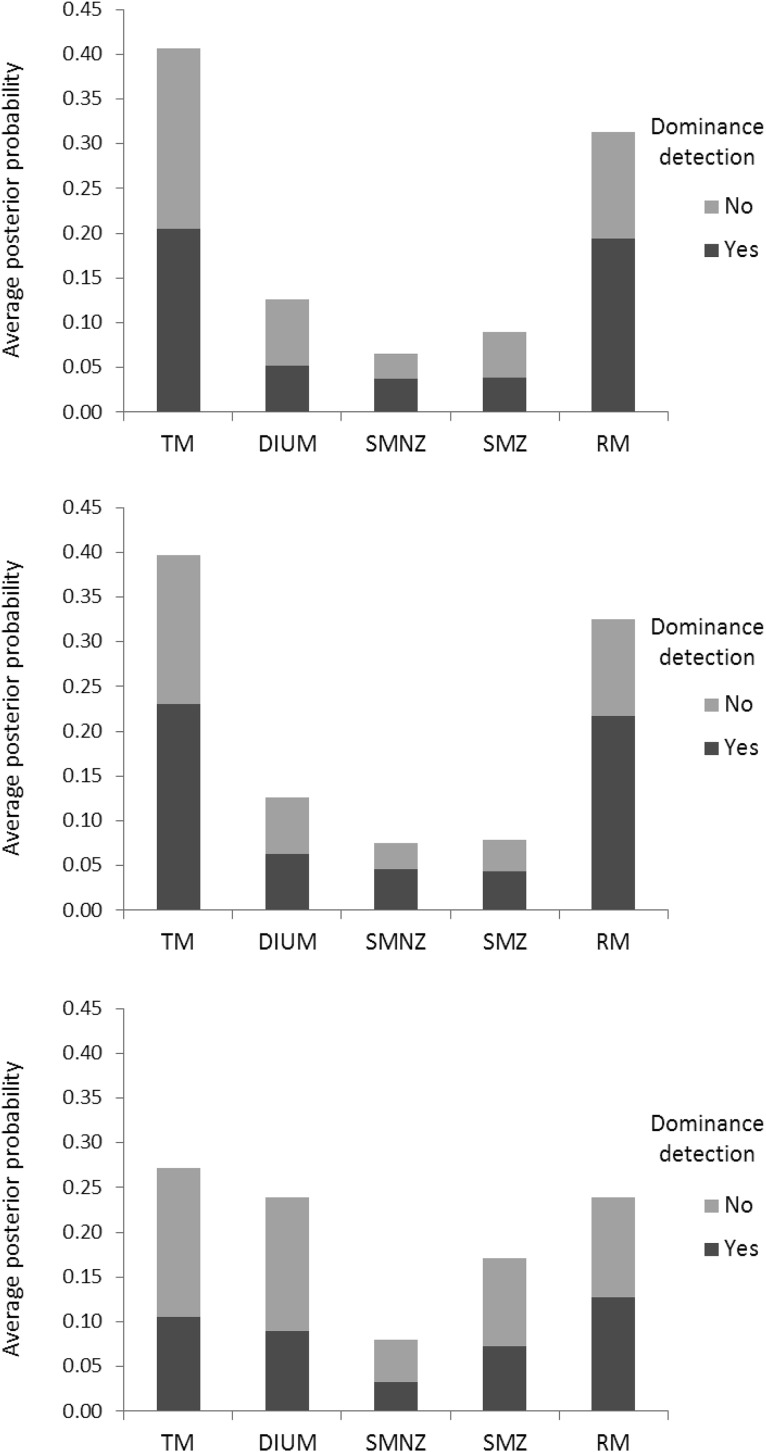
Posterior probabilities for the parallel model contest, averaged across participants, in Experiment 2 (top panel), Experiment 3 (center panel), and Experiment 4 (bottom panel). TM = Tradeoff Model; DIUM = Discounted Instantaneous Utility Model; SMNZ = No-Zero Sequences Model; SMZ = Zero Sequences Model; RM = Random-choice Model.

For a strictly quantitative assessment of relative performance (without reliance on rather arbitrary rules of thumb), we averaged posterior probabilities (the ratios between which are, under the assumption of equal priors, the Bayes Factors) of the respective models across participants, yielding .54 for TM, .17 for DIUM, .16 for SMNZ, and .12 for SMZ. Figure A1 depicts the average posterior probabilities of the five candidate models (with or without adjustment for dominance detection) in the parallel contest. TM performs best, and RM performs second best, suggesting that participants engaged in a fair amount of guessing. Moreover, RM obtains more support *with* adjustment for dominance detection than *without*, suggesting that guessing was not just a matter of distraction or carelessness, but rather of not knowing what to do in the absence of dominance relations. Table A1 shows how the supporters of the four candidate models in the main contest distribute across the five candidate models in the parallel contest. The participants either stay with their model or switch to RM. In raw numbers, most switches are from TM to RM, but that is because there are so many more supporters of TM; it is, actually, a *minority* that switches from TM to RM, just as we see minorities switching from DIUM to RM, and from SMZ to RM. Among supporters of SMNZ, on the other hand, we see a true exodus to RM, with more than 70% of the supporters taking part in it, meaning that SMNZ is particularly prone to attracting individuals with “noisy” choice behavior.

**Figure 3 fig3:**
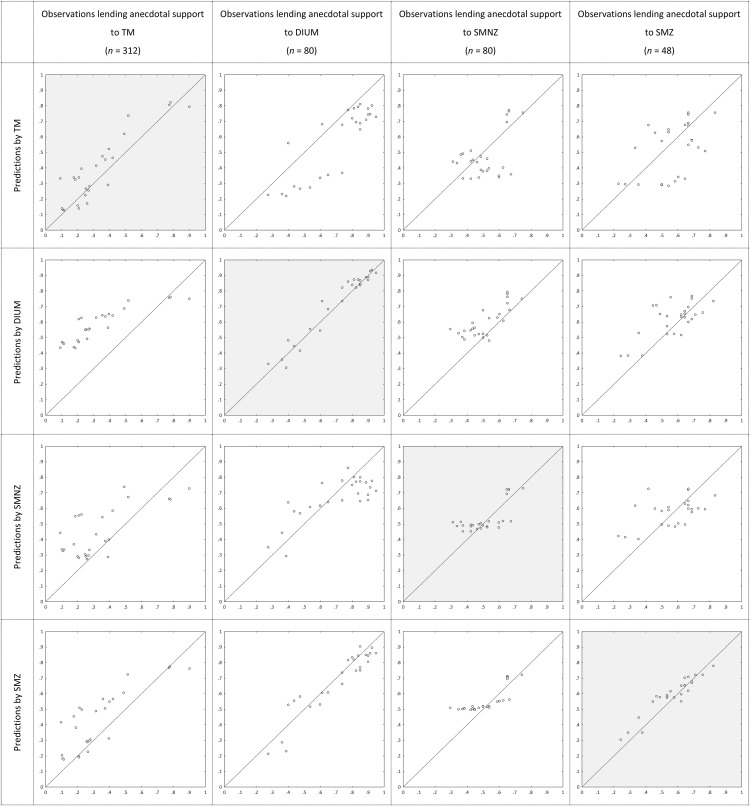
Observed proportions of participants choosing the high NPV option among those lending anecdotal support to the respective models (horizontal), plotted against the average predicted probabilities of choosing the high NPV option generated by the models (vertical), in Experiment 2. TM = Tradeoff Model; DIUM = Discounted Instantaneous Utility Model; SMNZ = No-Zero Sequences Model; SMZ = Zero Sequences Model.

Bayes Factors and posterior probabilities inform us about the relative performance of the models, not about how well each model describes the choices of those best described by it. Figure 3 depicts the observed proportions of participants choosing the high NPV option among those lending anecdotal support to the respective models in the main contest (horizontal), plotted against the average predicted probabilities of choosing the high NPV option generated by the models (vertical). Not surprisingly, each model describes the choices of *its* supporters best. Interesting is how TM and DIUM describe the supporters of one another. Most choice pairs, except for #1 to #4, were selected for generating an ordinal contrast between preference entailed by duration (TM, with linear utility) and preference entailed by Net Present Value (DIUM, with linear utility). Table 3 shows that supporters of TM indeed mostly prefer the low NPV option, whereas supporters of DIUM mostly prefer the high NPV option, and Figure 3 shows that TM tends to underpredict high NPV choices among supporters of DIUM, whereas DIUM tends to overpredict high NPV choices among supporters of TM (as the sequences model do as well).[Fig-anchor fig3]

For a quantitative assessment of descriptive accuracy, Table A2 shows the proportions of Bayesian *p* values lower than .05 among participants, lending anecdotal support to the respective models in the parallel contest. Not counting RM without dominance detection, which holds that each choice is a coin toss, and therefore receives a Bayesian *p* value of zero by definition, the models fit well overall, with TM, whether with or without adjustment for dominance detection, showing no *p* values lower than .05. When specified with adjustment for dominance detection, SMZ also does not show *p* values lower than .05, and nor does RM, which means that, also in terms of descriptive accuracy, a combination of guessing and dominance detection captures well what a portion of our participants is doing. Across specifications, DIUM is least accurate, and SMNZ second-least accurate.

Table 5 provides, for each model, the medians of the parameter posteriors, along with the lower and upper bounds of the 95% Highest Density Intervals (HDIs). In all four models, the utility function is concave (i.e., γ < 1), thus exhibiting diminishing sensitivity. This argues against all explanations of prior evidence that resorted to convex utility.[Table-anchor tbl5]

Concavity of the utility function is least pronounced among supporters of TM, and *most* pronounced among supporters SMNZ and SMZ, which may seem counterintuitive, since the sequences models include a desire for spreading (σ > 0) as a separate component, which would seem to relieve the utility function from capturing any desire for spreading through concave utility (γ < 1). This would be the case, however, if the spreading component and concave utility would both favor an option for approximating a uniform distribution. But this is not what the spreading component does: It *disfavors* an option for *departing from* a uniform distribution. The result is a hydraulic relationship between the spreading component, which disfavors an option for departing from a uniform distribution, and concave utility, which *does* favor an option for approximating a uniform distribution. Thus, not even in the sequences models, γ provides a “pure” assessment of diminishing sensitivity, independently from a desire for spreading.

While the HDIs for σ are wide, so is the HDI for κ in TM. This is because the utility function of TM exhibits only slight concavity (γ near to 1) over the outcomes of sequences that, in any given choice pair, total the same amount, so that differences between the accumulated utilities of the sequences change little across choice pairs. Therefore, TM explains choice behavior mostly on the basis of differences between the durations of the sequences, which creates redundancy between κ and ε, in that higher values of κ can more easily be compensated by lower values of ε. For instance, if concavity vanishes, and the differences between the accumulated utilities of the sequences are therefore always zero, an increase of κ from 18.73 (lower bound of the HDI) to 100.00 (upper bound of the HDI), which is an increase of κ* (relative weight) from .9493 to .9901, can be compensated by a decrease of ε from 4.2350 to 4.0605 (taken at equal distance from the median). In comparison with the narrow range of κ*, we obtain, for the sequences models, wider ranges of β* (between −.79 and −.46 for SMNZ, between −.51 and .06 for SMZ) and σ* (between −.20 and .87 for SMNZ, between .48 and .93 for SMZ); yet, it can be concluded that these models tend to converge on distaste for improvement and a desire for spreading.

### Discussion

In a Bayesian contest among candidate models at the disaggregate level, the Tradeoff Model (TM) came out as best describing most participants. In a parallel contest, RM came out as the strongest competitor to TM, especially when, alongside the choice processes described by these models, dominance detection was taken into account. The overall picture is that *weighing cumulative utility against the duration of utility accumulation, guessing, and dominance detection go a long way in explaining the behavior of our participants*.

In Experiment 1, we compared the competing models with respect to their predictions about the effects of revealing zero outcomes on aggregate choice behavior, and, in Experiment 2, we compared the models with respect to their ability to describe individual choice behavior. In the next experiment, we validate our disaggregate analysis against the asymmetric hidden-zero effect, one thread of evidence from the theoretical introduction.

## Experiment 3: The Cumulative Weight of Nothing

We expect the asymmetric hidden-zero effect to be driven by those supporting the Tradeoff Model (TM), because the later zero added to the smaller-sooner outcome (changing *SS* into *SS*_0_) has cumulative weight, whereas the sooner zero added to the larger-later outcome (changing *LL* into *LL*_0_) has not. In contrast, we expect those supporting the Discounted Instantaneous Utility Model (DIUM) to be immune to zero outcomes, because zero outcomes have zero utilities, and thus leave the discounted utilities of sequences unchanged.

Drawing on the analysis in Table 1, the No-Zero Sequences Model (SMNZ) would expect the later zero to have an effect as far as a desire for improvement reinforces a desire for spreading, and would expect the sooner zero to have no effect at all if the desire for improvement precisely offsets the desire for spreading. SMNZ would expect a *symmetric* hidden-zero effect if the desire for improvement eclipses the desire for spreading. The Zero Sequences Model (SMZ), however, expects *any* hidden-zero effect to be symmetrical, because revealing one zero means that the other zero will be inferred.

It should be emphasized that Bayesian analysis does not include the hidden-zero tasks serving to evaluate the hidden-zero effect, so that the above derivations are truly *predictions*.

### Method

#### Participants

Participants were 848 British residents, recruited through Maximiles. The sample was 54% female, with an average age of 42. Most (84%) were employed (61% full time), and a large majority (91%) had an academic degree (61% bachelors, 25% masters, 6% PhD). For 50% of the participants, the total combined income of all members of the family was between £20,000 and £59,999, for 23% it was £60,000 or more, and for 16% it was less than £20,000, with 11% preferring not to report their income. Overall, the sample characteristics were very similar to those in Experiment 2.

#### Stimuli

The stimuli were the same as in Experiment 2, except that one set of three choice tasks was added. These were choices between a smaller-sooner and a larger-later outcome, with the smaller-sooner outcome being £150, £300, or £450 today, and the larger-later outcome being £200, £400, and £600, respectively, in 1 year. Thus, the interest rate offered was held constant at 33.33%. Either no zero outcomes were stated, or only the sooner zero was stated, or only the later zero was stated.

#### Procedure

The three hidden-zero tasks were presented first. Participants were asked to imagine for these three decisions that they were entitled to receive money, that they would be given two options to choose from, and that, in each case, they would have to select the option they preferred. The order of the choice tasks was randomized across participants. Furthermore, the statement of zero outcomes was manipulated between participants. As in past research on the hidden-zero effect, the options were written in two consecutive lines, with *LL* (or *LL*_0_) always following *SS* (or *SS*_0_). The rest of the procedure was identical to that in Experiment 2.

### Results

#### Bayesian model contests

**Figure 4 fig4:**
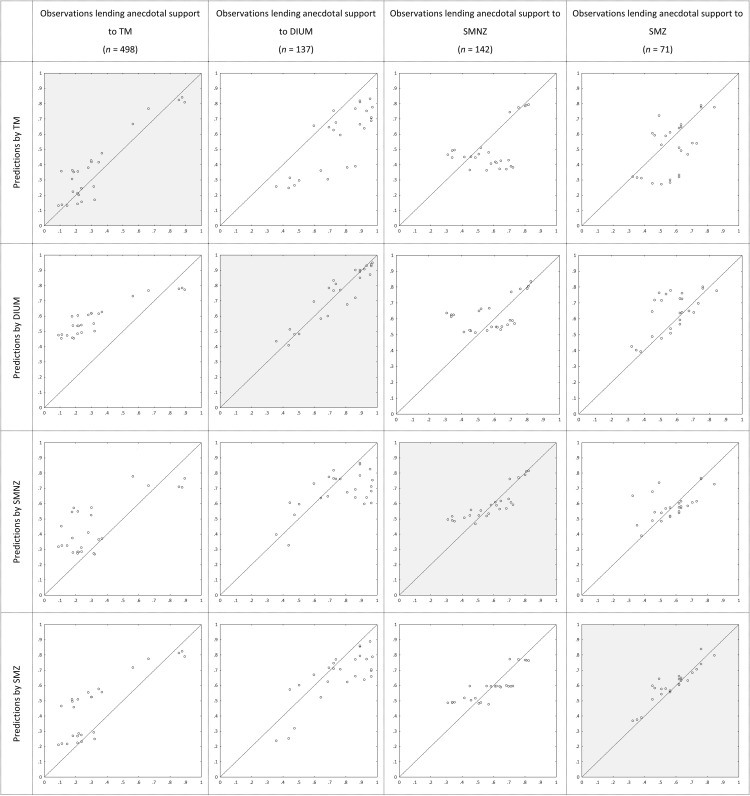
Observed proportions of participants choosing the high NPV option among those lending anecdotal support to the respective models (horizontal), plotted against the average predicted probabilities of choosing the high NPV option generated by the models (vertical), in Experiment 3. TM = Tradeoff Model; DIUM = Discounted Instantaneous Utility Model; SMNZ = No-Zero Sequences Model; SMZ = Zero Sequences Model.

Figure 2 depicts the proportions of participants lending progressively more convincing support for one model over all other candidate models in the main contest. By the informal rules of thumb, TM again receives the most support; for example, it receives more than twice as much substantial support as the other candidate models together. On average, posterior probabilities were .53 for TM, .18 for DIUM, .17 for SMNZ, and .12 for SMZ, almost identical to those in Experiment 2. Figure A1 depicts the average posterior probabilities of the five candidate models in the parallel contest, also almost identical to those in Experiment 2. Table A1 shows how the supporters of the four candidate models in the main contest distribute across the five candidate models in the parallel contest, and they essentially do so in the same way as in Experiment 2. Figure 4 depicts the observed proportions of participants choosing the high NPV option among those lending anecdotal support to the respective models in the main contest, plotted against the average predicted probabilities of choosing the high NPV option generated by the models, and Table A2 shows the proportions of Bayesian *p* values lower than .05 among those participants lending anecdotal support to the respective models in the parallel contest. It is safe to conclude that, both quantitatively and qualitatively, the results show a strong resemblance to those from Experiment 2.[Fig-anchor fig4]

#### External validation against the asymmetric hidden-zero effect

**Figure 5 fig5:**
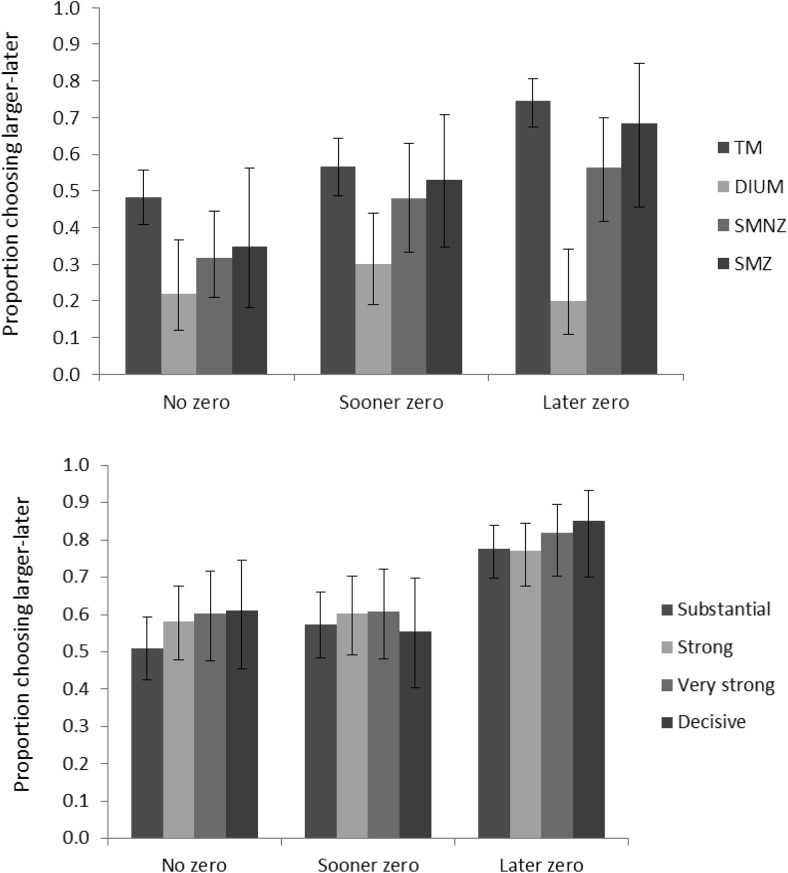
The effect of zero outcomes among those providing anecdotal support for the candidate models (top panel), and among those providing increasingly stronger support for the Tradeoff Model (bottom panel), in Experiment 3. TM = Tradeoff Model; DIUM = Discounted Instantaneous Utility Model; SMNZ = No-Zero Sequences Model; SMZ = Zero Sequences Model.

Concerning the three hidden-zero tasks, we computed, for each participant, the proportion of choices favoring the larger-later outcome (a measure of patience). The top panel of Figure 5 shows the results for those providing anecdotal support for the respective models. As expected, DIUM supporters are not affected by zero outcomes. In contrast, those supporting TM, SMNZ, and SMZ *are* affected by them, but more so by the later zero than by the sooner one. Let *p*_*L*_, *p*_*S*_, and *p*_*N*_ be the average proportions of patient choices in the later-zero, sooner-zero, and no-zero conditions, respectively. A measure of the asymmetry of the hidden-zero effect is then (*p*_*L*_ − *p*_*N*_) − (*p*_*S*_ − *p*_*N*_) = *p*_*L*_ − *p*_*S*_, where a *strictly* asymmetric hidden-zero effect is *p*_*L*_ − *p*_*N*_ > 0 and *p*_*S*_ − *p*_*N*_ = 0. The asymmetry is more pronounced among those supporting TM, *p*_*L*_ − *p*_*S*_ = .18, 95% CI [.08; .28], than among those supporting SMNZ, *p*_*L*_ − *p*_*S*_ = .08, 95% CI [−.13; .29], and SMZ, *p*_*L*_ − *p*_*S*_ = .15, 95% CI [−.13; .43], but those supporting TM do seem to be affected somewhat by the sooner zero, *p*_*S*_ − *p*_*N*_ = .09, 95% CI [−.02; .19]. We must realize, however, that we are including all participants who provide *anecdotal* support for a model, several of whom do not lend *convincing* support to it. The bottom panel of Figure 5 therefore shows the results among those lending progressively more convincing support to TM. We see the asymmetric hidden-zero effect that we expected: An effect of the later zero, but no effect of the sooner one, among those lending substantial support, *p*_*L*_ − *p*_*N*_ = .27, 95% CI [.16; .38], *p*_*S*_ − *p*_*N*_ = .06, 95% CI [−.06; .19], among those lending strong support, *p*_*L*_ − *p*_*N*_ = .19, 95% CI [.06; .32], *p*_*S*_ − *p*_*N*_ = .02, 95% CI [−.13; .17], among those lending very strong support, *p*_*L*_ − *p*_*N*_ = .24, 95% CI [.09; .39], *p*_*S*_ − *p*_*N*_ = .01, 95% CI [−.17; .18], and among those lending decisive support, *p*_*L*_ − *p*_*N*_ = .24, 95% CI [.05; .43], *p*_*S*_ − *p*_*N*_ = −.05, 95% CI [−.27; .16].[Fig-anchor fig5]

### Discussion

The results from Experiment 3 confirm that the asymmetric hidden-zero effect is driven by those supporting the Tradeoff Model (TM): Among those individuals, the later zero has an effect but the sooner zero has not, whereas, among those supporting the Discounted Instantaneous Utility Model (DIUM), neither zero has an effect. This constitutes an external validation of both models, and, for TM, it corroborates the cumulative weighing of time.

The Sequences Model (SM), however, fails the test of external validity. Among supporters of both variants, there were indications that *both* zeroes produced a rise in patience, but that the later zero produced a greater rise in patience than the sooner zero. For the No-Zero Sequences Model (SMNZ), this would mean that a desire for improvement outweighs a desire for spreading, whereas we see in Table 5 that *distaste* for improvement outweighed a desire for spreading. For the Zero Sequences Model (SMZ), any asymmetry of the hidden-zero effect is at odds with its assumption that, once a decision involves outcome sequences, concealed zeroes are inferred by the decision maker.

As to the Bayesian model contest itself, the overall picture that emerges from it is the same as in Experiment 2: *Weighing cumulative utility against the duration of utility accumulation, guessing, and dominance detection go a long way in explaining the behavior of our participants*.

## Experiment 4: Top It Up

In Experiment 4 we conducted an incentivized version of our previous experiments. This was done at the University of Warwick, with participants being entered into a lottery where they had a 1:40 chance that one of their choices would be paid out for real. Earnings were in the form of “Eating at Warwick” top ups (see http://www2.warwick.ac.uk/services/retail/eating), which can be added to the ID cards of every student and staff member at the University of Warwick. Primarily, as the name implies, the card is used for eating at university restaurants and coffee shops, but it can also be used to purchase groceries, theater tickets (at the Warwick Arts Center), and books (at the university bookstore). These cards can be preloaded by anyone who has the person’s student or staff number. For instance, parents can prepay for their child’s food by topping up their cards from home. These prepayments take effect immediately.

### Method

#### Participants and payments

Participants were 239 students from the University of Warwick, Coventry, Great Britain. (Three participants were dropped from further analysis due to a programming error.) They were recruited on the University of Warwick’s research participation system (SONA), and participated in exchange for a promise that six participants would receive one of their choices, worth up to £80, paid for real. On average, the sample was 19 years of age, with 59% being female.

#### Stimuli

The outcomes from Experiments 2 and 3 were divided by 20, yielding outcomes of £10, £20, £30, and £40. Real total payoffs therefore ranged from £20 (if choice pair #12 were selected for payout) to £80 (if choice pair #5 were selected for payout). Furthermore, the delays from the previous experiments were divided by six, yielding delays of two, four, and six months. All else about the stimuli remained the same.

#### Procedure

The experiment was run online, on November 5, 2014. In the introduction to the online questionnaire, participants were informed that they would make 26 decisions between payment plans, and that the payment plans were Eating at Warwick top ups on their student card. They were told that every plan would specify a top up amount to be paid in two months (January 5, 2015), in four months (March 5, 2015), and in six months (May 5, 2015), and that, for every choice they made, there would be a chance it would be paid for real. They were further told that six participants would be chosen at random, and that the chosen participants would actually be given one of their preferred plans. The instruction then read: “To enable us to enter you in the draw please provide your email address.” They were informed that everybody would receive a number upon completion of the experiment, and that the experimenter would randomly select six of these numbers; if their number was selected, one of the choices they made would be picked at random and paid out for real. They were further informed that everyone would receive an email listing the randomly selected numbers, and that the participants with these numbers would be sent a separate email stating what they would receive and when.

The next page of the instruction presented a rehearsal task, analogous to the one used in Experiments 2 and 3. After the rehearsal trial, participants were reminded that each option they chose might be paid out for real, so that they would have to choose carefully.

### Results

**Figure 6 fig6:**
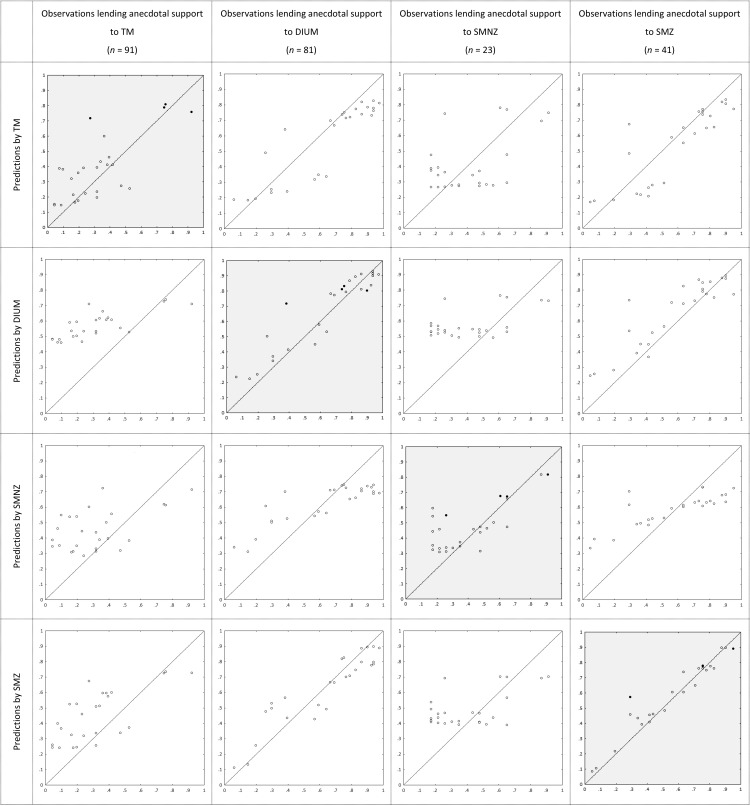
Observed proportions of participants choosing the high NPV option among those lending anecdotal support to the respective models (horizontal), plotted against the average predicted probabilities of choosing the high NPV option generated by the models (vertical), in Experiment 4. TM = Tradeoff Model; DIUM = Discounted Instantaneous Utility Model; SMNZ = No-Zero Sequences Model; SMZ = Zero Sequences Model.

Figure 2 depicts the proportions of participants lending progressively more convincing support for one model over all other candidate models in the main contests. By the informal rules of thumb, TM is still the best performing model, but it faces fiercer competition from SMZ, and especially from DIUM; for example, it receives 6.8% more substantial support than DIUM, its closest competitor, but it receives 5.1% less substantial support than DIUM and the other candidate models together. On average, the posterior probabilities were .36 for TM, .29 for DIUM, .13 for SMNZ, and .22 for SMZ, also attesting to the rise of SMZ, and especially of DIUM. Figure A1 shows the average posterior probabilities of the five candidate models in the parallel contest, and a stiff competition between the models is evident, with RM and DIUM now sharing the second place, and each coming right after TM, which is still in the first place. All four substantive psychological models obtained more support *with* adjustment for dominance detection than *without*. Table A1 shows how the supporters of the four candidate models in the main contest distribute across the five candidate models in the parallel contest, and they basically do so as in the same way as in Experiments 2 and 3, except that there is less migration from SMNZ to RM. Figure 6 depicts the observed proportions of participants choosing the high NPV option among those lending anecdotal support to the respective models in the main contest, plotted against the average predicted probabilities of choosing the high NPV option generated by the models. There is more dispersion around the identity line than in Experiments 2 and 3, especially for TM and SMNZ. However, Table A2 shows that, when specified with adjustment for dominance detection, all models in the parallel contest, including TM and SMNZ, hit the 0% rate of Bayesian *p* values lower than .05. TM remained at the 0% rate when specified without adjustment for dominance detection, as did DIUM, meaning that, also in terms of descriptive accuracy, DIUM performed better than in Experiments 2 and 3.[Fig-anchor fig6]

Table 5 provides, for each model, the medians of the parameter posteriors, along with the lower and upper bounds of the 95% Highest Density Intervals (HDIs). First, across the board, but especially among those supporting SMNZ and SMZ, choice behavior is determined more by the respective models (higher ε), meaning that the choice behavior is less “noisy.” Second, while adjustment for dominance detection (ϑ) was, among supporters of DIUM, already low in previous experiments, it dropped, among supporters of TM, SMNZ, and SMZ, to low levels as well, with the lowest level reached by TM. This is consistent with the fact that, in the parallel contest, the models benefited more than in Experiments 2 and 3 from an increased parsimony when the adjustment parameter was suppressed. The reason why ϑ goes to zero is a markedly reduced probability of choosing the decreasing sequence instead of the constant sequence it dominates (analogous to choice pair #3 in Table 3). Figure 6 plots the four choice pairs with dominance relations as solid circles. The choice pair involving the decreasing and constant sequence is the one far above the identity line, whereas the other three choice pairs involving dominance relations are reasonably close to that line. Thus, one does not want adjustment for dominance detection to make matters worse.

### Discussion

The results from Experiment 4, which introduced incentives, differ somewhat from those in Experiments 2 and 3. Most importantly, we saw an increase in support for the Zero Sequences Model (SMZ), which, as shown in Footnote 2, is the holistic analogue of atomistic discounting, and especially the Discounted Instantaneous Utility Model (DIUM). Why this change, and what to conclude from it?

The differences between Experiment 4 and the previous experiments include smaller outcomes, shorter delays, student population, less abstract outcomes, and the use of nonhypothetical payoffs. All of these changes, especially the use of less abstract outcomes, may have introduced motives in addition to those operating in the previous experiments. Importantly, payment in the “Eating at Warwick” currency may have promoted a discounting of individual outcome utilities, because later payoffs occurred close to the summer break, when many students might be going home or even leaving the university altogether, and would no longer be using their Eating at Warwick card. We thus ascribe the rise in support for DIUM to *a trimmed time horizon* in the mind of the decision makers. If correct, this explanation offers an important insight into the psychology of preferences for sequences, and into the variability of relative model performance across settings.

The main goal of all the changes was to provide nonhypothetical payoffs. This might bring decisions closer to the economic model and reduce errors in responding (e.g., Camerer & Hogarth, 1999; Hertwig & Ortmann, 2001). We indeed found choice behavior to be less “noisy.” We also found a surge in support for DIUM. Because DIUM is similar to the NPV model, which is the standard of economic rationality, one might infer that decisions were indeed brought “closer to the economic model.” However, the NPV model operates on money, whereas DIUM operates on the *instantaneous utility* of money, as a result of which it actually moves *away from* economic rationality by allowing for violations of dominance. Thus, increasing support for DIUM cannot be automatically taken to be increasing support for “the economic model.” Moreover, *even if* DIUM were taken to be the standard of economic rationality, it would definitely be less plausible that the incentives “magically” led to a discounting of individual outcome utilities than that the incentives led to a trimmed time horizon, which, in turn, led to a discounting of individual outcome utilities. In any case, the Tradeoff Model (TM) still received more support than any other model in the contest.

Finally, there is yet another indication that incentivization did not bring decisions closer to the economic model: The choice between a decreasing and a constant sequence was no longer a coin toss, which by itself is nonnormative, but rather strongly disfavored the decreasing sequence in relation to the constant one. This is a gross violation of dominance, meaning that participants indeed moved away from the economic model. The violation of dominance was problematic for all models in the contest, in that they far overpredicted the probability of choosing the dominant option.

## Old Evidence From a New Perspective

Given that the Tradeoff Model (TM), with its cumulative weighing of time, emerges as the winning model overall, the question becomes whether it can also accommodate previously reported evidence on preferences for sequences of unlabeled monetary gains, which, without exception, concerns *aggregate* choice behavior involving *two-outcome* sequences. We saw how it accommodates the asymmetric hidden-zero effect, and how, in disaggregate choice behavior, it is driven by those best described by TM, but what about the remaining evidence? Table 6 compiles that evidence, which, with the exception of the *asymmetric* hidden-zero effect, was addressed by Read and Scholten (2012). In this section, we discuss how TM accommodates the listed anomalies to choice by the Net Present Value model.[Table-anchor tbl6]

### Front-End Amount Effect and Its Reversal

We begin with the single one anomaly that TM, as specified in Equations (3a) and (3b), does *not* explain, the one that Read and Scholten (2012) call the *front-end amount effect*, as first reported by Rao and Li (2011). Consider a choice between “$10 today” (*SS*) and “$30 in two months” (*LL*), and another between “$510 today” (*SS*_*F*_) and “$500 today and $30 in two months” (*LL*_*F*_). The introduction of the common front-end amount of $500 decreases patience, that is, the probability of choosing *LL*_*F*_ is lower than the probability of choosing *LL*. This contradicts TM as specified in Equations (3a) and (3b). By a cumulative weighing of time, the delay of *LL* is two, while the duration of *LL*_*F*_ is shorter than two, which would benefit *LL*_*F*_. Furthermore, by concave utility, the utility difference between the options in period 1 (“today”) is less strongly in favor of *SS*_*F*_ than it is in favor of *SS*, that is, *u*(510) – *u*(500) < *u*(10) – *u*(0), which would benefit *LL*_*F*_ as well. On both counts, the front-end amount should *increase* patience.

The front-end amount effect must be appreciated along with a *reversal* of the effect, as reported by Read and Scholten (2012). Consider a choice between “$510 today” (*SS*_*A*_) and “$530 in two months” (*LL*_*A*_), and another between “$1,510 today” (*SS*_*FA*_) and “$1,000 today and $530 in two months” (*LL*_*FA*_). Now, the introduction of the common front-end amount of $1,000 *increases* patience, that is, the probability of choosing *LL*_*FA*_ is higher than the probability of choosing *LL*_*A*_. This is, of course, compatible with a cumulative weighing of time and concave utility, but, then, *why* the front-end amount effect, and *why* its reversal?

At this point, it must be recognized that the prime motivation for developing the tradeoff model were *similarity effects* (Scholten & Read, 2010), showing that intertemporal choices are influenced the similarity between the options along the time and outcome attributes. Accordingly, we attribute the reversal of the front-end amount effect to the similarity of the options along the outcome attribute upon introduction of the front-end amount.^9^ In the choice between *SS* and *LL*, a large majority preferred *LL*, and, indeed, the interest rate on offer was 73.2% per month (72,800% per year). Therefore, according to TM,

$$u\left(30\right)-u\left(10\right)>\kappa_{T|X}w\left(2\right),$$

where the left-hand side is the outcome advantage of *LL*, and the right-hand side is the time advantage of *SS*, with the former being larger than the latter, so that *LL* is preferred. In the subscript to κ, which measures impatience, *X* is the outcome advantage, and *T* is the time advantage, with *T*|*X* meaning that κ also depends on the similarity of the outcomes, as explained below. Upon introduction of the front-end amount, which leaves the interest rate unchanged, the majority preference for *LL* shrunk toward indifference between *SS*_*F*_ and *LL*_*F*_, which we write as follows:

u(30)−[u(510)−u(500)]=κT|XFw(t^F).

As discussed by Scholten and Read (2010), κ can, in any given local context, acquire two values, depending on “similarity.” The lower value applies when the time advantage of *SS* falls below a threshold, and the higher value applies when the time advantage falls above the threshold. This gives rise to a step function relating the time advantage to the weight assigned to the advantage (see Scholten & Read, 2010, Figure 3). Crucially, the threshold depends on the relative similarity of the outcomes and the delays. When the outcomes are treated as “dissimilar,” the threshold for the time advantage of *SS* rises, and the time advantage is therefore more likely to be *subthreshold*; this translates into the low value of κ, or less impatience. When the outcomes, on the other hand, are seen as “similar,” the threshold for the time advantage of *SS* falls, and the time advantage is thus more likely to be *suprathreshold*; this translates into the high value of κ, or more impatience. We argue that introduction of the front-end amount increases the similarity between the options along the outcome attribute, and, because the outcomes are more likely to be treated as “similar,” the time advantage of *SS* is more likely to be suprathreshold, that is, κ_*T*|*X*_*F*__ > κ_*T*|*X*_. If this similarity effect outweighs duration, by which *t̂*_*F*_ < 2, and concave utility, by which *u*(510) – *u*(500) < *u*(10), it drives the preference relation between *SS*_*F*_ and *LL*_*F*_ toward indifference.

In the choice between *SS*_*A*_ and *LL*_*A*_, however, a large majority preferred *SS*_*A*_, and, indeed, the interest rate on offer was now “only” 1.9% per month (still 26.0% per year).^10^ According to TM,

$$u\left(530\right)-u\left(510\right)<\kappa_{T|X_{A}}w\left(2\right).$$

Upon introduction of the front-end amount, the majority preference for *SS*_*F*_ shrunk toward indifference between *SS*_*FA*_ and *LL*_*FA*_, which we write as follows:

u(530)−[u(1,510)−u(1,000)]=κT|XFAw(t^FA),

where the similarity effect, that is, κ_*T*|*X*_*FA*__ > κ_*T*|*X*_*A*__, countervails, *but does not outweigh*, duration, by which *t̂*_*FA*_ < 2, and concave utility, by which *u*(1,510) – *u*(1,000) < *u*(510). One reason why similarity no longer prevails may be that $1,510 and $1,000 (in the reverse front-end amount effect) are less likely to be judged as “similar” than $510 and $500 (in the front-end amount effect). The other reason is that, by concave utility, the difference between *u*(510) and *u*(1,510) – *u*(1,000) is larger than the difference between *u*(10) and *u*(510) – *u*(500), meaning that concave utility becomes more prevalent. Thus, TM can accommodate the front-end amount effect and its reversal by combining duration and concave utility with similarity effects.

### Relocation Effect

Read and Scholten (2012) also reported what we call a *relocation effect*. Consider a choice between “$510 today” (*SS*_*F*_) and “$500 today and $30 in two months” (*LL*_*F*_), and another between “$10 today and $500 in two months” (*SS*_*B*_) and “$530 in two months” (*LL*_*B*_). Thus, the common amount of $500 is relocated from the front end in the first choice to the back end in the second choice. Relocation decreases patience, that is, the probability of choosing *LL*_*B*_ is lower than the probability of choosing *LL*_*F*_, although it leaves the interest rate unchanged. Formally, we might have:

u(30)−[u(510)−u(500)]=κT|XFw(t^F),

and

[u(530)−u(500)]−u(10)<κT|XB[w(2)−w(t^B)].

Assuming that $530 and $500 are not less likely to be treated as “similar” than $510 and $500, the relocation effect arises when concave utility outweighs duration. The time disadvantage of *LL*_*B*_ is smaller than that of *LL*_*F*_, that is, 2 – *t̂*_*B*_ < 1 while *t̂*_*F*_ > 1. Countervailing duration is concave utility, by which the outcome advantage of *LL*_*B*_ is smaller than that of *LL*_*F*_, that is, *u*(530) < *u*(30) + *u*(500) and −*u*(500) – *u*(10) < −*u*(510). The effect of concave utility can be expected to be substantial, because the utility decrements from *u*(30) to *u*(530) −*u*(500), and from −[*u*(510) − *u*(500)] to –*u*(10), are substantial (see also Read & Scholten, 2012, Figure 2). Thus, TM can accommodate the relocation effect through concave utility, and the desire for spreading it generates.

### Violations of Independence

Three additional anomalies to the Net Present Value model are *violations of independence* (Koopmans, 1960), a condition that is invoked in the axiomatic derivation of the discounted utility model. Independence states that time periods yielding identically valued outcomes regardless of one’s choice (a *common consequence*) should cancel out, and therefore not affect preference. The evidence shows that adding a common consequence *does* affect preference. Loewenstein (1987) provided a first demonstration in the domain of consumption, which, as shown by Loewenstein and Prelec (1993), can be accommodated by the sequences model. We continue to concentrate on unlabeled monetary gains, and discuss violations of independence in that restricted domain.

Read and Scholten (2012) reported a violation that we call the *common-consequence effect*. Consider a choice between “$300 today” (*SS*) and “$400 in 50 weeks” (*LL*), and another between “$300 today and $350 in 4 weeks” (*SS*_*C*_) and “$350 in 4 weeks and $400 in 50 weeks” (*LL*_*C*_). In the choice between *SS* and *LL*, few participants preferred *LL*, but, in the choice between *SS*_*C*_ and *LL*_*C*_, more participants preferred *LL*_*C*_, a small but reliable effect. Therefore, the common consequence increases patience, that is, the probability of choosing *LL*_*C*_ is higher than the probability of choosing *LL*. Formally, we might have:

$$u\left(400\right)-u\left(300\right)<\kappa_{T|X}w\left(50\right),$$

and

[u(400)−u(300)]+[u(350)−u(350)]≤κT|XC[w(t^LLC)−w(t^SSC)].

Obviously, the common consequence does not affect the utility difference between the options, but the common consequence is written down so as to imply that it increases, by the similarity effect, the marginal impact of time, that is, κ_*T*|*X*_*C*__ > κ_*T*|*X*_. Outweighing the similarity effect, however, is the effect of duration: As a result of the common consequence, the duration of *SS*_*C*_ is longer than the delay of *SS*, that is, *t̂*_*SS*_*C*__ > 0, and the duration of *LL*_*C*_ is shorter than the delay of *LL*, that is, *t̂*_*LL*_*C*__ < 50, thus increasing patience. As mentioned earlier, the effect was small, and this analysis indeed suggests a delicate balance between similarity and duration.

The remaining violations of independence in the literature involve common consequences located *before*, rather than *between*, the smaller-sooner and the larger-later outcome. Urminsky and Kivetz (2011) reported what they call a *mere-token effect*, by which the common consequence increases patience, but does not increase patience much more as the token becomes larger. However, Read and Scholten (2012) showed, drawing on their formulation of the tradeoff model, that this depends on how much larger the token becomes. Consider a choice between “$200 in one week” (*SS*) and “$400 in one year” (*LL*), another between “$50 tomorrow and $200 in one week” (*SS*_*M*_) and “$50 tomorrow and $400 in one year” (*LL*_*M*_), and yet another between “$5,000 tomorrow and $200 in one week” (*SS*_*AM*_) and “$5,000 tomorrow and $400 in one year” (*LL*_*AM*_). A minority preferred *LL* over *SS*, participants were equally divided *SS*_*M*_ and *LL*_*M*_, and a majority preferred *LL*_*AM*_ over *SS*_*AM*_. Therefore, the probability of choosing *LL*_*M*_ was higher (by a narrow but reliable margin) than the probability of choosing *LL*, and the probability of choosing *LL*_*AM*_ was higher (by a wider margin) than the probability of choosing *LL*_*M*_. This result, which we call the *amplification effect*, can be written as follows:

$$u\left(400\right)-u\left(200\right)<\kappa_{T|X_{C}}\left[w\left(365\right)-w\left(7\right)\right],$$

[u(400)−u(200)]+[u(50)−u(50)]=κT|XM[w(t^LLM)−w(t^SSM)],

and

[u(400)−u(200)]+[u(5,000)−u(5,000)]>κT|XAM[w(t^LLAM)−w(t^SSAM)].

**Figure 7 fig7:**
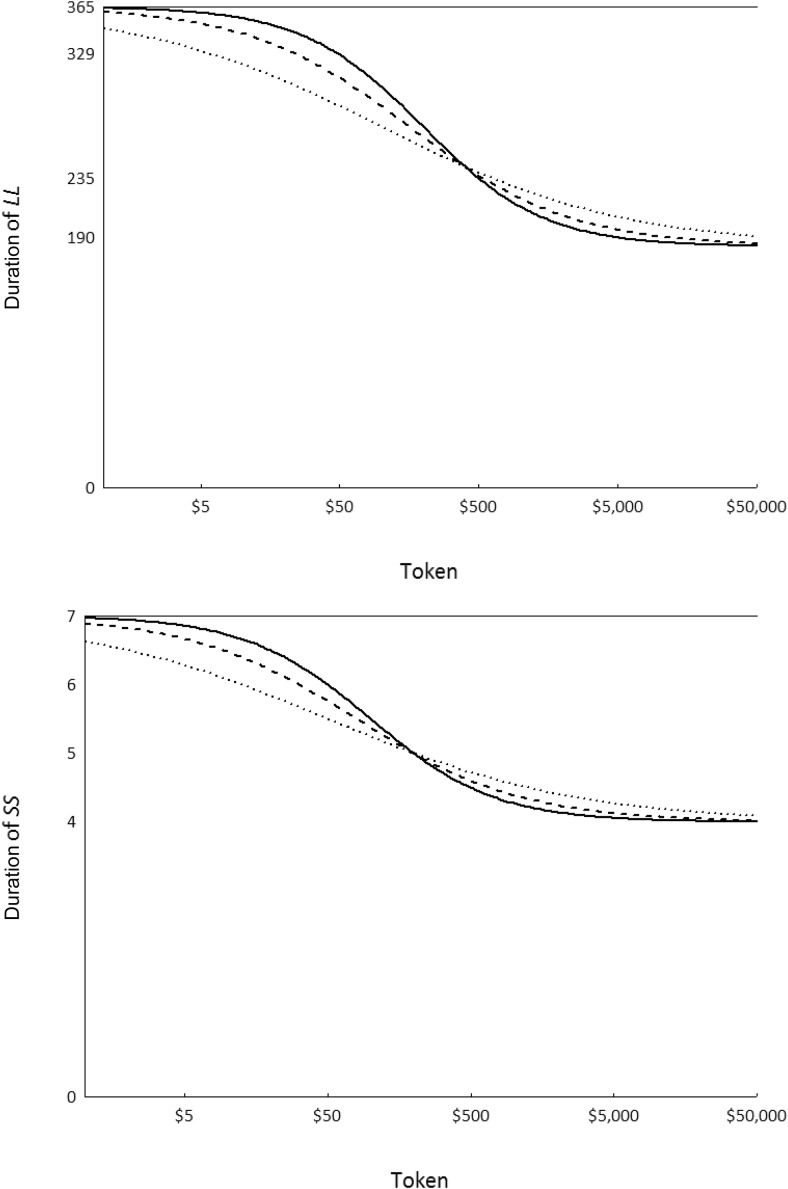
Duration of *SS* and *LL* as a function of token magnitude. Duration is given in days, and computed with *u*(*x*) = *x*^γ^, where γ = 1 (solid line), .75 (dashed line), and .50 (dotted line). Numbers along the ordinate are the duration under linear utility for the $50, $500, and $5,000 tokens. The tokens, along the abscissa, are logarithmically spaced.

The observed effects are, according to TM, carried entirely by duration. Figure 7 shows how duration, under a cumulative weighing of time, decreases as the token increases, separately for *SS* (which the tokens transform into *SS*_*M*_ and *SS*_*AM*_) and *LL* (which the tokens transform into *LL*_*M*_ and *LL*_*AM*_). With the $50 token being introduced, the duration of *SS* decreases, under linear utility, from seven to six days, whereas the duration of *LL* decreases more, from 365 to 329 days. If duration outweighs the similarity effect, by which κ_*T*|*X*_*M*__ > κ_*T*|*X*_, and diminishing sensitivity, by which the marginal impact of delay or duration decreases with their length, that is, *w*′(365) < *w*′(7), we obtain the mere-token effect. With the $50 token being replaced by a $5,000 token, the duration of *SS* decreases, under linear utility, from six to four days, while the duration of *LL* decreases much more, from 329 to 190 days. If, as is likely, duration outweighs diminishing sensitivity, we obtain the amplification effect. Therefore, the violations of independence seen in the literature can be comprehensively dealt with by a cumulative weighing of time, which yields the duration of utility accumulation.[Fig-anchor fig7]

### Violations of Dominance

A final piece of evidence concerns *violations of dominance*, as reported by Scholten and Read (2014). One violation involved monetary gains. Consider a choice between “$75 today” (*SS*) and “$100 in one year” (*LL*), and another between “$75 today *and* $5 in one year” (*SS*_*D*_), which dominates *SS*, and “$100 in one year” (*LL*_*D*_), which is the same as *LL*. A large majority preferred *SS*, but a *minority* preferred *SS*_*D*_. Another violation of dominance involved monetary losses. Consider a choice between “paying $75 today” (*SS*) and paying $100 in one year” (*LL*), and another between “paying $75 today *and* paying $5 in one year” (*SS*_*D*_), which is dominated by *SS*, and “paying £100 in one year” (*LL*_*D*_), which is the same as *LL*. A majority preferred *SS*, but a *larger* majority preferred *SS*_*D*_. The explanation offered by TM is that the duration of *SS*_*D*_ is longer than the delay of *SS*; with $75 today accumulating to $80 in one year, the duration will be “a bit more than half a year,” *significantly* longer than “today.” With gains, this drastically decreases the appeal of *SS*_*D*_, and, with losses, it increases the appeal of *SS*_*D*_ (not as drastically, because, as we will point out in the General Discussion, people differ in their time preference for losses). These violations of dominance, involving losses as well as gains, can therefore also be dealt with by a cumulative weighing of time.

## General Discussion

Our theoretical and empirical analysis follows up on rather scarce research that has been done on preferences for sequences of unlabeled monetary gains. We extended the intertemporal tradeoff model (Read & Scholten, 2012) from two-period to multiple-period sequences, and proposed that people choose between sequences by trading off accumulated utilities of gains against the duration of utility accumulation. In Experiment 1, aggregate choice behavior provided qualitative support for the revised tradeoff model over its original specification, and over two other candidate models from the literature: The discounted instantaneous utility model and Loewenstein and Prelec’s (1993) sequences model. In three subsequent experiments, the last one incentivized, disaggregate choice behavior provided quantitative support for the tradeoff model in a Bayesian model contest. In Experiment 3, we showed that the asymmetric hidden-zero effect, which was a prime motivation for revising the tradeoff model, was indeed driven by those best described by the tradeoff model. Finally, we showed how the revised tradeoff model can accommodate remaining evidence from the literature, if it makes room for similarity effects, which was a prime motivation for developing the tradeoff model (Scholten & Read, 2010).

### Paramorphic Representations of Choice

The cumulative weighing of time should be appreciated as a paramorphic feature of the tradeoff model, like other “gestalt” properties in models of decision making. Tversky and Kahneman (1992), in proposing cumulative prospect theory, probably did not contend that people actually go through the mathematically ingenious computation of decision weights; rather, the computations were meant to capture the intuition that people are more sensitive to extreme outcomes in a probability distribution than to intermediate ones (Fennema & Wakker, 1997). Similarly, Loewenstein and Prelec (1993), in proposing the sequences model, probably did not contend that people actually go through laborious comparisons between cumulative utilities afforded by a sequence and those that would be afforded by a uniform sequence; rather, the comparisons were meant to capture the intuition that people are sensitive to improvement and spreading of outcomes in a sequence. On a less speculative note, Loomes (2010), in proposing his *perceived relative argument model* of risky choice, stressed that it is “a model of decisions often made quite quickly and on the basis of perceptions rather than after long deliberation involving complex calculation. The structure of the model is therefore intended to capture tendencies in the ways perceptions are formed and judgments are made: it is not suggested that people actually make calculations strictly according to the formulae but, rather, that the formulae capture key features of the ways in which decision parameters are perceived and processed.” In the same spirit, the cumulative weighing of time is meant to capture the intuition that people are sensitive to how quickly the outcomes in a sequence accumulate: “How much utility do I get until this point in time, how much do I get until that point, and how long does it take?”

### The Tradeoff Model as an Attribute-Based Choice Model

In contrast with choice between single dated outcomes, which, according to Scholten et al. (2014), follows a stochastic *ratio* rule, choice between outcome sequences was assumed to follow a stochastic *difference* rule. If, as in Equation (3b), the difference between weighted durations is traded off against differences in accumulated utilities by a currency parameter κ, rather than a *nonlinear* tradeoff function *Q* (Scholten & Read, 2010; Scholten et al., 2014), the tradeoff model might be rearranged into an (arithmetically) “discounted utility” model, in which choice would be *alternative based* rather than attribute based. However, as mentioned in the introduction, this interpretation of the tradeoff model has the undesirable characteristic that a sequence of *positive* outcomes might receive a *negative* “discounted utility.” Moreover, as discussed in the previous section, even choice between sequences can be susceptible to similarity effects, which expose attribute-based comparisons.

### Features of Sequences

We examined the feasibility of our approach to preferences for sequences in experiments that adopted standard practice in research on this topic, which is that the options afford the same total amount of money, and that the money is distributed over contiguous future time periods. Future model contests should be run under less restrictive conditions. As discussed earlier, the sequences model holds that discounting of individual outcome utilities interacts with the “gestalt” properties of outcomes sequences (improvement and spreading) when a sequence starts in (or close to) the present (e.g., 0, 1, 2) or when time periods are noncontiguous (e.g., 1, 2, 10). The tradeoff model would continue to operate as it does with contiguous future time periods, although it would be necessary to allow the time-weighting function *w* to exhibit diminishing sensitivity to delays, so as to capture the disproportionate impact of shorter durations of outcome sequences. Similarly, the discounted instantaneous utility model would continue to operate as it does with contiguous future time periods, although it would be necessary to generalize the exponential discount function, which exhibits constant sensitivity, into a hyperbolic discount function, which exhibits diminishing sensitivity, and thus captures the disproportionate impact of shorter delays to the outcomes in a sequence.

Future model contests should also consider choices between options that afford *different* total amounts of money. In the introduction, we gave the example of a choice between the rising profile {$100, $150} and the falling profile {$150, $100}, in which the options afford the same total amount of money, and therefore imply a 0% interest rate. One alternative to this would be a choice between {$100, $250} and {$200, $100}, which, as the choice between $100 in one year and $150 in two years, implies a 50% interest rate. We used options with the same total payoffs not only to ensure continuity with past research, but also, and much more importantly, to ensure identification of choice tasks in which the Net Present Value model would dictate a certain choice *regardless of the discount rate*, and the tradeoff model, by the duration of outcome accumulation, would dictate the opposite choice. We thus obtained a particularly strong contrast between the tradeoff model and the model of economic rationality, but future research might be guided by other motives.

### Money and Consumption

Most empirical research on preferences for sequences has used sequences of consumption experiences or labeled monetary money starting either in the present or in the future (Chapman, 1996; Guyse et al., 2002; Loewenstein & Prelec, 1993; Loewenstein & Sicherman, 1991; Matsumoto et al., 2000; Read & Powell, 2002). We tried to tap into “raw” preferences for sequences, or preferences purely driven by the magnitudes of, and delays to, the outcomes in the sequence, by using sequences of *unlabeled future money*, thus reducing the role of factors like *expectations*, which might exist in preferences for profiles of wage payments or life-time health (Chapman, 1996), the difference in *uncertainty* between present and future money (Epper, Fehr-Duda, & Bruhin, 2011; Halevy, 2008), and *satiation* with consumption experiences that money can buy (Reuben, Sapienza, & Zingales, 2010; Baucells & Sarin, 2007). Loewenstein and Prelec (1993) partly motivated the sequences model with the evidence on preferences for sequences of income from labor or rent (Loewenstein & Sicherman, 1991), but applied the model (equivalent the Zero Sequences Model) to preferences for sequences of consumption experiences, and found that it outperformed a consumption analogue of the discounted instantaneous utility model. In our application of the models to preferences for sequences of unlabeled future money, it was the other way around. More must be done to better understand the variability of relative model performance across settings, and thus come to further insight into the psychology of preferences for sequences.

It must be recognized, however, that consumption decisions, although a legitimate focus of inquiry, do complicate a *controlled* analysis of intertemporal preferences. Reuben et al. (2010) identified a number of hurdles for a controlled analysis of intertemporal consumption decisions: Not only do consumption goods easily lead to satiation (e.g., 100 candy bars in one year; see Estle et al., 2007), they also differ from individual to individual in their desirability (some love candy bars, others hate them), they introduce intertemporal uncertainty about tastes (how much will I like a candy bar in one year?), and they are not easily divisible into an arbitrary number of units (trading off two candy bars in one year against one candy bar today already entails a 100% yearly interest rate). Money removes all hurdles in one sweep. As Abdellaoui et al. (2010) conclude: “It is hard to see how one could construct sequences of, say, candy bars like we did for money amounts and, to the best of our knowledge, no study has been able to measure the utility of consumption for intertemporal choice. Developing methods to measure the intertemporal utility for consumption is an important topic for future research but as yet this cannot be fulfilled” (p. 861).

The distinction between money and consumption is also important from a theoretical perspective, and Abdellaoui et al. (2010) actually made the above observations in discussing economic theory. Economic analyses of intertemporal choice have been dominated by Samuelson’s (1937) Discounted Utility (DU) model (Loewenstein & Prelec, 1992). Like the Net Present Value (NPV) model, the DU model assumes that the future is discounted exponentially, but unlike the NPV model, which is defined over monetary outcomes, the DU model is defined over consumption. The complete picture of the connection between the NPV model and the DU model is as follows: (a) Given two monetary sequences, choose the one with the highest NPV at the prevailing interest rate, because it places you on the highest attainable intertemporal budget constraint (the income decision), and (b) given the intertemporal budget constraint, choose the consumption stream with the highest DU (the consumption decision). Analyses of choices between monetary sequences place us in stage (a), and psychological models, *including* the Discounted Instantaneous Utility Model, question the NPV model as an accurate description of how people make these choices. Abdellaoui et al. (2010) basically argue that, given the problems in measuring intertemporal utility for consumption, this is the best we can do, which, according to the authors, “seems to lead to the undesirable conclusion that economics is unable to quantify one of its principal models” (p. 861).

### Sequences of Losses

A natural thing to do is to extend the analysis from preferences for sequences of gains to preferences of sequences of *losses* (Guyse & Simon, 2011). As our discussion of Scholten and Read’s (2014) violations of dominance (in the previous section) suggests, the tradeoff model, with its cumulative weighing of time, may apply equally well to preferences for sequences of losses as to preferences for sequences of gains. The aggregate data showed positive time preference, in that a longer duration was better. However, it is likely that these aggregate data conceal disaggregate diversity. Yates and Watts (1975) showed that only half of their participants preferred to pay an amount of money later rather than sooner (positive time preference); the other half preferred to pay it sooner rather than later (*negative* time preference). Hardisty, Appelt, and Weber (2013) showed that negative time preference for monetary losses may transpire *even* in aggregate data. In response to this evidence, Scholten et al. (2016) extended the tradeoff model to accommodate heterogeneity in preferences for single dated losses; when applied to preferences for *sequences* of losses, the tradeoff model must take that heterogeneity into consideration as well, as, indeed, any model should.

### Sequences of Gains and Losses

Further complications arise if we broaden the scope to sequences that *combine* gains and losses. These come even closer to the real-life examples with which we started this paper. The versatile discounted instantaneous utility model, with its atomistic approach to preferences for sequences, can readily be extended to mixed sequences. It is not immediately obvious how the tradeoff model and the sequences model can be extended to mixed sequences; perhaps, drawing on the property of gain-loss separability from prospect theory (Wu & Markle, 2008), it may be that people evaluate the gain and loss parts of the sequences separately; thus, in the tradeoff model, the cumulative weighing of time would occur separately in the two domains, and so would, in the sequences model, the computation of improvement and spreading scores.

The issue of mixed sequences lies wide open as an avenue for future research. There is *some* evidence (Jiang, Hu, & Zhu, 2014; Rao & Li, 2011; Scholten & Read, 2014; Sun & Jiang, 2015), and a common pattern is that the introduction of mixed sequences increases patience. It may, more generally, be that sequences reduce the salience of time: In choice between single dated outcomes, one only needs to attend to two different outcomes occurring at two different delays, but, when choice involves sequences, the *evolution* of outcomes must be tracked over time, which may draw attention away from delays, and toward the outcomes (see also Jiang et al., 2014; Sun & Jiang, 2015). However, our analysis shows that there is much more to preferences for sequences than just an overall rise in patience, and past evidence on preference for unmixed sequences already attests to this, in the form of the *asymmetric* hidden-zero effect, the front-end amount effect, the relocation effect, and the amplification effect (see Table 6). The evidence presented in this paper further exposes the rich psychology behind preferences for sequences.

## Supplementary Material

10.1037/xge0000198.supp

## Figures and Tables

**Table 1 tbl1:** Threads of Evidence as Dealt With by the Discounted Instantaneous Utility Model (DIUM), the Sequences Model (SM), and the Tradeoff Model (TM)

Model	Thread of evidence		
	Undecided preference between decreasing and constant sequences	Asymmetric hidden-zero effect	Preference for faster accumulation, i.e., {1,000, 0, 1,000} ≻ {0, 500, 0}
DIUM	Concave utility countervails discounting	Unaccounted for	Convex utility outweighs discounting
SM	(a) Distaste for improvement countervailed by concave utility and a desire for spreading; (b) distaste for improvement and spreading countervailed by concave utility; (c) convex utility and distaste for improvement countervailed by a desire for spreading; (d) a desire for improvement countervailed by convex utility and distaste for spreading; (e) a desire for improvement and spreading countervailed by convex utility; (f) concave utility and a desire for improvement countervailed by distaste for spreading	Concealed zeroes are not inferred; with the later zero (*SS*_0_), a desire for improvement reinforces a desire for spreading; with the sooner zero (*LL*_0_), a desire for improvement countervails a desire for spreading	(a) Concealed zeroes are not inferred; convex utility; (b) concealed zeroes are inferred; convex utility reinforces distaste for spreading or outweighs a desire for spreading; (c) concealed zeroes are inferred; distaste for spreading outweighs concave utility
TM	Cumulative weighing of time countervails concave utility	Cumulative weighing of time	Cumulative weighing of time outweighs concave utility

**Table 2 tbl2:** Sequences, Duration Under a Linear Utility Function, and 95% Confidence Intervals (LOWER, UPPER) for Percentages of Participants (%_H_) Choosing the High NPV Option When Zeroes are not Revealed (N = 356) and When They are Revealed (N = 347), in Experiment 1

#	High NPV				Low NPV				Confidence interval		
	*t*_1_	*t*_2_	*t*_3_	*t̂*	*t*_1_	*t*_2_	*t*_3_	*t̂*	LOWER	*%*_*H*_	UPPER
No-zero condition											
1	300		300	2.33		600		2.00	38.11	43.26	48.40
2	400		200	2.20		600		2.00	47.34	52.53	57.72
3	400		400	2.33		800		2.00	38.11	43.26	48.40
4	600		200	2.14		800		2.00	49.04	54.21	59.39
Zero condition											
1	300	0	300	2.25	0	600	0	2.50	56.26	61.38	66.51
2	400	0	200	2.14	0	600	0	2.50	57.15	62.25	67.35
3	400	0	400	2.25	0	800	0	2.50	55.38	60.52	65.66
4	600	0	200	2.10	0	800	0	2.50	61.31	66.28	71.26
*Note.* NPV = Net Present Value.											

**Table 3 tbl3:** Choice Pairs and Percentages of Participants Choosing the High NPV Option Among Those Supporting the Tradeoff Model (TM, n = 317), the Discounted Instantaneous Utility Model (DIUM, n = 80), the No-Zero Sequences Model (SMNZ, n = 78), and the Zero Sequences Model (SMZ, n = 45), and Among All Those Participating in Experiment 2 (Sample, N = 520)

#	High NPV			Low NPV			Model supported				Sample
	*t*_1_	*t*_2_	*t*_3_	*t*_1_	*t*_2_	*t*_3_	TM	DIUM	SMNZ	SMZ	
1	800	400	200	200	400	800	77.92	81.25	65.38	68.89	75.77
2	600	400	200	200	400	600	77.29	77.50	65.38	68.89	74.81
3	600	400	200	400	400	400	51.42	61.25	65.38	44.44	54.42
4	400	400	400	200	400	600	90.22	85.00	74.36	82.22	86.35
5	800		800	600	200	800	48.90	40.00	65.38	35.56	48.85
6	800		200	200	800		26.50	73.75	66.67	62.22	42.88
7	600		200	200	600		20.82	65.00	58.97	66.67	37.31
8	400		200	200	400		10.41	60.00	47.44	62.22	28.08
9	600		200		800		37.22	91.25	50.00	55.56	49.04
10	400		200		600		31.55	95.00	53.85	46.67	45.96
11	400		400		800		22.71	90.00	44.87	53.33	39.04
12	200		200		400		21.14	85.00	34.62	48.89	35.38
13	200	200	200		600		42.27	90.00	37.18	64.44	50.77
14	200	400	200		800		35.96	83.75	38.46	62.22	45.96
15	400	200	200		800		40.06	92.50	43.59	64.44	50.77
16	400	400	200	200	800		28.08	80.00	53.85	68.89	43.46
17	600	400	200	400	800		27.44	82.50	50.00	64.44	42.50
18	800	400	200	600	800		26.18	73.75	52.56	71.11	41.35
19	800		200	400	600		20.82	53.75	60.26	53.33	34.62
20	800		200	600	400		11.36	47.50	43.59	57.78	25.77
21	600		200	400	400		10.73	43.75	38.46	53.33	23.65
22	600	200	200	400	600		25.87	85.00	50.00	75.56	42.88
23	600	200	200	200	800		39.75	88.75	62.82	66.67	53.08
24	800		600	600	400	400	18.93	38.75	44.87	37.78	27.50
25	600		600	400	400	400	9.78	27.50	29.49	28.89	17.12
26	400		800	200	400	600	17.67	36.25	42.31	24.44	24.81
*Note*. Choice pairs #10, #11, and #9 correspond to choice pairs #2, #3, and #4 in Table 2. NPV = Net Present Value.											

**Table 4 tbl4:** Model Recovery Exercise: Proportions of Occasions, of 1,000, in Which a Generating Model Was Recovered as the Winning Model, of Four

Generating model	Recovered model			
	TM	DIUM	SMNZ	SMZ
TM	.94	.01	.02	.03
DIUM	.12	.80	.06	.02
SMNZ	.01	.08	.89	.03
SMZ	.06	.04	.03	.87
*Note*. TM = Tradeoff Model; DIUM = Discounted Instantaneous Utility Model; SMNZ = No-Zero Sequences Model; SMZ = Zero Sequences Model.											

**Table 5 tbl5:** Medians (Md), Along With the Lower Bounds (L) and Upper Bounds (U), of the 95% Highest Density Intervals of Model Parameters in Experiments 2, 3, and 4 (E2, E3, and E4), Among Those Supporting the Tradeoff Model (TM, n = 312, 498, and 91), the Discounted Instantaneous Utility Model (DIUM, n = 80, 137, and 81), the No-Zero Sequences Model (SMNZ, n = 80, 142, and 23), and the Zero Sequences Model (SMZ, n = 48, 71, and 41)

Experiment	TM				DIUM				SMNZ				SMZ			
		*L*	*Md*	*U*		*L*	*Md*	*U*		*L*	*Md*	*U*		*L*	*Md*	*U*
E2	ε	3.90	4.15	4.43	ε	.12	.29	.52	ε	.00	.80	4.90	ε	.17	.61	1.25
	γ	.95	.97	.99	γ	.46	.55	.64	γ	.00	.13	.45	γ	.00	.15	.28
	ϑ	.11	.16	.22	ϑ	.00	.04	.13	ϑ	.14	.27	.40	ϑ	.06	.19	.31
	κ	18.73	63.66	100.00	δ	.39	.50	.63	β	−99.99	−58.56	−14.45	β	−29.20	−10.02	6.03
									σ	−25.51	67.47	99.99	σ	27.06	72.43	100.00
E3	ε	4.20	4.46	4.83	ε	.32	.58	.92	ε	.00	.10	1.18	ε	.18	.54	1.01
	γ	.97	.99	1.00	γ	.48	.54	.60	γ	.18	.45	1.31	γ	.00	.12	.26
	ϑ	.29	.33	.38	ϑ	.04	.17	.30	ϑ	.20	.34	.43	ϑ	.24	.34	.44
	κ	5.58	37.52	84.41	δ	.24	.30	.36	β	−99.15	−78.31	−31.55	β	−29.43	−8.86	7.90
									σ	1.57	24.00	54.74	σ	25.50	72.28	100.00
E4	ε	4.21	5.34	8.29	ε	.71	.84	1.21	ε	.03	3.09	8.66	ε	1.58	2.18	2.83
	γ	.90	.96	.99	γ	.57	.74	.94	γ	.09	.24	.66	γ	.23	.29	.36
	ϑ	.00	.02	.08	ϑ	.00	.05	.13	ϑ	.00	.14	.31	ϑ	.00	.08	.18
	κ	.70	4.10	8.88	δ	.77	.89	.98	β	−19.05	−7.26	3.18	β	−62.00	−38.11	−3.38
									σ	36.63	76.87	100.00	σ	9.71	62.25	99.99

**Table 6 tbl6:** Tradeoff Model With a Cumulative Weighing of Time and Anomalies to the Net Present Value Model Reported in the Literature

Designation	Comparison		Effect	Accommodated by Equations (3a) and (3b)?
	Control	Experimental		
Hidden-zero effect	*SS*: (*x*_*S*_, *t*_*S*_) *LL*: (*x*_*L*_, *t*_*L*_)	*SS*_0_: (*x*_*S*_, *t*_*S*_; 0, *t*_*L*_) *LL*_0_: (0, *t*_*S*_; *x*_*L*_, *t*_*L*_)	More patience	Yes
		Sooner *SS*: (*x*_*S*_, *t*_*S*_)	No	
Asymmetric hidden-zero effect	*SS*: (*x*_*S*_, *t*_*S*_) *LL*: (*x*_*L*_, *t*_*L*_)	zero *LL*_0_: (0, *t*_*S*_; *x*_*L*_, *t*_*L*_)	effect	Yes
		Later *SS*_0_: (*x*_*S*_, *t*_*S*_; 0, *t*_*L*_) zero *LL*: (*x*_*L*_, *t*_*L*_)	More patience	
Front-end amount effect	*SS*: (*x*_*S*_, *t*_*S*_) *LL*: (*x*_*L*_, *t*_*L*_)	*SS*_*F*_: (*x*_*F*_ + *x*_*S*_, *t*_*S*_) *LL*_*F*_: (*x*_*F*_, *t*_*S*_; *x*_*L*_, *t*_*L*_)	Less patience	No
Reverse front-end amount effect	*SS*_*A*_: (*x*_*A*_ + *x*_*S*_, *t*_*S*_) *LL*_*A*_: (*x*_*A*_ + *x*_*L*_, *t*_*L*_)	*SS*_*FA*_: (*x*_*F*_ + *x*_*A*_ + *x*_*S*_, *t*_*S*_) *LL*_*FA*_: (*x*_*F*_, *t*_*S*_; *x*_*A*_ + *x*_*L*_, *t*_*L*_)	More patience	Yes
Relocation effect	*SS*_*F*_: (*x*_*R*_ + *x*_*S*_, *t*_*S*_) *LL*_*F*_: (*x*_*R*_, *t*_*S*_; *x*_*L*_, *t*_*L*_)	*SS*_*B*_: (*x*_*S*_, *t*_*S*_; *x*_*R*_, *t*_*L*_) *LL*_*B*_: (*x*_*R*_ + *x*_*L*_, *t*_*L*_)	Less patience	Yes
Common-consequence effect^a^	*SS*: (*x*_*S*_, *t*_*S*_) *LL*: (*x*_*L*_, *t*_*L*_)	*SS*_*C*_: (*x*_*S*_, *t*_*S*_; *x*_*C*_, *t*_*C*_) *LL*_*C*_: (*x*_*C*_, *t*_*C*_; *x*_*L*_, *t*_*L*_)	More patience	Yes
Mere-token effect^a^	*SS*: (*x*_*S*_, *t*_*S*_) *LL*: (*x*_*L*_, *t*_*L*_)	*SS*_*M*_: (*x*_*M*_, *t*_*M*_; *x*_*S*_, *t*_*S*_) *LL*_*M*_: (*x*_*M*_, *t*_*M*_; *x*_*L*_, *t*_*L*_)	More patience	Yes
Amplification effect^a^	*SS*_*M*_: (*x*_*M*_, *t*_*M*_; *x*_*S*_, *t*_*S*_) *LL*_*M*_: (*x*_*M*_, *t*_*M*_; *x*_*L*_, *t*_*L*_)	*SS*_*AM*_: (*x*_*A*_ + *x*_*M*_, *t*_*M*_; *x*_*S*_, *t*_*S*_) *LL*_*AM*_: (*x*_*A*_ + *x*_*M*_, *t*_*M*_; *x*_*L*_, *t*_*L*_)	More patience	Yes
Violations of dominance	*SS*: (*x*_*S*_, *t*_*S*_) *LL*: (*x*_*L*_, *t*_*L*_)	*SS*_*D*_: (*x*_*S*_, *t*_*S*_; *x*_*D*_, *t*_*D*_) *LL*_*D*_: (*x*_*L*_, *t*_*L*_)	More patience	Yes
^a^ Violations of independence (Koopmans, 1960).				

**Table A1 tbl7:** Distribution (Row-Wise Percentages) of Those Lending Support to the Four Candidate Models in the Main Contest Across the Five Candidate Models in the Parallel Contest, in Experiments 2, 3, and 4

Main contest	Parallel contest																	
	Experiment 2						Experiment 3						Experiment 4					
	TM	DIUM	SMNZ	SMZ	RM	*n*	TM	DIUM	SMNZ	SMZ	RM	*n*	TM	DIUM	SMNZ	SMZ	RM	*n*
TM	212	0	0	0	100	312	334	0	0	1	163	498	64	0	0	0	27	91
	66%	0%	0%	0%	32%		67%	0%	0%	0%	33%		70%	0%	0%	0%	30%	
DIUM	0	62	0	0	18	80	0	102	0	3	32	137	0	61	0	1	19	81
	0%	77.5%	0%	0%	22.5%		0%	74.5%	0%	2%	23.5%		0%	75%	0%	1%	24%	
SMNZ	2	0	22	0	56	80	1	0	40	0	101	142	0	0	13	0	10	23
	2.5%	0%	27.5%	0%	70%		1%	0%	28%	0%	71%		0%	0%	56.5%	0%	43.5%	
SMZ	1	0	0	37	10	48	2	1	0	56	12	71	0	2	2	31	6	41
	2%	0%	0%	77%	21%		3%	1%	0%	79%	17%		0%	5%	5%	75.5%	14.5%	
*Note.* TM = Tradeoff Model; DIUM = Discounted Instantaneous Utility Model; SMNZ = No-Zero Sequences Model; SMZ = Zero Sequences Model; RM = Random-choice Model.																		

**Table A2 tbl8:** Percentages of Bayesian p Values Lower Than .05 Among Those Lending Anecdotal Support to the Respective Models in the Parallel Contest

Experiment	Model									
	Adjustment for dominance detection					No adjustment for dominance detection				
	TM	DIUM	SMNZ	SMZ	RM	TM	DIUM	SMNZ	SMZ	RM
2	0%	16.67%	7.14%	0%	0%	0%	9.09%	14.29%	3.70%	100%
3	0%	15.09%	3.85%	0%	0%	0%	11.76%	7.14%	9.68%	100%
4	0%	0%	0%	0%	0%	0%	0%	9.09%	4.00%	100%
*Note.* TM = Tradeoff Model; DIUM = Discounted Instantaneous Utility Model; SMNZ = No-Zero Sequences Model; SMZ = Zero Sequences Model; RM = Random-choice Model.										

## Footnotes

1 The MTurk survey included several sections on intertemporal choice and risky choice, but the choice between the low and high NPV options was always the first section. The sample was 52% female, with an average age of 34. A majority (58%) were employed (46% full time), and 23% were student. A majority (62%) had an academic degree (47% bachelors, 13% masters, 2% PhD). The second survey included several sections on intertemporal choice, and the choice between the low and high NPV options was always the fourth section. The sample was 57% female, with an average of 52. A majority (54%) were employed (40% full time), and 3% were student. This sample was not as well educated as the first, with 42% having concluded high school, and 37% having concluded university (27% bachelors, 8% masters, 2% PhD).

2 When outcomes are distributed over three contiguous time periods and outcome sequences afford the same total amount of money, as in our experiments, distaste for improvement in the sequences model is analogous to discounting in the discounted instantaneous utility model. To see the analogy, compare the sequences {*x*_1_, *x*_2_, *x*_3_} and {*y*_1_, *y*_2_, *y*_3_}, both summing to *c*. In terms of monetary values, the difference between weighted improvement scores is β(*x*_3_ – *x*_1_) – β(*y*_3_ – *y*_1_). At the same time, the difference between Net Present Values is [δ*x*_1_ + δ^2^(*c* – *x*_1_ – *x*_3_) + δ^3^*x*_3_] – [δ*y*_1_ + δ^2^(*c* – *y*_1_ – *y*_3_) + δ^3^*y*_3_], or [(δ – δ^2^)*x*_1_ – (δ^2^ – δ^3^)*x*_3_] – [(δ – δ^2^)*y*_1_ – (δ^2^ – δ^3^)*y*_3_}, which, given distaste for improvement, that is, β < 0, is analogous to the difference between weighted improvement scores, but with discounting giving a greater weight to period 1 than to period 3.

3 In Loewenstein and Prelec’s (1993) statement of the model, the spreading scores are weighted +σ. We reversed this to −σ, so that the sign of σ indicates whether an individual has a positive (σ > 0) or negative (σ < 0) attitude toward spreading.

4 Duration is also known in the world of finance, where *D* is the duration of a bond (Macaulay, 1938). In the *Macaulay duration*, *a*_*j*_ is given as follows: $a_{j}=\frac{x_{j}/{\left(1+r\right)}^{t_{j}}}{\Sigma_{i=1}^{n}x_{i}/{\left(1+r\right)}^{t_{i}}}.$ In comparison with the preceding calculation of duration, the outcomes are replaced by the Net Present Values of the outcomes. Thus, when discounting the future (*r* > 0), an outcome loses impact with its distance from the present; if it does not, in the absence of discounting (*r* = 0), we obtain the weighted average again.

5 A widely discussed topic in the literature on intertemporal choice is whether discounting is “exponential,” as in the discounted instantaneous utility model, or “hyperbolic.” The discount function of the discounted instantaneous utility model can be given as *d*(*t*) = δ^*t*^ = *e*^−κ*t*^, where κ > 0 is constant discounting. Loewenstein and Prelec (1992) generalized this into *d*(*t*) = (1 + τ*t*)^−κτ^, where κ > 0 is discounting, and τ > 0 is the departure from constant discounting. Rearranging the terms in Equation (3b), and reducing each outcome sequence to a single dated outcome (*x*, *t*), the (arithmetically) “discounted utility” of each outcome would be *u*(*x*) − κ*w*(*t*), where κ > 0 is “discounting.” When the time-weighing function is given as *w*(*t*) = (1/τ)log(1 + τ*t*), where τ > 0 is diminishing sensitivity to delays (Scholten & Read, 2010), Loewenstein and Prelec’s (1992) generalized hyperbolic discount function follows from exponential discounting over weighted delays, that is, *d*(*t*) = *e*^−κ*w*(*t*)^, meaning that hyperbolic discounting is equivalent to diminishing sensitivity to delays (see also Scholten & Read, 2006). In our investigation, sequences span three future and contiguous time periods (i.e., 1, 2, and 3), so that any diminishing sensitivity to delays is difficult to identify. We therefore let *w* be the identity function (i.e., τ → 0), which also ensures formal equivalence to the discounted instantaneous utility model.

6 We used a power value function, because it is widely used in formal modeling, and because it is versatile in allowing for both diminishing sensitivity (γ < 1) and augmenting sensitivity (γ > 1) to outcomes. The tradeoff model, however, was originally proposed to have a logarithmic value function, analogous to the time-weighing function in Footnote 5, that is, *v*(*x*) = (1/γ)log(1 + γ*x*), where γ > 0 is diminishing sensitivity to outcomes (Scholten & Read, 2010). Because our results strongly argue in favor of diminishing sensitivity, the logarithmic value function may be revisited in future modeling.

7 We ran two additional analyses. One used uniform priors from 0 to 1 for γ, δ, and ϑ, the density function 1/(*x* + 1)^2^ for the priors on κ and ε, which were restricted to positive values, and the density function 0.5/(|*x*| + 1)^2^ for the priors on β and σ, which can take on negative as well as positive values. Another analysis used the same priors as in the main contest, except that γ was uniform from 0 to 1 rather than 0 to 5. These analyses yielded the same headline result as the main contest, which is that TM comes out as the winning model.

8 Plots of posteriors of model parameters in the individual-level and group-level analyses are available as online supplemental material.

9 For more discussion of similarity effects in intertemporal choice, see Leland (2002), Rubinstein (2003), Scholten and Read (2010), and Stevens (2016).

10 In a choice-based matching study by Scholten, Read, and Sanborn (2014), participants demanded on average an interest rate of 6.7% per month, so one might expect that the typical participant will regard an interest rate of 1.9% to be low.

## Parallel Contest

In this Appendix, we report the results of a contest, paralleling the main contest, between five candidate models. There are the four substantive psychological models from the main contest. Then there is the Random-choice Model (RM), which is technically a reduced version of any of the four substantive psychological models (when ε is zero). Each of the five candidate models was specified either with or without a parameter for dominance detection (ϑ), making for 10 models in total. Figure A1 shows the posterior probabilities of the five candidate models in the parallel contest, averaged across participants, with the dark and light shades indicating the parts of the support obtained with and without the parameter for dominance detection. Table A1 shows how the supporters of the four candidate models in the main contest distribute across the five candidate models in the parallel contest. Each of the five candidate models takes the posterior probabilities of its variant with adjustment for dominance detection and its variant without adjustment for dominance detection, averaged across the posterior probabilities of all 10 models in the parallel contest. Averaging posteriors is the same as having an average prior for ϑ, that is, an average of the prior with a spike at zero and the prior with a uniform distribution over the interval from 0 to 1. Table A2 shows the proportions of Bayesian *p* values lower than .05, an aggregate measure of badness of fit, among those lending anecdotal support to the respective models in the parallel contest.[Fig-anchor fig8][Table-anchor tbl7][Table-anchor tbl8]
