# Correction to Scholten, Read, and Sanborn (2016)

**DOI:** 10.1037/xge0000313

**Published:** 2017-05

In the article “Cumulative Weighing of Time in Intertemporal Tradeoffs” by Marc Scholten, Daniel Read, and Adam Sanborn (*Journal of Experimental Psychology*: *General*, 2016, Vol. 145, No. 9, pp. 1177–1205. http://dx.doi.org/10.1037/xge0000198), there was an error in Table 1. The preference for faster accumulation read {1,000, 0, 1,000} ≻ {0, 500, 0}. It should read {0, 1,000, 0} ≻ {500, 0, 500}. In addition, in the section **Descriptive Accuracy**, all the equations with the inequality “>” should read “≥” instead. The impact of this change is that, when considering the best model for each participant, as measured by Bayes Factors, the absolute goodness of fit, as measured by Bayesian *p*-values, were better than reported in both Table A2 and the text. All of the corrected cells in Table A2 are 0%, meaning that none of the participants across Experiments 2–4 had a significantly (*p* < .05) poor fit by the model that described them best. None of the conclusions drawn in the text are altered by this change.

