# Supplementary figures and images for: Cumulative Weighing of Time in Intertemporal Tradeoffs (part 2 of 3)
Source: J Exp Psychol Gen. 2016 Sep;145(9):1177–205. doi: 10.1037/xge0000198 (PMC4998108; doi:10.1037/xge0000198)

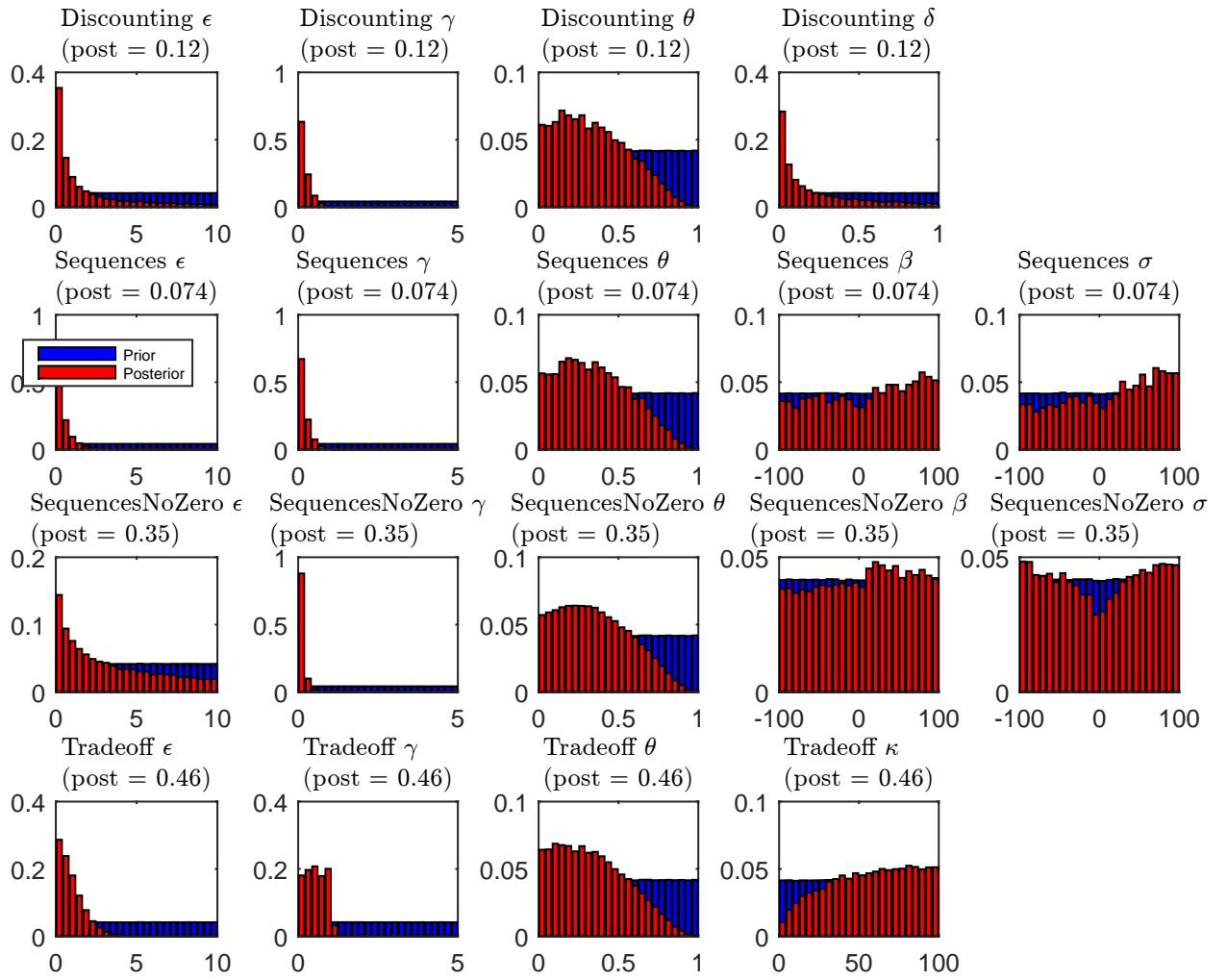

Supplement: Supplementary file 1 [file Scholten_Individuals.zip › plots/e29_p192_eg2_priors_and_posteriors.pdf]

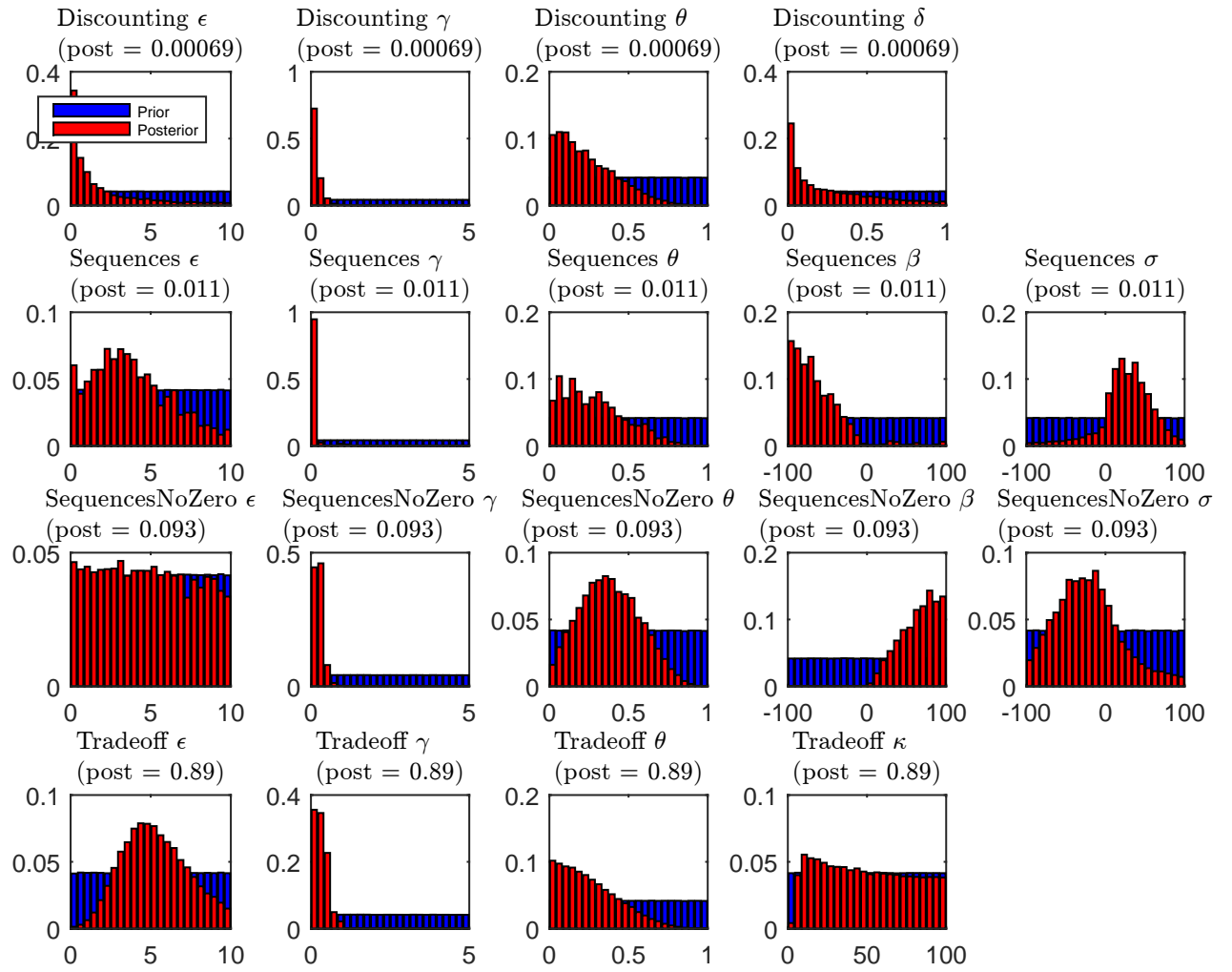

Supplement: Supplementary file 1 [file Scholten_Individuals.zip › plots/e29_p193_eg2_priors_and_posteriors.pdf]

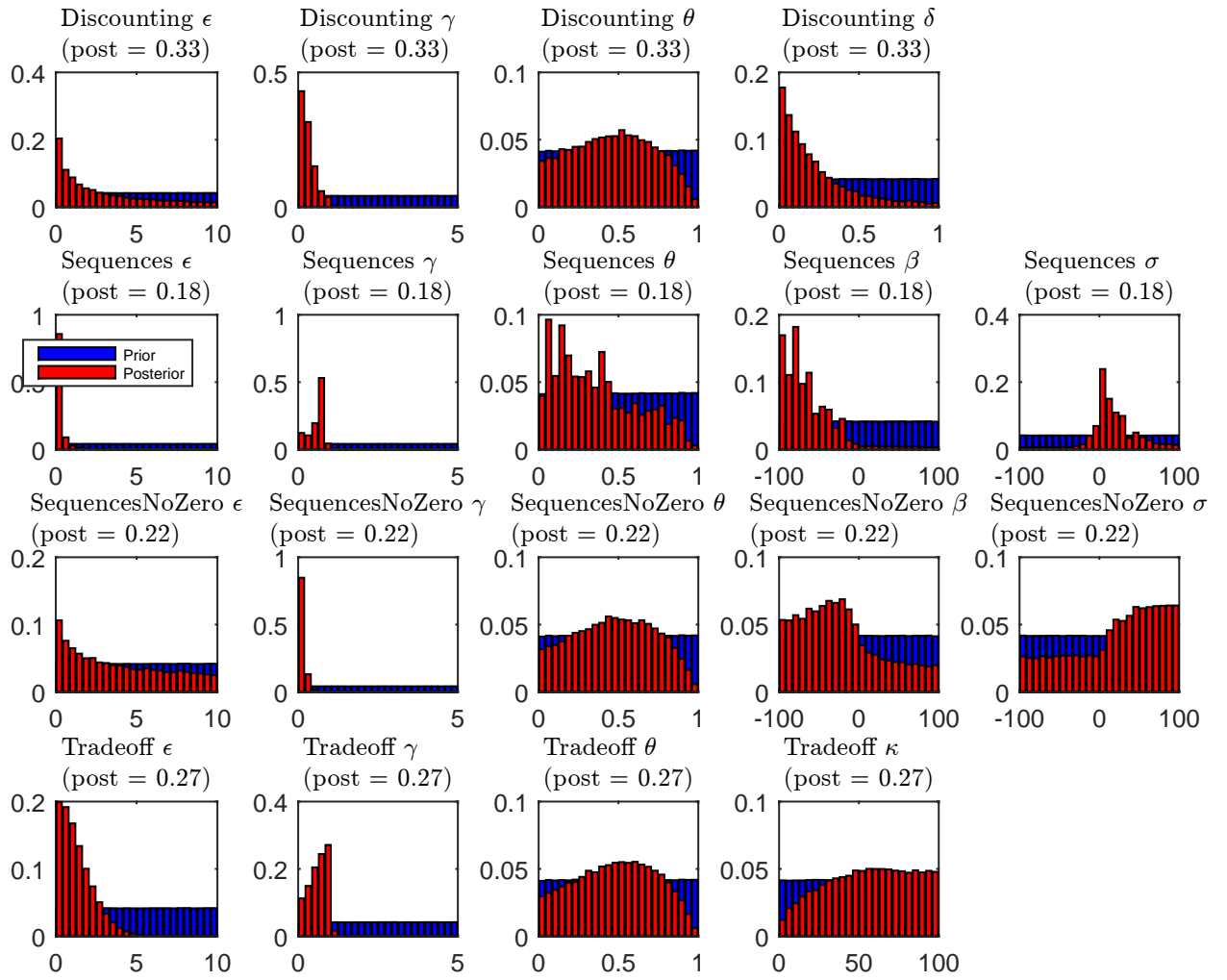

Supplement: Supplementary file 1 [file Scholten_Individuals.zip › plots/e29_p194_eg2_priors_and_posteriors.pdf]

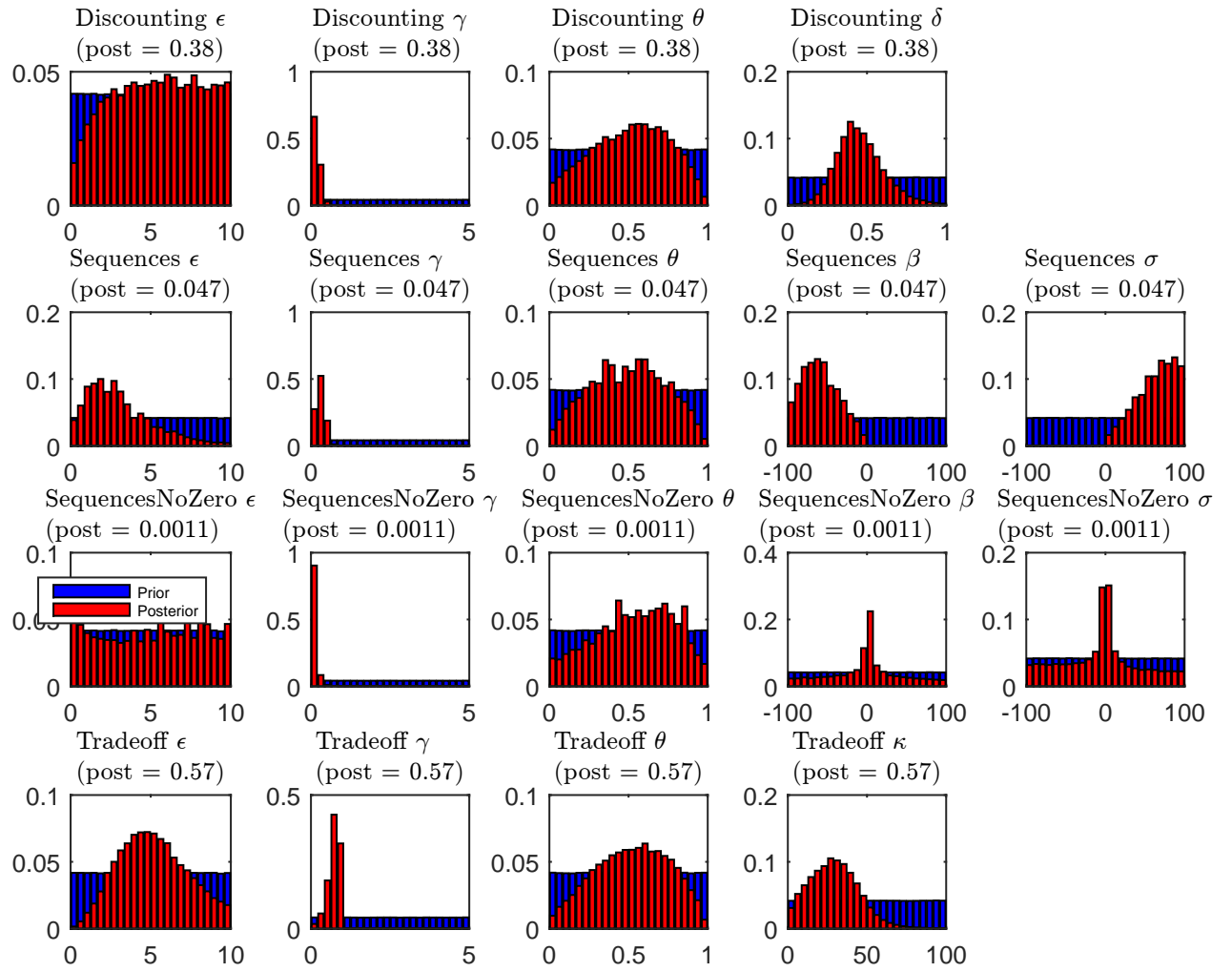

Supplement: Supplementary file 1 [file Scholten_Individuals.zip › plots/e29_p195_eg2_priors_and_posteriors.pdf]

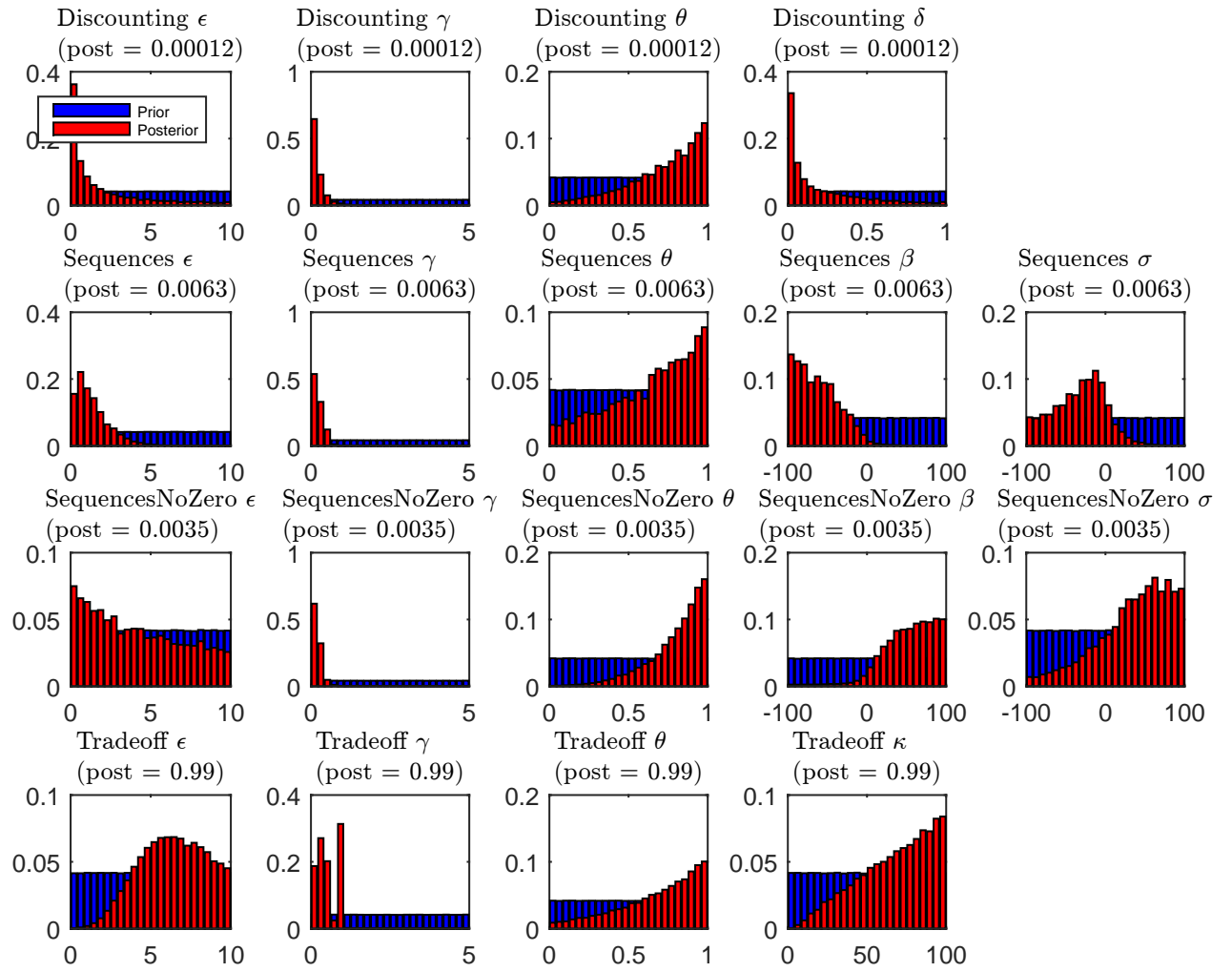

Supplement: Supplementary file 1 [file Scholten_Individuals.zip › plots/e29_p196_eg2_priors_and_posteriors.pdf]

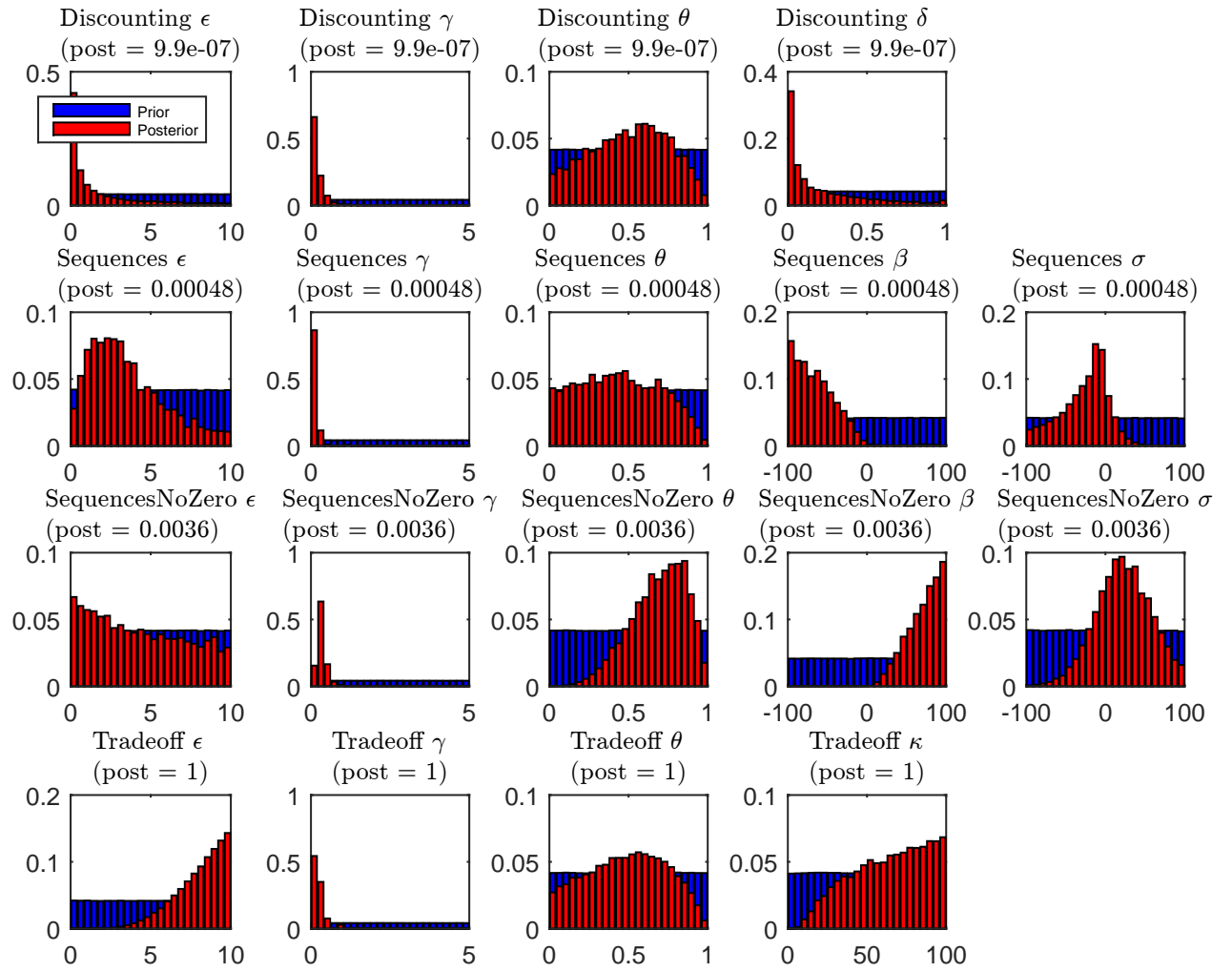

Supplement: Supplementary file 1 [file Scholten_Individuals.zip › plots/e29_p197_eg2_priors_and_posteriors.pdf]

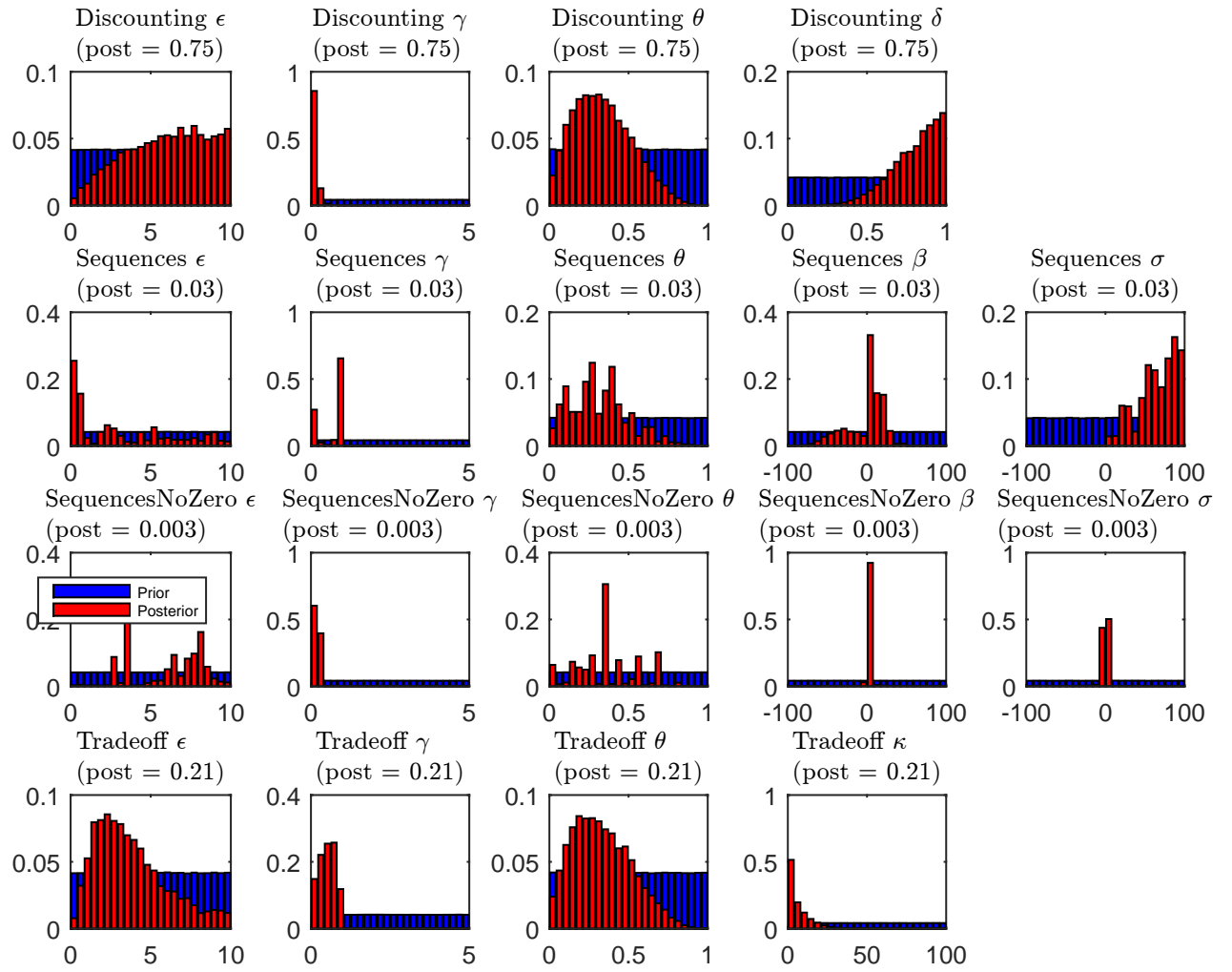

Supplement: Supplementary file 1 [file Scholten_Individuals.zip › plots/e29_p198_eg2_priors_and_posteriors.pdf]

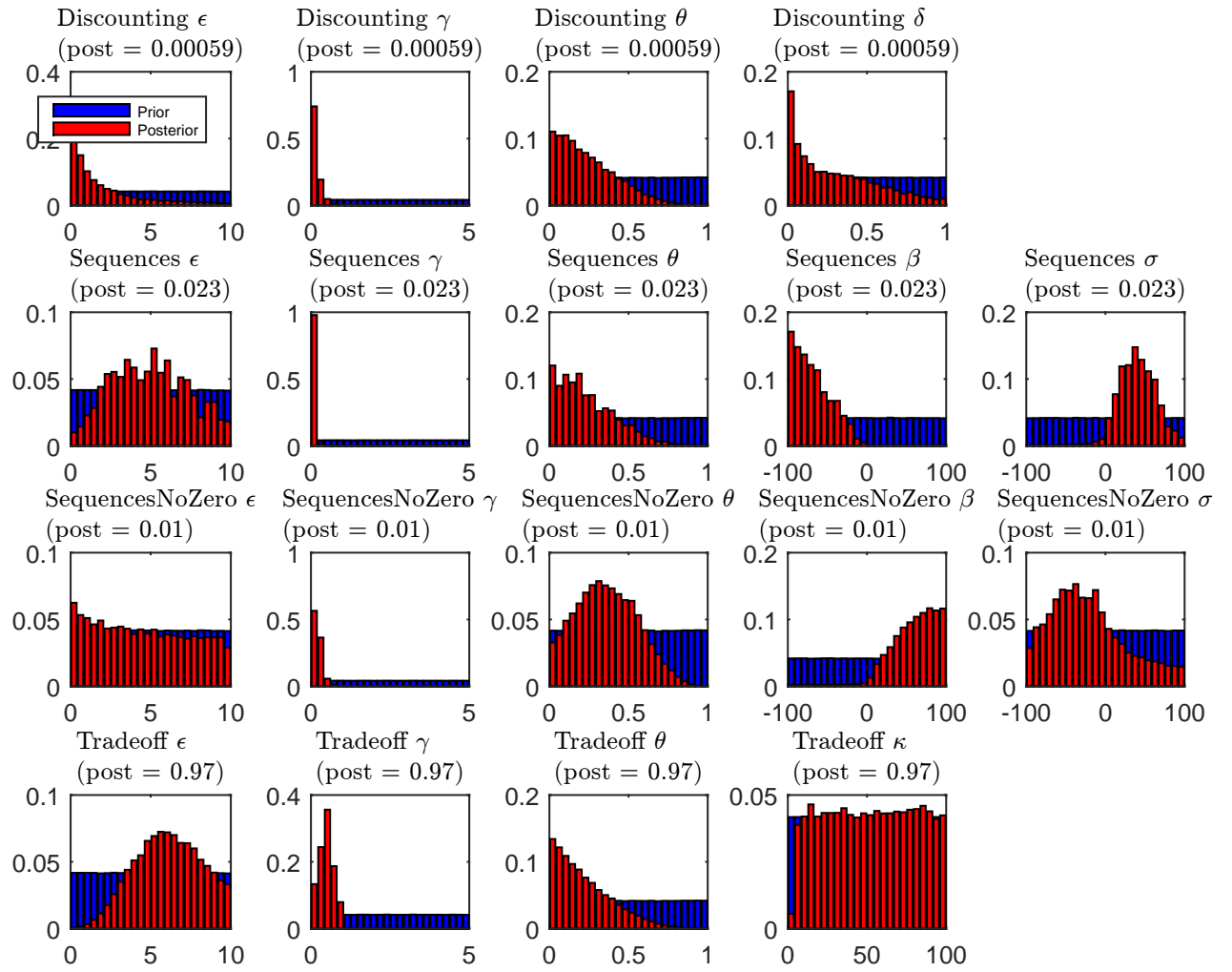

Supplement: Supplementary file 1 [file Scholten_Individuals.zip › plots/e29_p199_eg2_priors_and_posteriors.pdf]

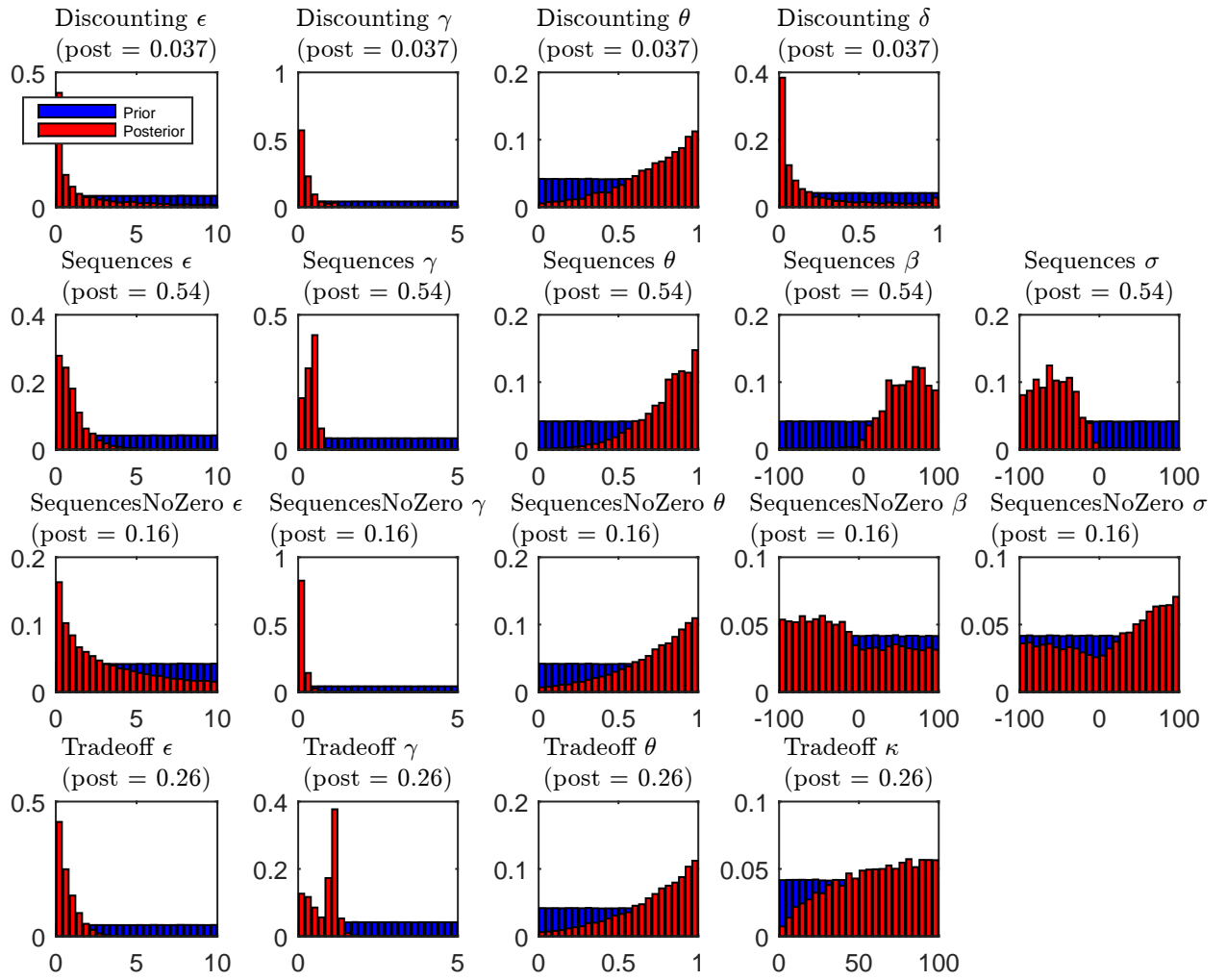

Supplement: Supplementary file 1 [file Scholten_Individuals.zip › plots/e29_p2_eg2_priors_and_posteriors.pdf]

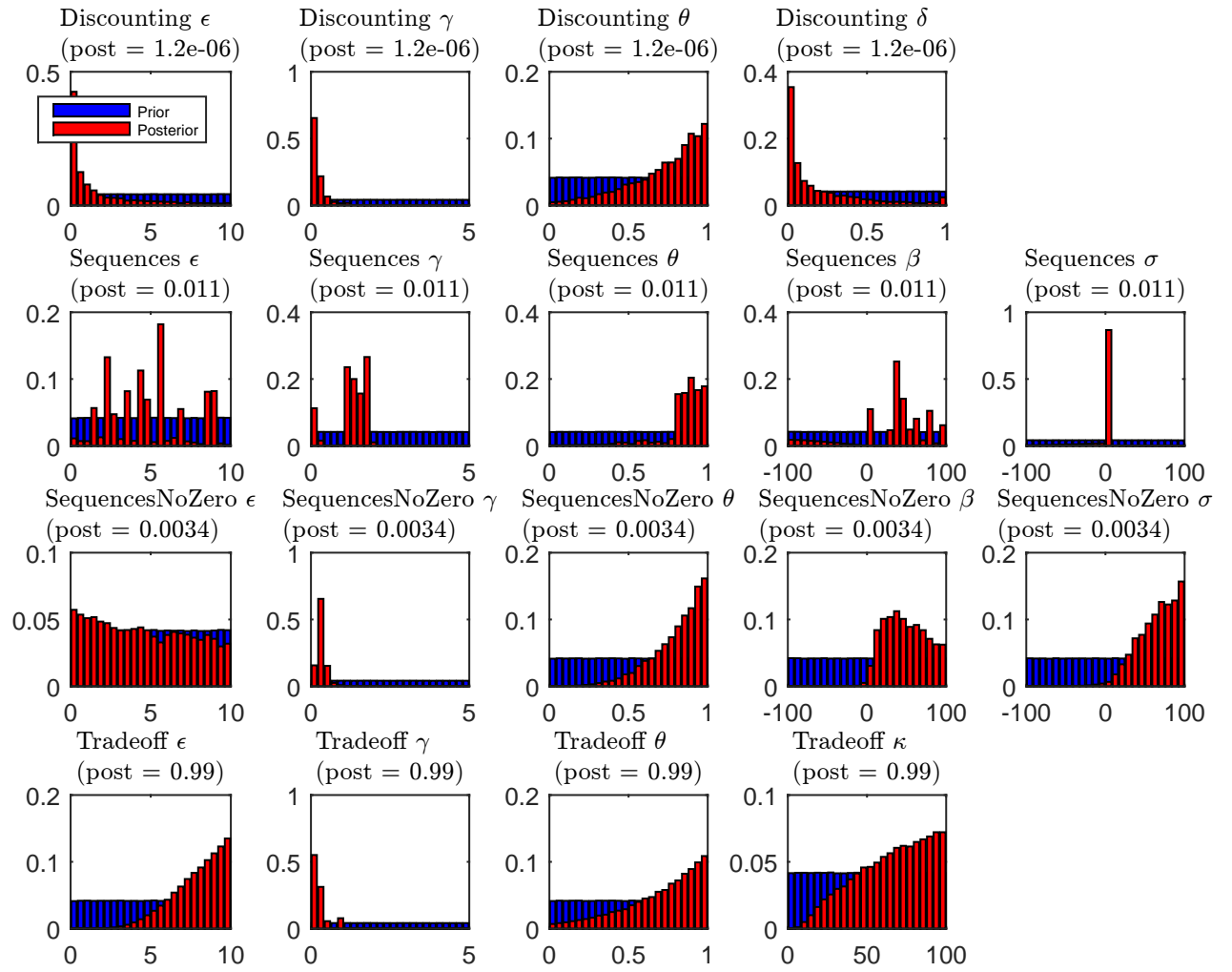

Supplement: Supplementary file 1 [file Scholten_Individuals.zip › plots/e29_p20_eg2_priors_and_posteriors.pdf]

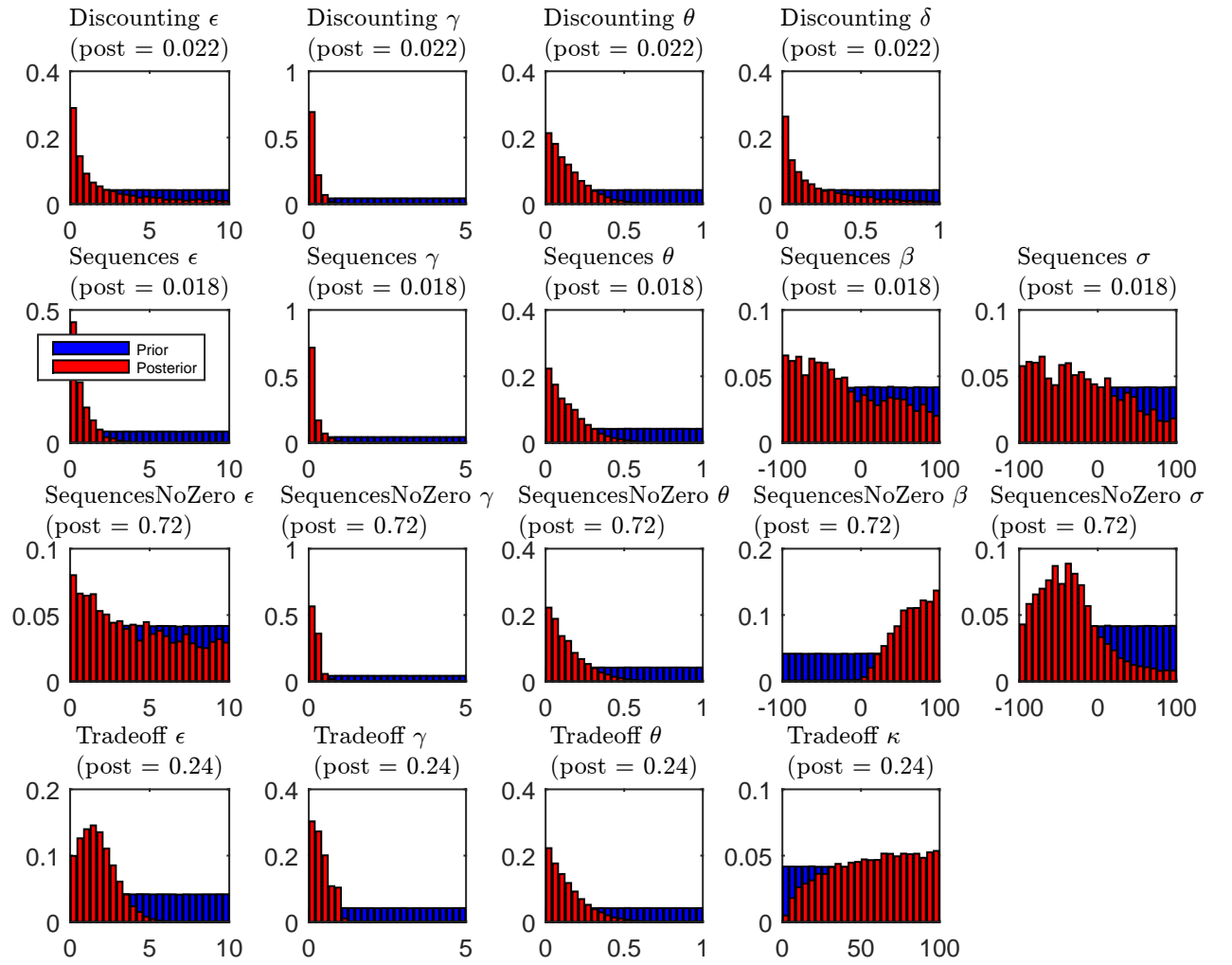

Supplement: Supplementary file 1 [file Scholten_Individuals.zip › plots/e29_p200_eg2_priors_and_posteriors.pdf]

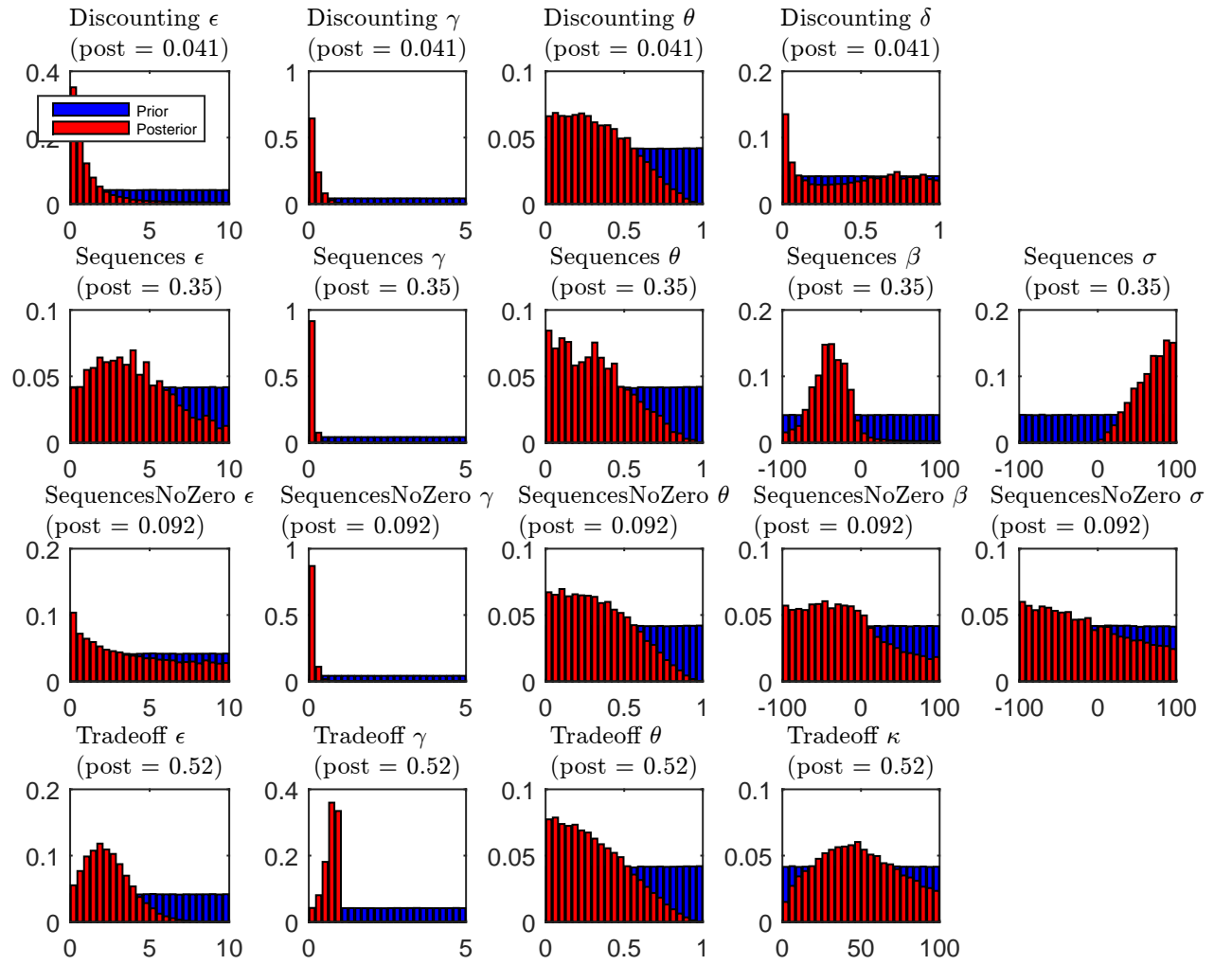

Supplement: Supplementary file 1 [file Scholten_Individuals.zip › plots/e29_p201_eg2_priors_and_posteriors.pdf]

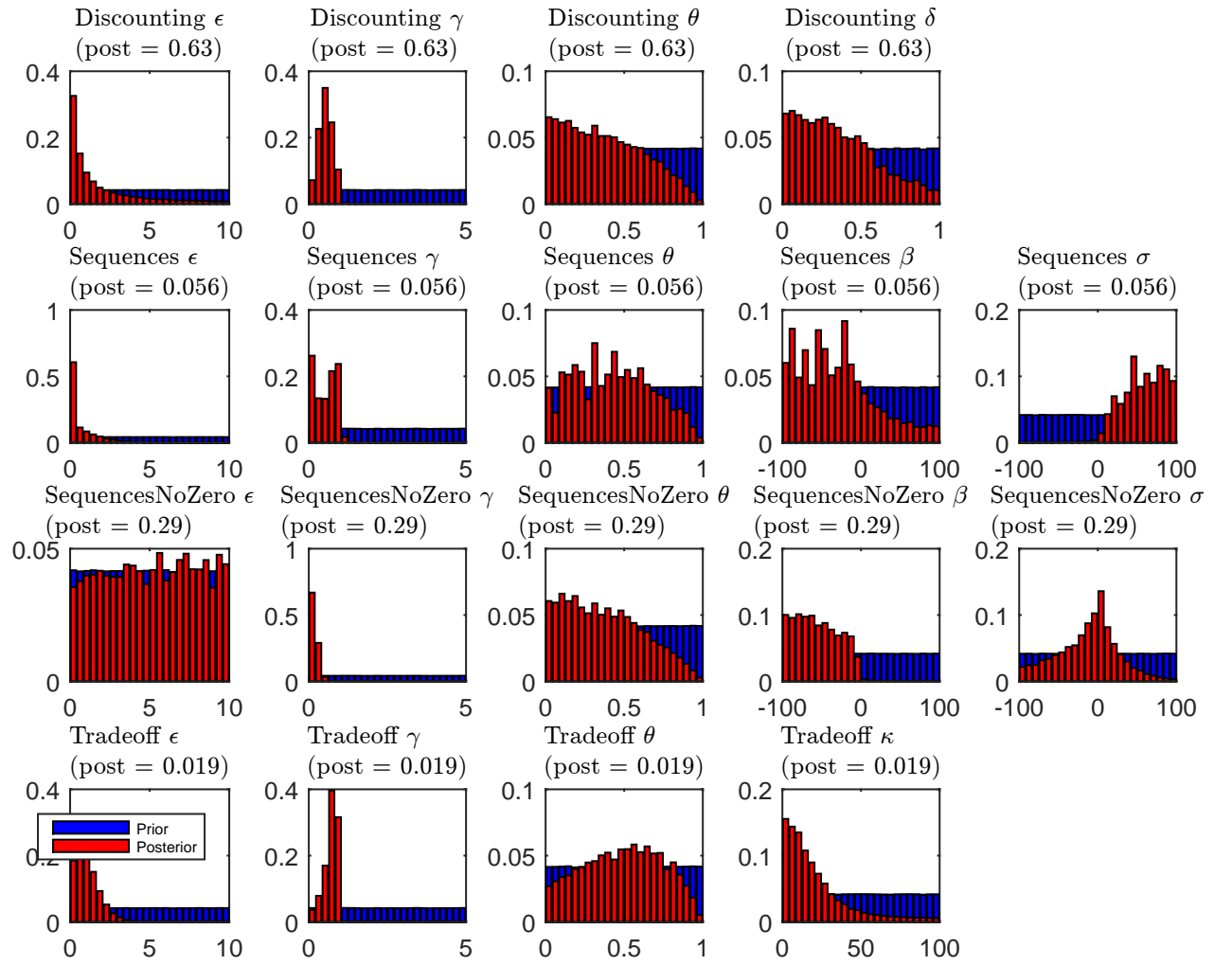

Supplement: Supplementary file 1 [file Scholten_Individuals.zip › plots/e29_p202_eg2_priors_and_posteriors.pdf]

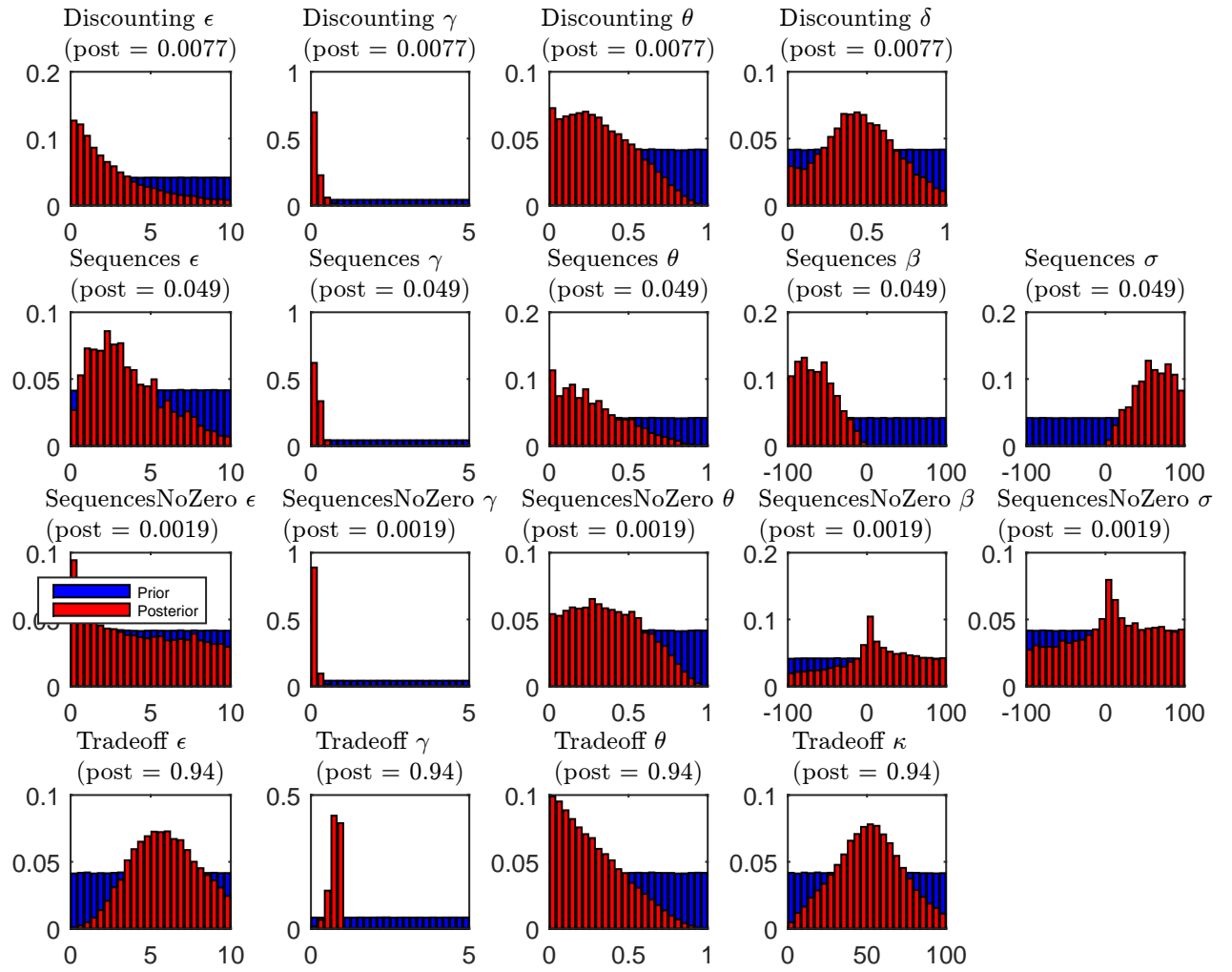

Supplement: Supplementary file 1 [file Scholten_Individuals.zip › plots/e29_p203_eg2_priors_and_posteriors.pdf]

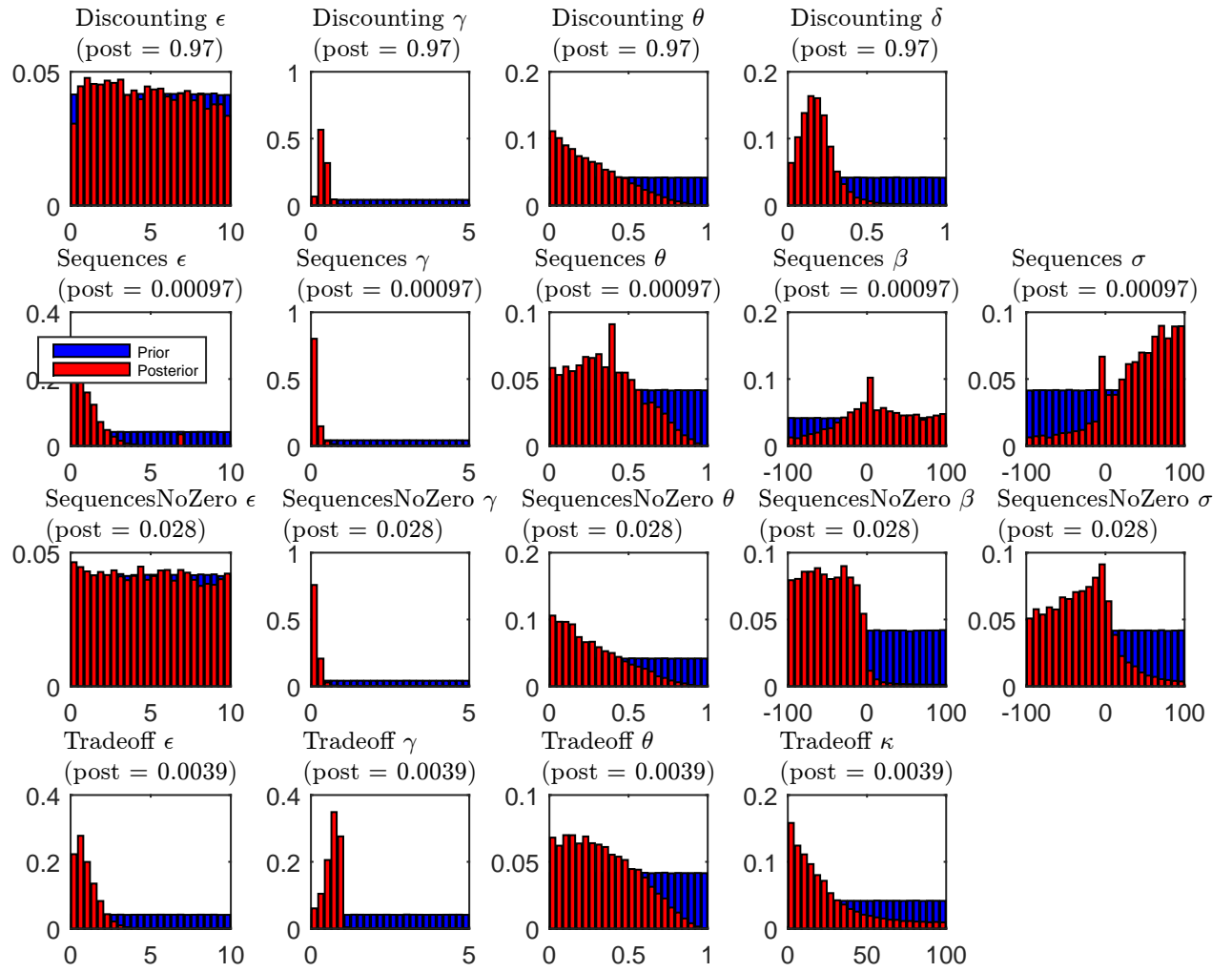

Supplement: Supplementary file 1 [file Scholten_Individuals.zip › plots/e29_p204_eg2_priors_and_posteriors.pdf]

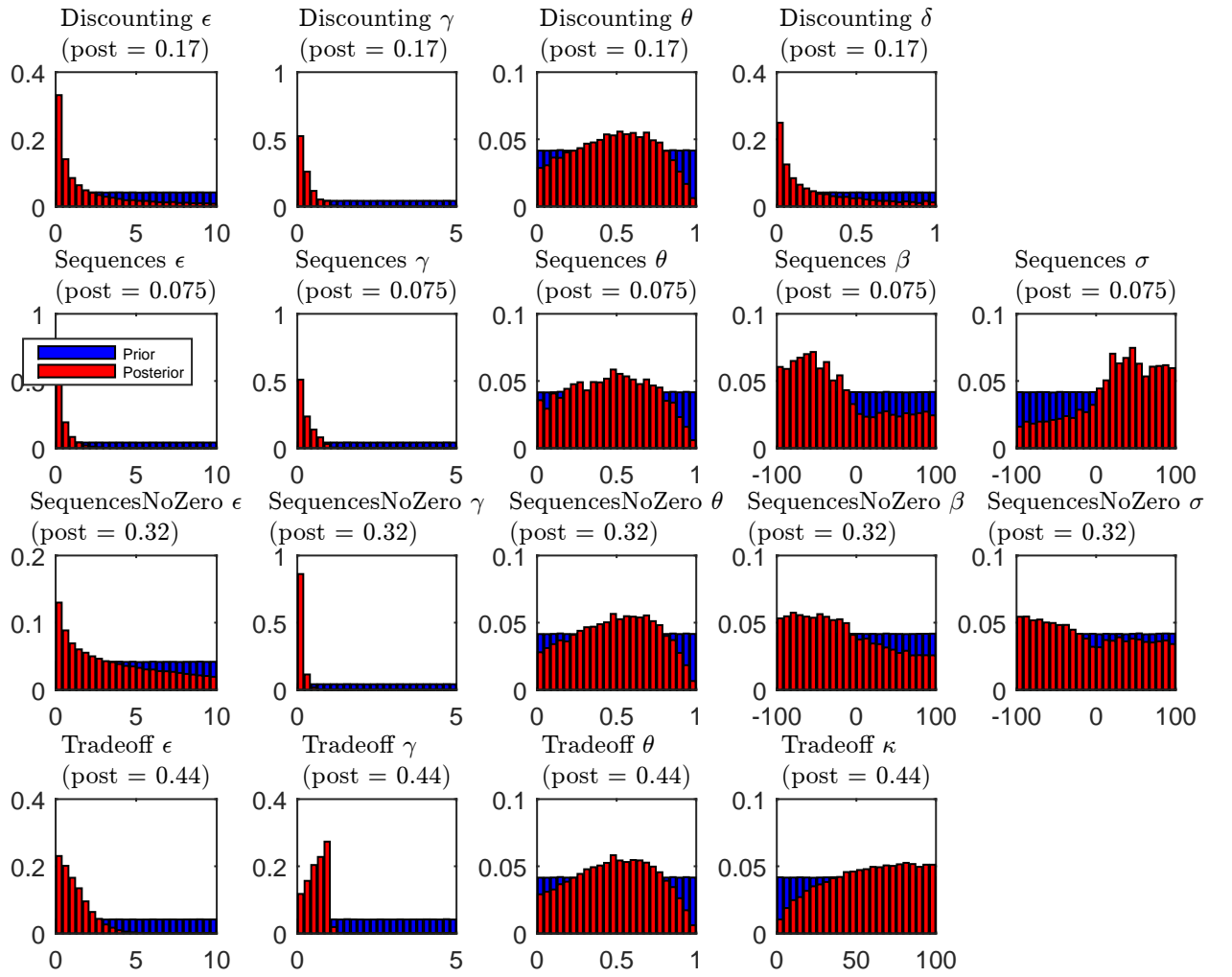

Supplement: Supplementary file 1 [file Scholten_Individuals.zip › plots/e29_p205_eg2_priors_and_posteriors.pdf]

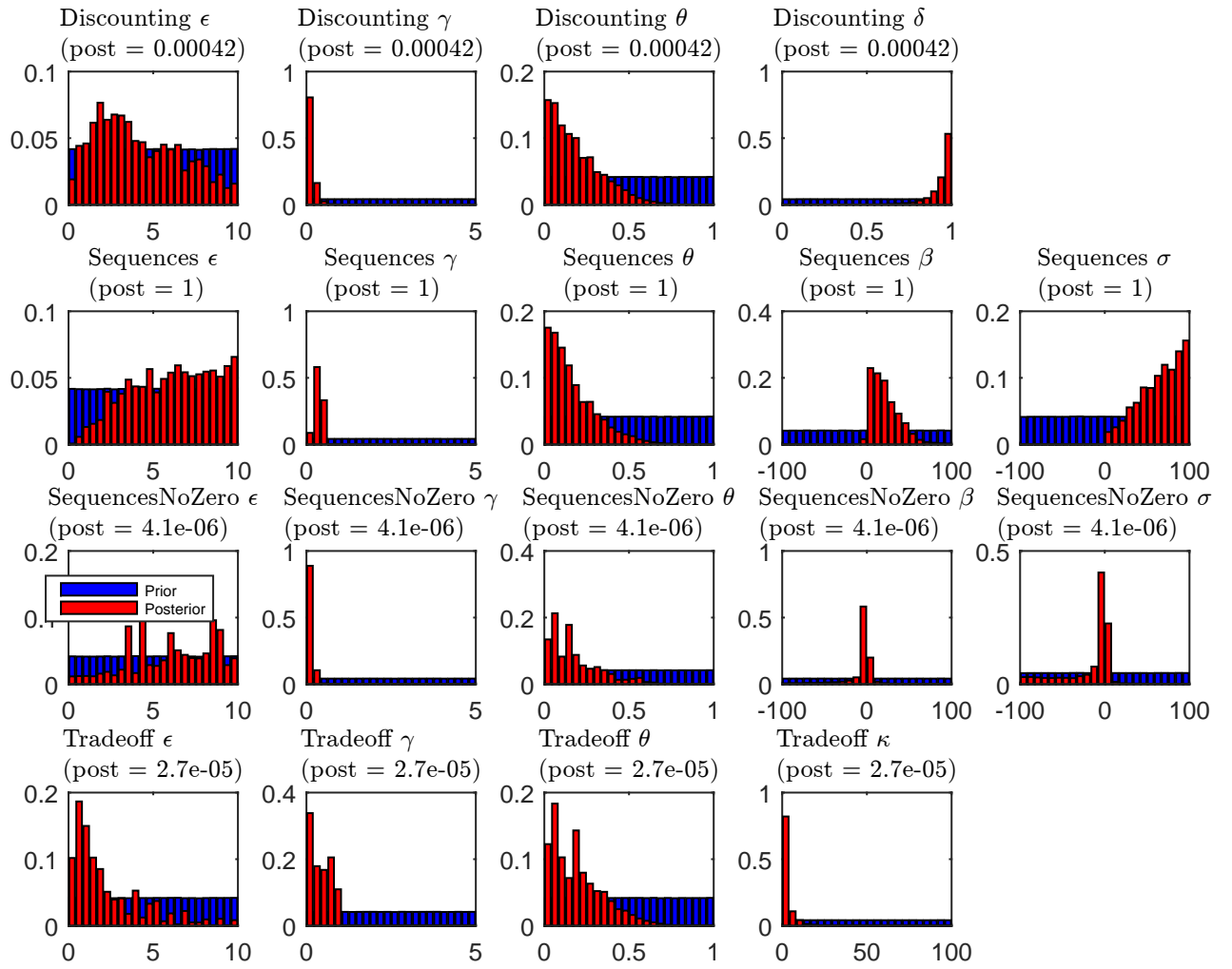

Supplement: Supplementary file 1 [file Scholten_Individuals.zip › plots/e29_p206_eg2_priors_and_posteriors.pdf]

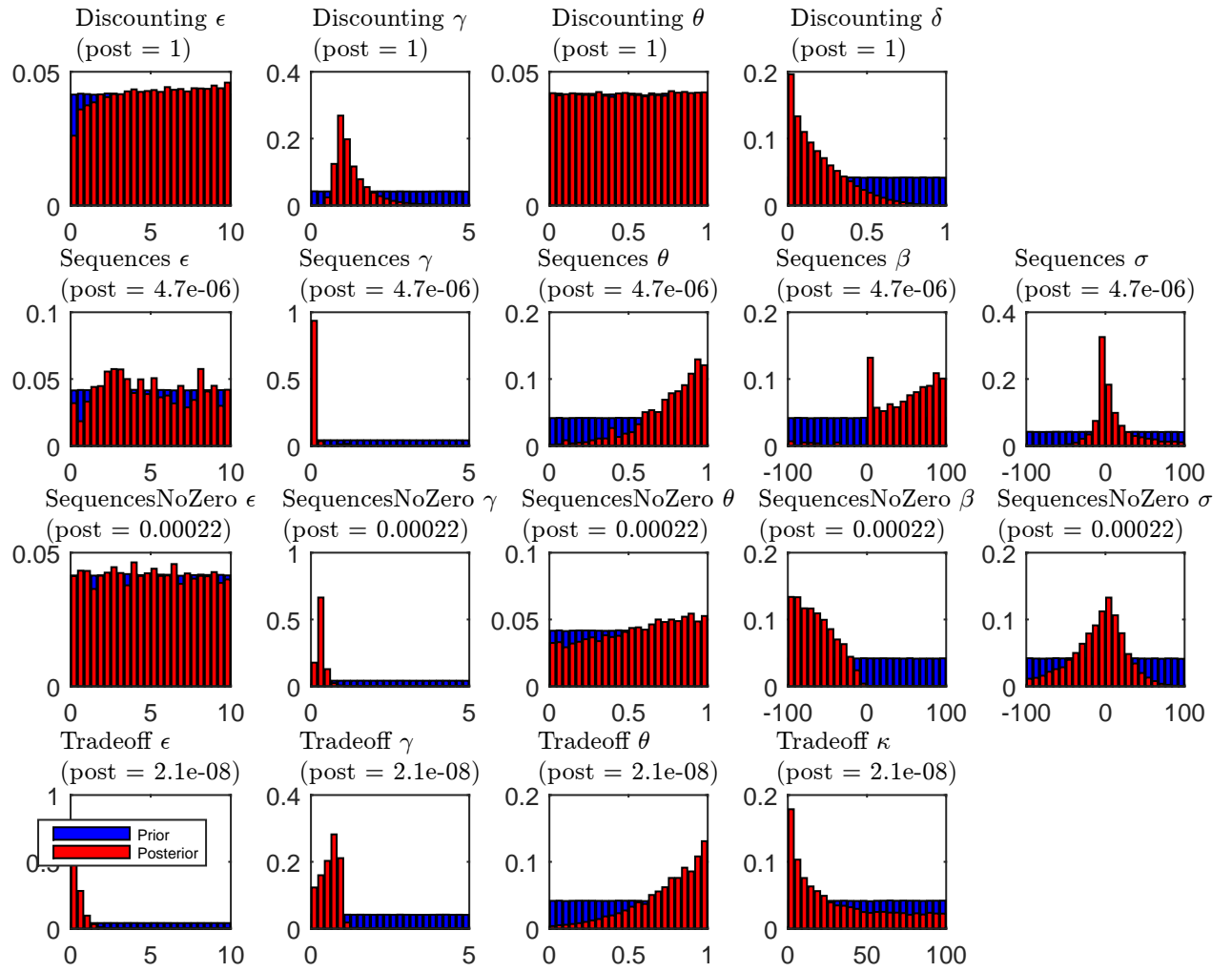

Supplement: Supplementary file 1 [file Scholten_Individuals.zip › plots/e29_p207_eg2_priors_and_posteriors.pdf]

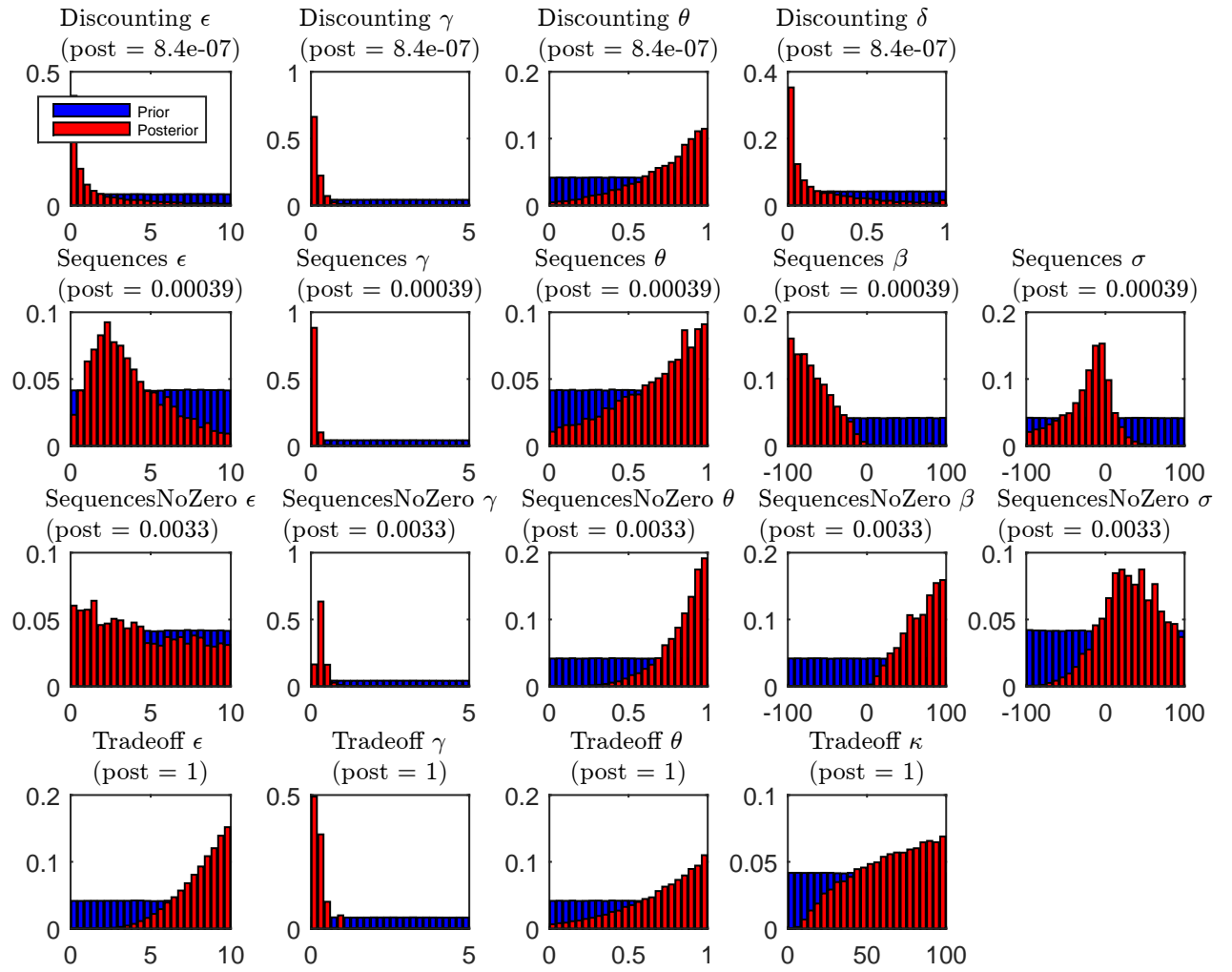

Supplement: Supplementary file 1 [file Scholten_Individuals.zip › plots/e29_p208_eg2_priors_and_posteriors.pdf]

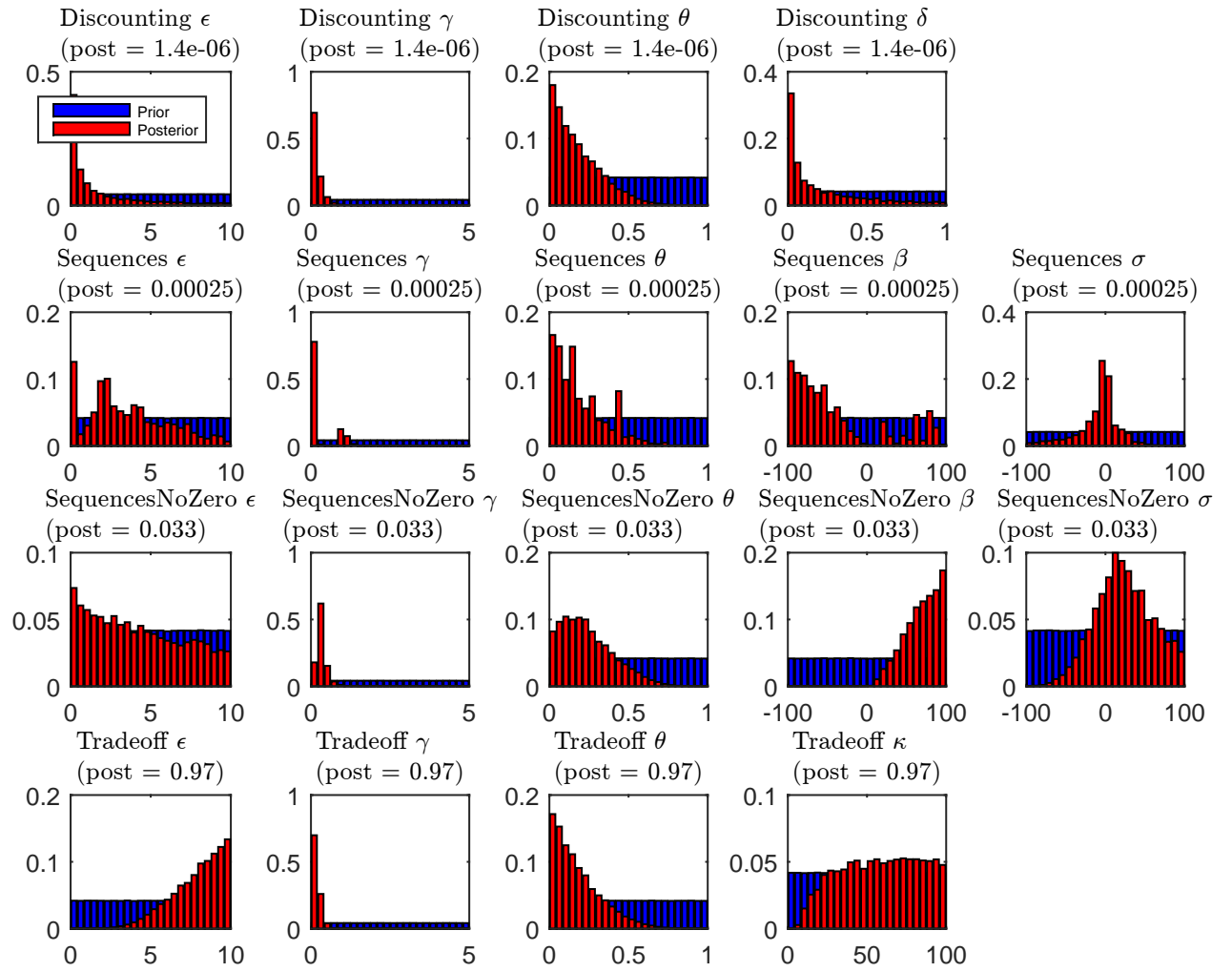

Supplement: Supplementary file 1 [file Scholten_Individuals.zip › plots/e29_p209_eg2_priors_and_posteriors.pdf]

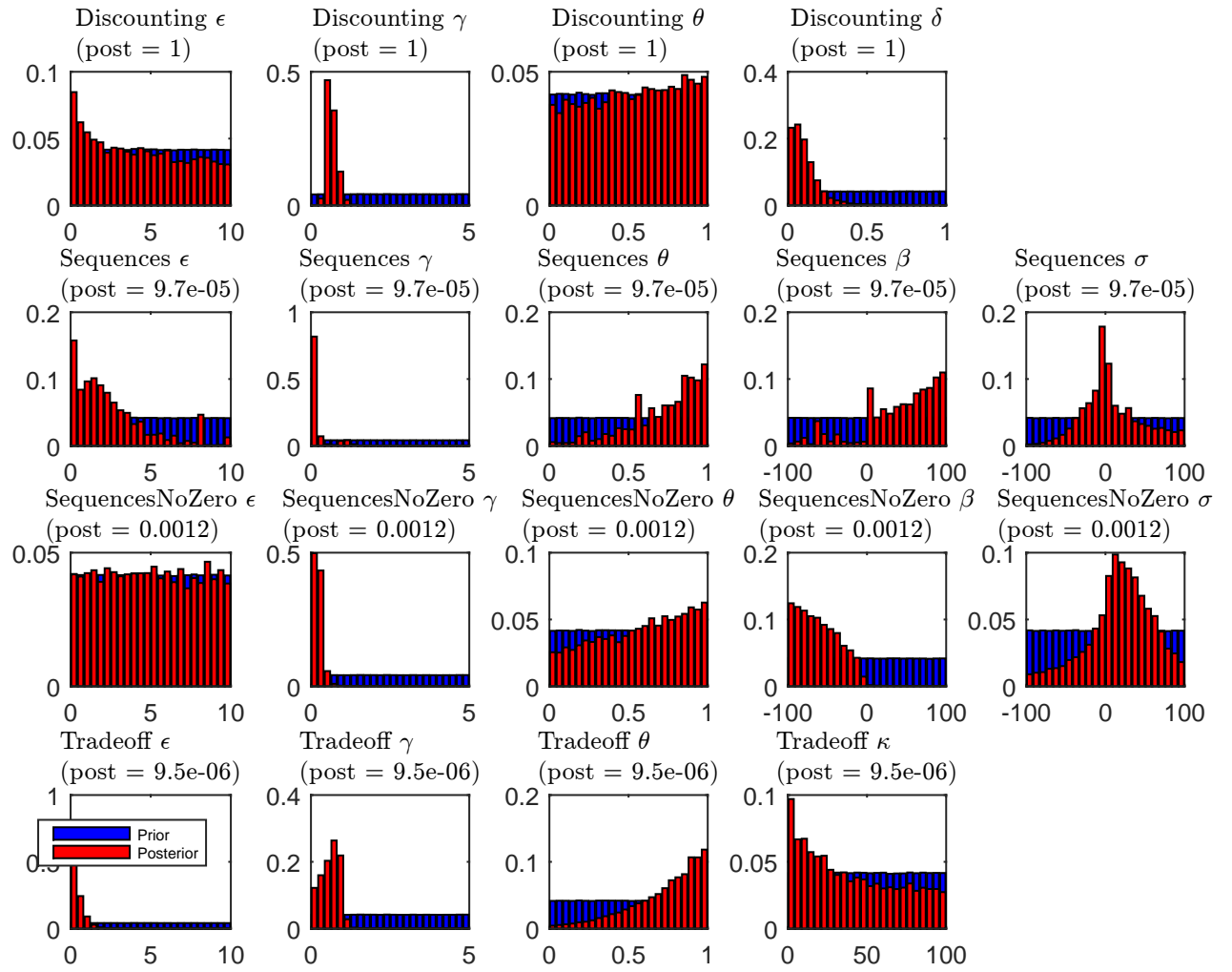

Supplement: Supplementary file 1 [file Scholten_Individuals.zip › plots/e29_p21_eg2_priors_and_posteriors.pdf]

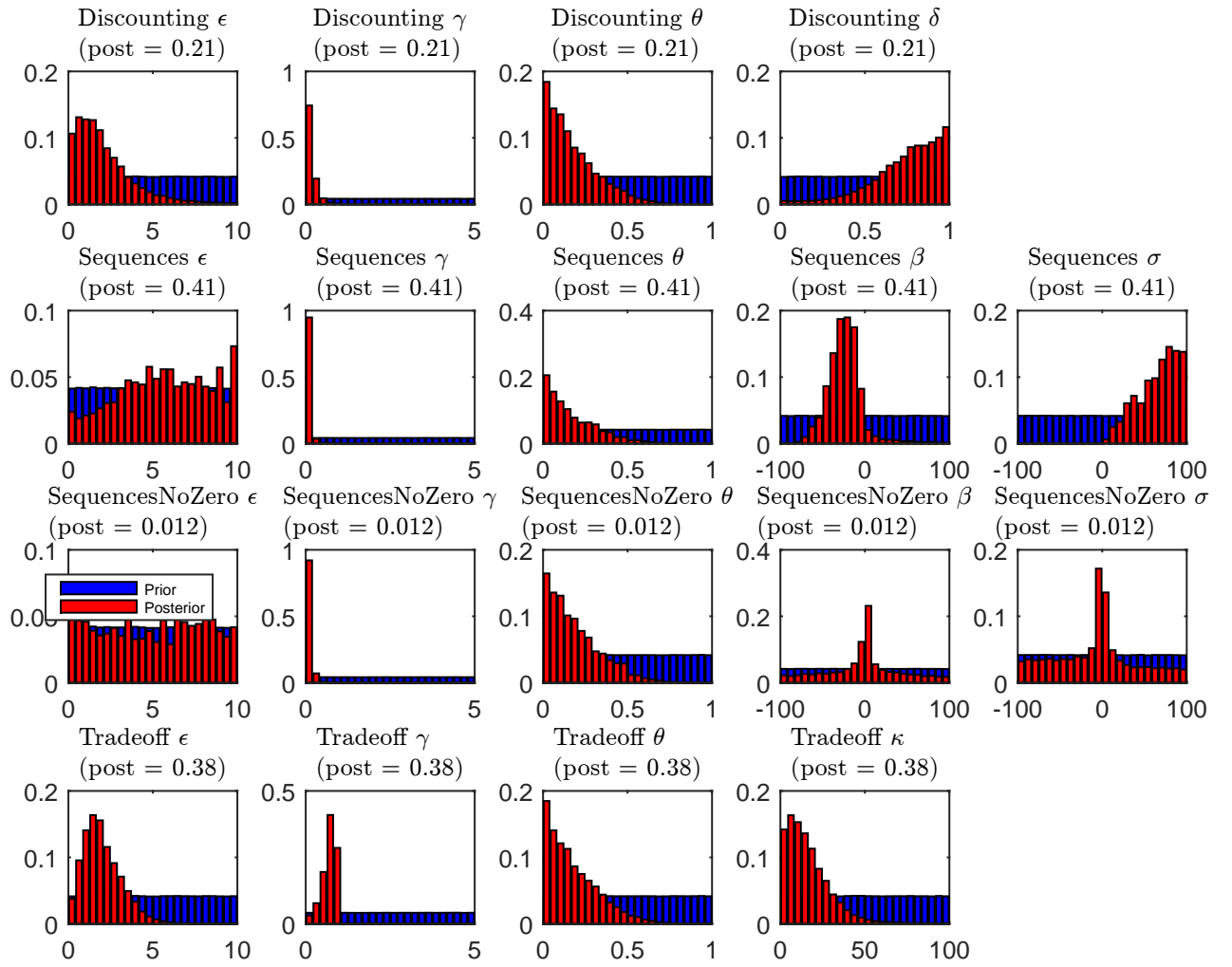

Supplement: Supplementary file 1 [file Scholten_Individuals.zip › plots/e29_p210_eg2_priors_and_posteriors.pdf]

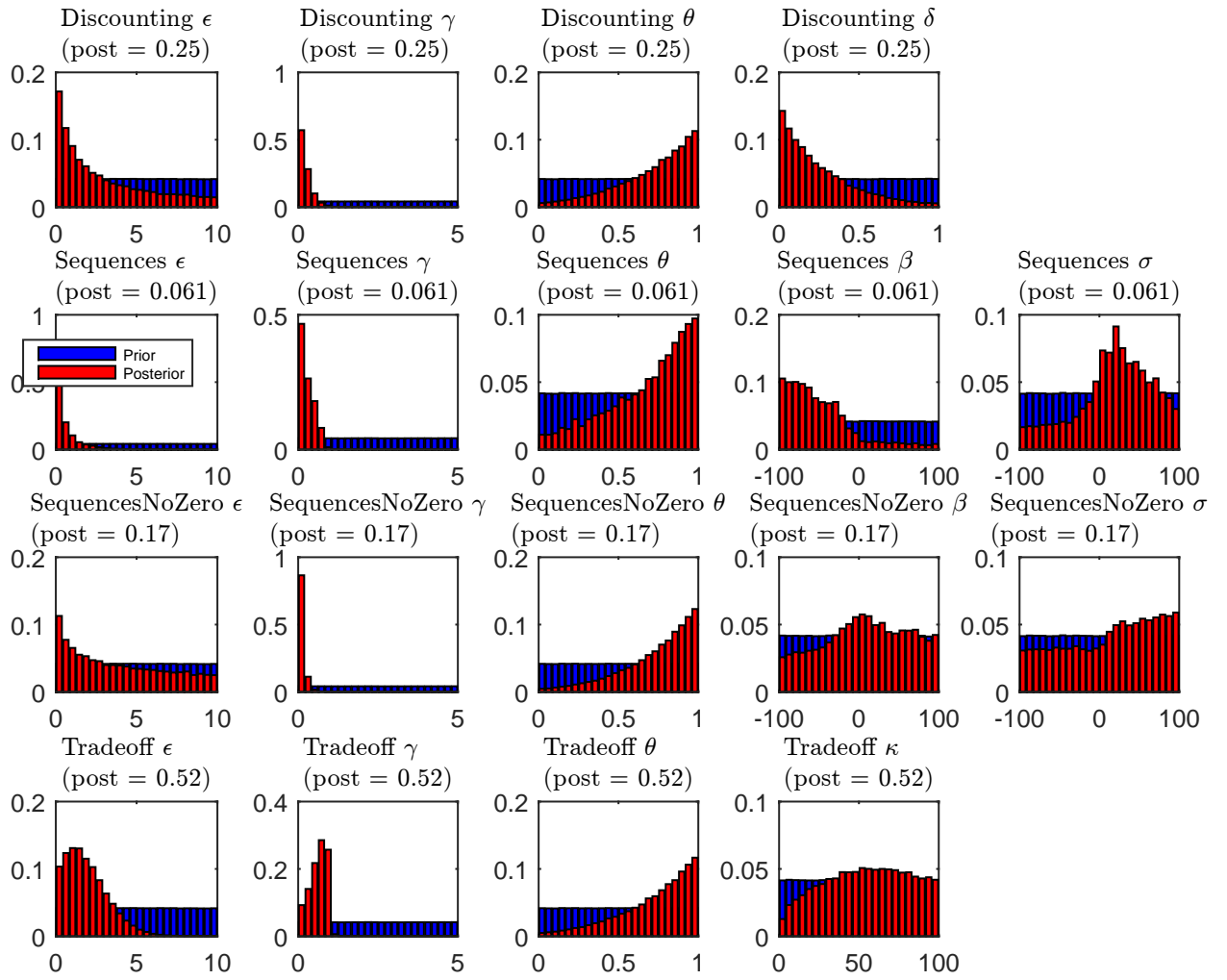

Supplement: Supplementary file 1 [file Scholten_Individuals.zip › plots/e29_p211_eg2_priors_and_posteriors.pdf]

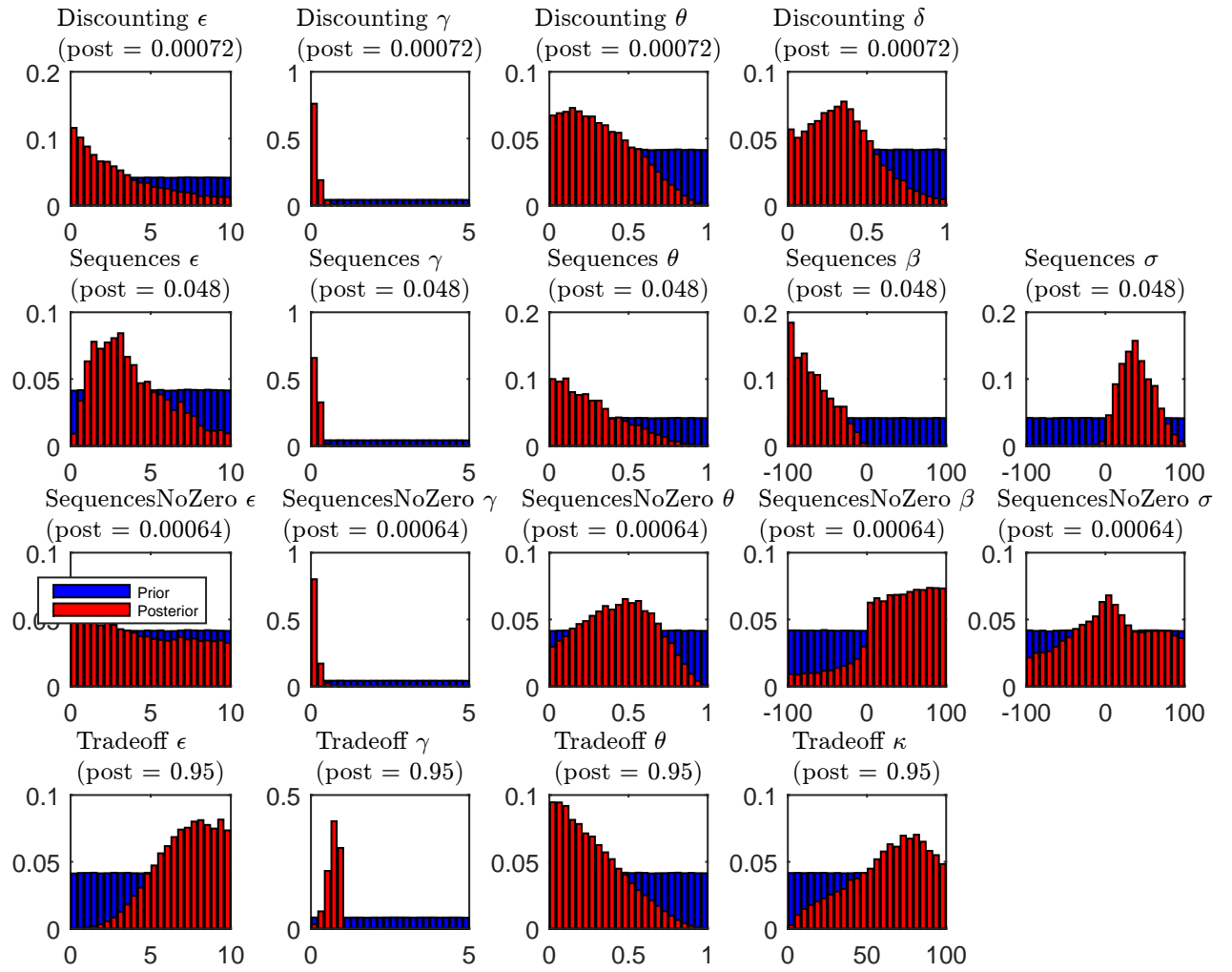

Supplement: Supplementary file 1 [file Scholten_Individuals.zip › plots/e29_p212_eg2_priors_and_posteriors.pdf]

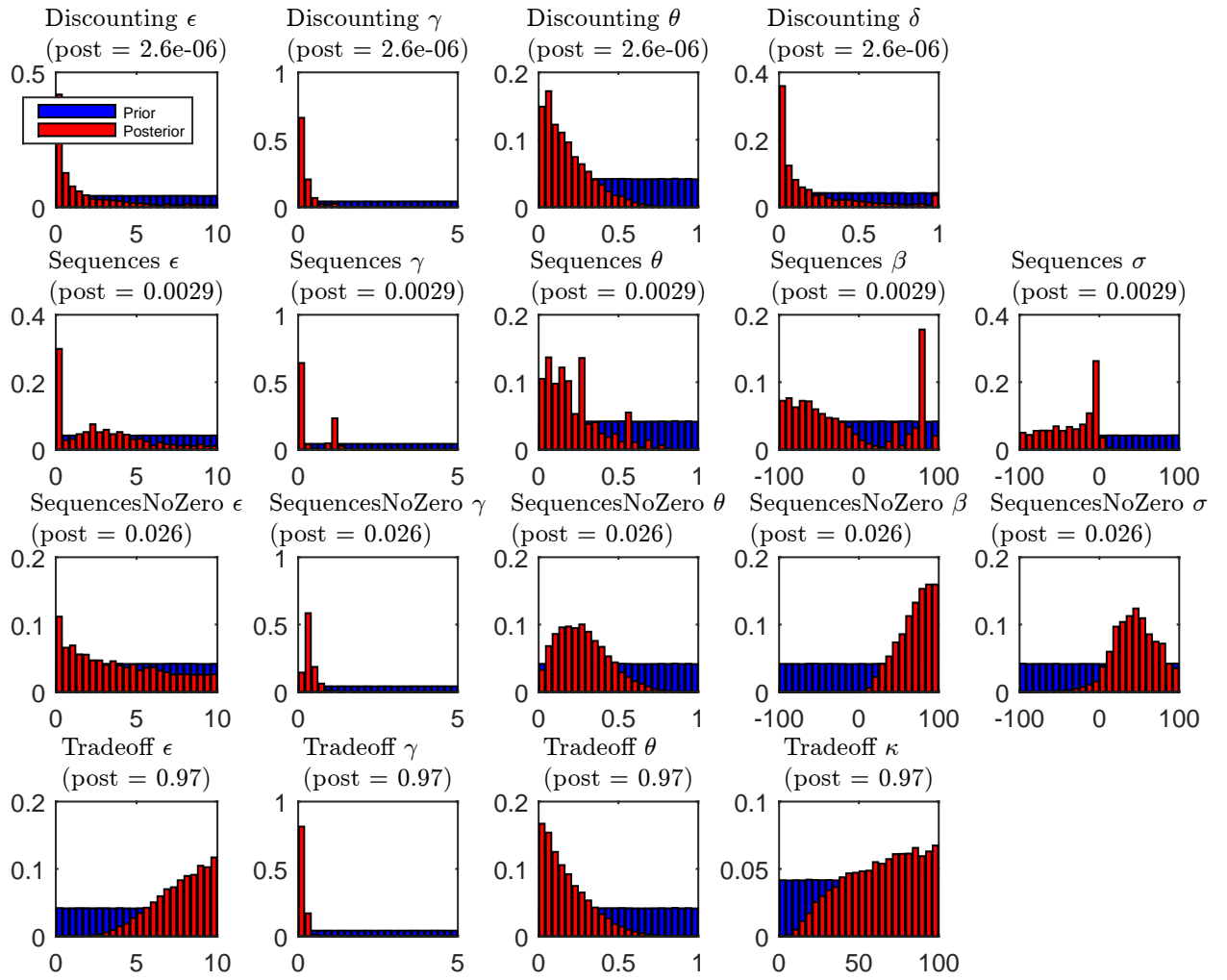

Supplement: Supplementary file 1 [file Scholten_Individuals.zip › plots/e29_p213_eg2_priors_and_posteriors.pdf]

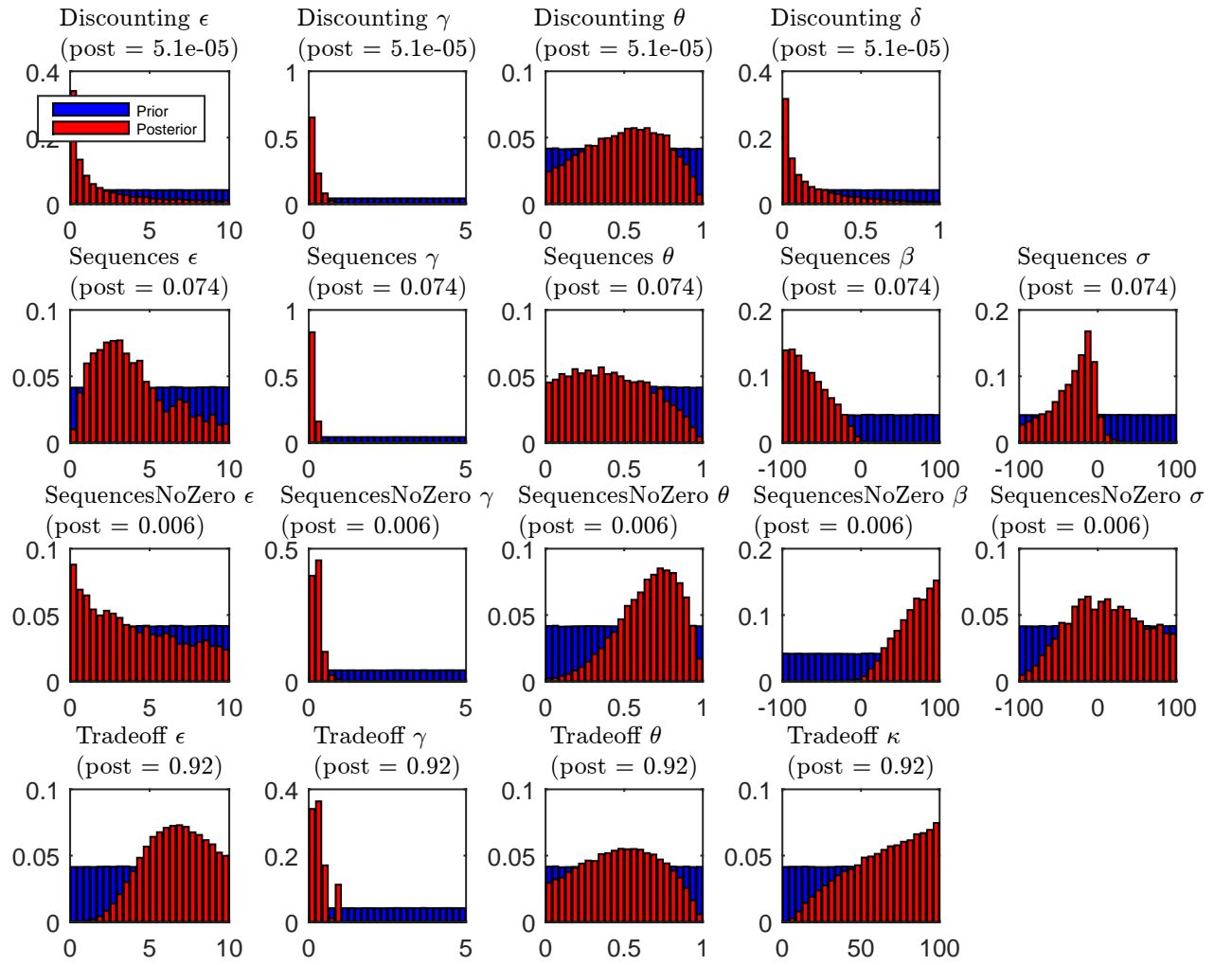

Supplement: Supplementary file 1 [file Scholten_Individuals.zip › plots/e29_p214_eg2_priors_and_posteriors.pdf]

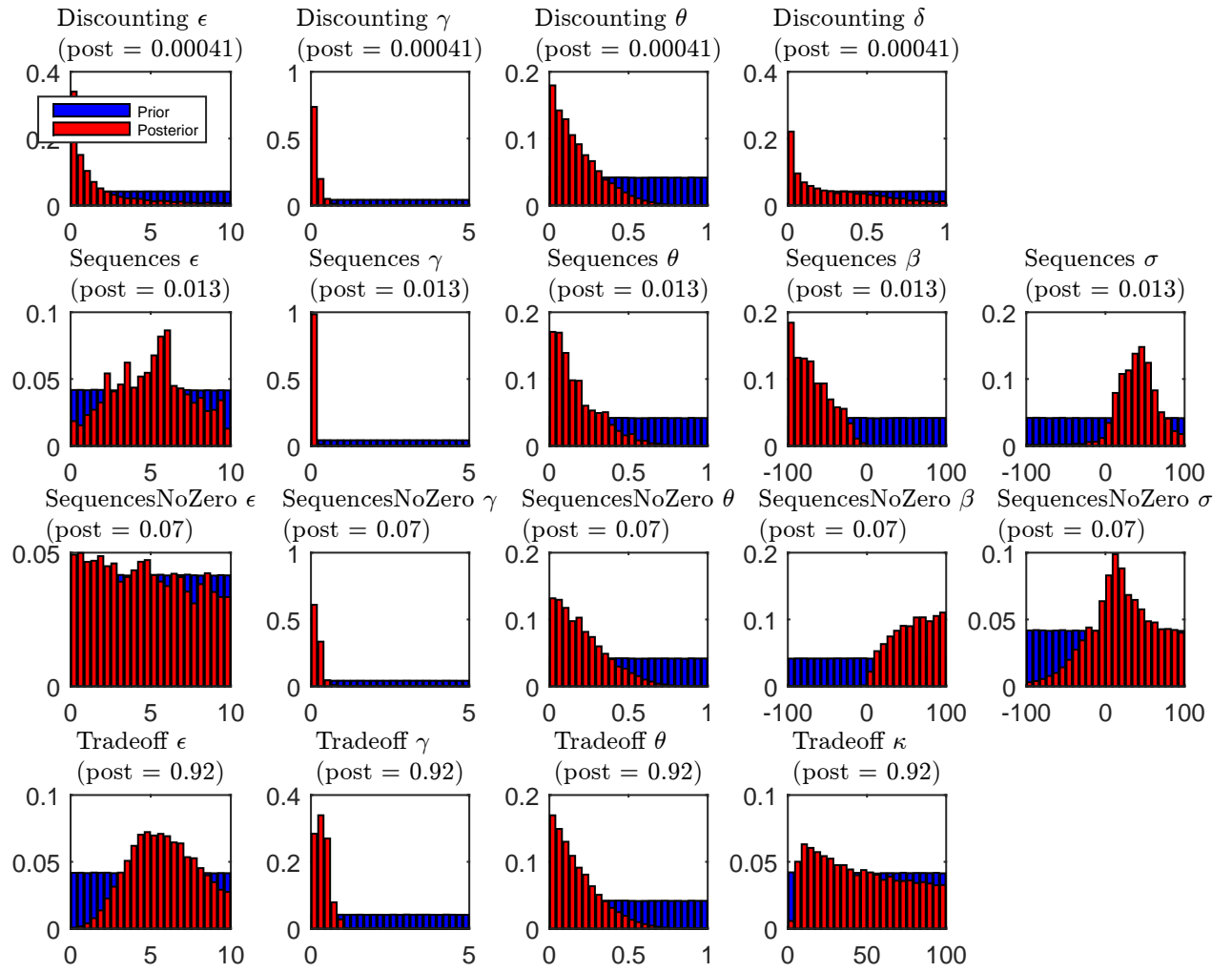

Supplement: Supplementary file 1 [file Scholten_Individuals.zip › plots/e29_p215_eg2_priors_and_posteriors.pdf]

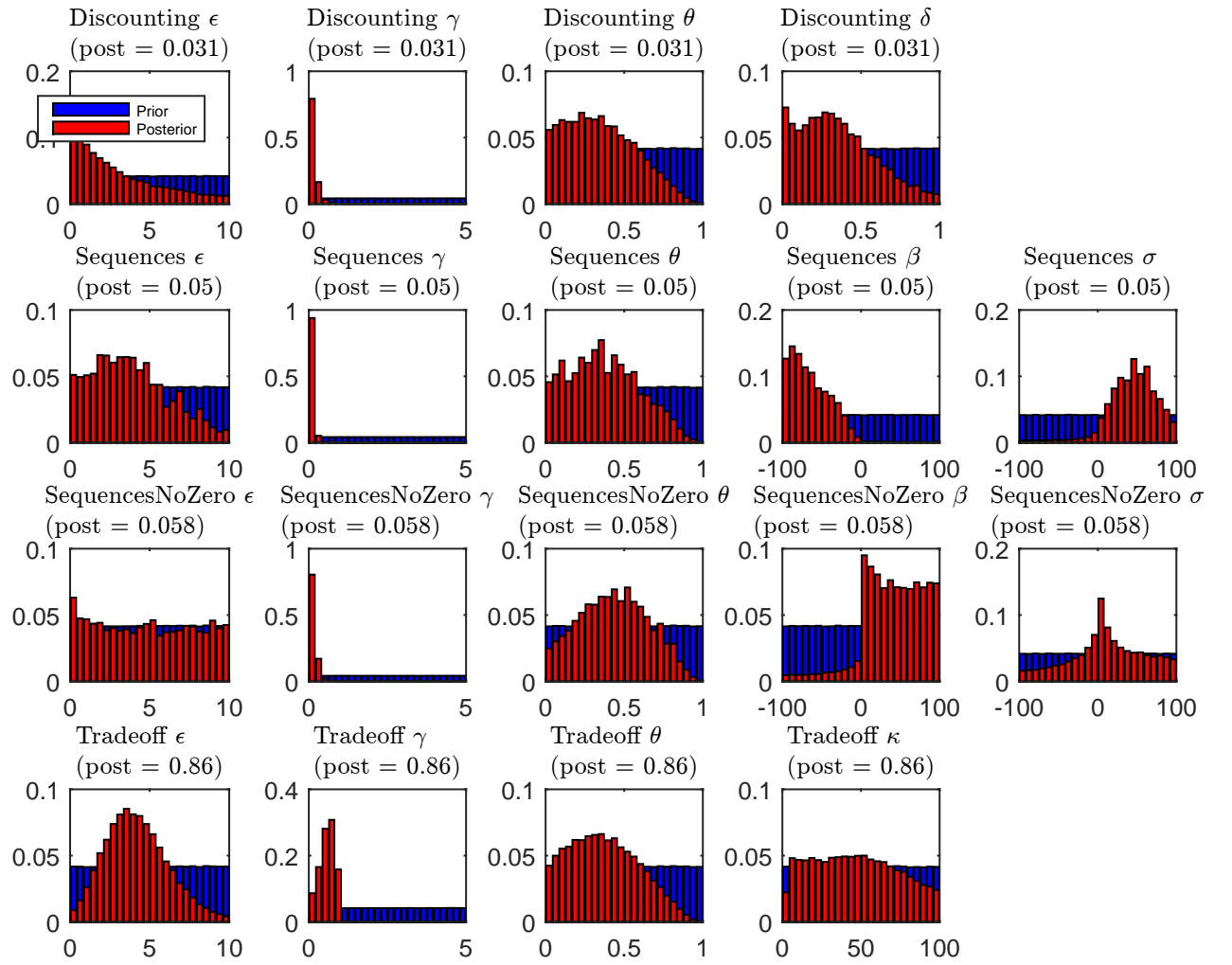

Supplement: Supplementary file 1 [file Scholten_Individuals.zip › plots/e29_p216_eg2_priors_and_posteriors.pdf]

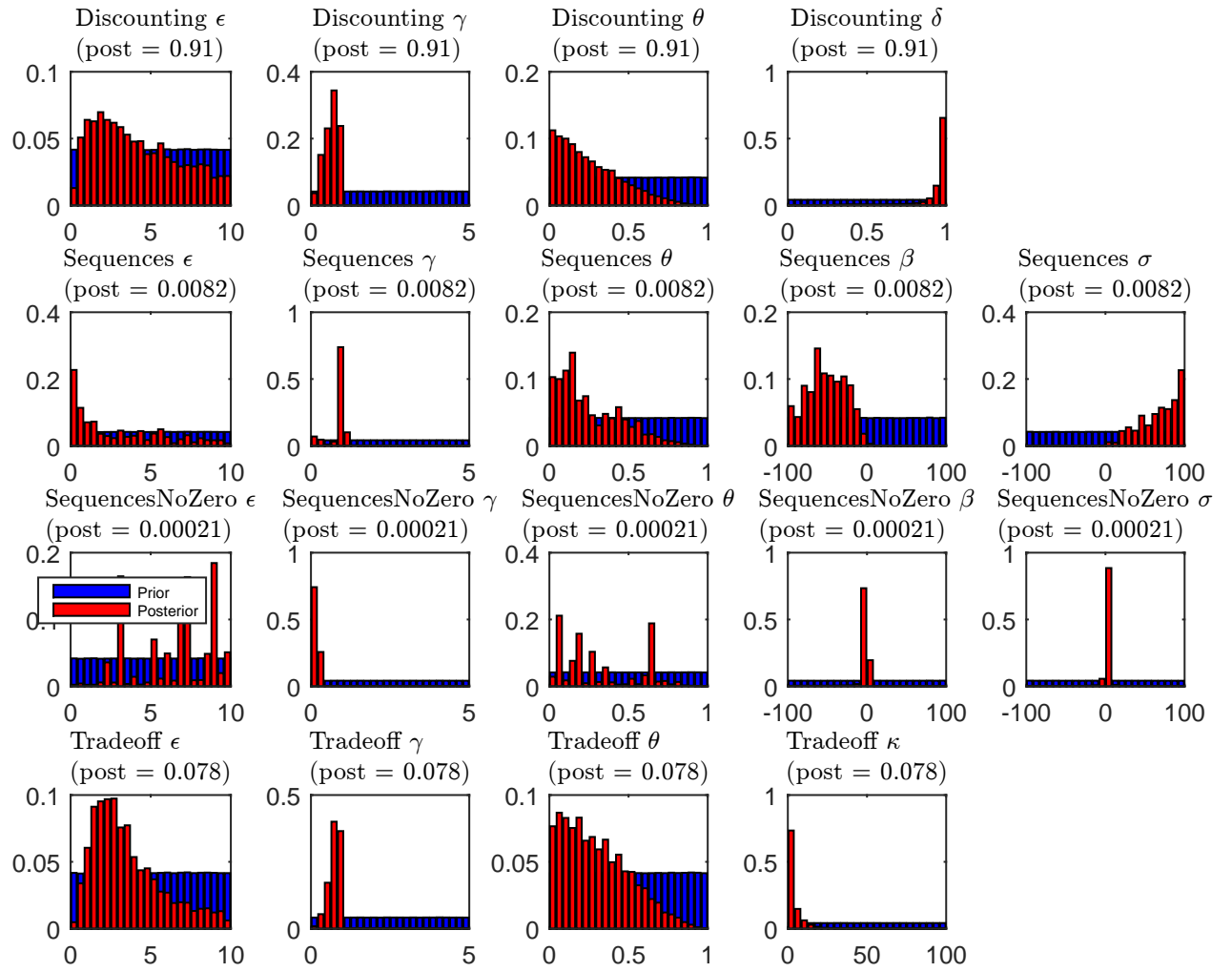

Supplement: Supplementary file 1 [file Scholten_Individuals.zip › plots/e29_p217_eg2_priors_and_posteriors.pdf]

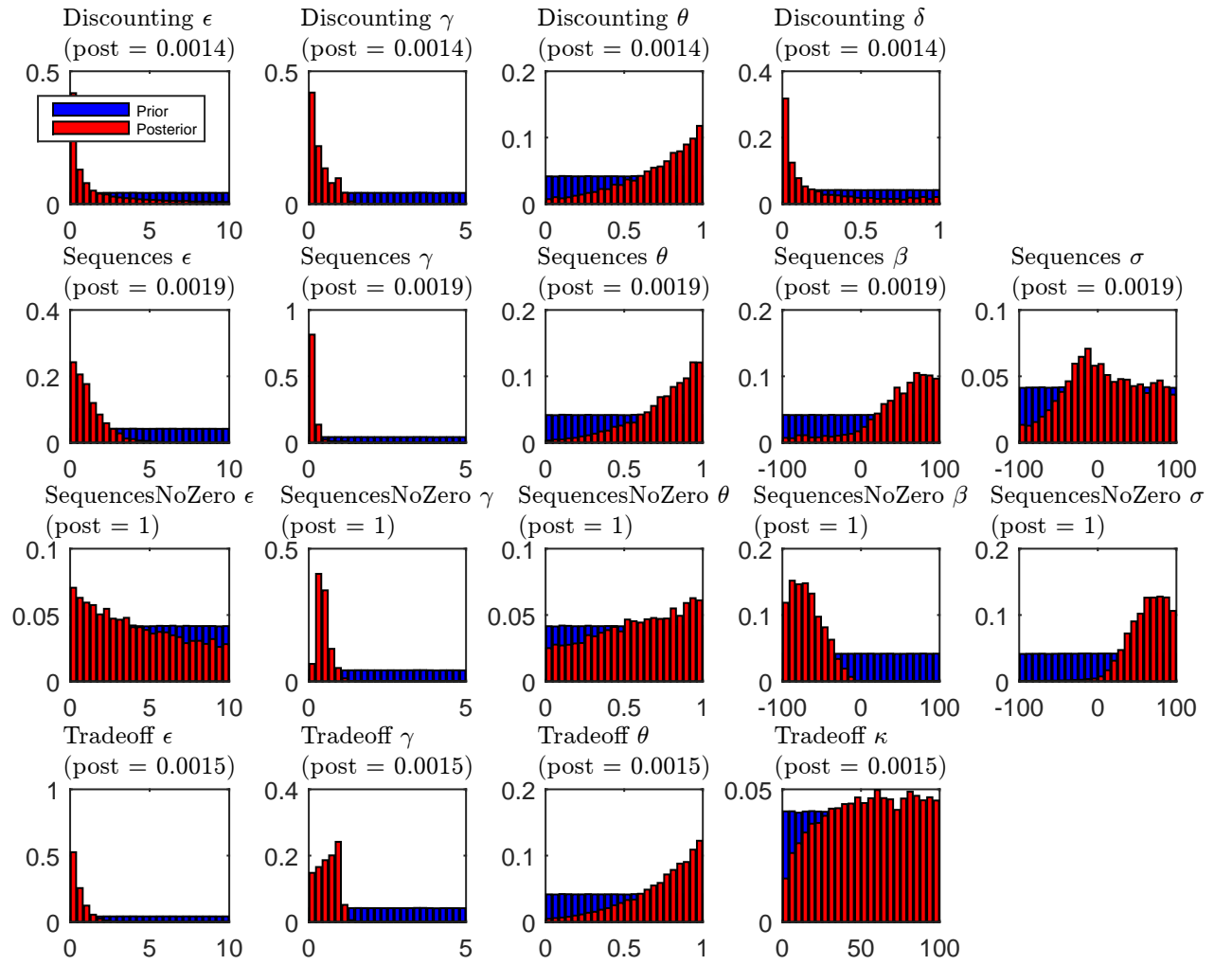

Supplement: Supplementary file 1 [file Scholten_Individuals.zip › plots/e29_p218_eg2_priors_and_posteriors.pdf]

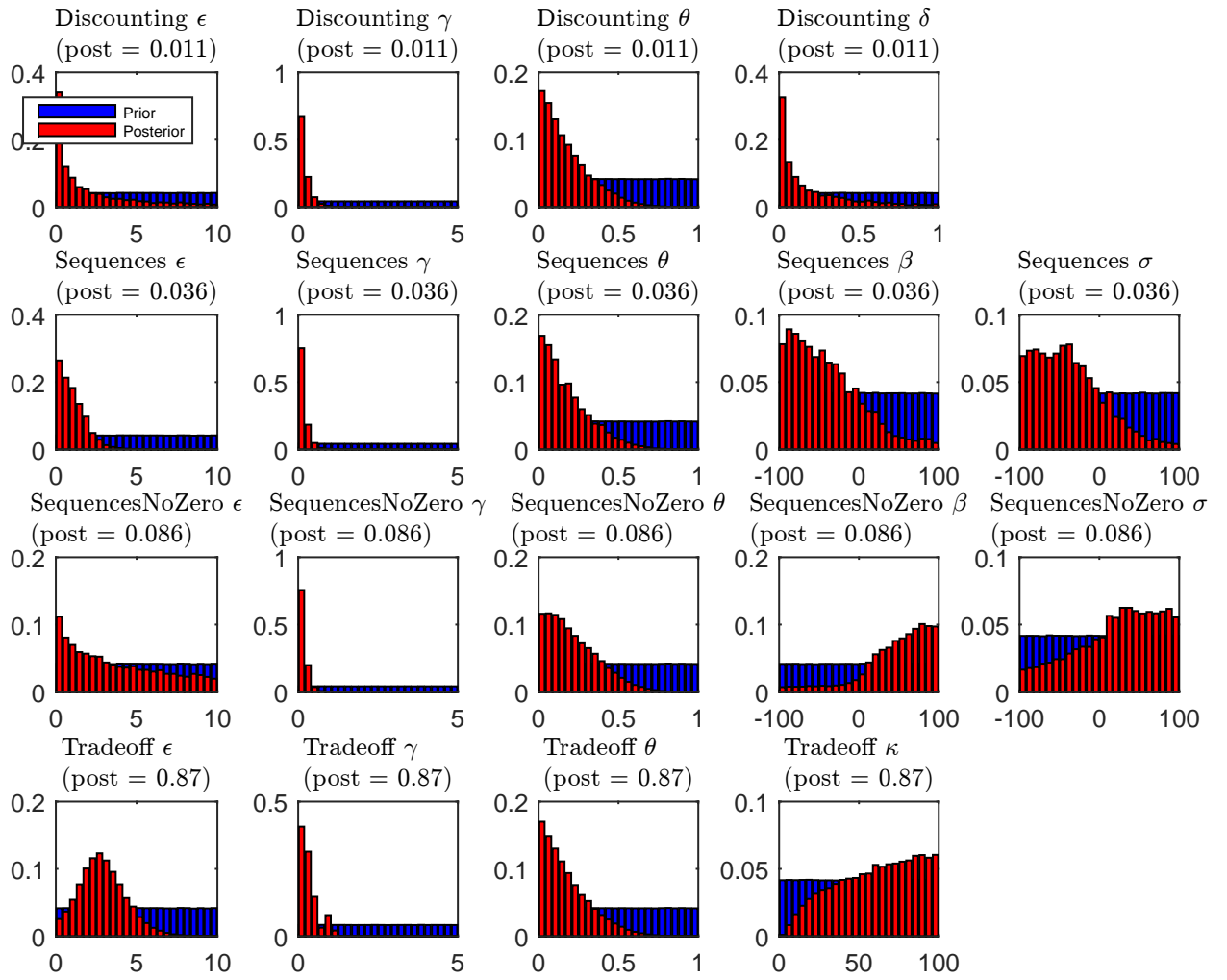

Supplement: Supplementary file 1 [file Scholten_Individuals.zip › plots/e29_p219_eg2_priors_and_posteriors.pdf]

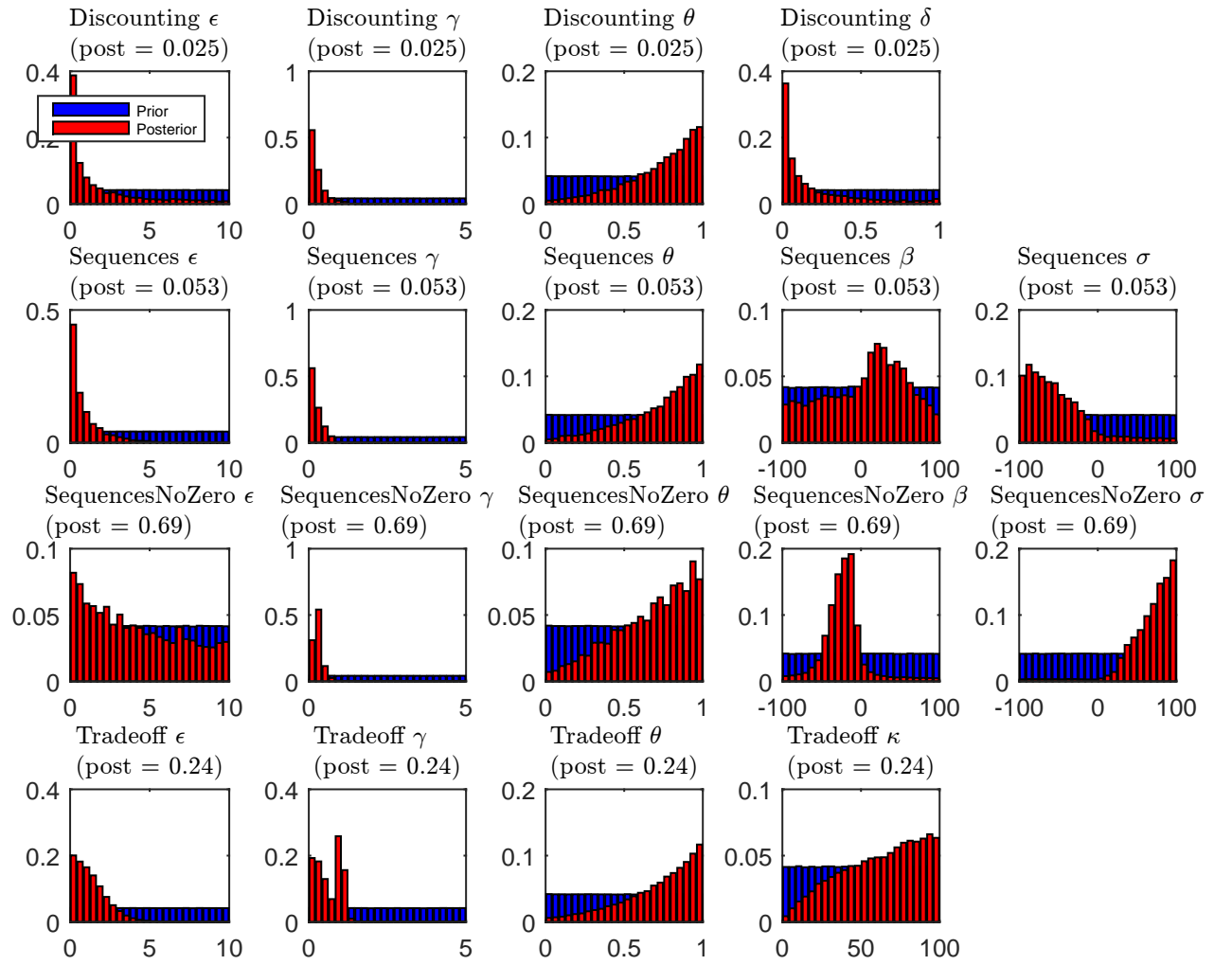

Supplement: Supplementary file 1 [file Scholten_Individuals.zip › plots/e29_p22_eg2_priors_and_posteriors.pdf]

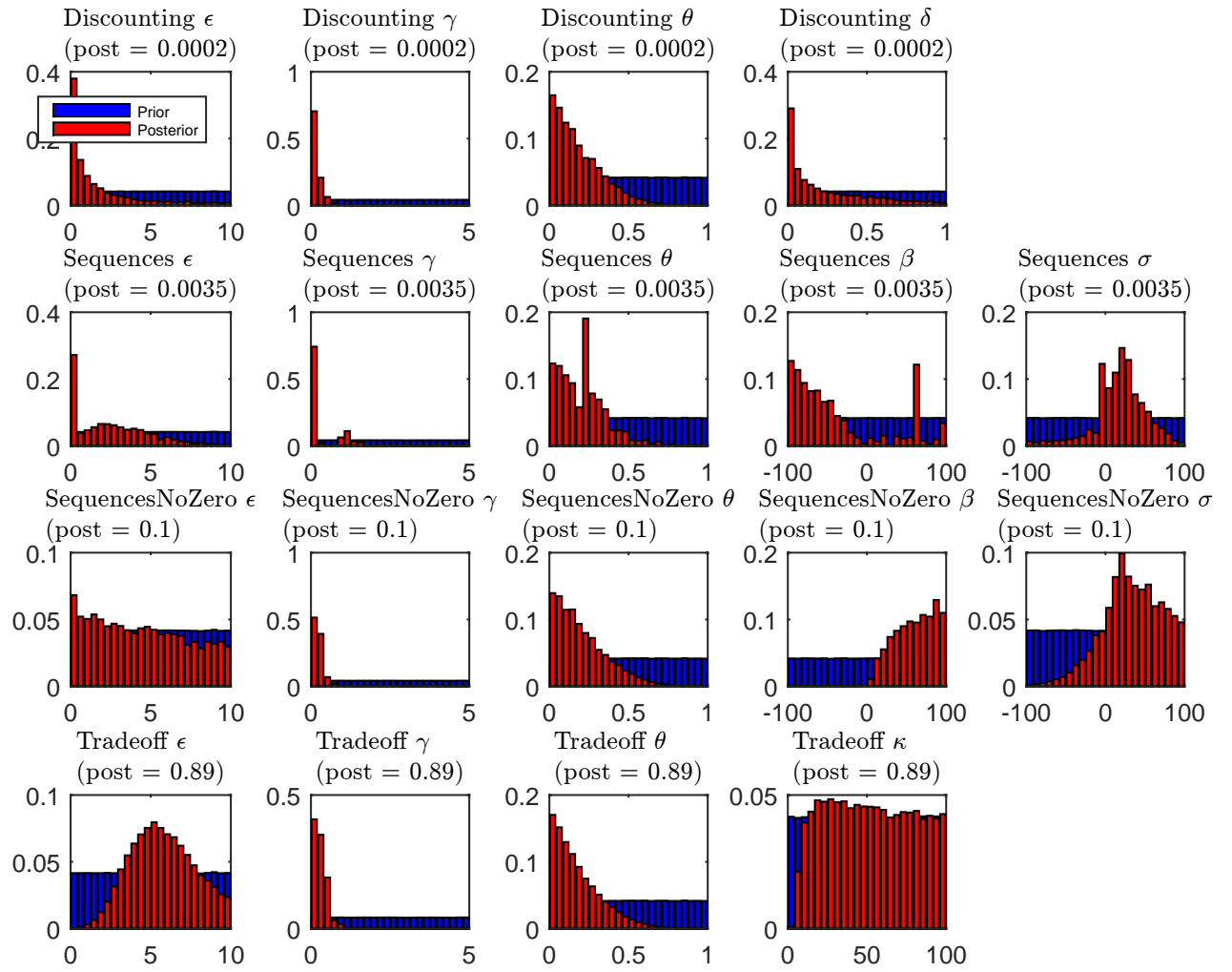

Supplement: Supplementary file 1 [file Scholten_Individuals.zip › plots/e29_p220_eg2_priors_and_posteriors.pdf]

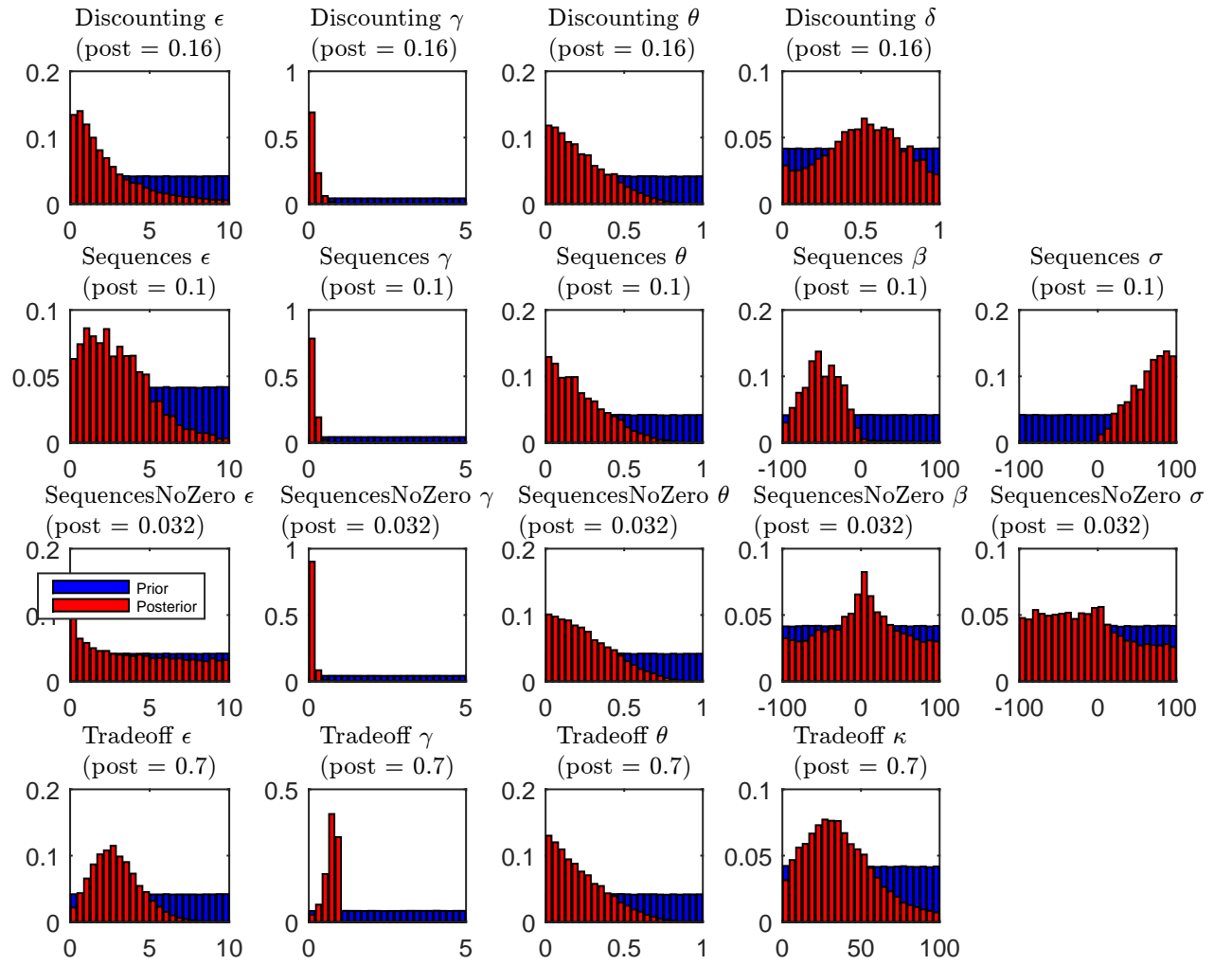

Supplement: Supplementary file 1 [file Scholten_Individuals.zip › plots/e29_p221_eg2_priors_and_posteriors.pdf]

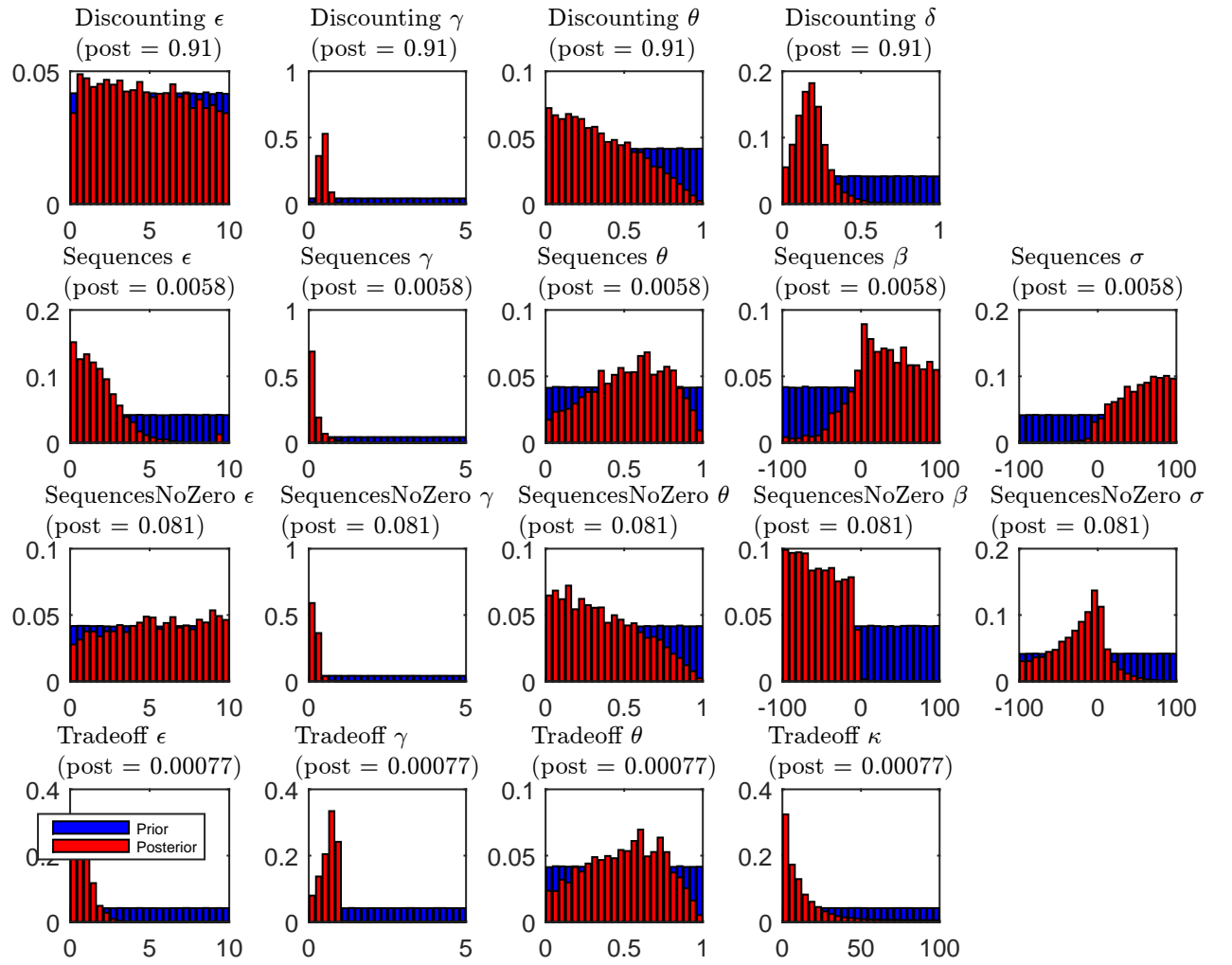

Supplement: Supplementary file 1 [file Scholten_Individuals.zip › plots/e29_p222_eg2_priors_and_posteriors.pdf]

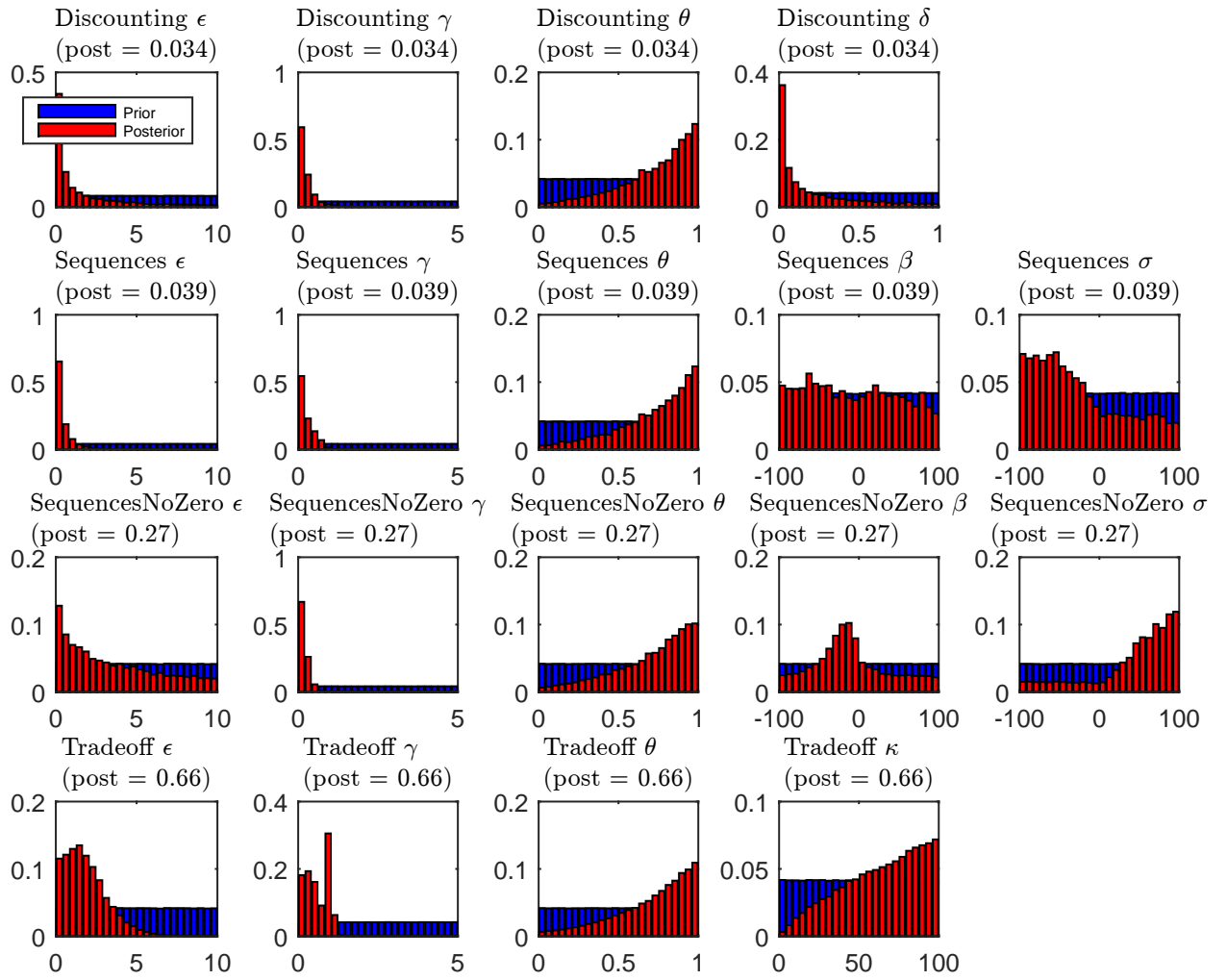

Supplement: Supplementary file 1 [file Scholten_Individuals.zip › plots/e29_p223_eg2_priors_and_posteriors.pdf]

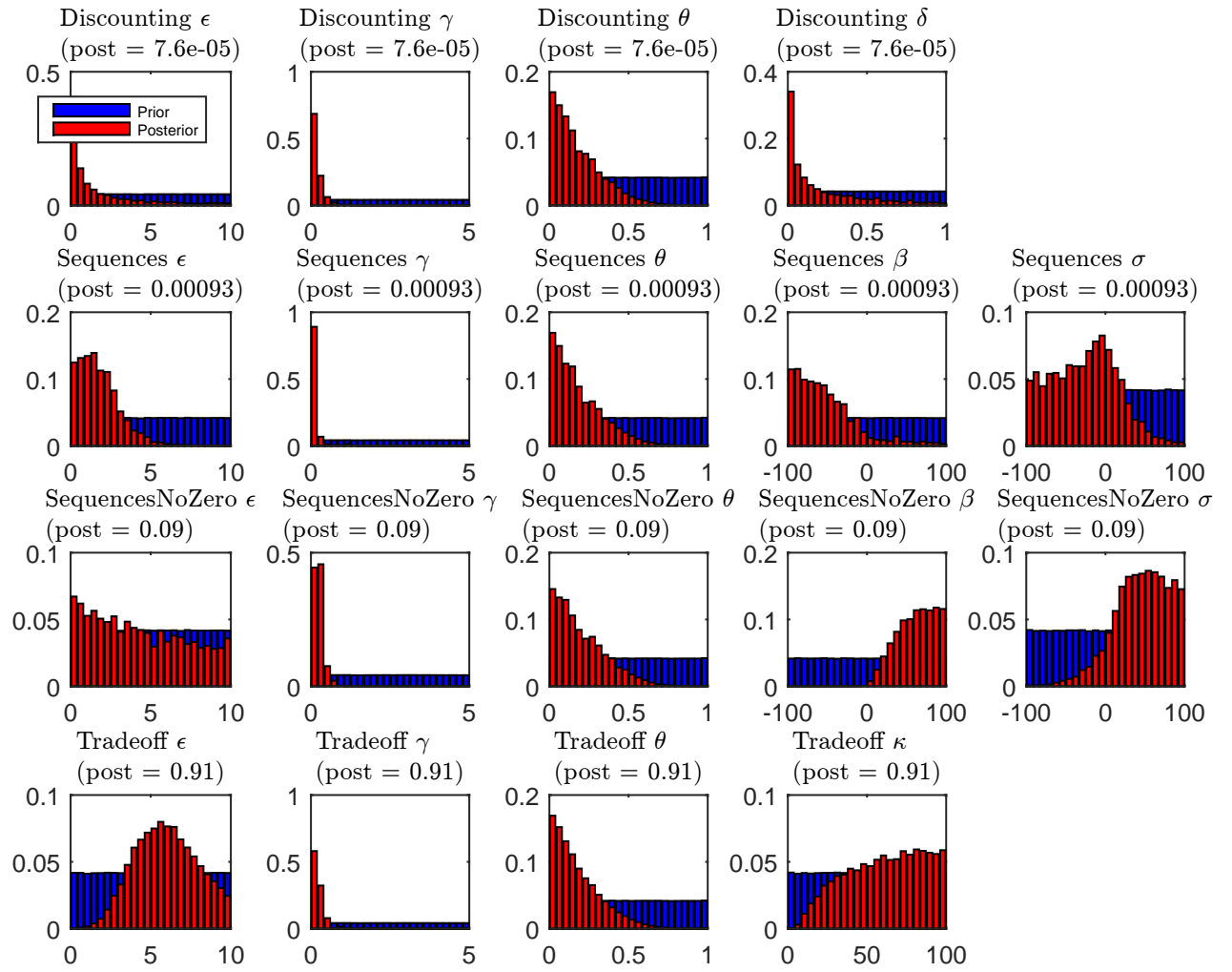

Supplement: Supplementary file 1 [file Scholten_Individuals.zip › plots/e29_p224_eg2_priors_and_posteriors.pdf]

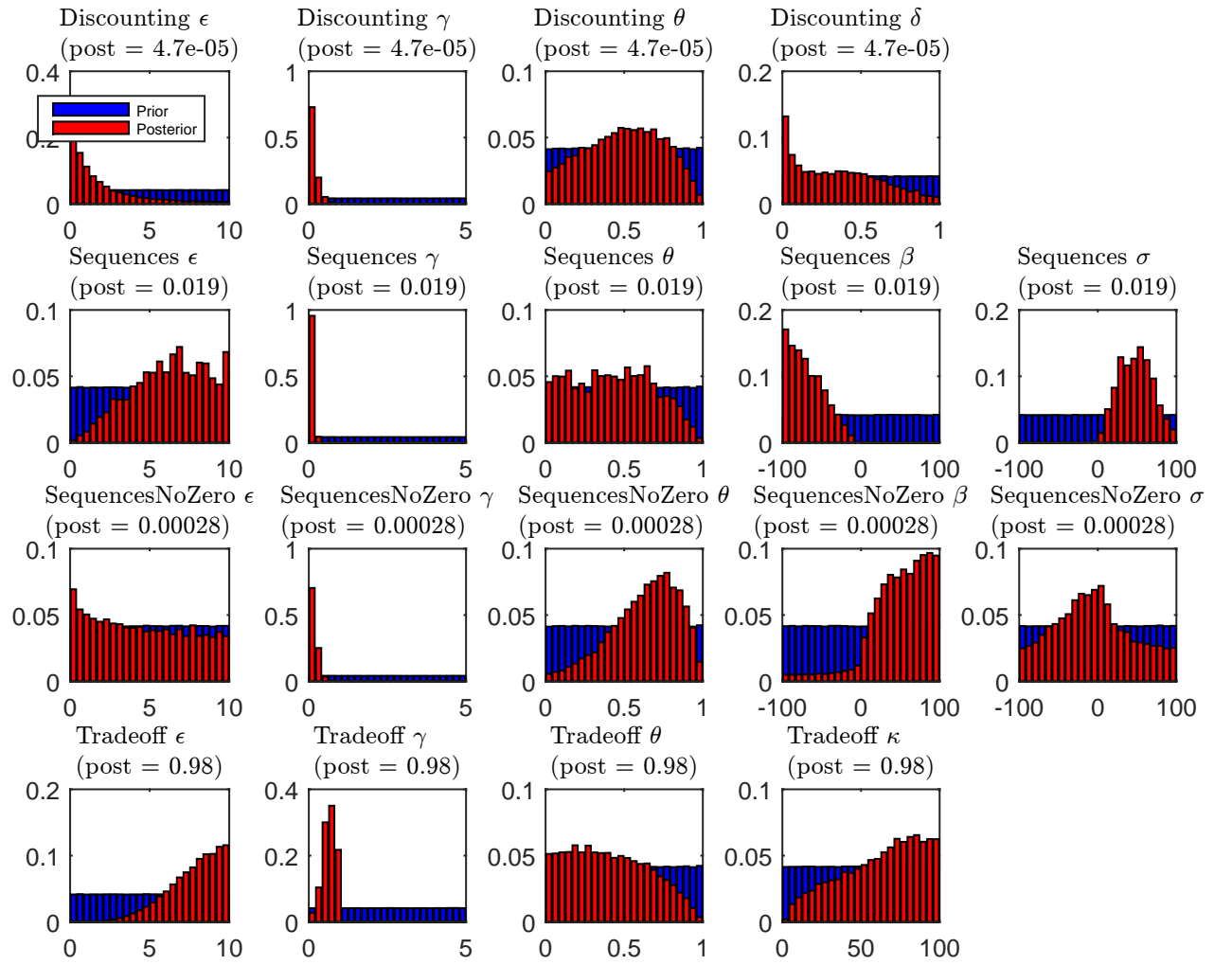

Supplement: Supplementary file 1 [file Scholten_Individuals.zip › plots/e29_p225_eg2_priors_and_posteriors.pdf]

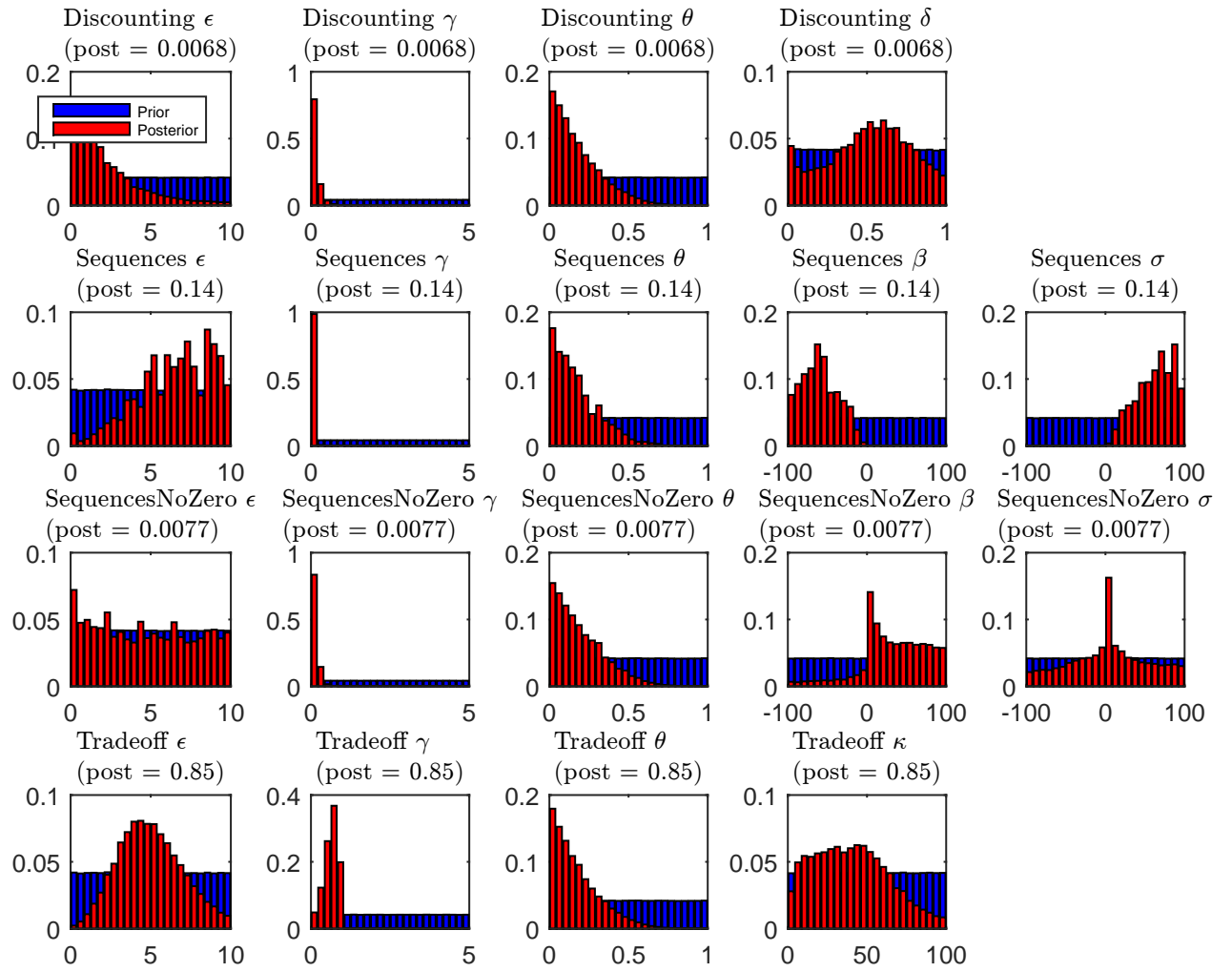

Supplement: Supplementary file 1 [file Scholten_Individuals.zip › plots/e29_p226_eg2_priors_and_posteriors.pdf]

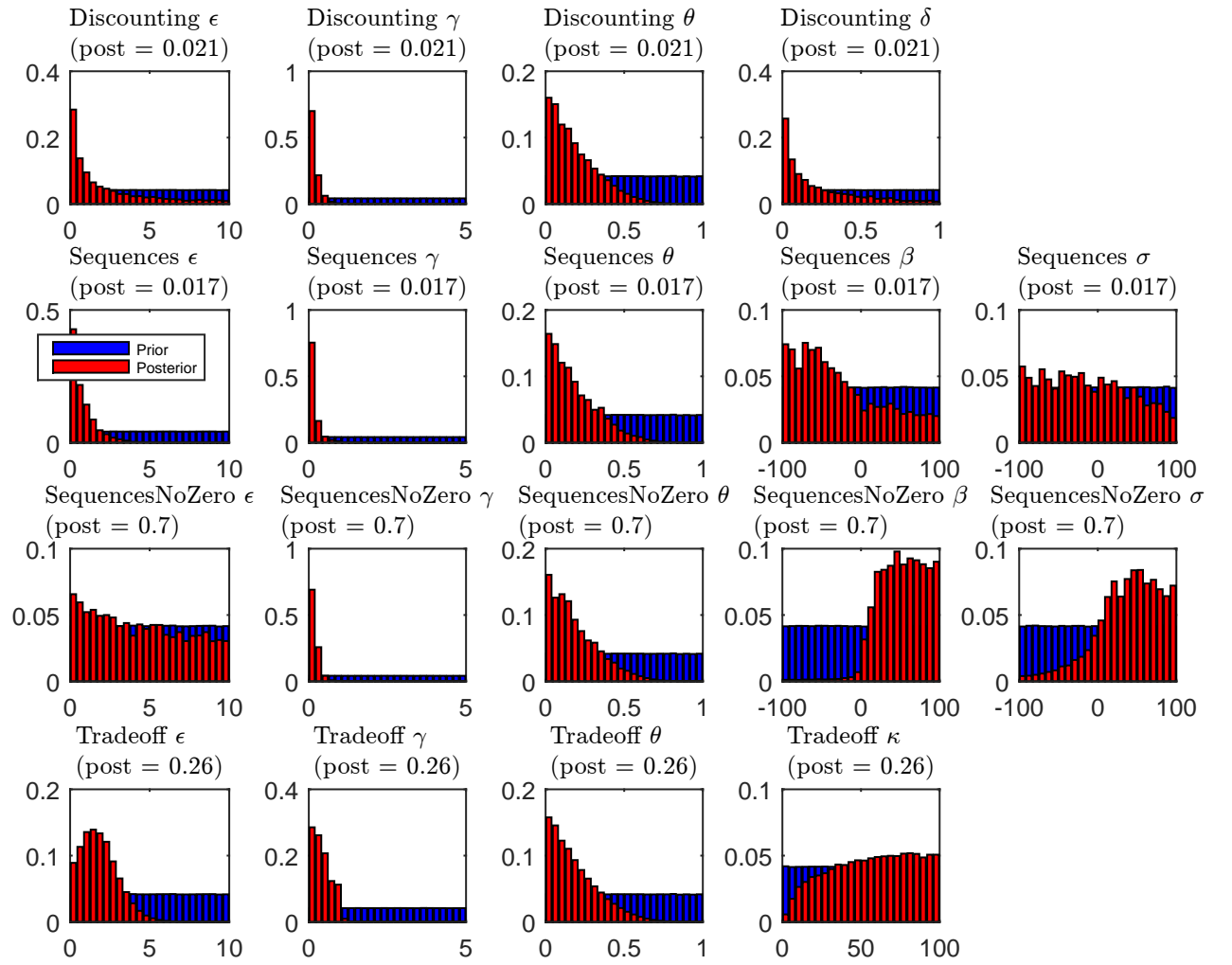

Supplement: Supplementary file 1 [file Scholten_Individuals.zip › plots/e29_p227_eg2_priors_and_posteriors.pdf]

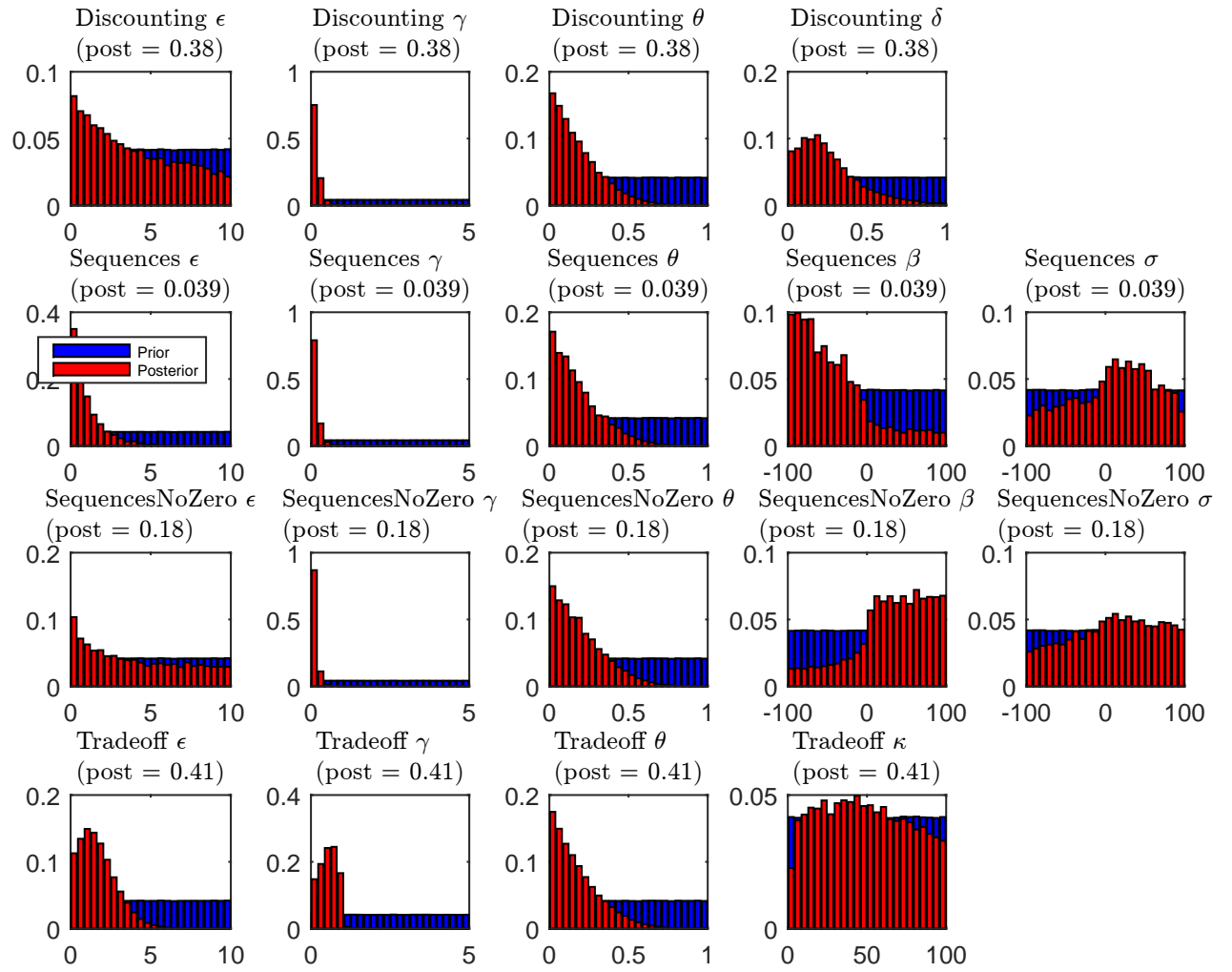

Supplement: Supplementary file 1 [file Scholten_Individuals.zip › plots/e29_p228_eg2_priors_and_posteriors.pdf]

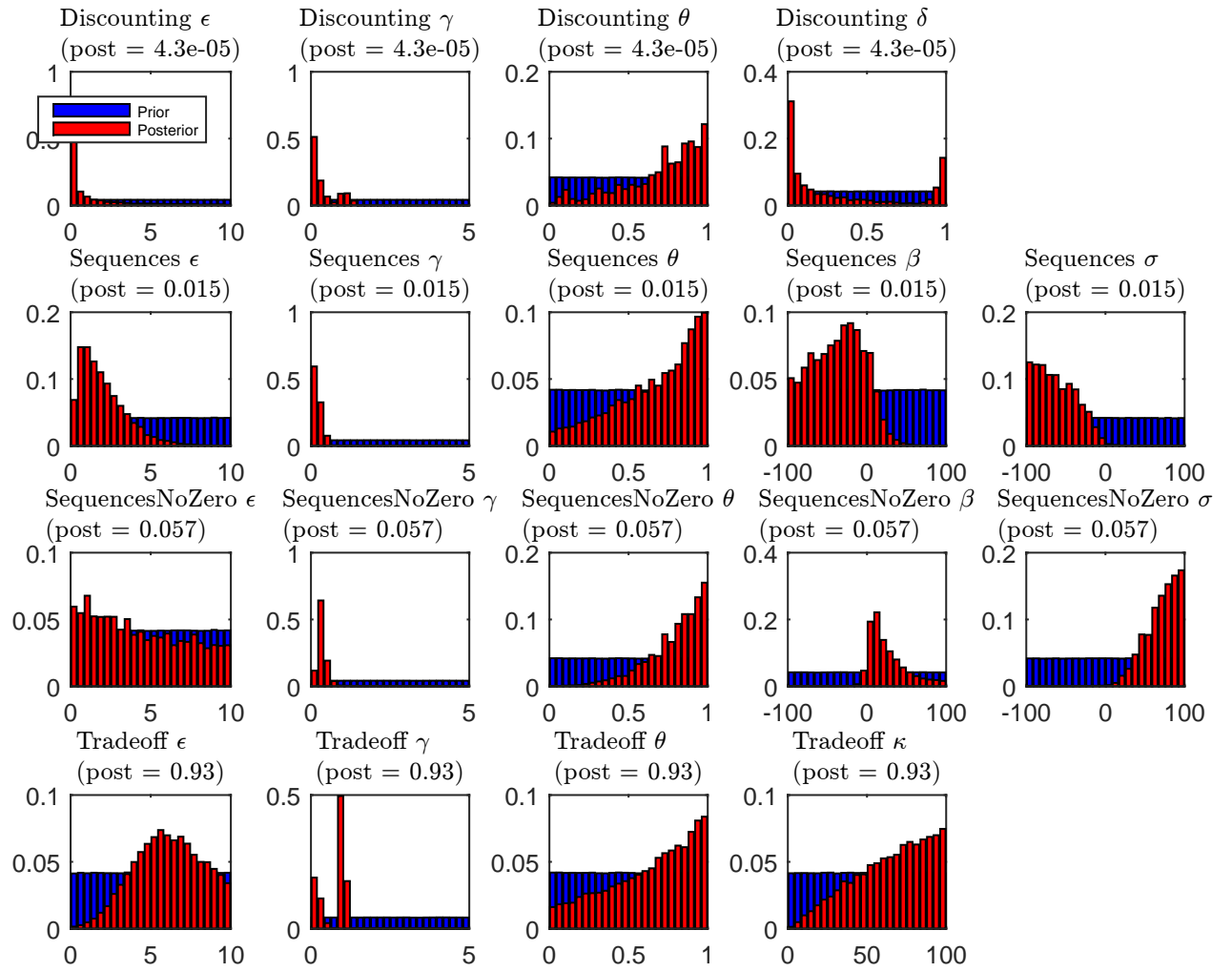

Supplement: Supplementary file 1 [file Scholten_Individuals.zip › plots/e29_p229_eg2_priors_and_posteriors.pdf]

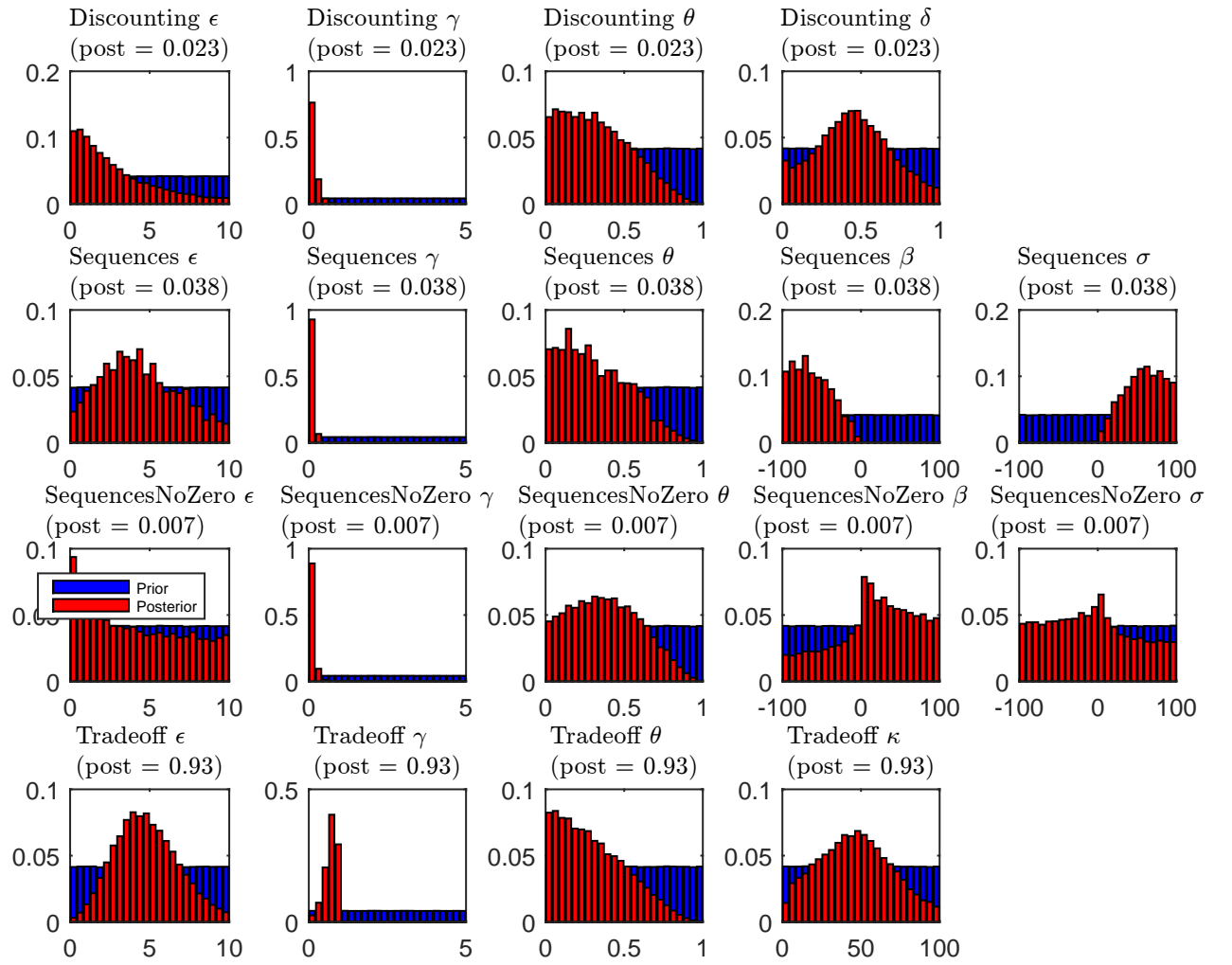

Supplement: Supplementary file 1 [file Scholten_Individuals.zip › plots/e29_p23_eg2_priors_and_posteriors.pdf]

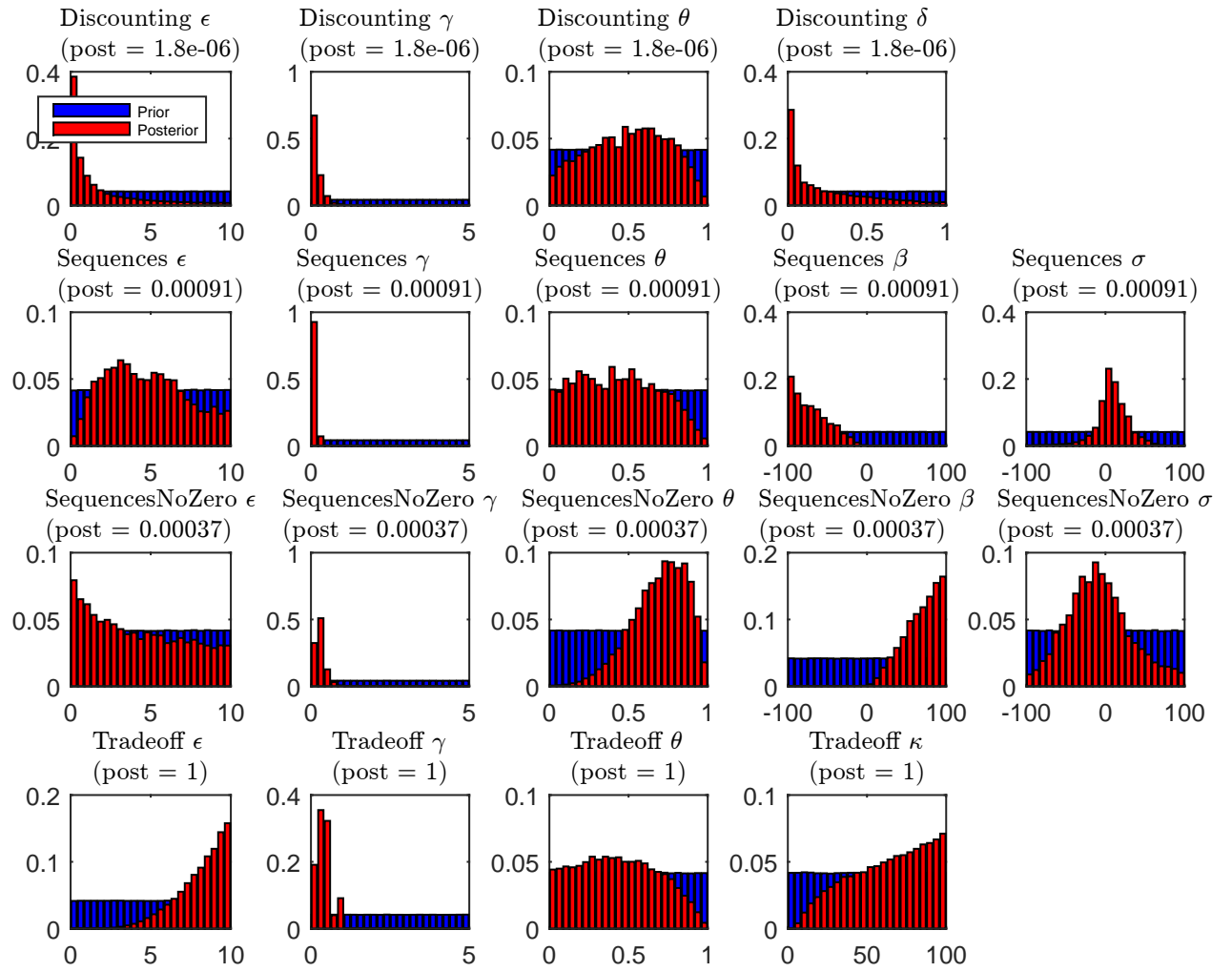

Supplement: Supplementary file 1 [file Scholten_Individuals.zip › plots/e29_p230_eg2_priors_and_posteriors.pdf]

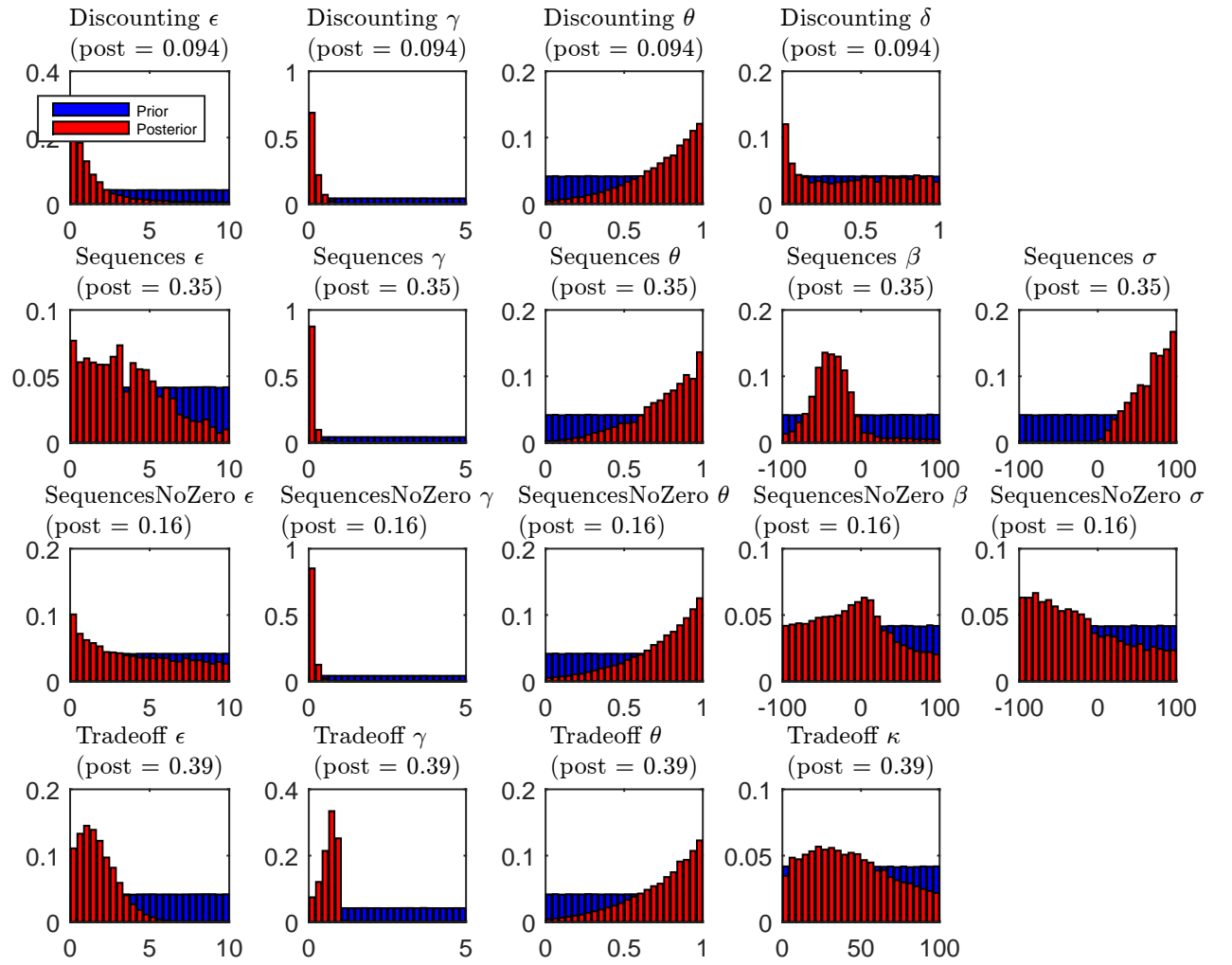

Supplement: Supplementary file 1 [file Scholten_Individuals.zip › plots/e29_p231_eg2_priors_and_posteriors.pdf]

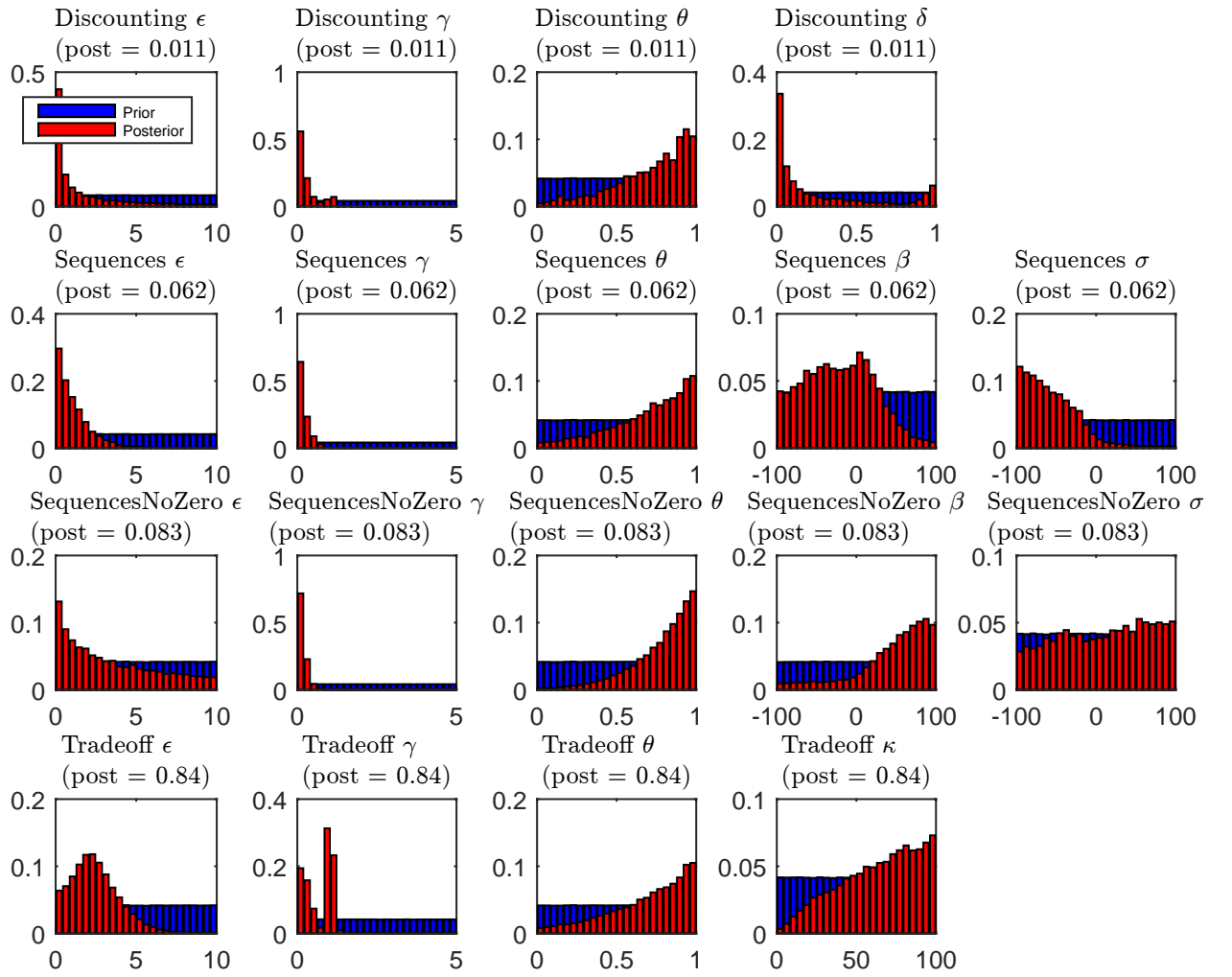

Supplement: Supplementary file 1 [file Scholten_Individuals.zip › plots/e29_p232_eg2_priors_and_posteriors.pdf]

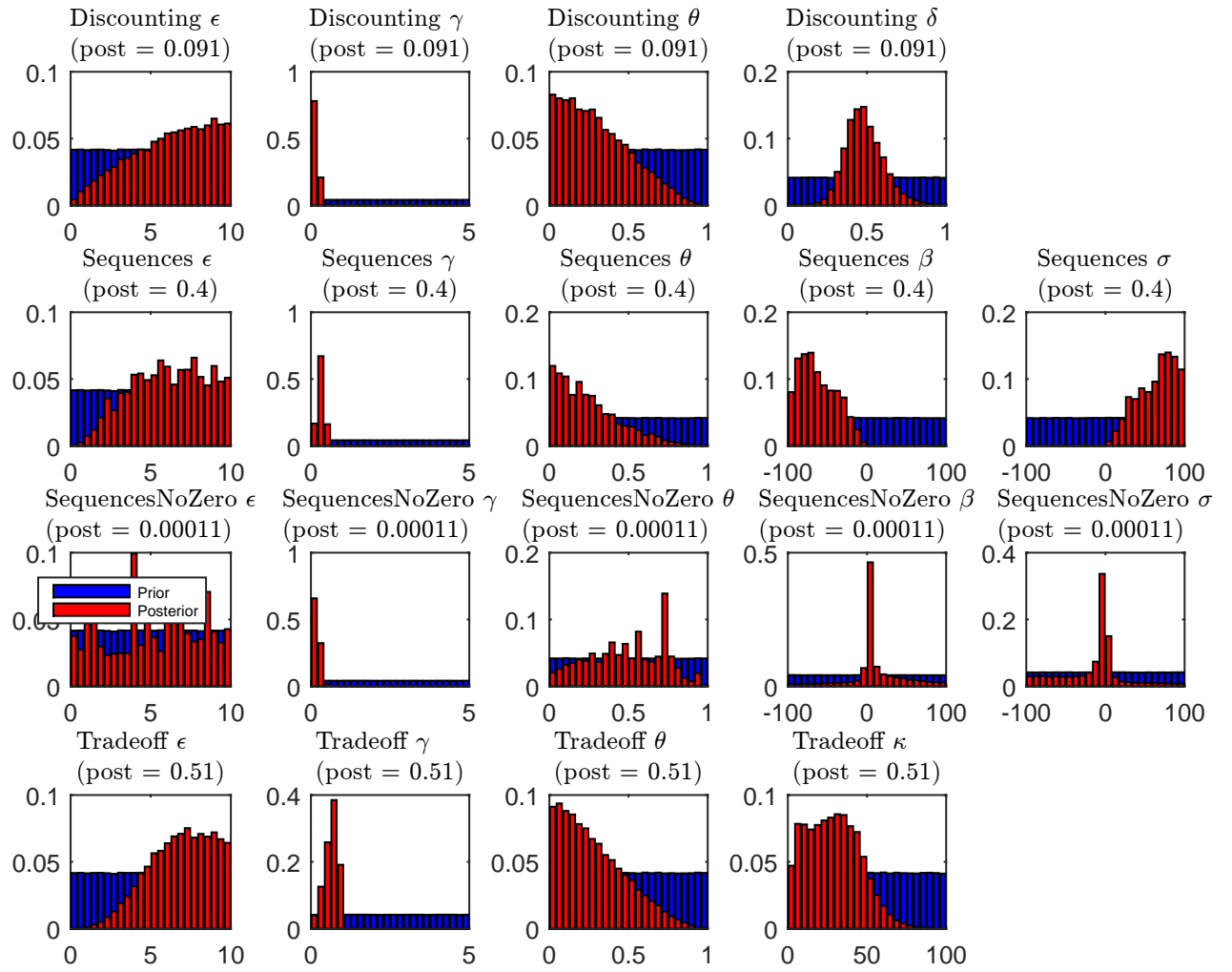

Supplement: Supplementary file 1 [file Scholten_Individuals.zip › plots/e29_p233_eg2_priors_and_posteriors.pdf]

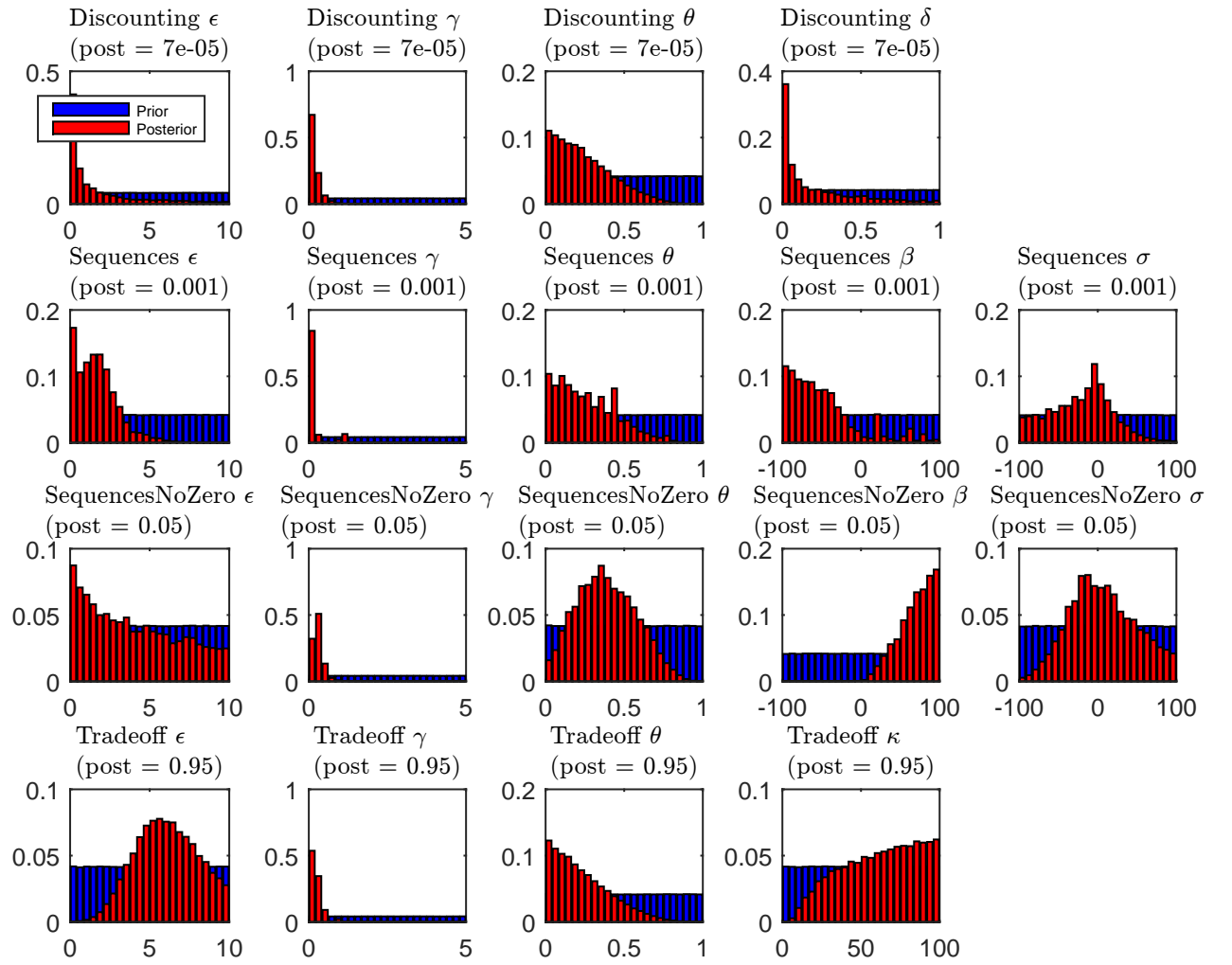

Supplement: Supplementary file 1 [file Scholten_Individuals.zip › plots/e29_p234_eg2_priors_and_posteriors.pdf]

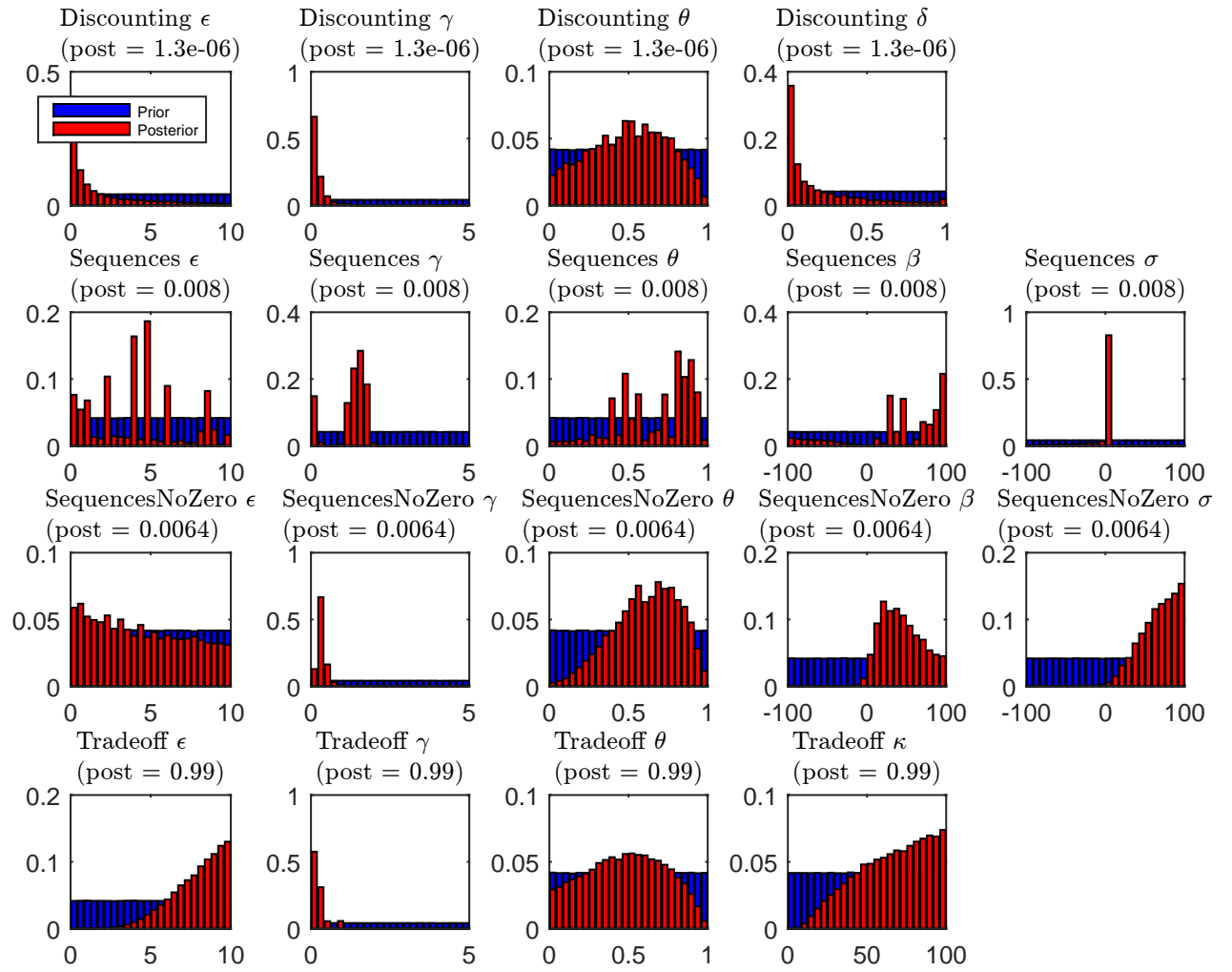

Supplement: Supplementary file 1 [file Scholten_Individuals.zip › plots/e29_p235_eg2_priors_and_posteriors.pdf]

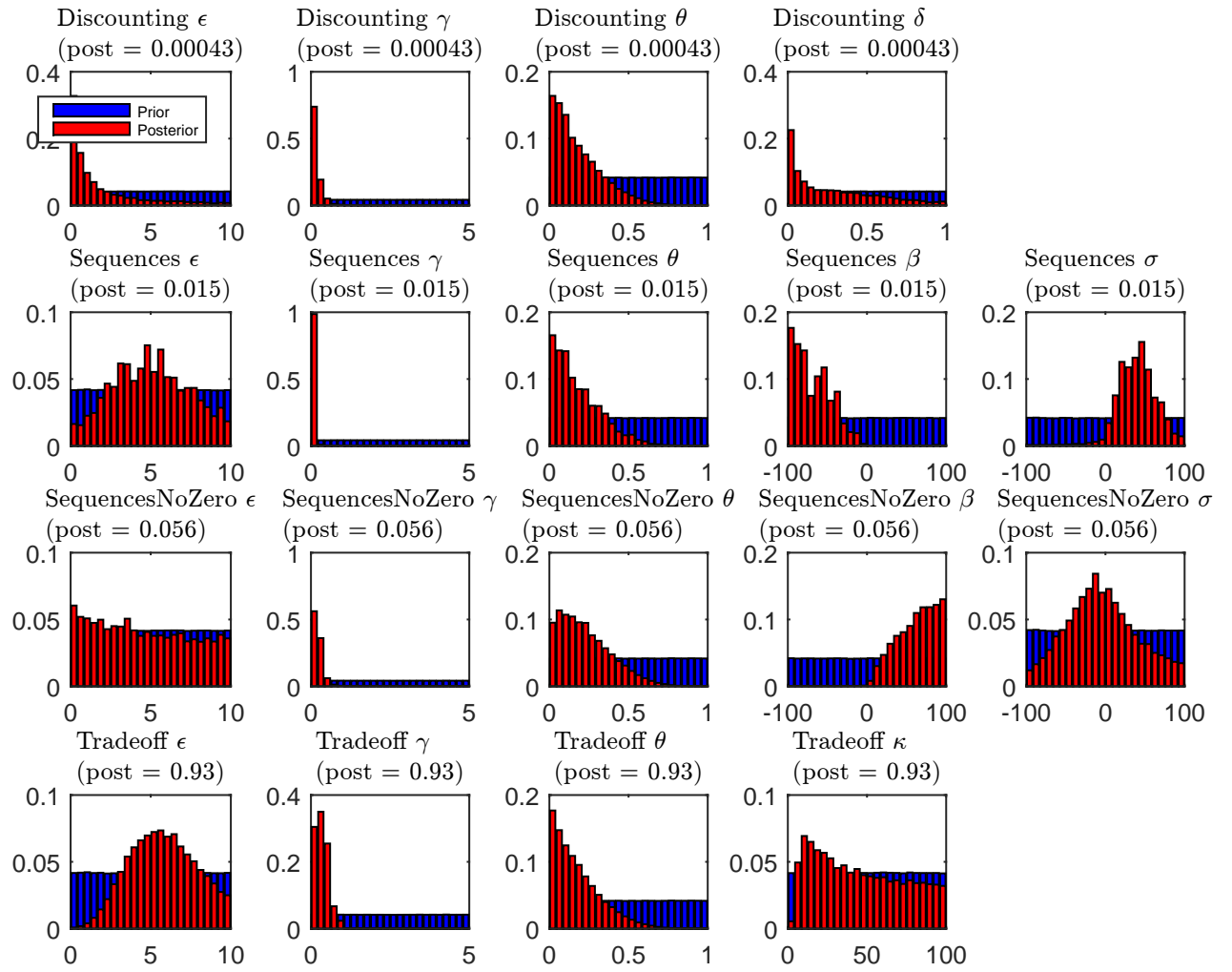

Supplement: Supplementary file 1 [file Scholten_Individuals.zip › plots/e29_p236_eg2_priors_and_posteriors.pdf]

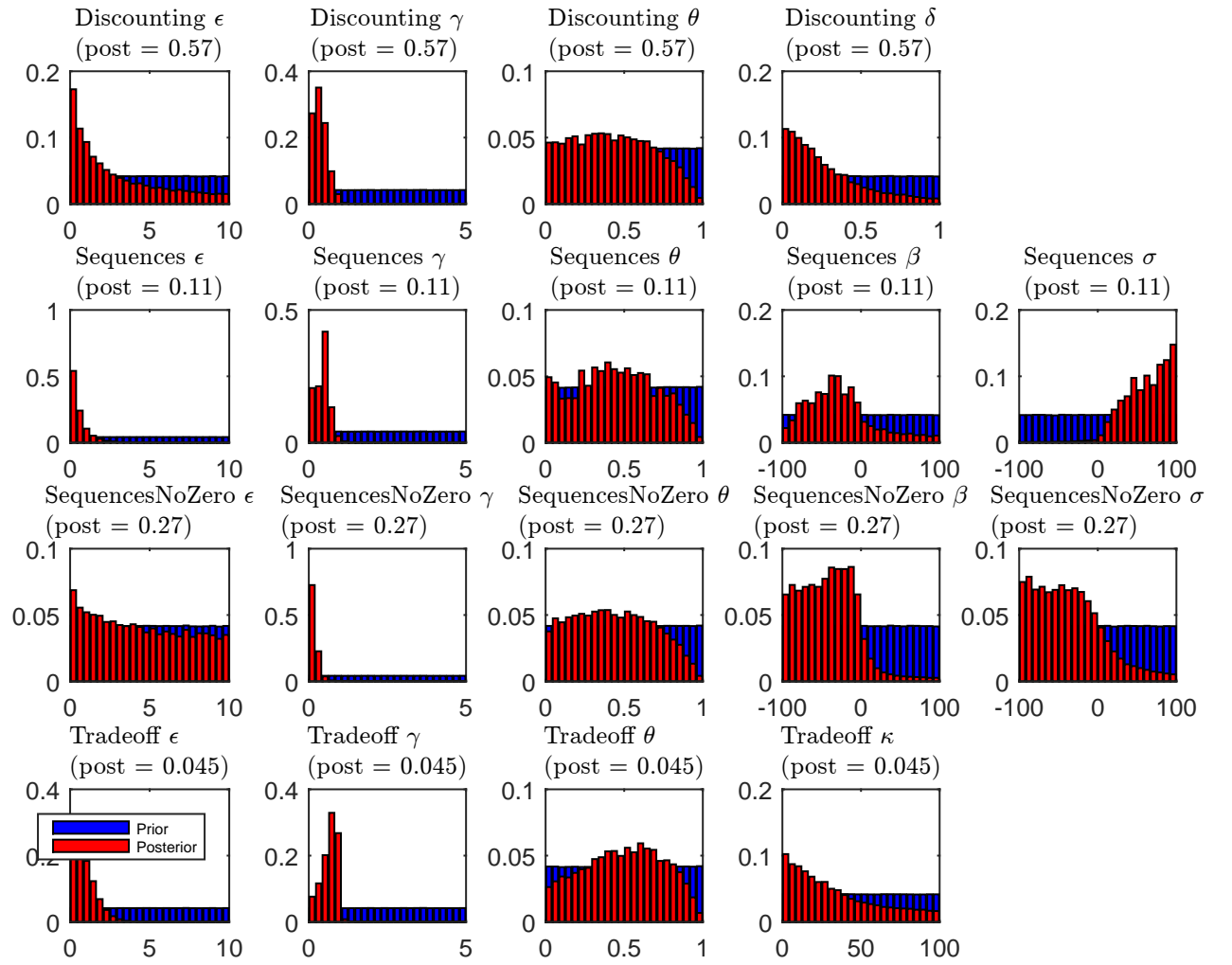

Supplement: Supplementary file 1 [file Scholten_Individuals.zip › plots/e29_p237_eg2_priors_and_posteriors.pdf]

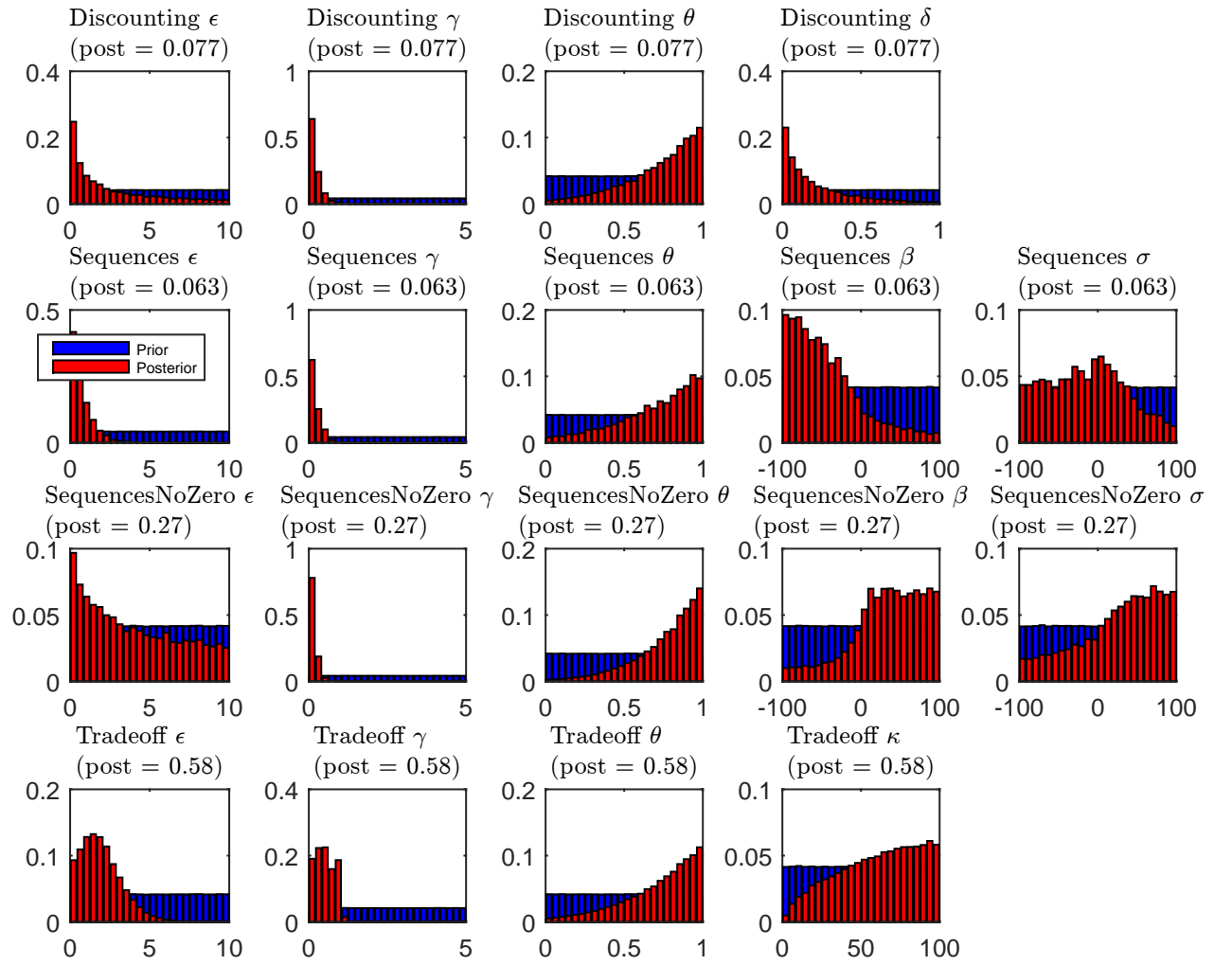

Supplement: Supplementary file 1 [file Scholten_Individuals.zip › plots/e29_p238_eg2_priors_and_posteriors.pdf]

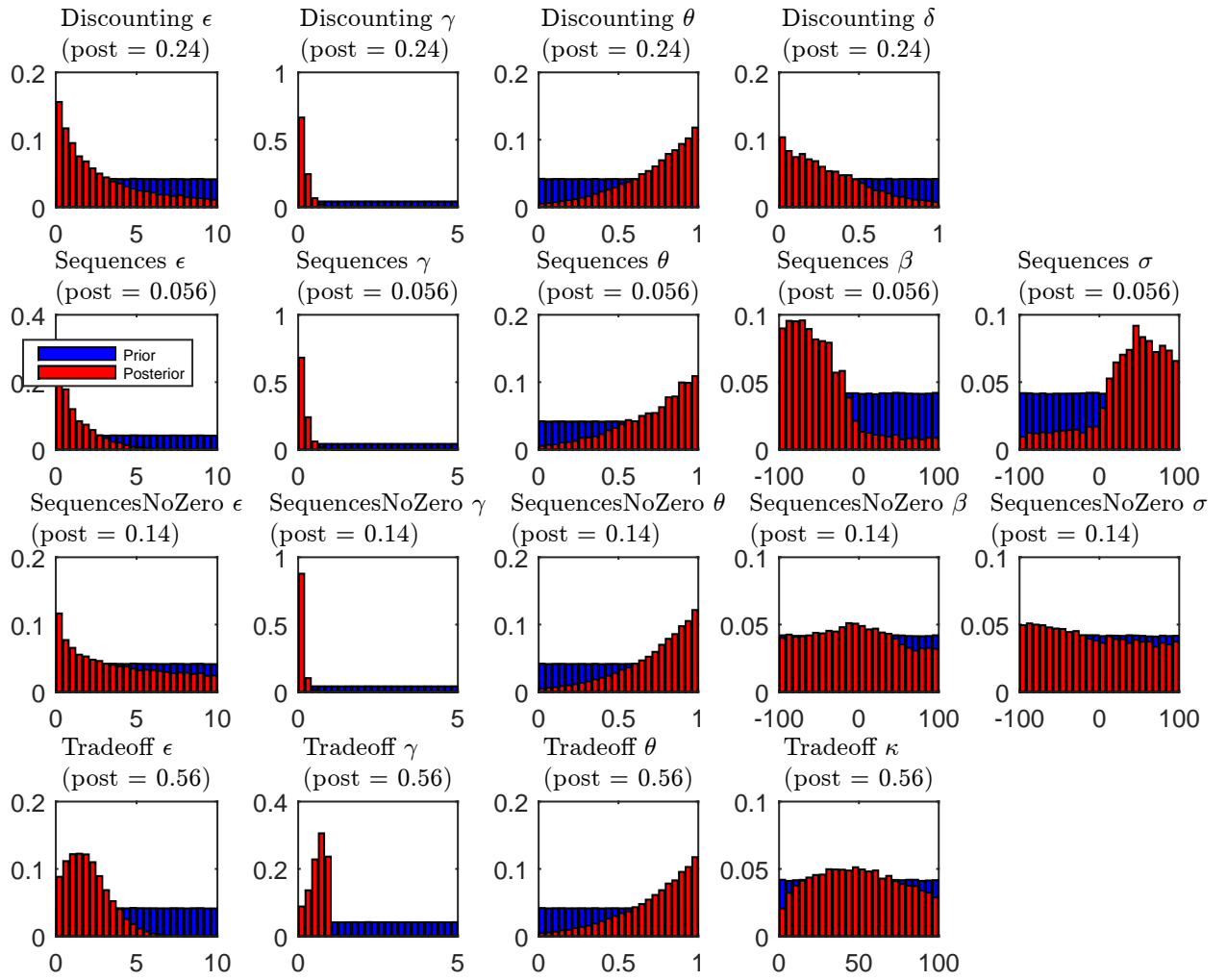

Supplement: Supplementary file 1 [file Scholten_Individuals.zip › plots/e29_p239_eg2_priors_and_posteriors.pdf]

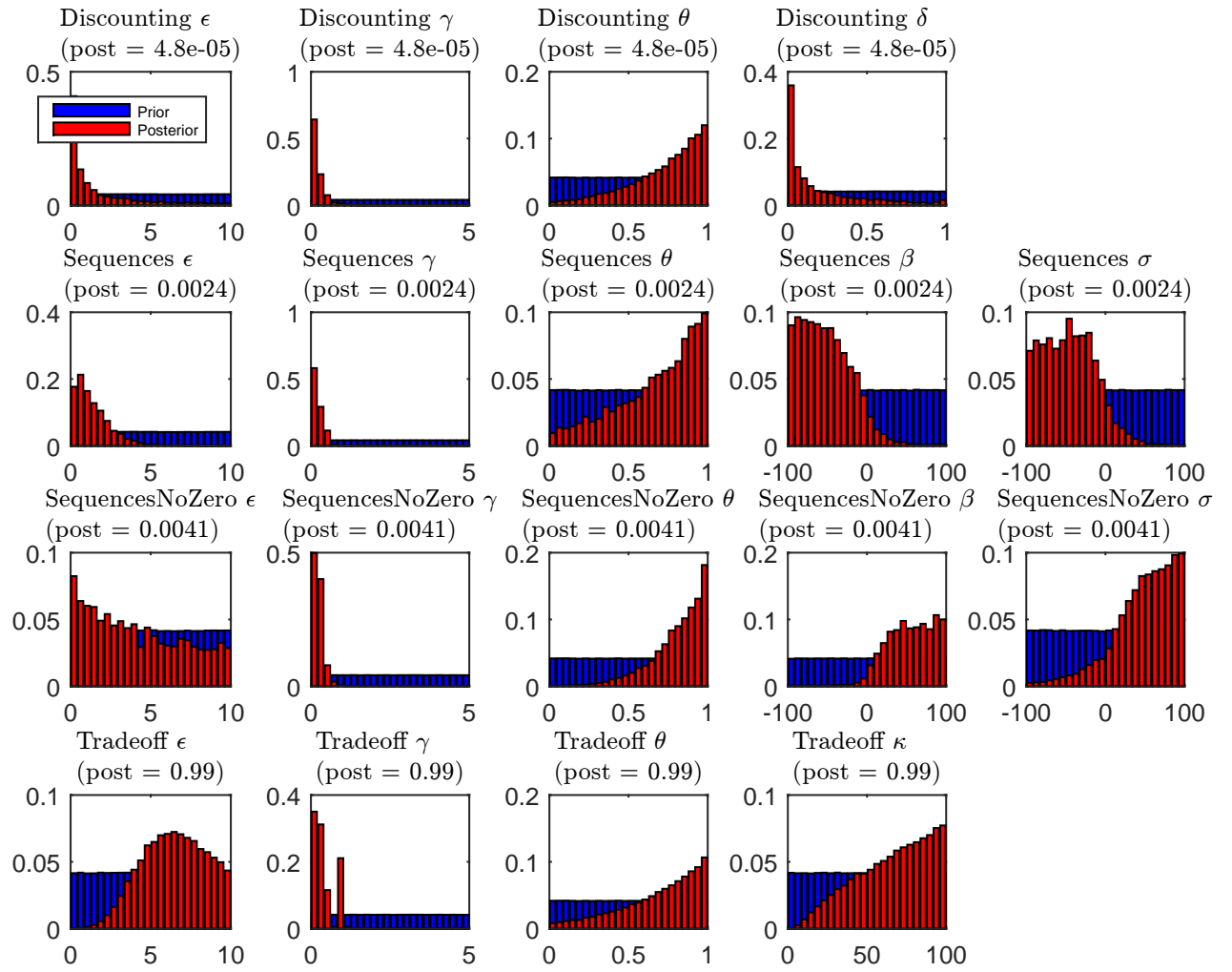

Supplement: Supplementary file 1 [file Scholten_Individuals.zip › plots/e29_p24_eg2_priors_and_posteriors.pdf]

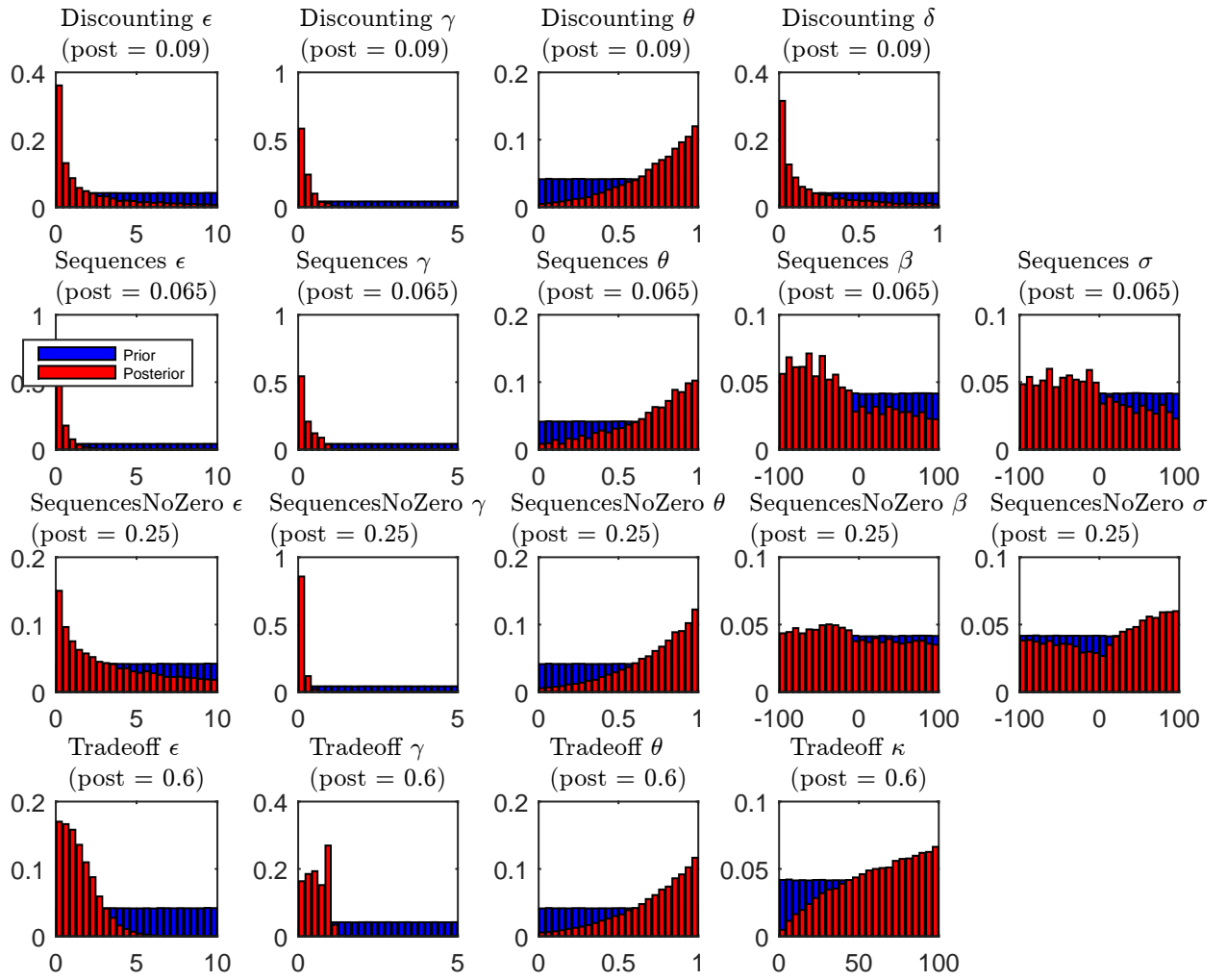

Supplement: Supplementary file 1 [file Scholten_Individuals.zip › plots/e29_p240_eg2_priors_and_posteriors.pdf]

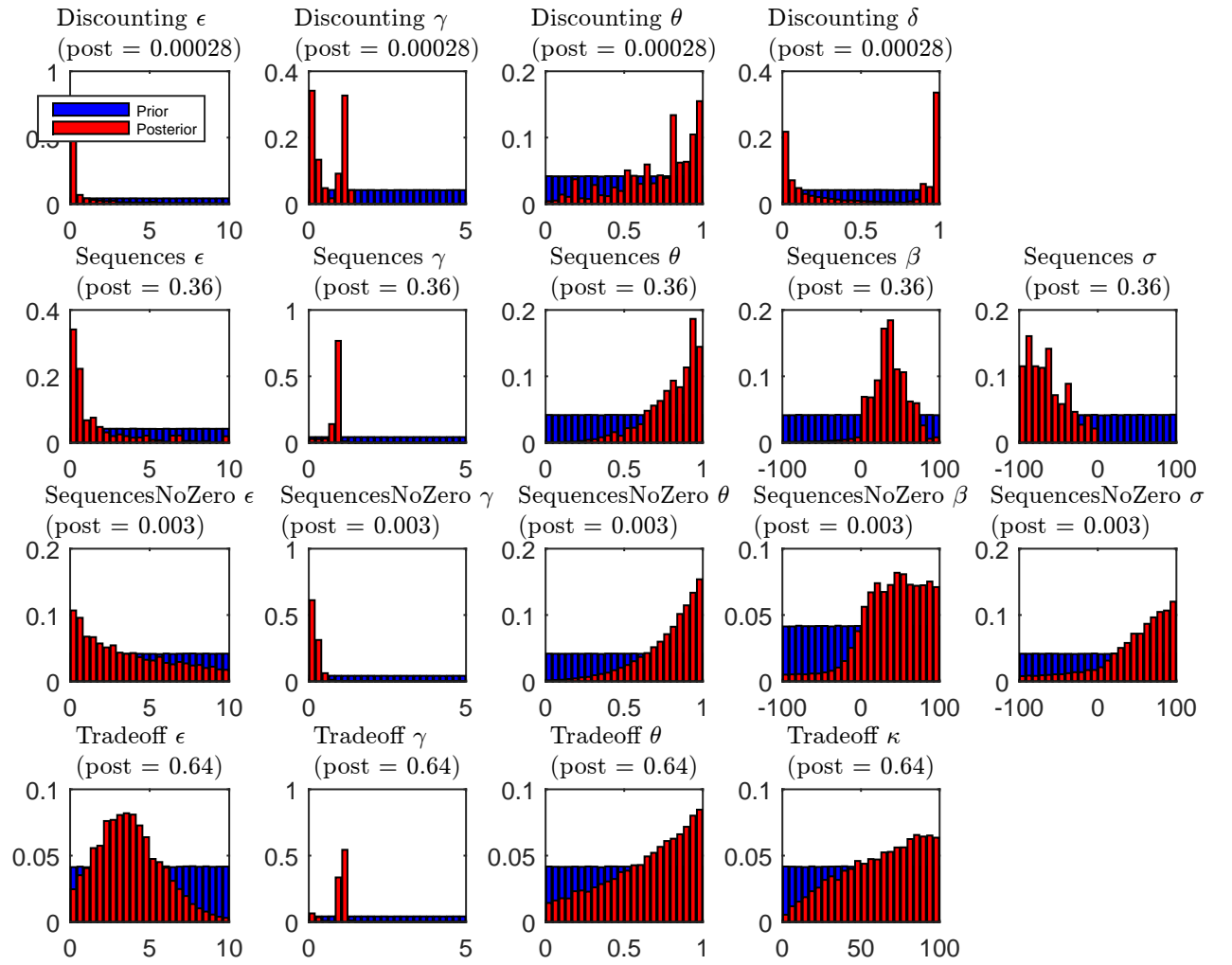

Supplement: Supplementary file 1 [file Scholten_Individuals.zip › plots/e29_p241_eg2_priors_and_posteriors.pdf]

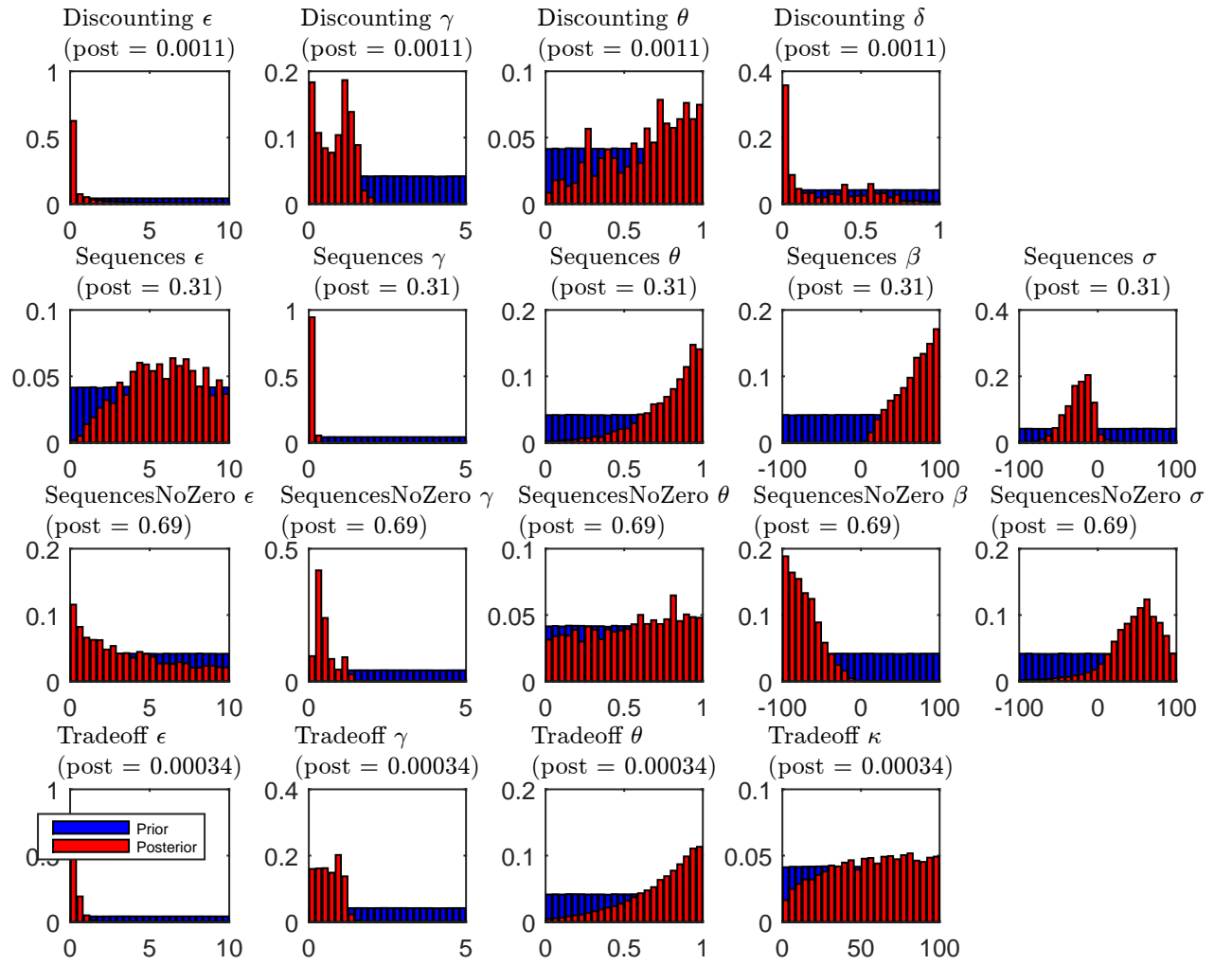

Supplement: Supplementary file 1 [file Scholten_Individuals.zip › plots/e29_p242_eg2_priors_and_posteriors.pdf]

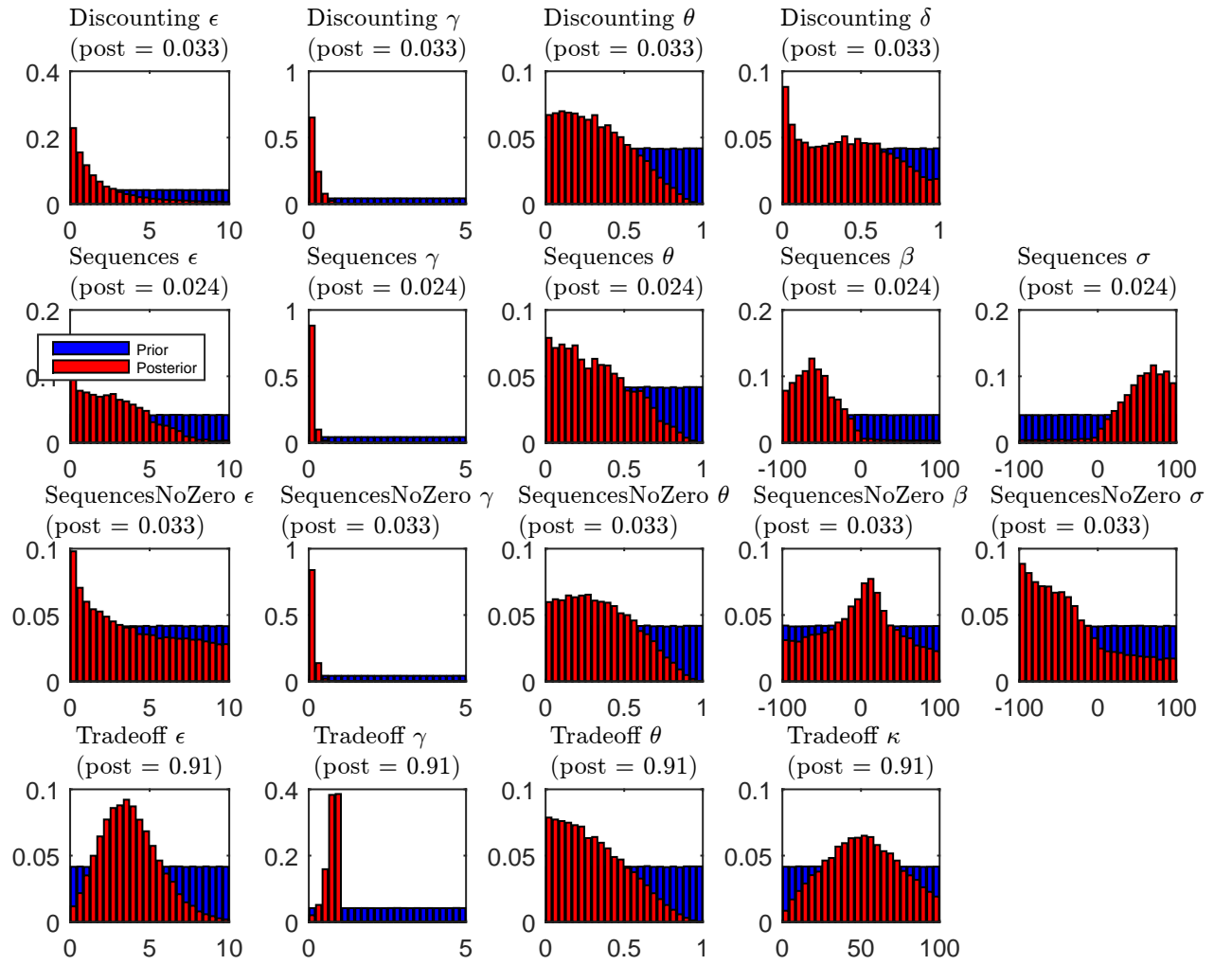

Supplement: Supplementary file 1 [file Scholten_Individuals.zip › plots/e29_p243_eg2_priors_and_posteriors.pdf]

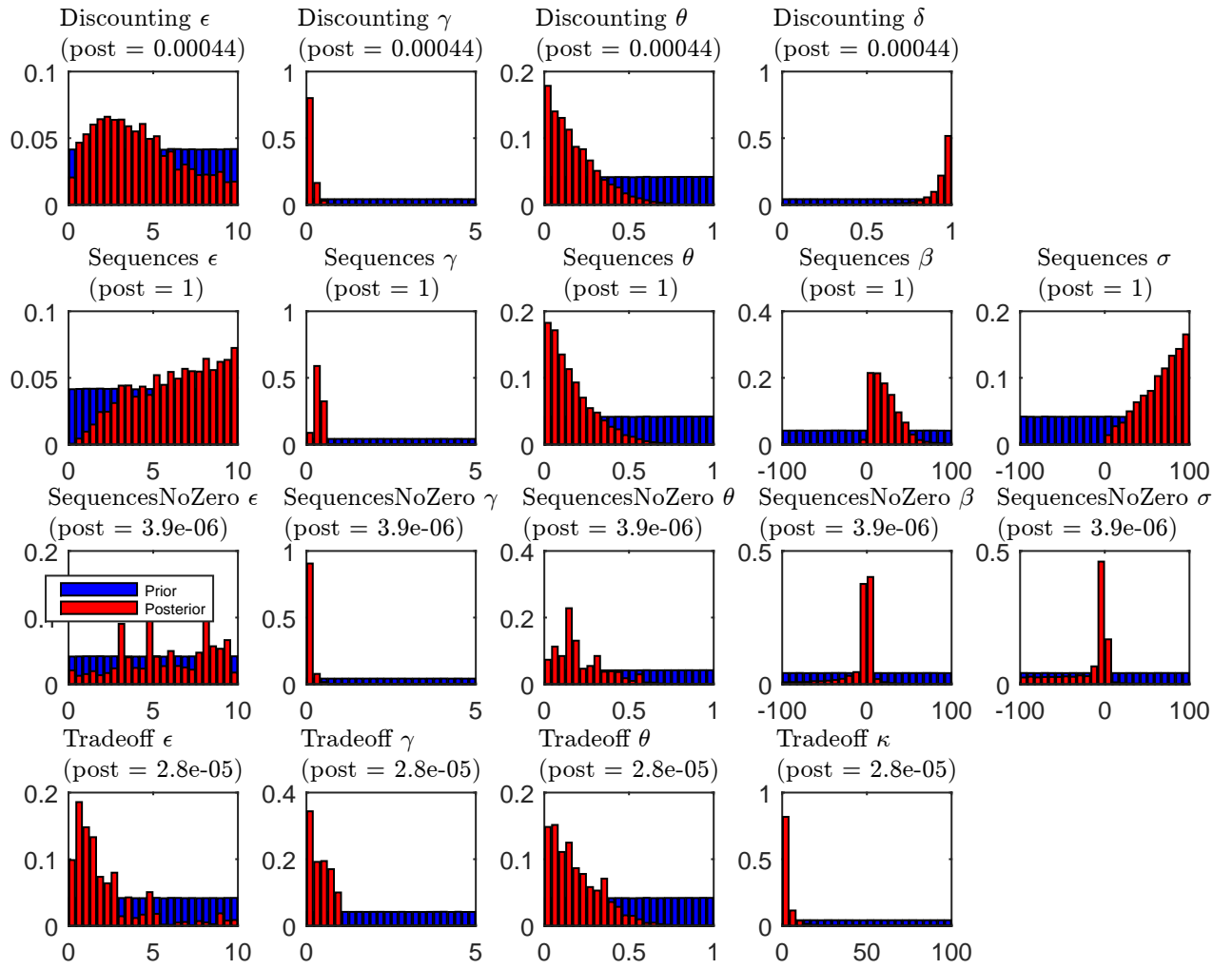

Supplement: Supplementary file 1 [file Scholten_Individuals.zip › plots/e29_p244_eg2_priors_and_posteriors.pdf]

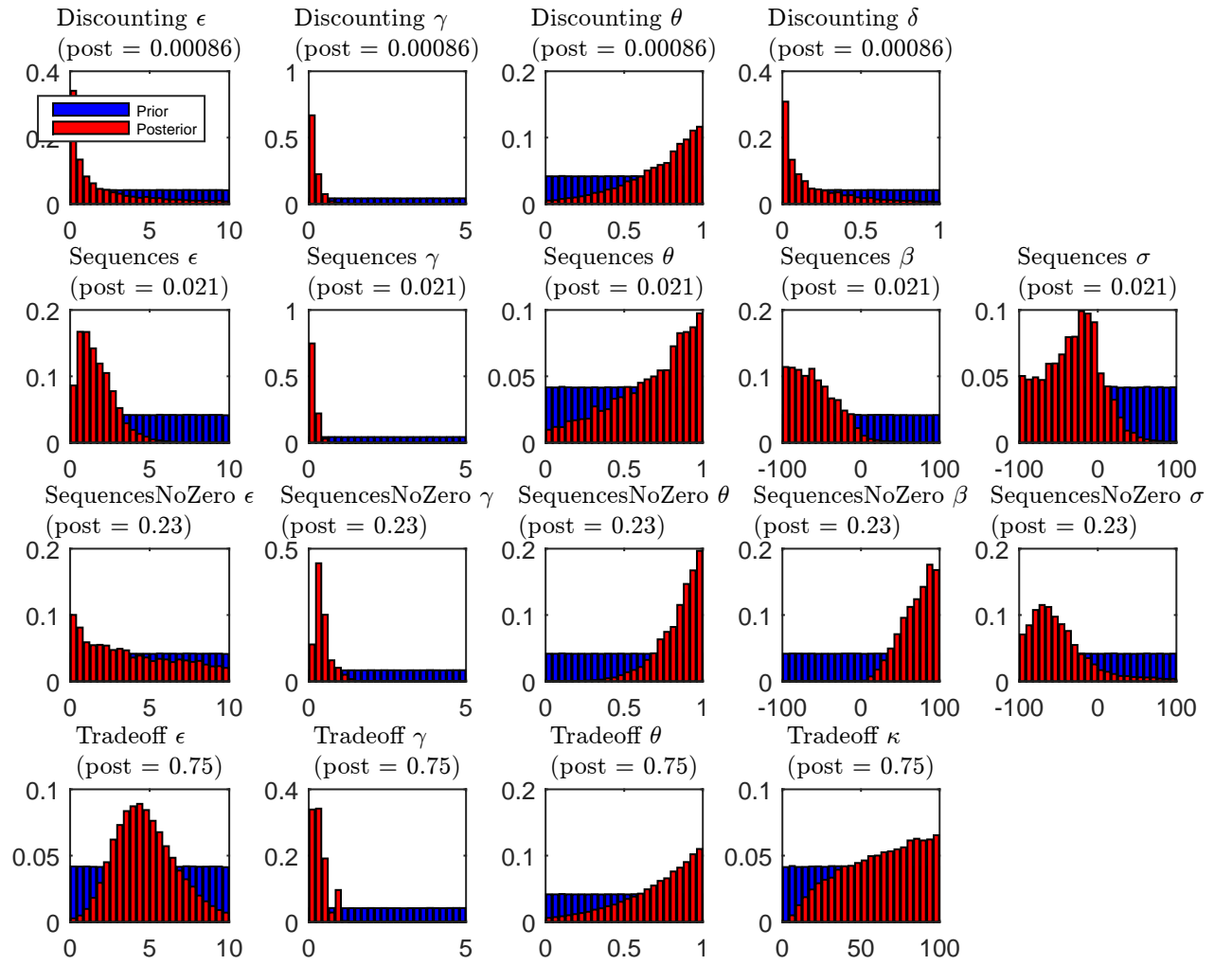

Supplement: Supplementary file 1 [file Scholten_Individuals.zip › plots/e29_p245_eg2_priors_and_posteriors.pdf]

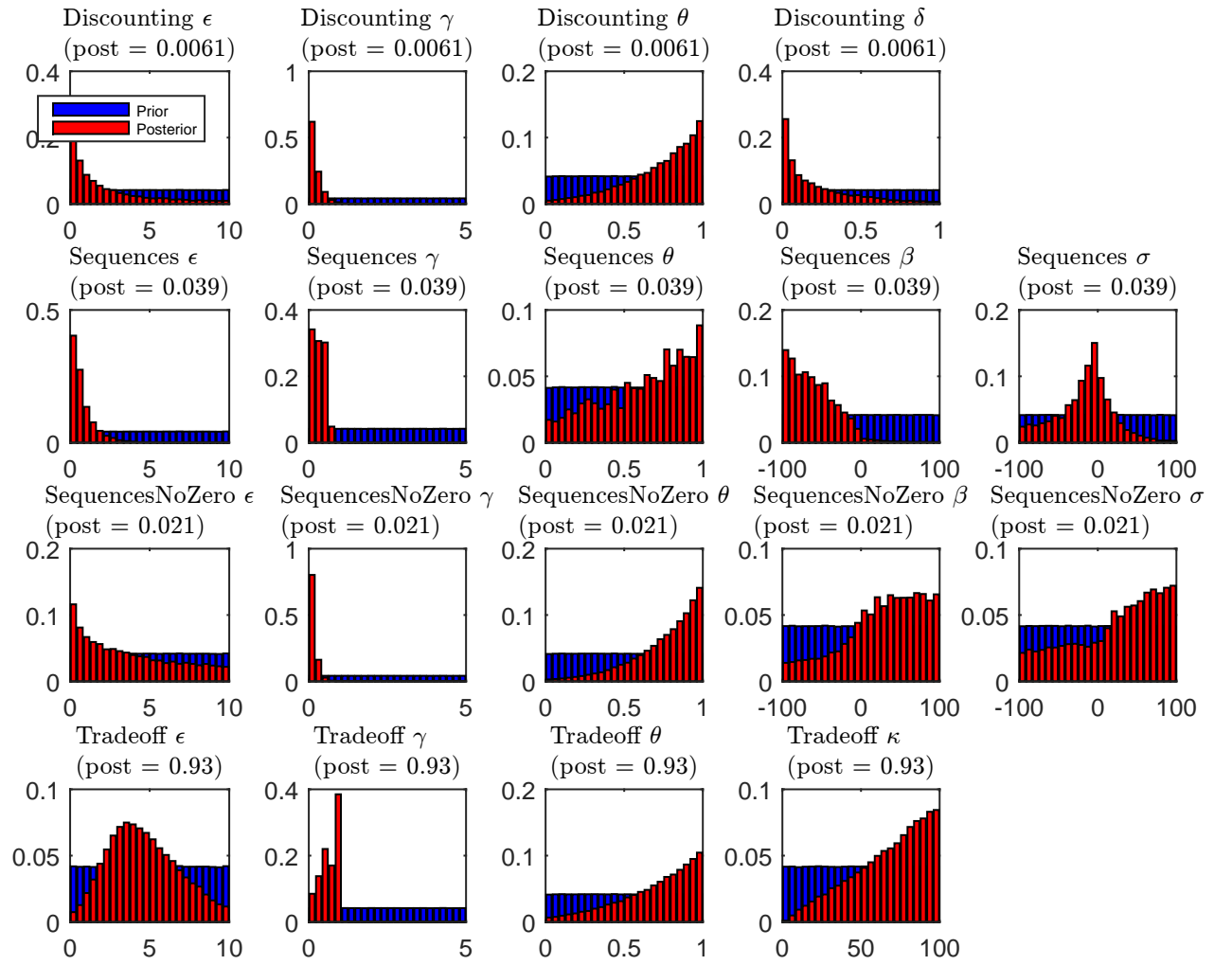

Supplement: Supplementary file 1 [file Scholten_Individuals.zip › plots/e29_p246_eg2_priors_and_posteriors.pdf]

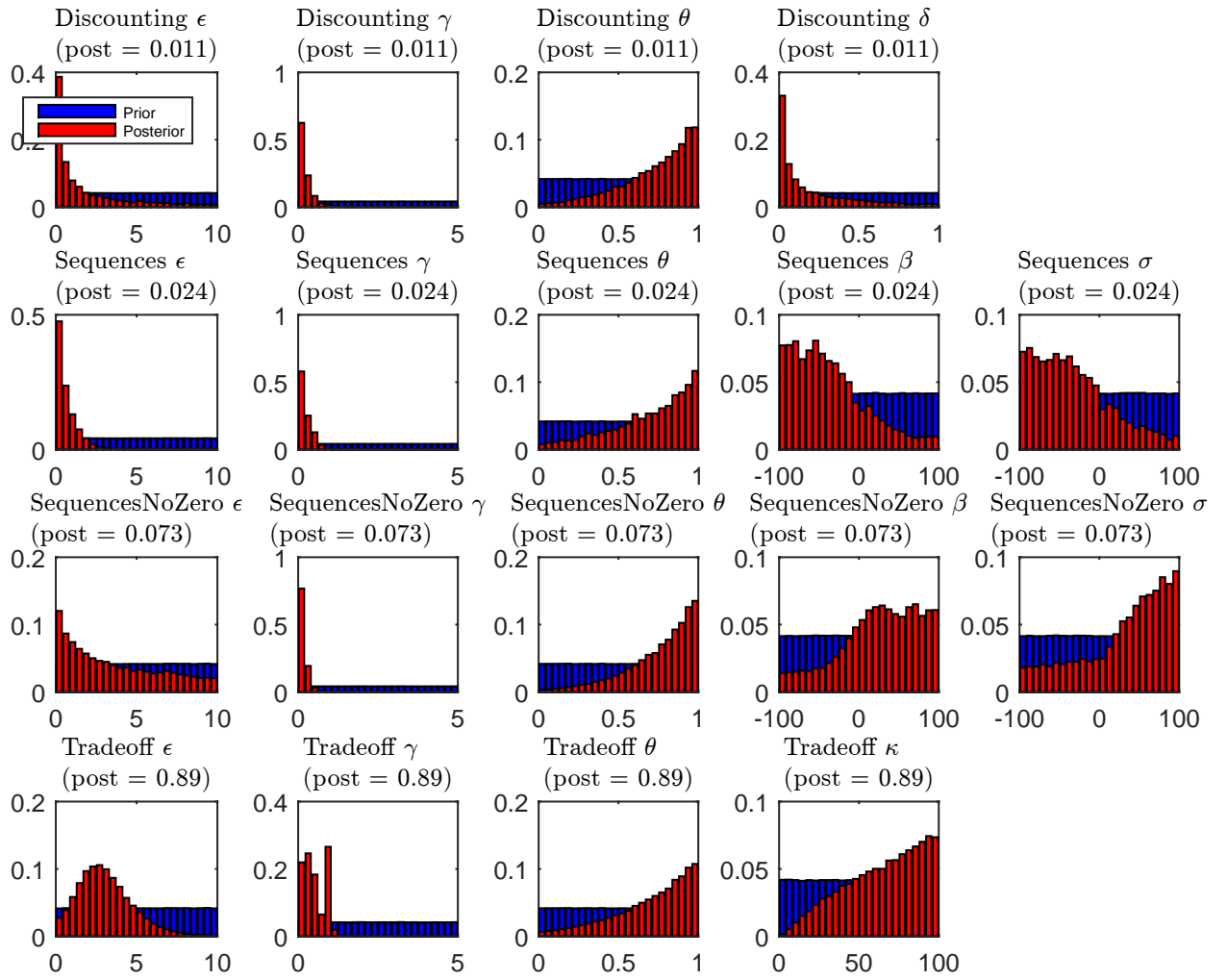

Supplement: Supplementary file 1 [file Scholten_Individuals.zip › plots/e29_p247_eg2_priors_and_posteriors.pdf]

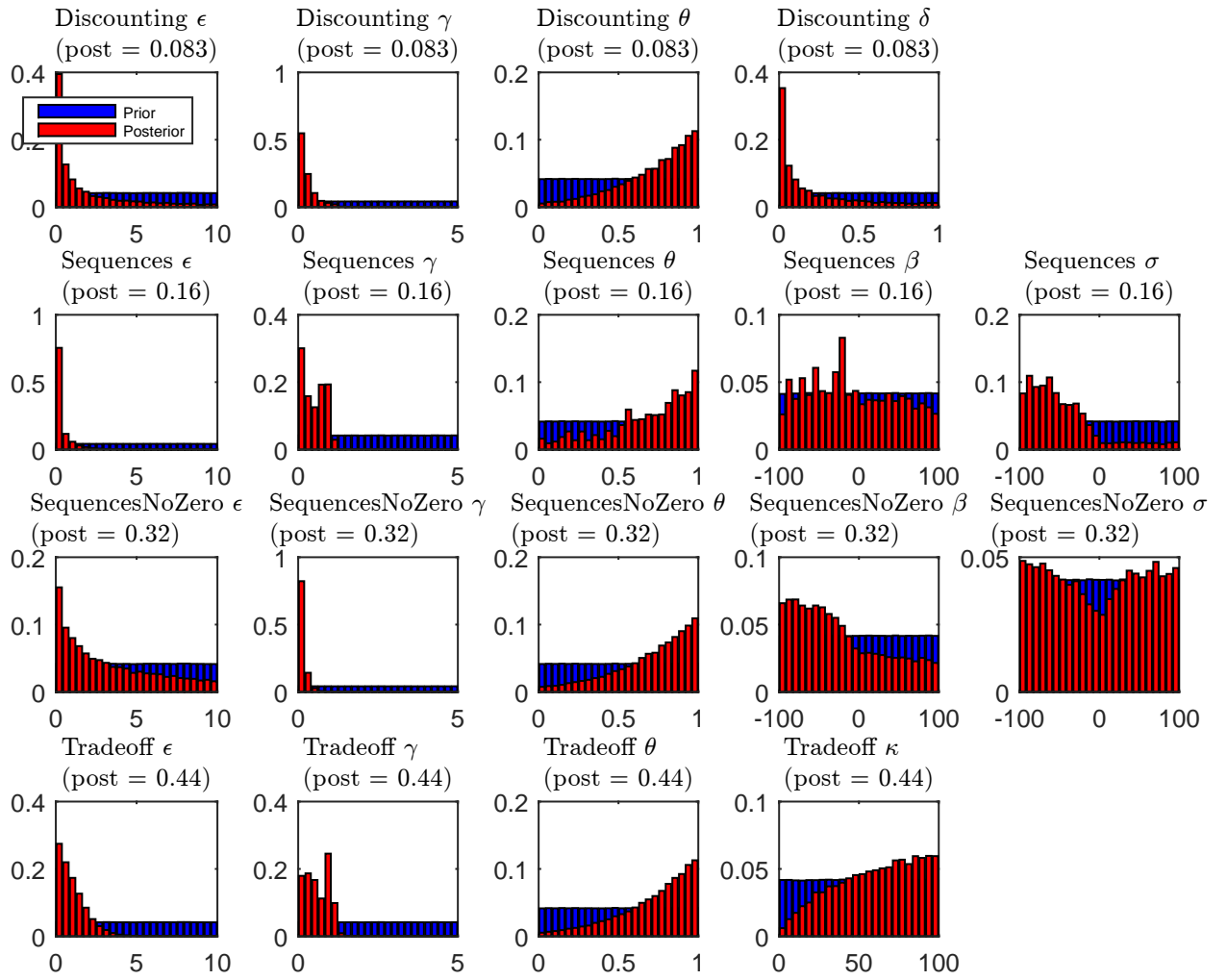

Supplement: Supplementary file 1 [file Scholten_Individuals.zip › plots/e29_p248_eg2_priors_and_posteriors.pdf]

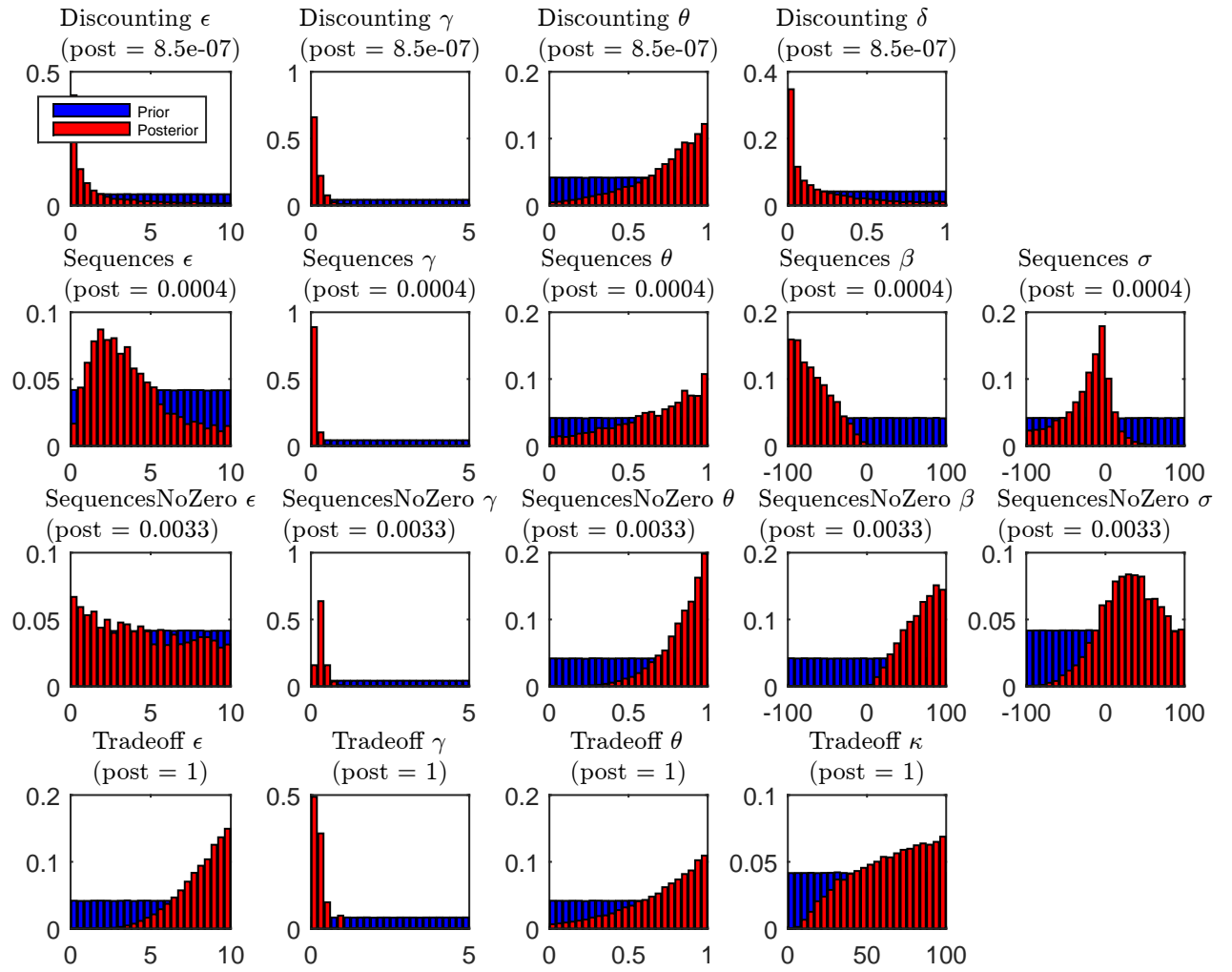

Supplement: Supplementary file 1 [file Scholten_Individuals.zip › plots/e29_p249_eg2_priors_and_posteriors.pdf]

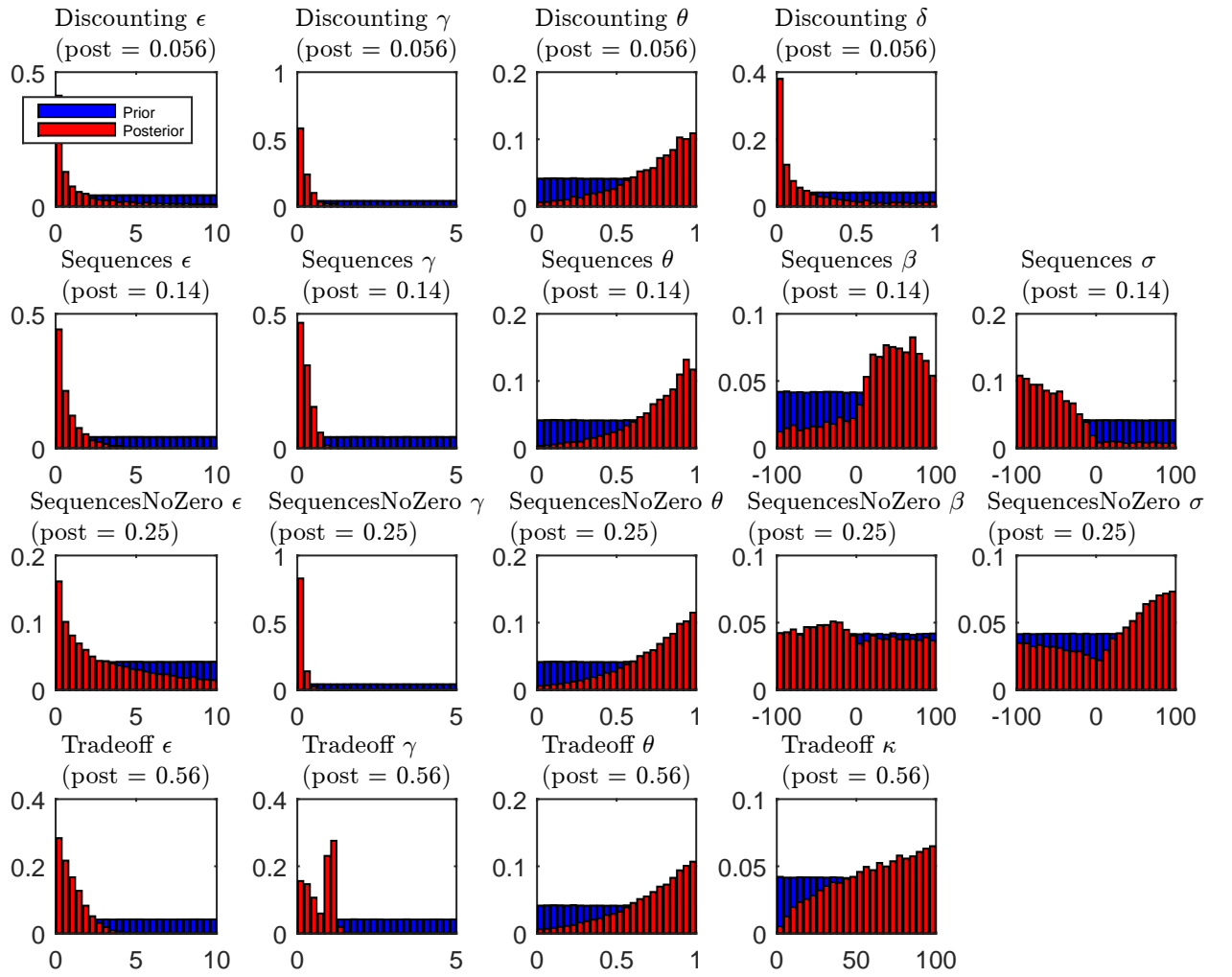

Supplement: Supplementary file 1 [file Scholten_Individuals.zip › plots/e29_p25_eg2_priors_and_posteriors.pdf]

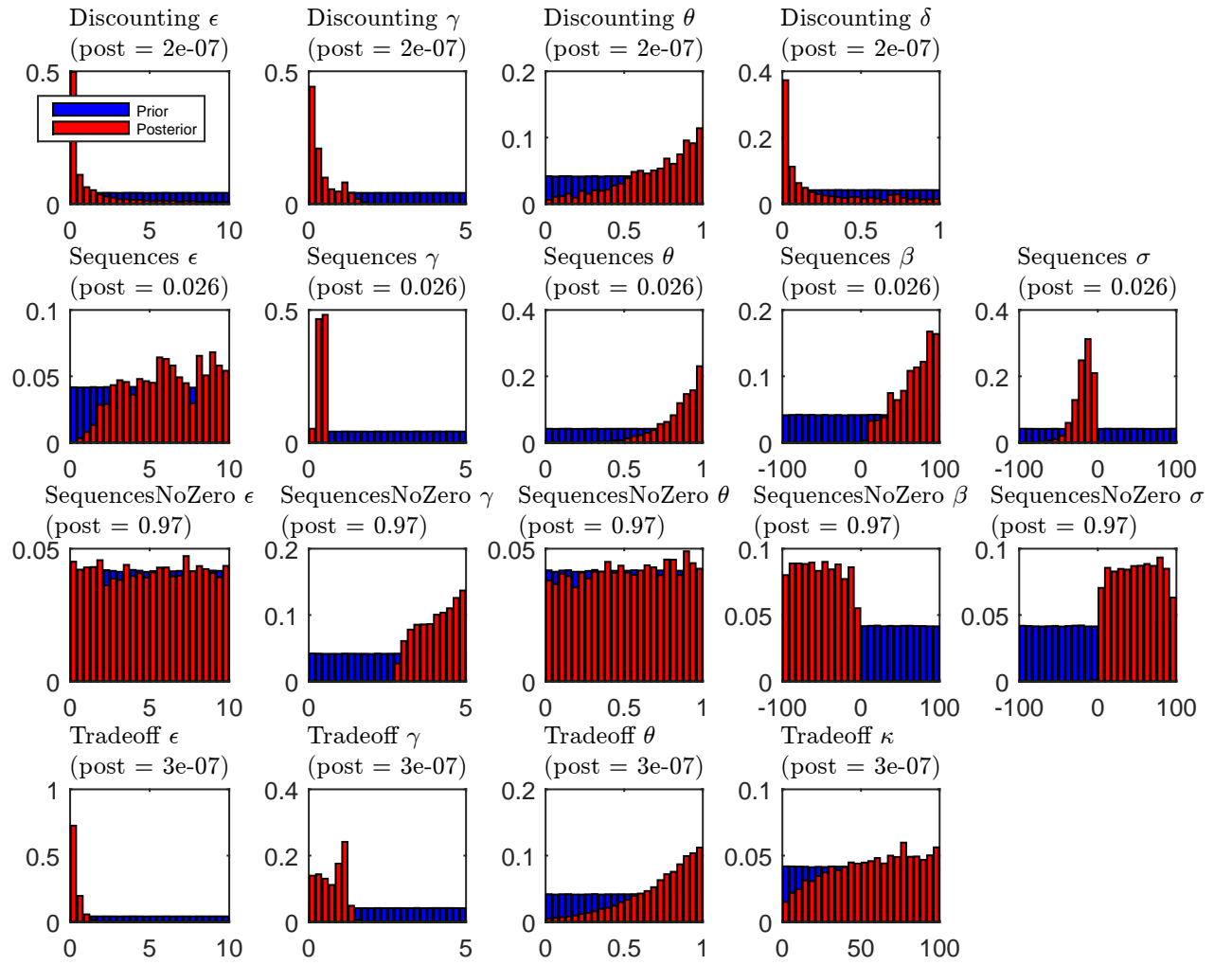

Supplement: Supplementary file 1 [file Scholten_Individuals.zip › plots/e29_p250_eg2_priors_and_posteriors.pdf]

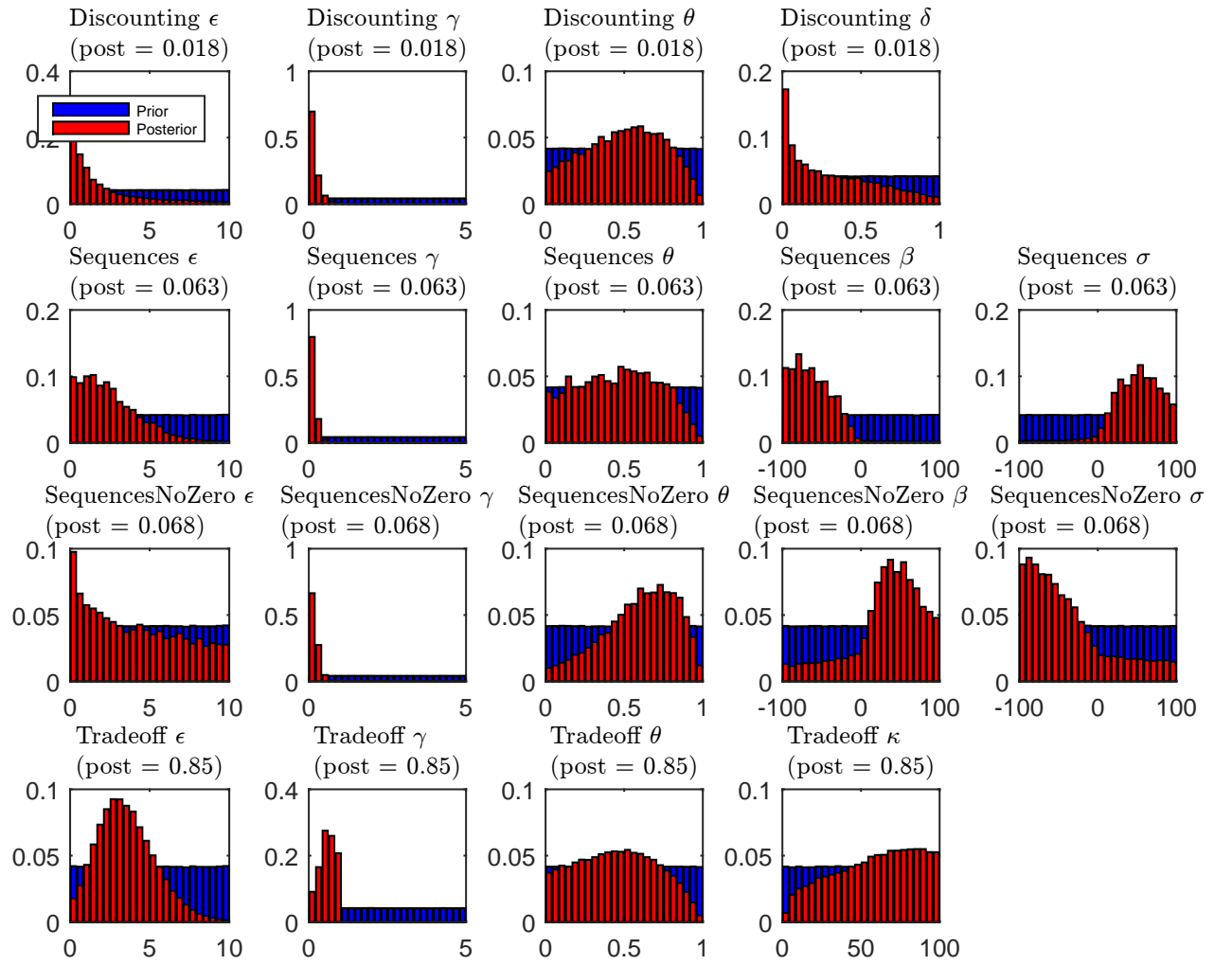

Supplement: Supplementary file 1 [file Scholten_Individuals.zip › plots/e29_p251_eg2_priors_and_posteriors.pdf]

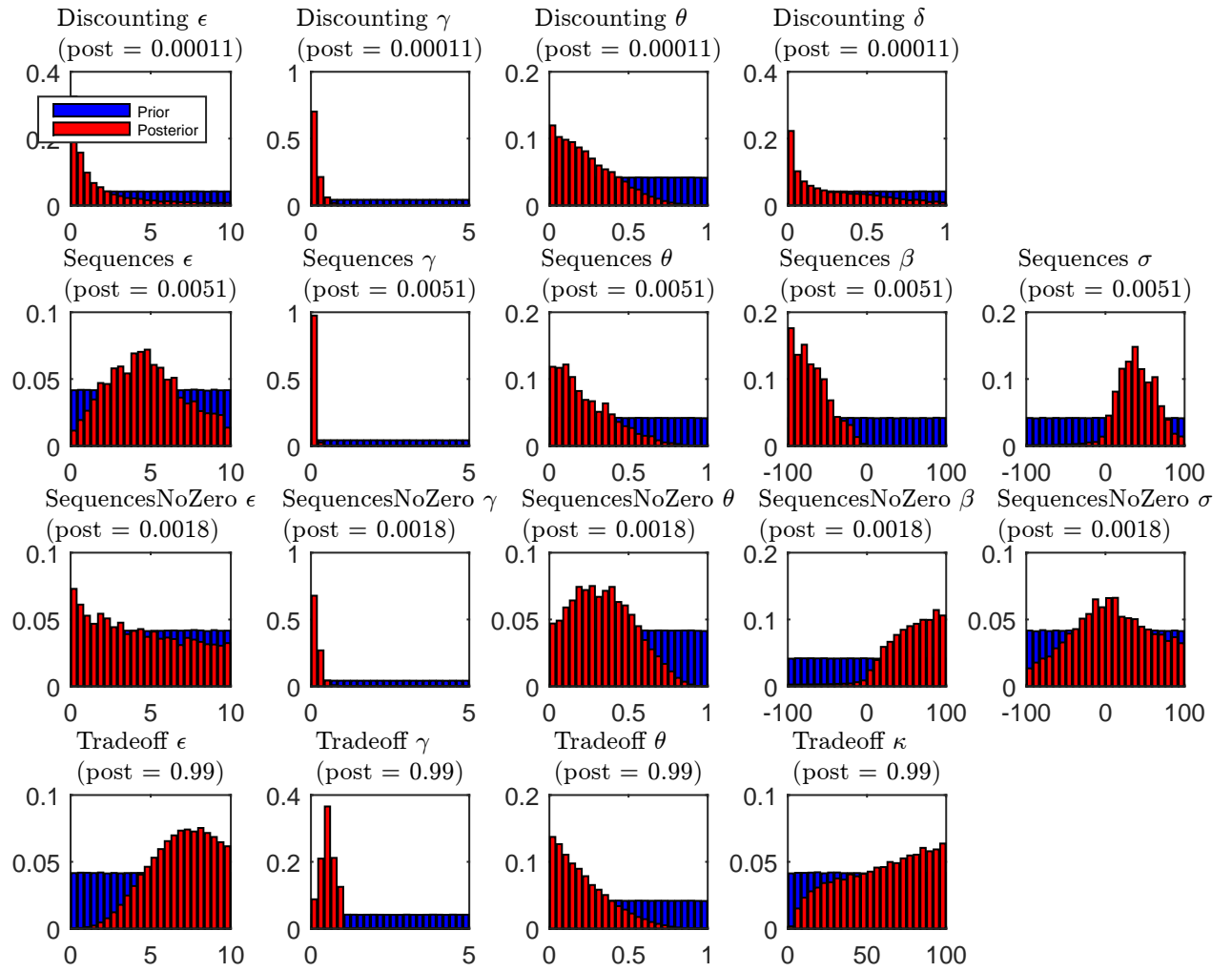

Supplement: Supplementary file 1 [file Scholten_Individuals.zip › plots/e29_p252_eg2_priors_and_posteriors.pdf]

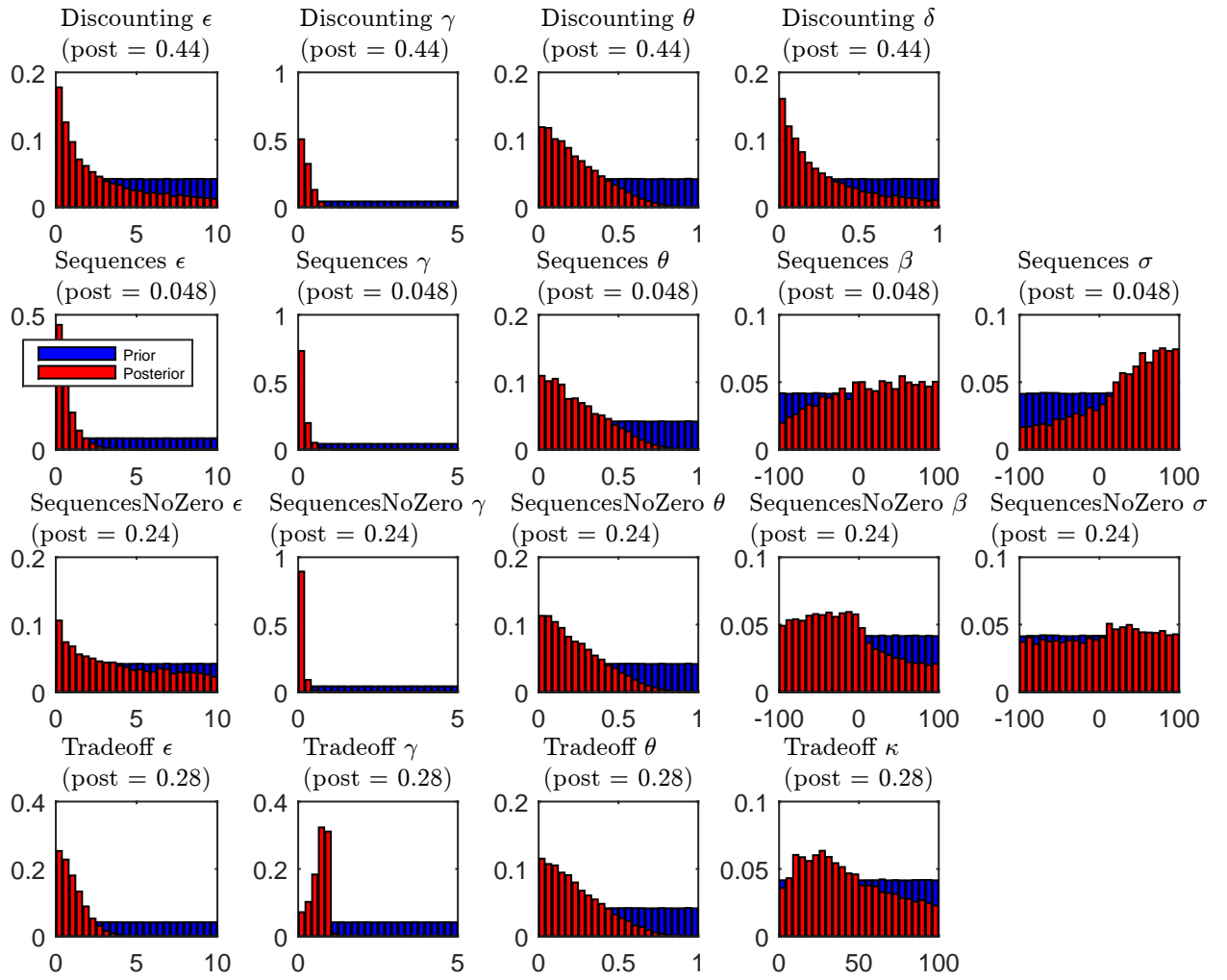

Supplement: Supplementary file 1 [file Scholten_Individuals.zip › plots/e29_p253_eg2_priors_and_posteriors.pdf]

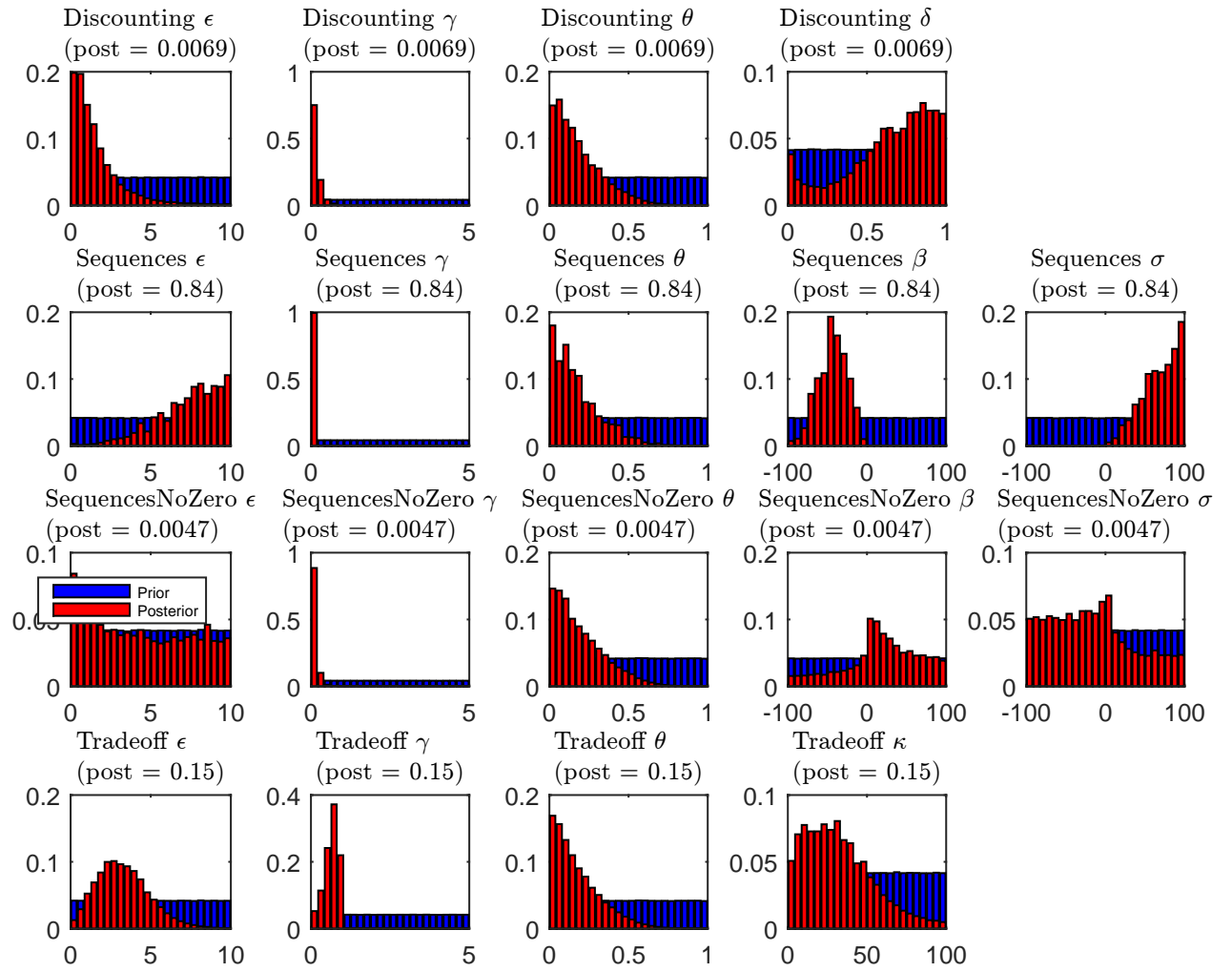

Supplement: Supplementary file 1 [file Scholten_Individuals.zip › plots/e29_p254_eg2_priors_and_posteriors.pdf]

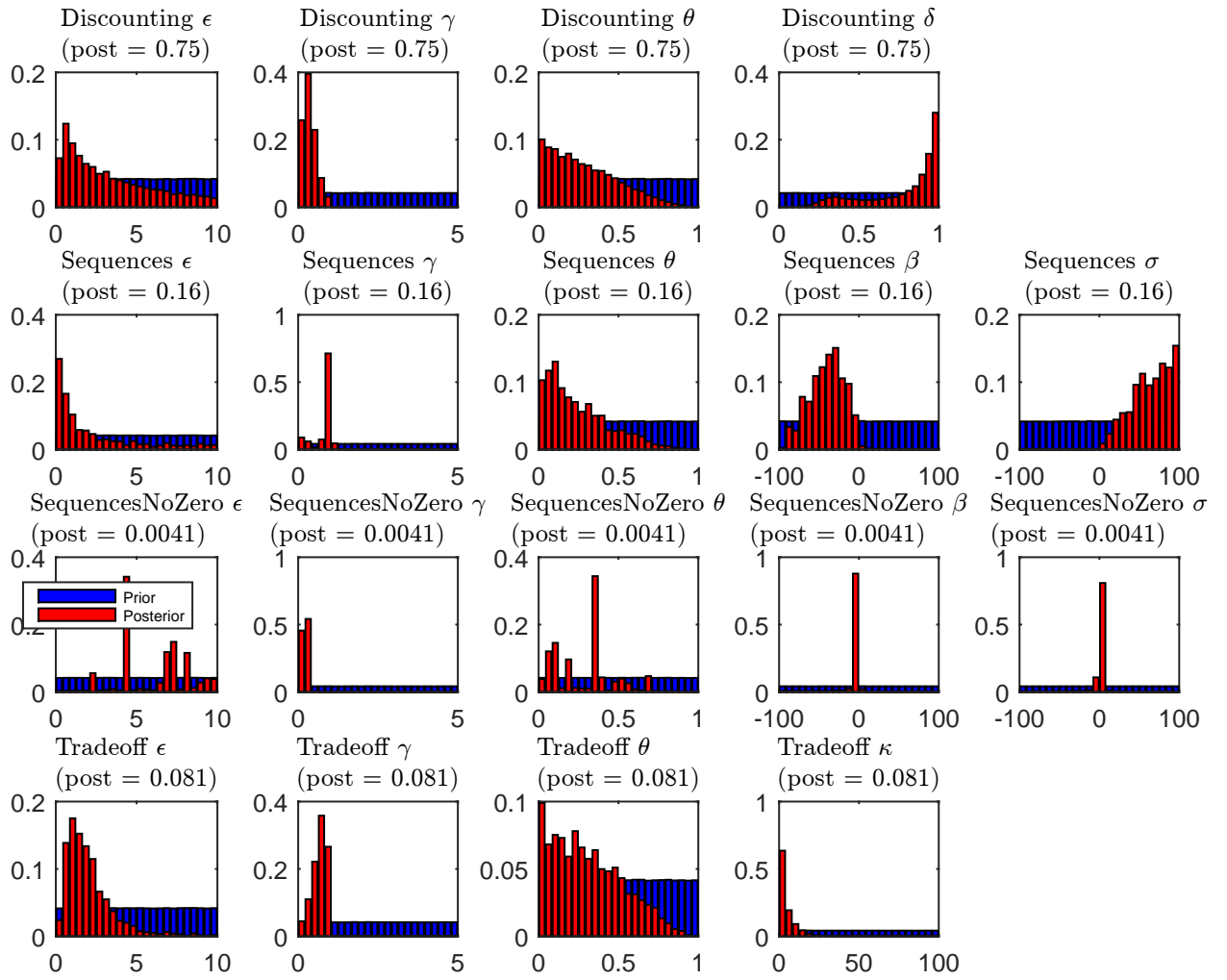

Supplement: Supplementary file 1 [file Scholten_Individuals.zip › plots/e29_p255_eg2_priors_and_posteriors.pdf]

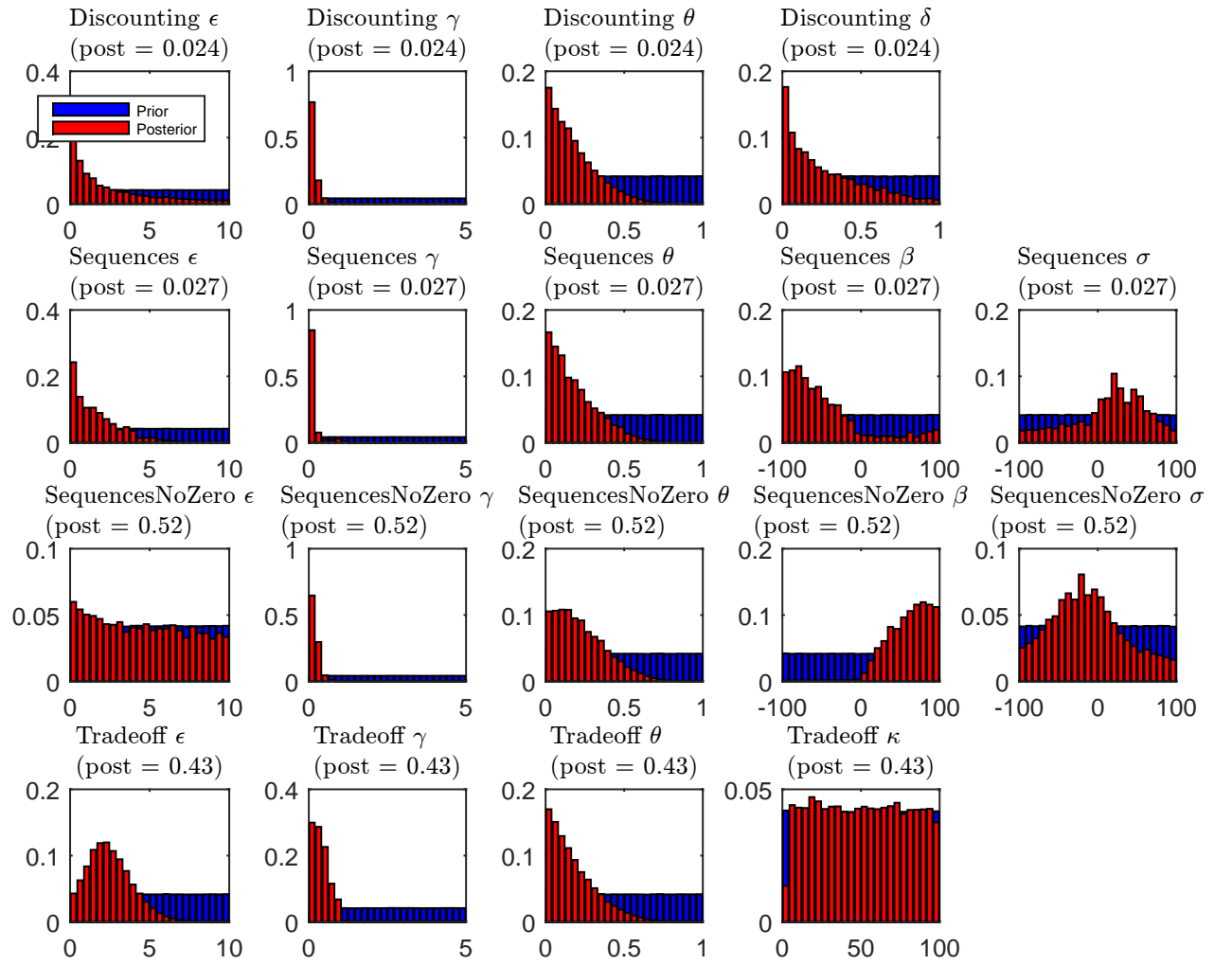

Supplement: Supplementary file 1 [file Scholten_Individuals.zip › plots/e29_p256_eg2_priors_and_posteriors.pdf]

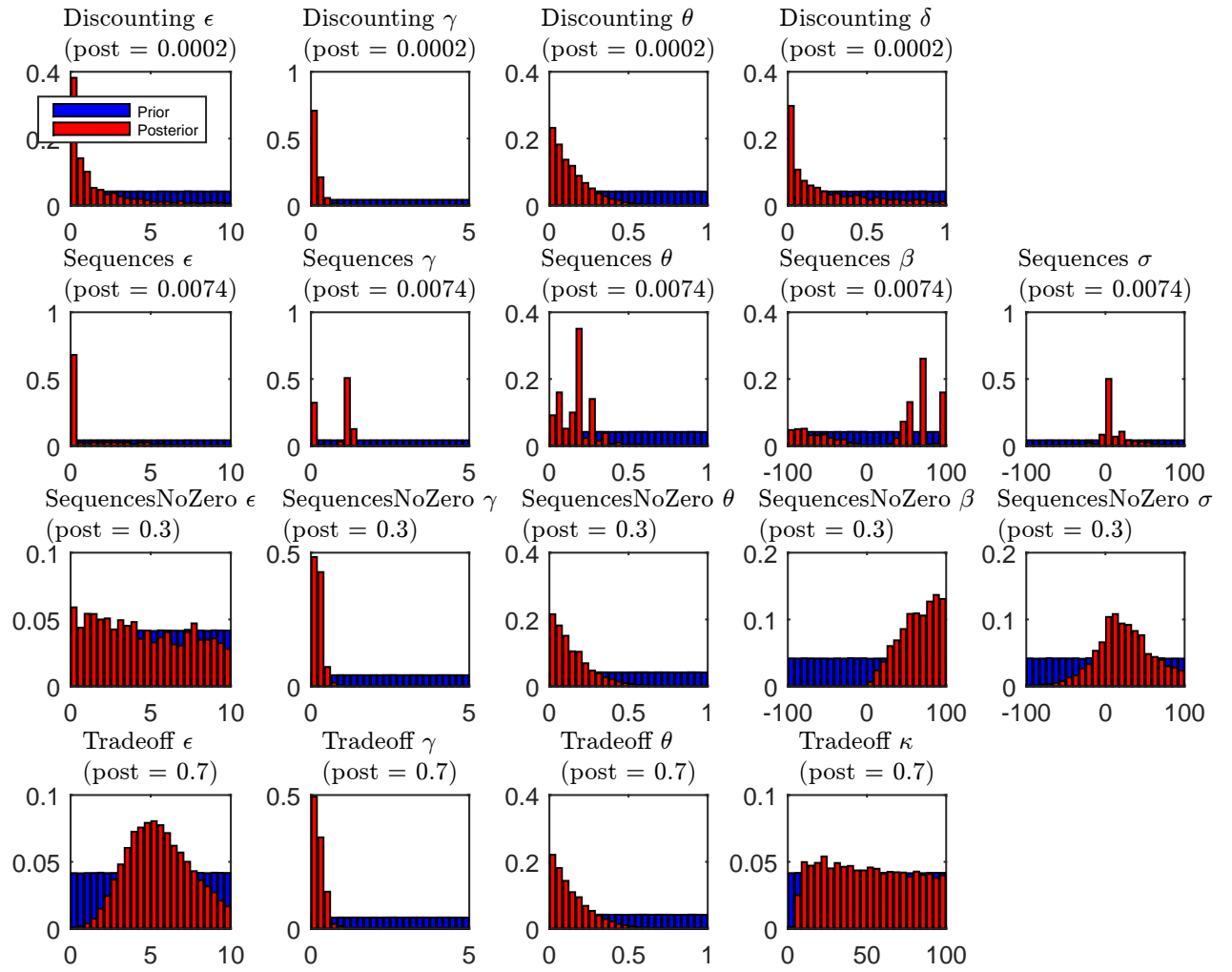

Supplement: Supplementary file 1 [file Scholten_Individuals.zip › plots/e29_p257_eg2_priors_and_posteriors.pdf]

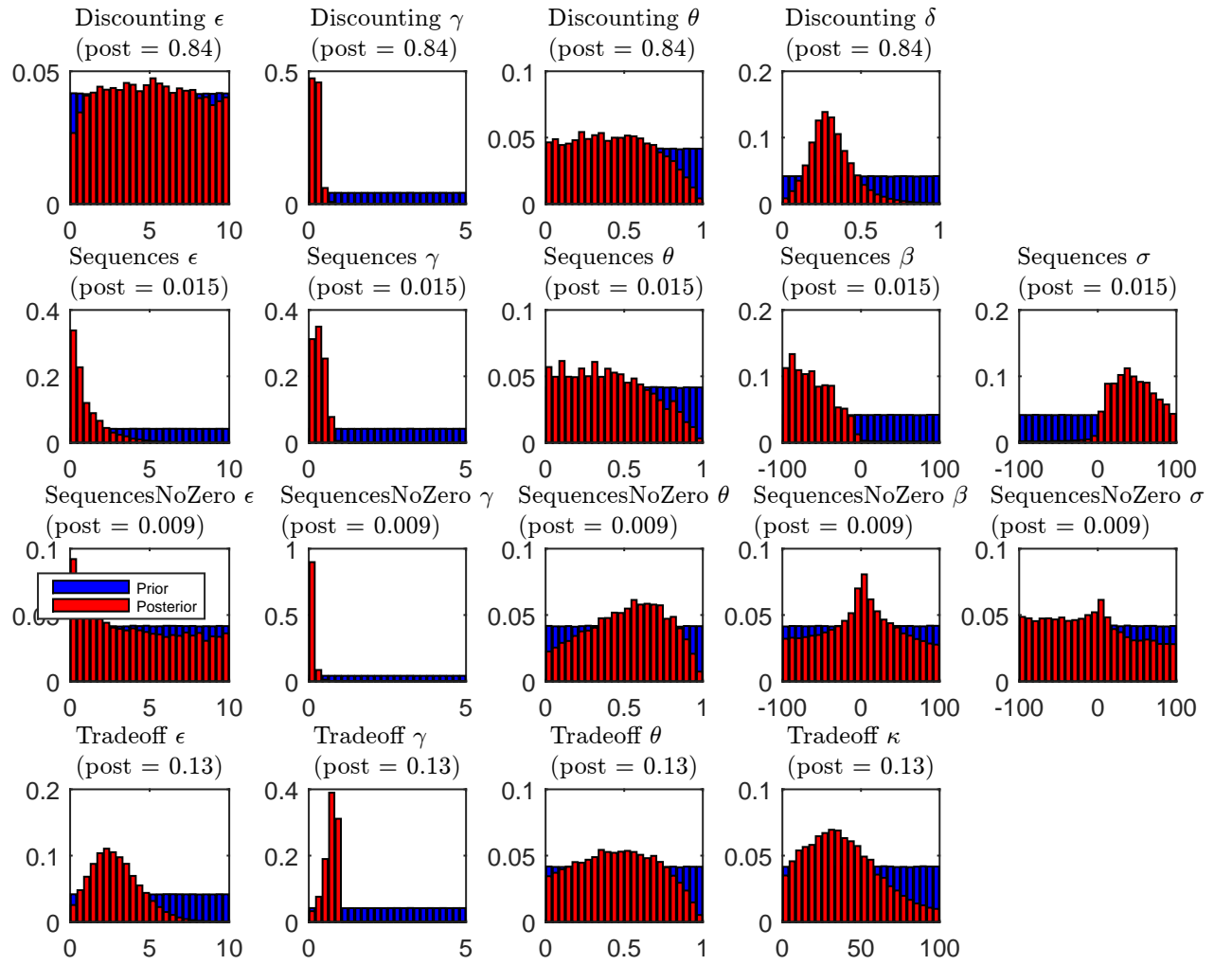

Supplement: Supplementary file 1 [file Scholten_Individuals.zip › plots/e29_p258_eg2_priors_and_posteriors.pdf]

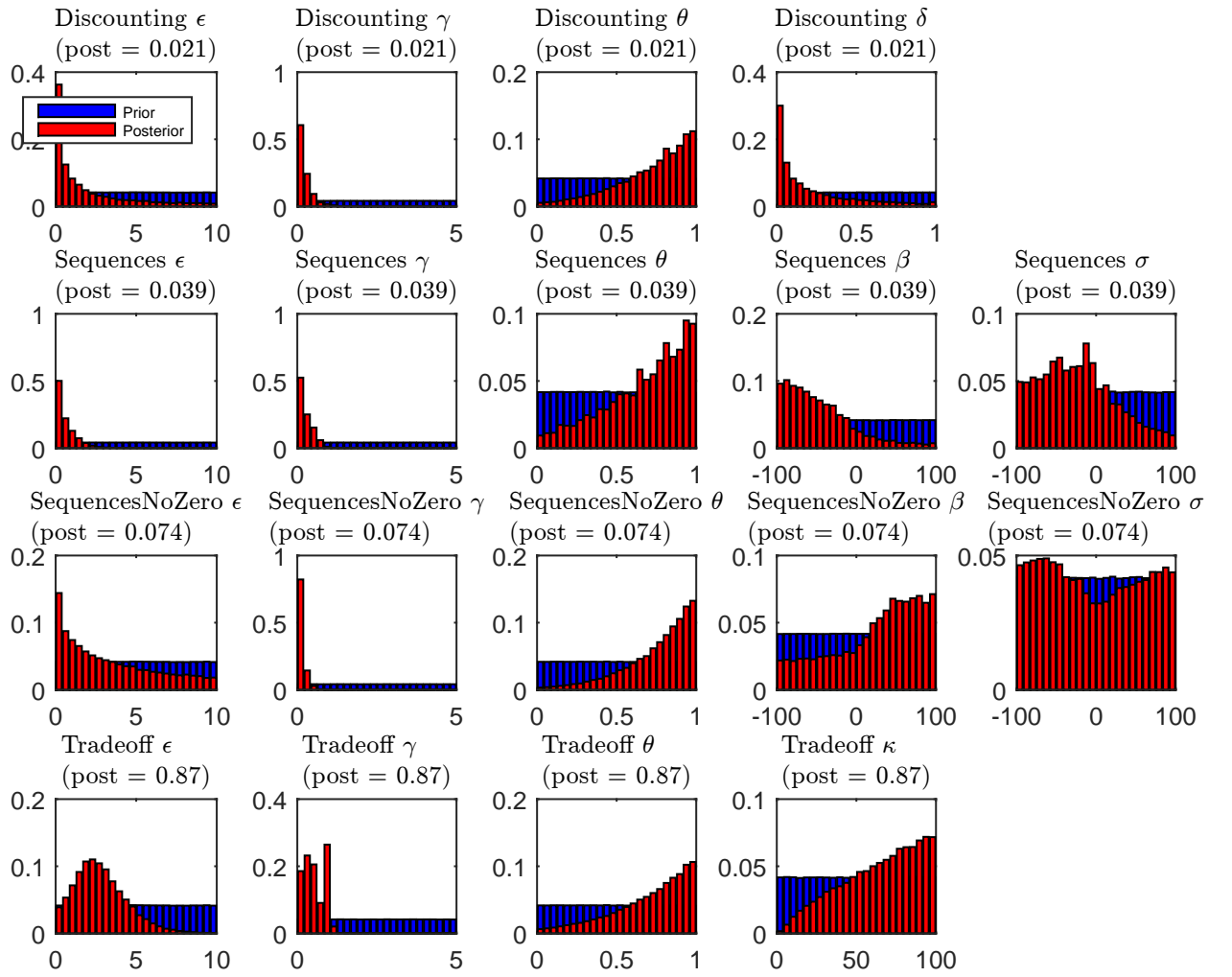

Supplement: Supplementary file 1 [file Scholten_Individuals.zip › plots/e29_p259_eg2_priors_and_posteriors.pdf]

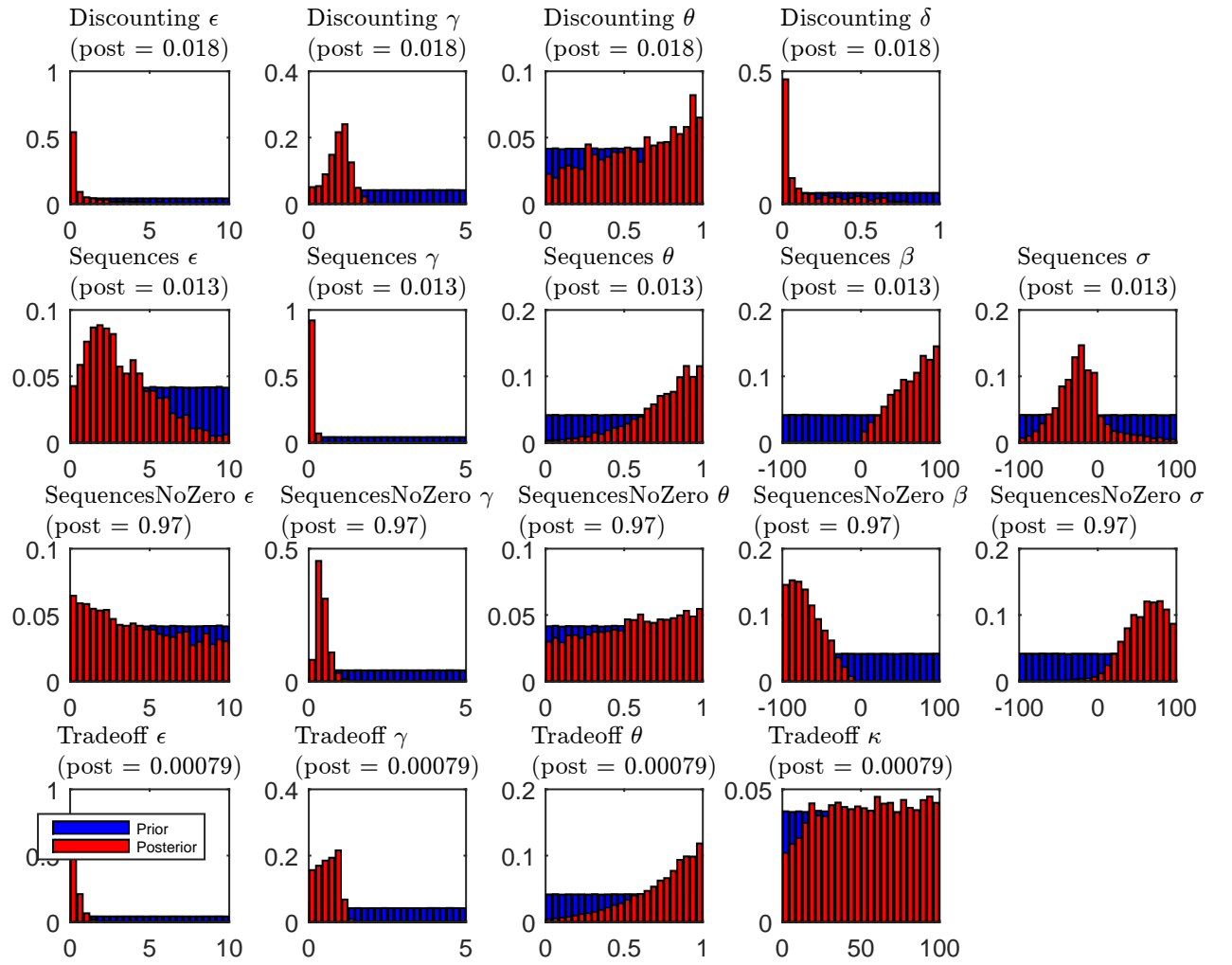

Supplement: Supplementary file 1 [file Scholten_Individuals.zip › plots/e29_p26_eg2_priors_and_posteriors.pdf]

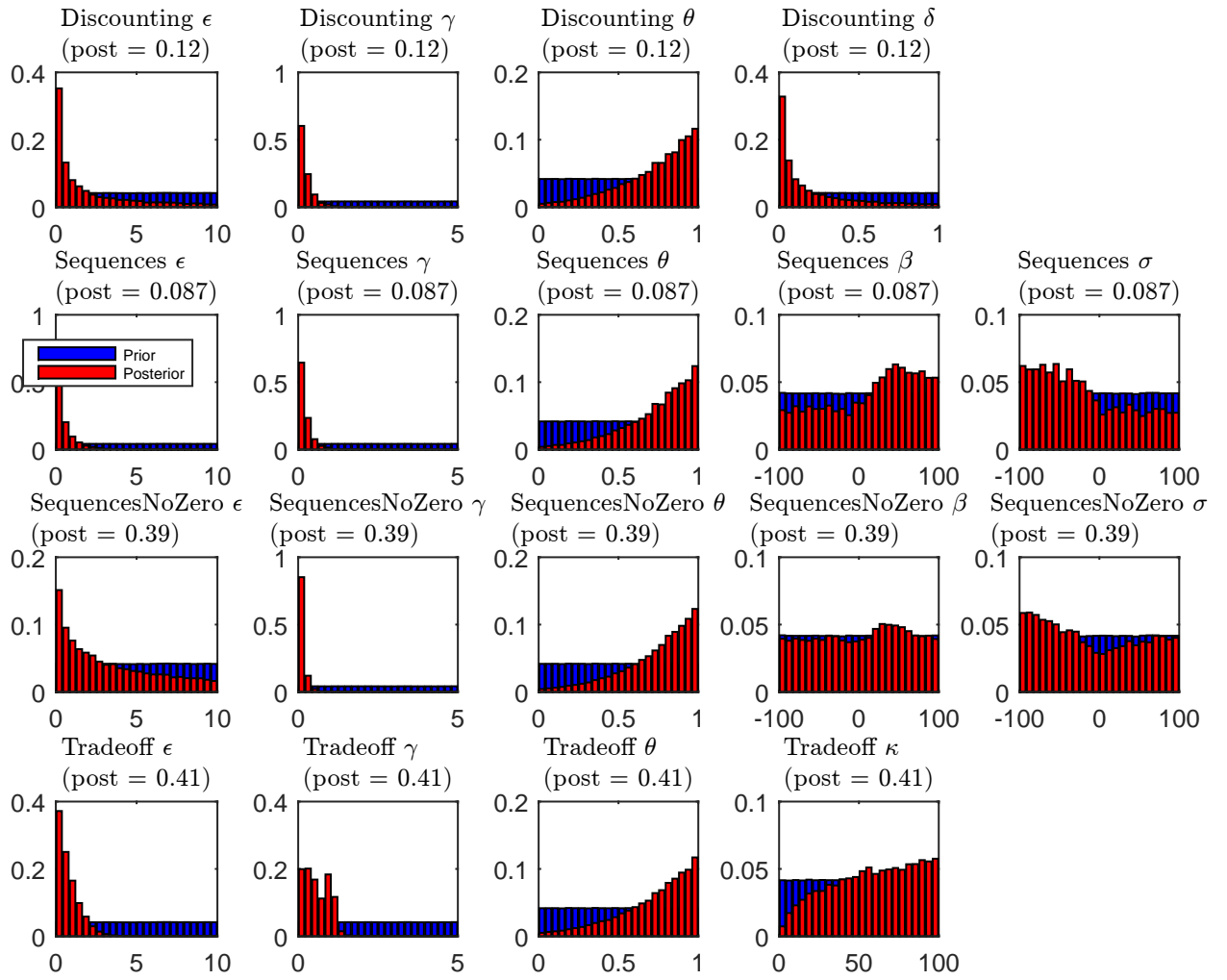

Supplement: Supplementary file 1 [file Scholten_Individuals.zip › plots/e29_p260_eg2_priors_and_posteriors.pdf]

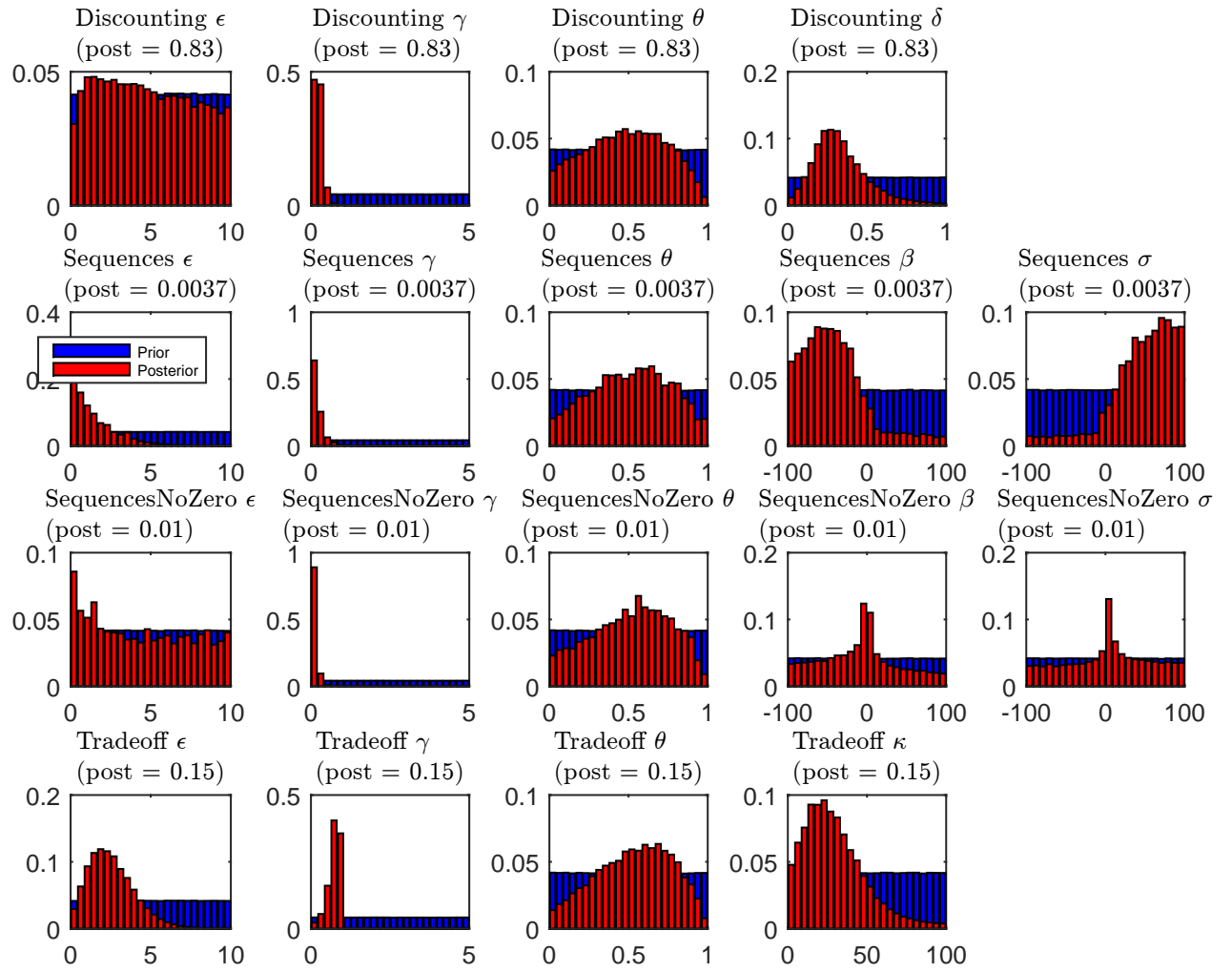

Supplement: Supplementary file 1 [file Scholten_Individuals.zip › plots/e29_p261_eg2_priors_and_posteriors.pdf]

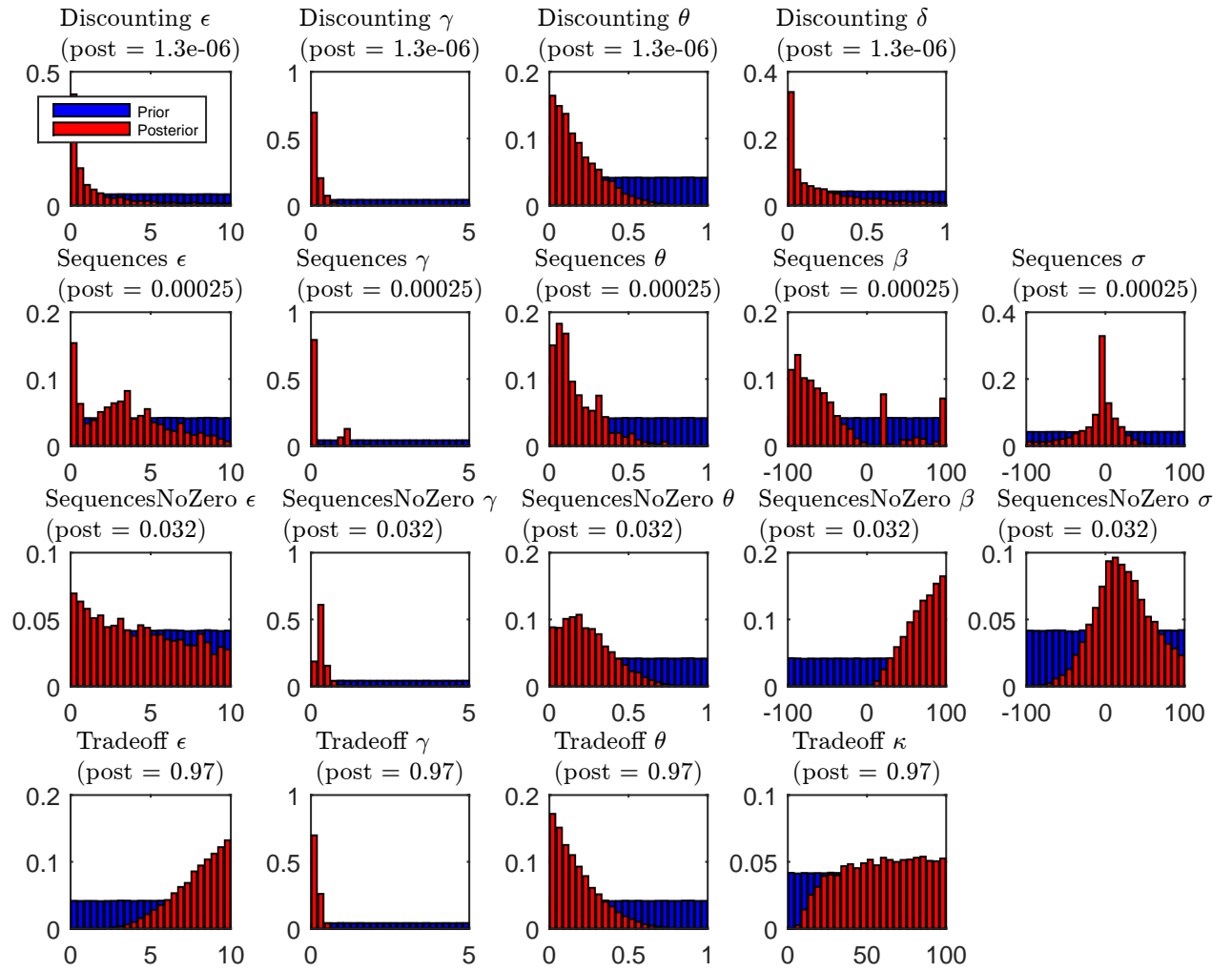

Supplement: Supplementary file 1 [file Scholten_Individuals.zip › plots/e29_p262_eg2_priors_and_posteriors.pdf]

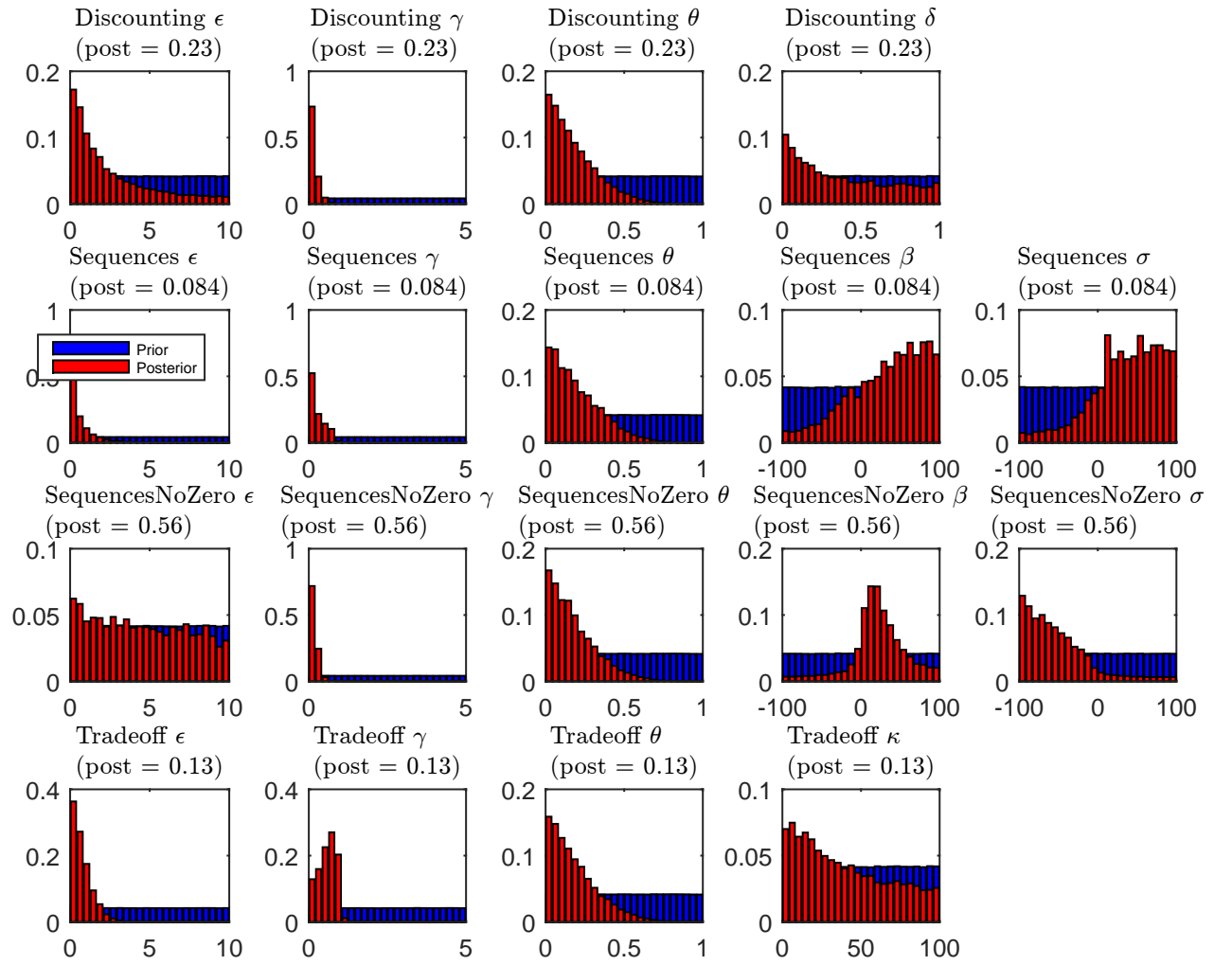

Supplement: Supplementary file 1 [file Scholten_Individuals.zip › plots/e29_p263_eg2_priors_and_posteriors.pdf]

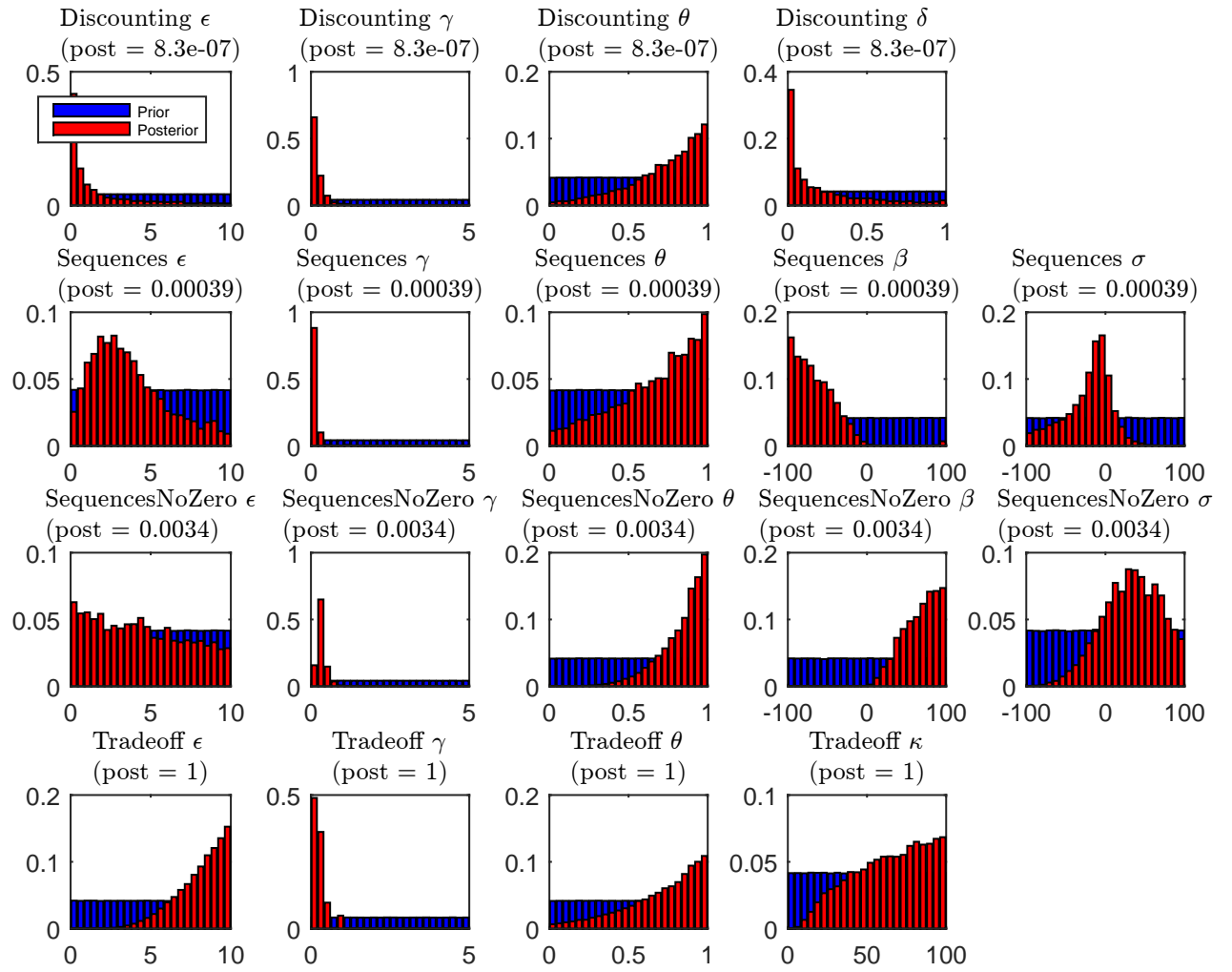

Supplement: Supplementary file 1 [file Scholten_Individuals.zip › plots/e29_p264_eg2_priors_and_posteriors.pdf]

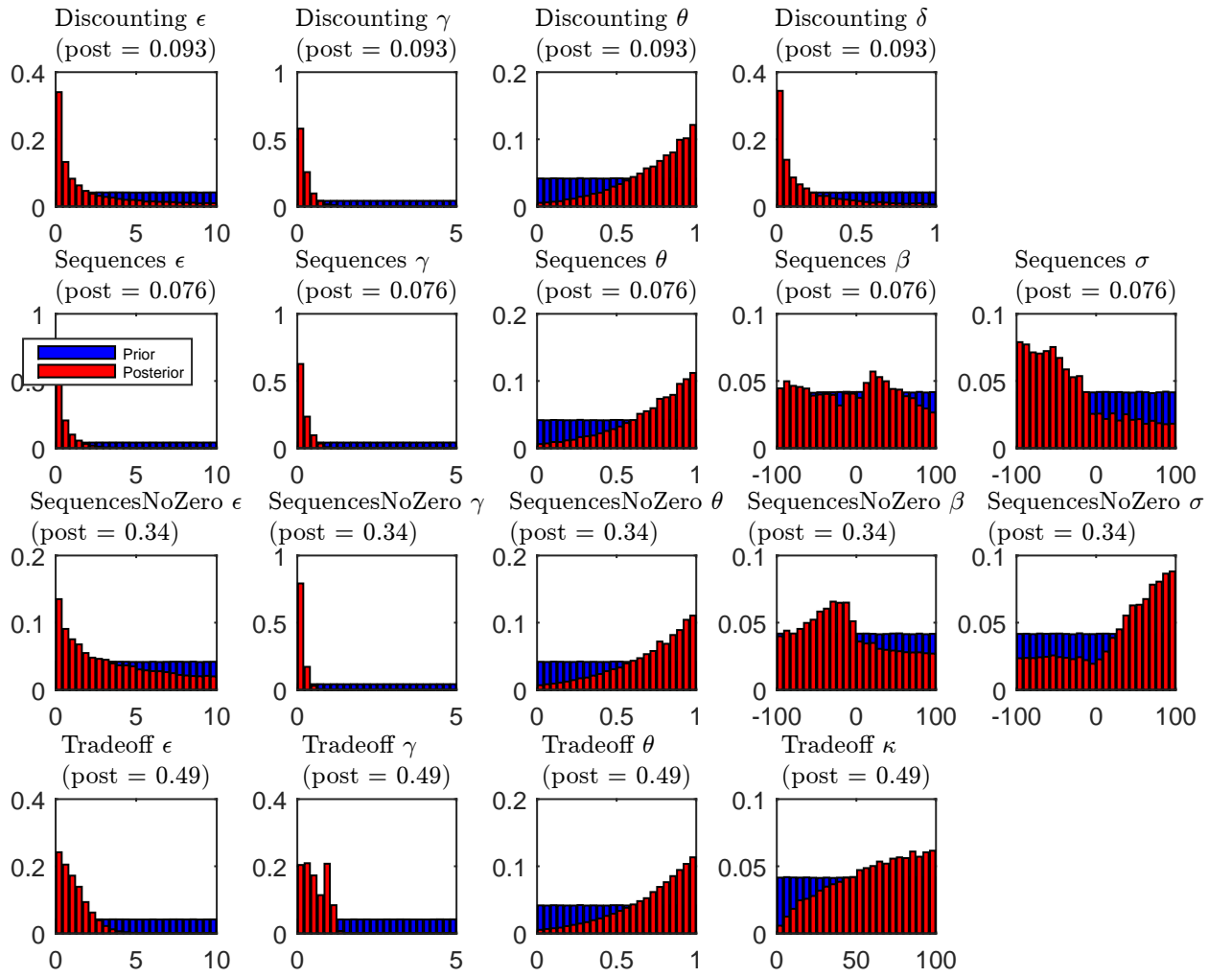

Supplement: Supplementary file 1 [file Scholten_Individuals.zip › plots/e29_p265_eg2_priors_and_posteriors.pdf]

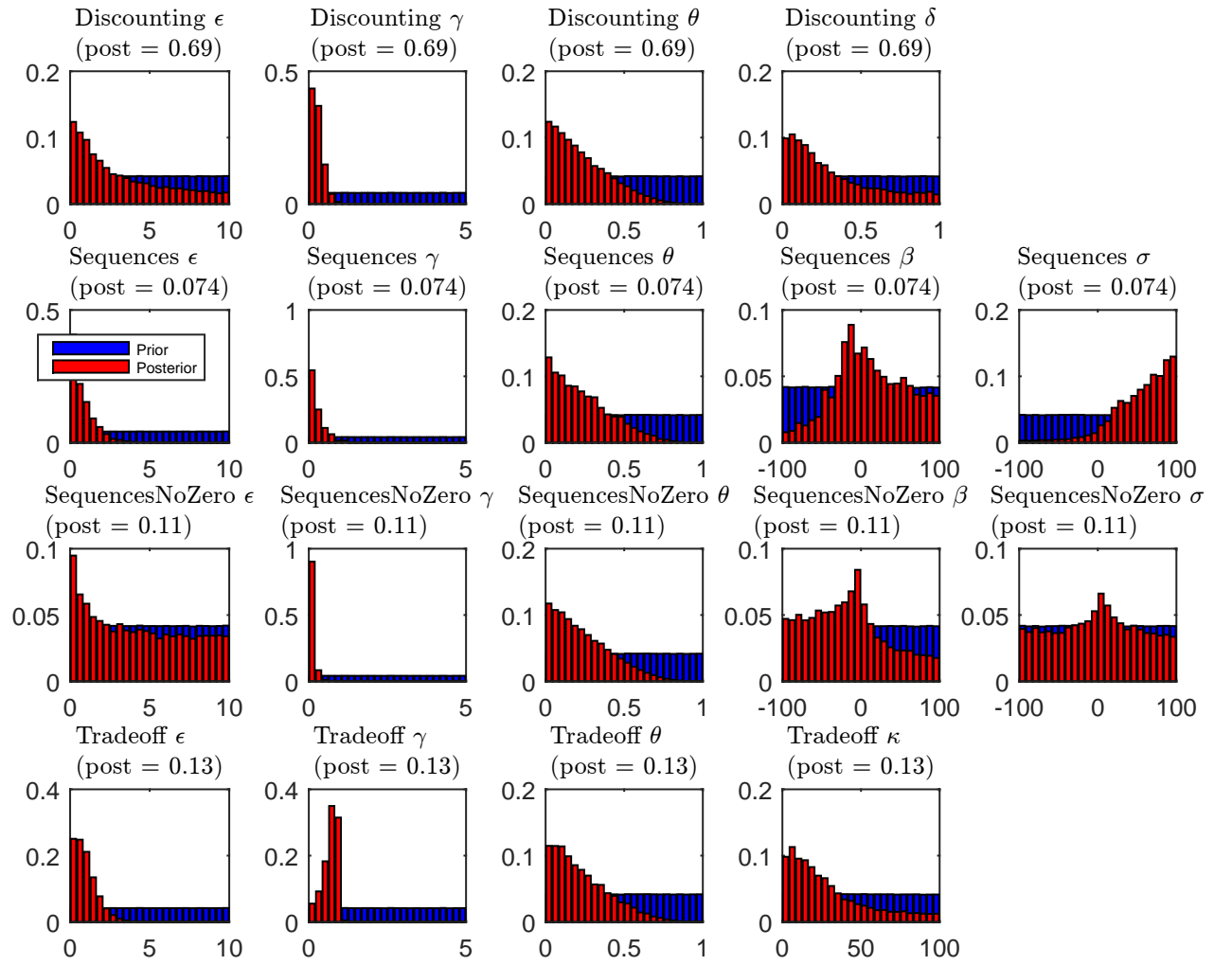

Supplement: Supplementary file 1 [file Scholten_Individuals.zip › plots/e29_p266_eg2_priors_and_posteriors.pdf]

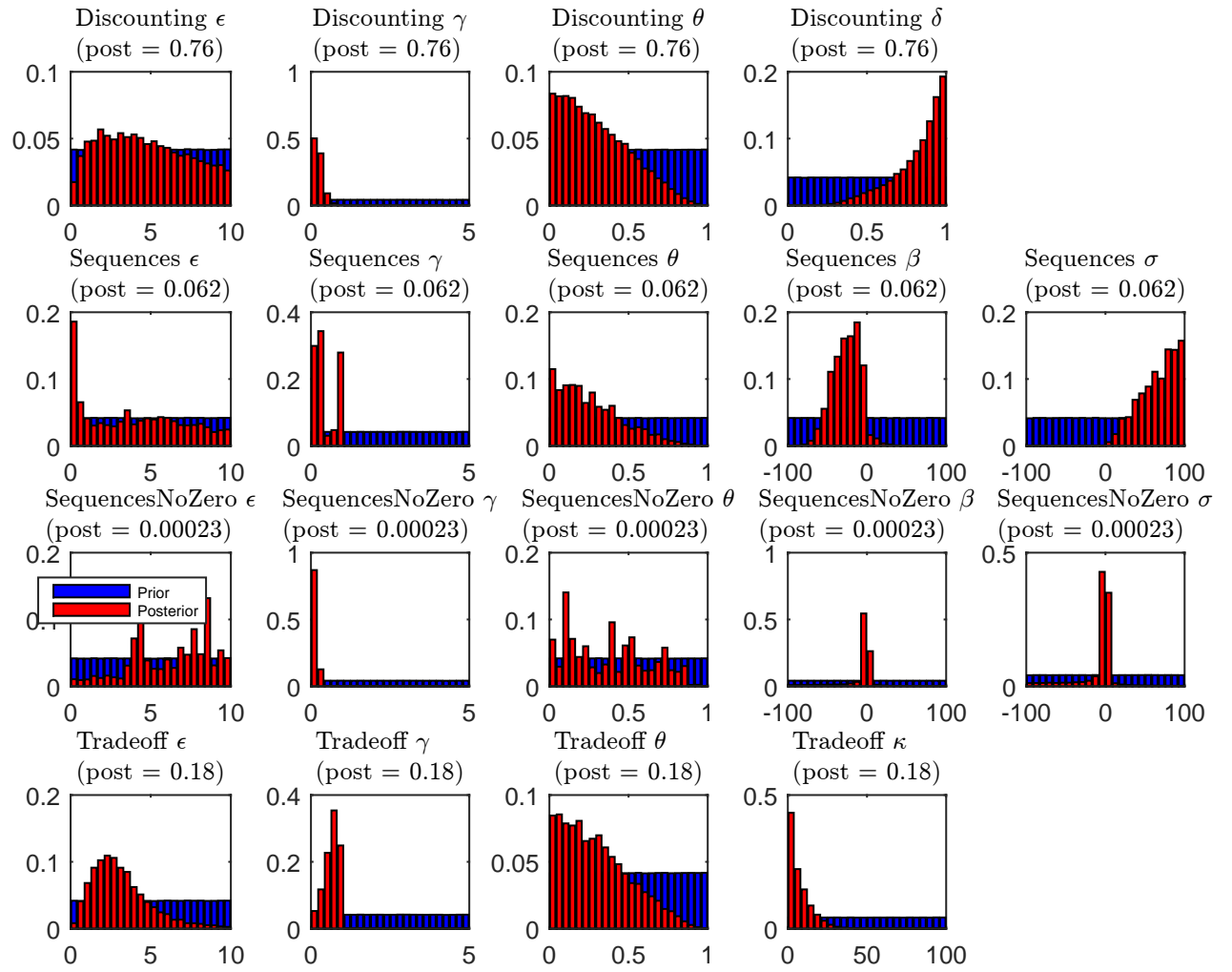

Supplement: Supplementary file 1 [file Scholten_Individuals.zip › plots/e29_p267_eg2_priors_and_posteriors.pdf]

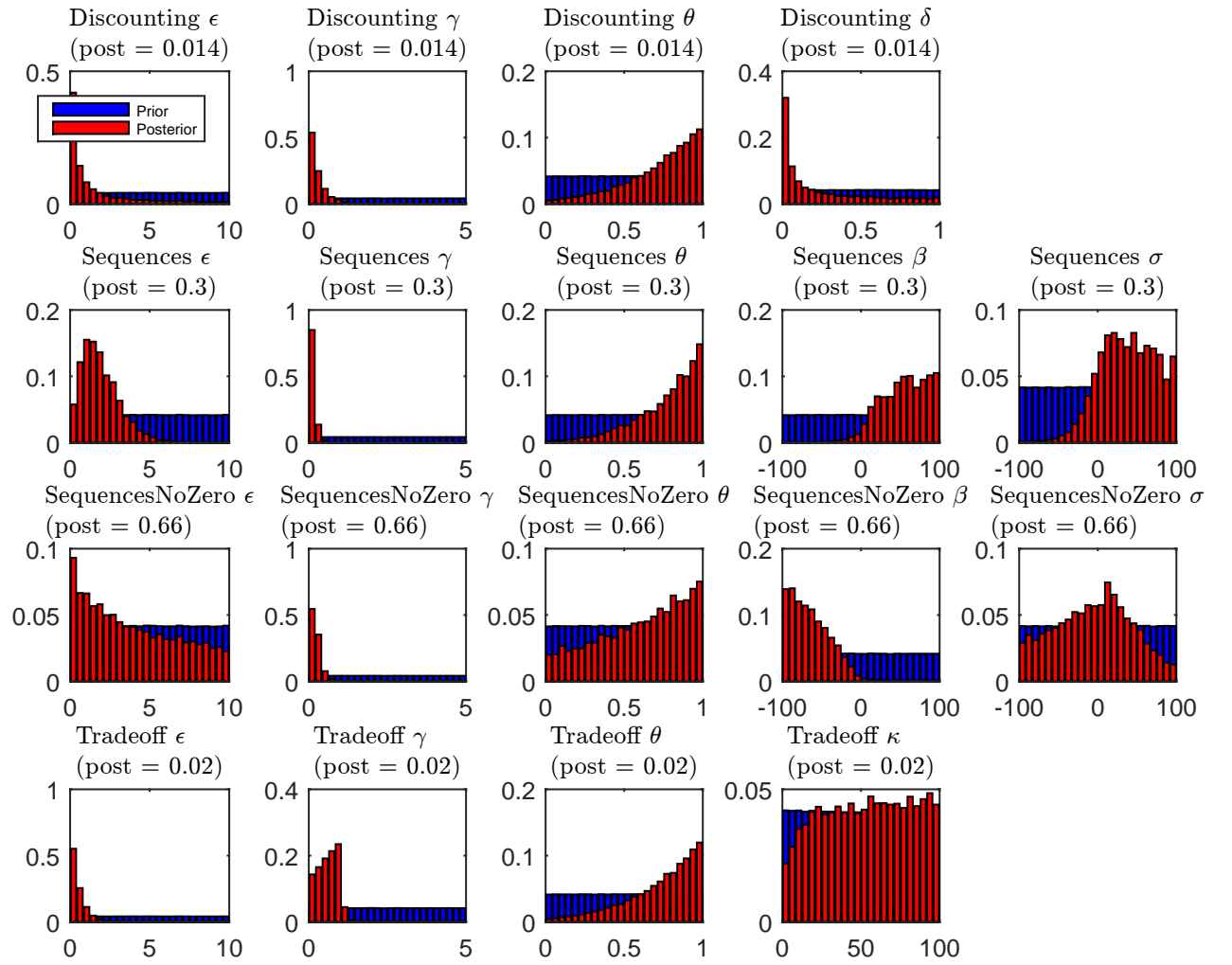

Supplement: Supplementary file 1 [file Scholten_Individuals.zip › plots/e29_p268_eg2_priors_and_posteriors.pdf]

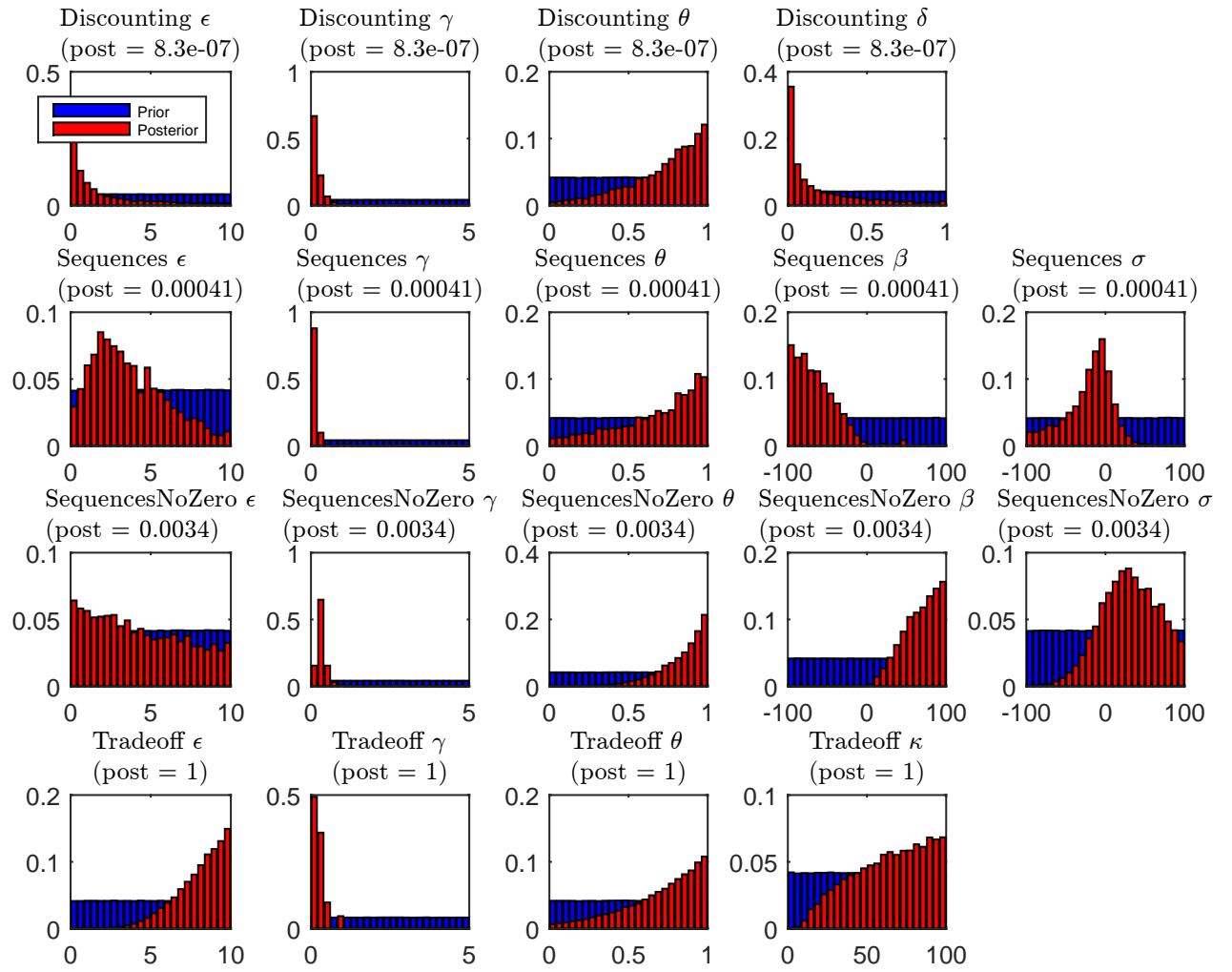

Supplement: Supplementary file 1 [file Scholten_Individuals.zip › plots/e29_p269_eg2_priors_and_posteriors.pdf]

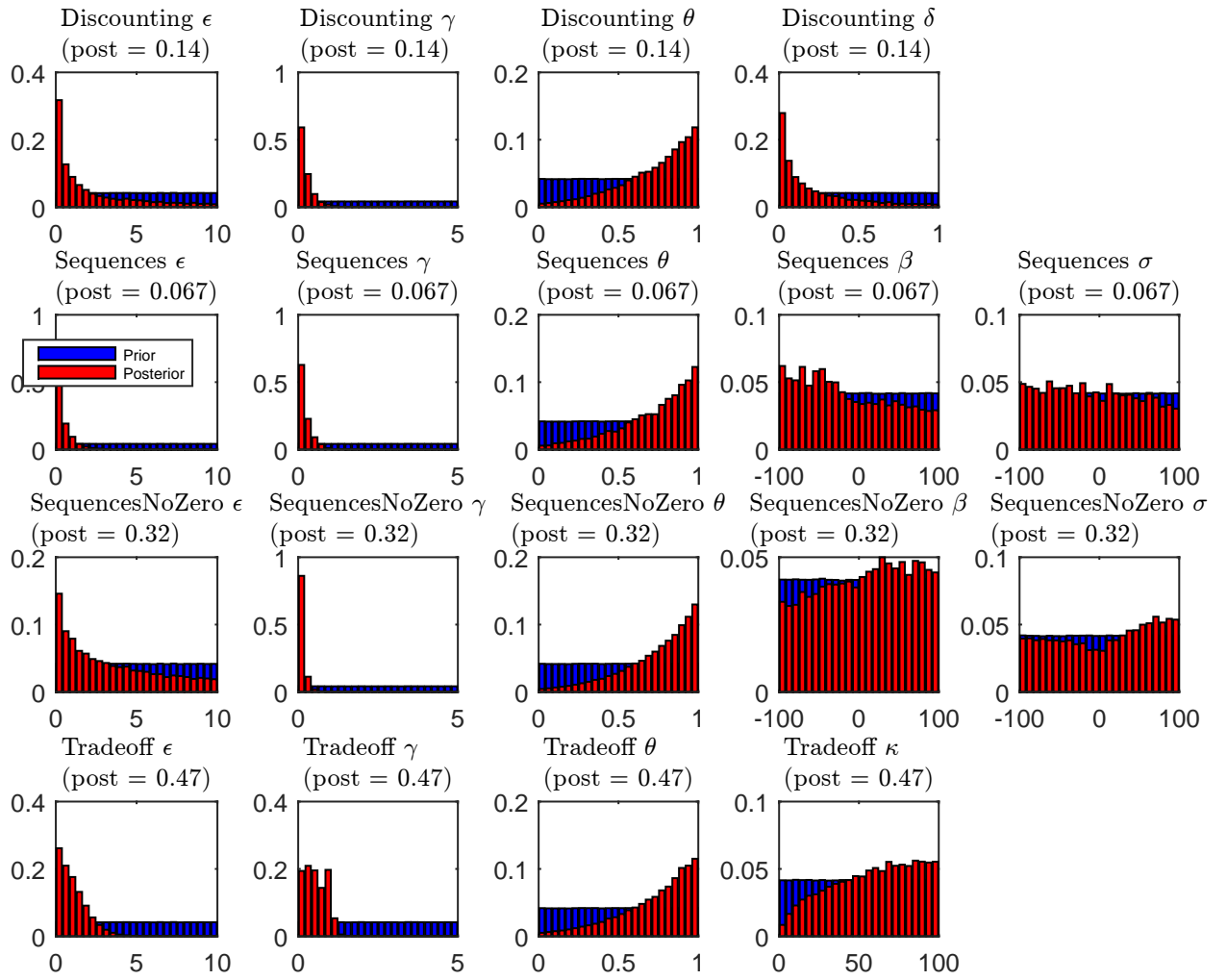

Supplement: Supplementary file 1 [file Scholten_Individuals.zip › plots/e29_p27_eg2_priors_and_posteriors.pdf]

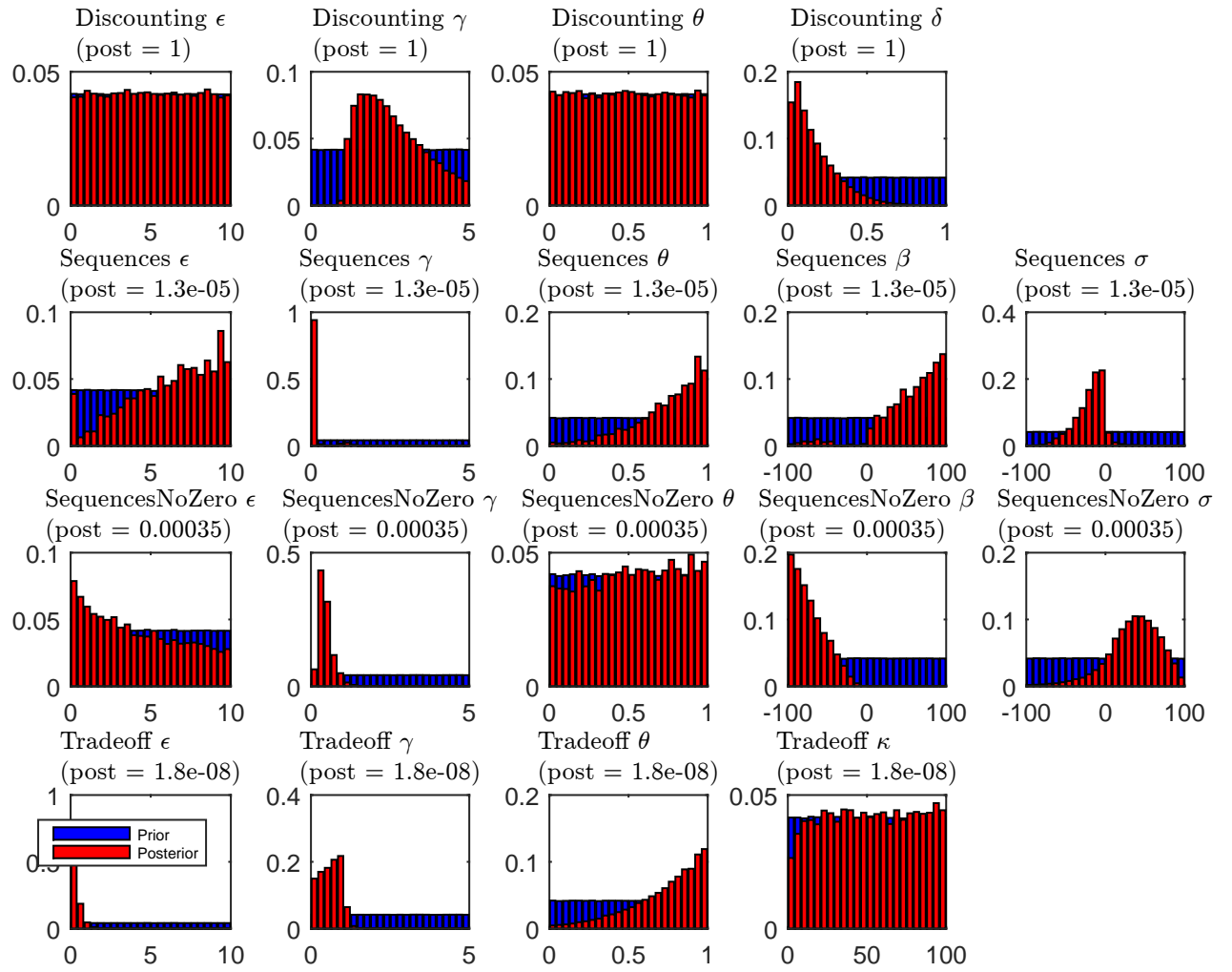

Supplement: Supplementary file 1 [file Scholten_Individuals.zip › plots/e29_p270_eg2_priors_and_posteriors.pdf]

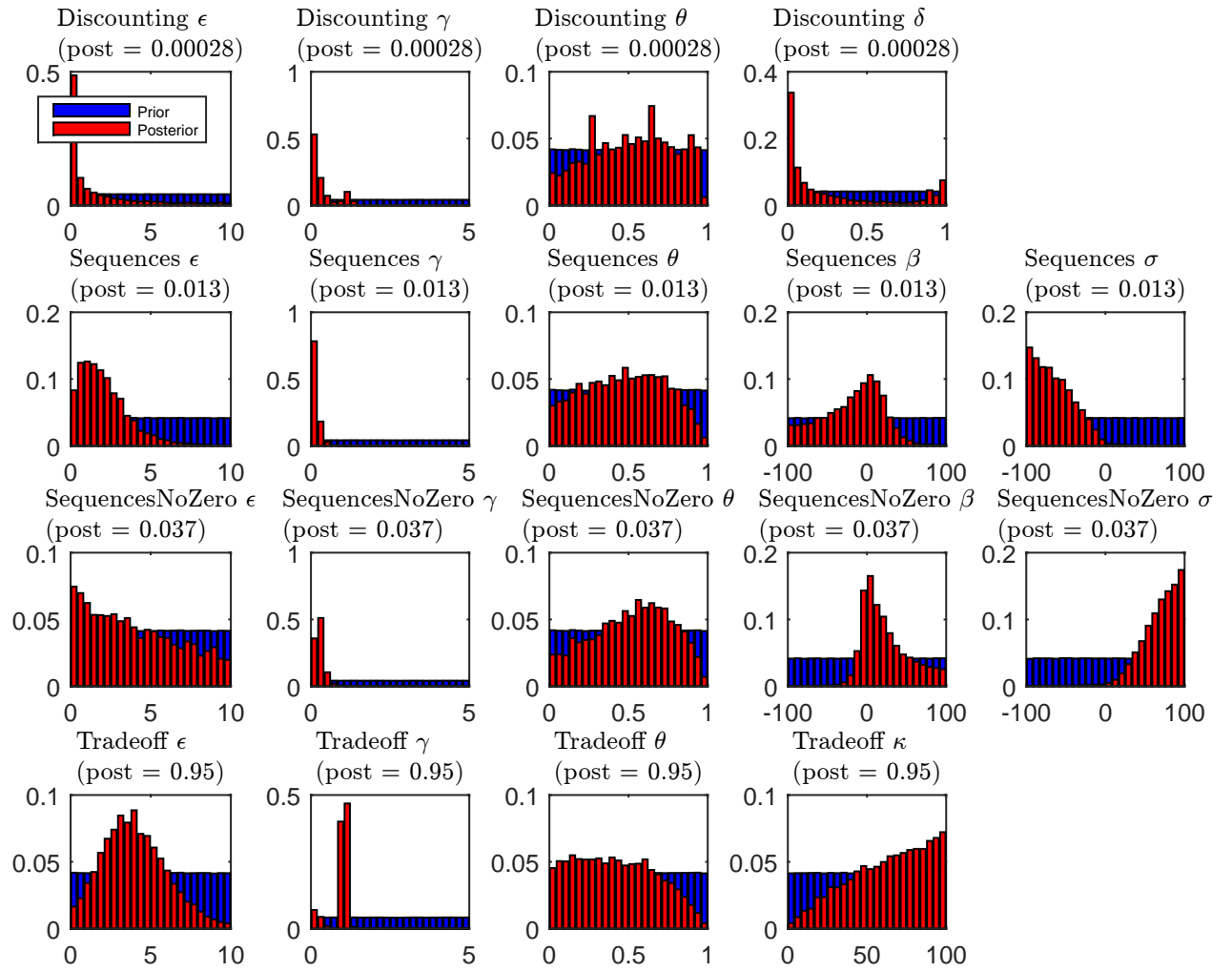

Supplement: Supplementary file 1 [file Scholten_Individuals.zip › plots/e29_p271_eg2_priors_and_posteriors.pdf]

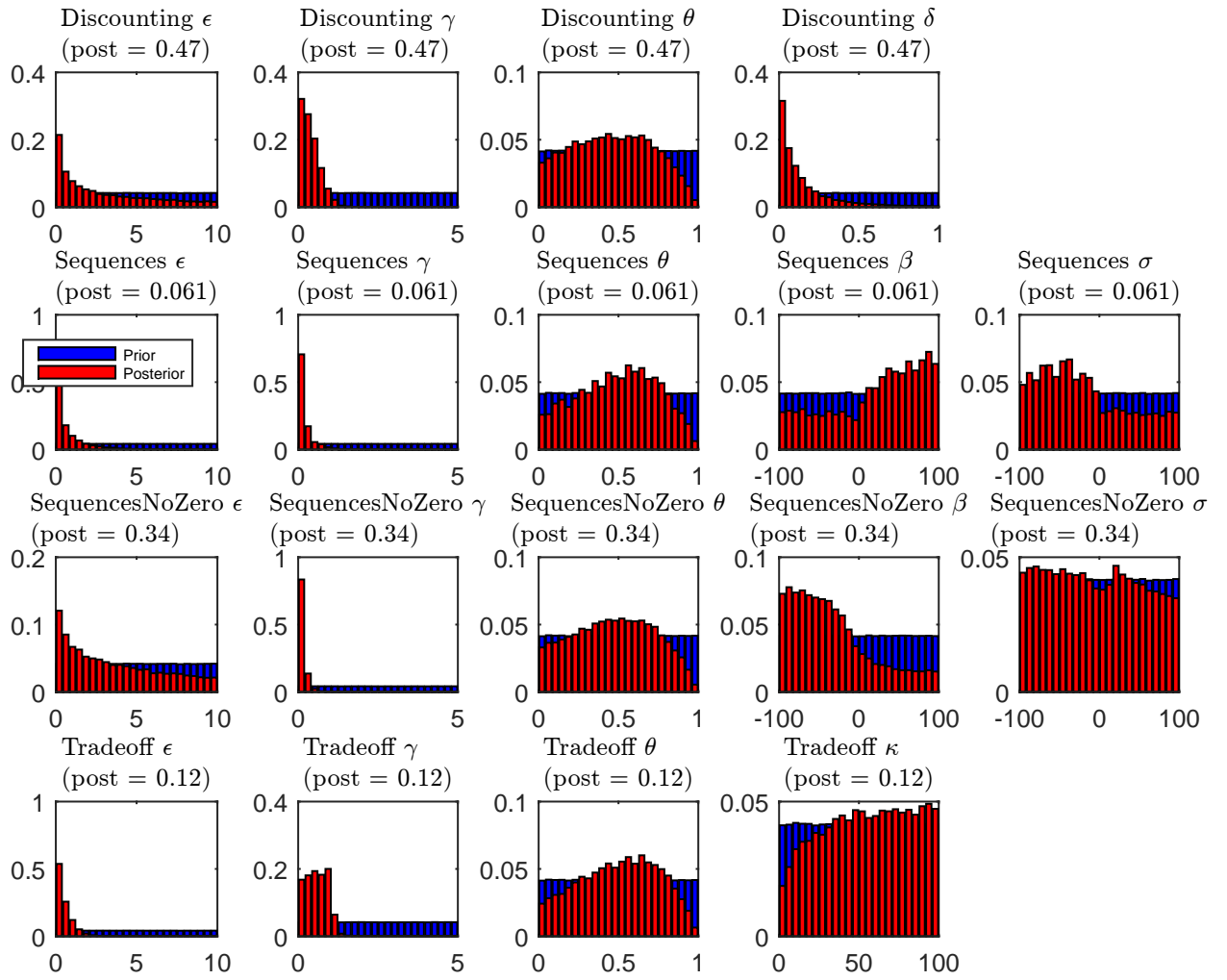

Supplement: Supplementary file 1 [file Scholten_Individuals.zip › plots/e29_p272_eg2_priors_and_posteriors.pdf]

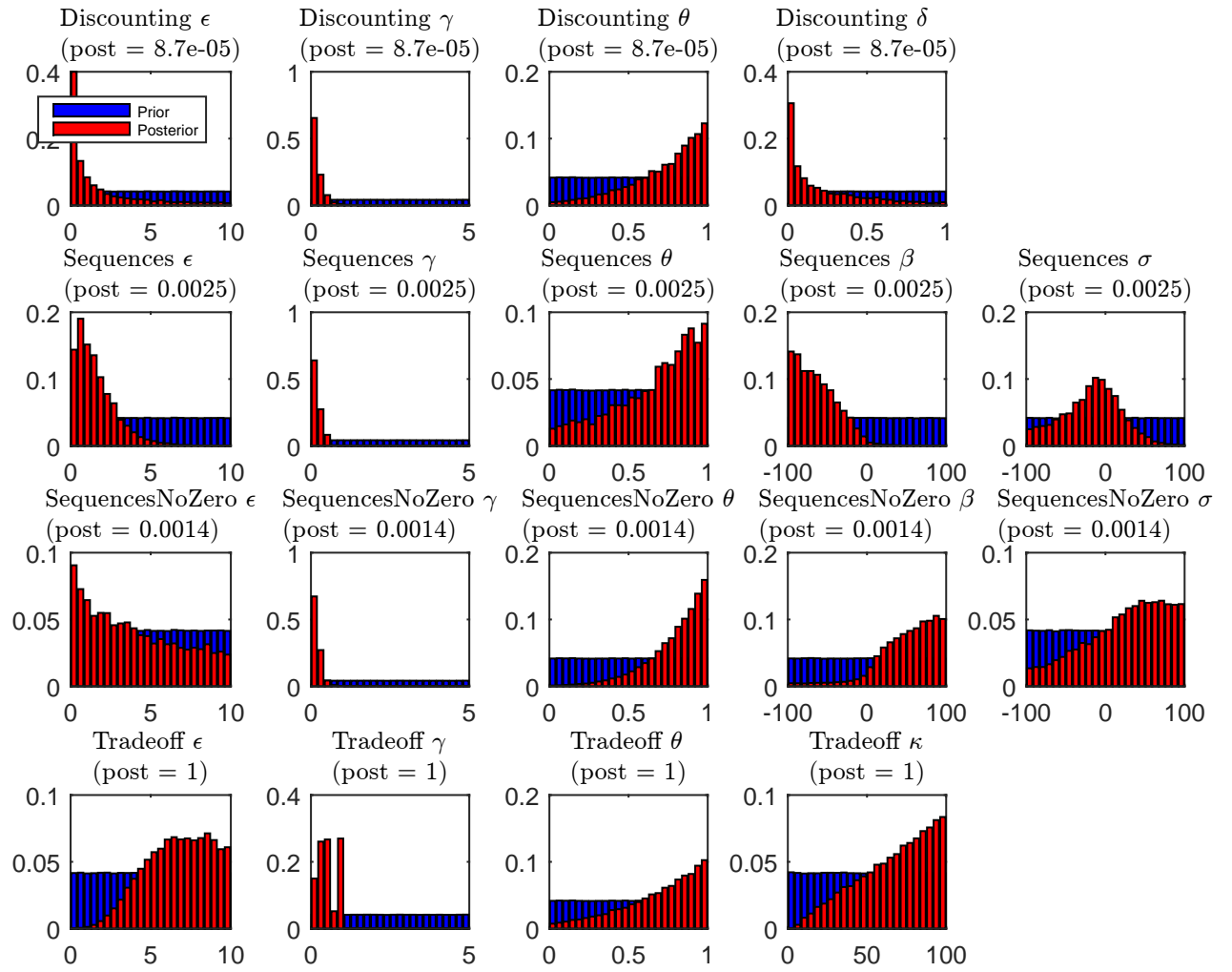

Supplement: Supplementary file 1 [file Scholten_Individuals.zip › plots/e29_p273_eg2_priors_and_posteriors.pdf]

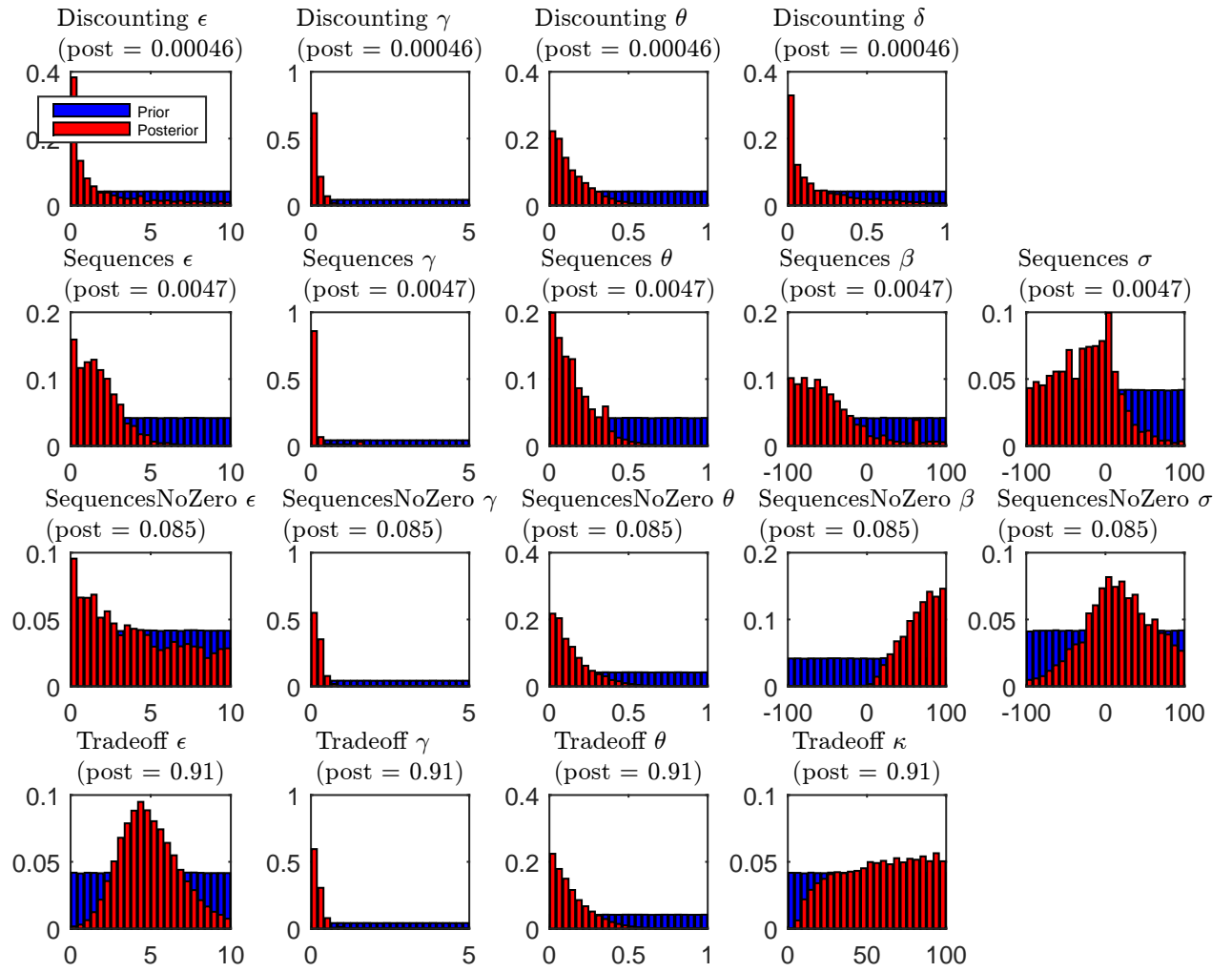

Supplement: Supplementary file 1 [file Scholten_Individuals.zip › plots/e29_p274_eg2_priors_and_posteriors.pdf]

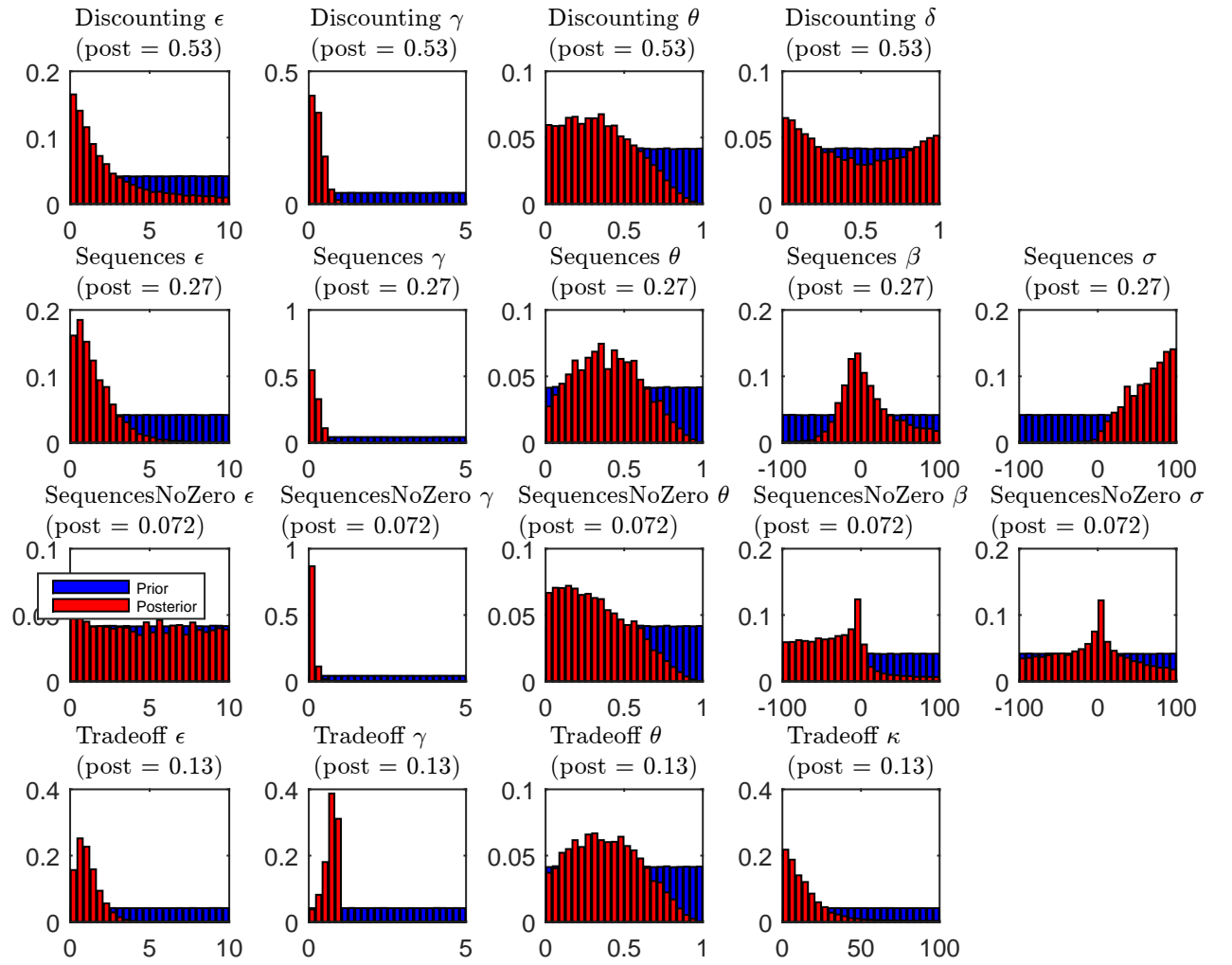

Supplement: Supplementary file 1 [file Scholten_Individuals.zip › plots/e29_p275_eg2_priors_and_posteriors.pdf]

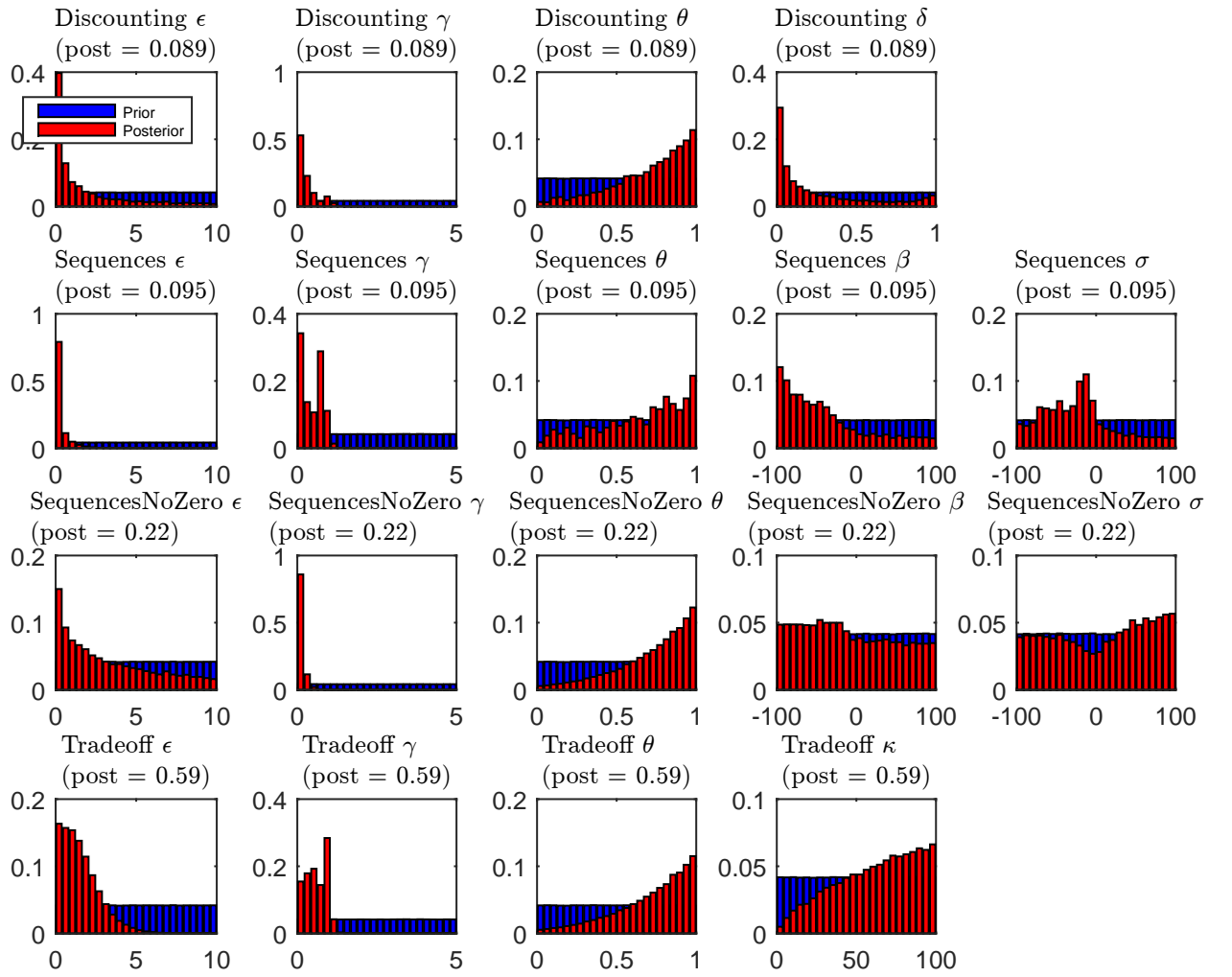

Supplement: Supplementary file 1 [file Scholten_Individuals.zip › plots/e29_p276_eg2_priors_and_posteriors.pdf]

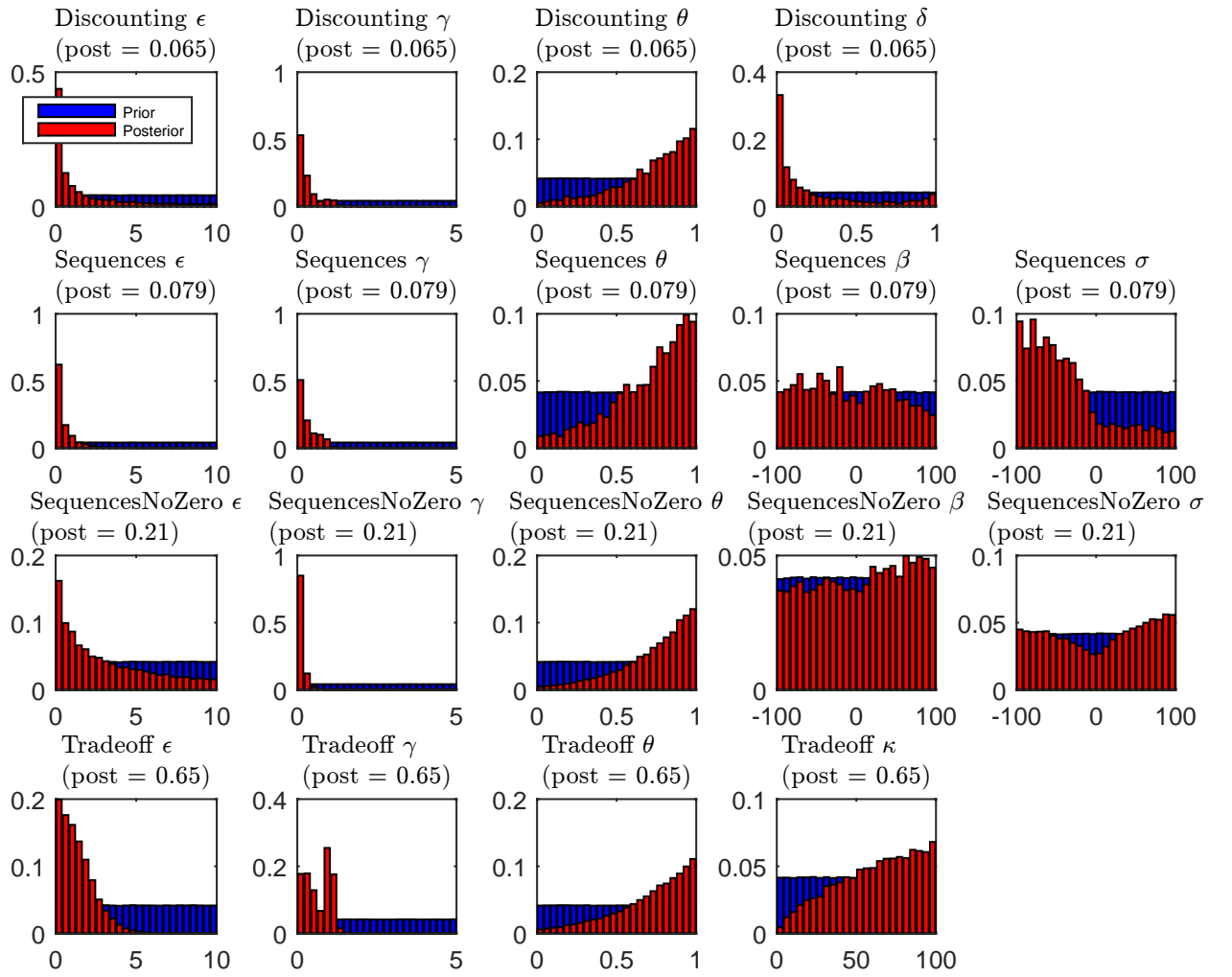

Supplement: Supplementary file 1 [file Scholten_Individuals.zip › plots/e29_p277_eg2_priors_and_posteriors.pdf]

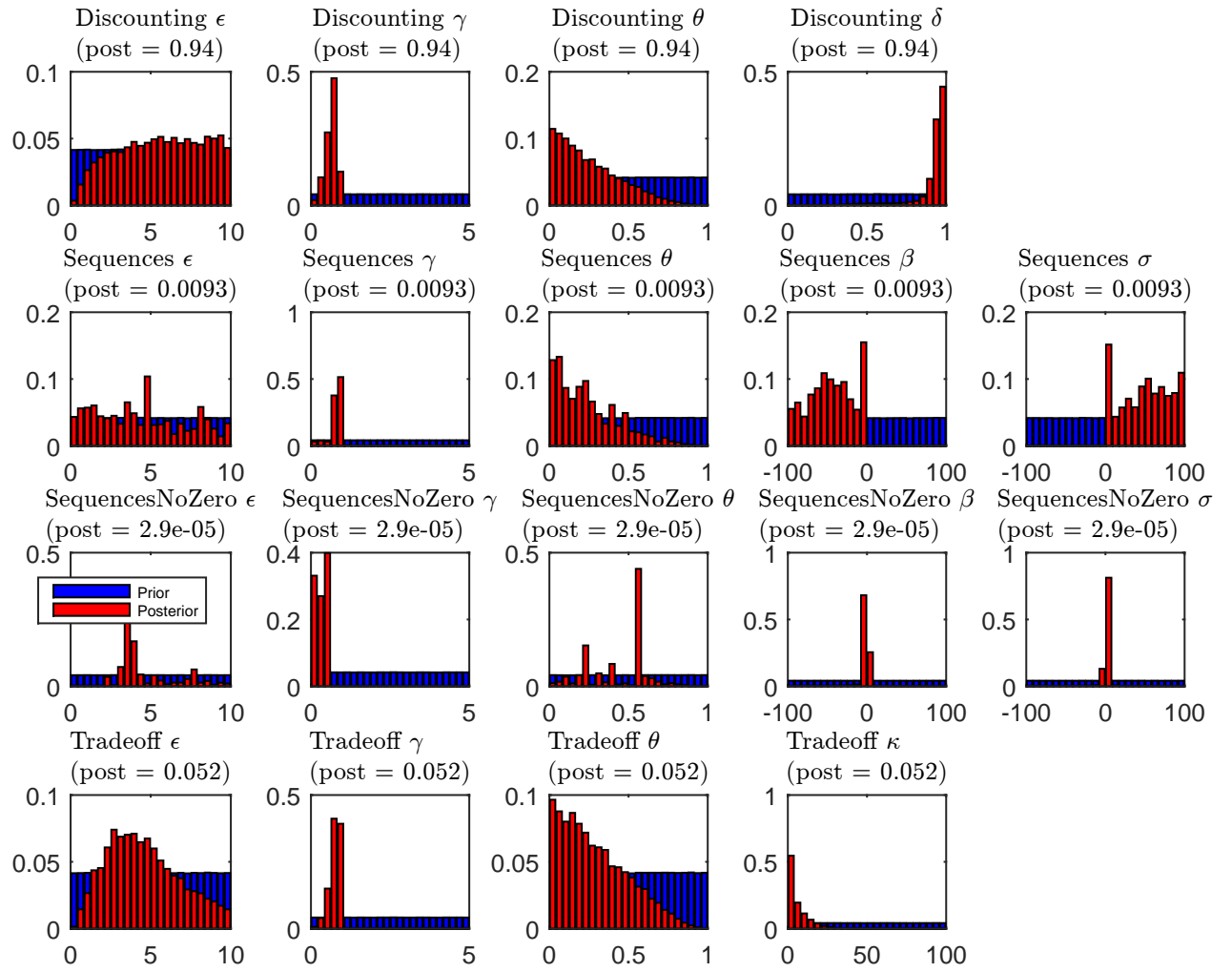

Supplement: Supplementary file 1 [file Scholten_Individuals.zip › plots/e29_p278_eg2_priors_and_posteriors.pdf]

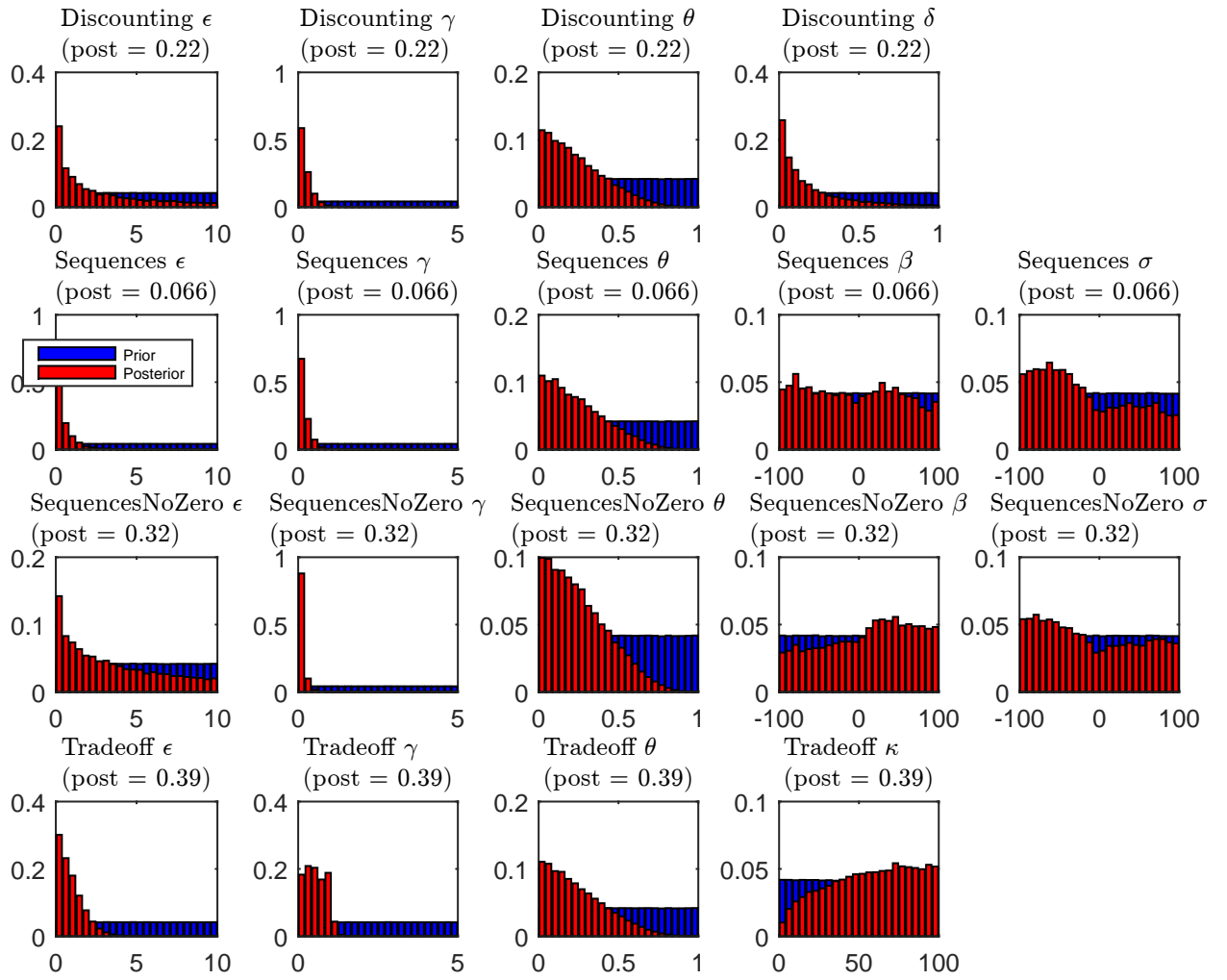

Supplement: Supplementary file 1 [file Scholten_Individuals.zip › plots/e29_p279_eg2_priors_and_posteriors.pdf]

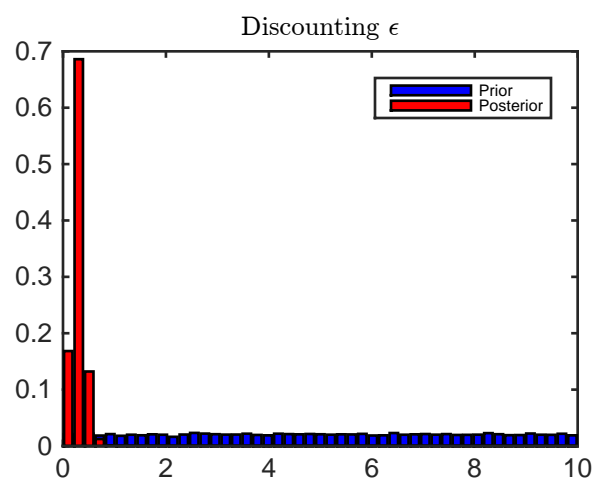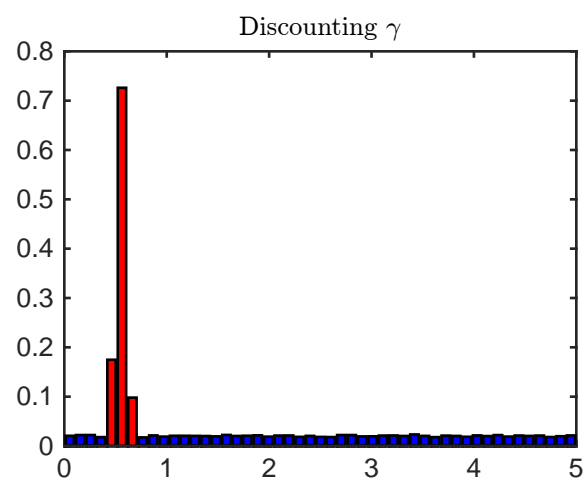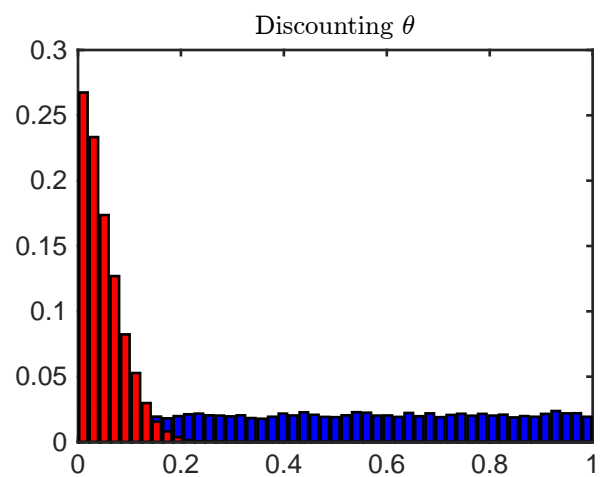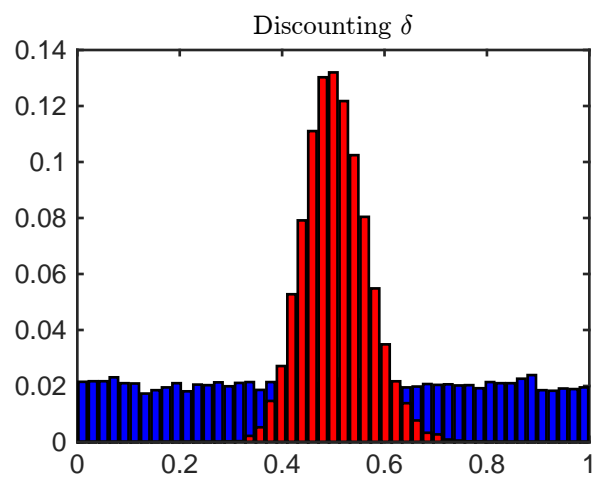

Supplement: Supplementary file 2 [file Scholten.zip › Scholten, Read, and Sanborn. Plots Posteriors. Groups/E29 Discounting Priors and Posteriors.pdf]

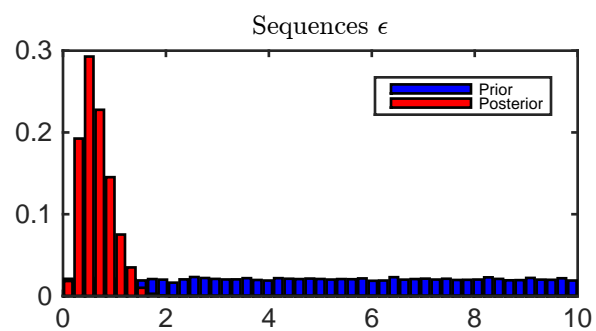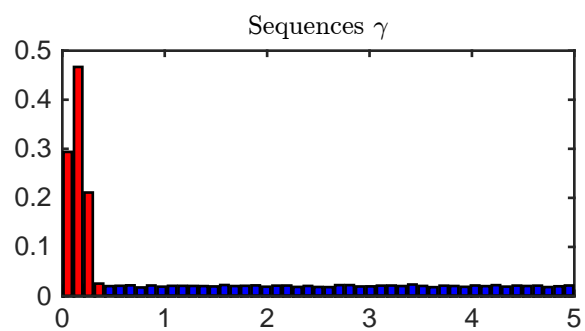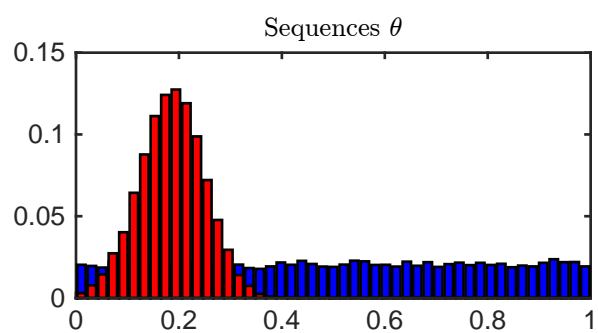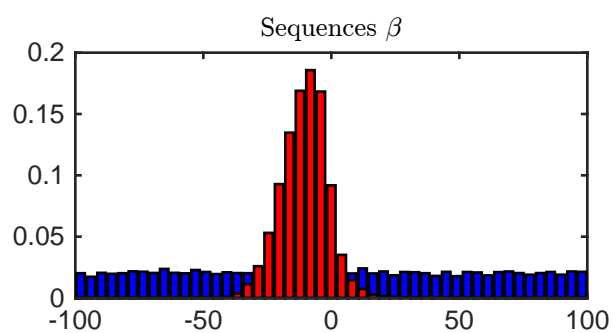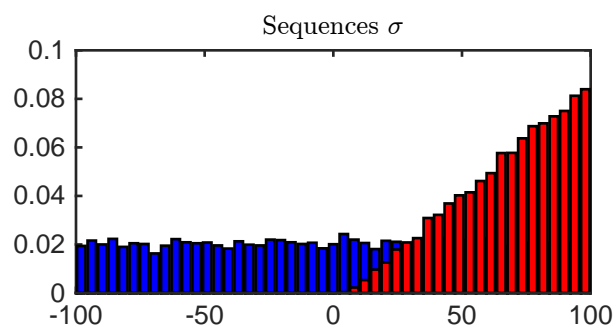

Supplement: Supplementary file 2 [file Scholten.zip › Scholten, Read, and Sanborn. Plots Posteriors. Groups/E29 Sequences Priors and Posteriors.pdf]

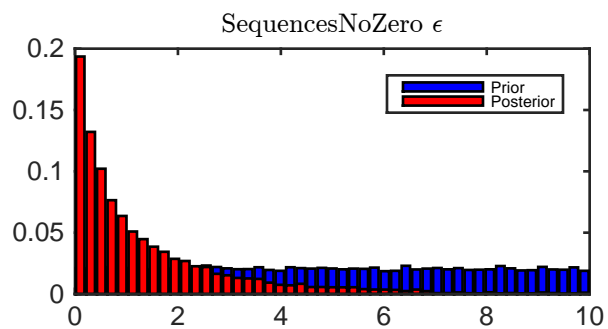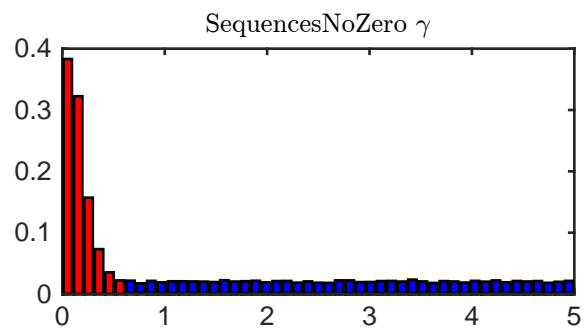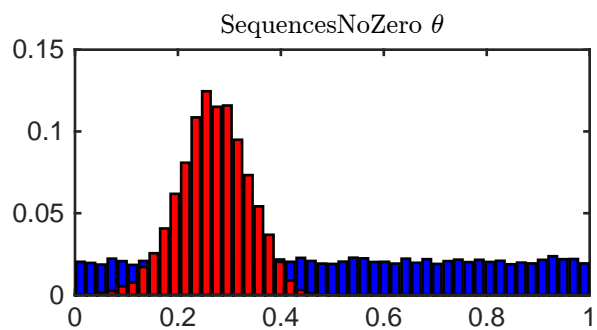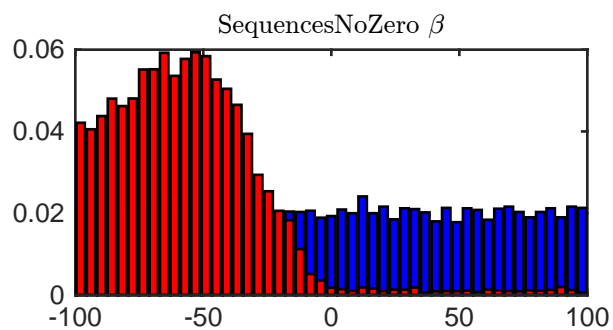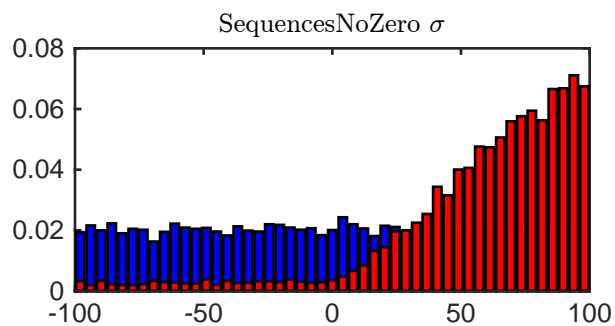

Supplement: Supplementary file 2 [file Scholten.zip › Scholten, Read, and Sanborn. Plots Posteriors. Groups/E29 SequencesNoZero Priors and Posteriors.pdf]
