# Supplementary figures and images for: Cumulative Weighing of Time in Intertemporal Tradeoffs (part 1 of 3)
Source: J Exp Psychol Gen. 2016 Sep;145(9):1177–205. doi: 10.1037/xge0000198 (PMC4998108; doi:10.1037/xge0000198)

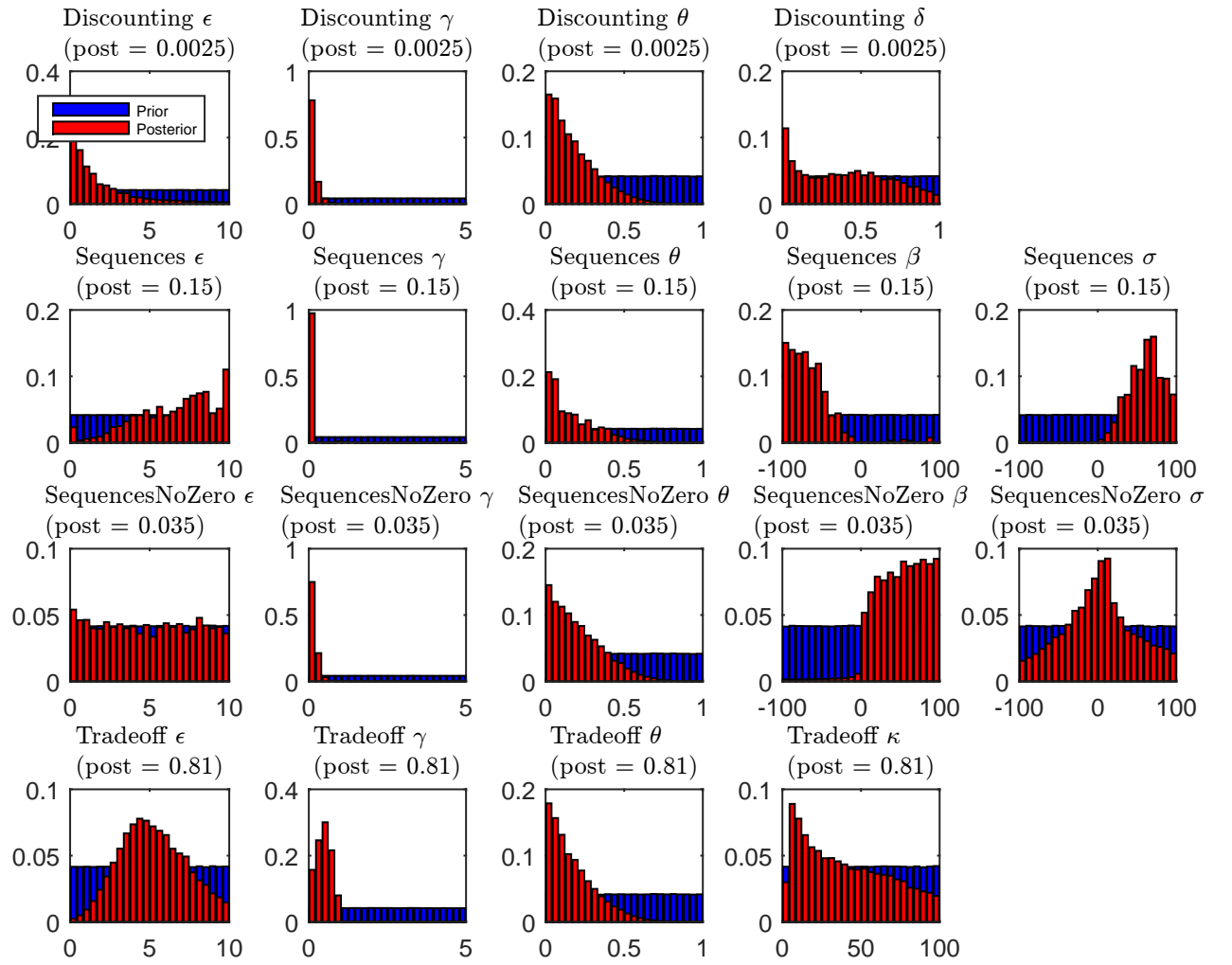

Supplement: Supplementary file 1 [file Scholten_Individuals.zip › plots/e29_p1_eg2_priors_and_posteriors.pdf]

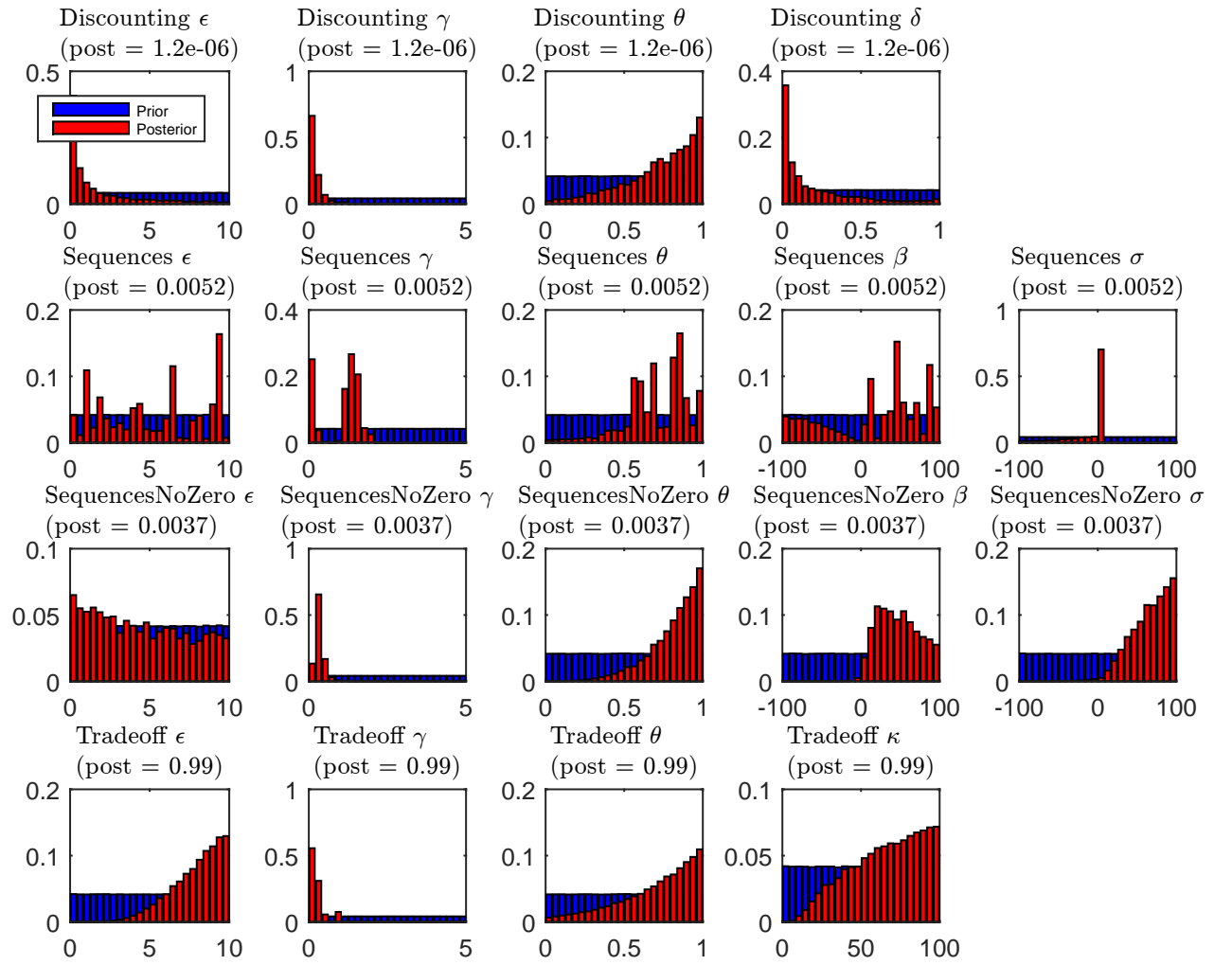

Supplement: Supplementary file 1 [file Scholten_Individuals.zip › plots/e29_p10_eg2_priors_and_posteriors.pdf]

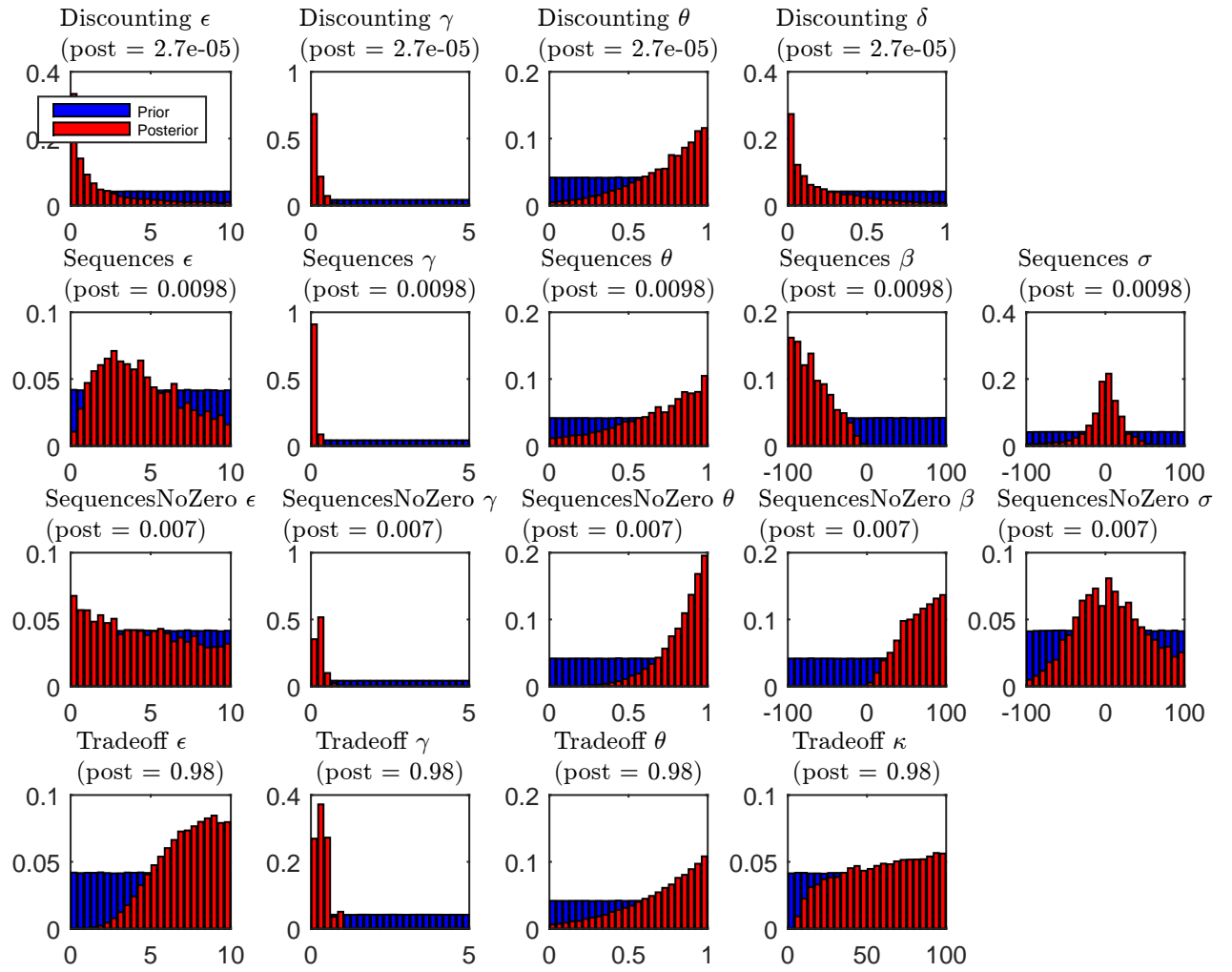

Supplement: Supplementary file 1 [file Scholten_Individuals.zip › plots/e29_p100_eg2_priors_and_posteriors.pdf]

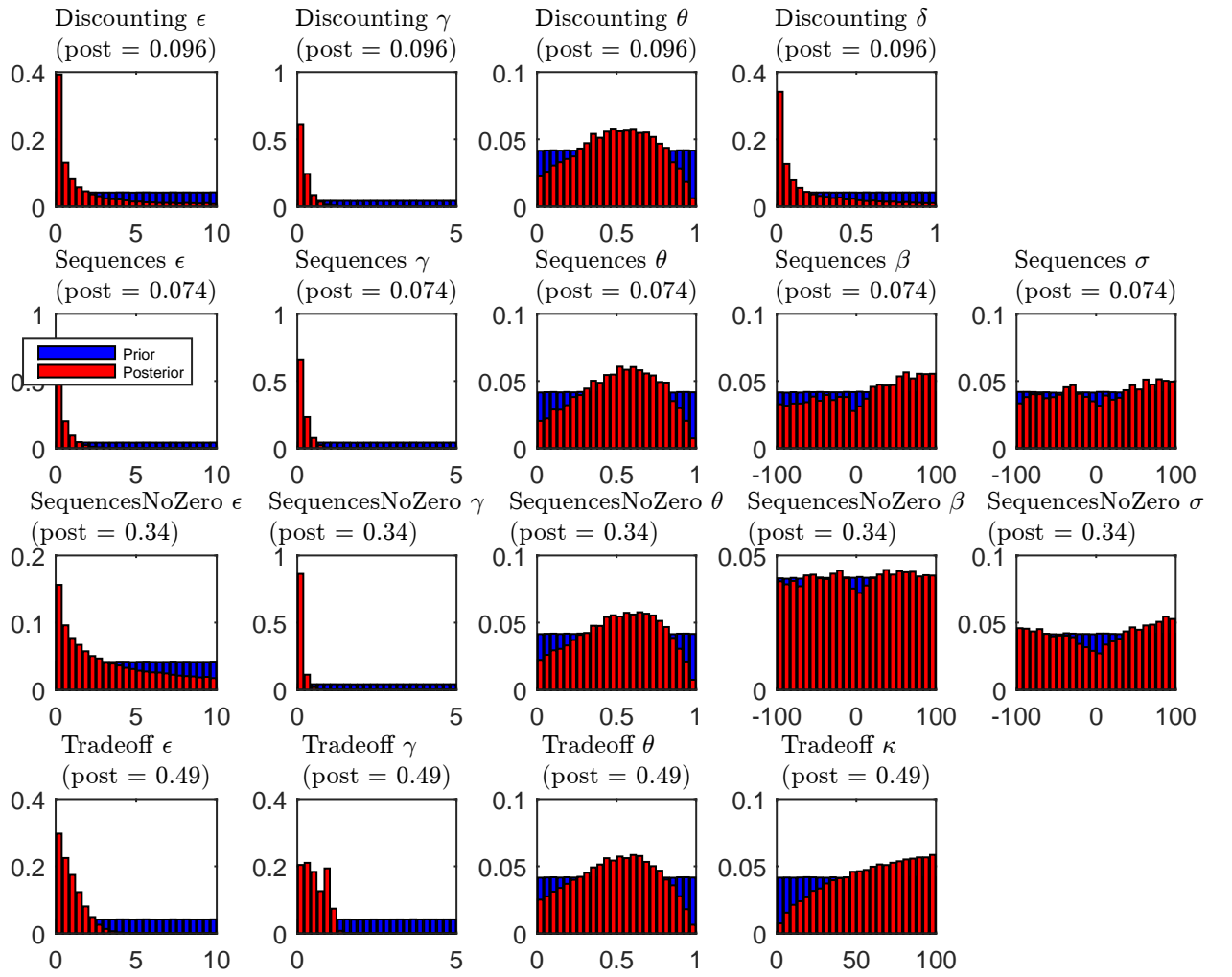

Supplement: Supplementary file 1 [file Scholten_Individuals.zip › plots/e29_p101_eg2_priors_and_posteriors.pdf]

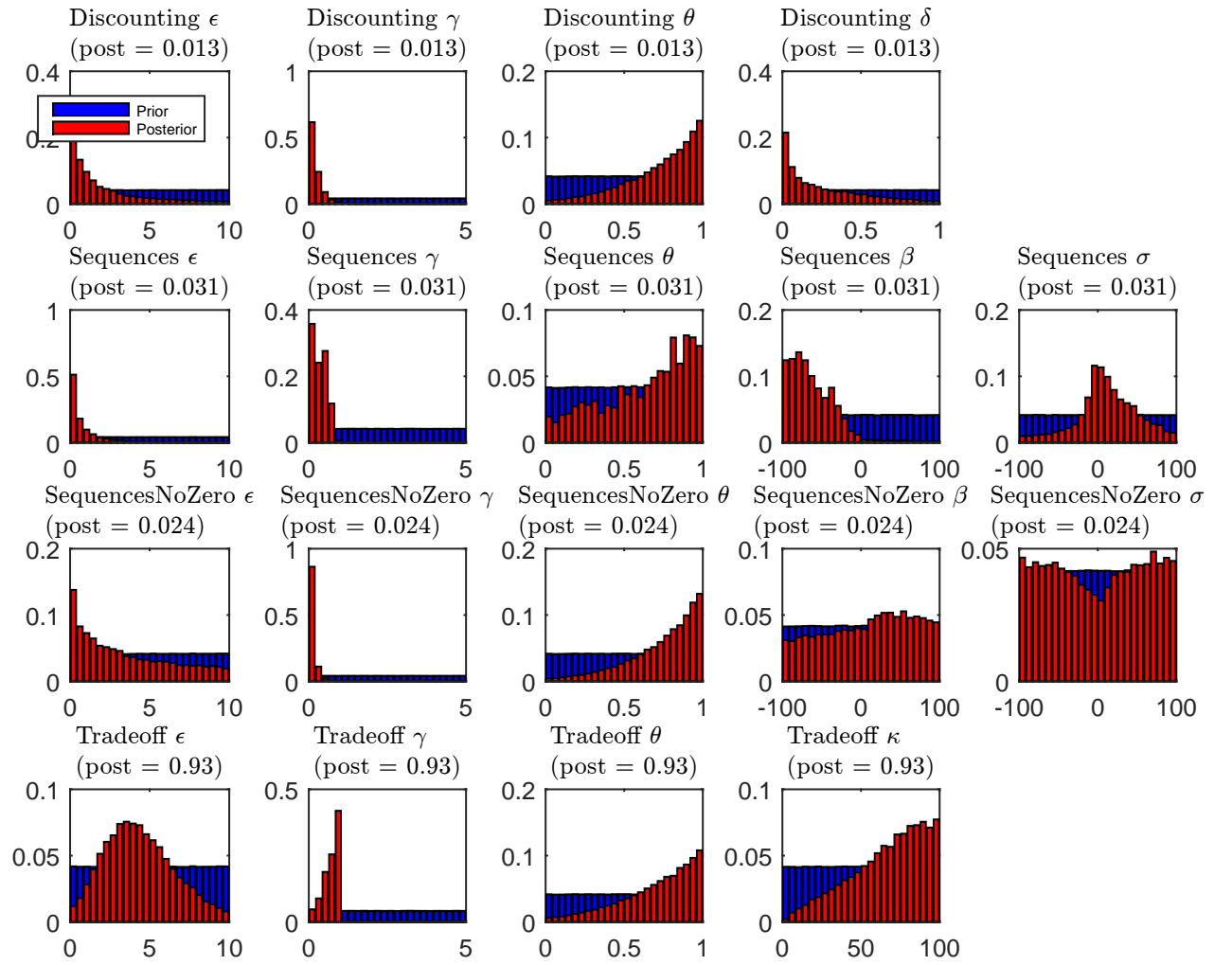

Supplement: Supplementary file 1 [file Scholten_Individuals.zip › plots/e29_p102_eg2_priors_and_posteriors.pdf]

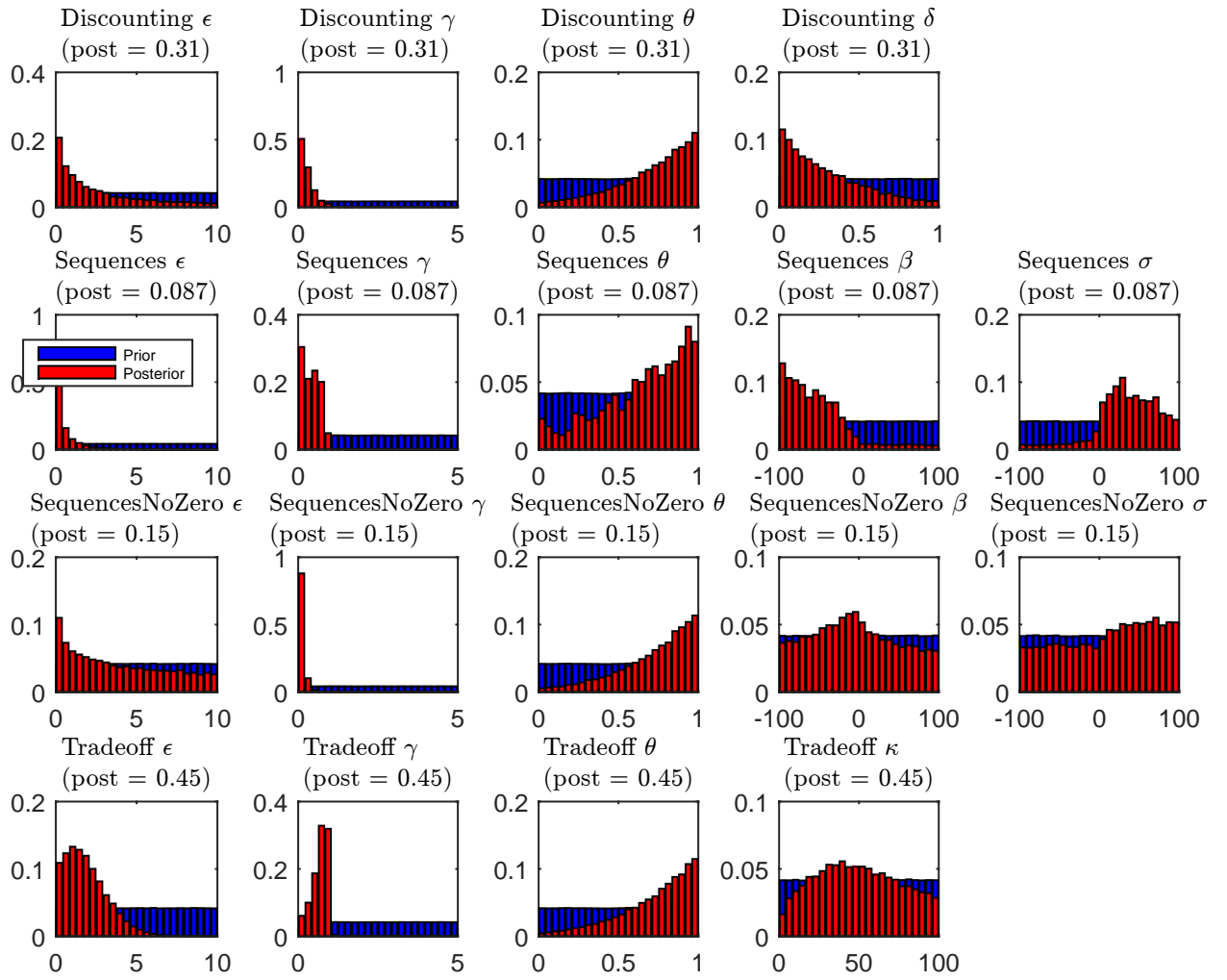

Supplement: Supplementary file 1 [file Scholten_Individuals.zip › plots/e29_p103_eg2_priors_and_posteriors.pdf]

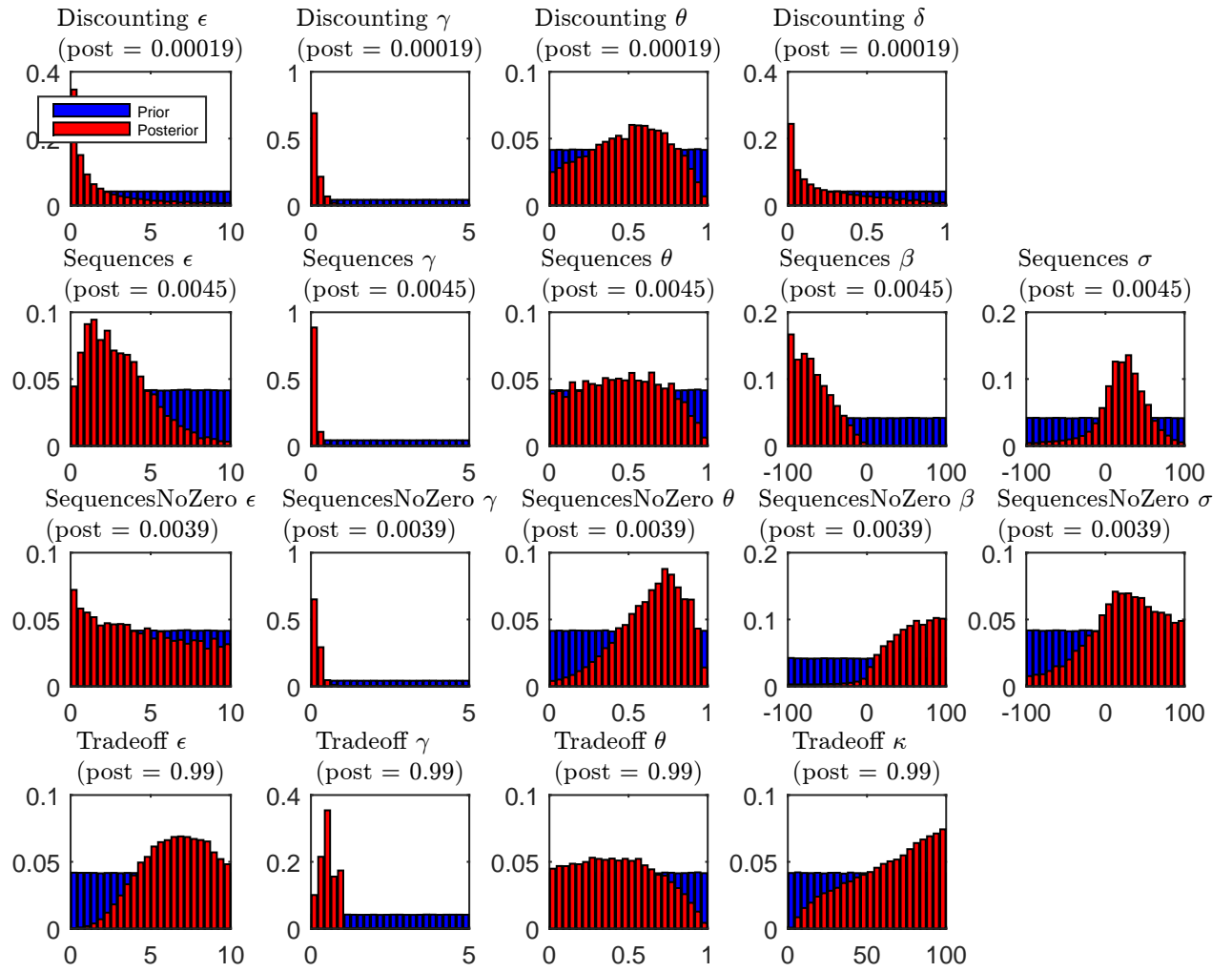

Supplement: Supplementary file 1 [file Scholten_Individuals.zip › plots/e29_p104_eg2_priors_and_posteriors.pdf]

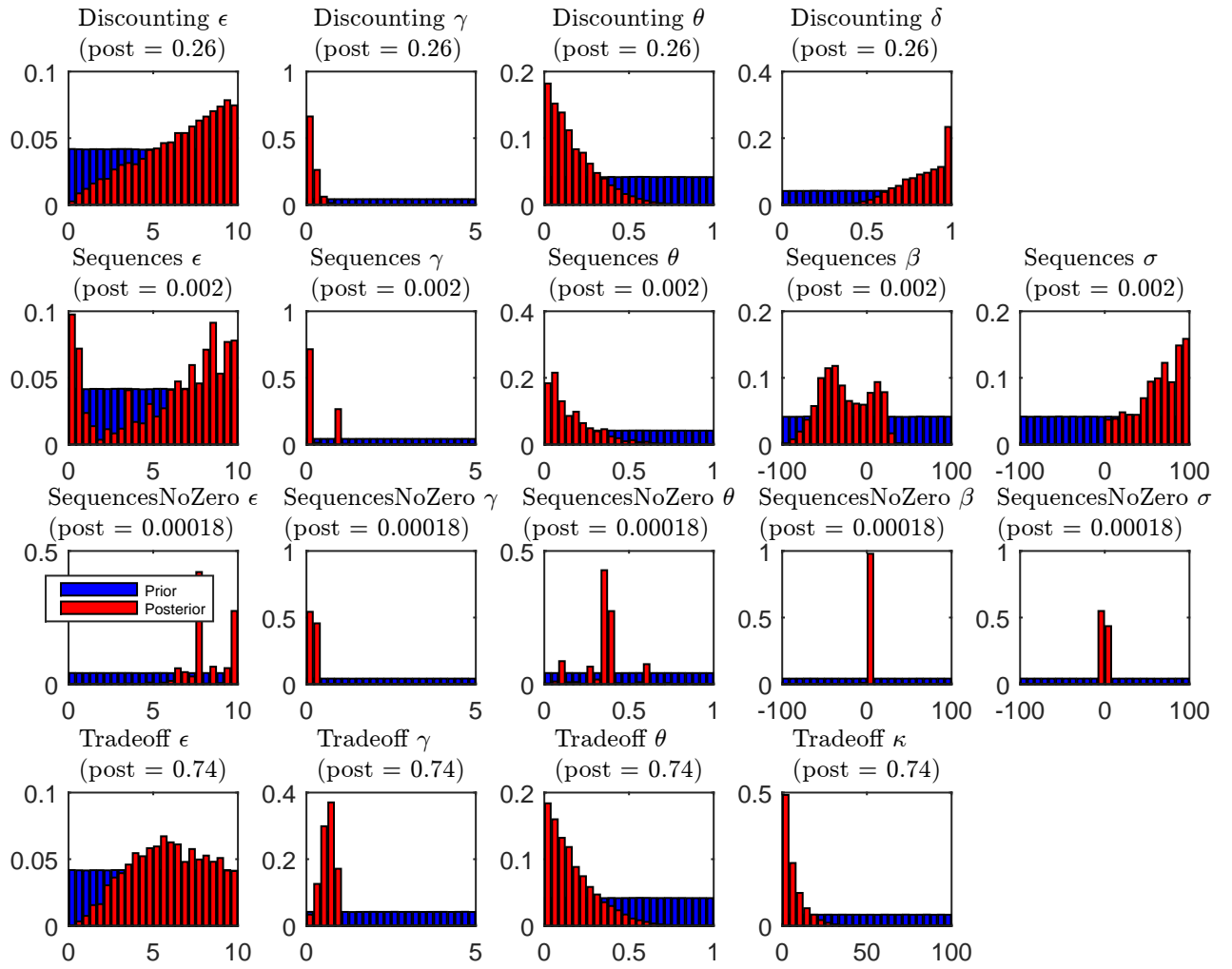

Supplement: Supplementary file 1 [file Scholten_Individuals.zip › plots/e29_p105_eg2_priors_and_posteriors.pdf]

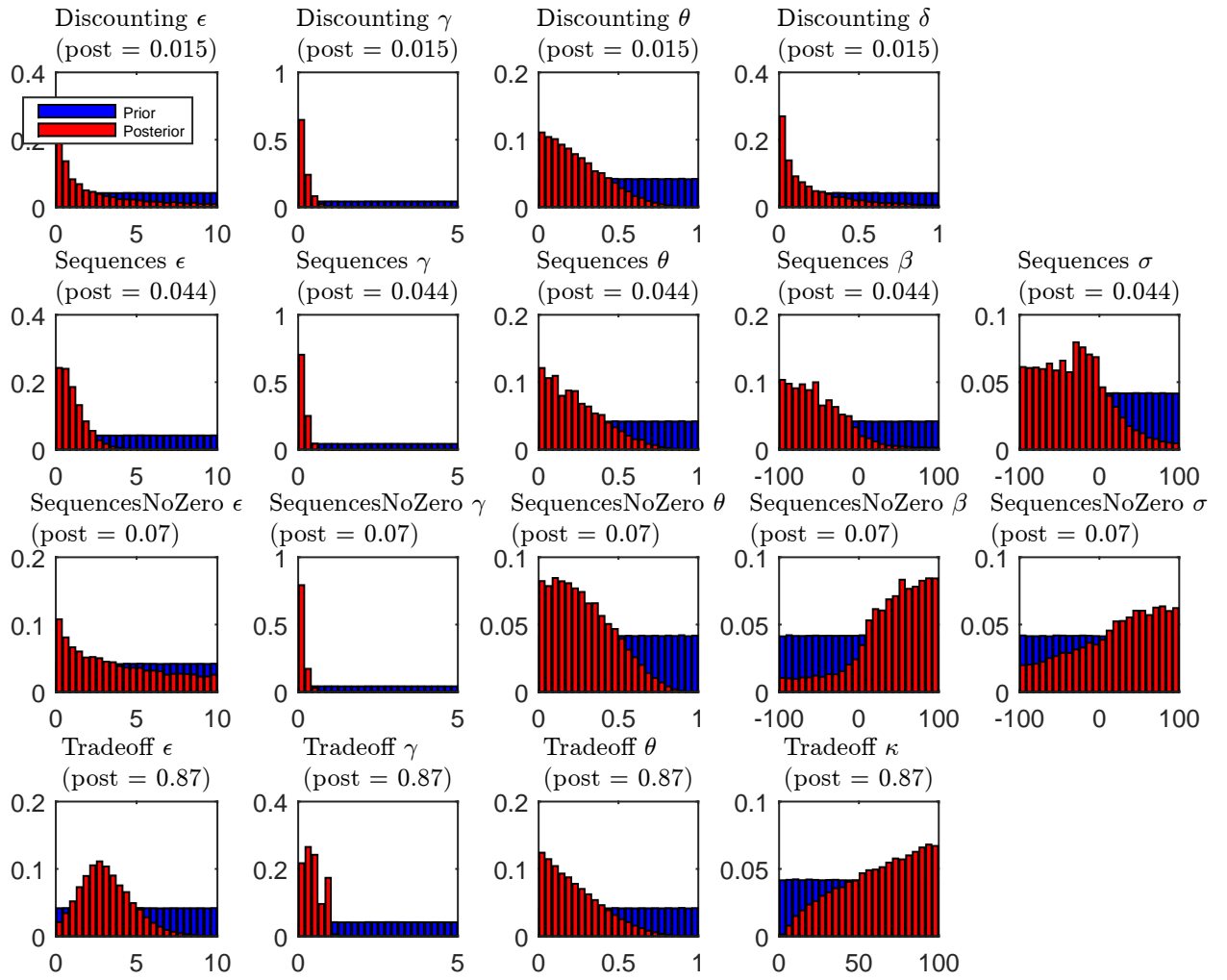

Supplement: Supplementary file 1 [file Scholten_Individuals.zip › plots/e29_p106_eg2_priors_and_posteriors.pdf]

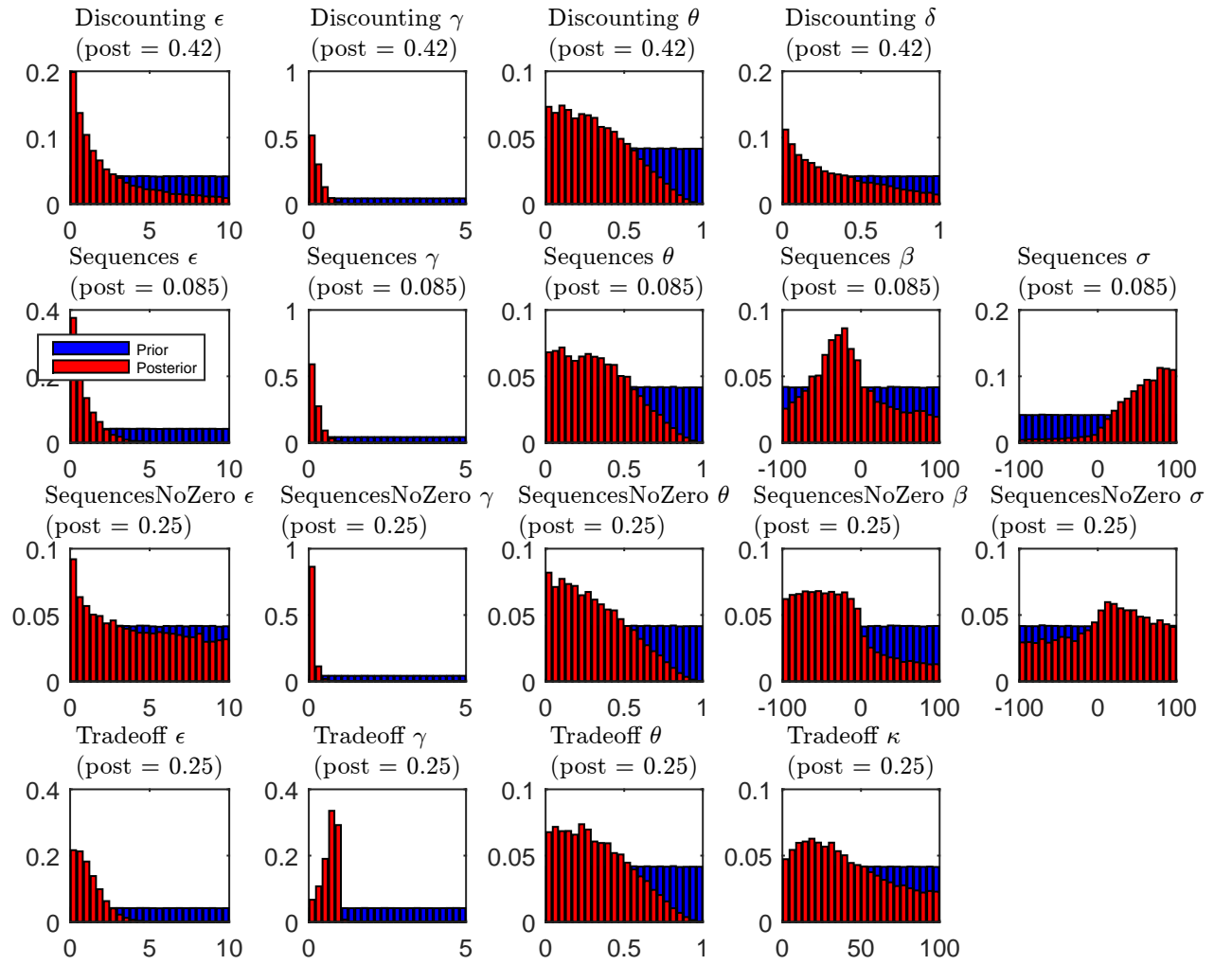

Supplement: Supplementary file 1 [file Scholten_Individuals.zip › plots/e29_p107_eg2_priors_and_posteriors.pdf]

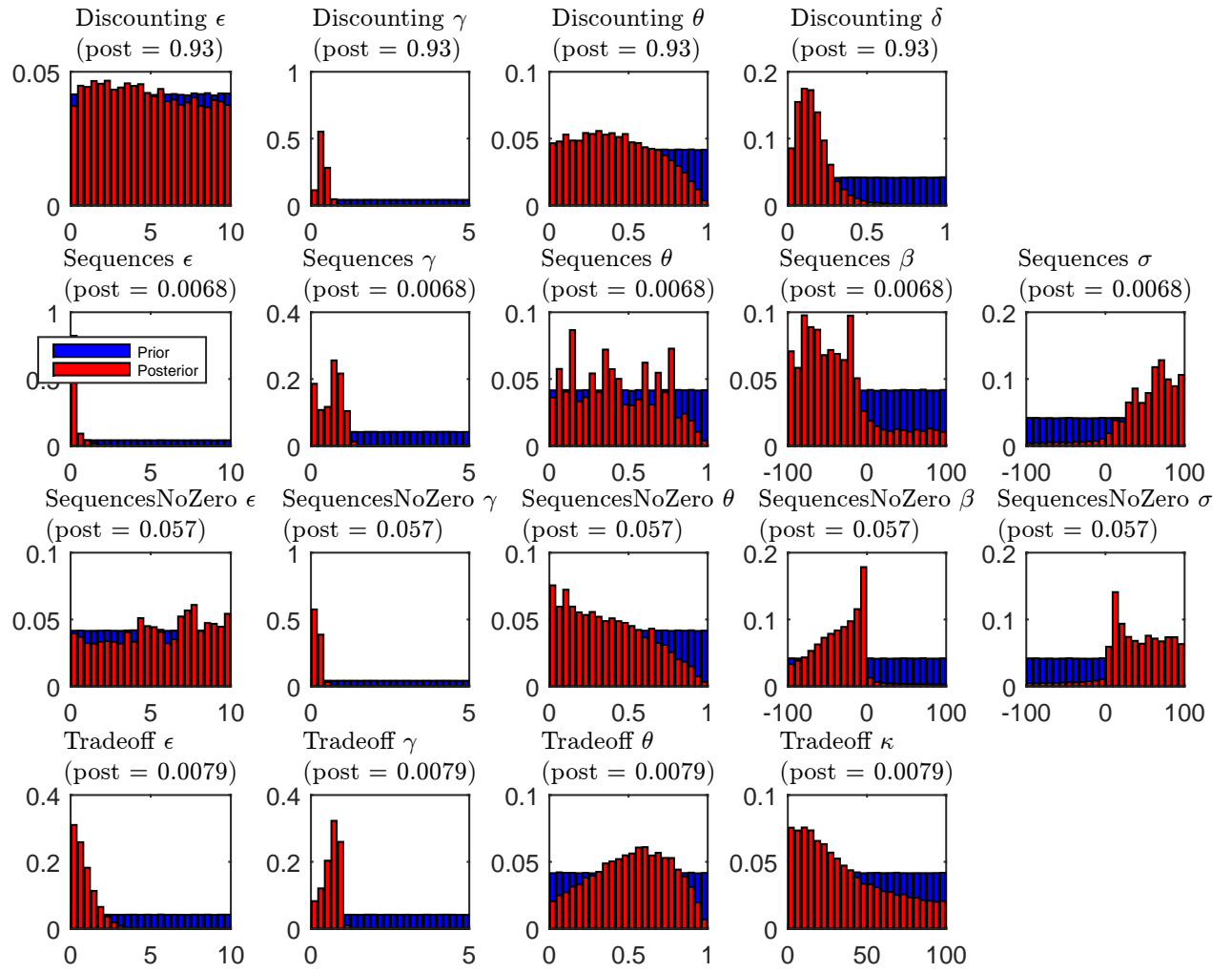

Supplement: Supplementary file 1 [file Scholten_Individuals.zip › plots/e29_p108_eg2_priors_and_posteriors.pdf]

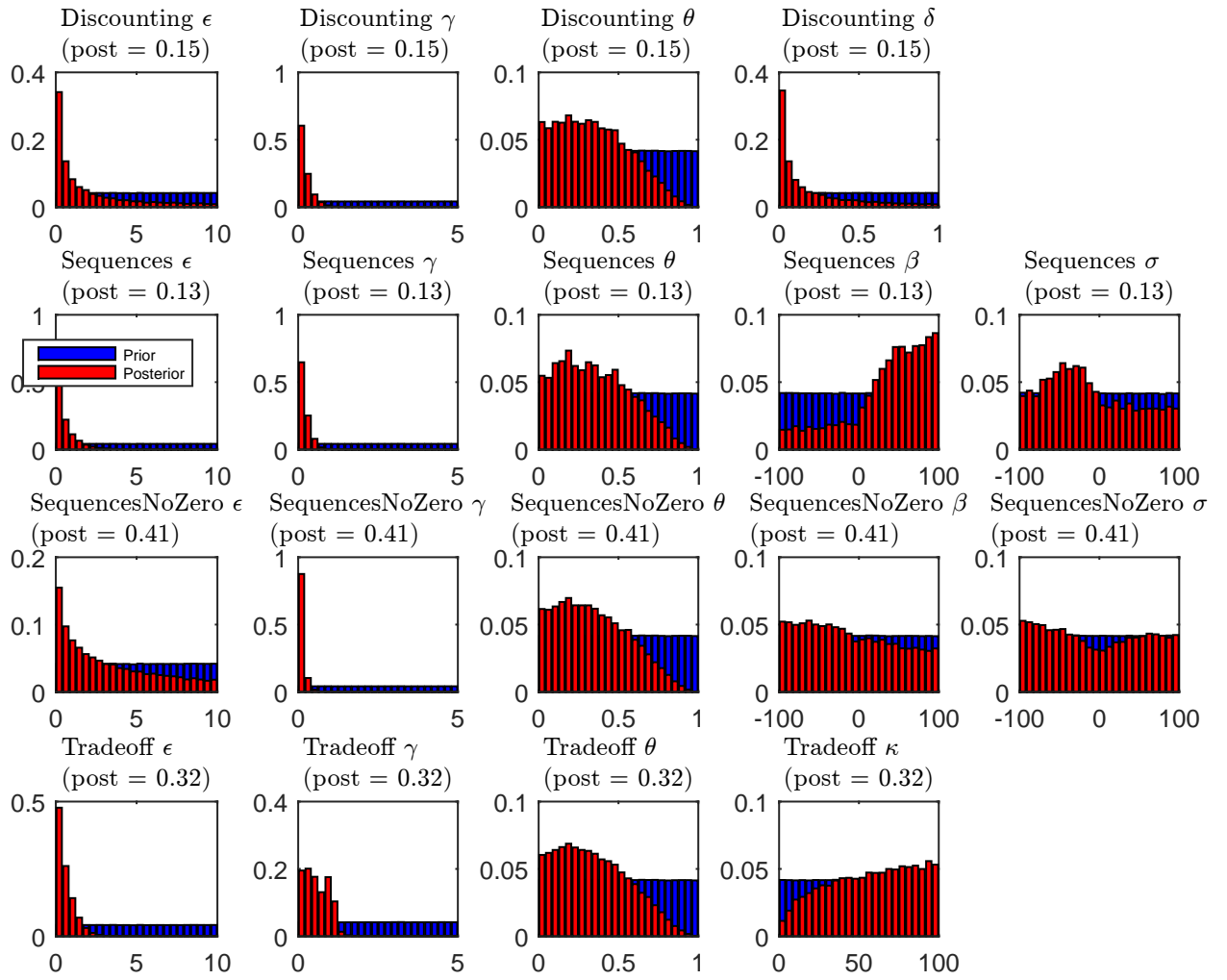

Supplement: Supplementary file 1 [file Scholten_Individuals.zip › plots/e29_p109_eg2_priors_and_posteriors.pdf]

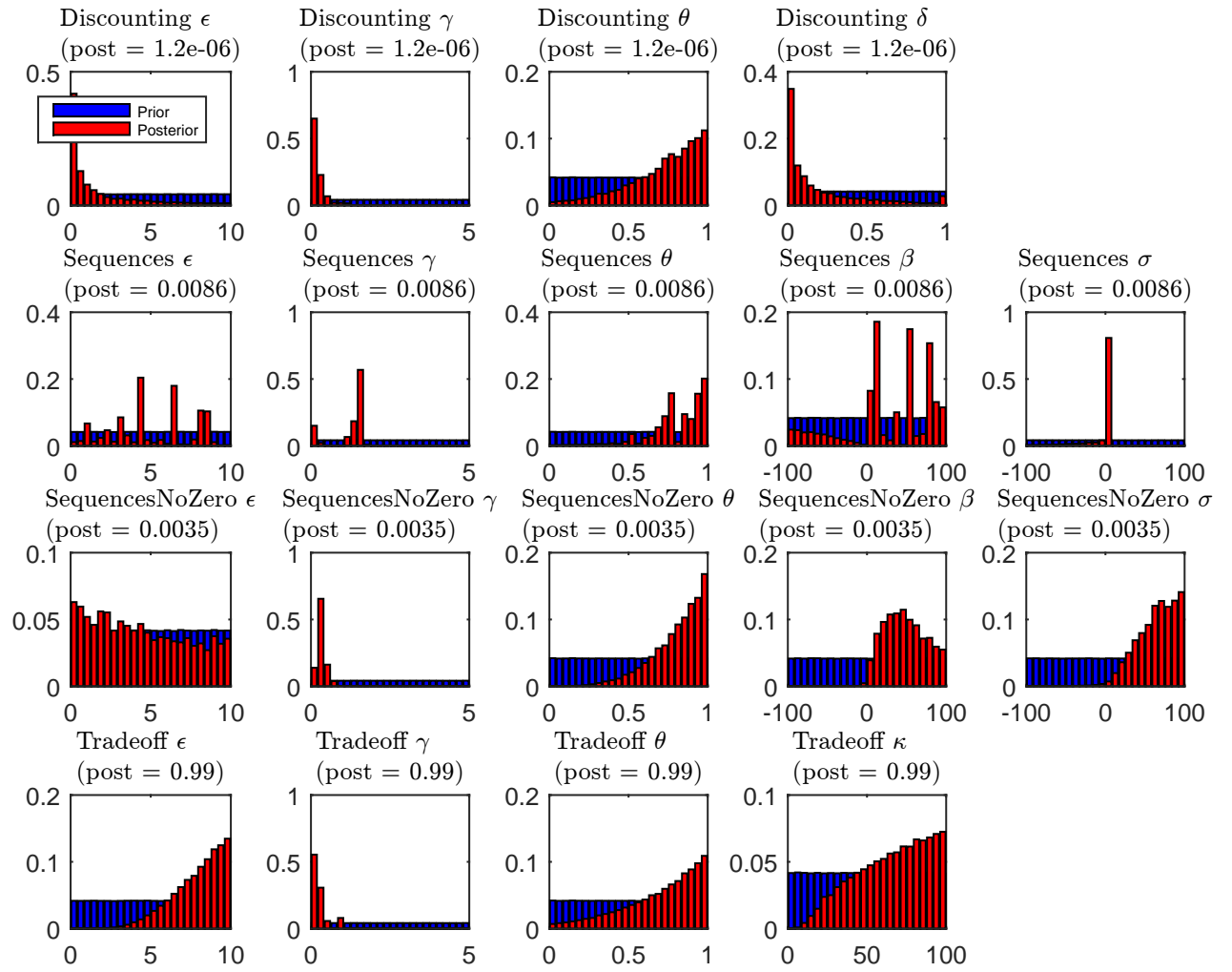

Supplement: Supplementary file 1 [file Scholten_Individuals.zip › plots/e29_p11_eg2_priors_and_posteriors.pdf]

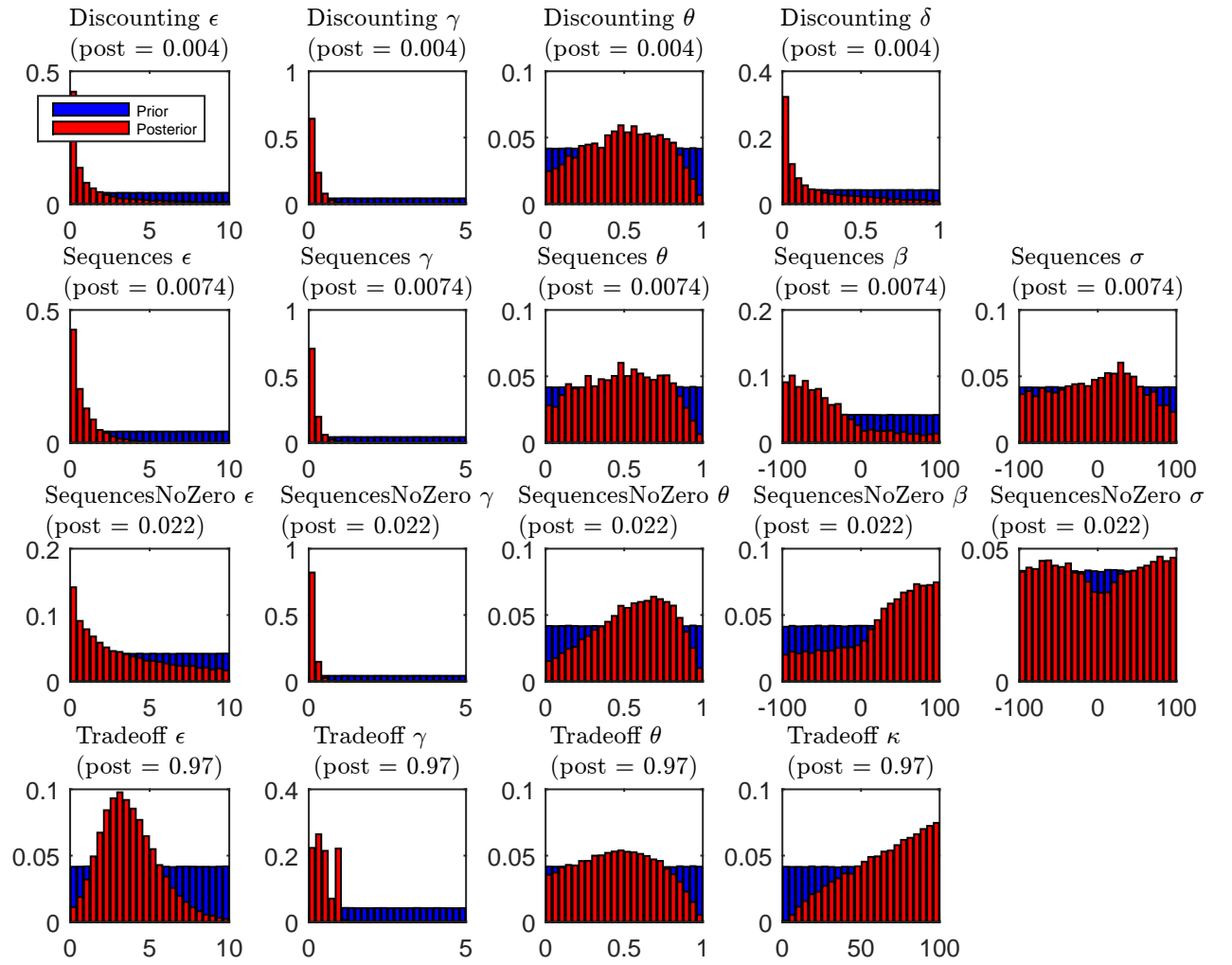

Supplement: Supplementary file 1 [file Scholten_Individuals.zip › plots/e29_p110_eg2_priors_and_posteriors.pdf]

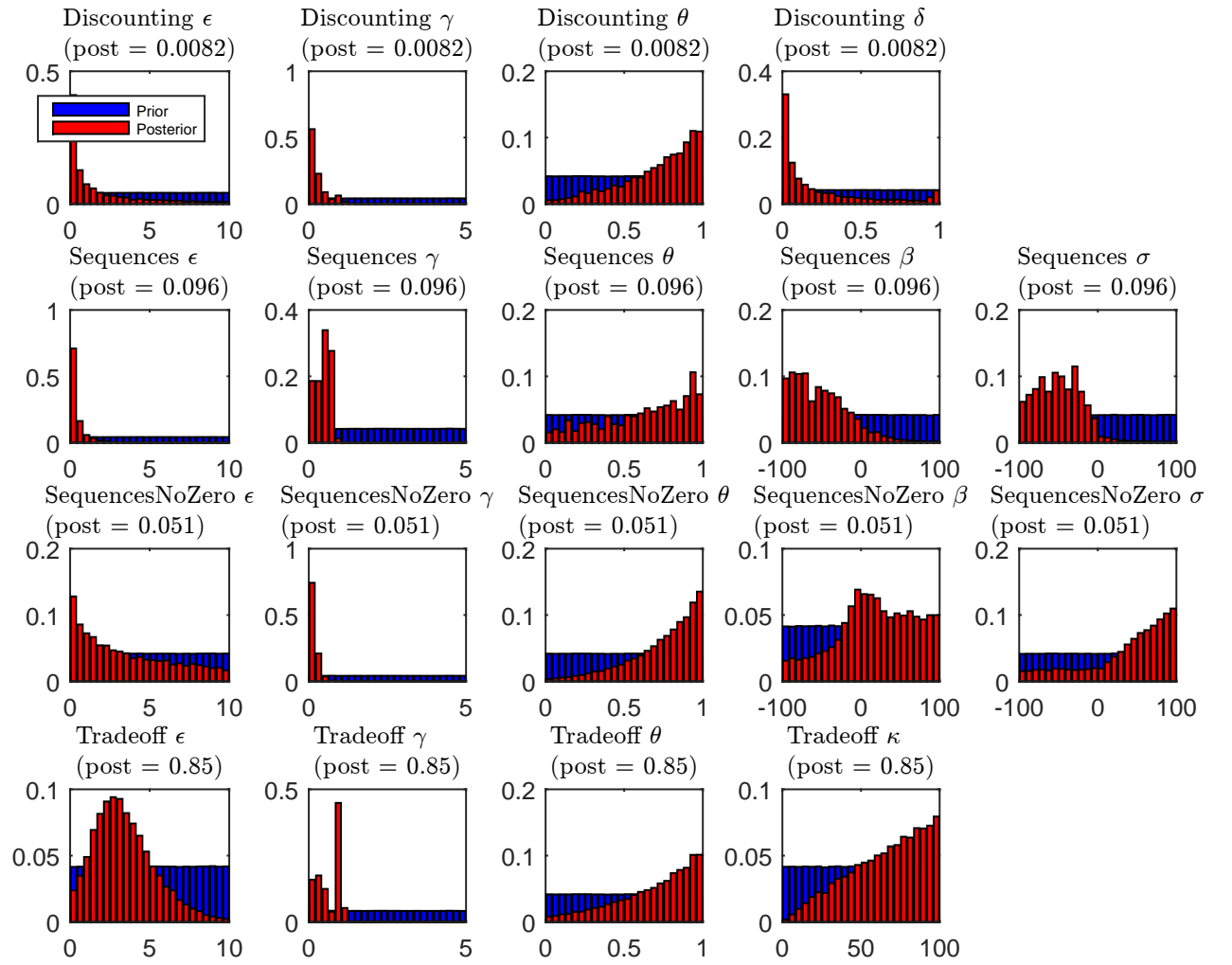

Supplement: Supplementary file 1 [file Scholten_Individuals.zip › plots/e29_p113_eg2_priors_and_posteriors.pdf]

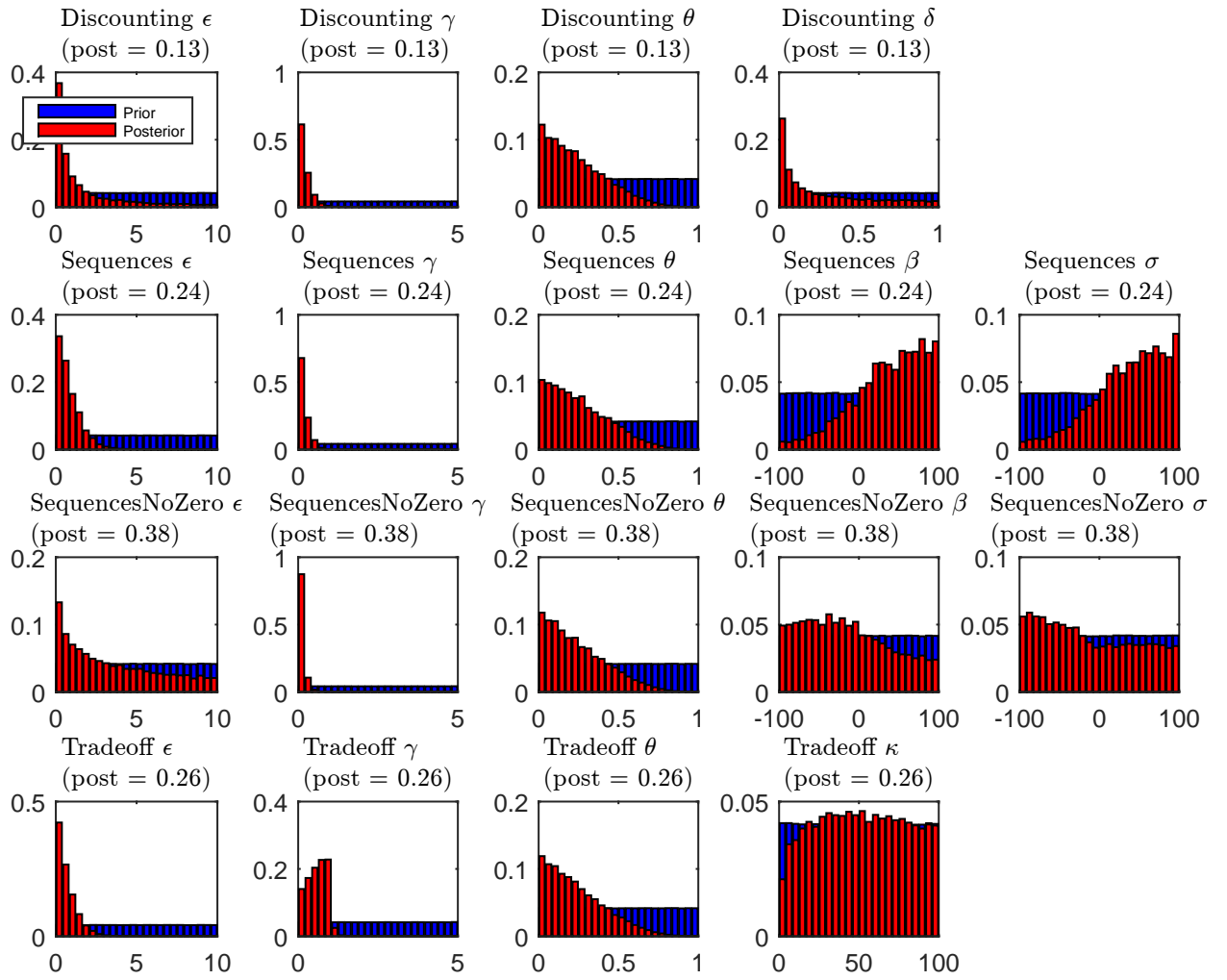

Supplement: Supplementary file 1 [file Scholten_Individuals.zip › plots/e29_p114_eg2_priors_and_posteriors.pdf]

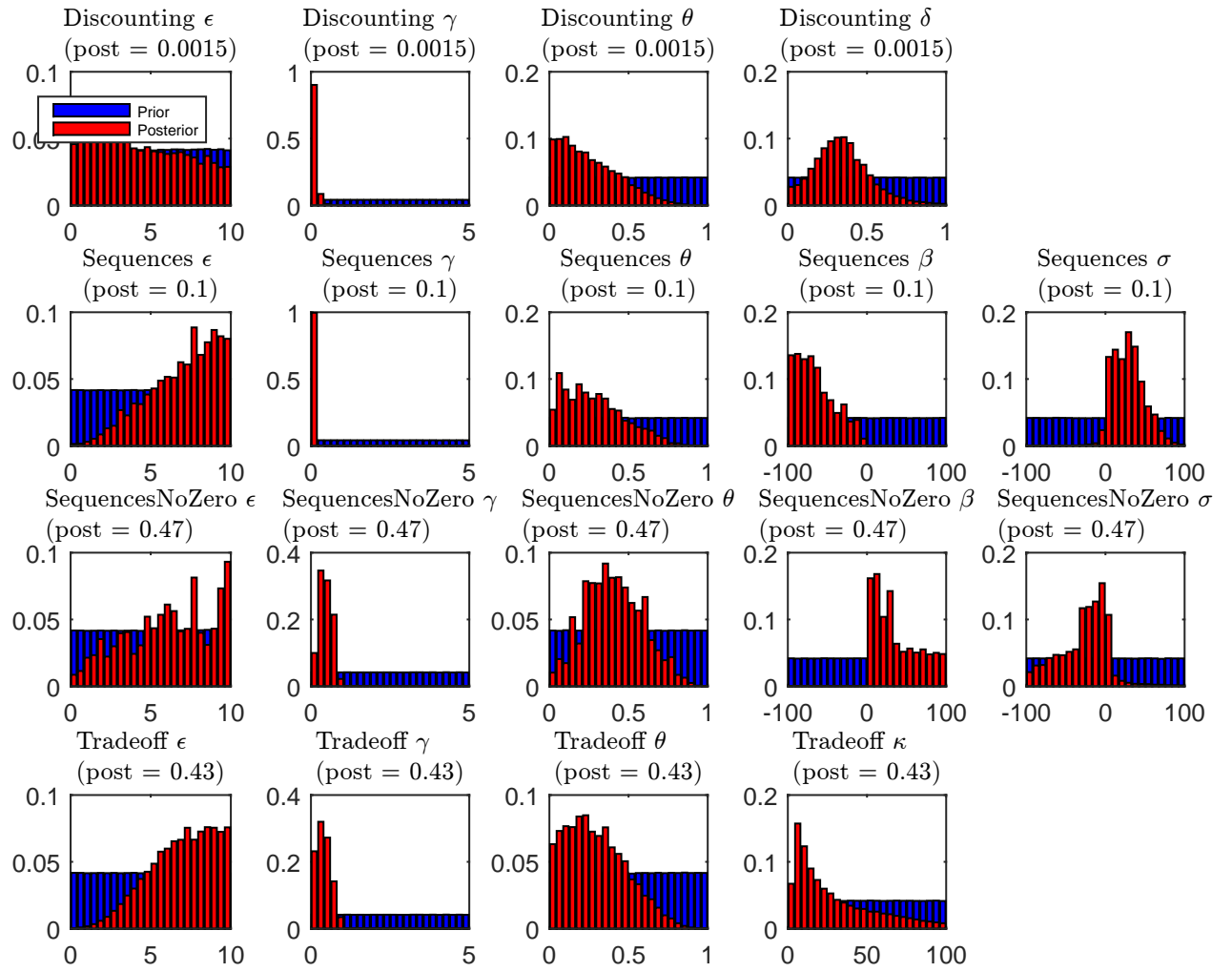

Supplement: Supplementary file 1 [file Scholten_Individuals.zip › plots/e29_p115_eg2_priors_and_posteriors.pdf]

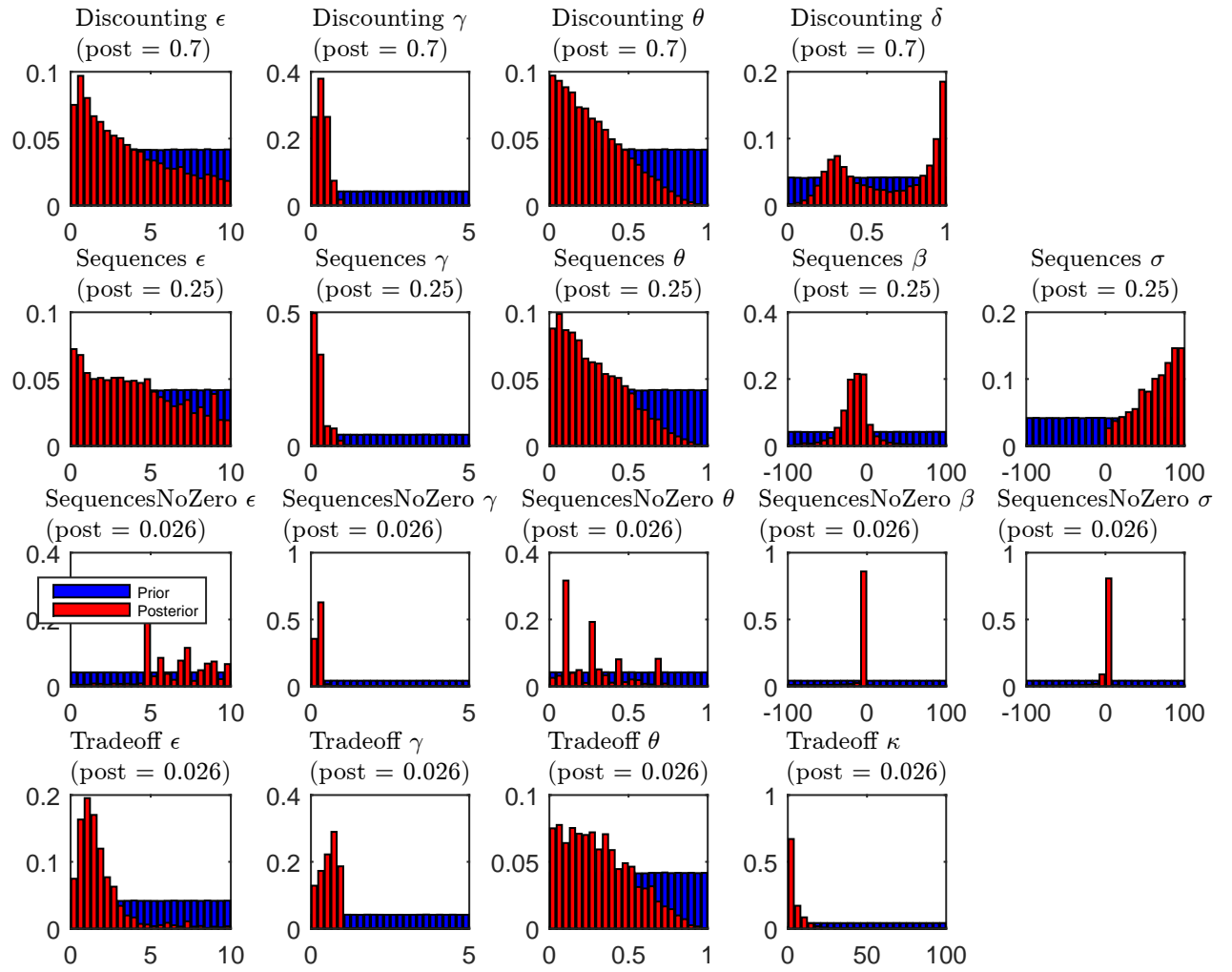

Supplement: Supplementary file 1 [file Scholten_Individuals.zip › plots/e29_p116_eg2_priors_and_posteriors.pdf]

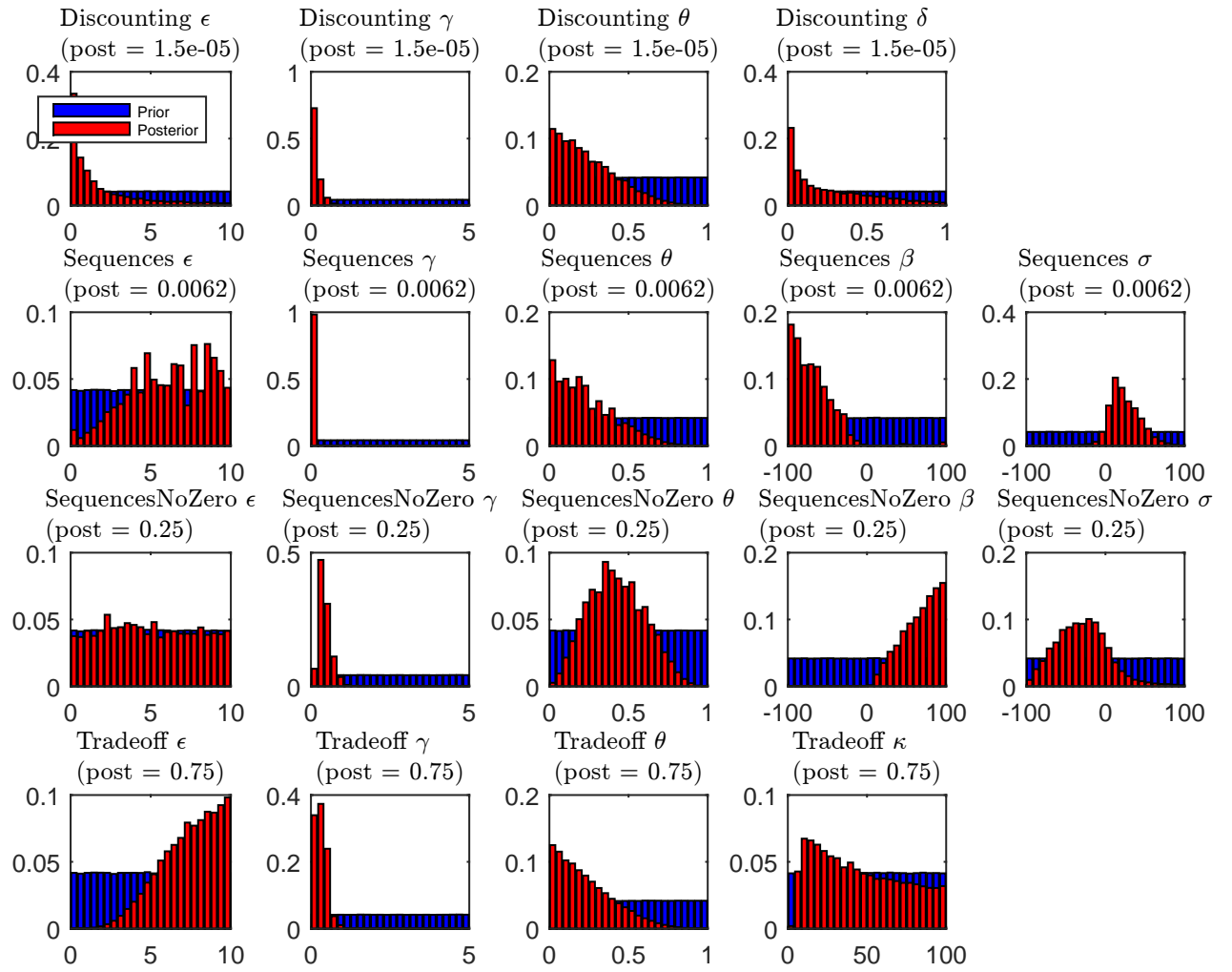

Supplement: Supplementary file 1 [file Scholten_Individuals.zip › plots/e29_p117_eg2_priors_and_posteriors.pdf]

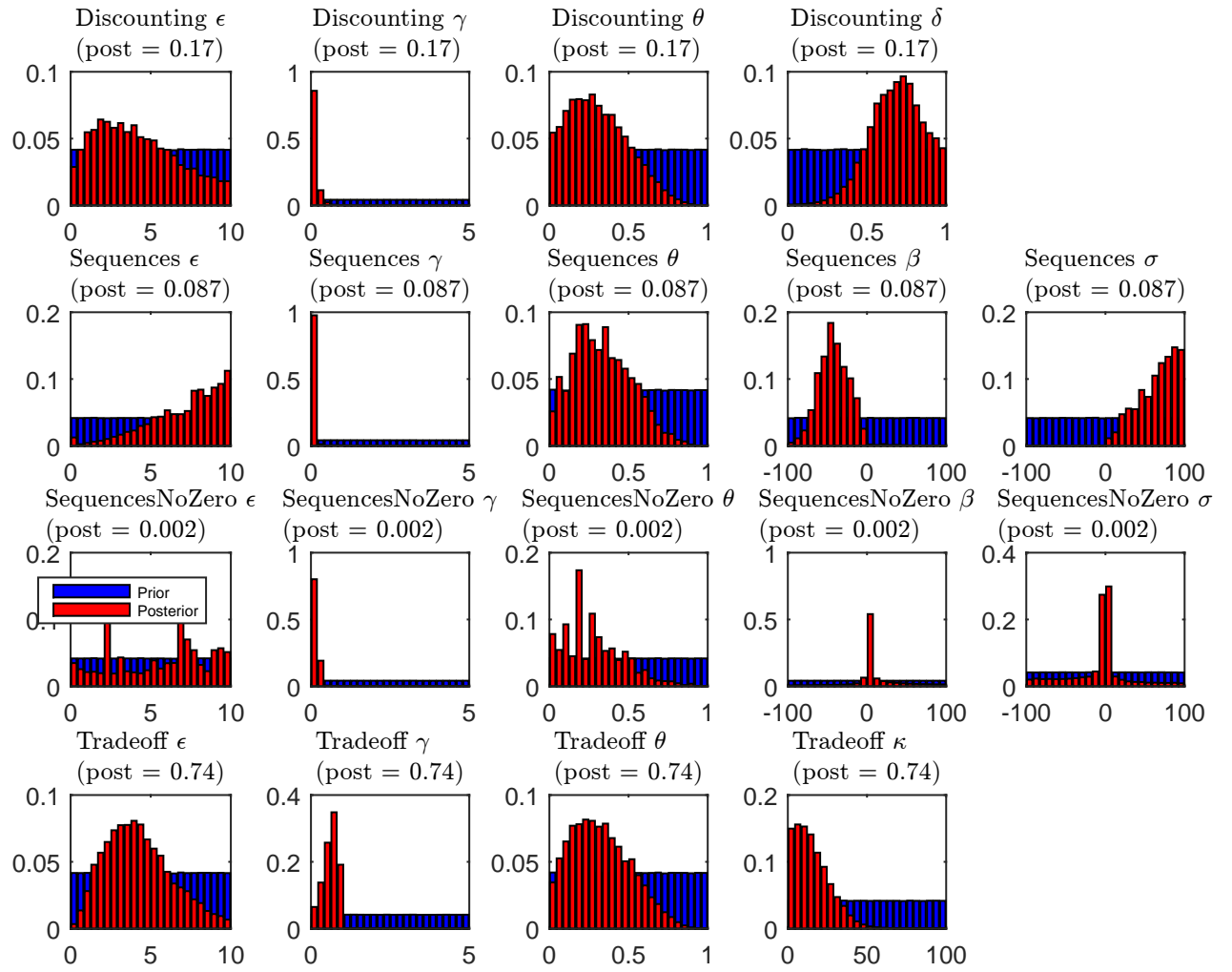

Supplement: Supplementary file 1 [file Scholten_Individuals.zip › plots/e29_p118_eg2_priors_and_posteriors.pdf]

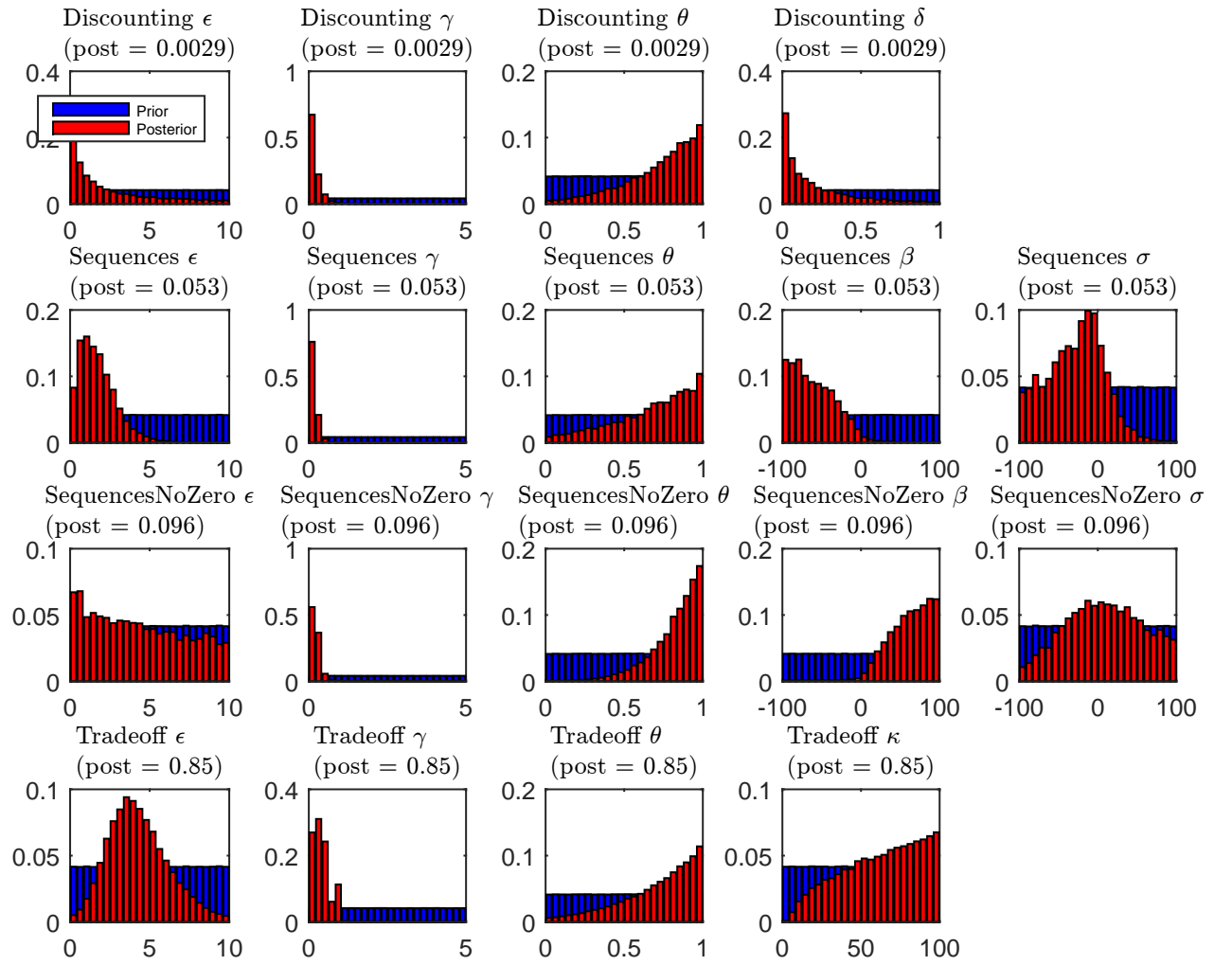

Supplement: Supplementary file 1 [file Scholten_Individuals.zip › plots/e29_p12_eg2_priors_and_posteriors.pdf]

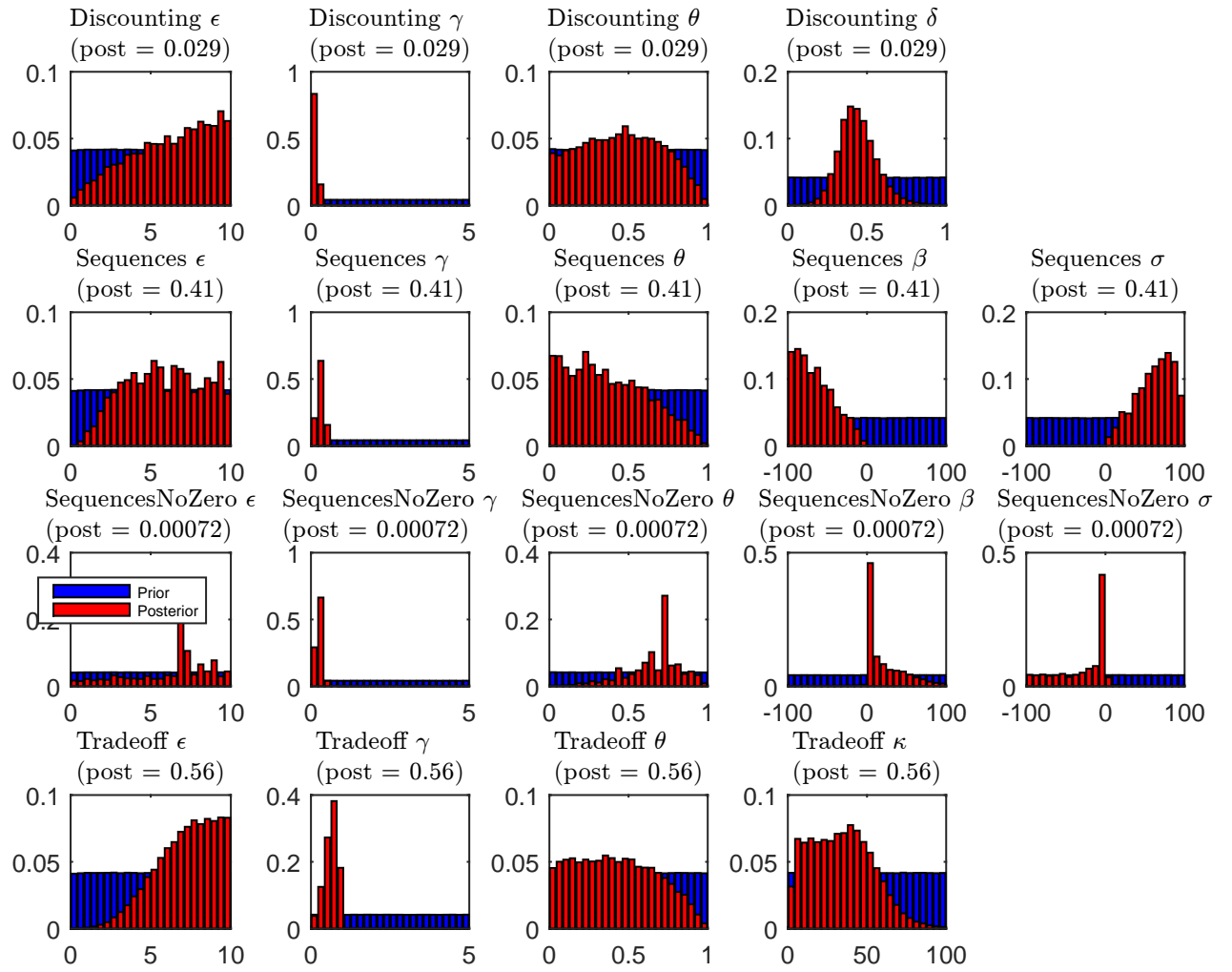

Supplement: Supplementary file 1 [file Scholten_Individuals.zip › plots/e29_p120_eg2_priors_and_posteriors.pdf]

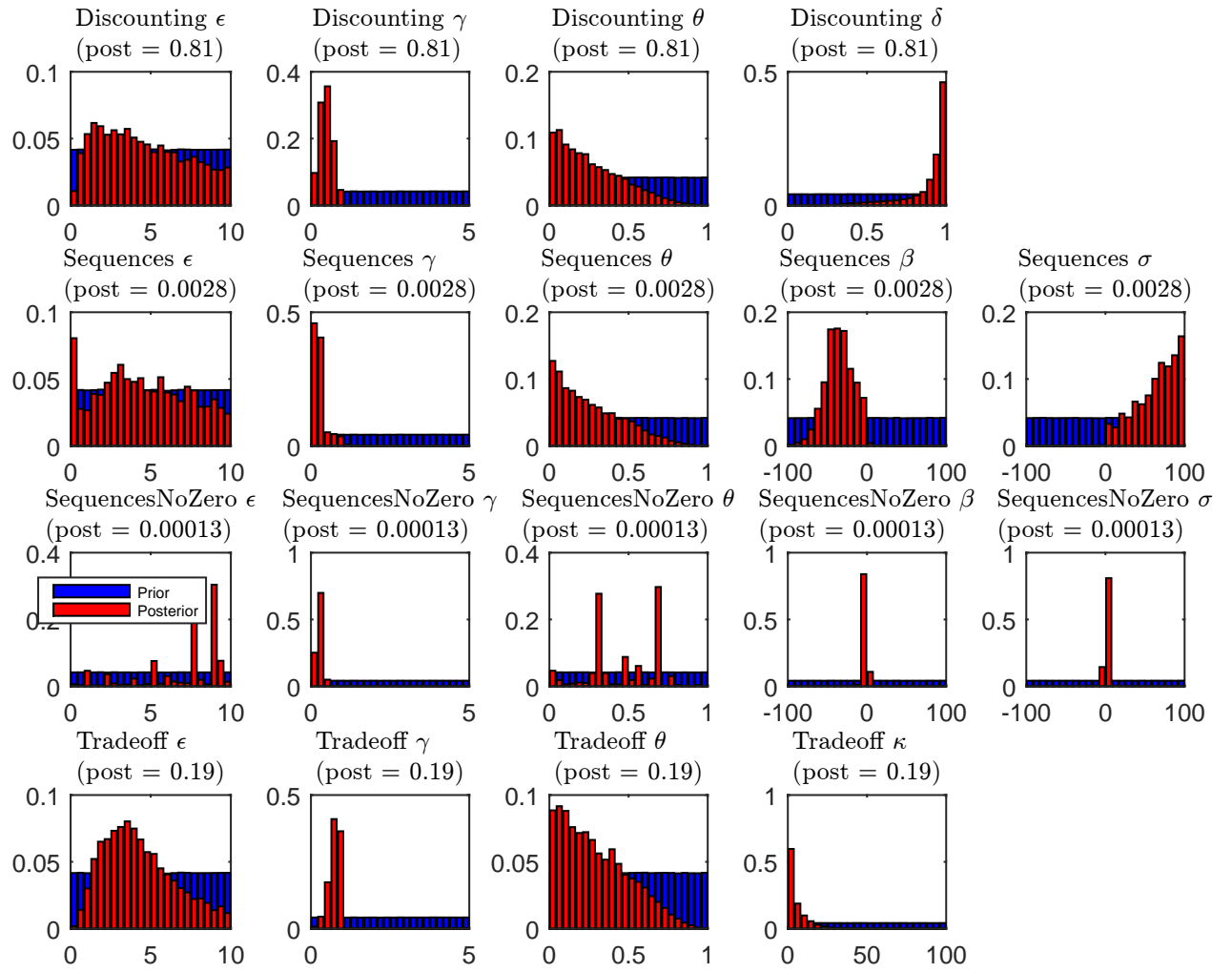

Supplement: Supplementary file 1 [file Scholten_Individuals.zip › plots/e29_p121_eg2_priors_and_posteriors.pdf]

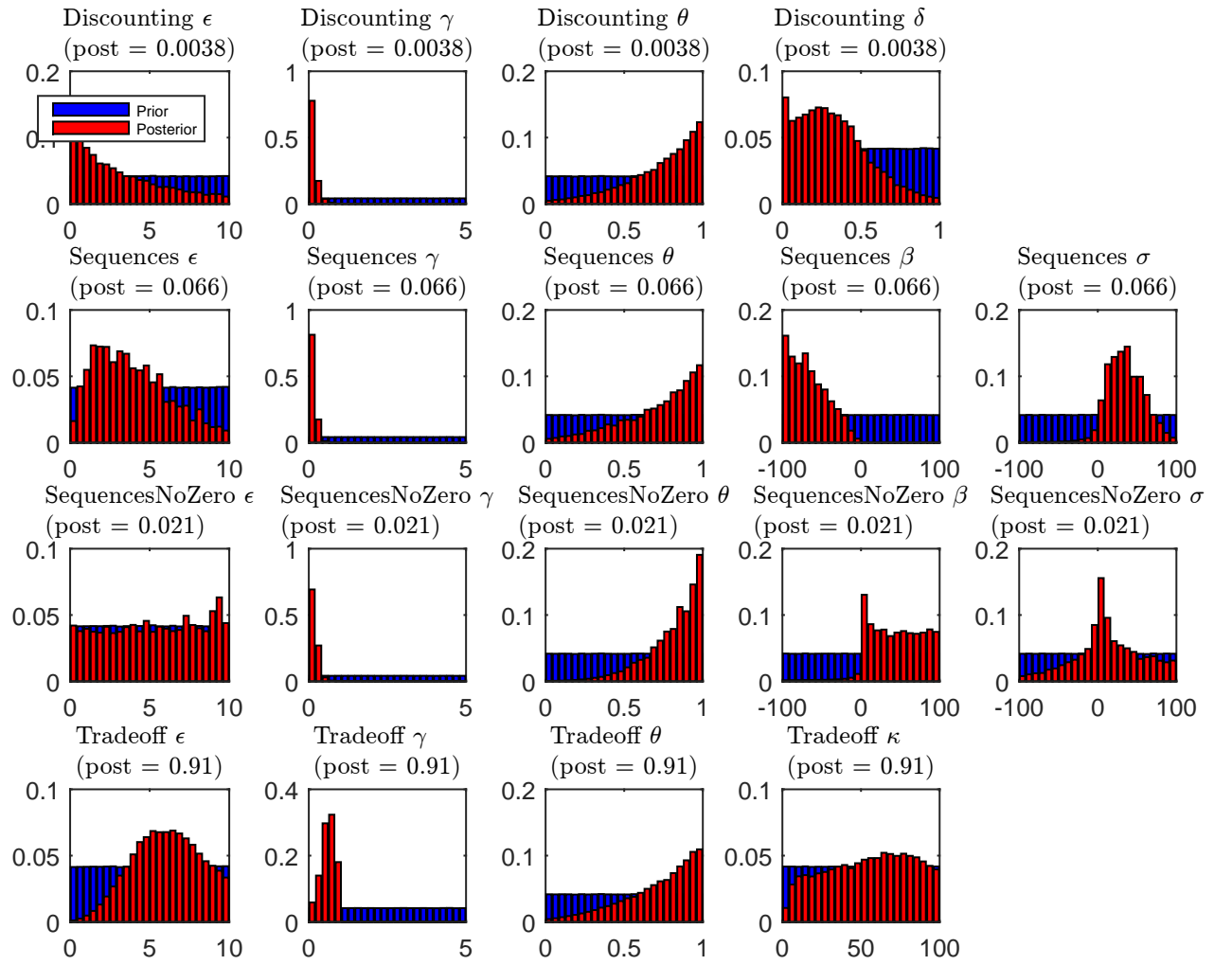

Supplement: Supplementary file 1 [file Scholten_Individuals.zip › plots/e29_p122_eg2_priors_and_posteriors.pdf]

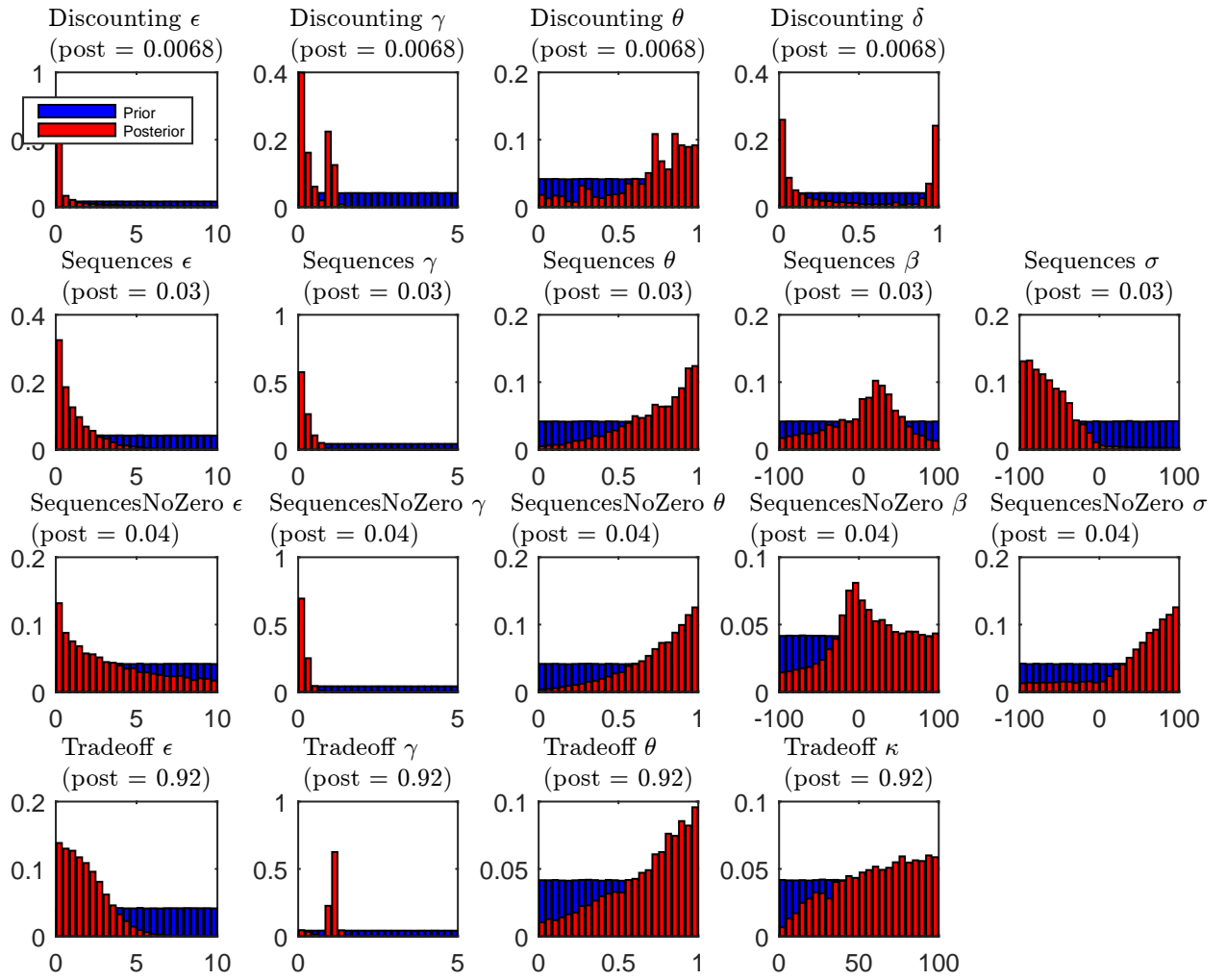

Supplement: Supplementary file 1 [file Scholten_Individuals.zip › plots/e29_p123_eg2_priors_and_posteriors.pdf]

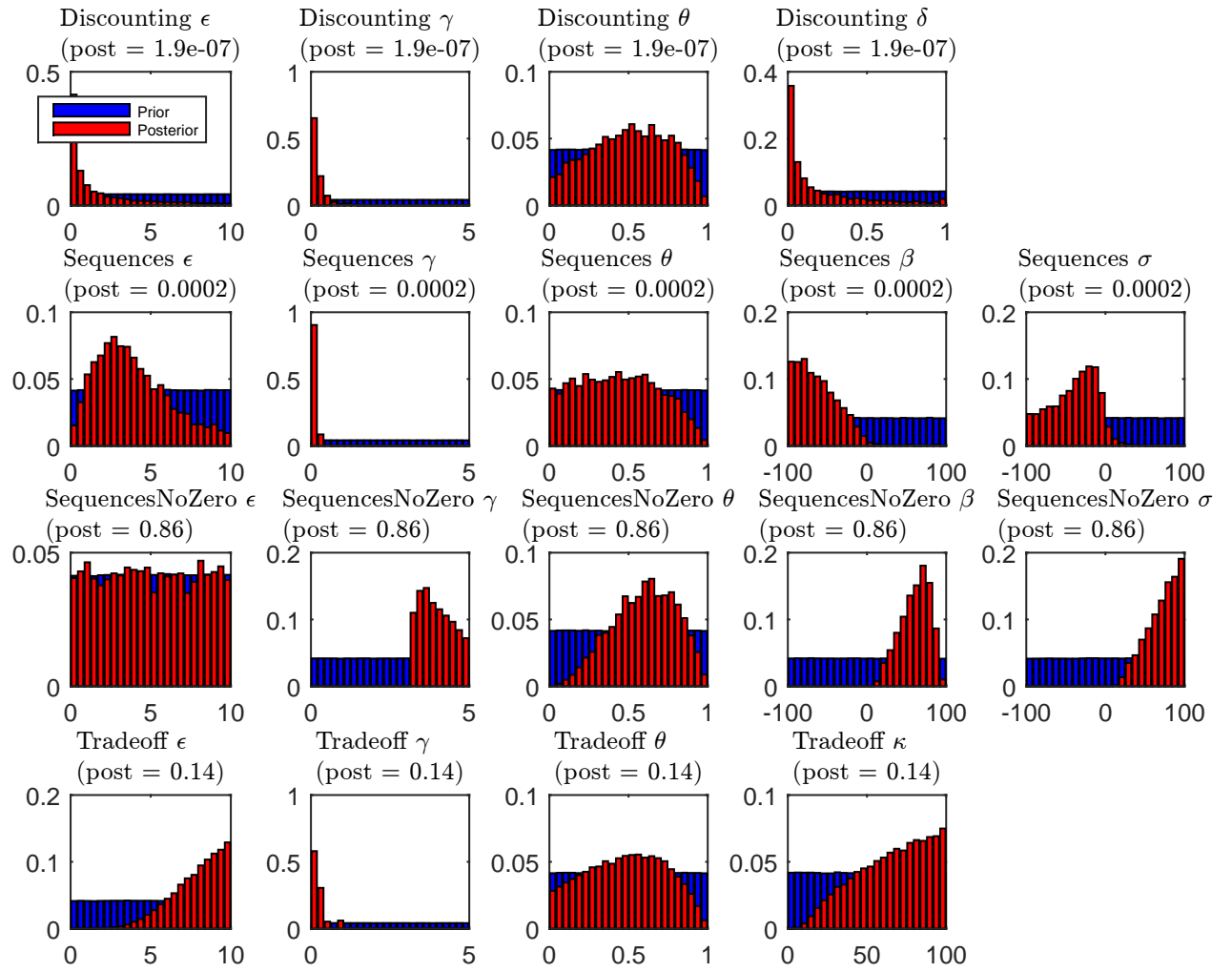

Supplement: Supplementary file 1 [file Scholten_Individuals.zip › plots/e29_p124_eg2_priors_and_posteriors.pdf]

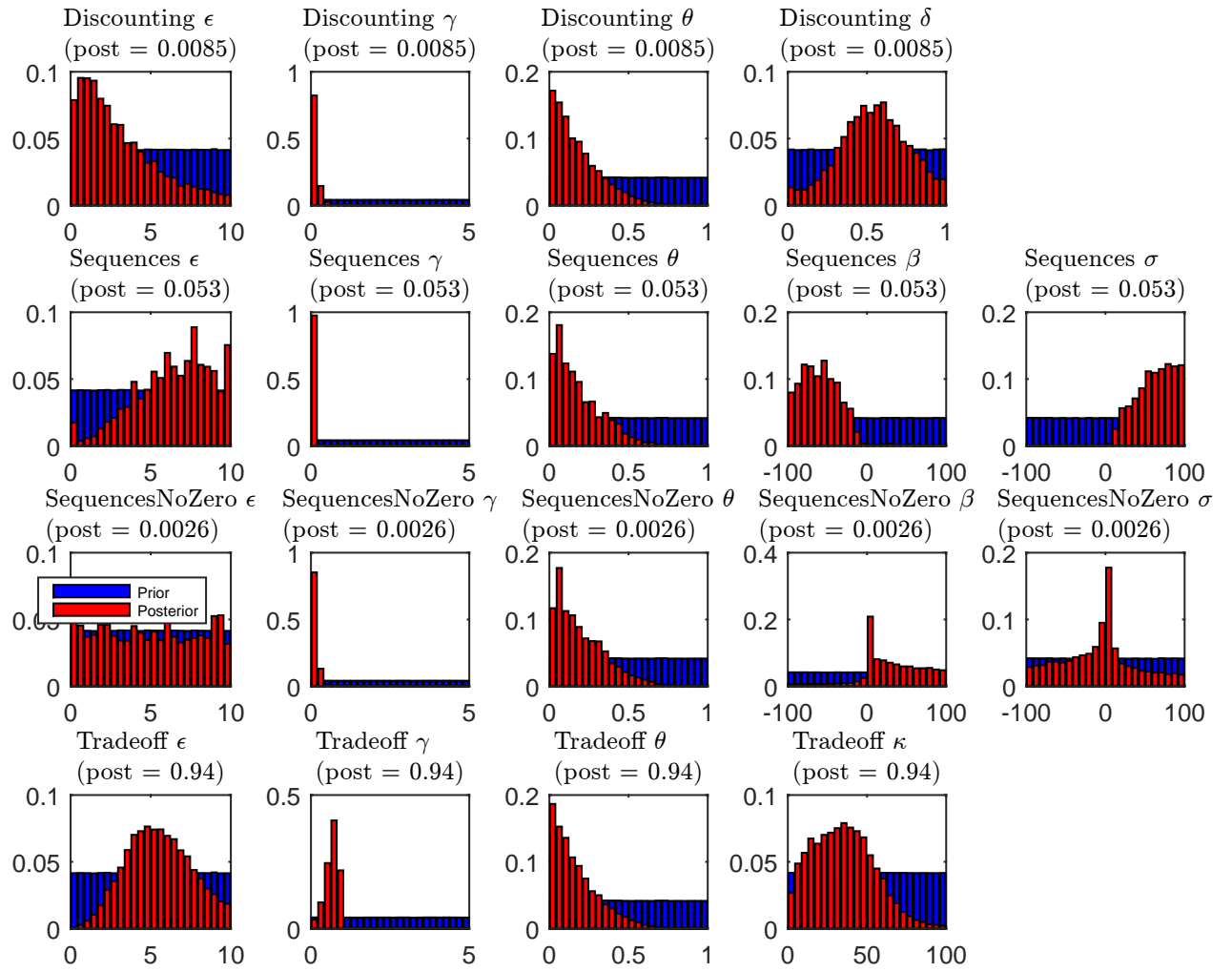

Supplement: Supplementary file 1 [file Scholten_Individuals.zip › plots/e29_p125_eg2_priors_and_posteriors.pdf]

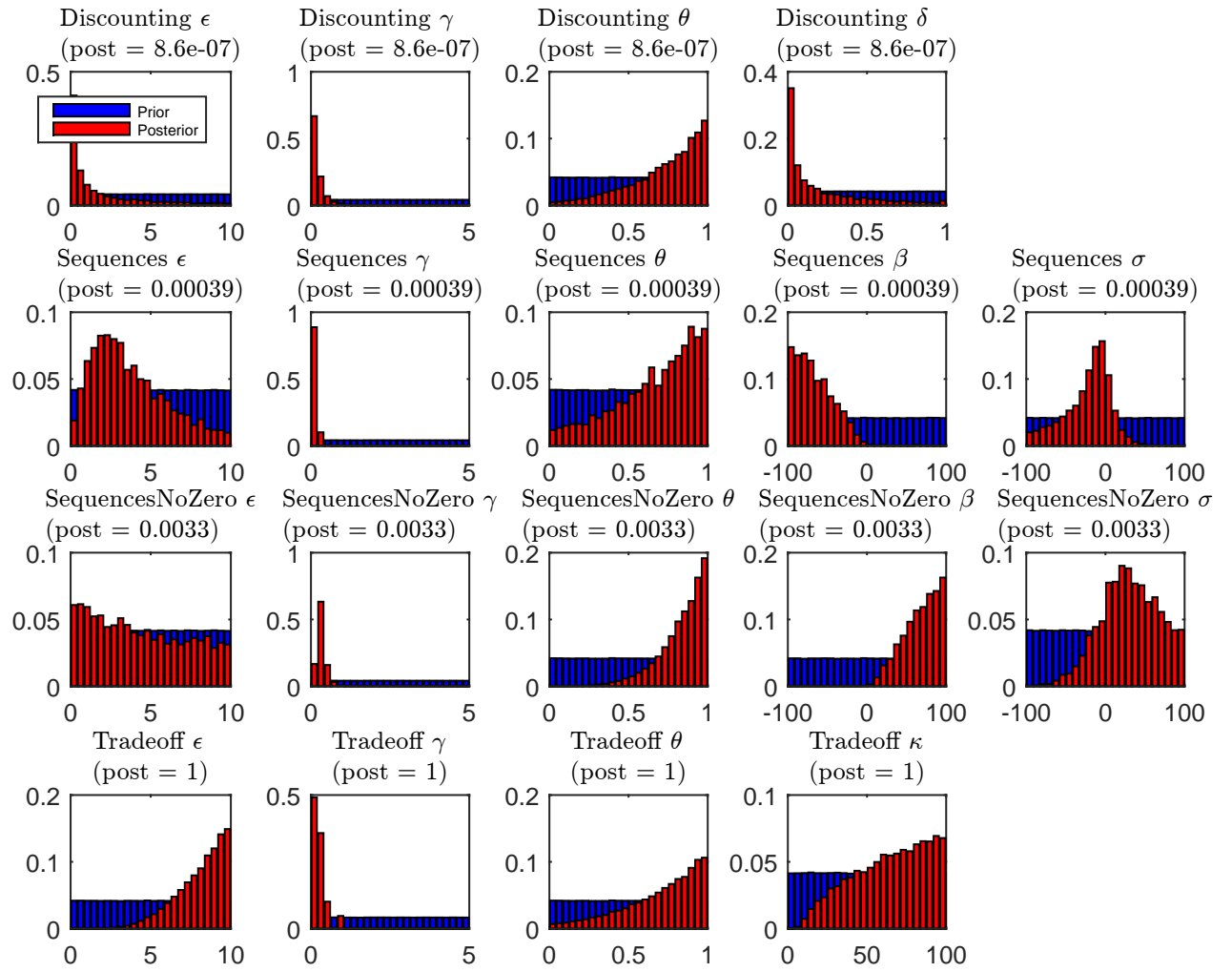

Supplement: Supplementary file 1 [file Scholten_Individuals.zip › plots/e29_p126_eg2_priors_and_posteriors.pdf]

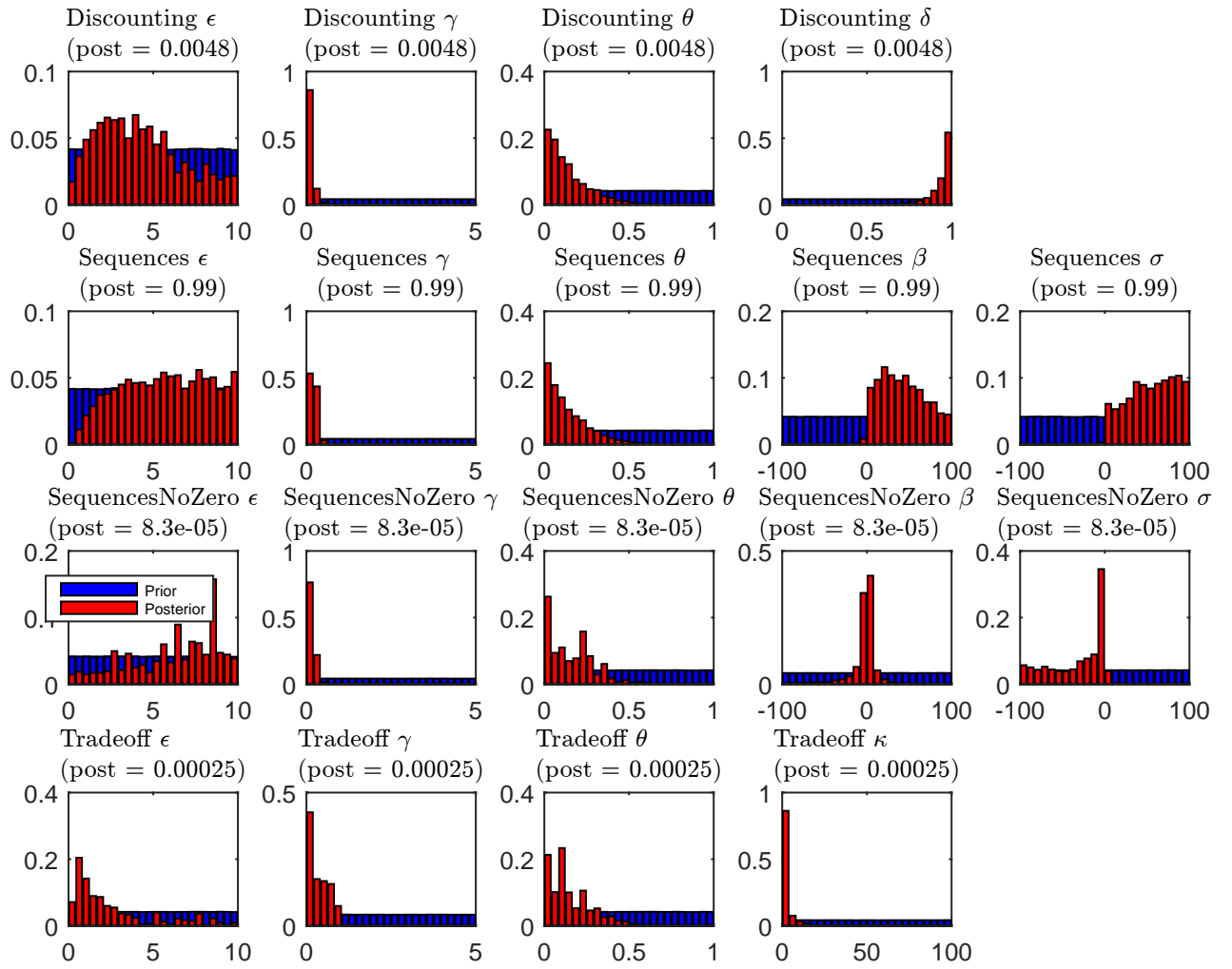

Supplement: Supplementary file 1 [file Scholten_Individuals.zip › plots/e29_p127_eg2_priors_and_posteriors.pdf]

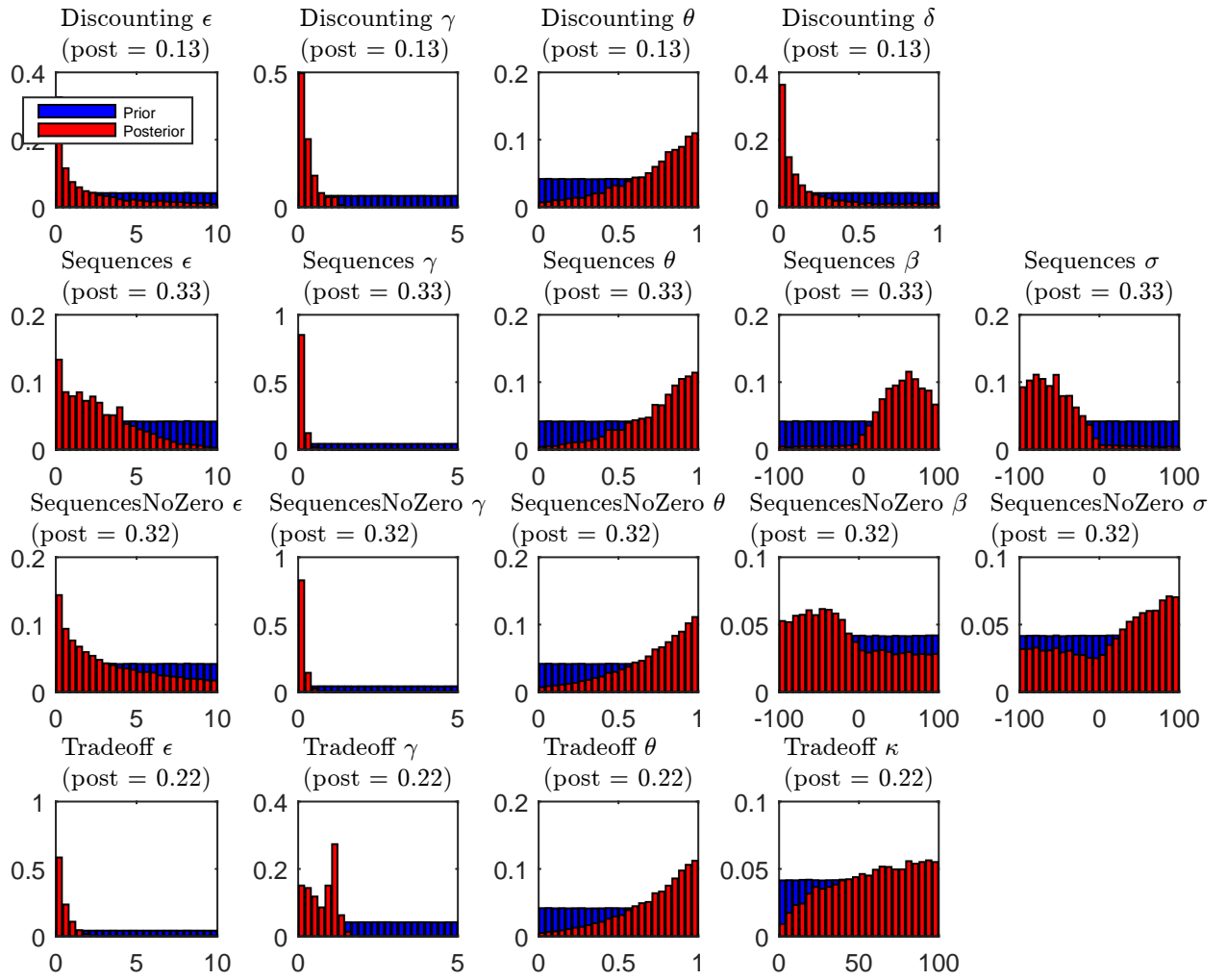

Supplement: Supplementary file 1 [file Scholten_Individuals.zip › plots/e29_p128_eg2_priors_and_posteriors.pdf]

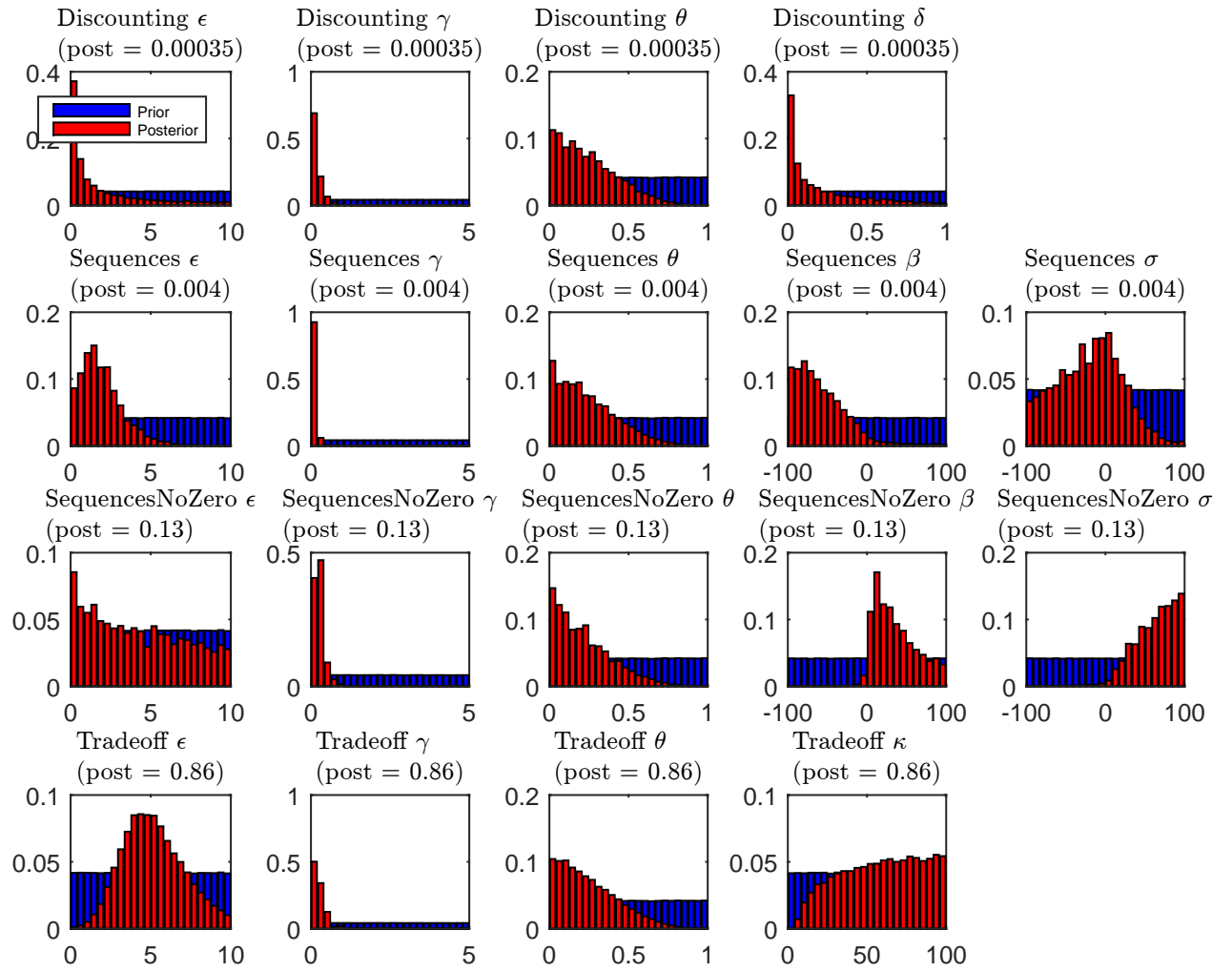

Supplement: Supplementary file 1 [file Scholten_Individuals.zip › plots/e29_p129_eg2_priors_and_posteriors.pdf]

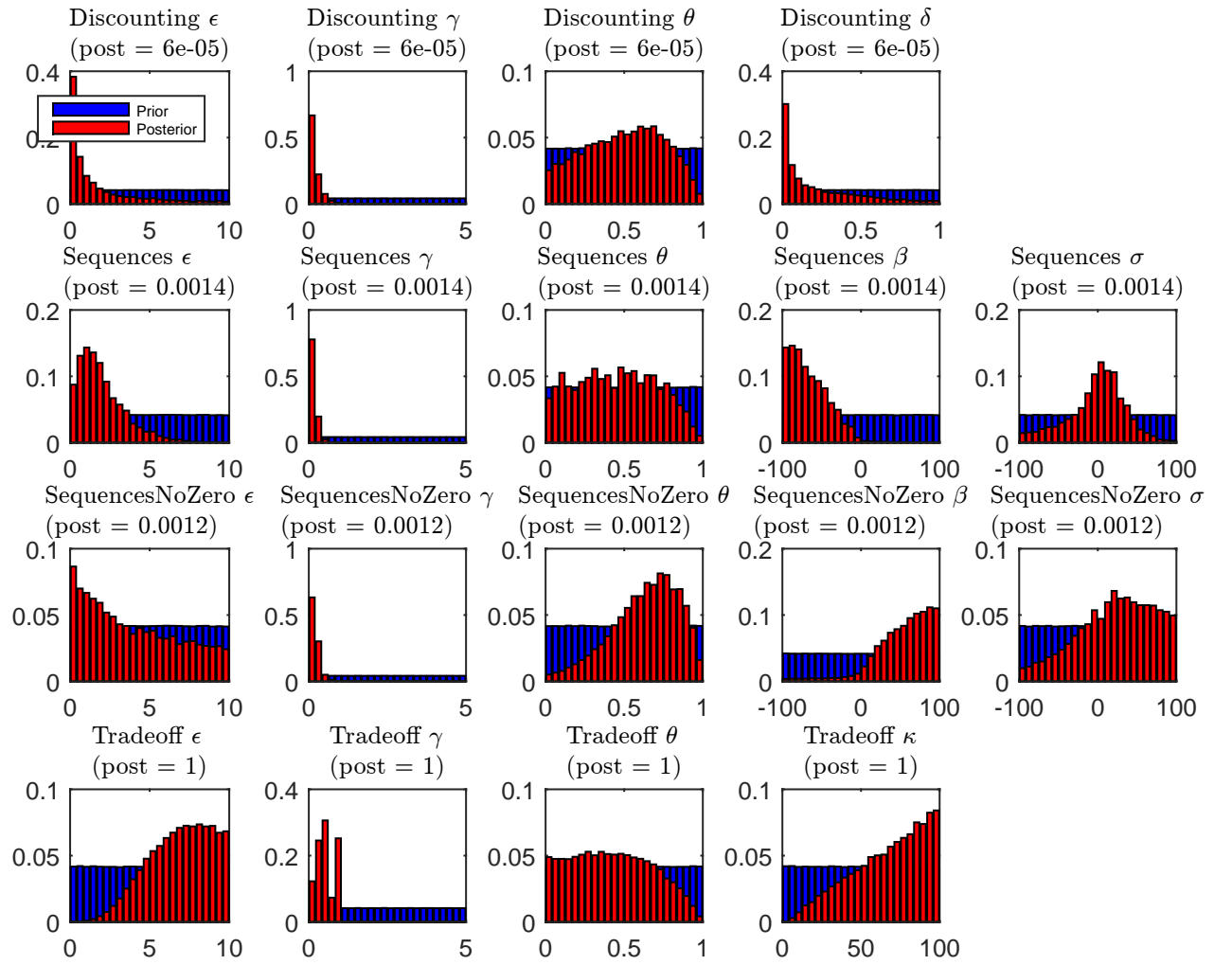

Supplement: Supplementary file 1 [file Scholten_Individuals.zip › plots/e29_p13_eg2_priors_and_posteriors.pdf]

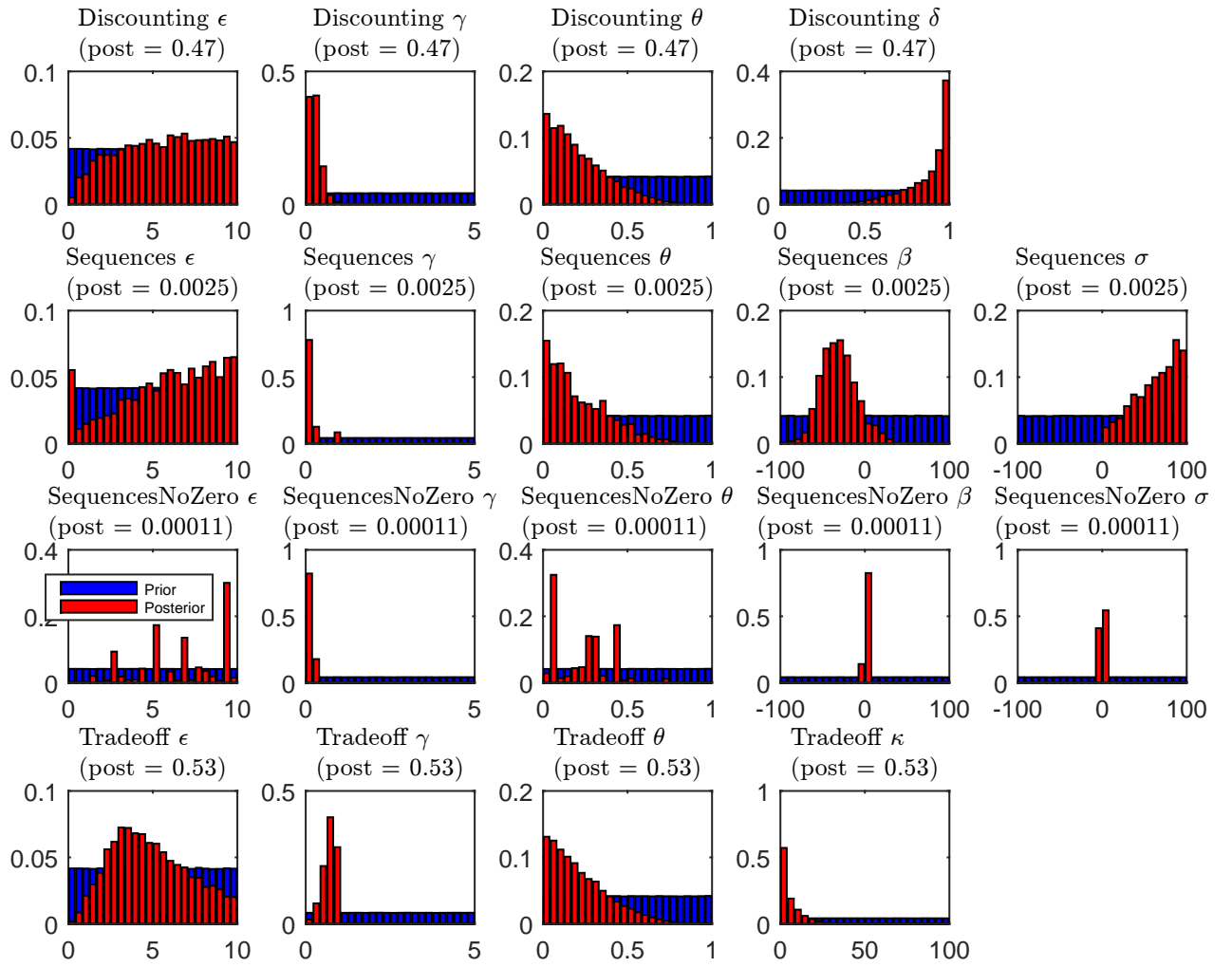

Supplement: Supplementary file 1 [file Scholten_Individuals.zip › plots/e29_p130_eg2_priors_and_posteriors.pdf]

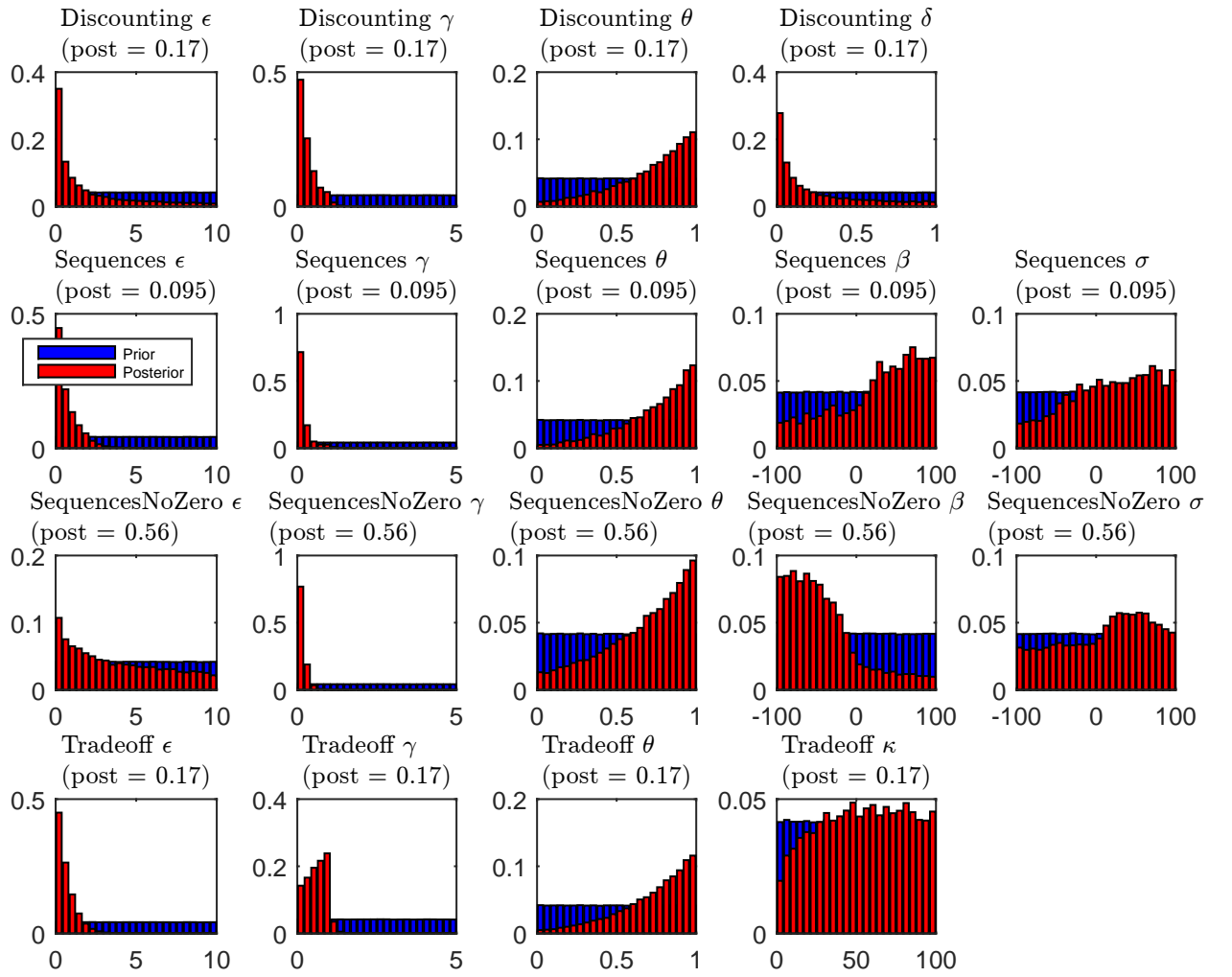

Supplement: Supplementary file 1 [file Scholten_Individuals.zip › plots/e29_p131_eg2_priors_and_posteriors.pdf]

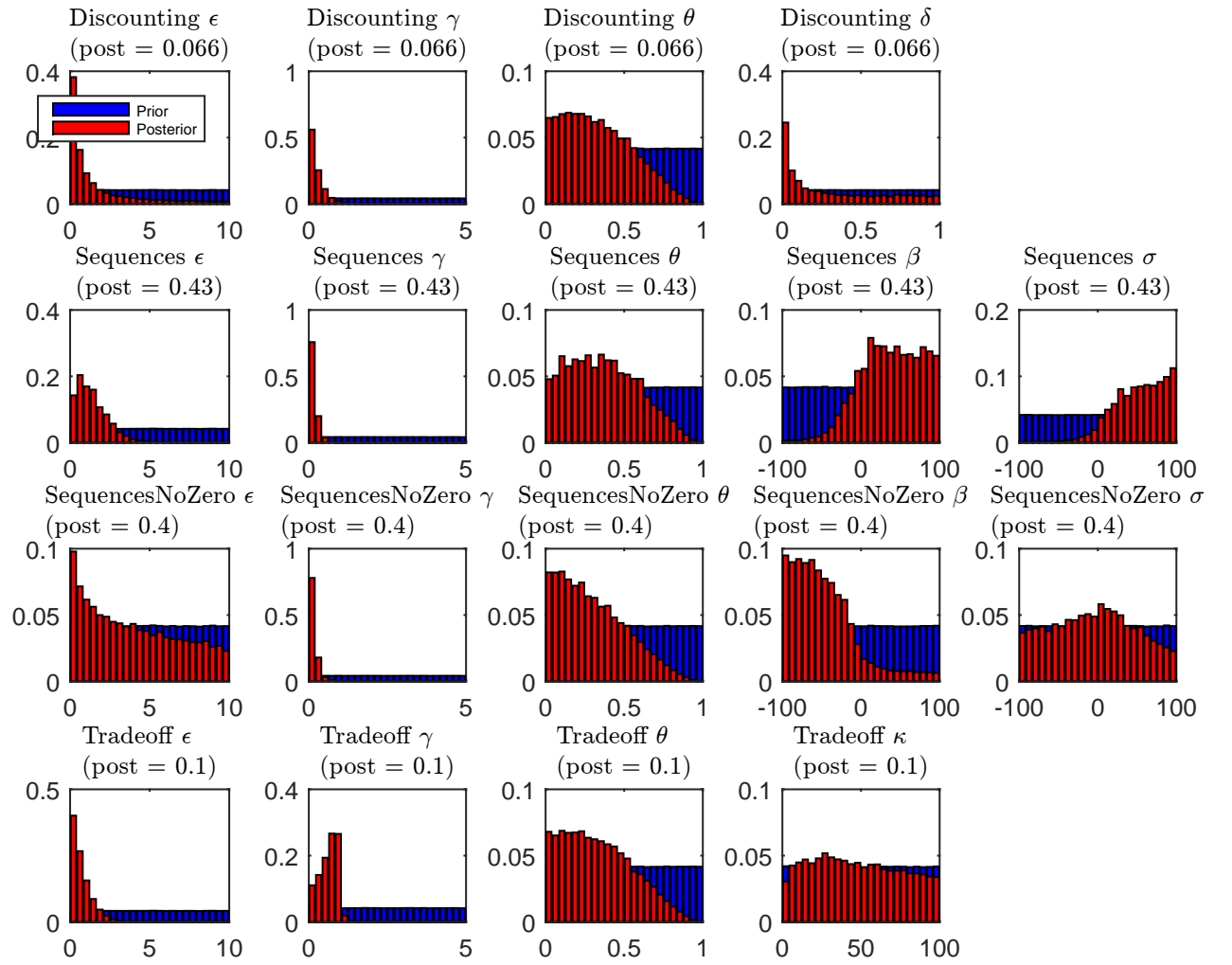

Supplement: Supplementary file 1 [file Scholten_Individuals.zip › plots/e29_p132_eg2_priors_and_posteriors.pdf]

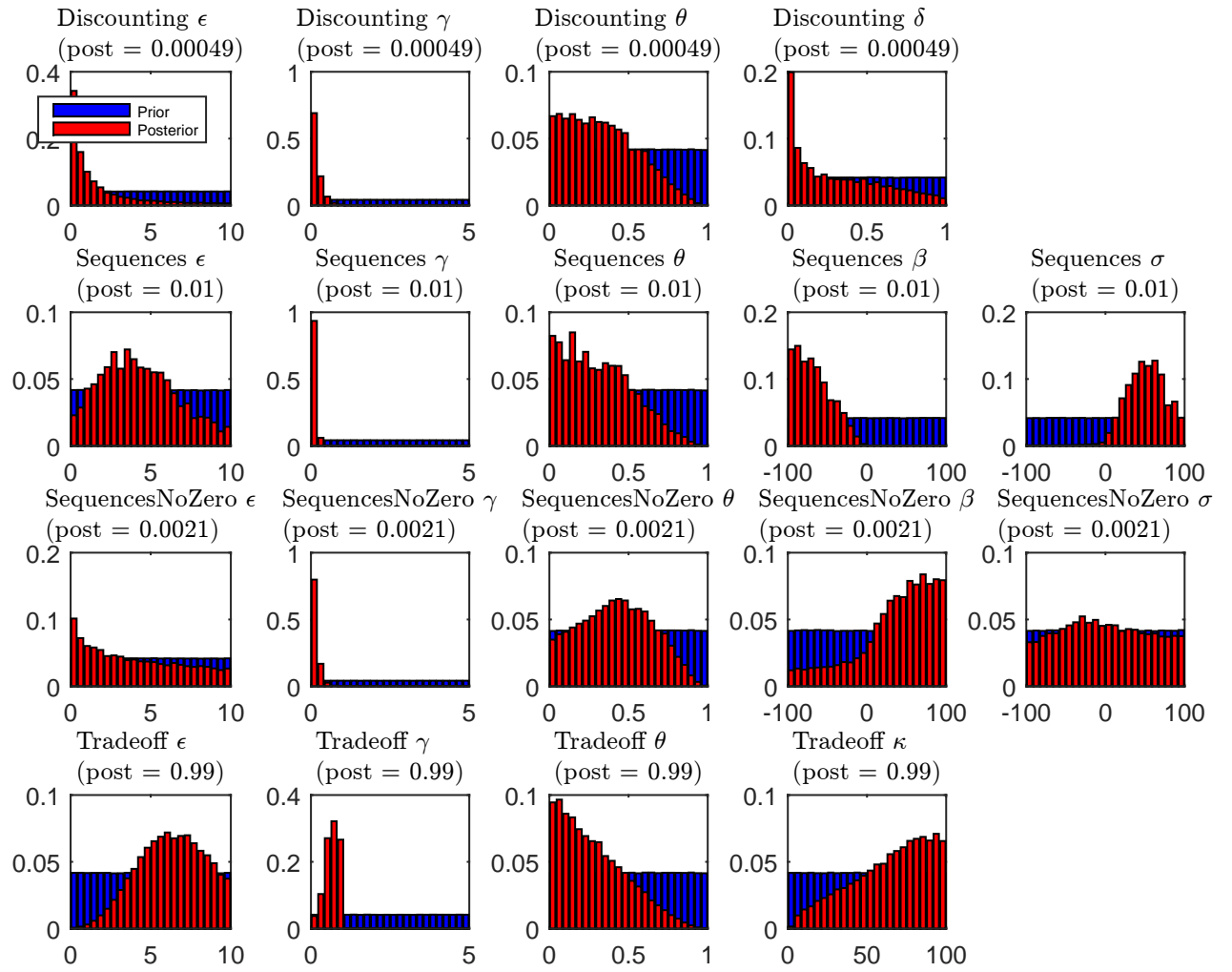

Supplement: Supplementary file 1 [file Scholten_Individuals.zip › plots/e29_p133_eg2_priors_and_posteriors.pdf]

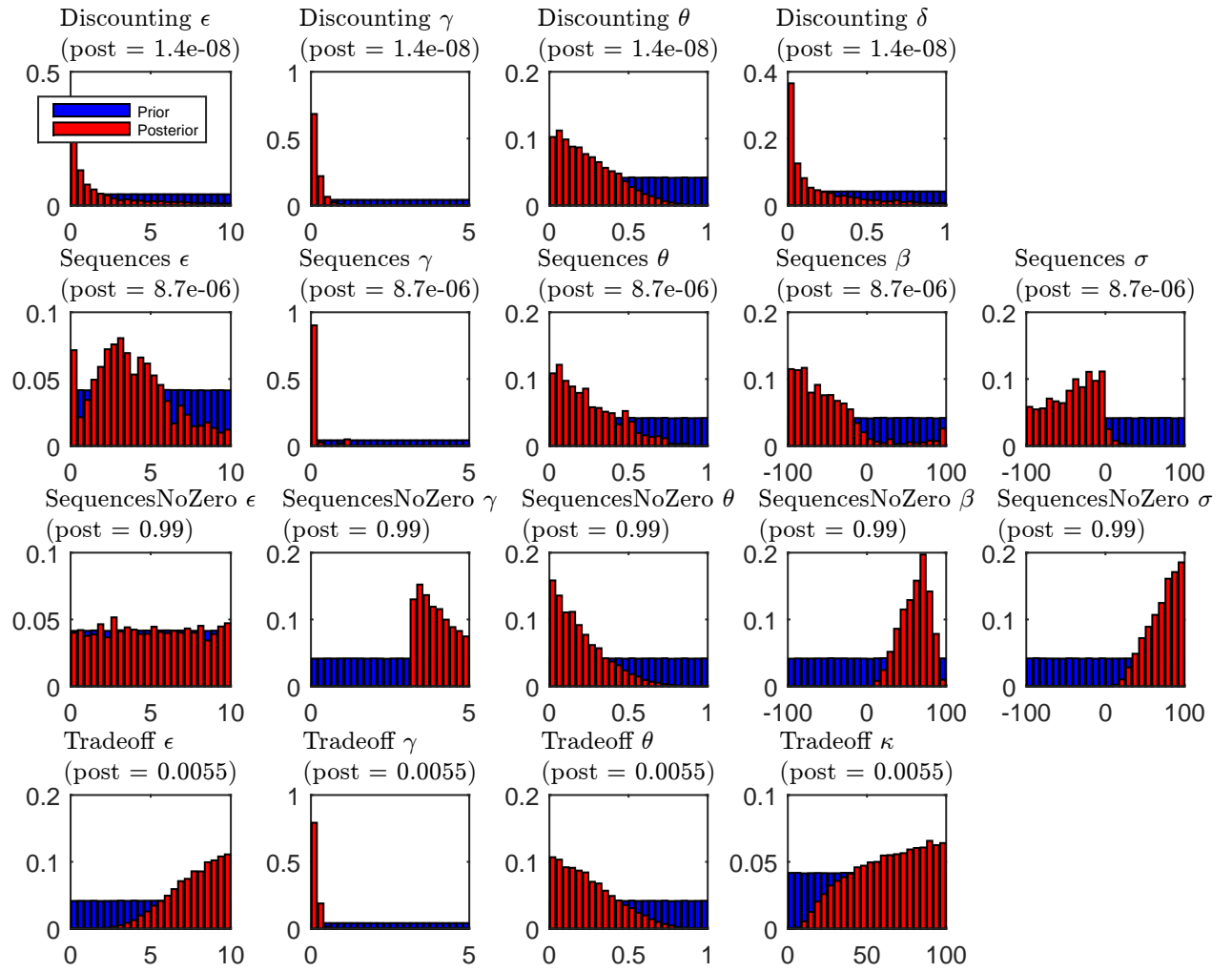

Supplement: Supplementary file 1 [file Scholten_Individuals.zip › plots/e29_p134_eg2_priors_and_posteriors.pdf]

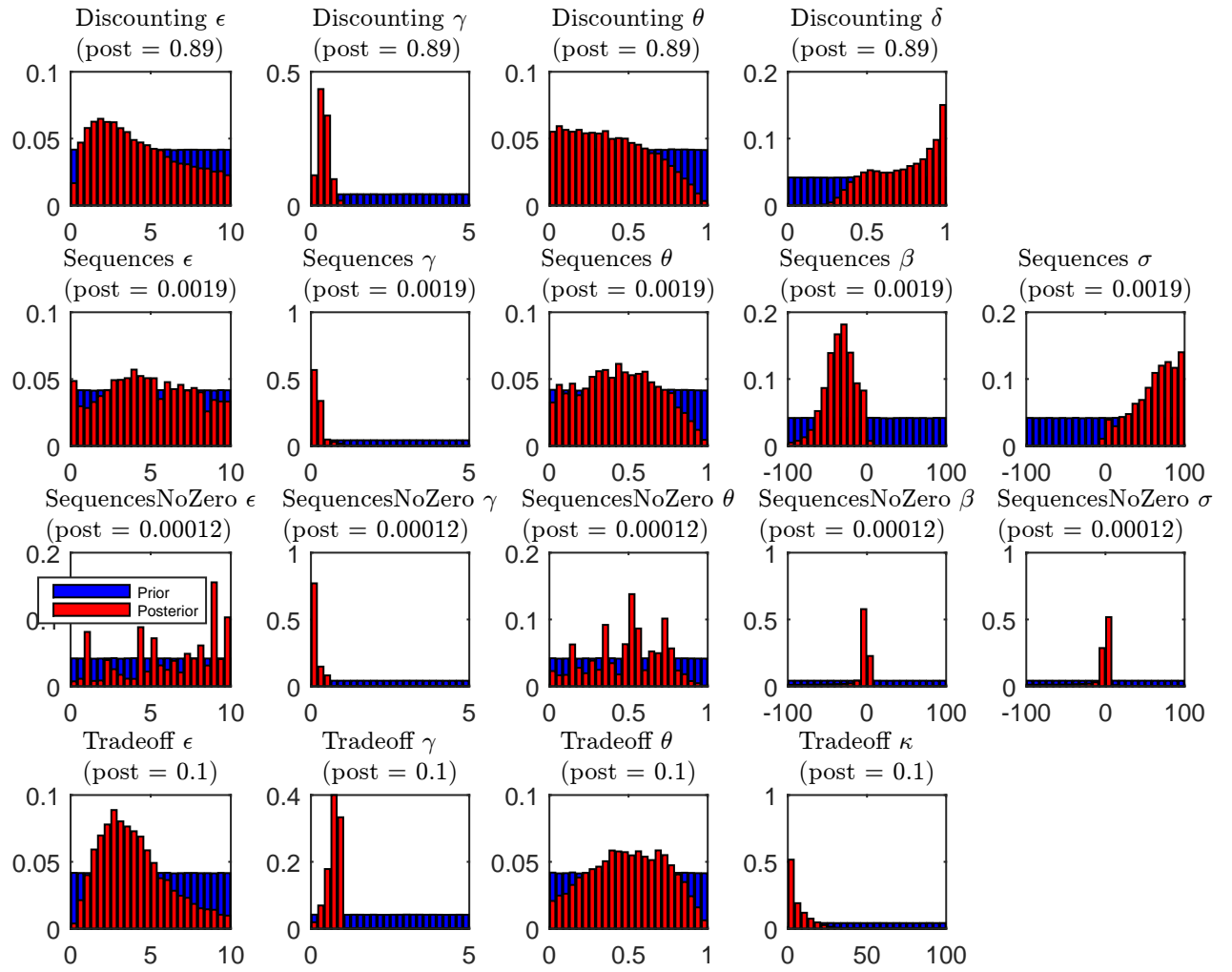

Supplement: Supplementary file 1 [file Scholten_Individuals.zip › plots/e29_p135_eg2_priors_and_posteriors.pdf]

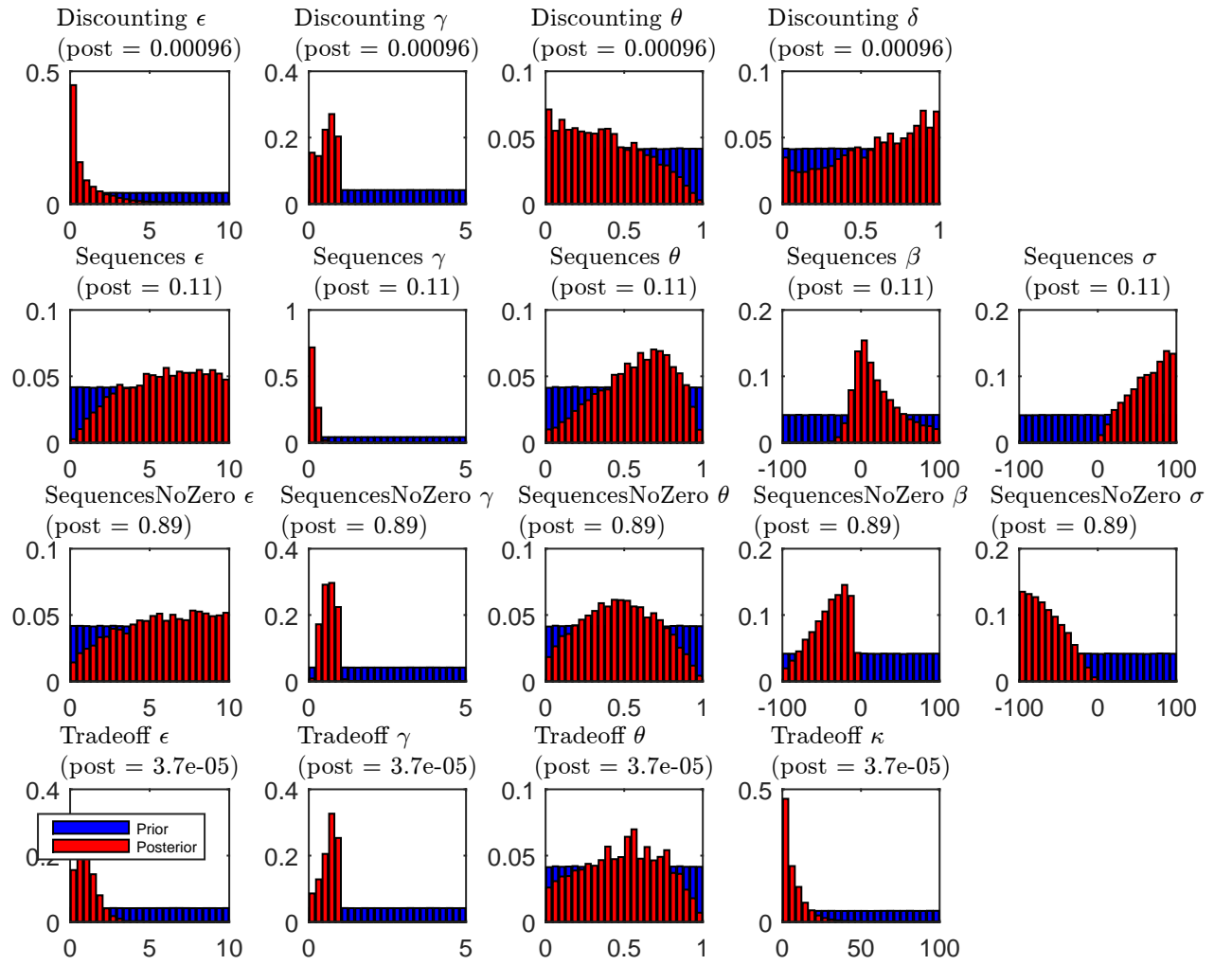

Supplement: Supplementary file 1 [file Scholten_Individuals.zip › plots/e29_p136_eg2_priors_and_posteriors.pdf]

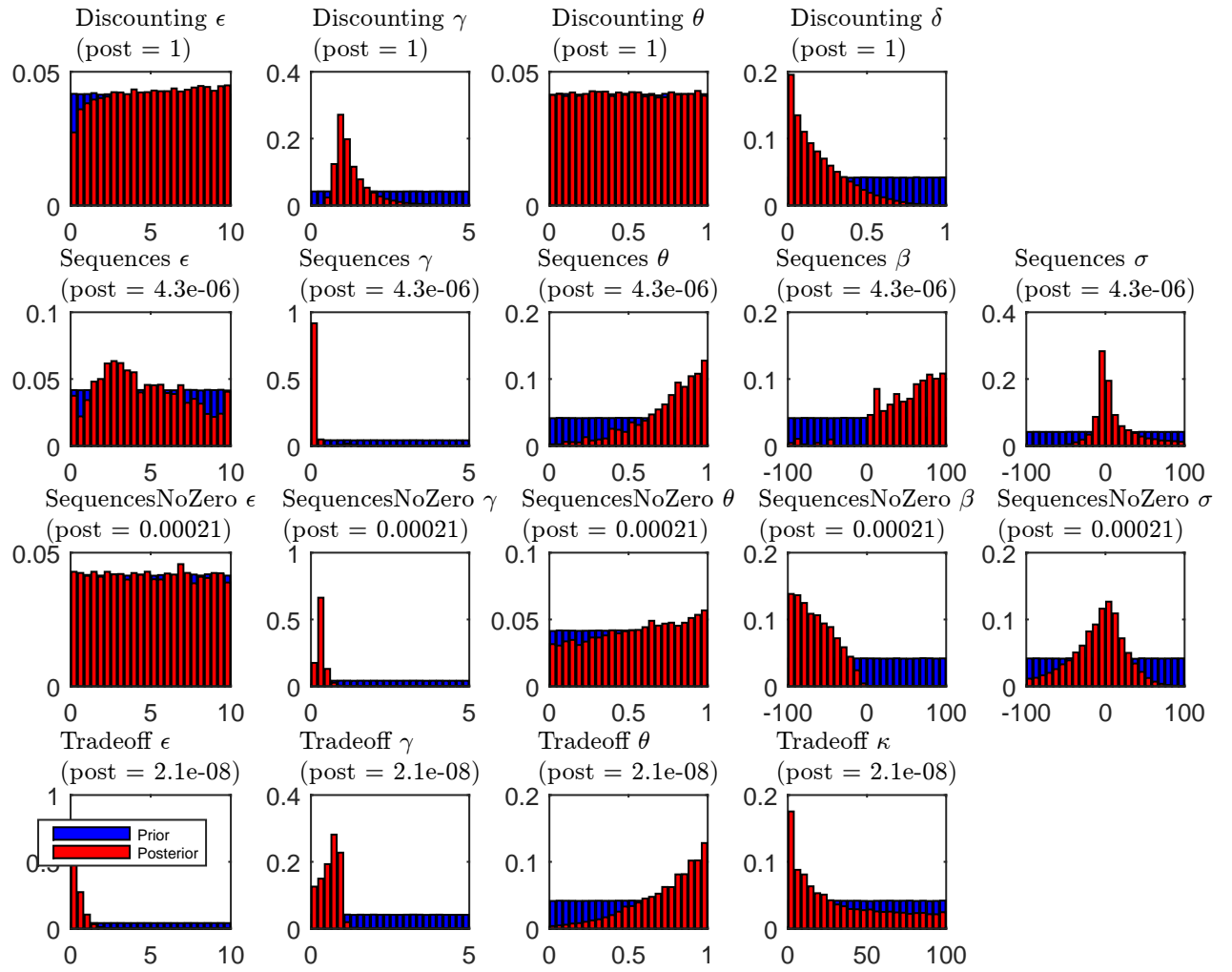

Supplement: Supplementary file 1 [file Scholten_Individuals.zip › plots/e29_p137_eg2_priors_and_posteriors.pdf]

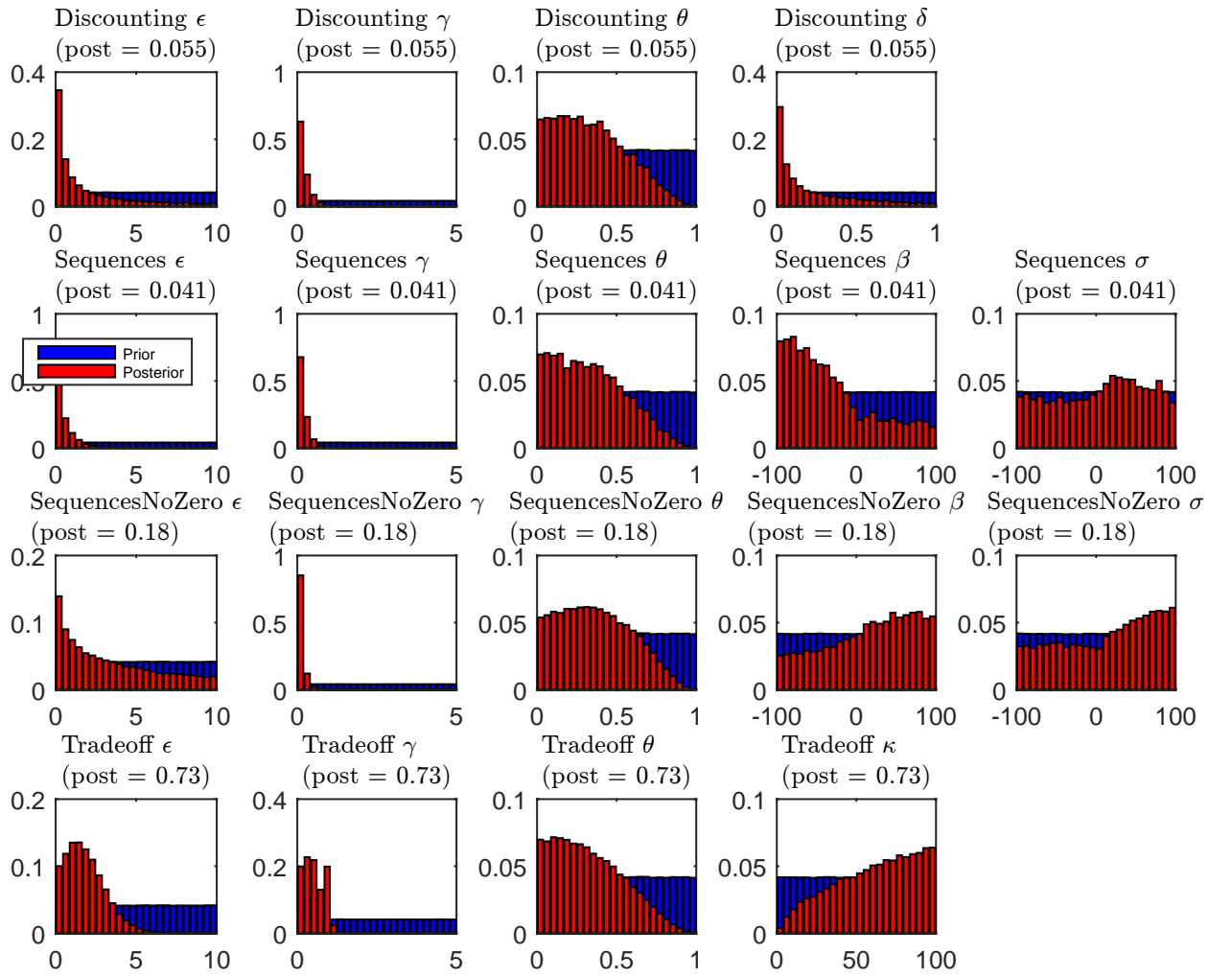

Supplement: Supplementary file 1 [file Scholten_Individuals.zip › plots/e29_p138_eg2_priors_and_posteriors.pdf]

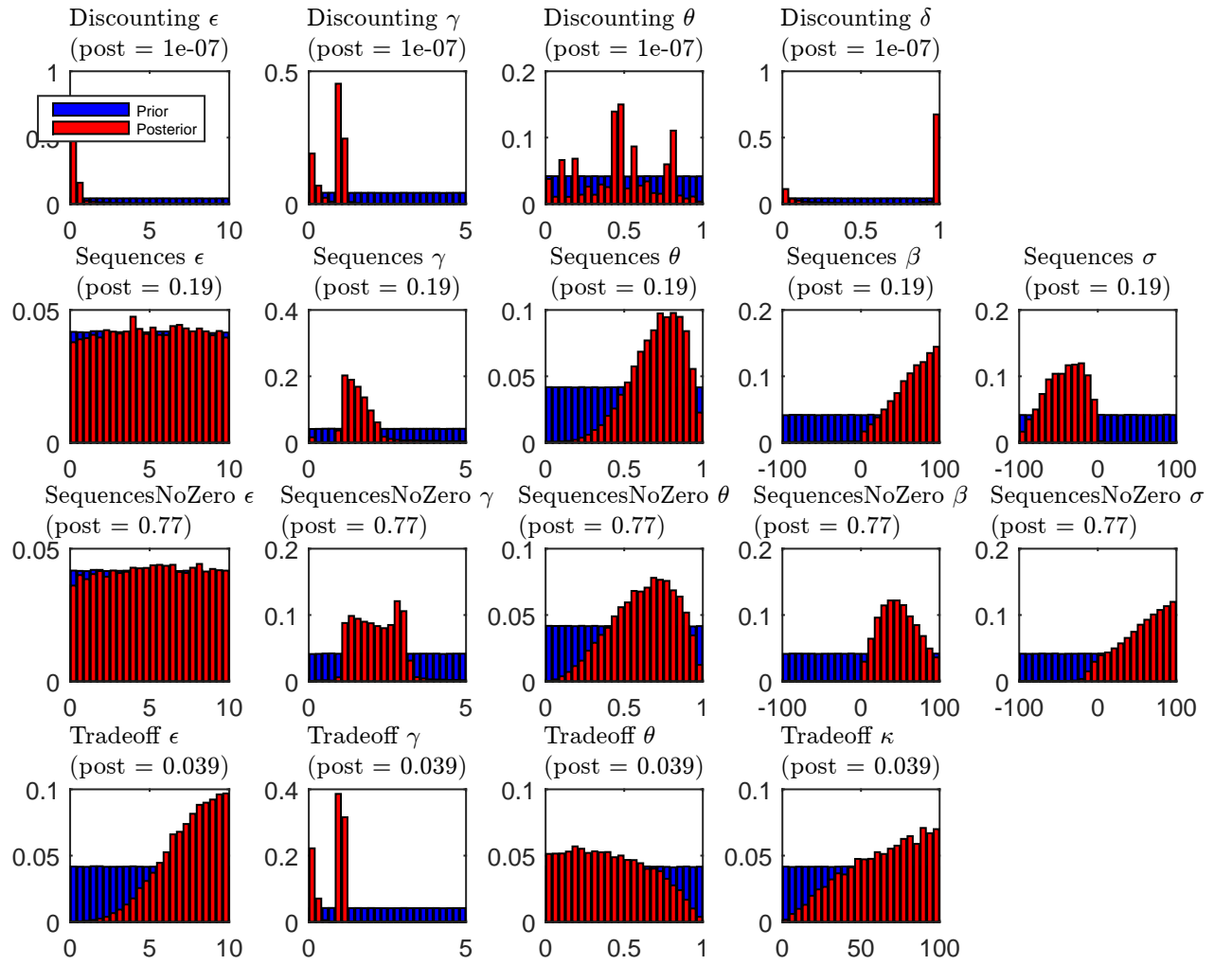

Supplement: Supplementary file 1 [file Scholten_Individuals.zip › plots/e29_p139_eg2_priors_and_posteriors.pdf]

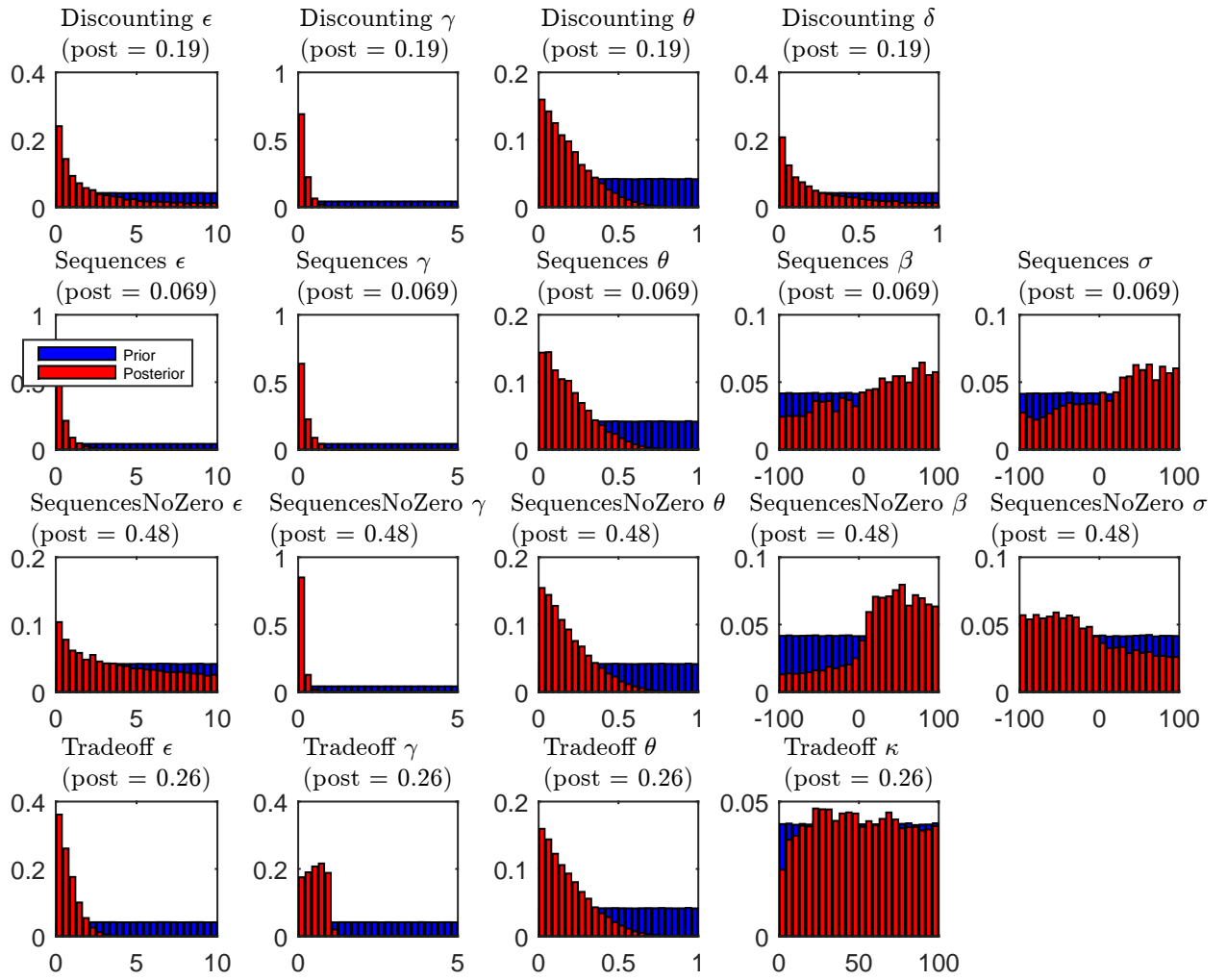

Supplement: Supplementary file 1 [file Scholten_Individuals.zip › plots/e29_p14_eg2_priors_and_posteriors.pdf]

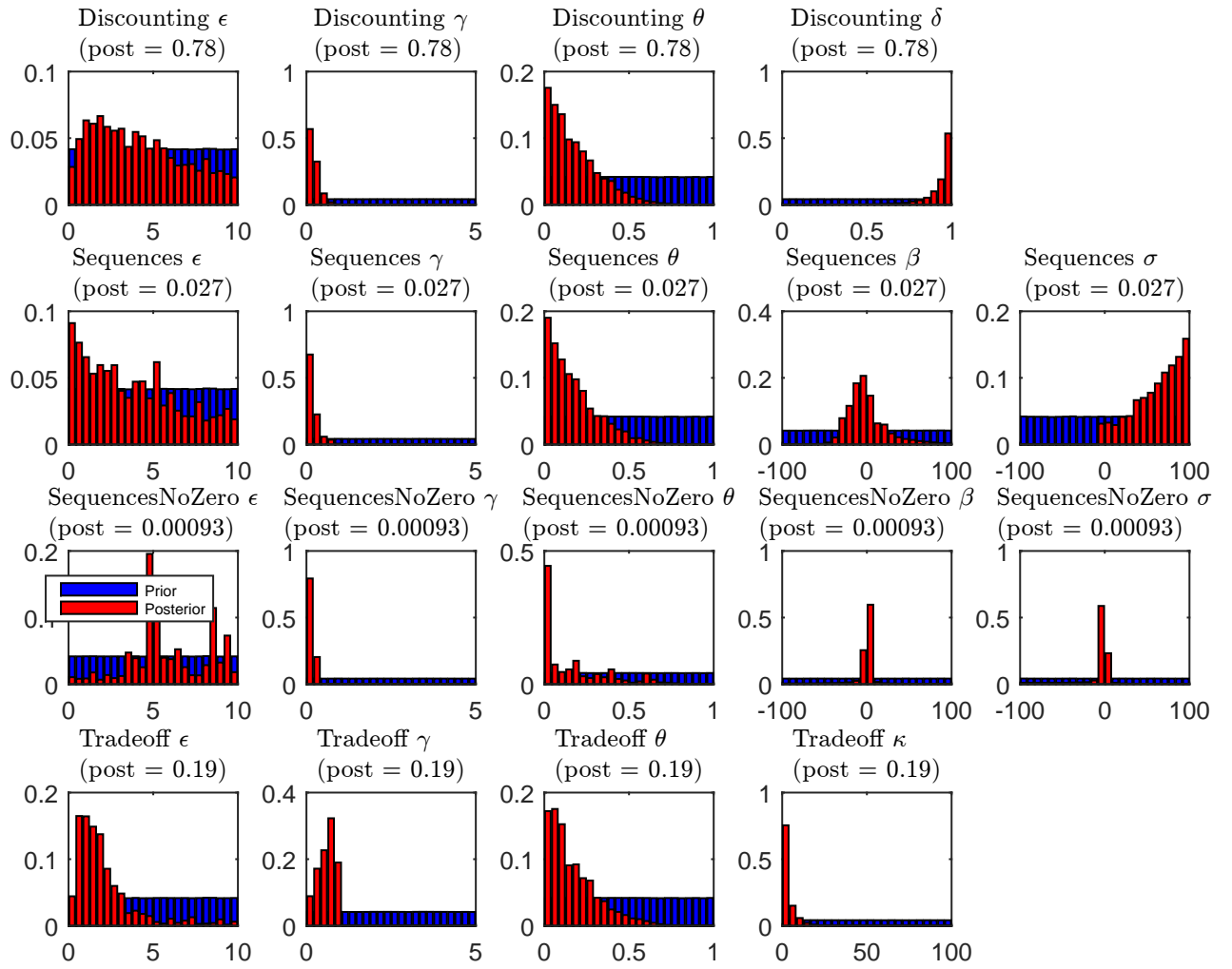

Supplement: Supplementary file 1 [file Scholten_Individuals.zip › plots/e29_p140_eg2_priors_and_posteriors.pdf]

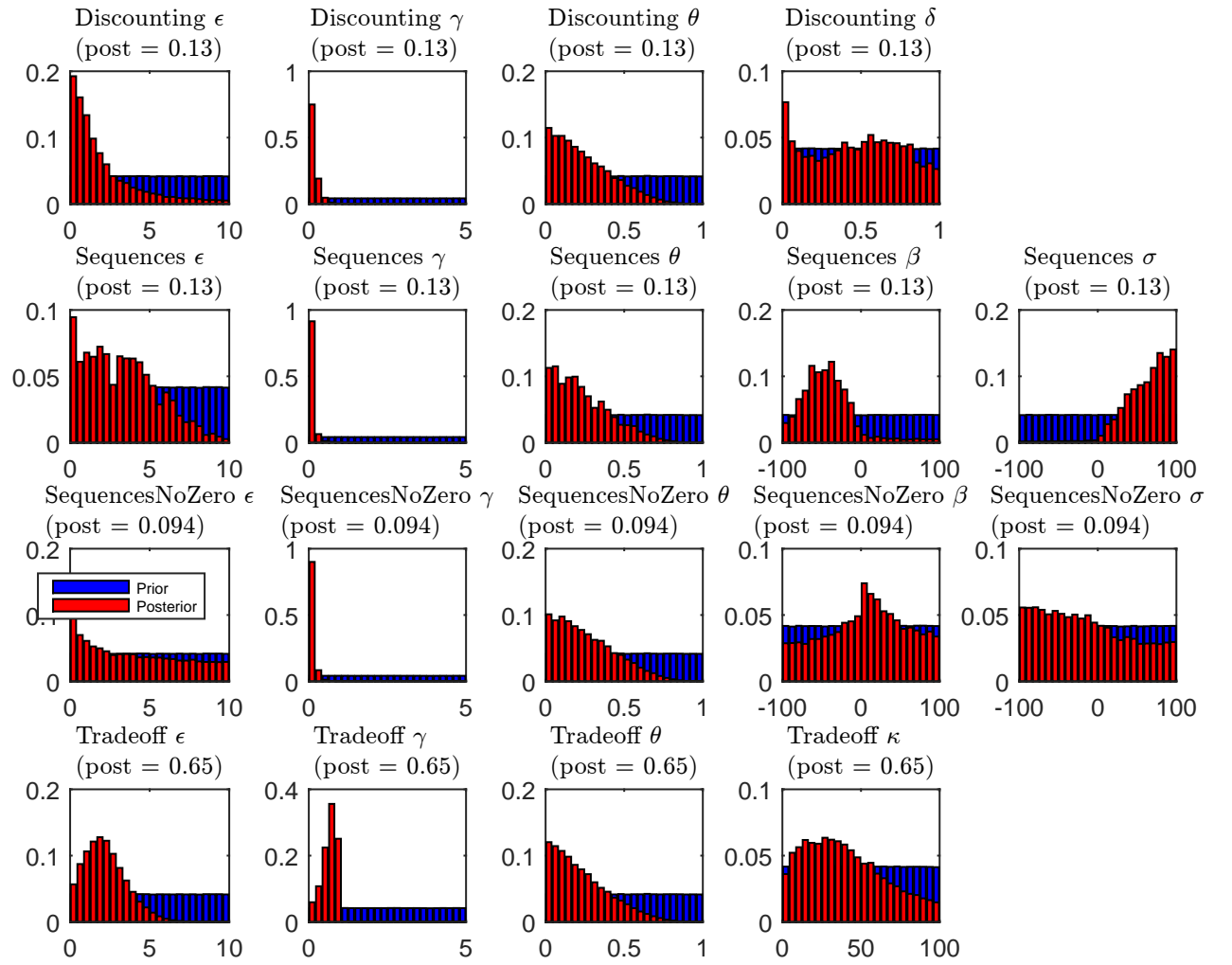

Supplement: Supplementary file 1 [file Scholten_Individuals.zip › plots/e29_p141_eg2_priors_and_posteriors.pdf]

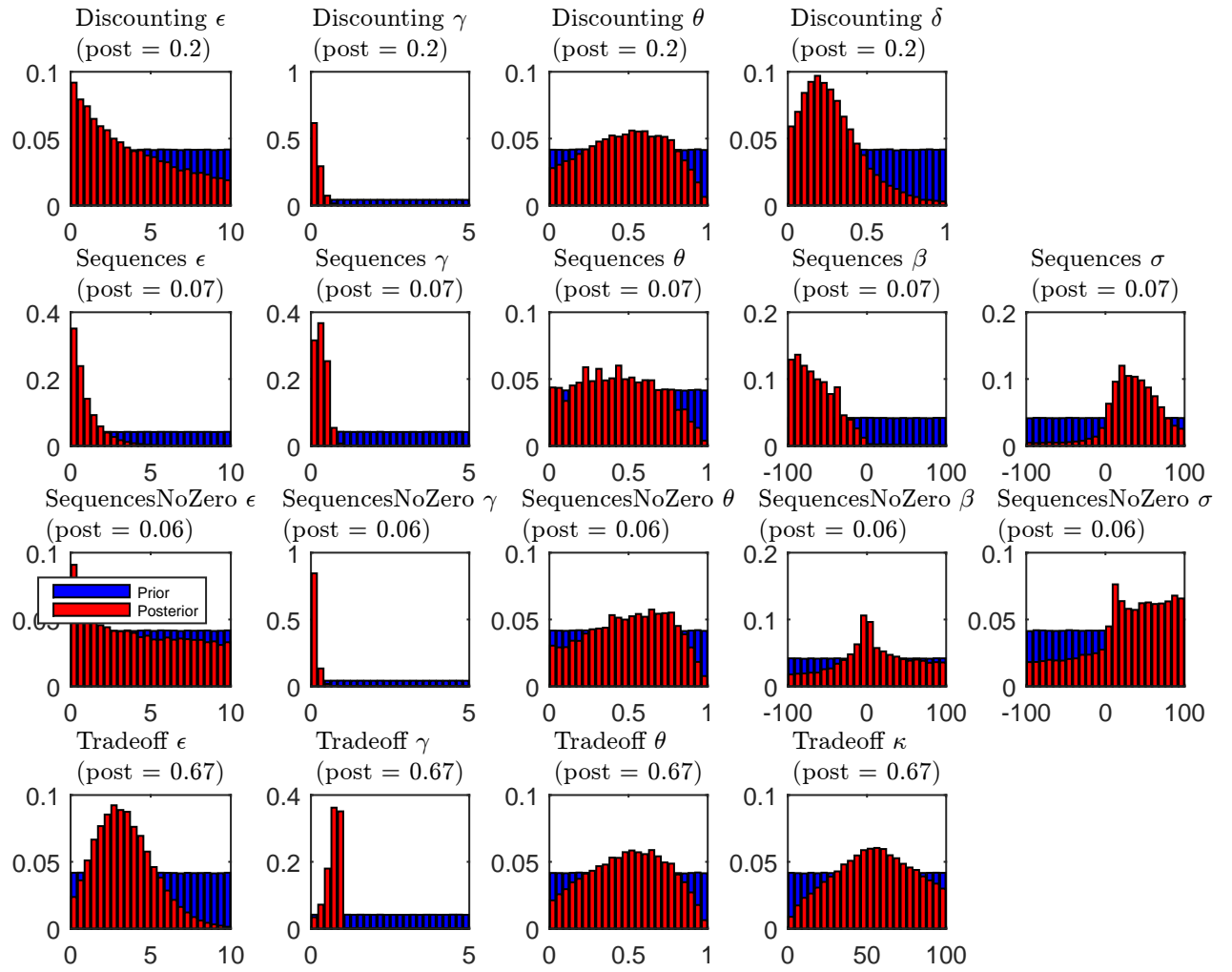

Supplement: Supplementary file 1 [file Scholten_Individuals.zip › plots/e29_p142_eg2_priors_and_posteriors.pdf]

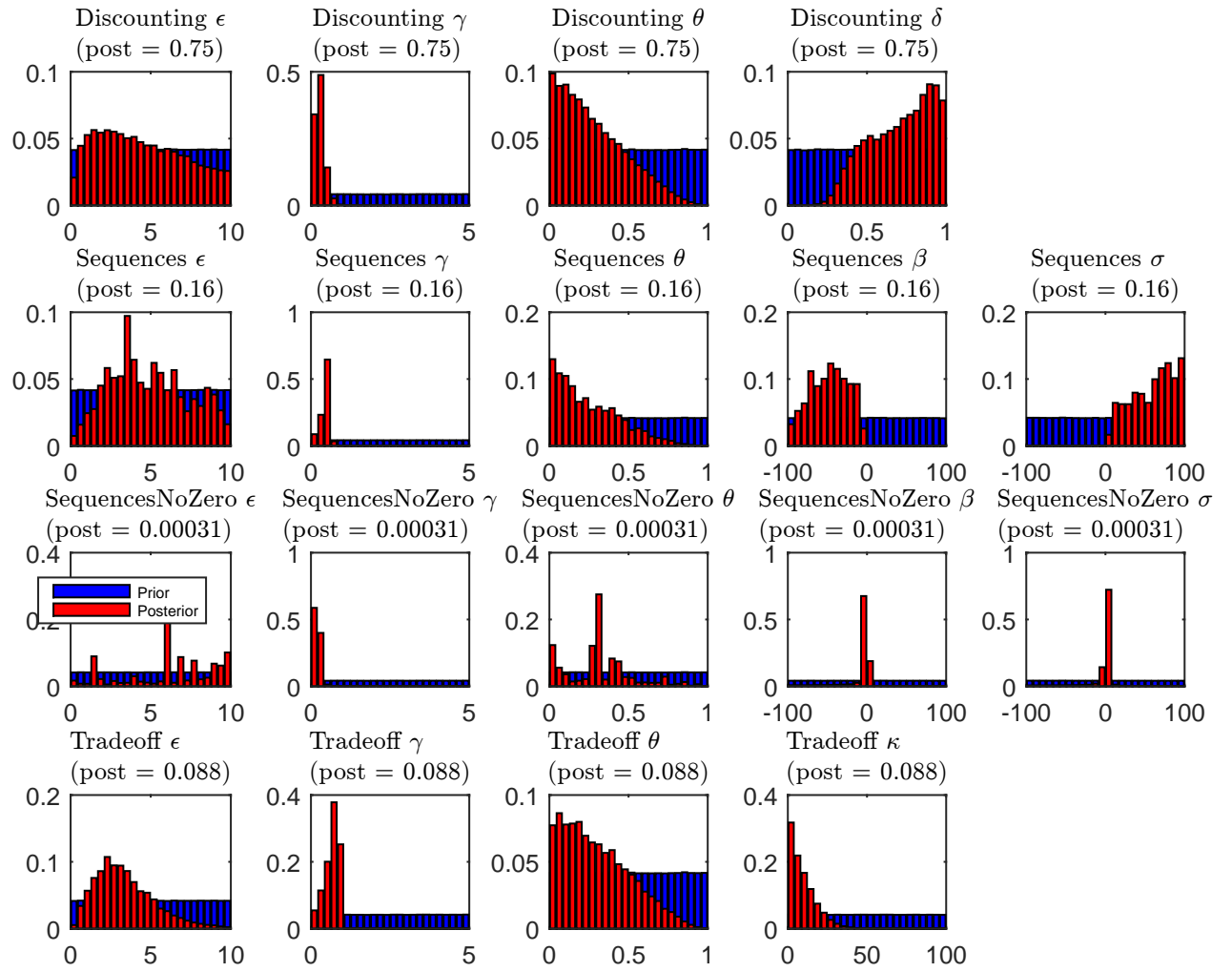

Supplement: Supplementary file 1 [file Scholten_Individuals.zip › plots/e29_p143_eg2_priors_and_posteriors.pdf]

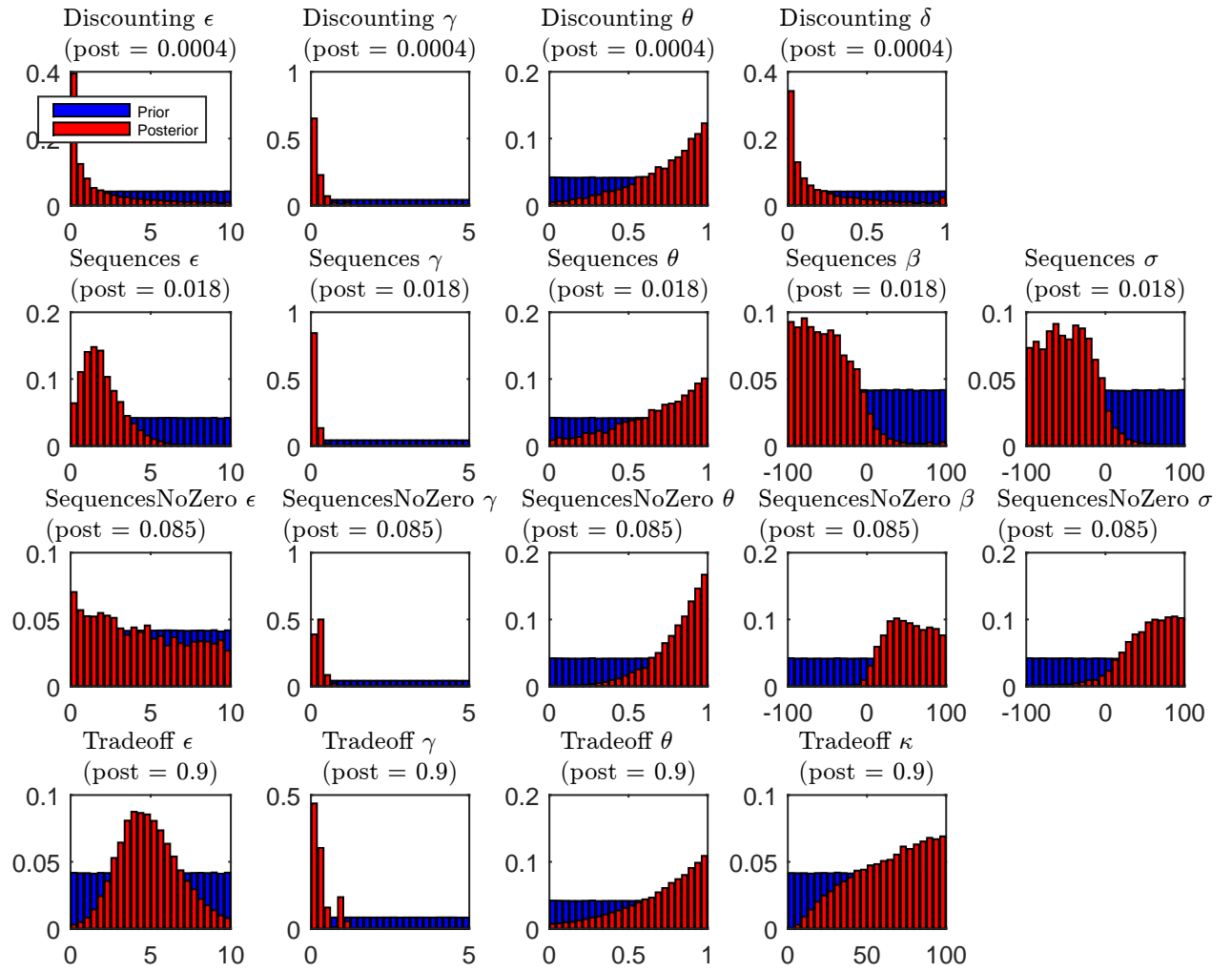

Supplement: Supplementary file 1 [file Scholten_Individuals.zip › plots/e29_p144_eg2_priors_and_posteriors.pdf]

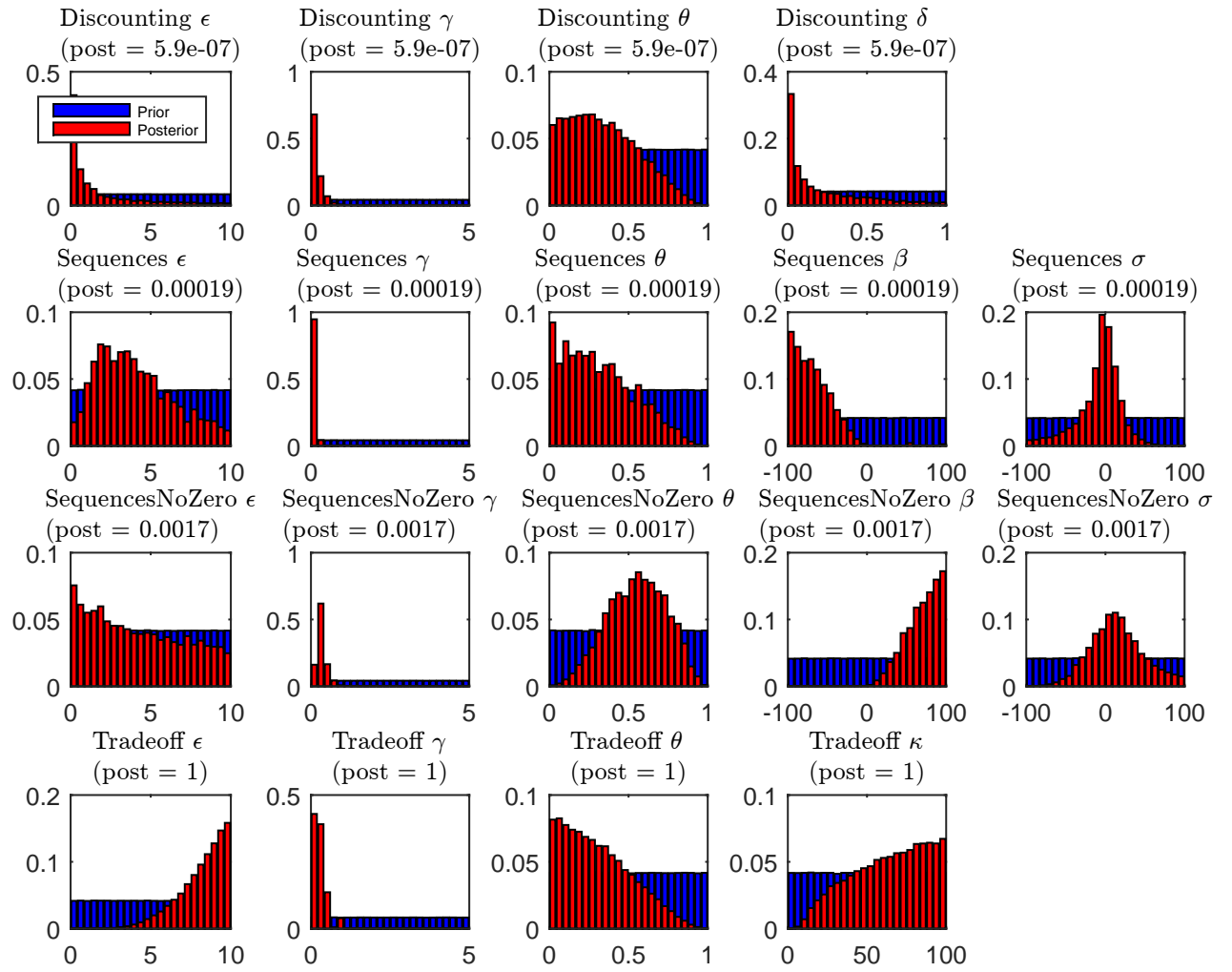

Supplement: Supplementary file 1 [file Scholten_Individuals.zip › plots/e29_p145_eg2_priors_and_posteriors.pdf]

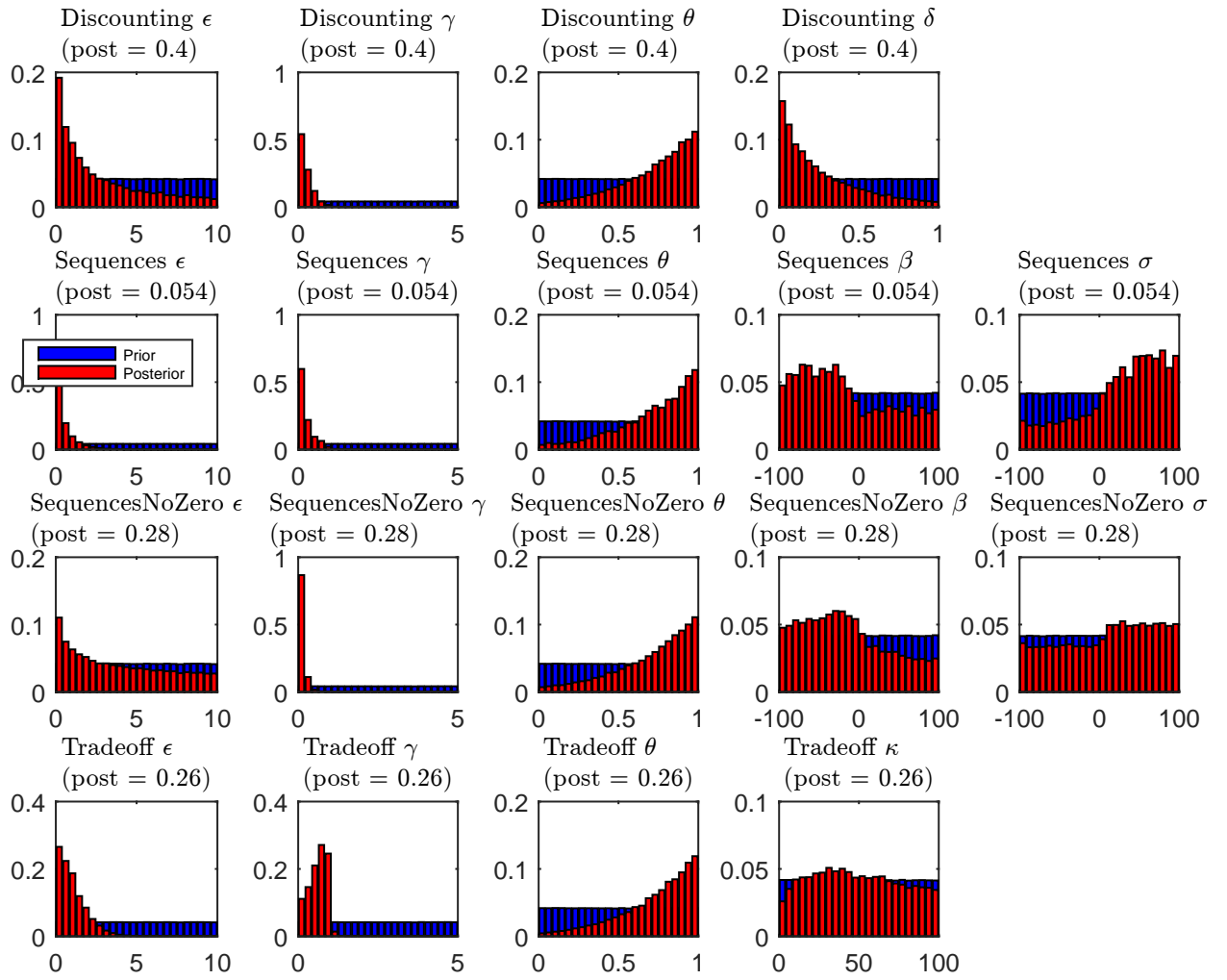

Supplement: Supplementary file 1 [file Scholten_Individuals.zip › plots/e29_p146_eg2_priors_and_posteriors.pdf]

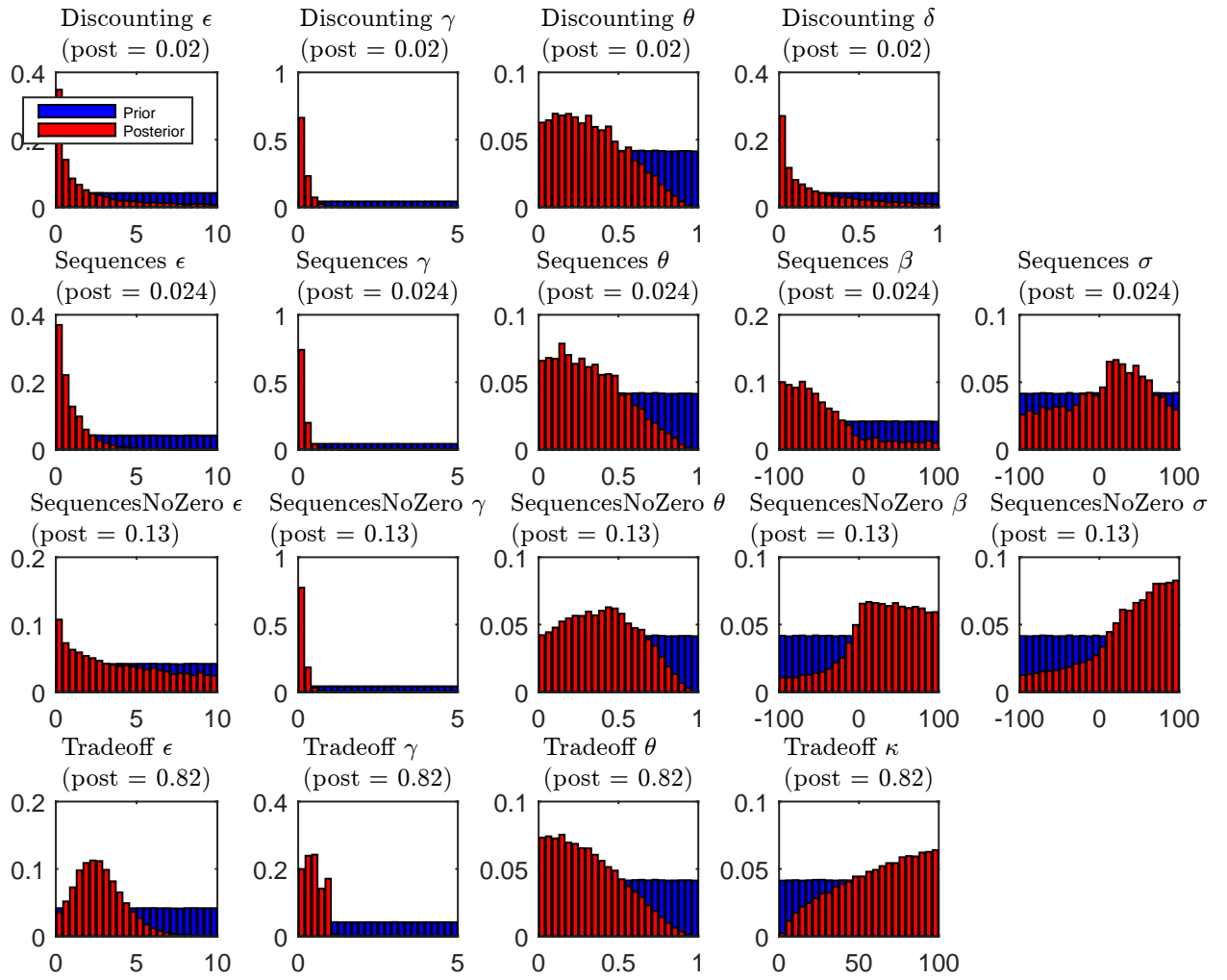

Supplement: Supplementary file 1 [file Scholten_Individuals.zip › plots/e29_p147_eg2_priors_and_posteriors.pdf]

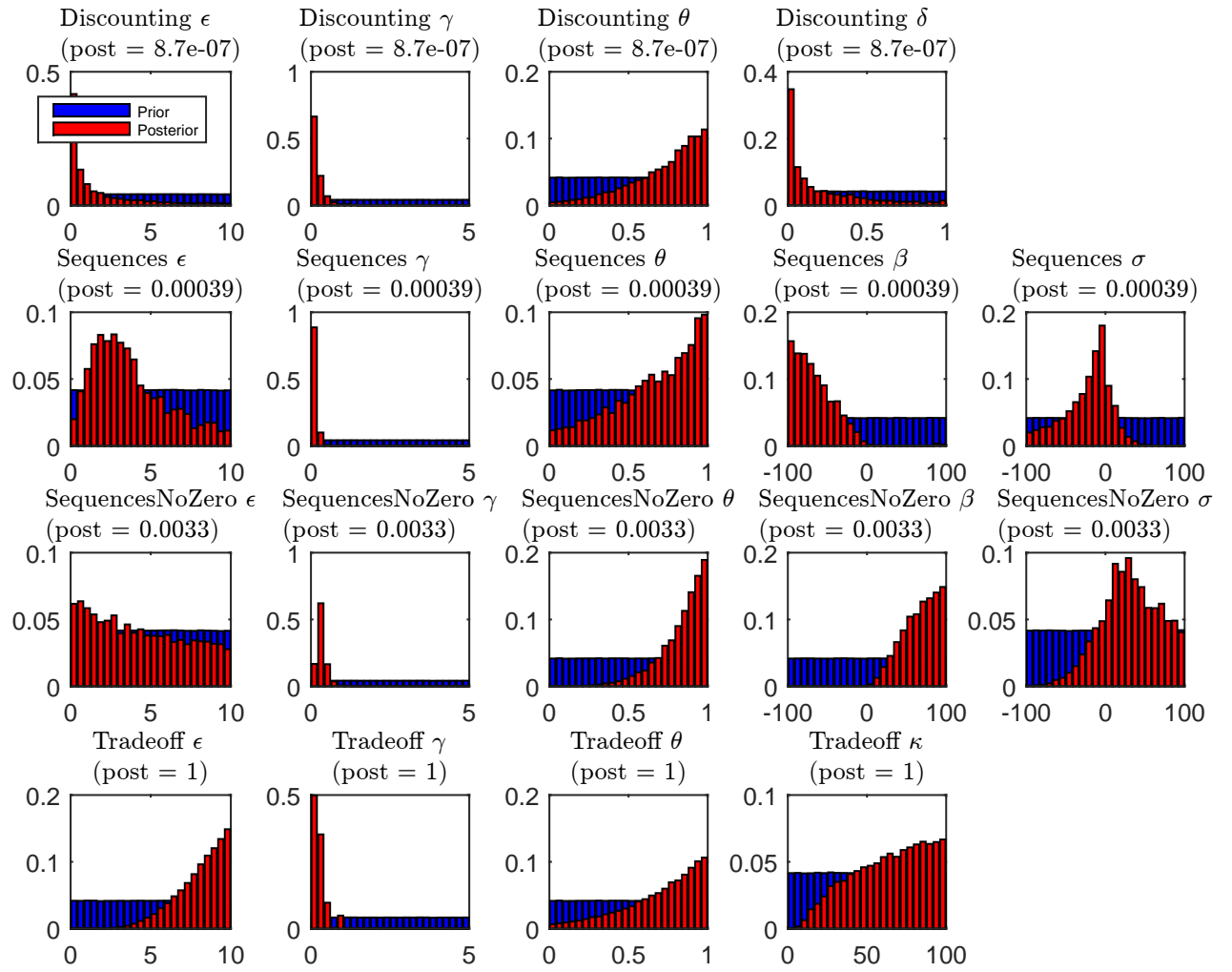

Supplement: Supplementary file 1 [file Scholten_Individuals.zip › plots/e29_p148_eg2_priors_and_posteriors.pdf]

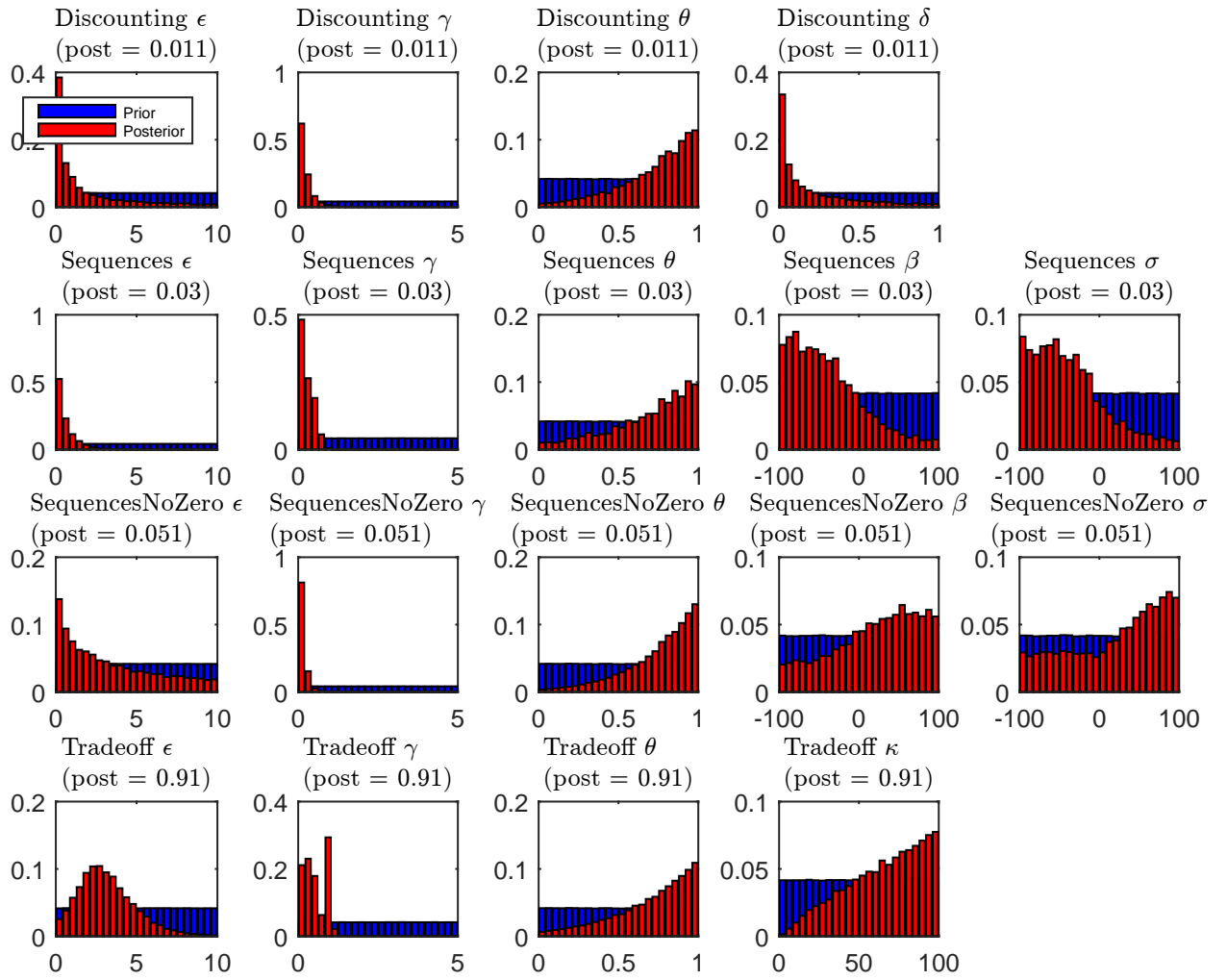

Supplement: Supplementary file 1 [file Scholten_Individuals.zip › plots/e29_p149_eg2_priors_and_posteriors.pdf]

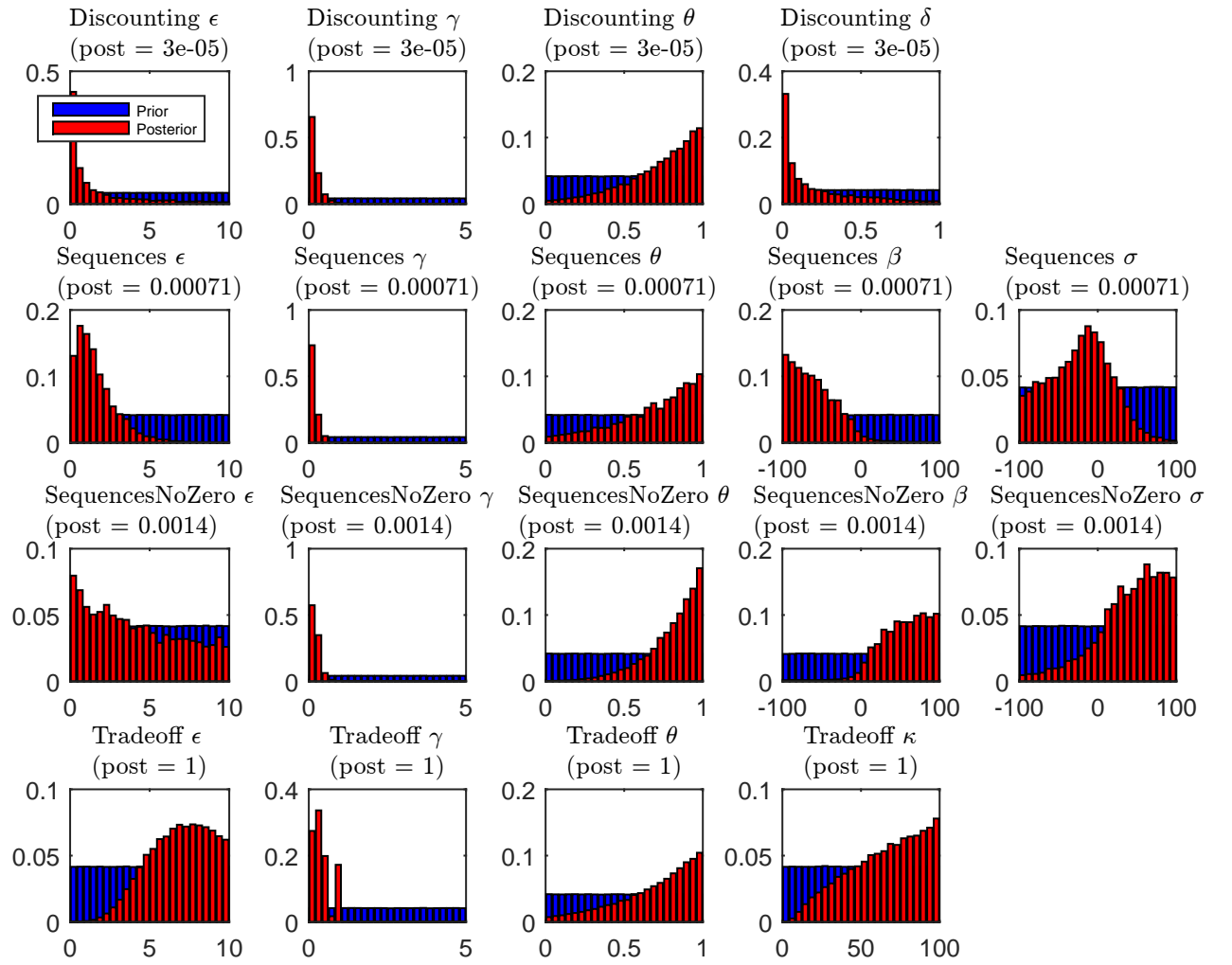

Supplement: Supplementary file 1 [file Scholten_Individuals.zip › plots/e29_p15_eg2_priors_and_posteriors.pdf]

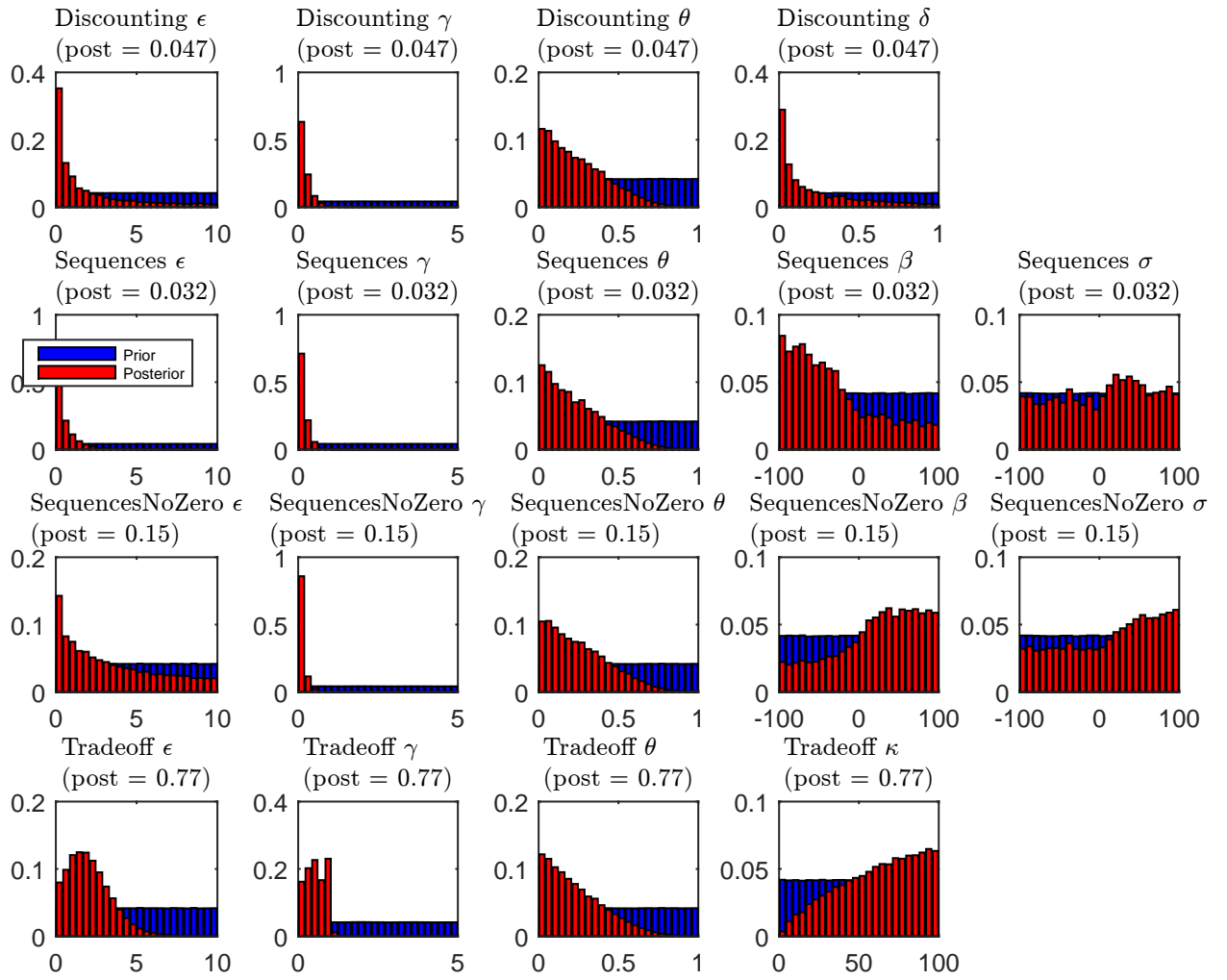

Supplement: Supplementary file 1 [file Scholten_Individuals.zip › plots/e29_p150_eg2_priors_and_posteriors.pdf]

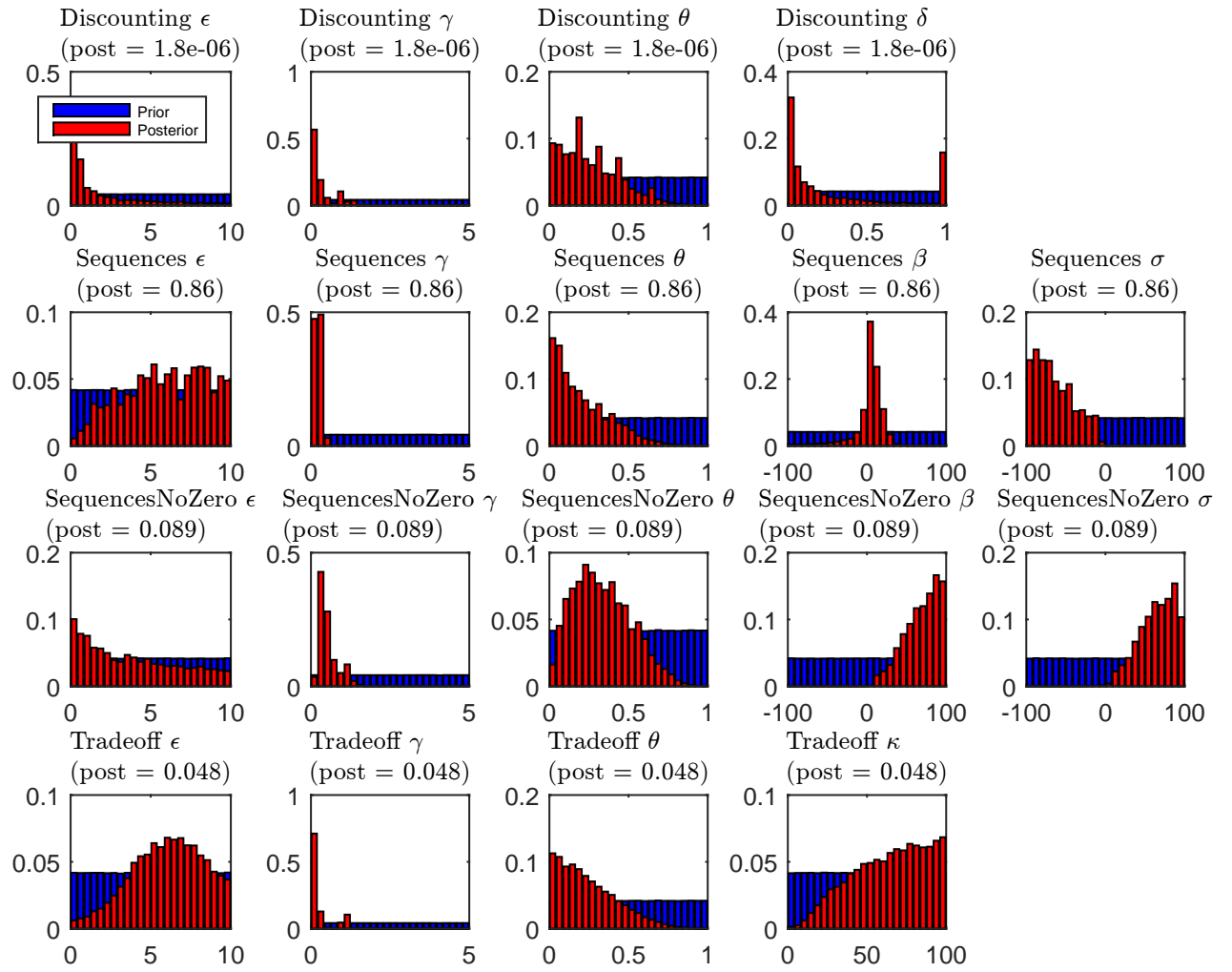

Supplement: Supplementary file 1 [file Scholten_Individuals.zip › plots/e29_p151_eg2_priors_and_posteriors.pdf]

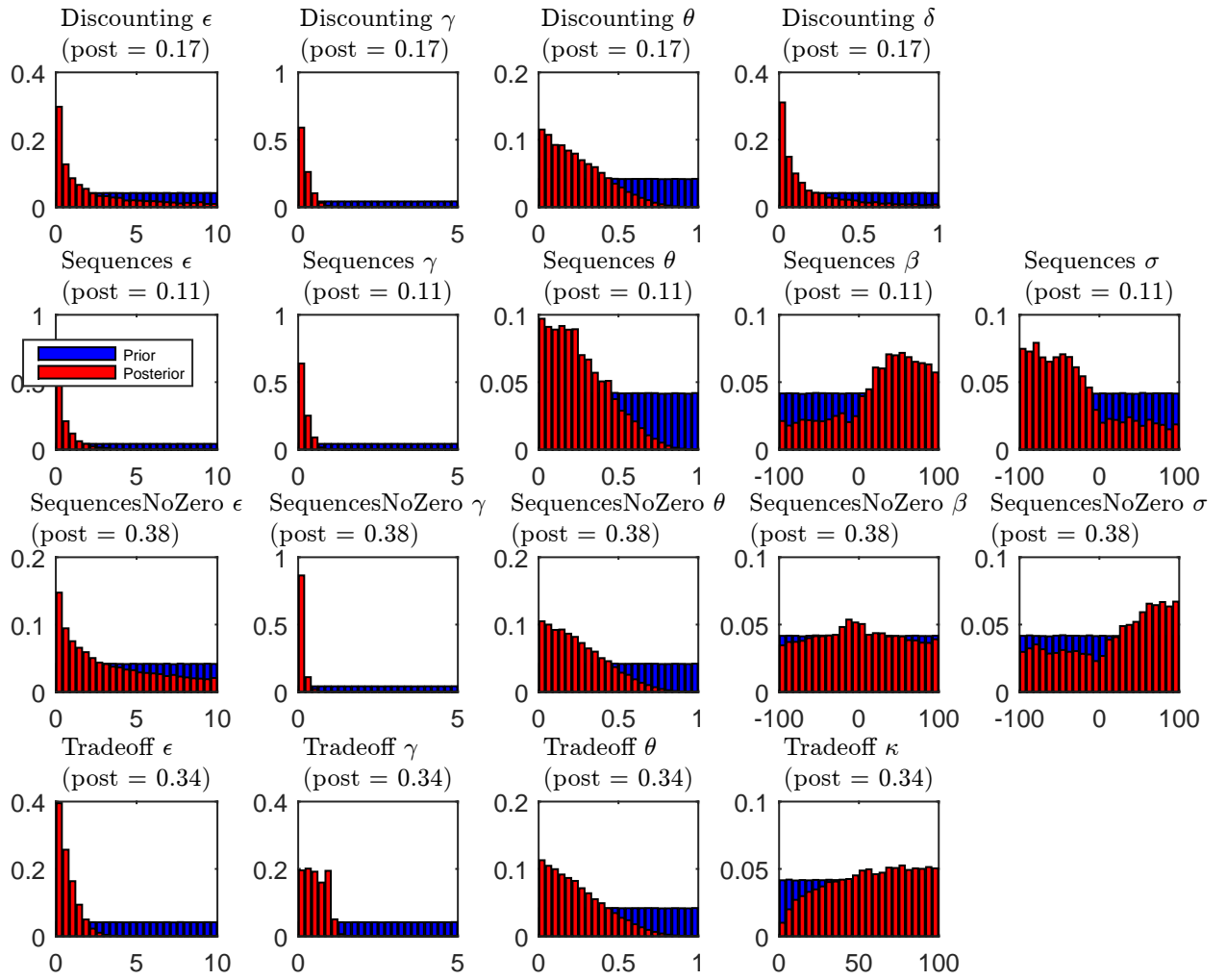

Supplement: Supplementary file 1 [file Scholten_Individuals.zip › plots/e29_p152_eg2_priors_and_posteriors.pdf]

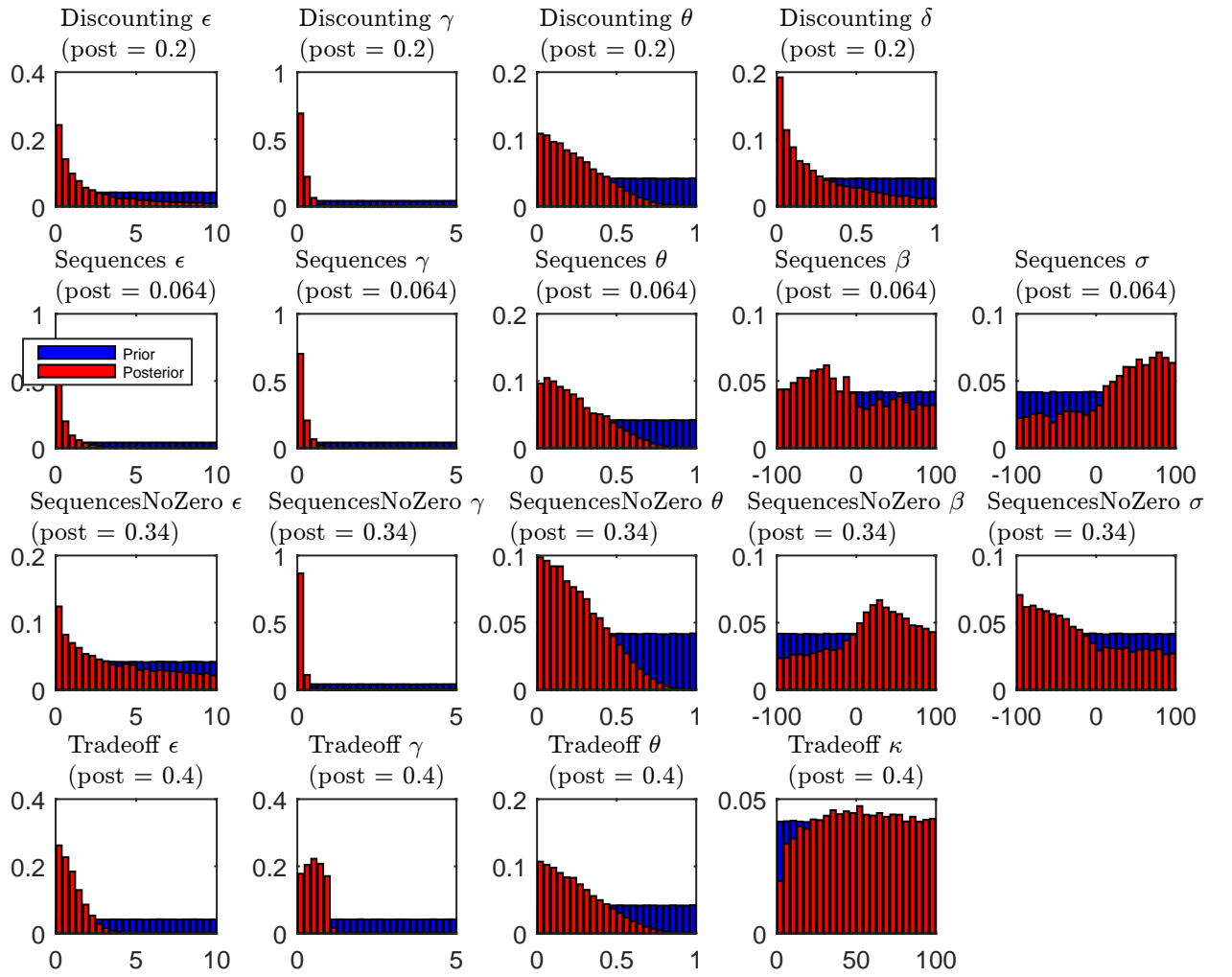

Supplement: Supplementary file 1 [file Scholten_Individuals.zip › plots/e29_p153_eg2_priors_and_posteriors.pdf]

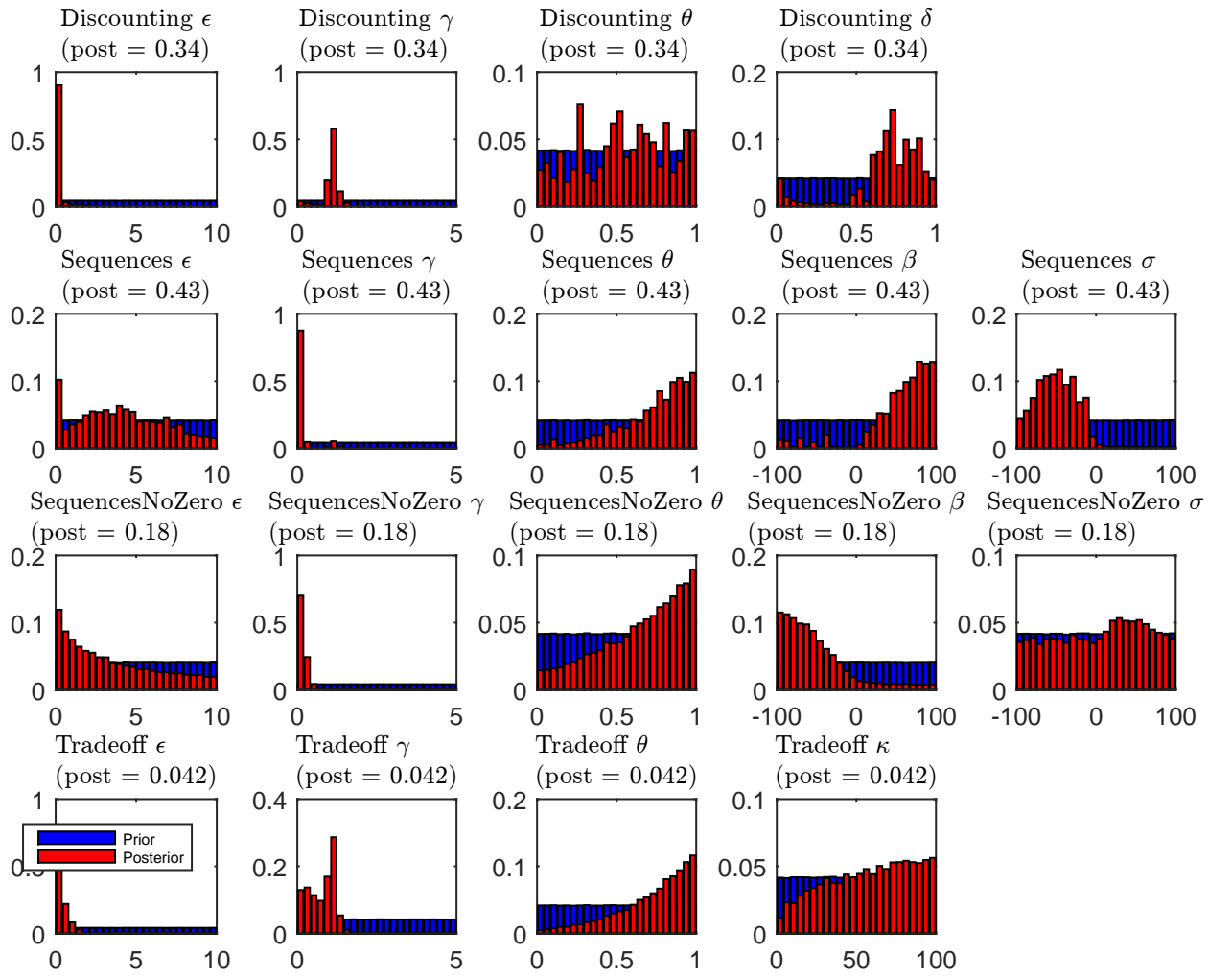

Supplement: Supplementary file 1 [file Scholten_Individuals.zip › plots/e29_p154_eg2_priors_and_posteriors.pdf]

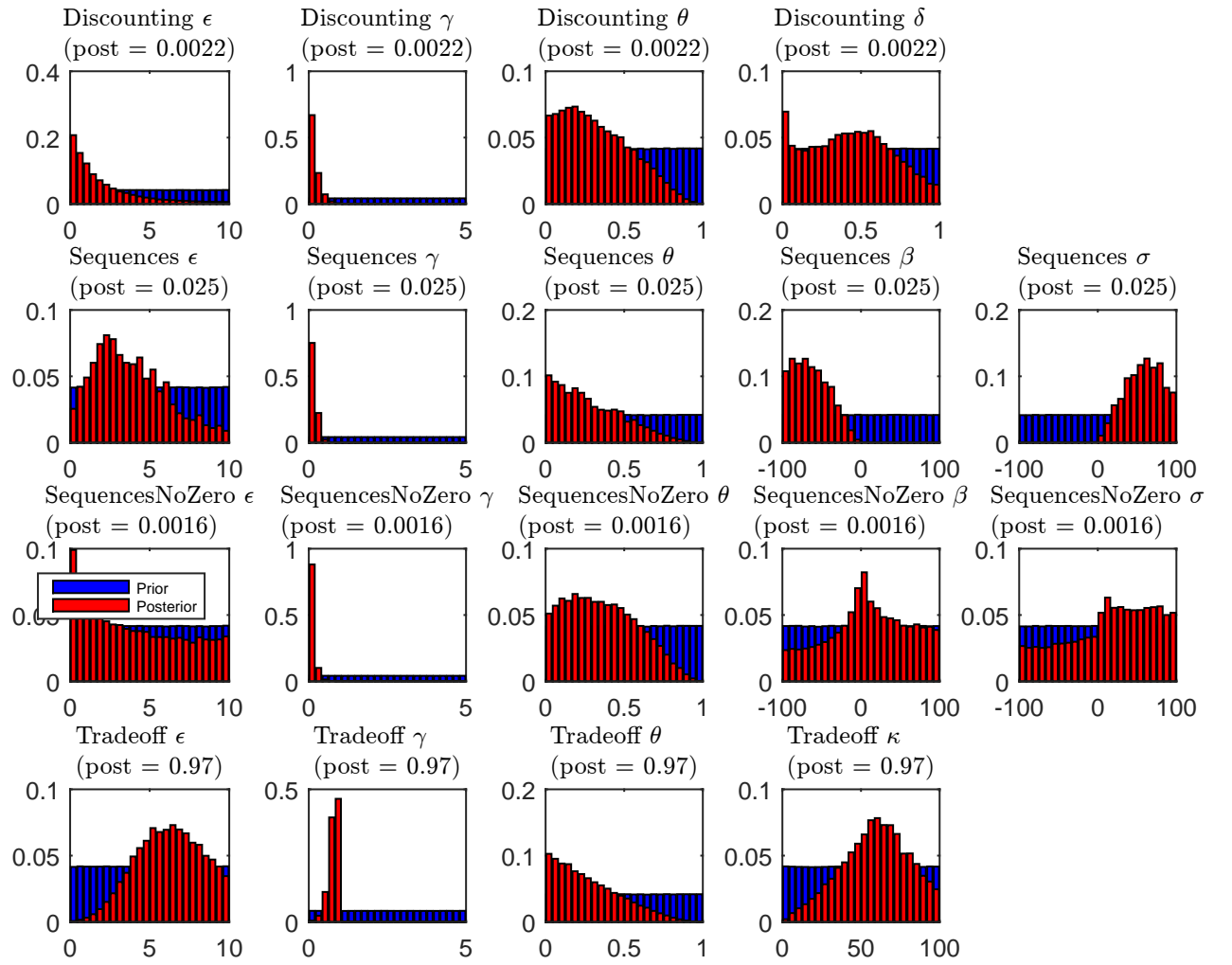

Supplement: Supplementary file 1 [file Scholten_Individuals.zip › plots/e29_p155_eg2_priors_and_posteriors.pdf]

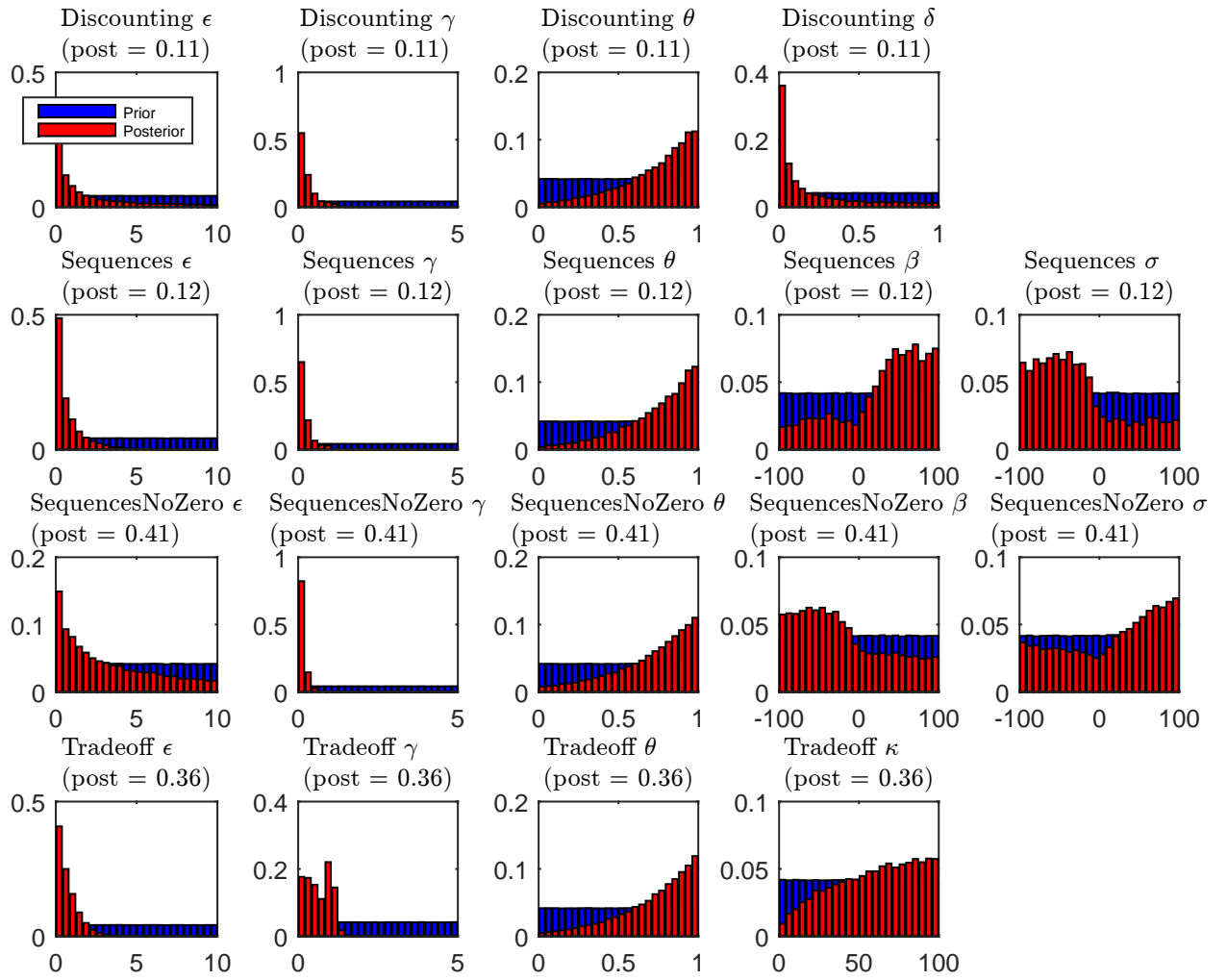

Supplement: Supplementary file 1 [file Scholten_Individuals.zip › plots/e29_p156_eg2_priors_and_posteriors.pdf]

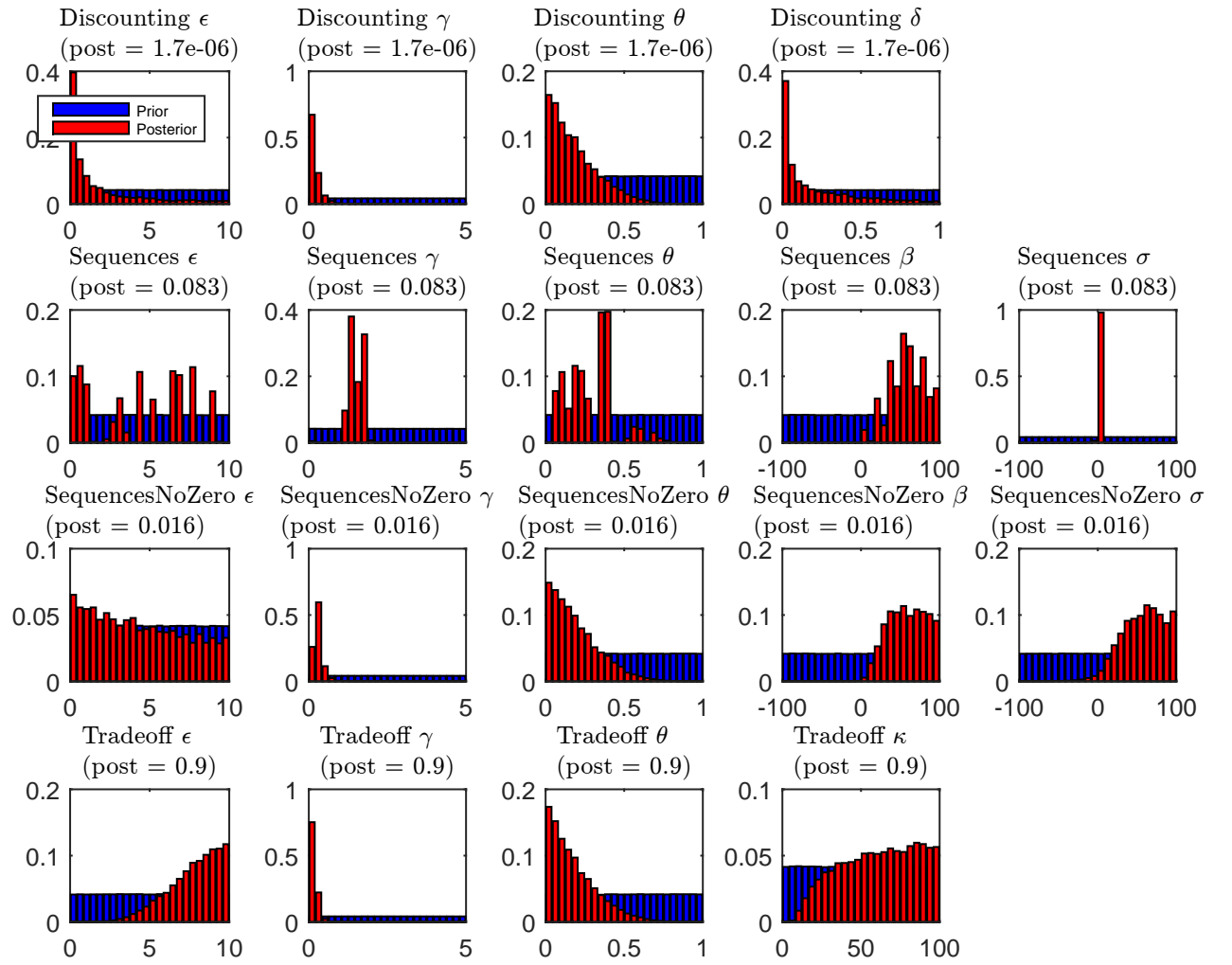

Supplement: Supplementary file 1 [file Scholten_Individuals.zip › plots/e29_p157_eg2_priors_and_posteriors.pdf]

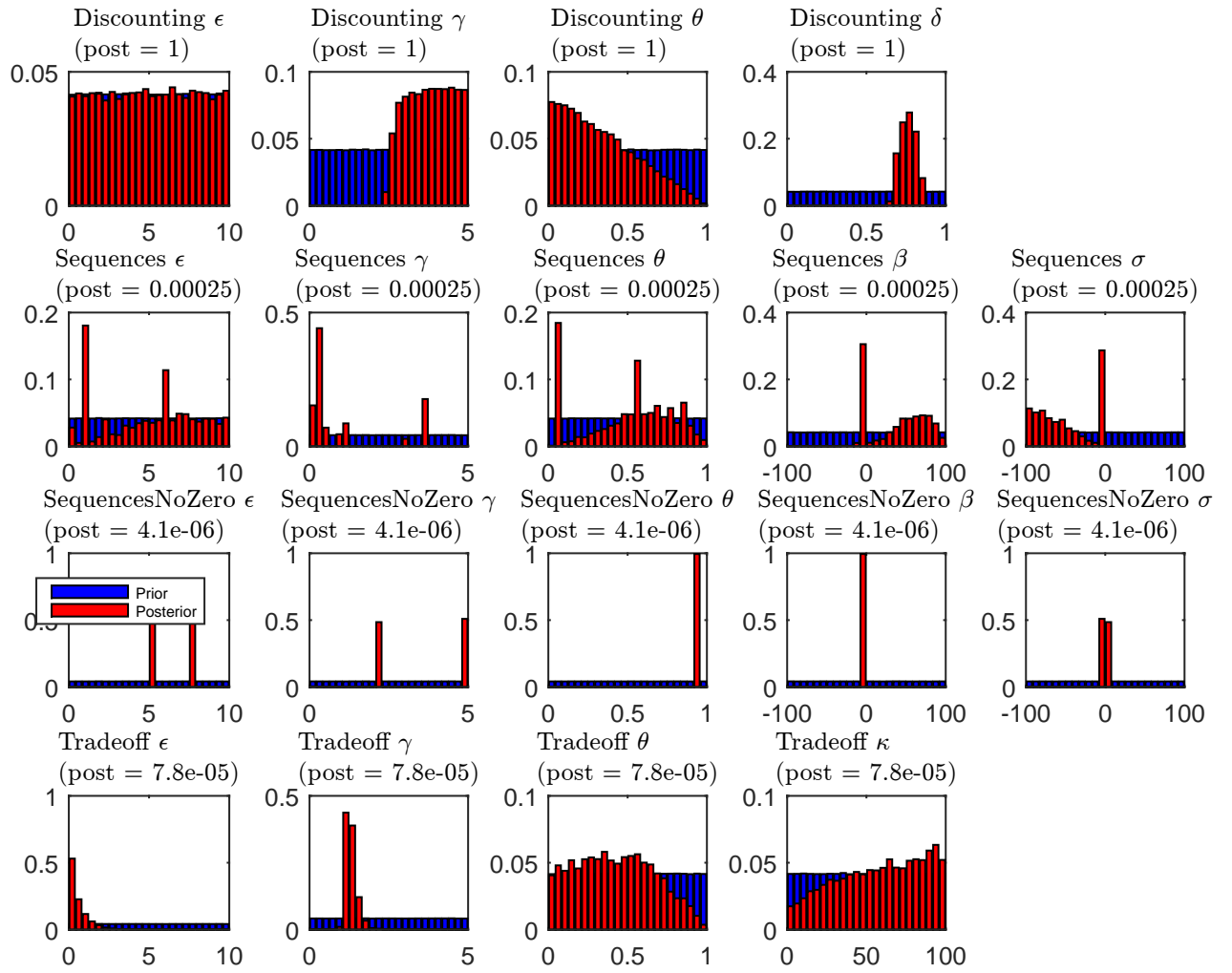

Supplement: Supplementary file 1 [file Scholten_Individuals.zip › plots/e29_p158_eg2_priors_and_posteriors.pdf]

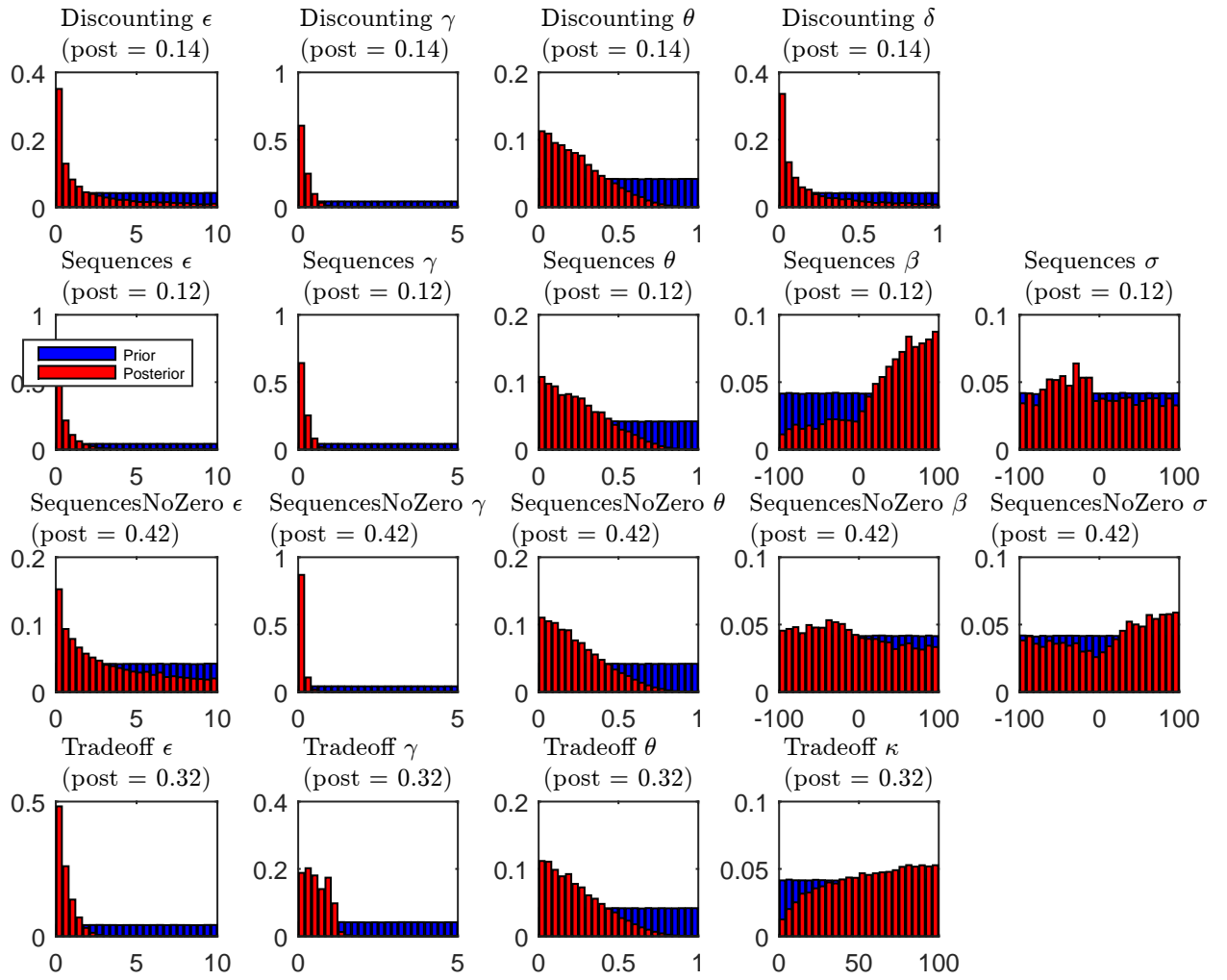

Supplement: Supplementary file 1 [file Scholten_Individuals.zip › plots/e29_p159_eg2_priors_and_posteriors.pdf]

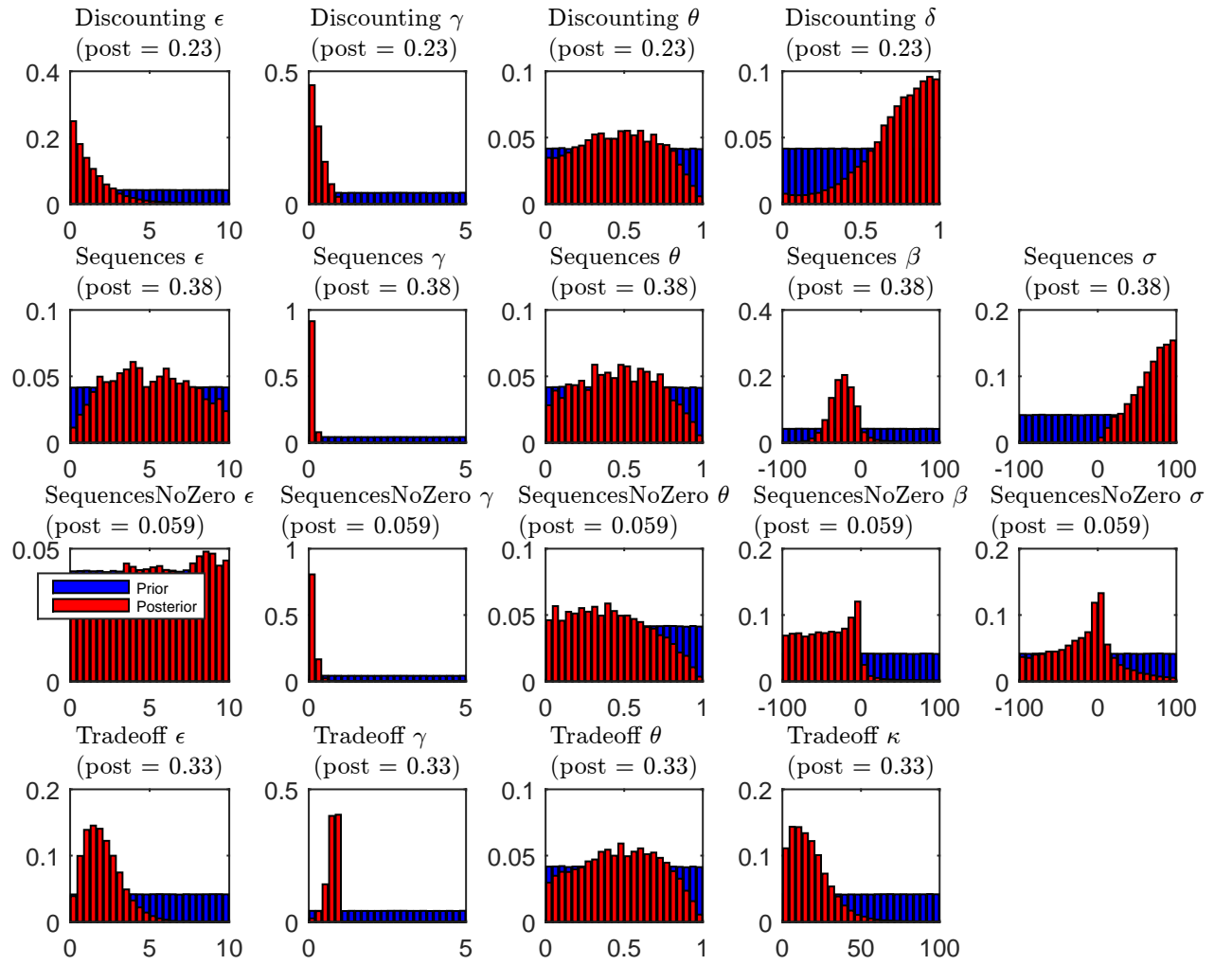

Supplement: Supplementary file 1 [file Scholten_Individuals.zip › plots/e29_p16_eg2_priors_and_posteriors.pdf]

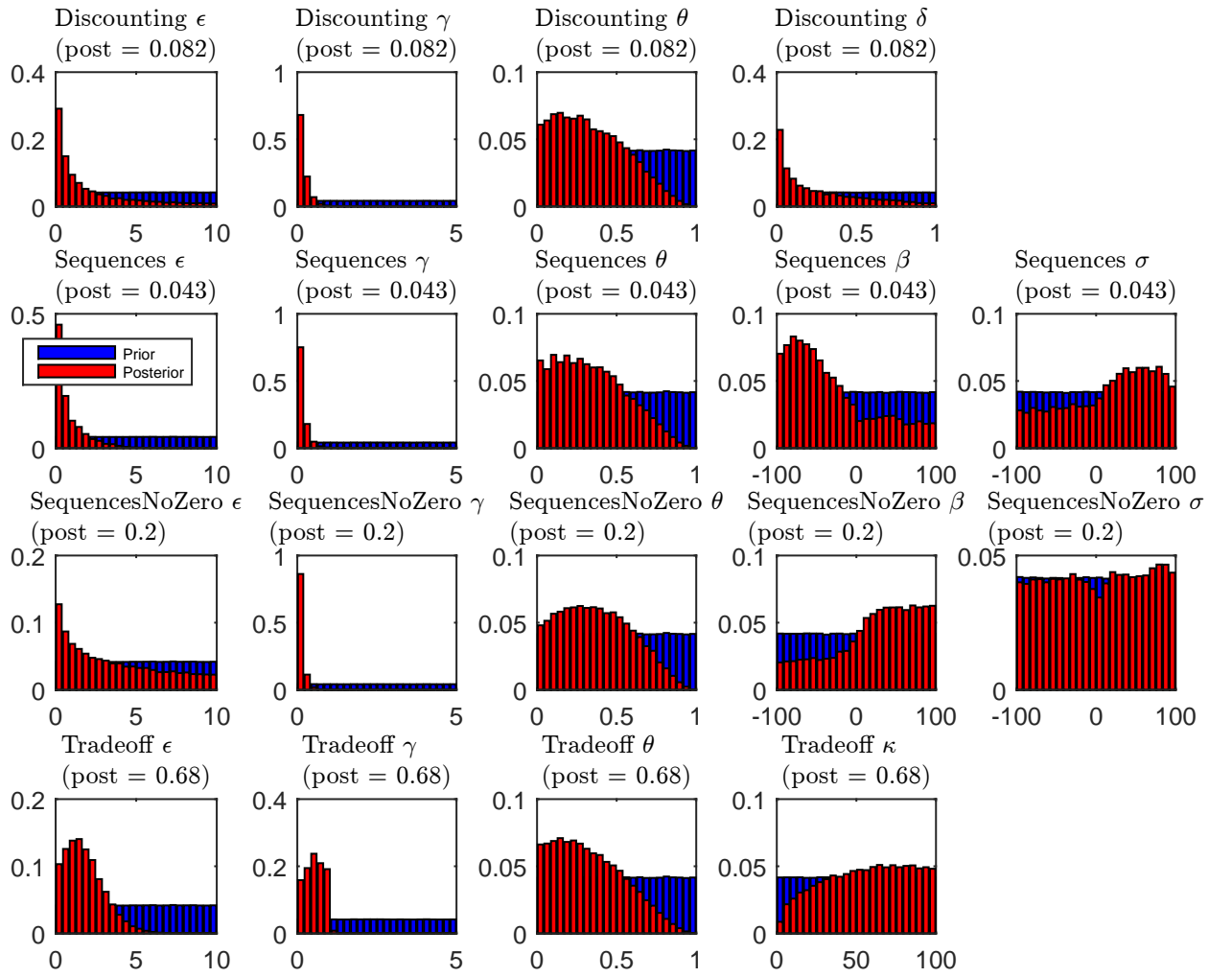

Supplement: Supplementary file 1 [file Scholten_Individuals.zip › plots/e29_p160_eg2_priors_and_posteriors.pdf]

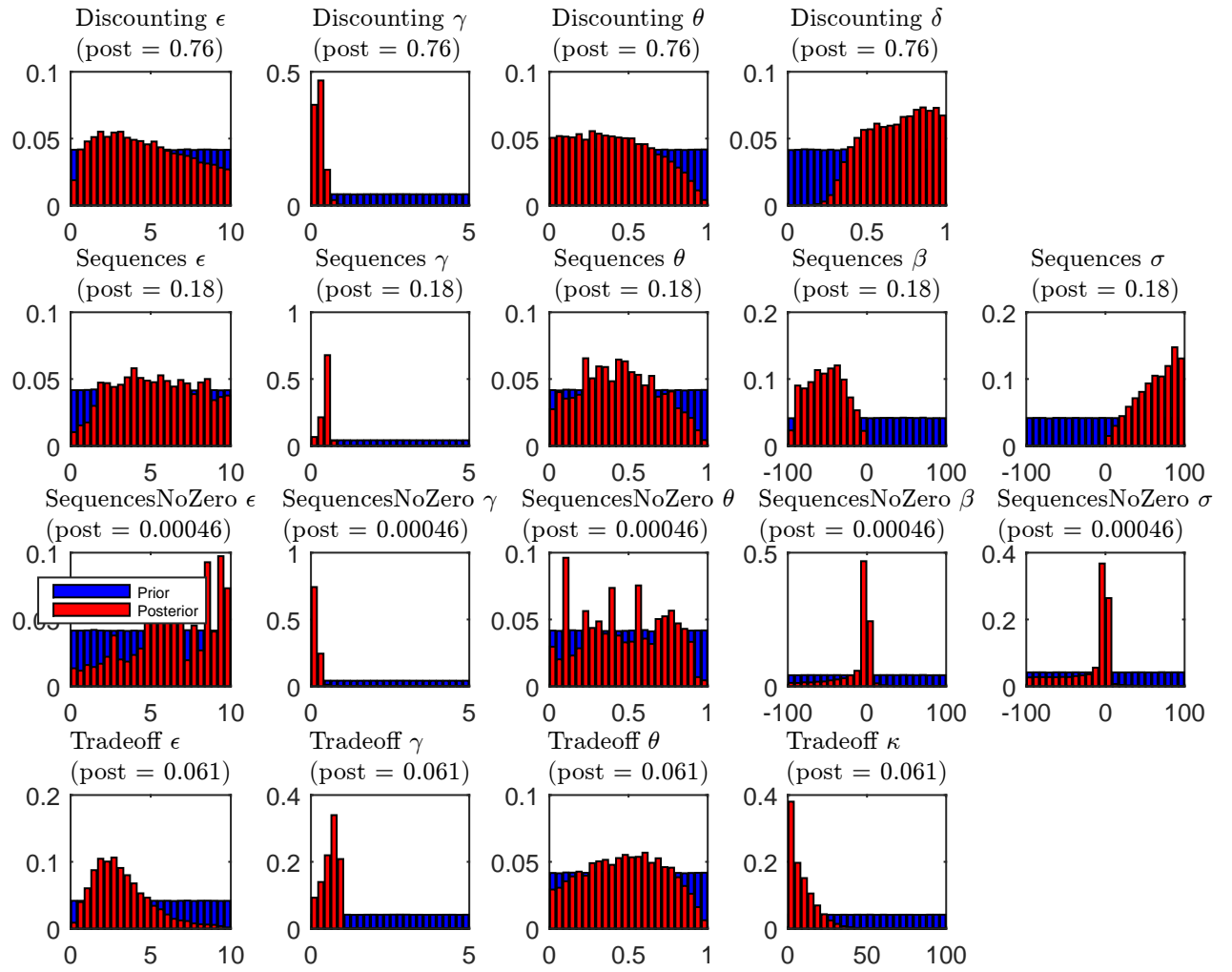

Supplement: Supplementary file 1 [file Scholten_Individuals.zip › plots/e29_p161_eg2_priors_and_posteriors.pdf]

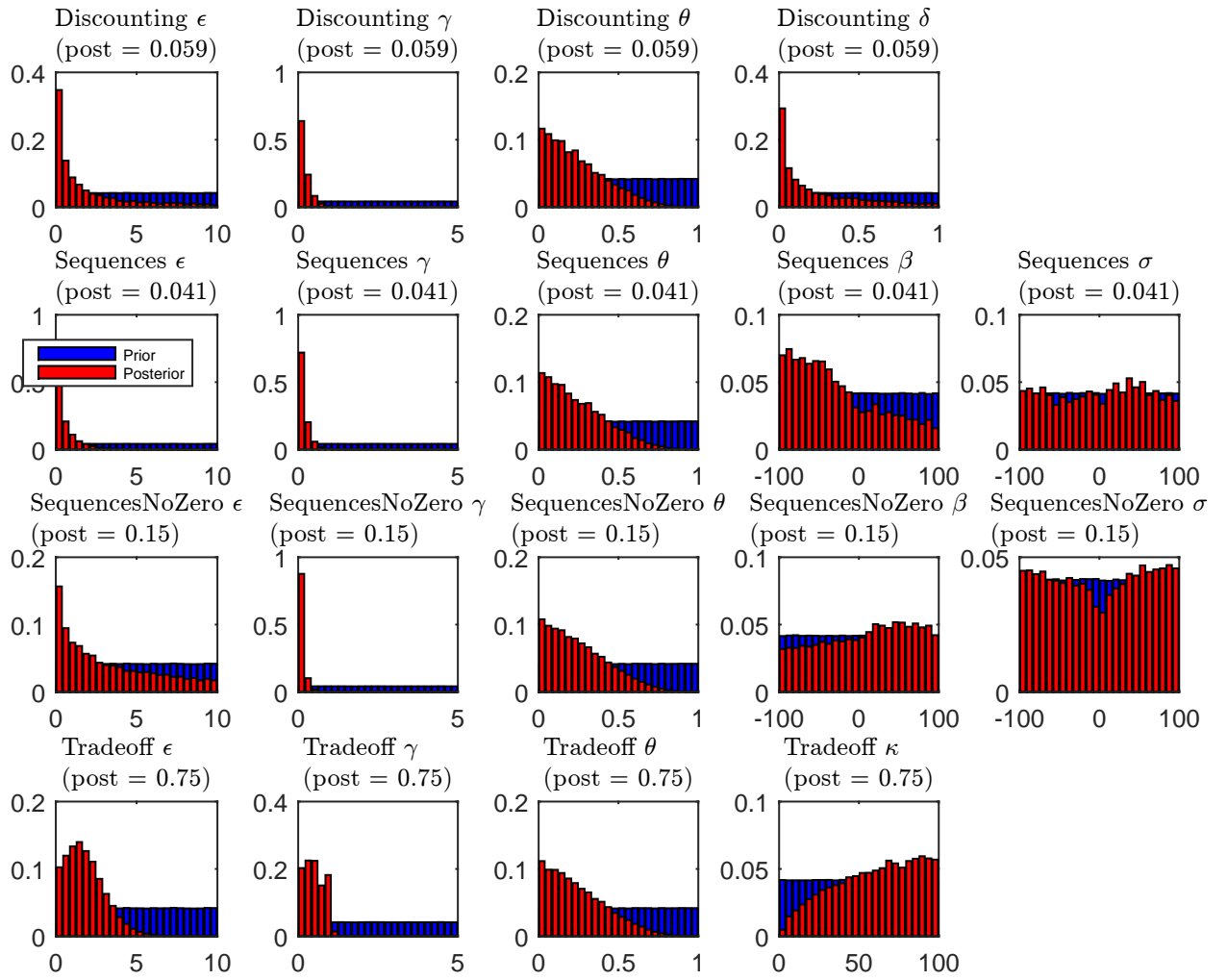

Supplement: Supplementary file 1 [file Scholten_Individuals.zip › plots/e29_p162_eg2_priors_and_posteriors.pdf]

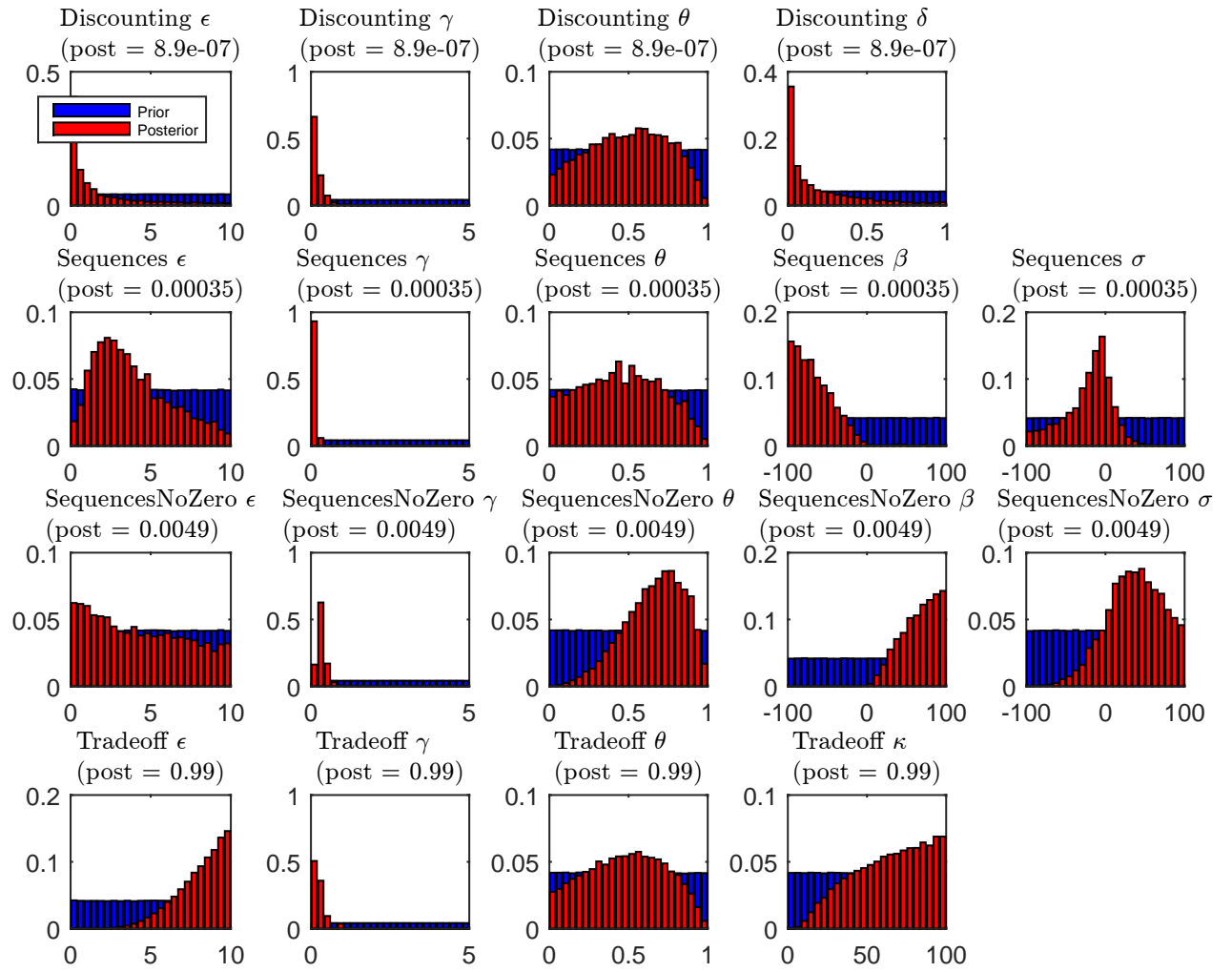

Supplement: Supplementary file 1 [file Scholten_Individuals.zip › plots/e29_p163_eg2_priors_and_posteriors.pdf]

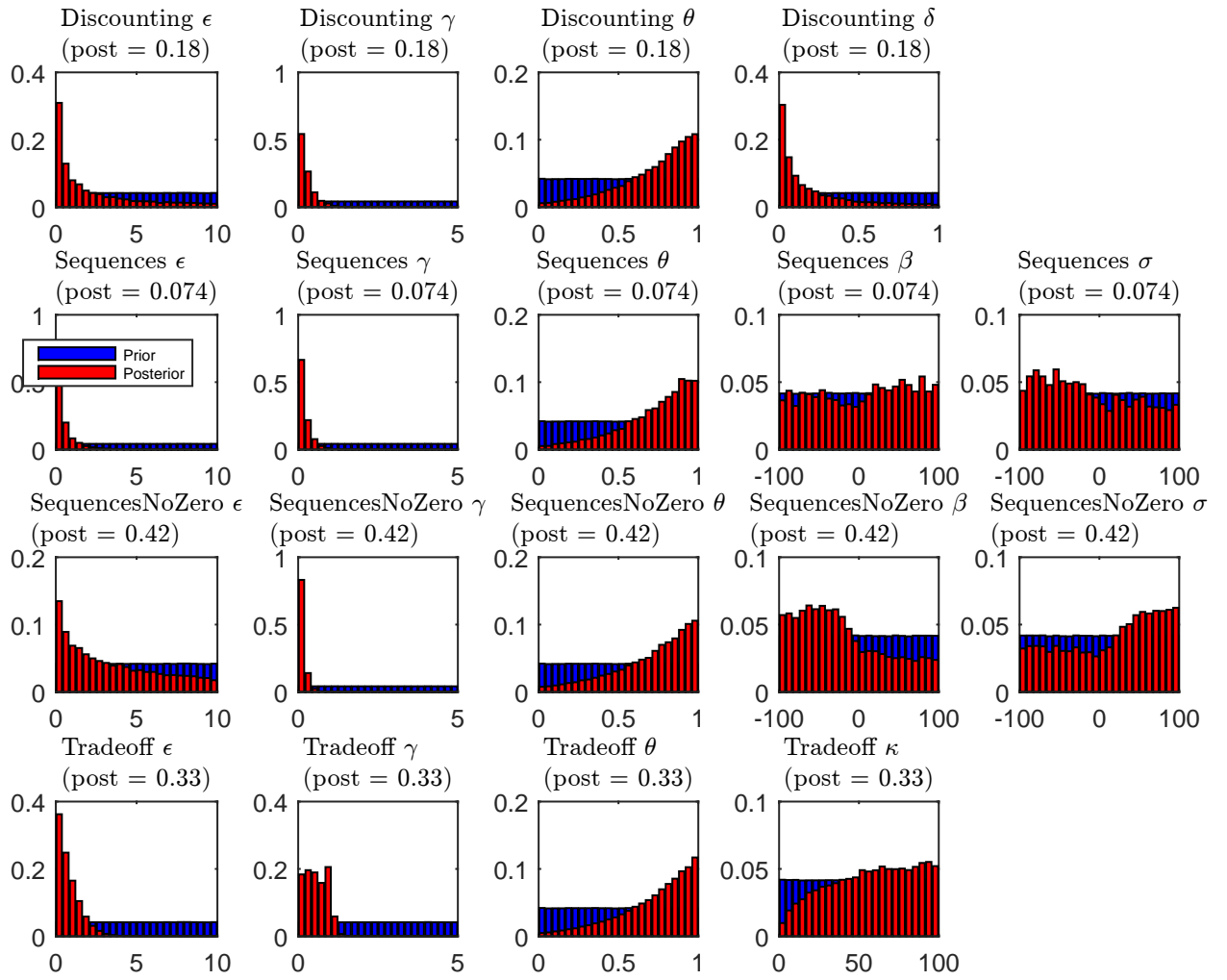

Supplement: Supplementary file 1 [file Scholten_Individuals.zip › plots/e29_p164_eg2_priors_and_posteriors.pdf]

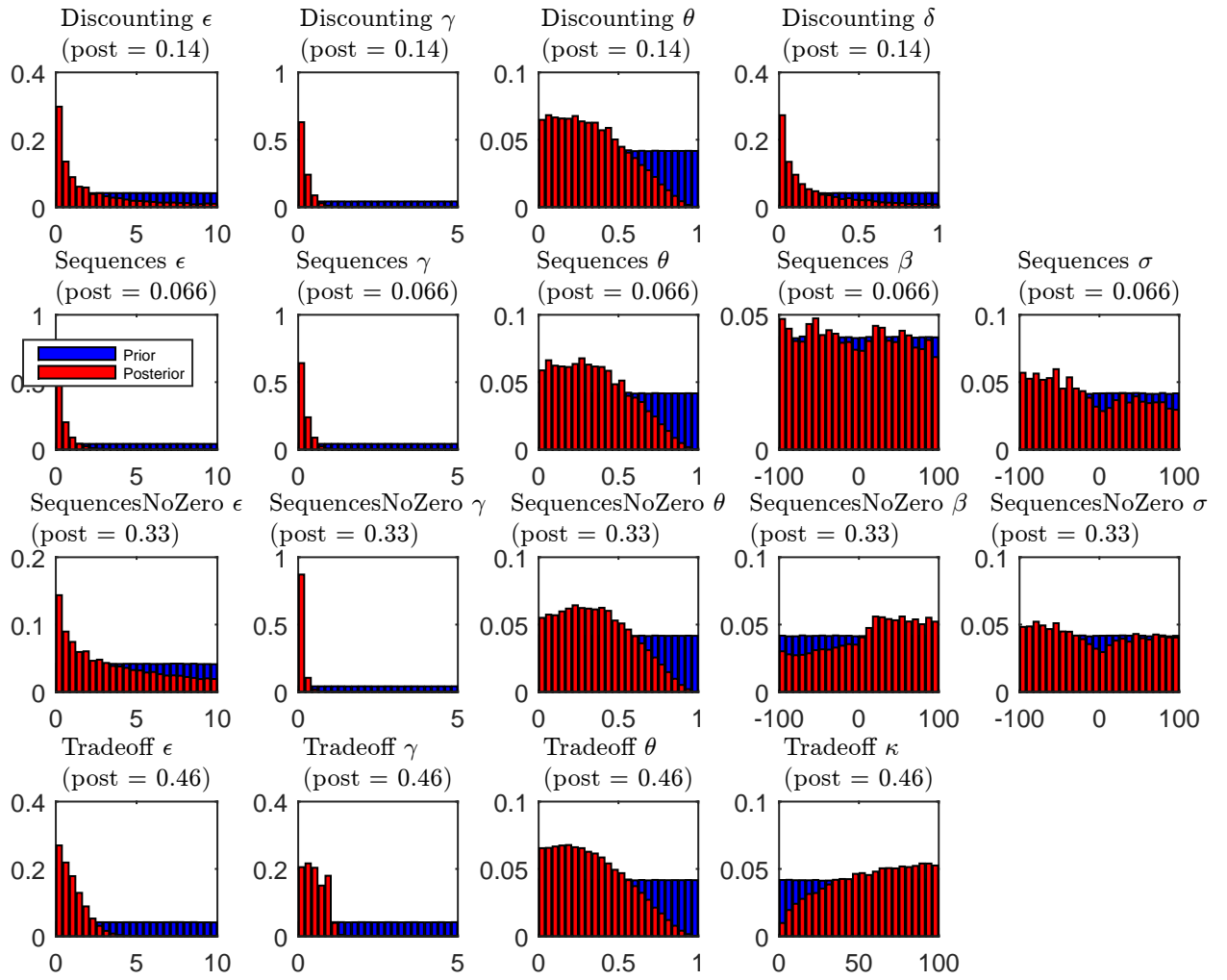

Supplement: Supplementary file 1 [file Scholten_Individuals.zip › plots/e29_p165_eg2_priors_and_posteriors.pdf]

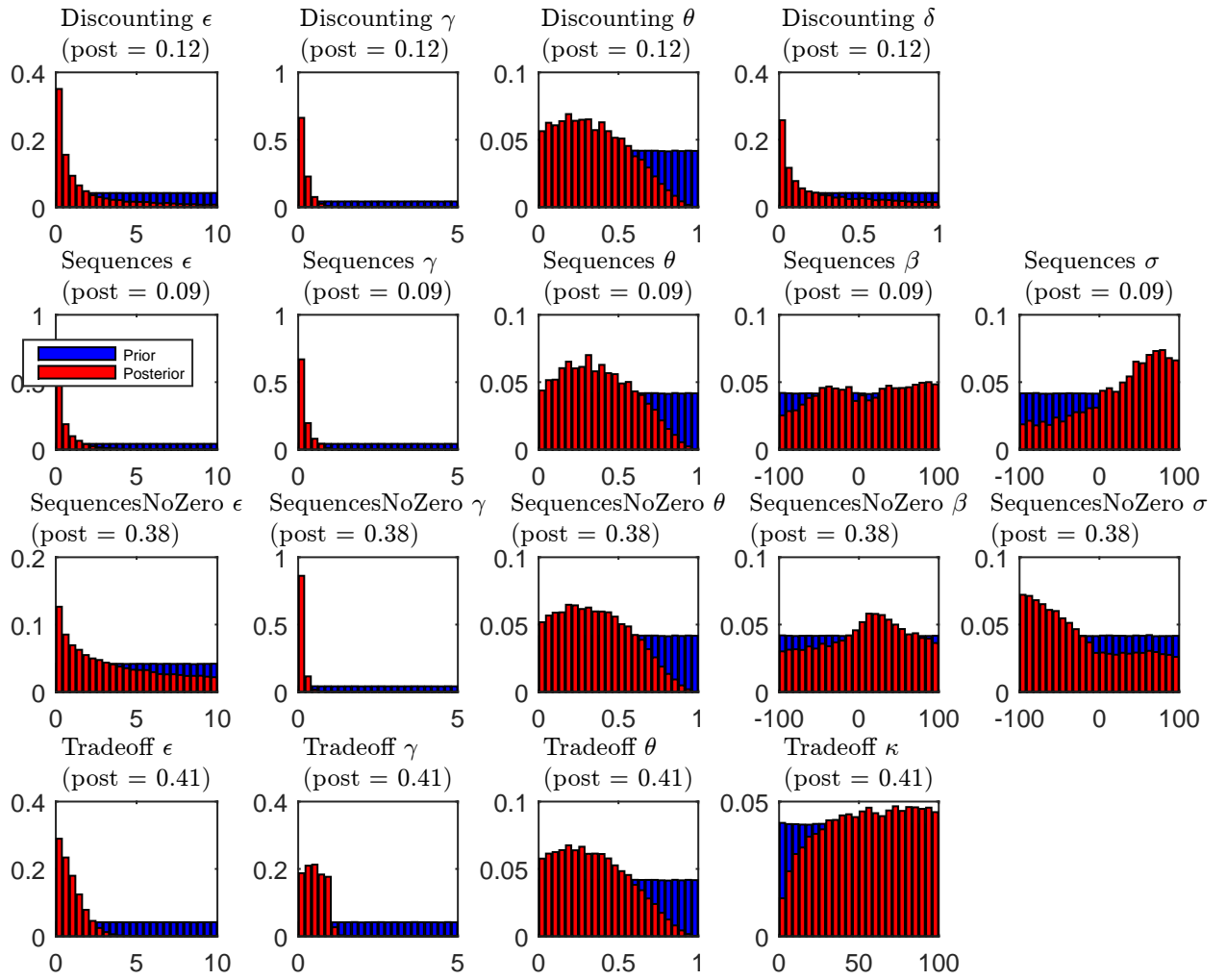

Supplement: Supplementary file 1 [file Scholten_Individuals.zip › plots/e29_p166_eg2_priors_and_posteriors.pdf]

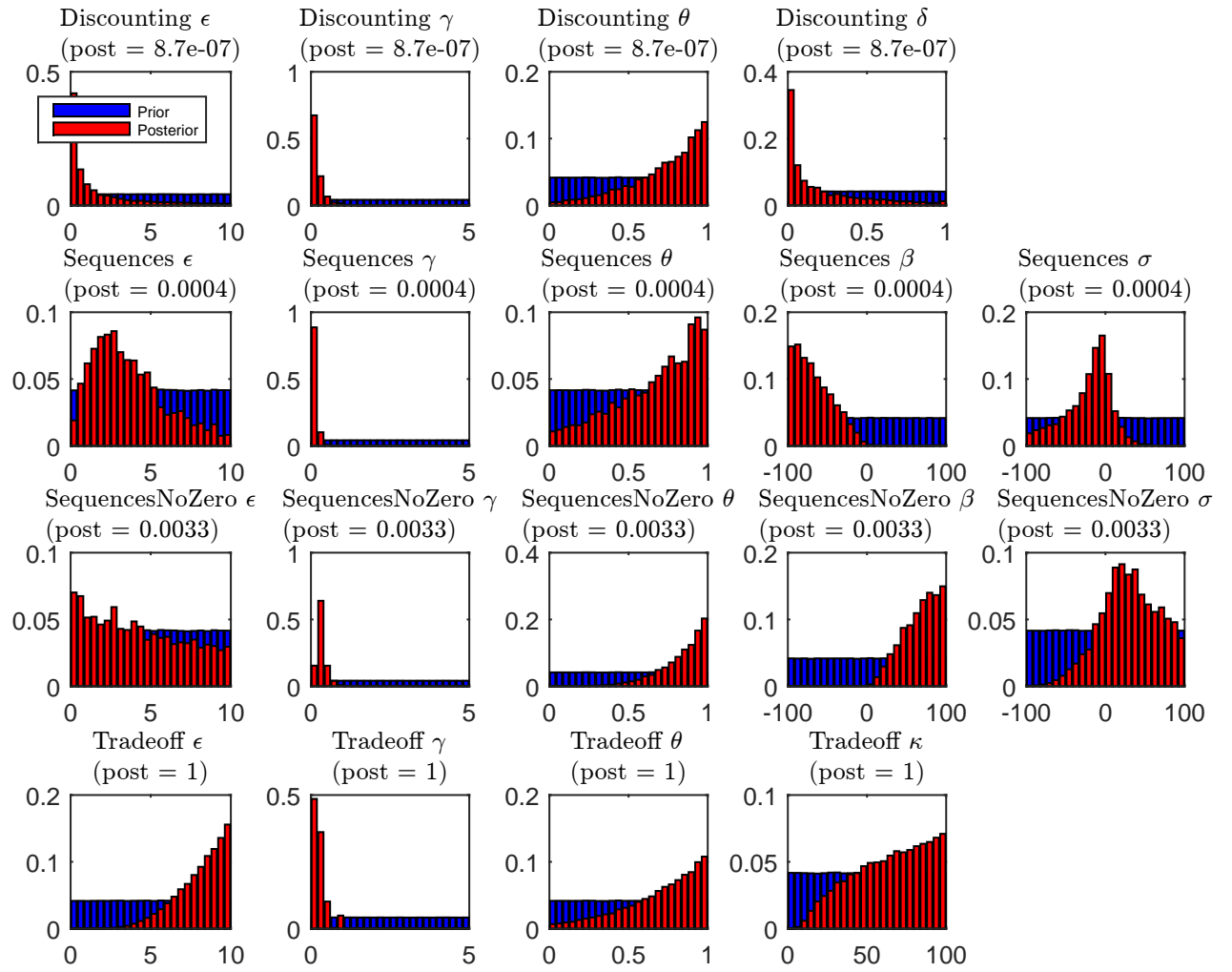

Supplement: Supplementary file 1 [file Scholten_Individuals.zip › plots/e29_p167_eg2_priors_and_posteriors.pdf]

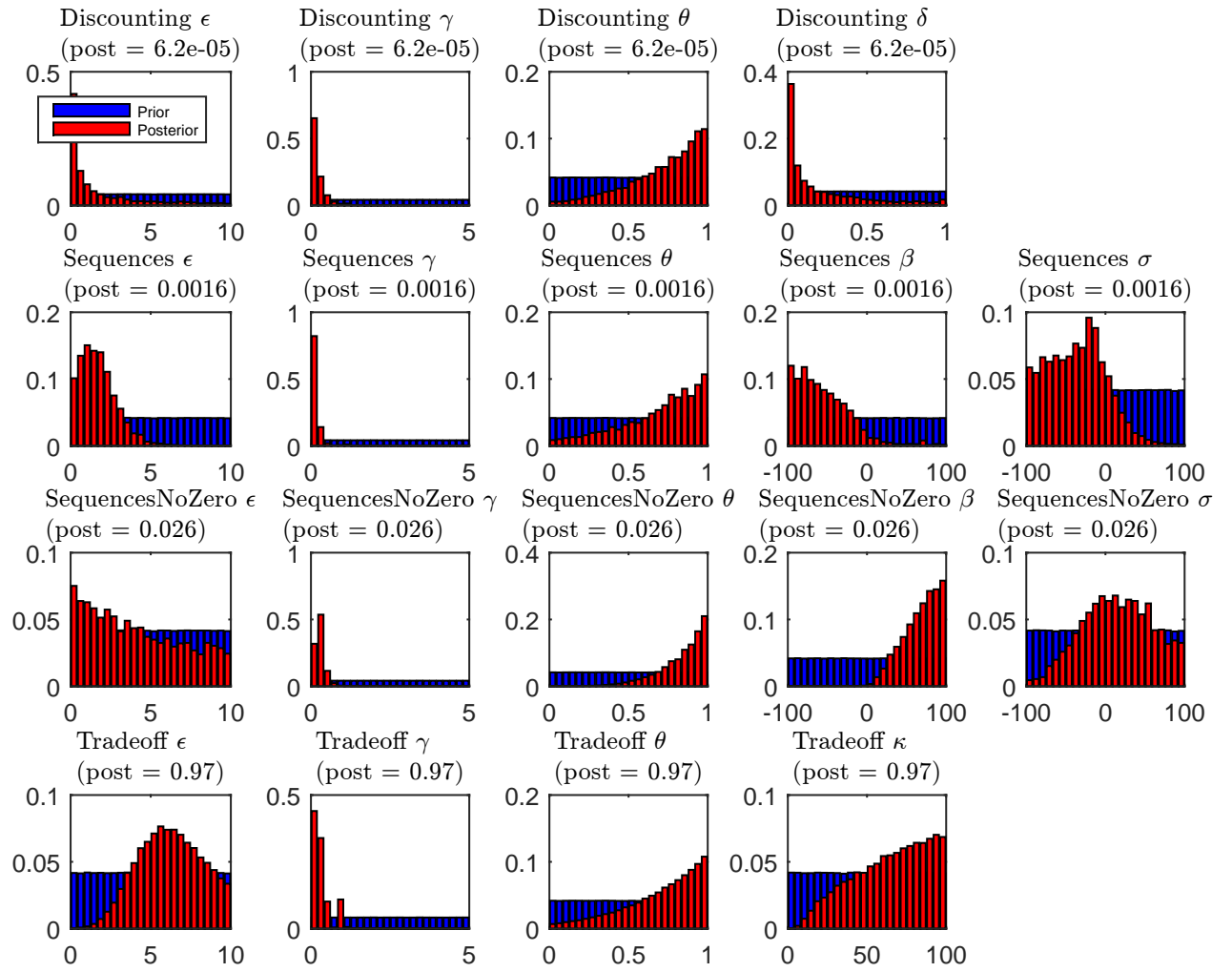

Supplement: Supplementary file 1 [file Scholten_Individuals.zip › plots/e29_p168_eg2_priors_and_posteriors.pdf]

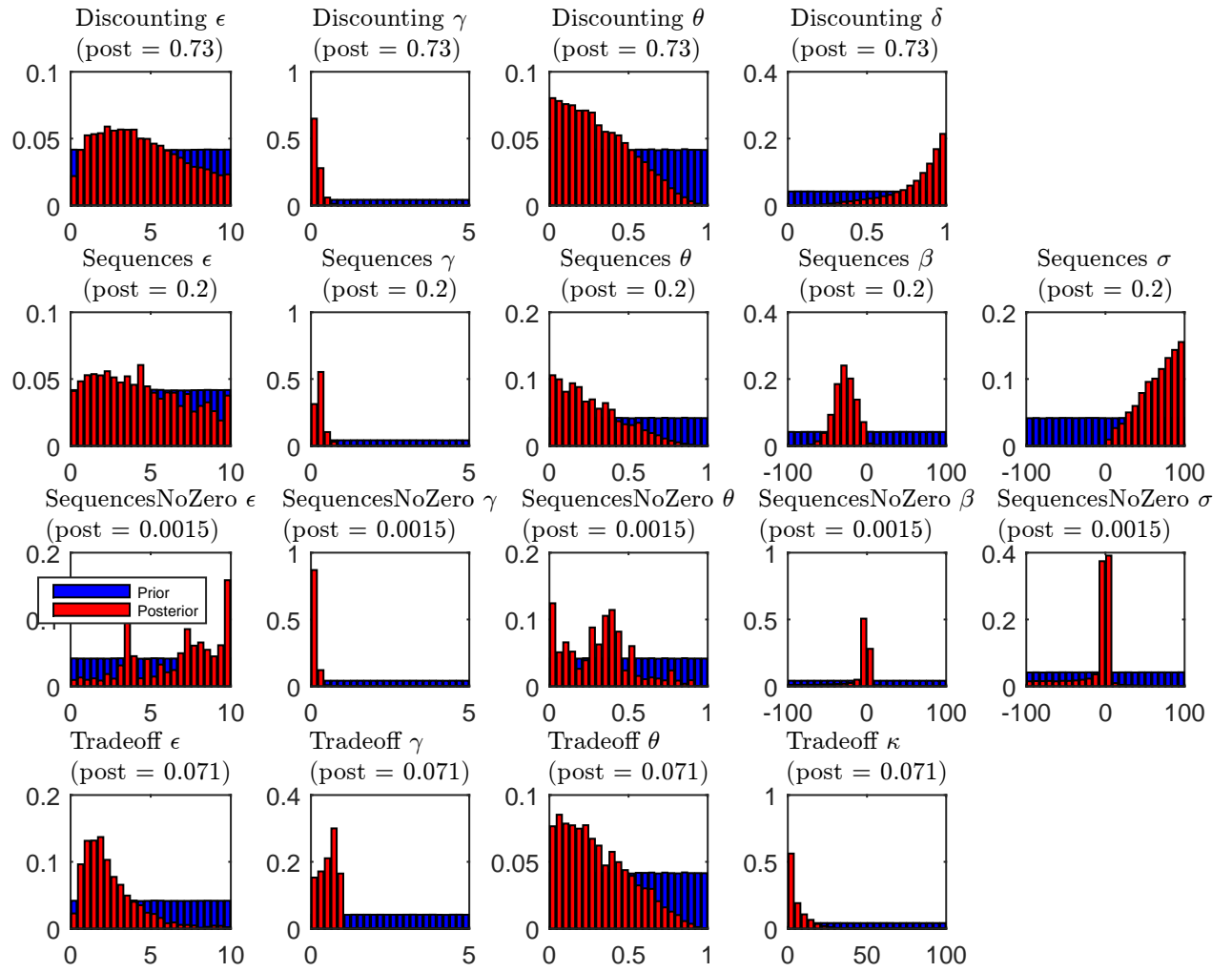

Supplement: Supplementary file 1 [file Scholten_Individuals.zip › plots/e29_p169_eg2_priors_and_posteriors.pdf]

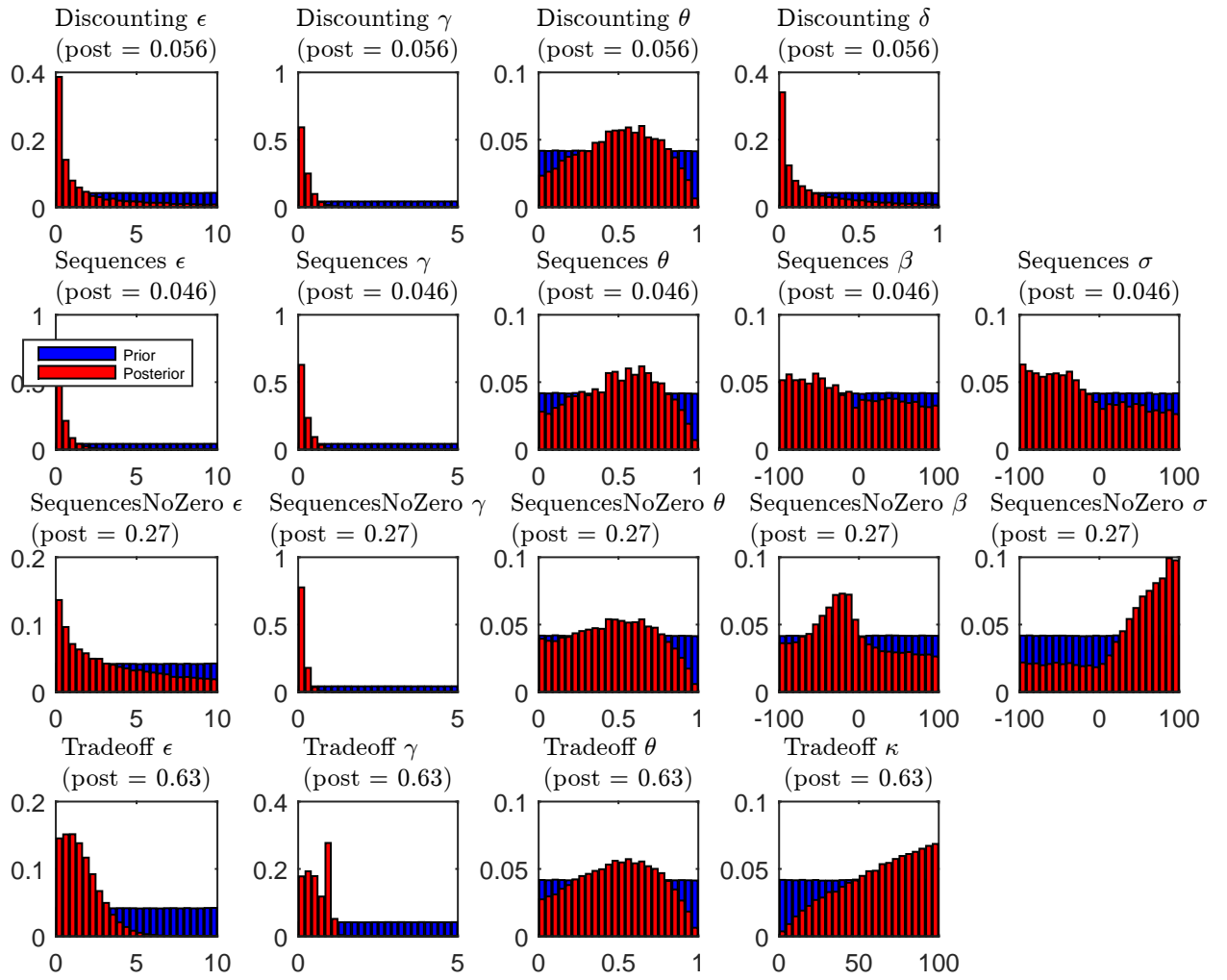

Supplement: Supplementary file 1 [file Scholten_Individuals.zip › plots/e29_p17_eg2_priors_and_posteriors.pdf]

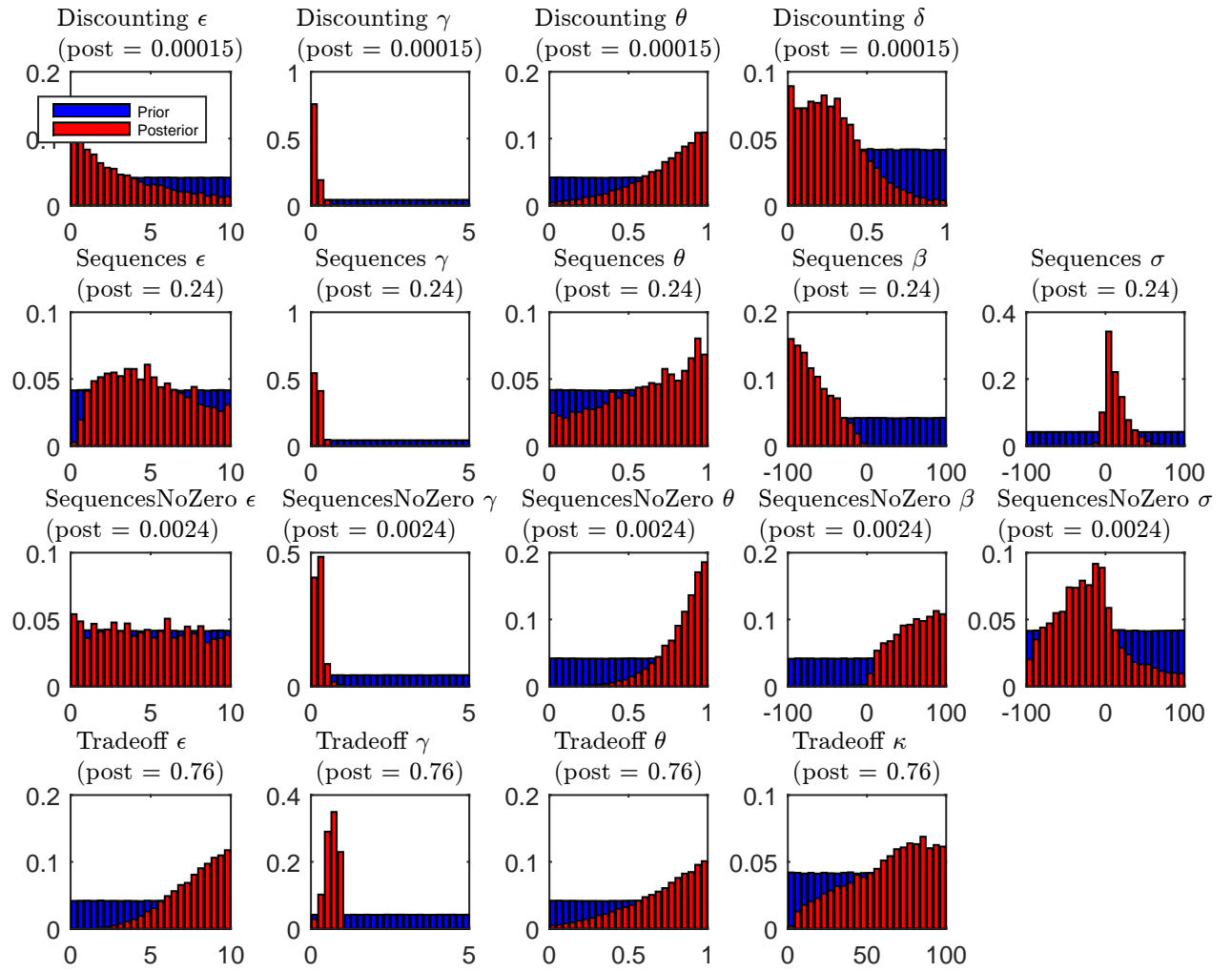

Supplement: Supplementary file 1 [file Scholten_Individuals.zip › plots/e29_p170_eg2_priors_and_posteriors.pdf]

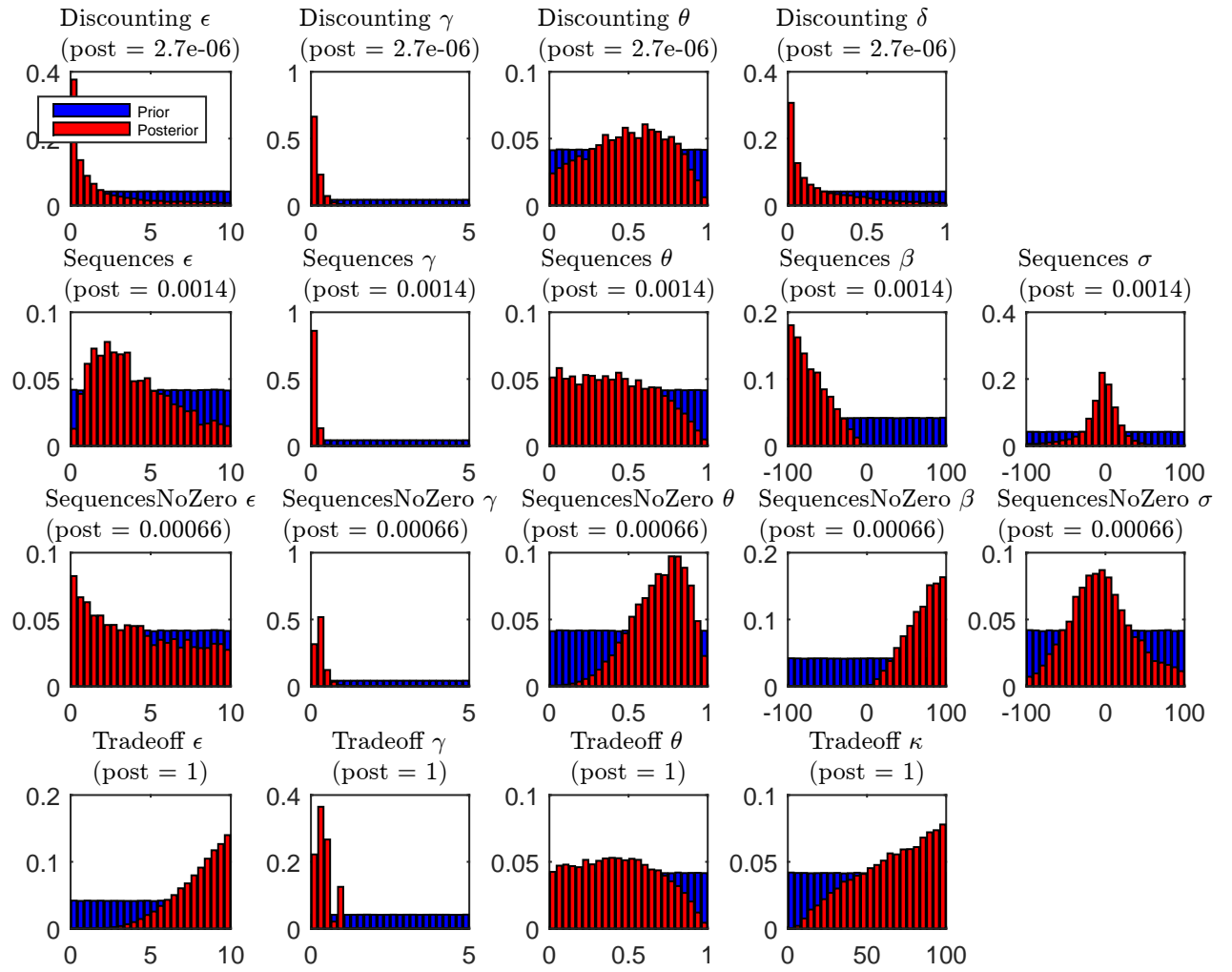

Supplement: Supplementary file 1 [file Scholten_Individuals.zip › plots/e29_p171_eg2_priors_and_posteriors.pdf]

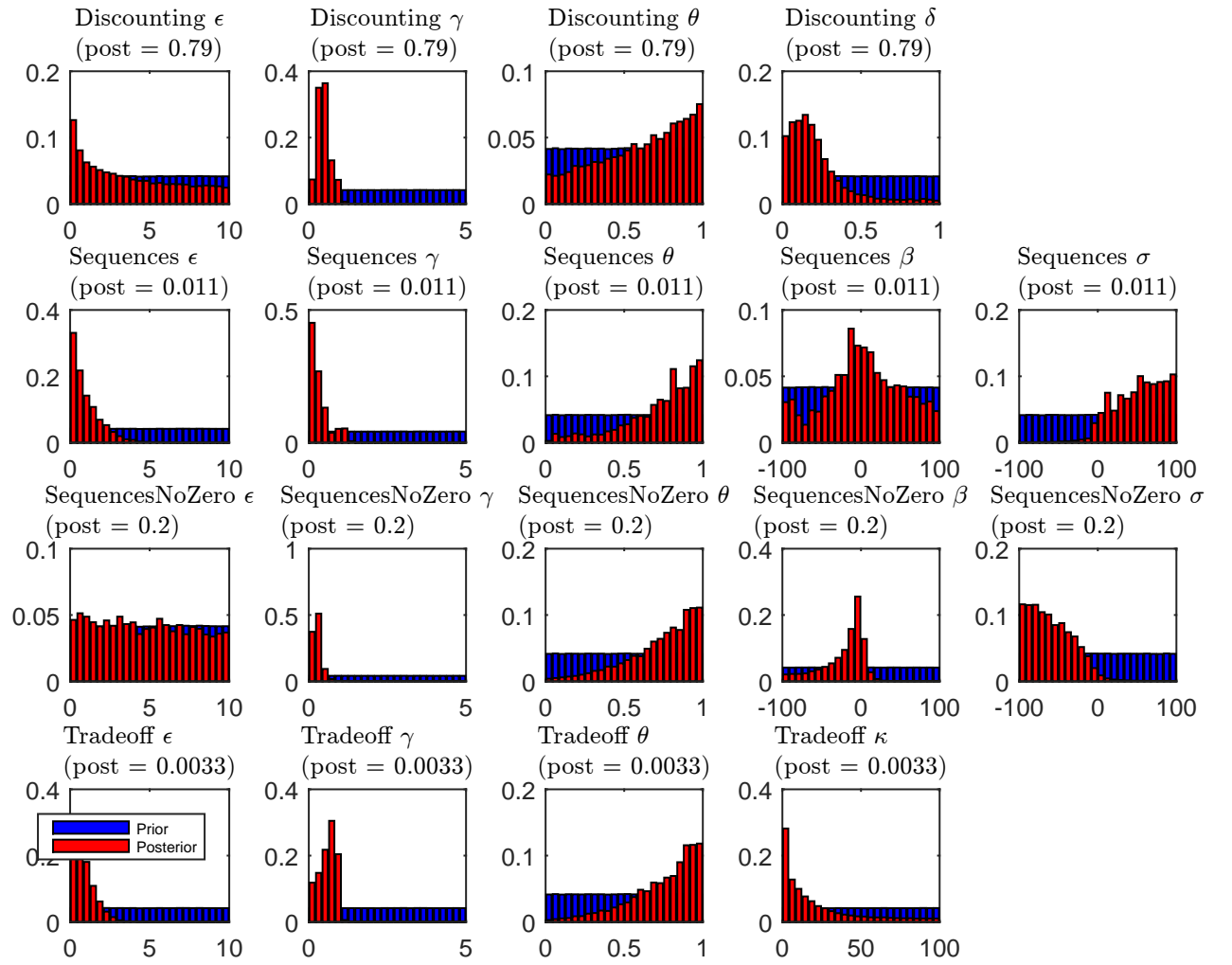

Supplement: Supplementary file 1 [file Scholten_Individuals.zip › plots/e29_p172_eg2_priors_and_posteriors.pdf]

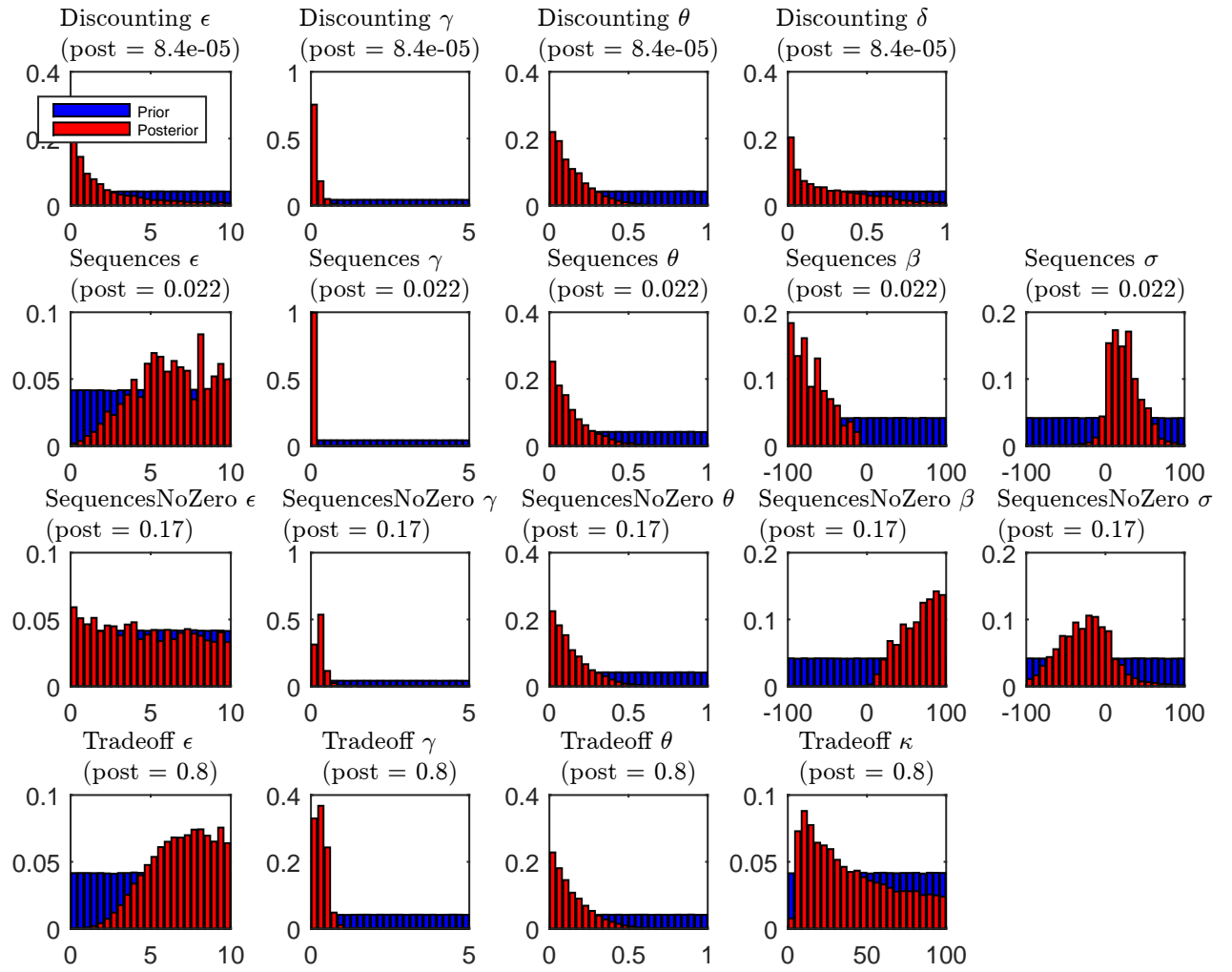

Supplement: Supplementary file 1 [file Scholten_Individuals.zip › plots/e29_p173_eg2_priors_and_posteriors.pdf]

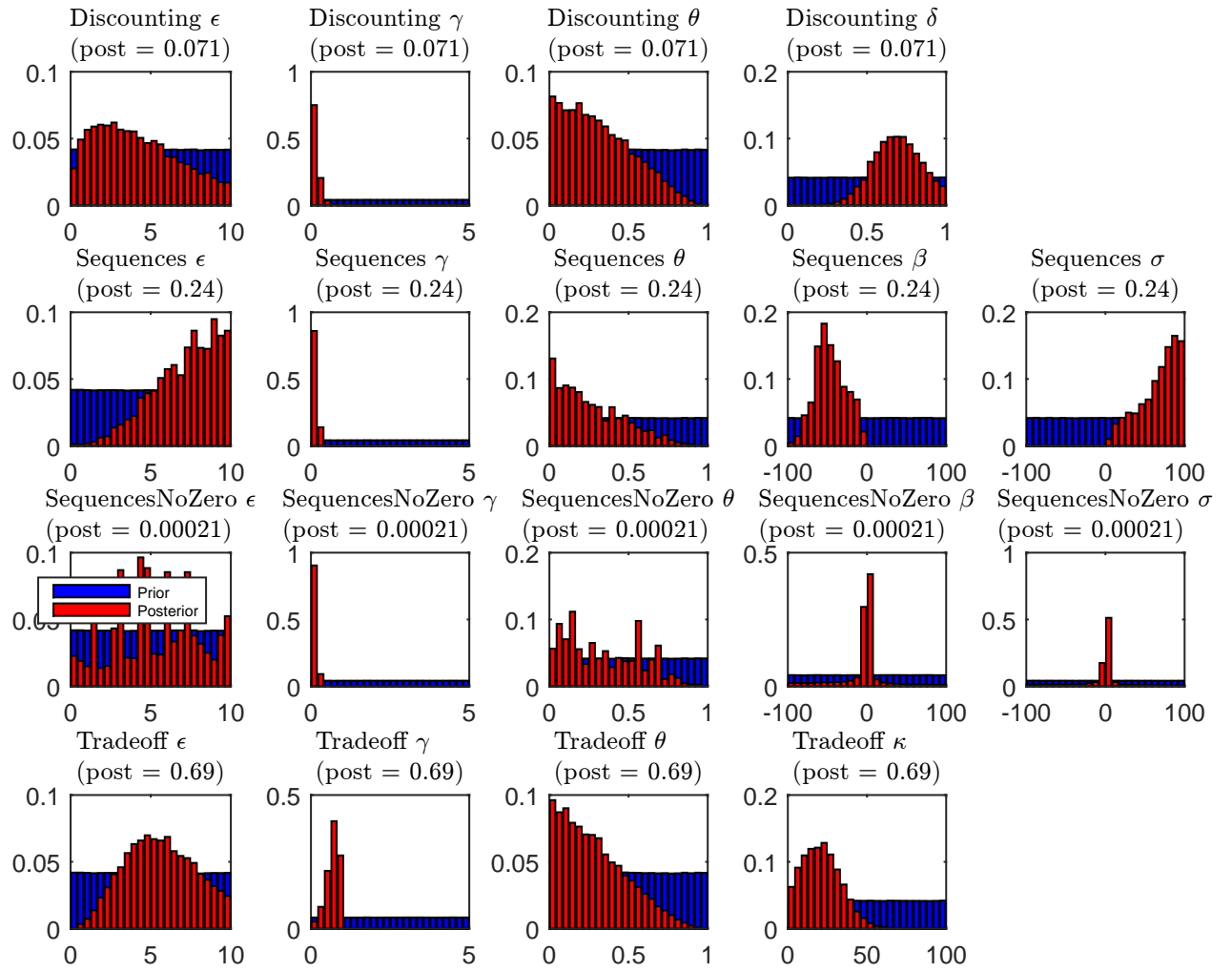

Supplement: Supplementary file 1 [file Scholten_Individuals.zip › plots/e29_p174_eg2_priors_and_posteriors.pdf]

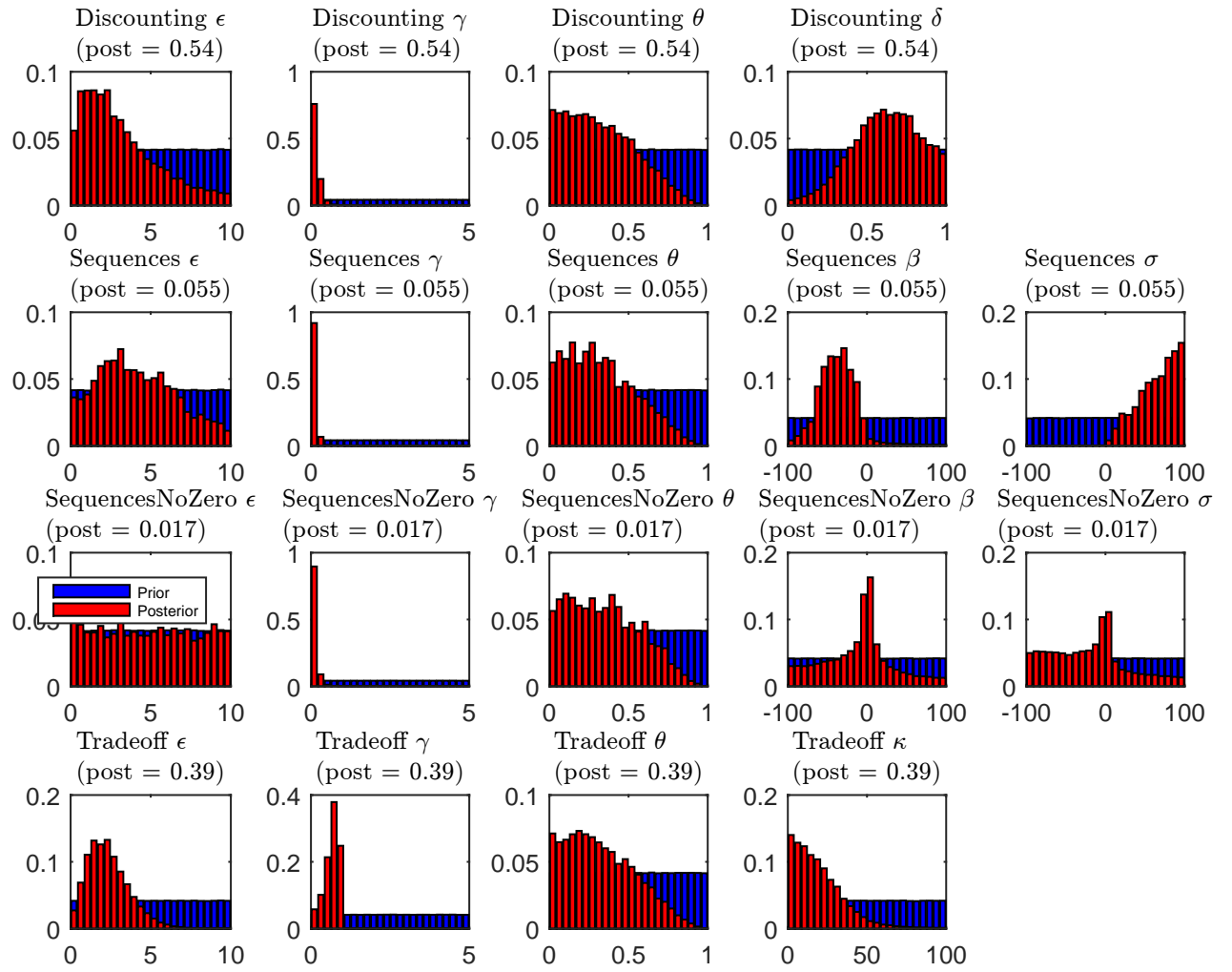

Supplement: Supplementary file 1 [file Scholten_Individuals.zip › plots/e29_p175_eg2_priors_and_posteriors.pdf]

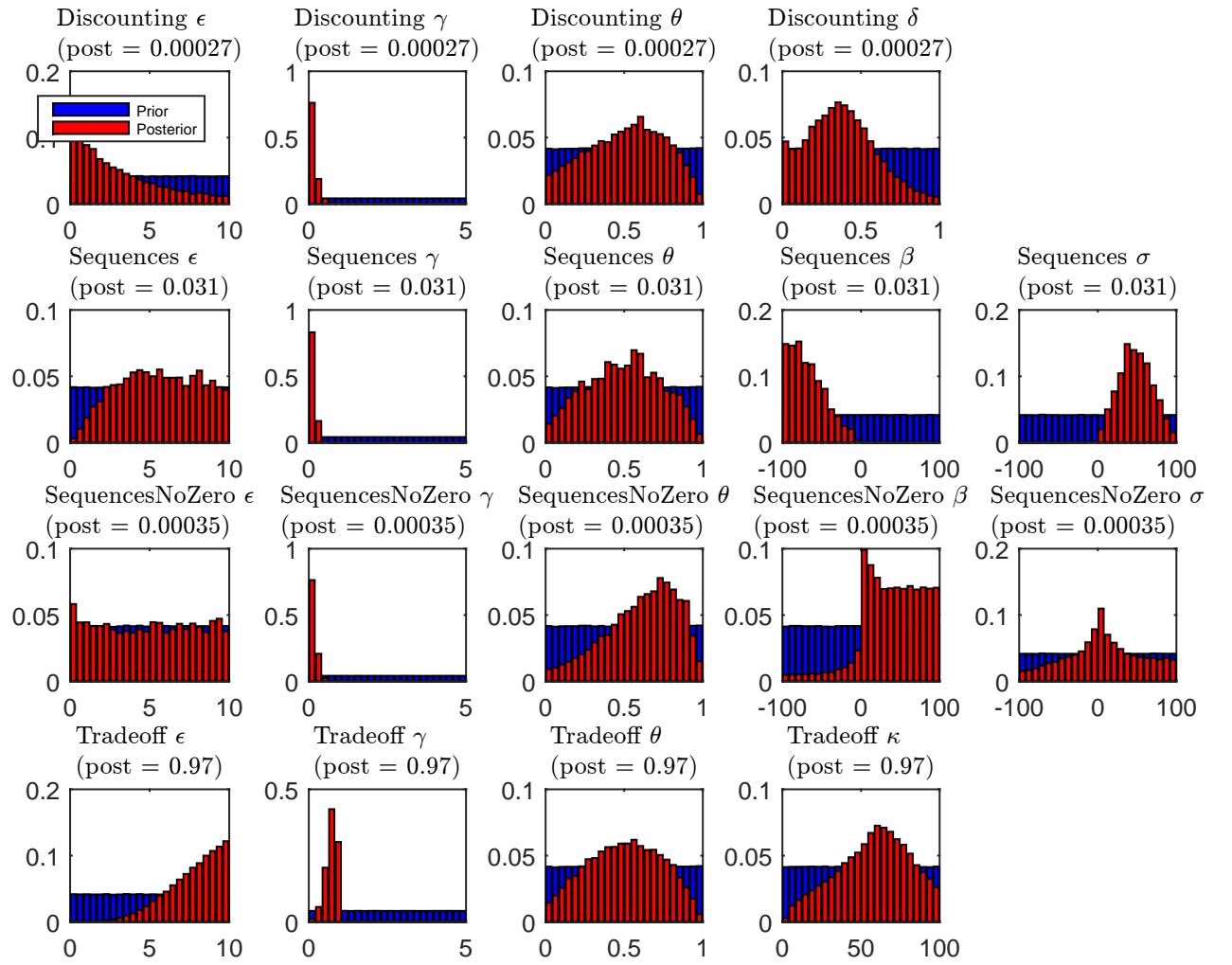

Supplement: Supplementary file 1 [file Scholten_Individuals.zip › plots/e29_p176_eg2_priors_and_posteriors.pdf]

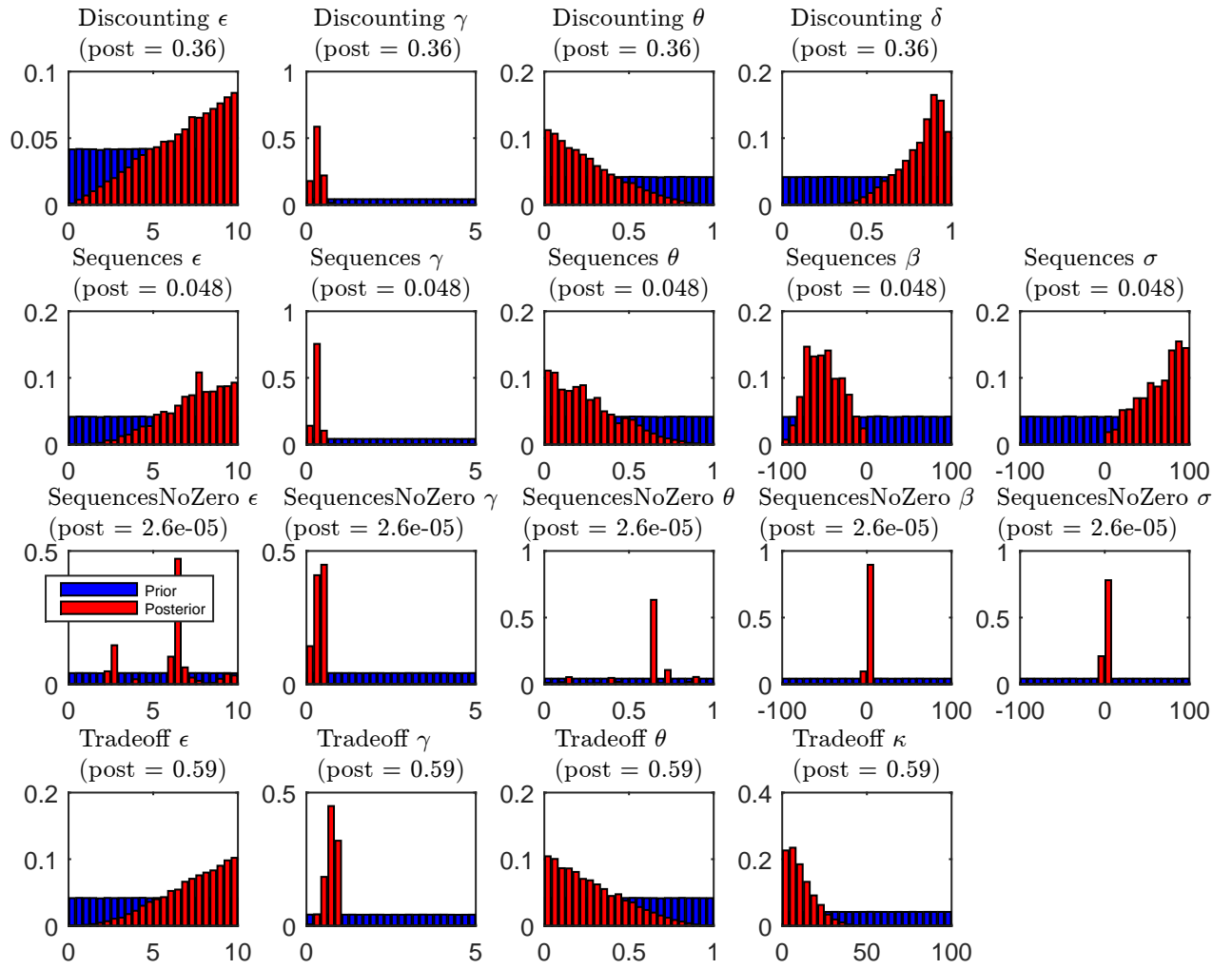

Supplement: Supplementary file 1 [file Scholten_Individuals.zip › plots/e29_p177_eg2_priors_and_posteriors.pdf]

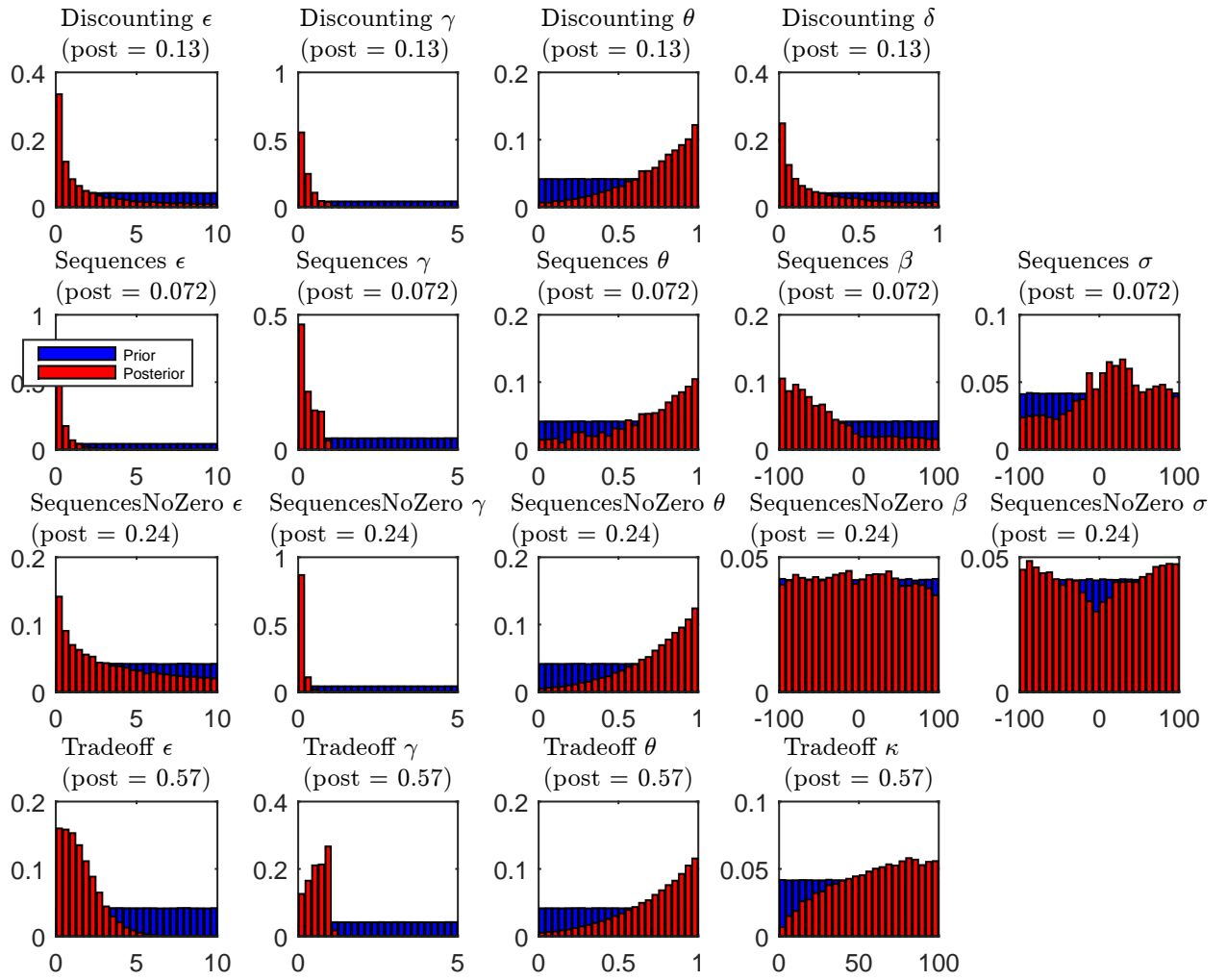

Supplement: Supplementary file 1 [file Scholten_Individuals.zip › plots/e29_p178_eg2_priors_and_posteriors.pdf]

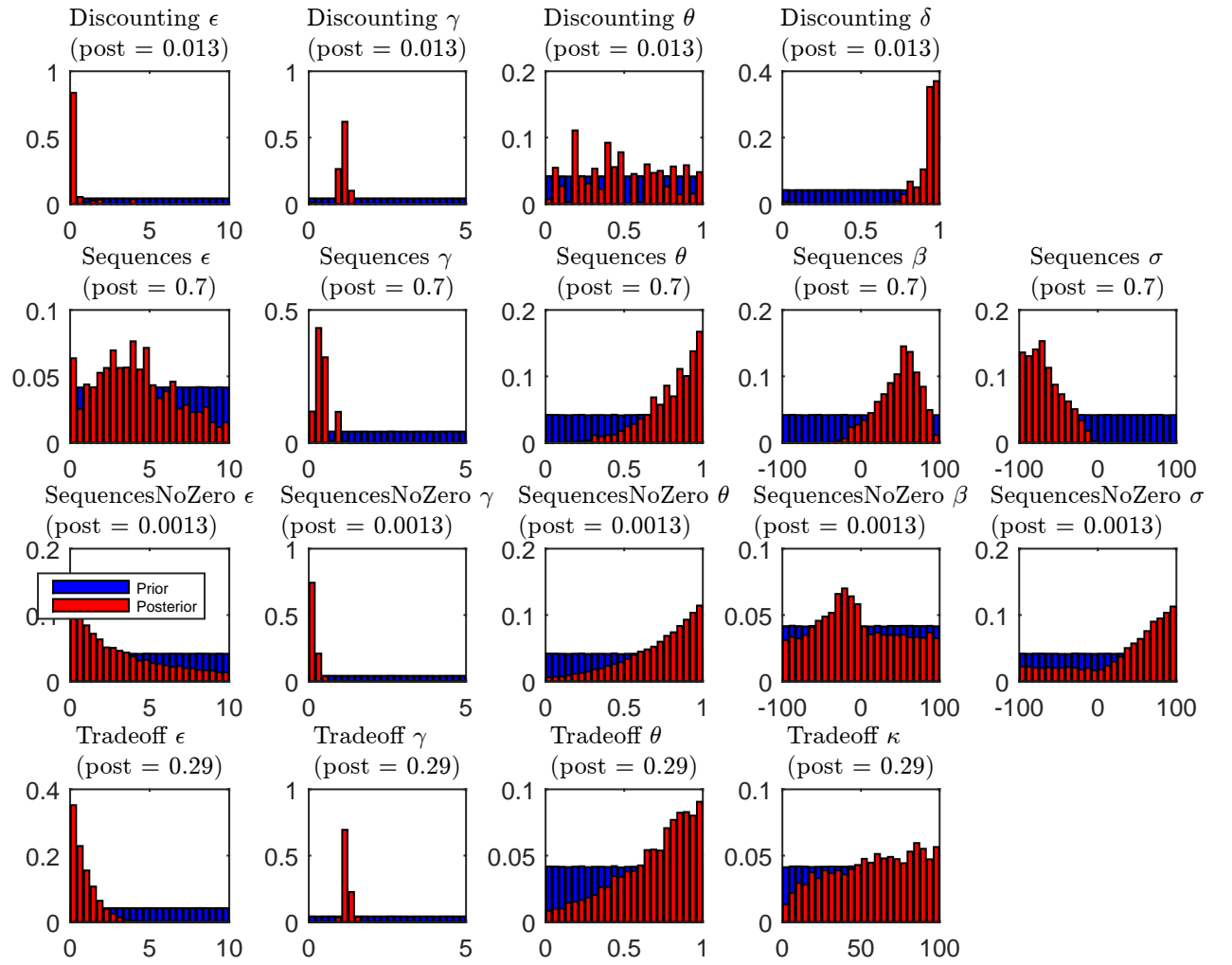

Supplement: Supplementary file 1 [file Scholten_Individuals.zip › plots/e29_p179_eg2_priors_and_posteriors.pdf]

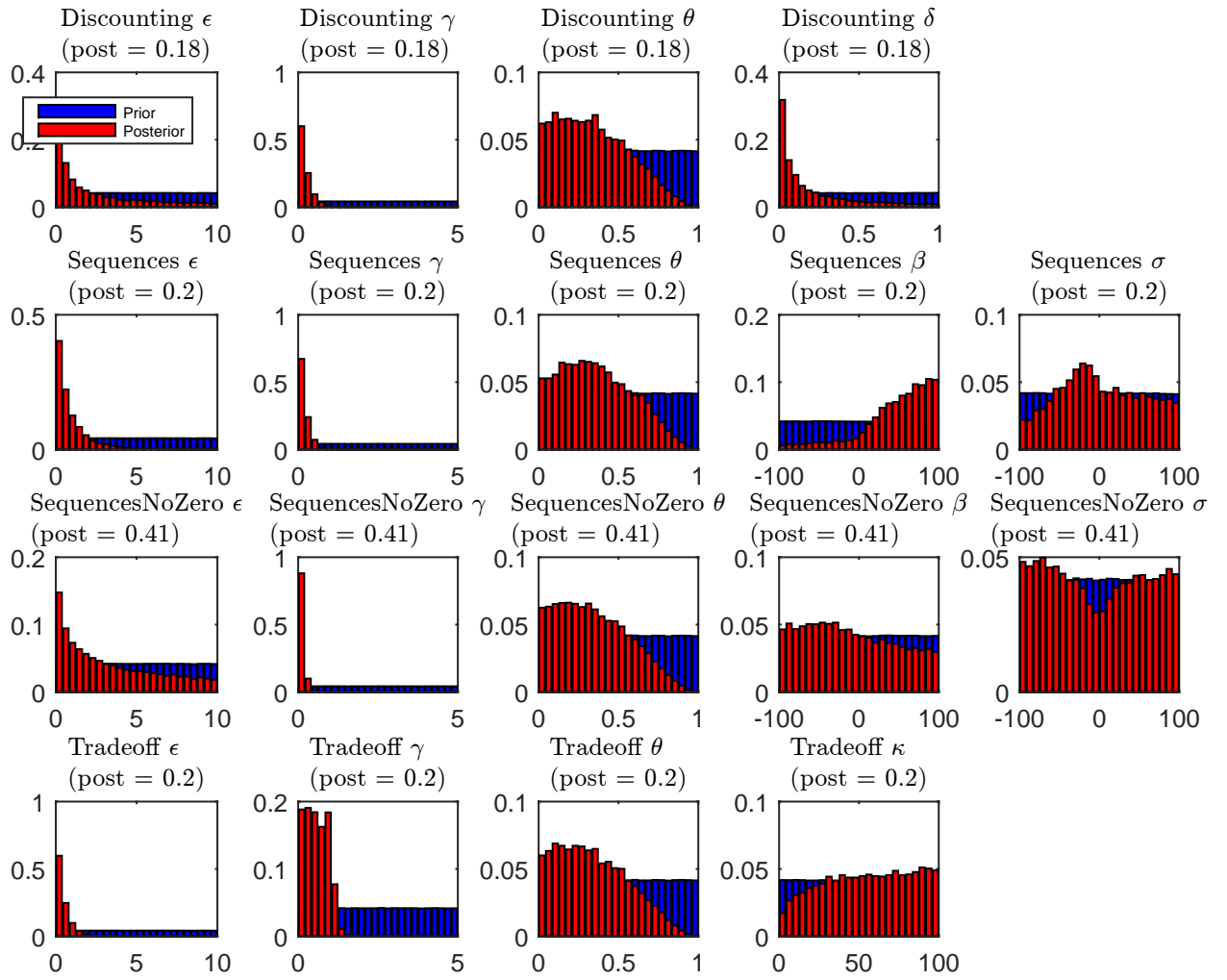

Supplement: Supplementary file 1 [file Scholten_Individuals.zip › plots/e29_p18_eg2_priors_and_posteriors.pdf]

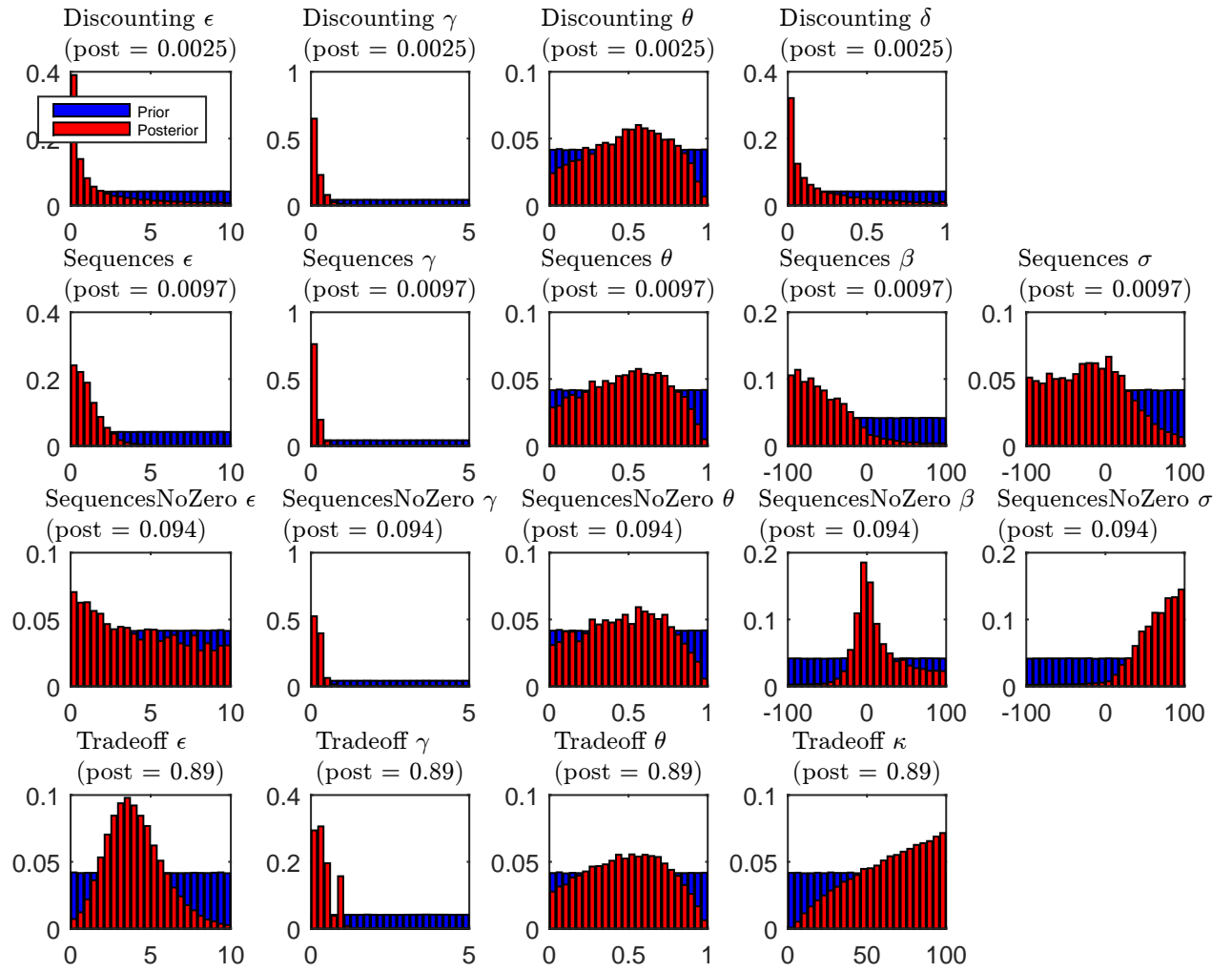

Supplement: Supplementary file 1 [file Scholten_Individuals.zip › plots/e29_p180_eg2_priors_and_posteriors.pdf]

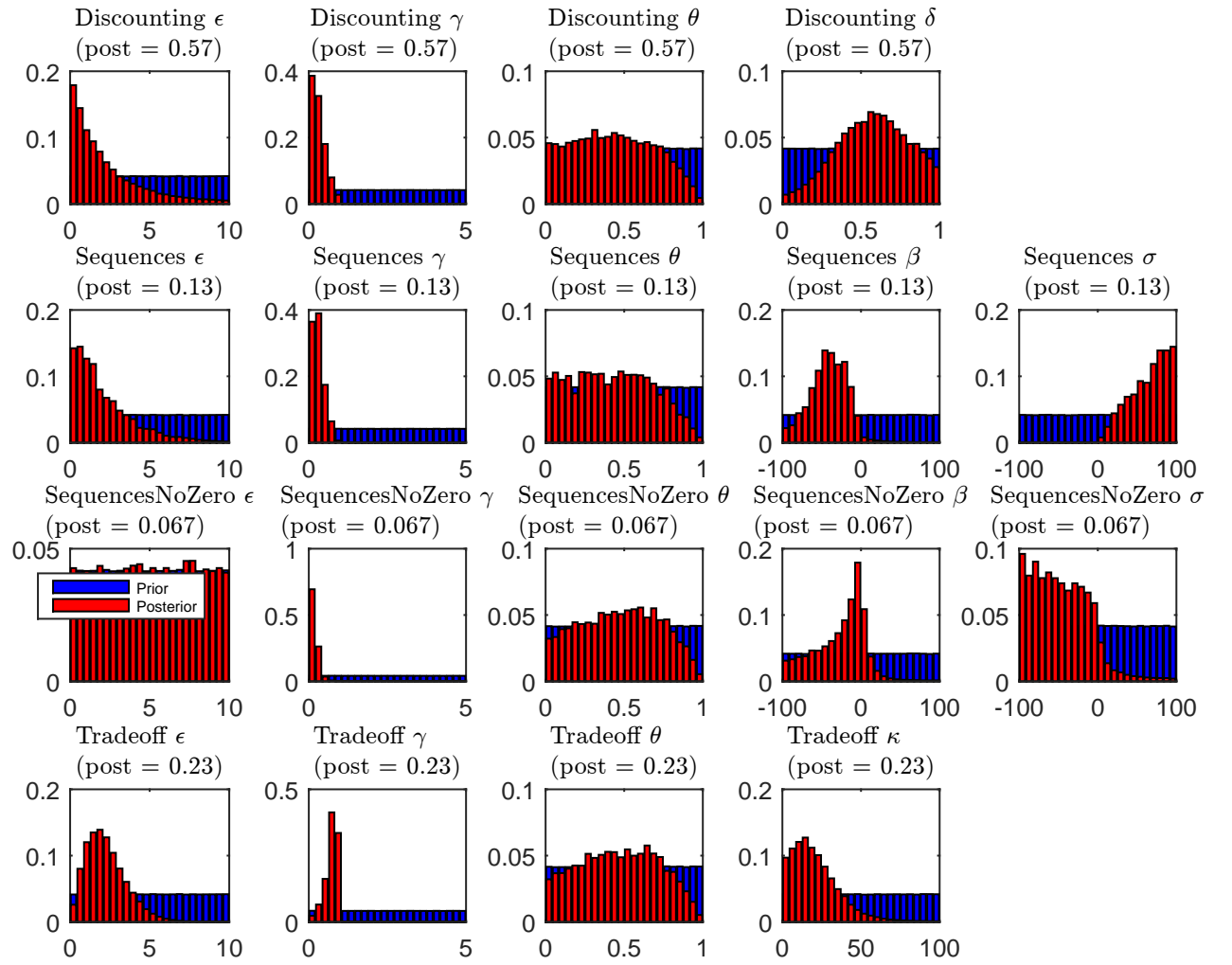

Supplement: Supplementary file 1 [file Scholten_Individuals.zip › plots/e29_p181_eg2_priors_and_posteriors.pdf]

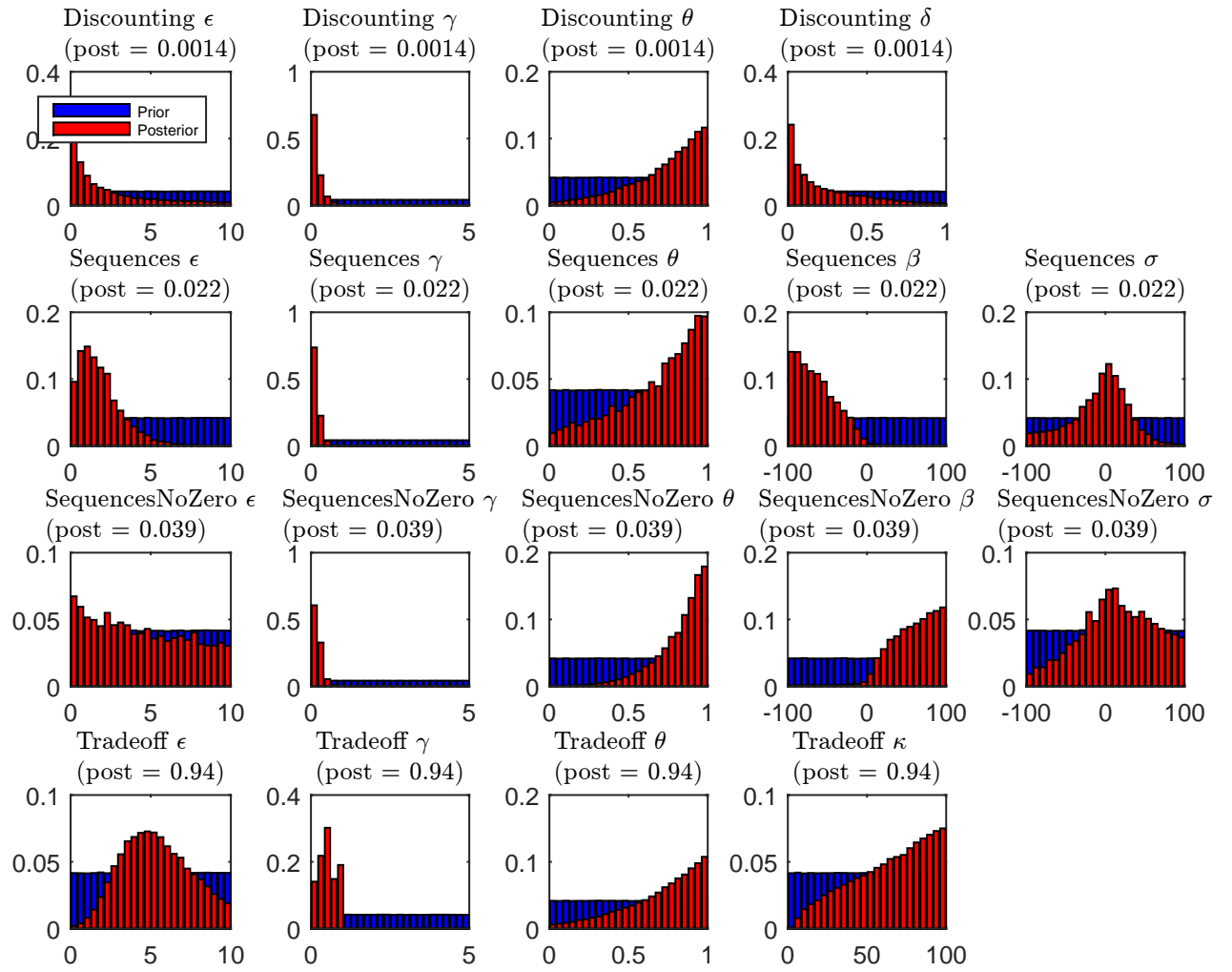

Supplement: Supplementary file 1 [file Scholten_Individuals.zip › plots/e29_p182_eg2_priors_and_posteriors.pdf]

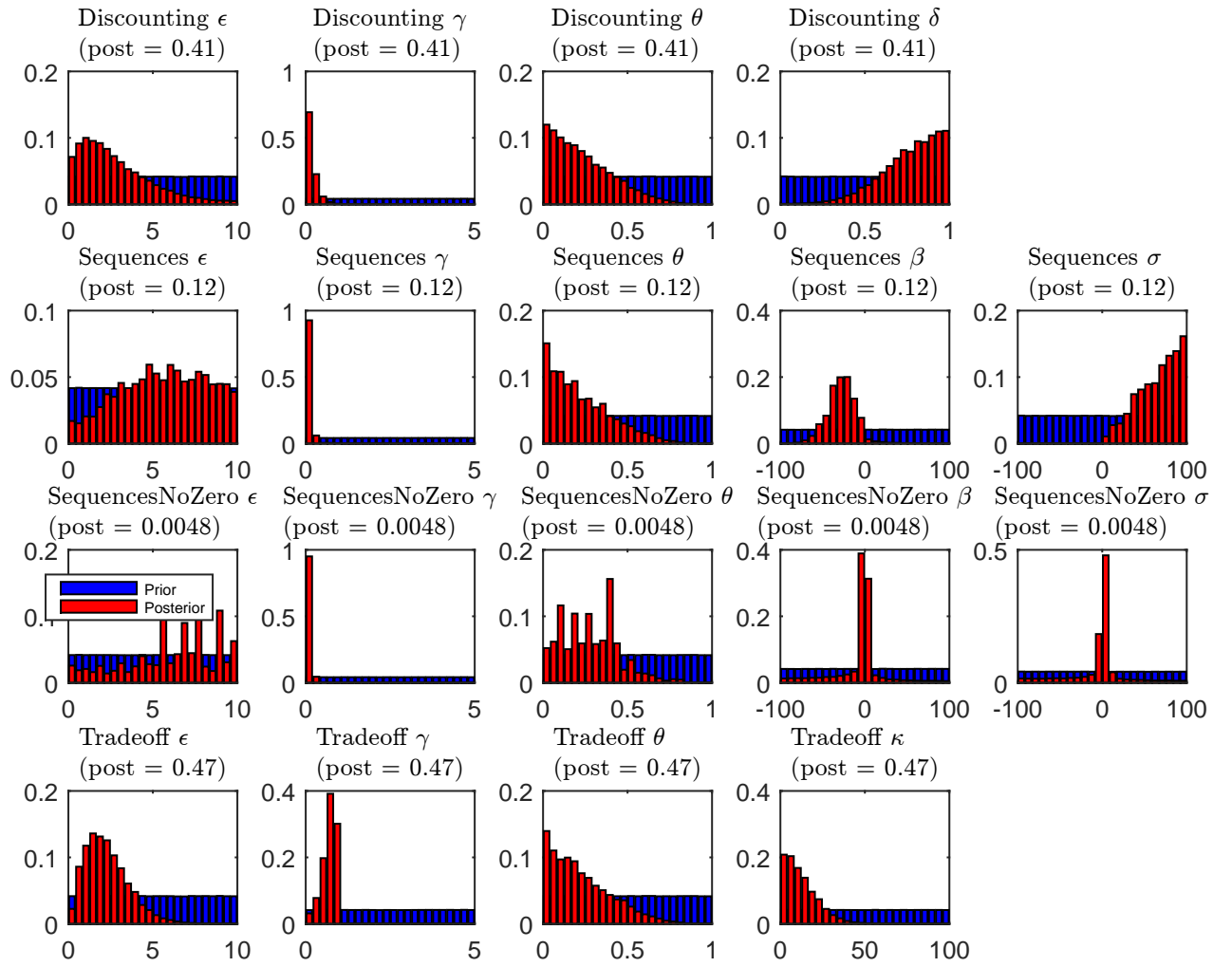

Supplement: Supplementary file 1 [file Scholten_Individuals.zip › plots/e29_p183_eg2_priors_and_posteriors.pdf]

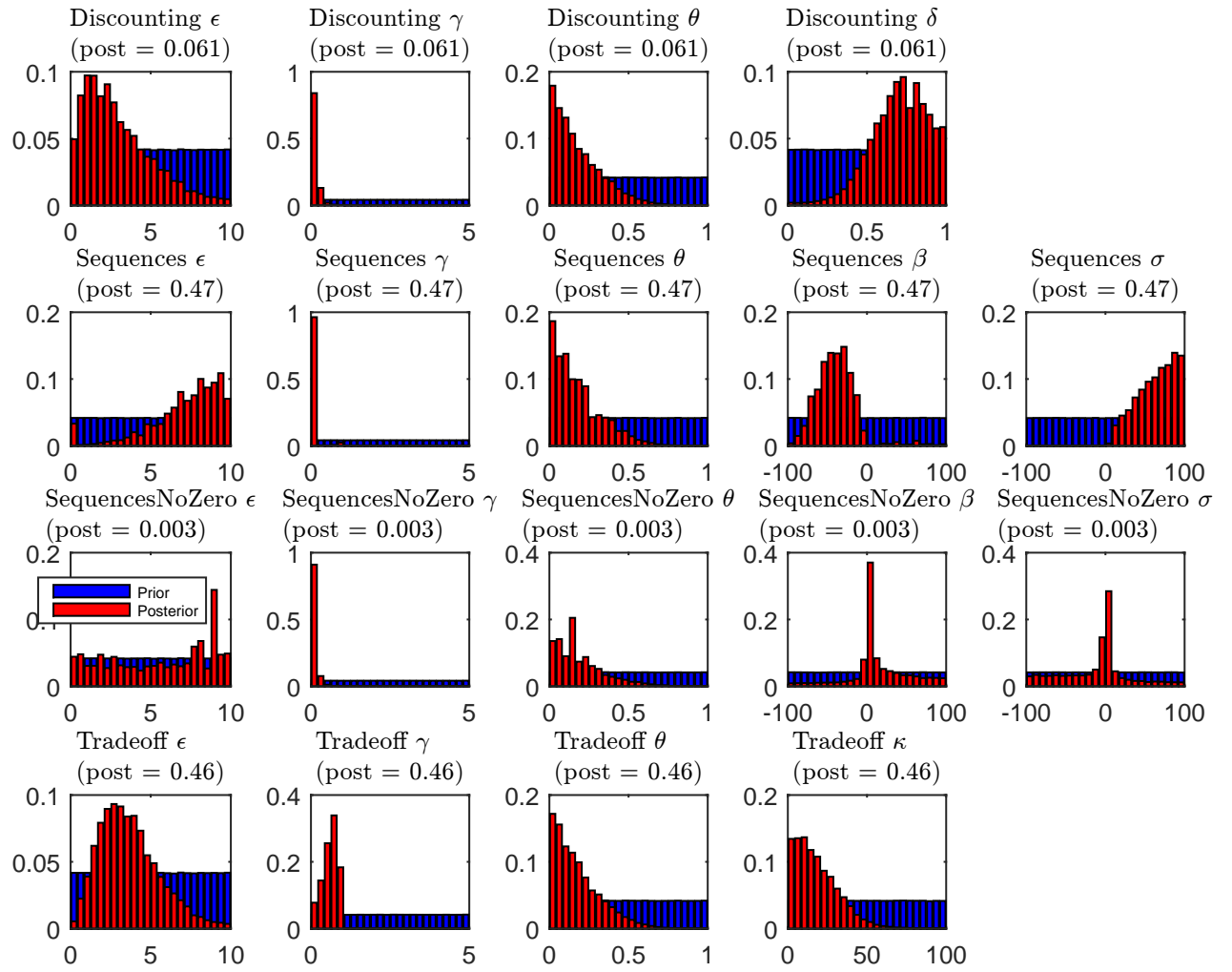

Supplement: Supplementary file 1 [file Scholten_Individuals.zip › plots/e29_p184_eg2_priors_and_posteriors.pdf]

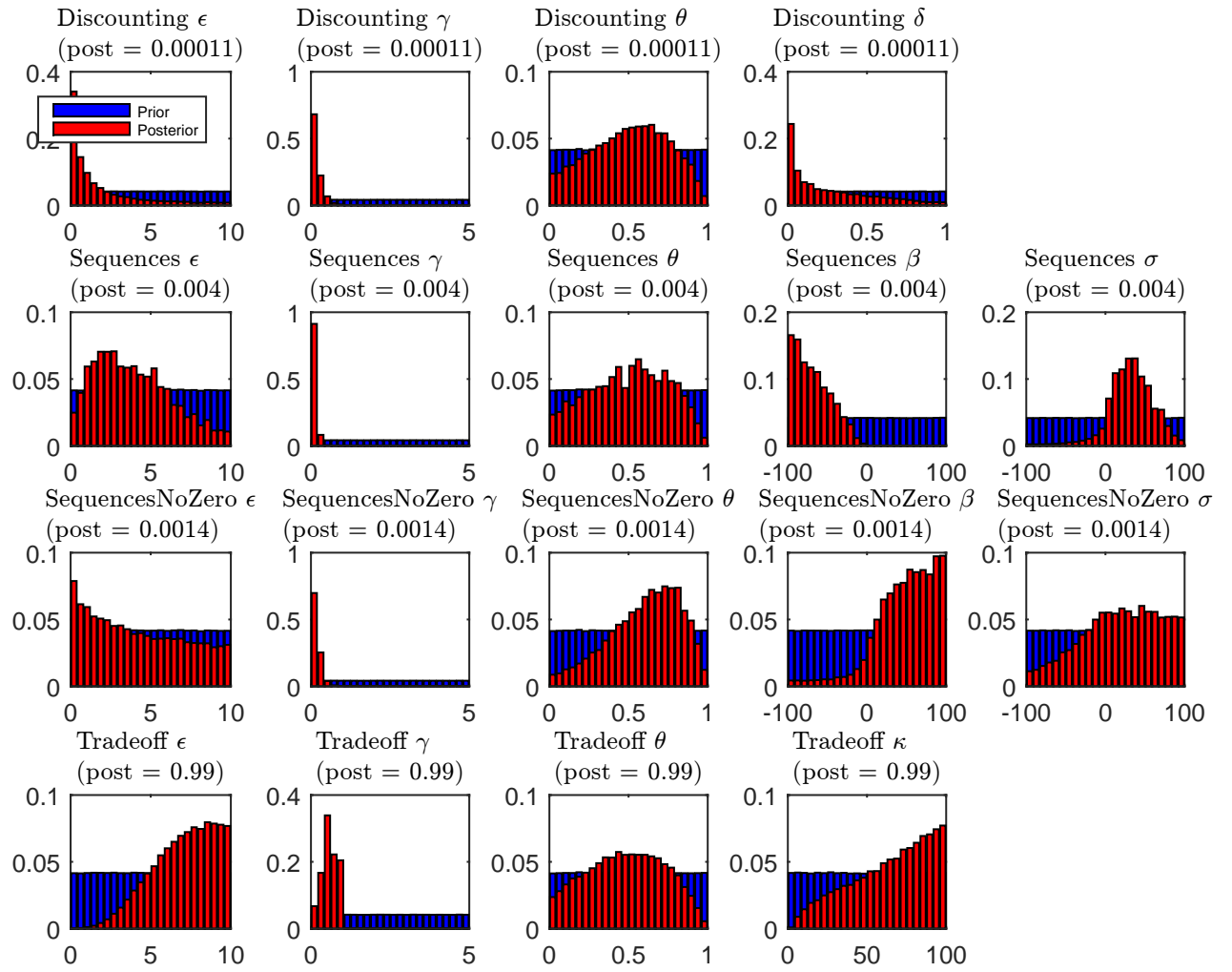

Supplement: Supplementary file 1 [file Scholten_Individuals.zip › plots/e29_p185_eg2_priors_and_posteriors.pdf]

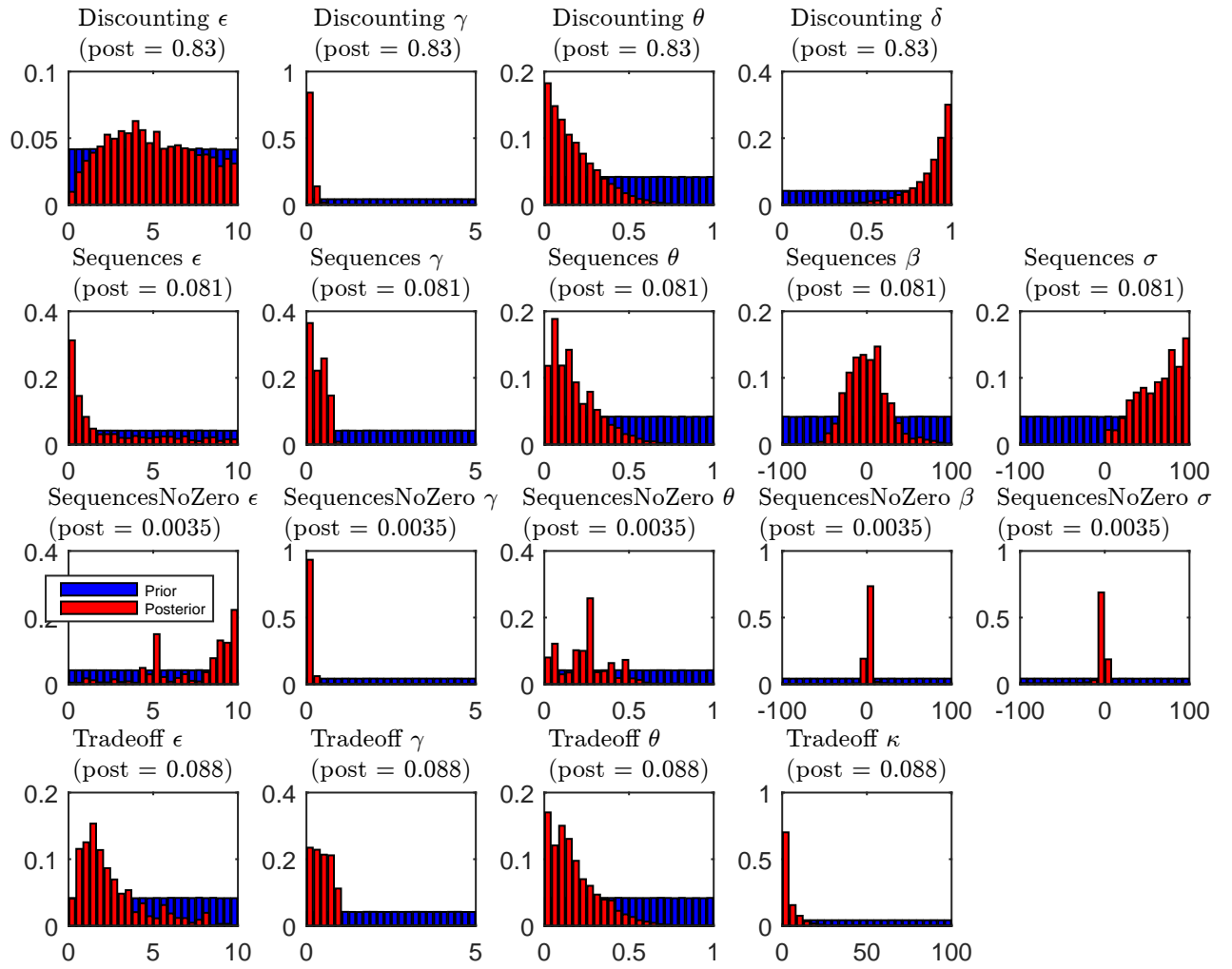

Supplement: Supplementary file 1 [file Scholten_Individuals.zip › plots/e29_p186_eg2_priors_and_posteriors.pdf]

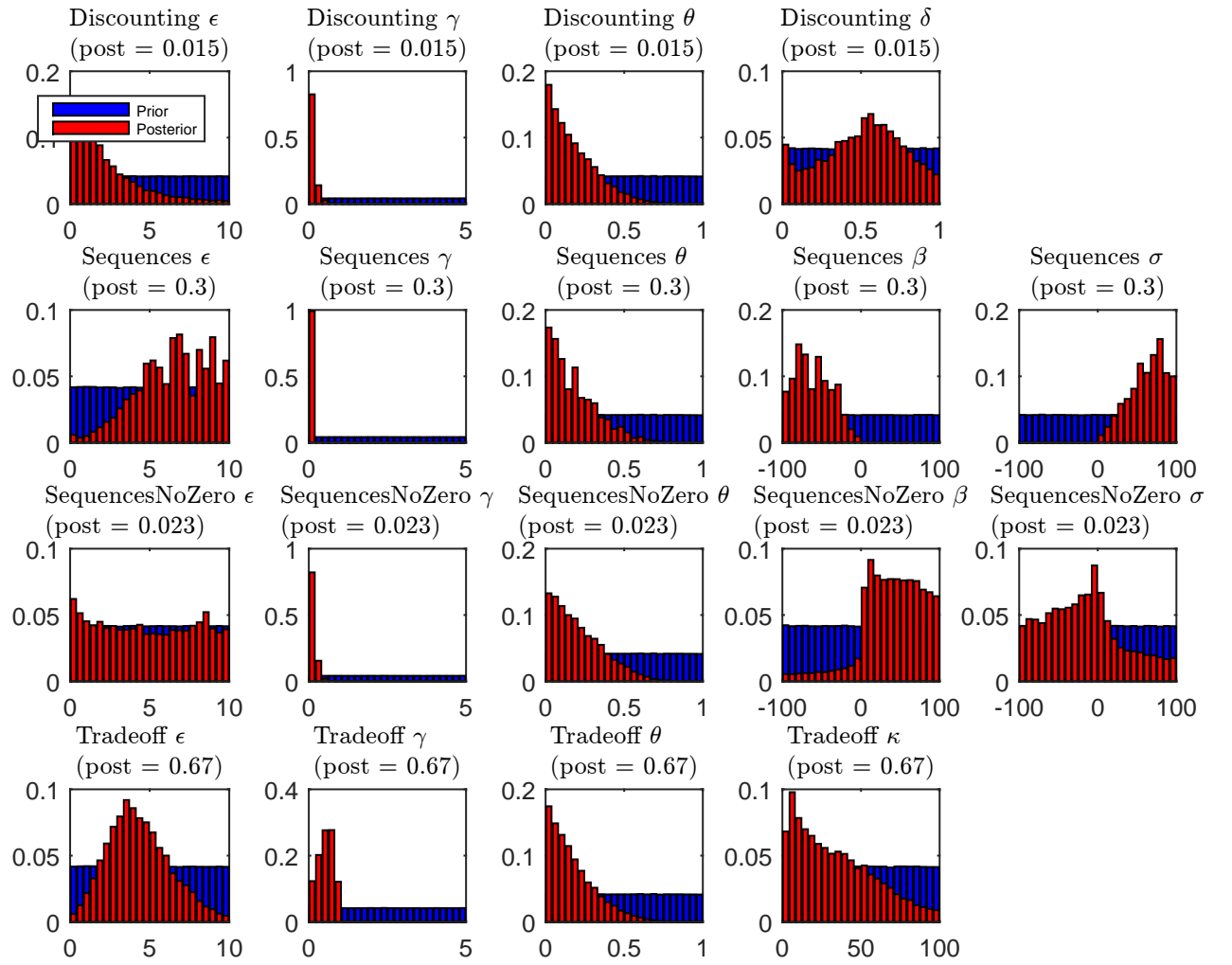

Supplement: Supplementary file 1 [file Scholten_Individuals.zip › plots/e29_p187_eg2_priors_and_posteriors.pdf]

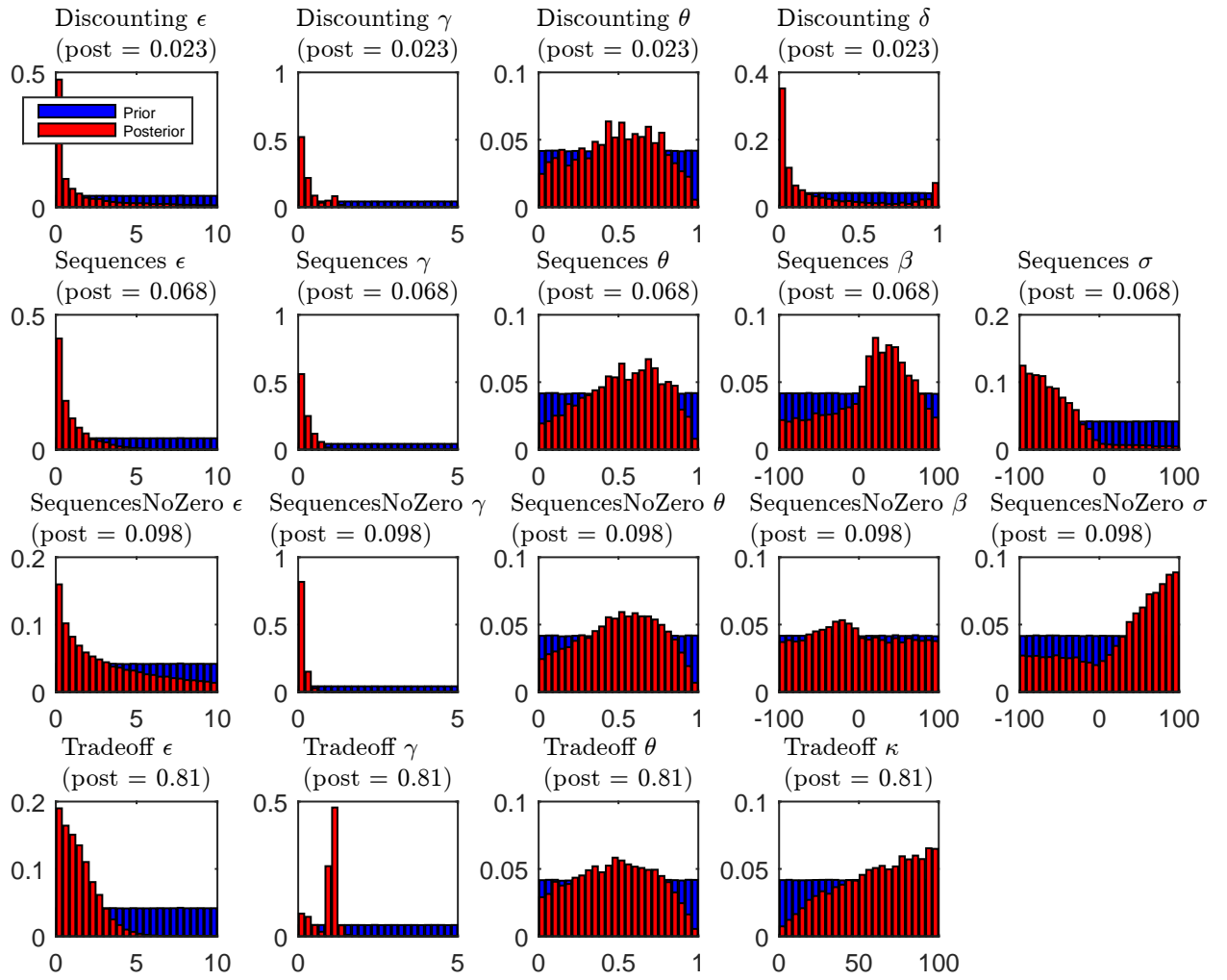

Supplement: Supplementary file 1 [file Scholten_Individuals.zip › plots/e29_p188_eg2_priors_and_posteriors.pdf]

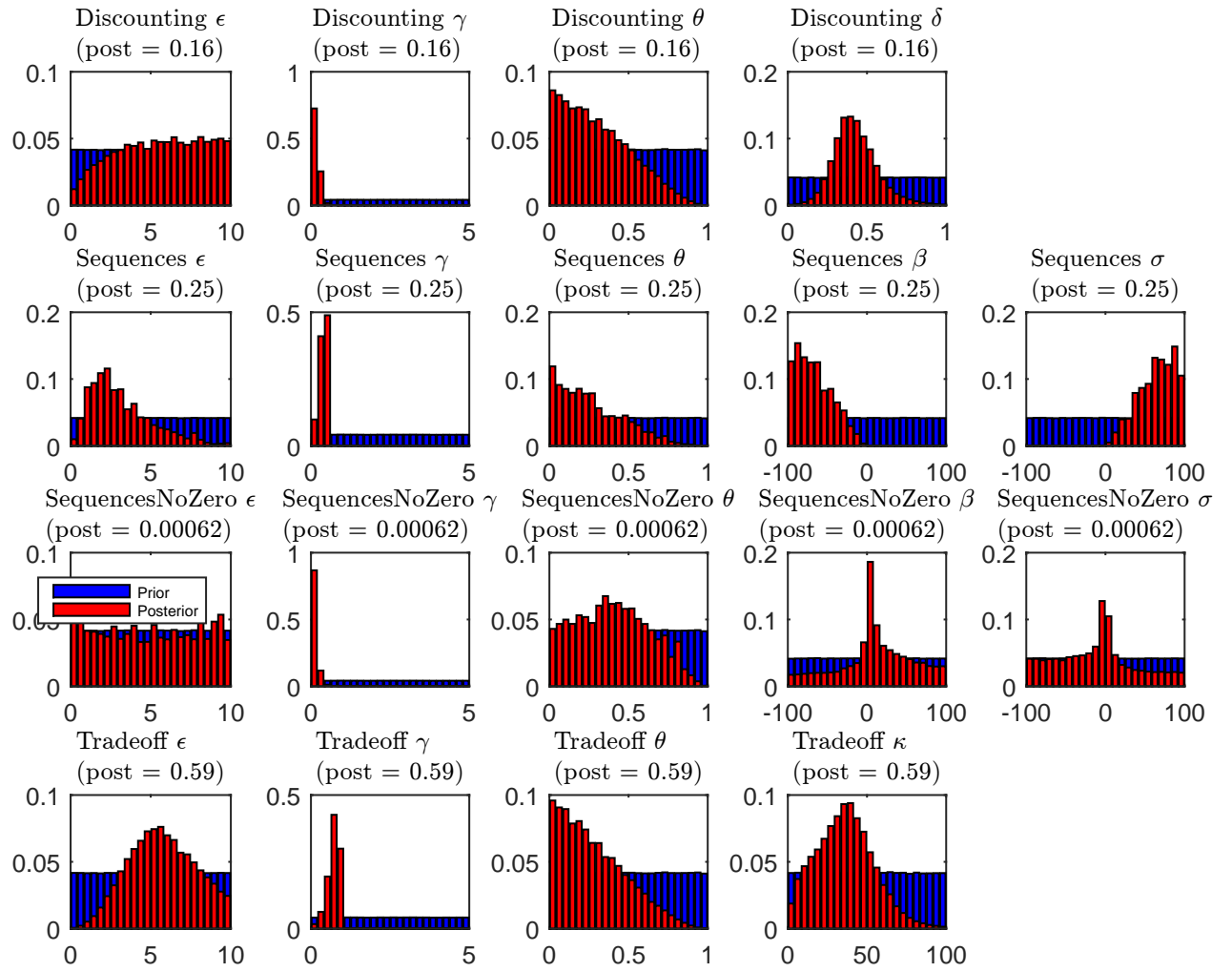

Supplement: Supplementary file 1 [file Scholten_Individuals.zip › plots/e29_p189_eg2_priors_and_posteriors.pdf]

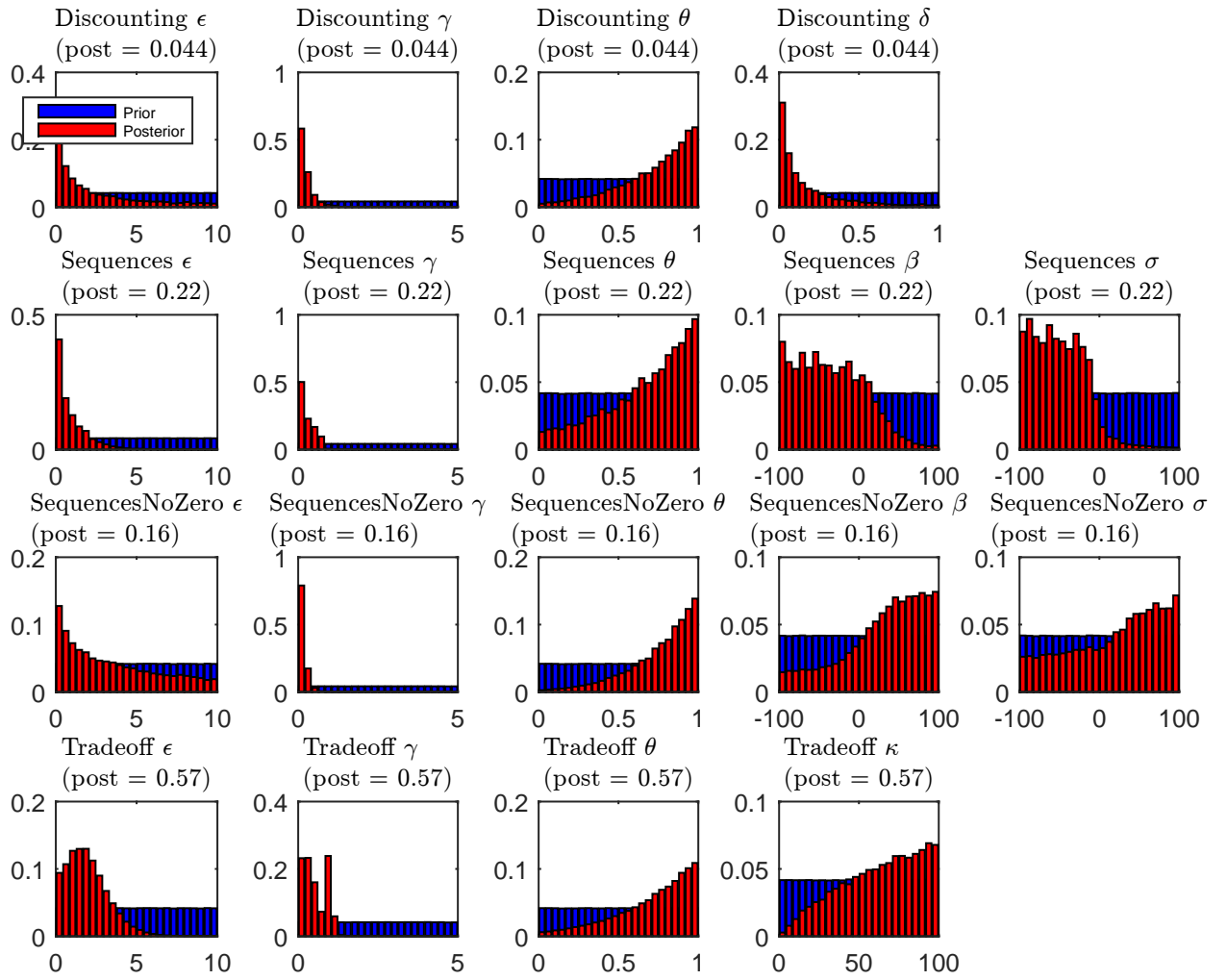

Supplement: Supplementary file 1 [file Scholten_Individuals.zip › plots/e29_p19_eg2_priors_and_posteriors.pdf]

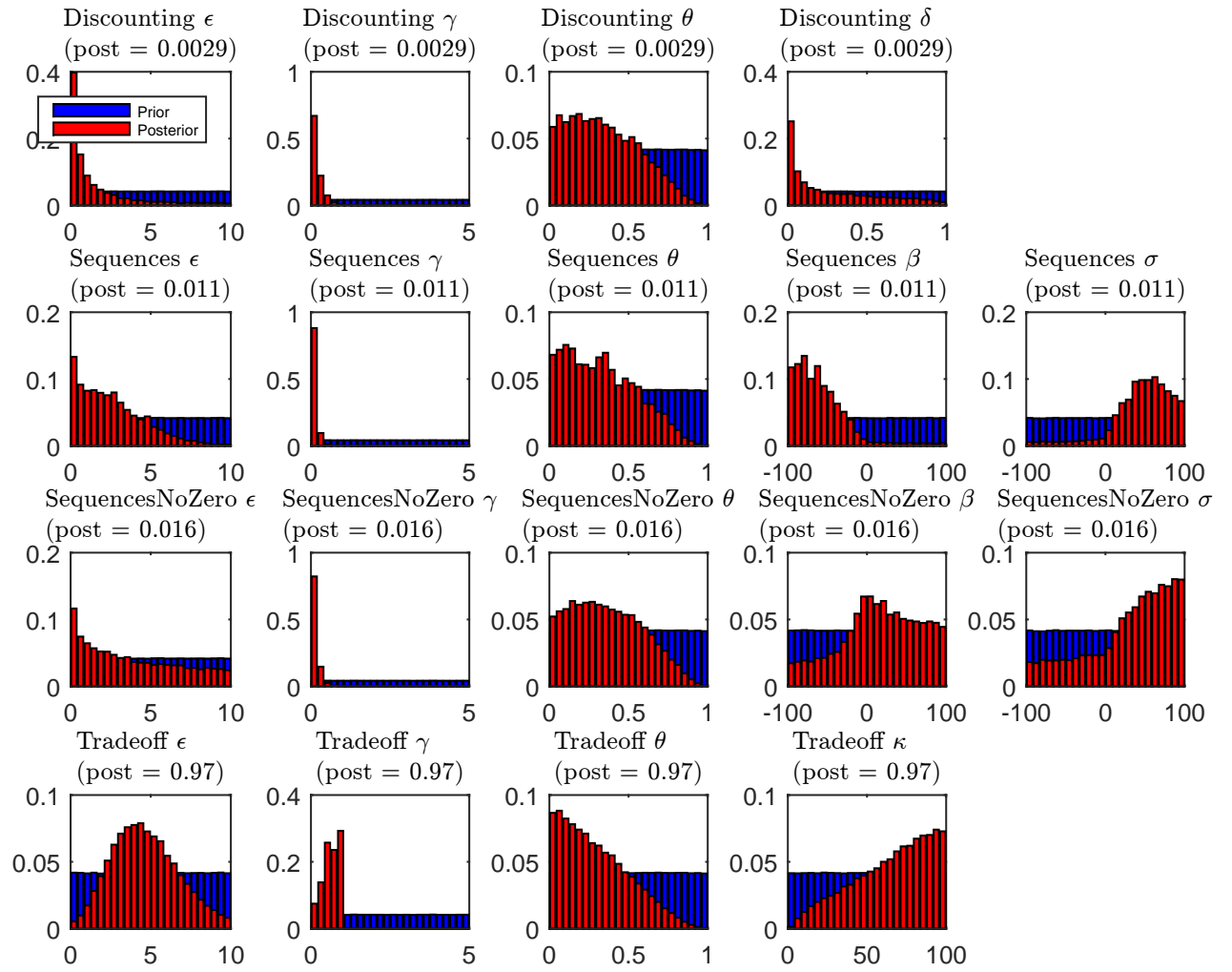

Supplement: Supplementary file 1 [file Scholten_Individuals.zip › plots/e29_p190_eg2_priors_and_posteriors.pdf]

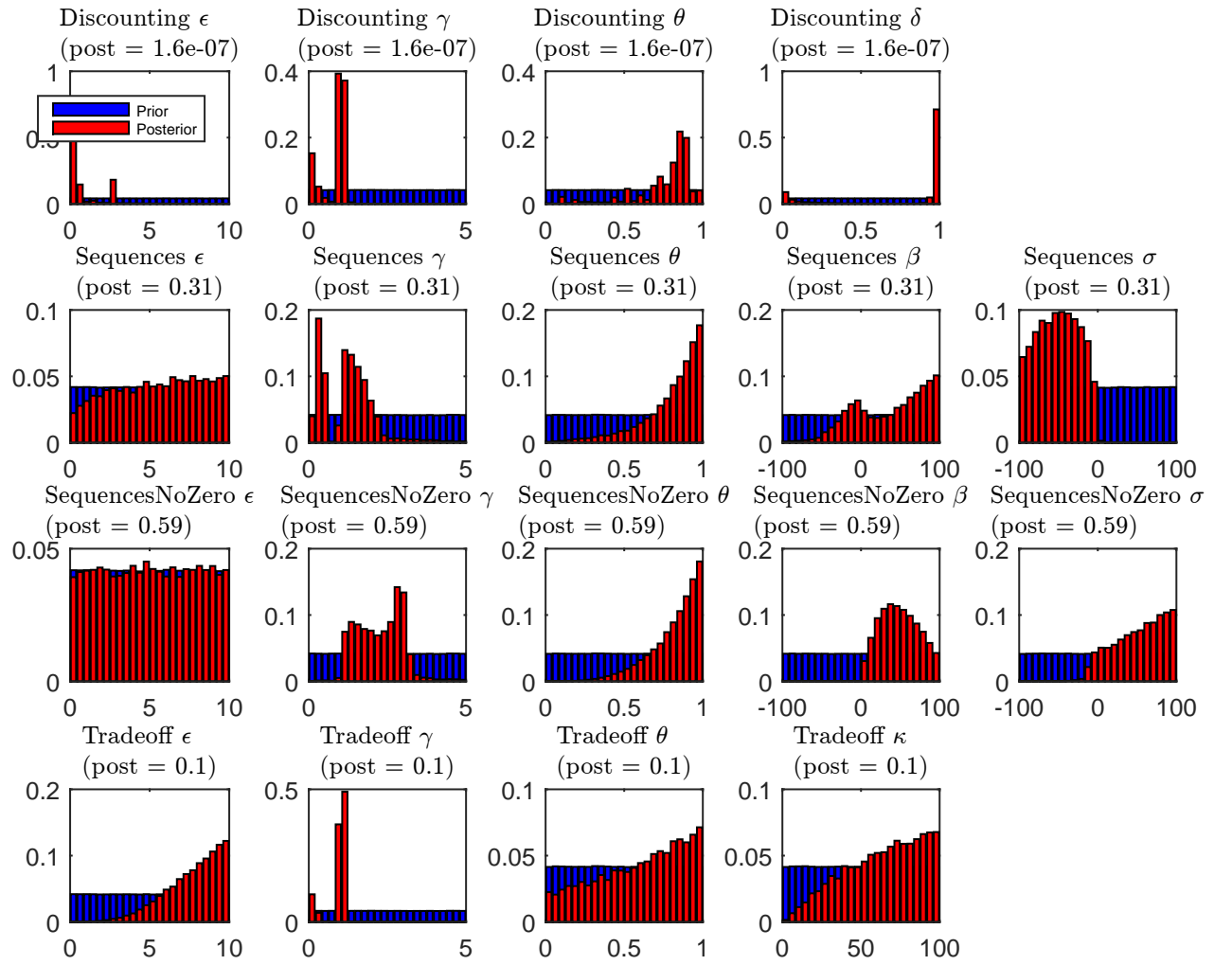

Supplement: Supplementary file 1 [file Scholten_Individuals.zip › plots/e29_p191_eg2_priors_and_posteriors.pdf]
