# Supplementary figures and images for: Cumulative Weighing of Time in Intertemporal Tradeoffs (part 3 of 3)
Source: J Exp Psychol Gen. 2016 Sep;145(9):1177–205. doi: 10.1037/xge0000198 (PMC4998108; doi:10.1037/xge0000198)

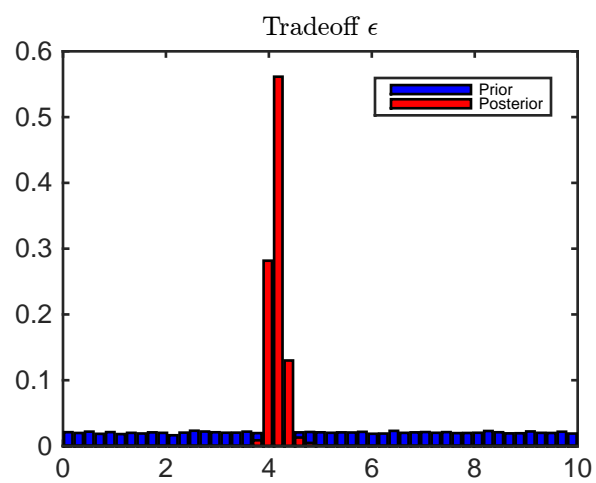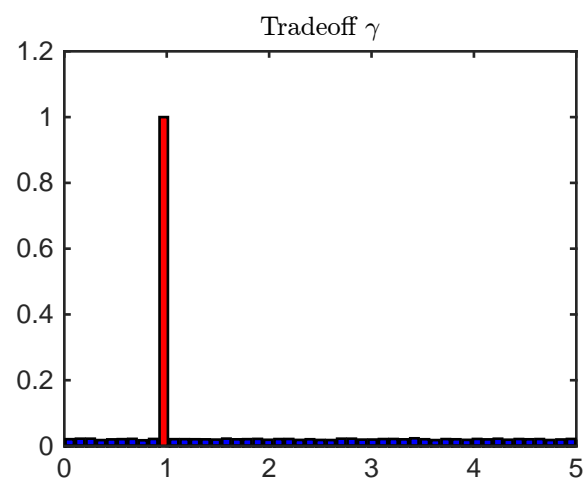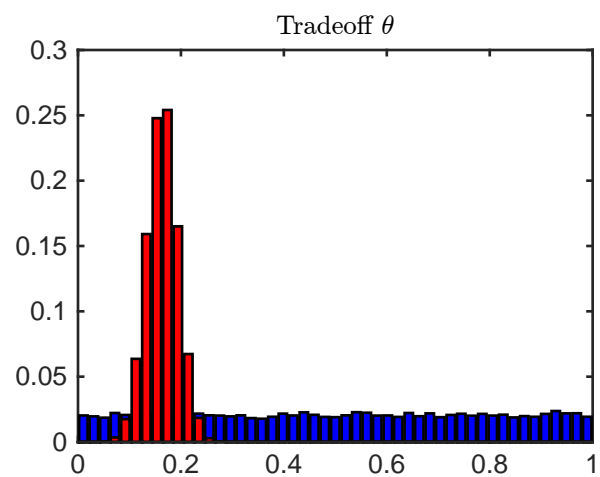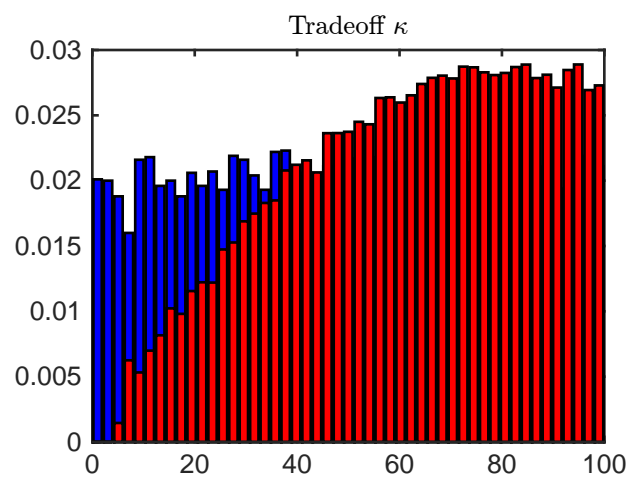

Supplement: Supplementary file 2 [file Scholten.zip › Scholten, Read, and Sanborn. Plots Posteriors. Groups/E29 Tradeoff Priors and Posteriors.pdf]

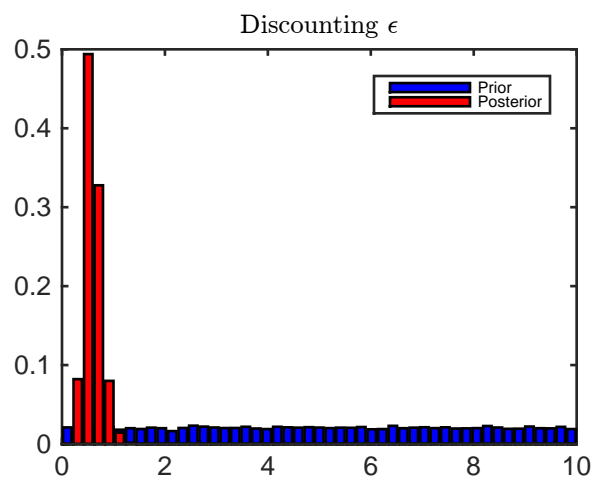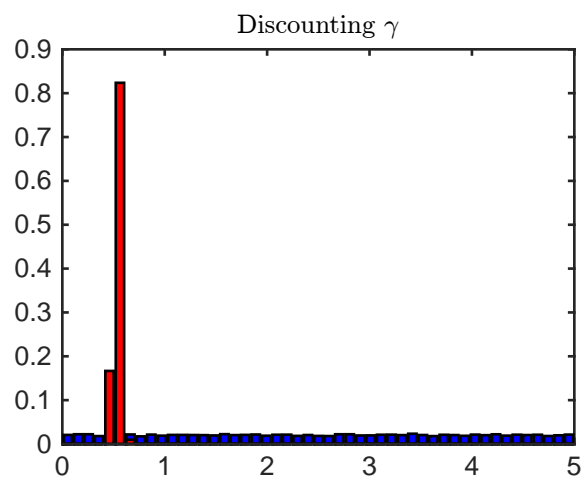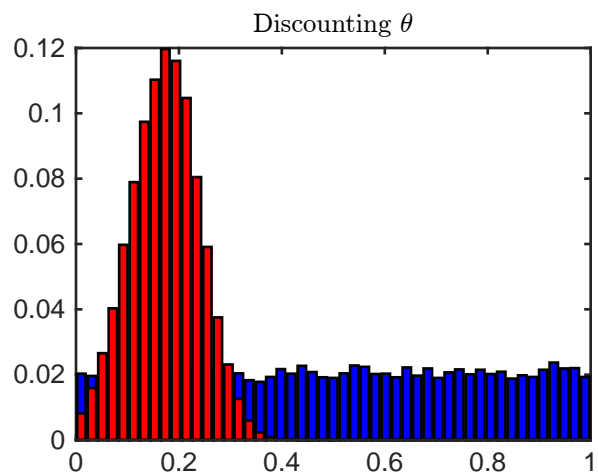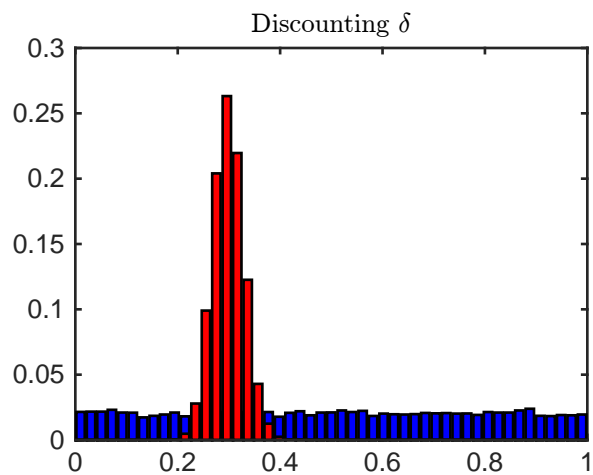

Supplement: Supplementary file 2 [file Scholten.zip › Scholten, Read, and Sanborn. Plots Posteriors. Groups/E30 Discounting Priors and Posteriors.pdf]

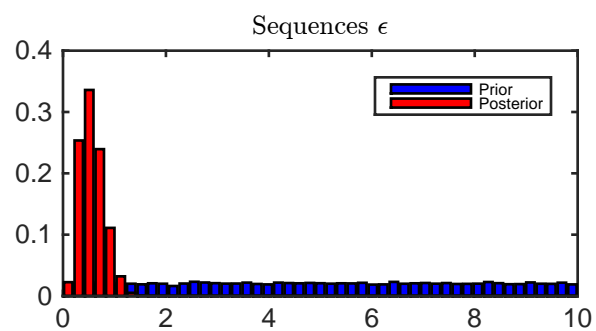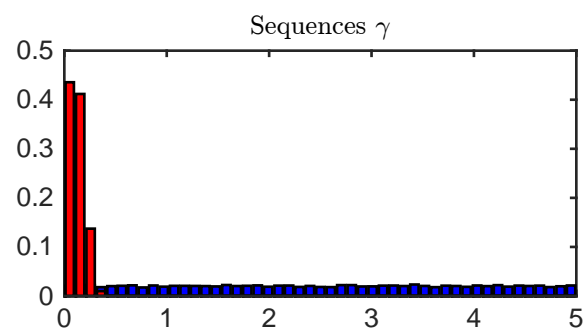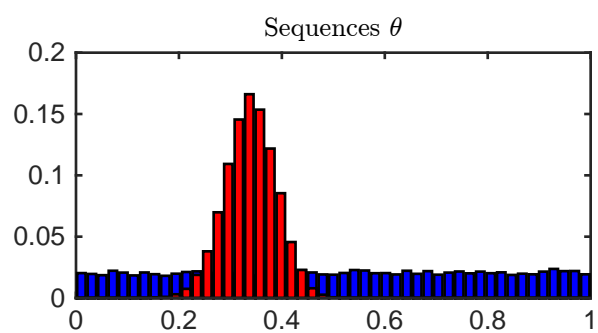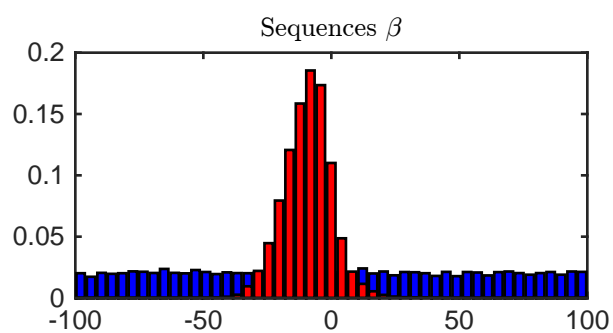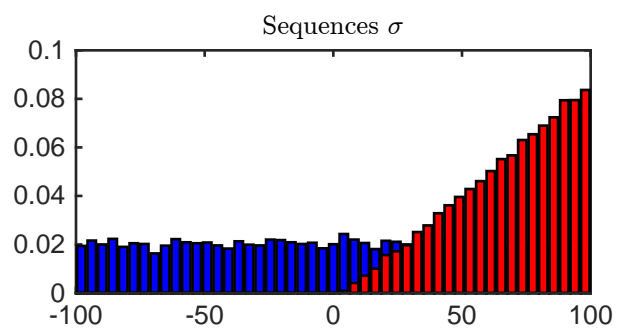

Supplement: Supplementary file 2 [file Scholten.zip › Scholten, Read, and Sanborn. Plots Posteriors. Groups/E30 Sequences Priors and Posteriors.pdf]

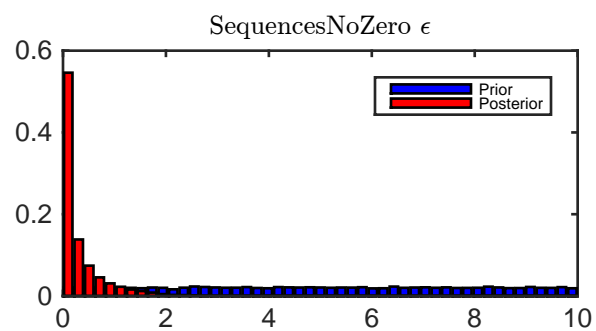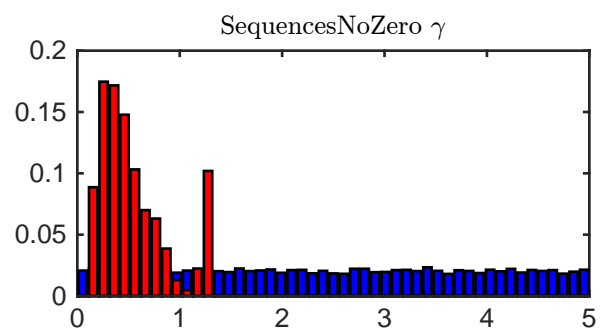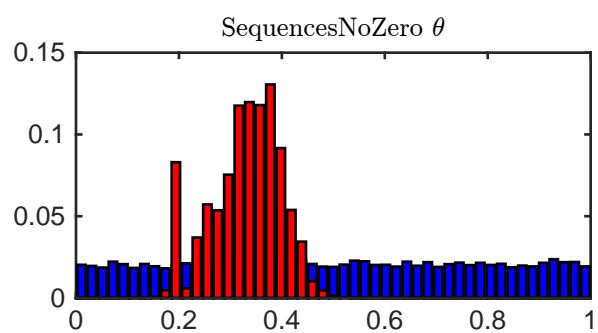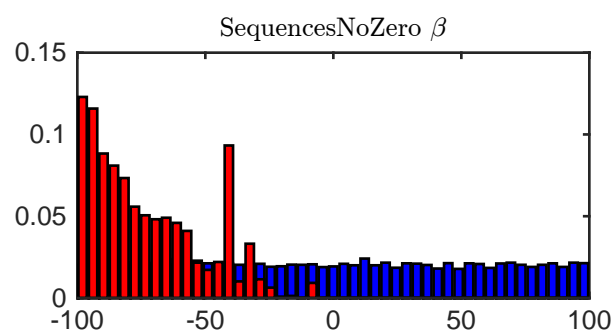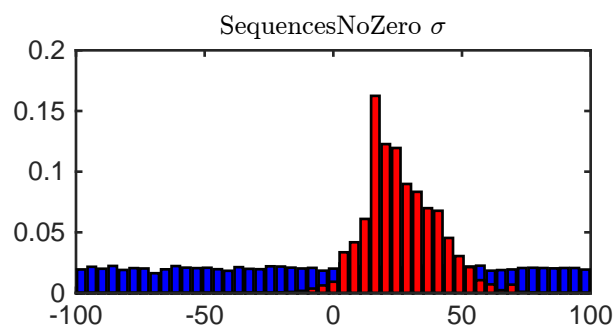

Supplement: Supplementary file 2 [file Scholten.zip › Scholten, Read, and Sanborn. Plots Posteriors. Groups/E30 SequencesNoZero Priors and Posteriors.pdf]

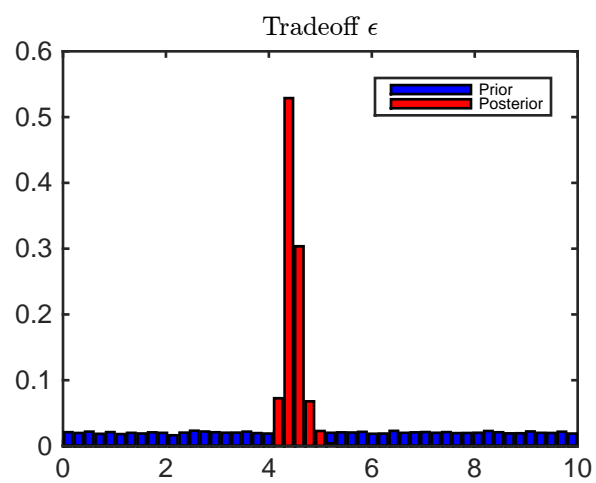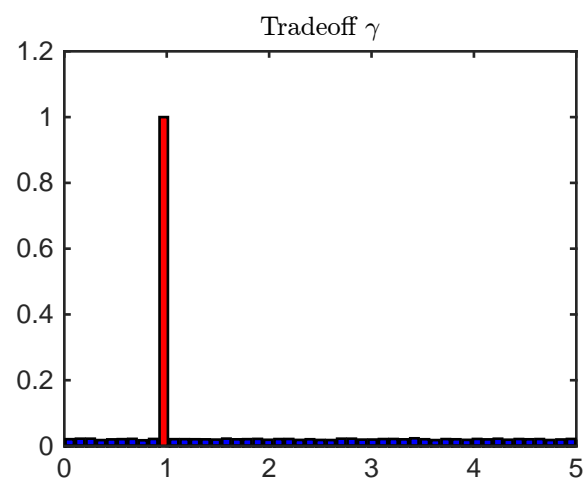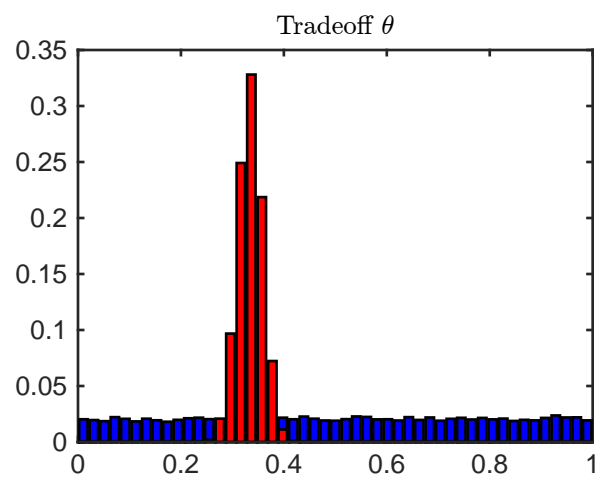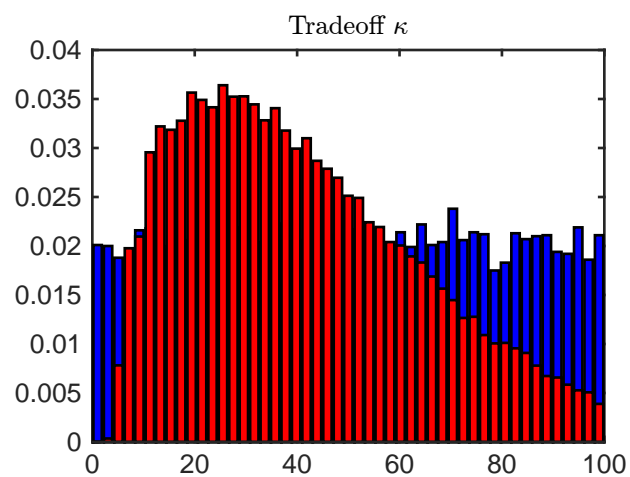

Supplement: Supplementary file 2 [file Scholten.zip › Scholten, Read, and Sanborn. Plots Posteriors. Groups/E30 Tradeoff Priors and Posteriors.pdf]

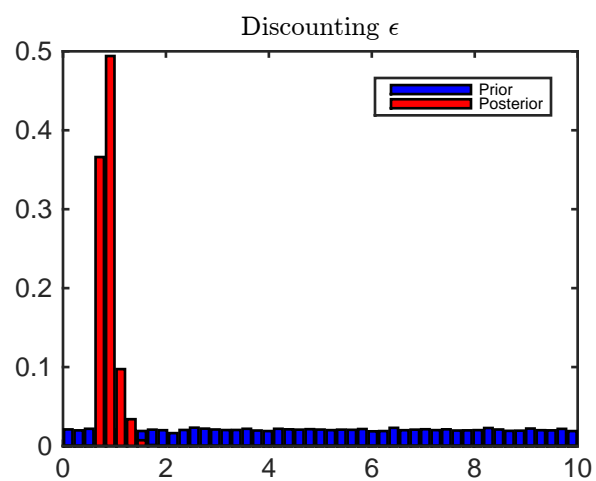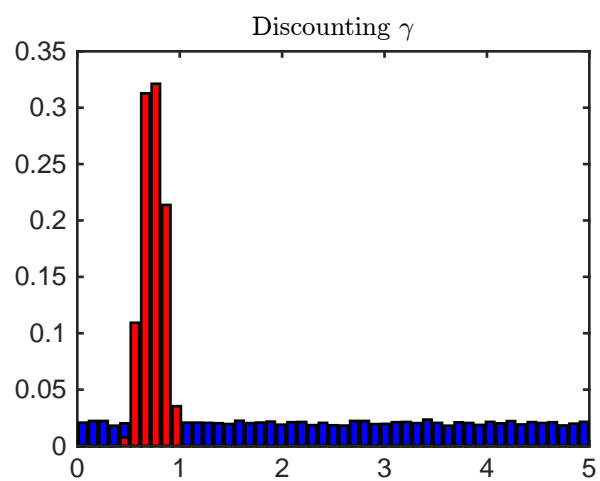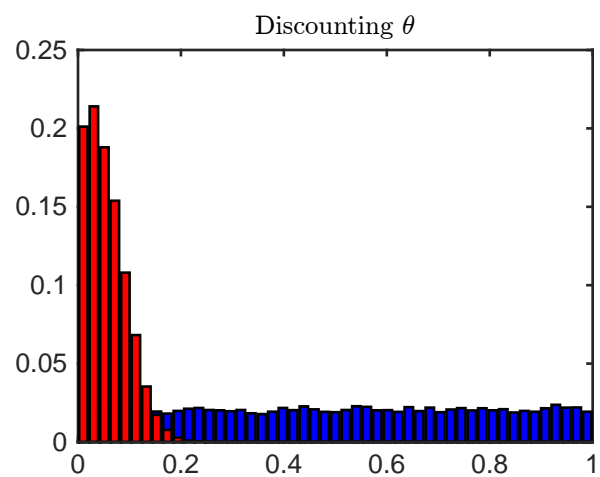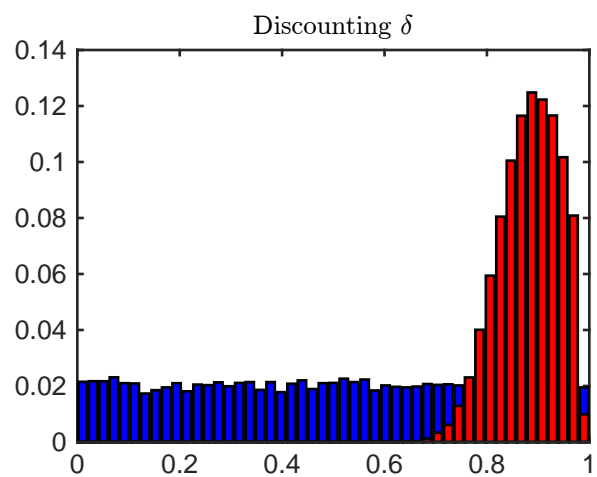

Supplement: Supplementary file 2 [file Scholten.zip › Scholten, Read, and Sanborn. Plots Posteriors. Groups/E31 Discounting Priors and Posteriors.pdf]

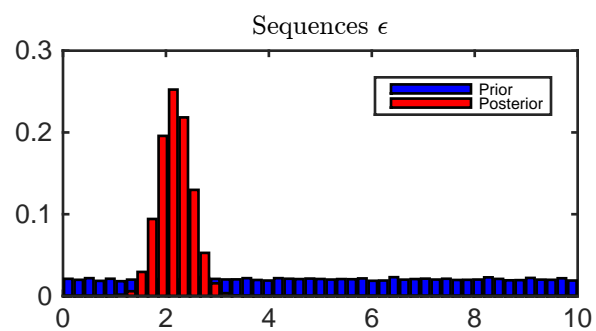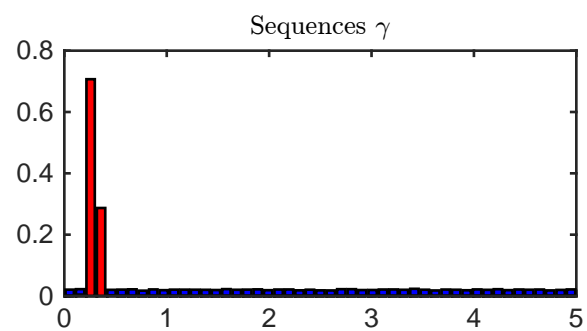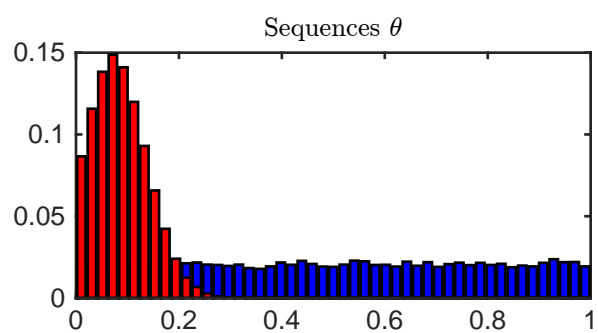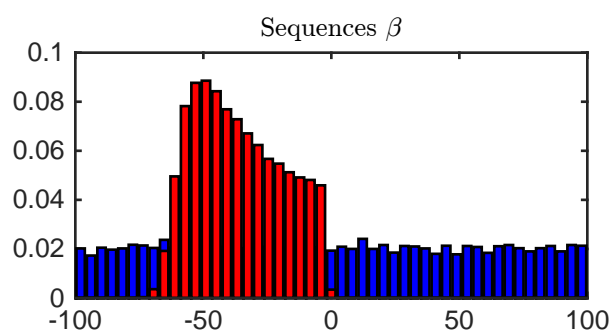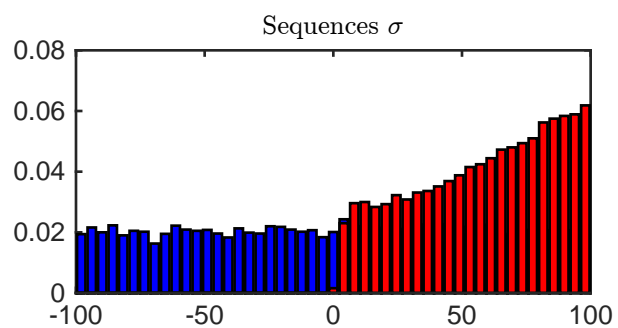

Supplement: Supplementary file 2 [file Scholten.zip › Scholten, Read, and Sanborn. Plots Posteriors. Groups/E31 Sequences Priors and Posteriors.pdf]

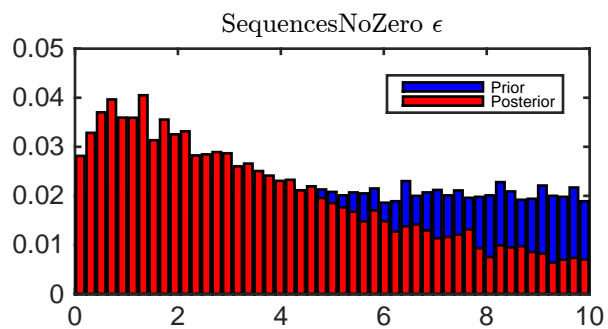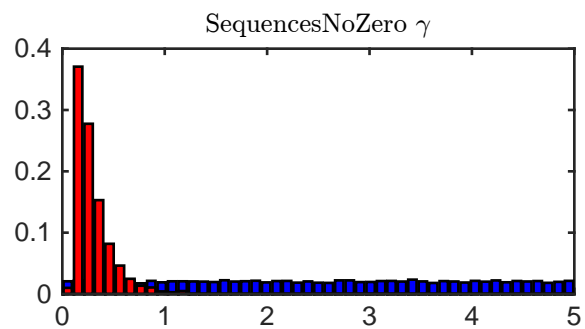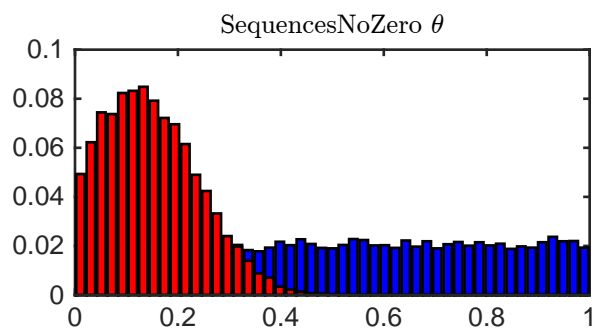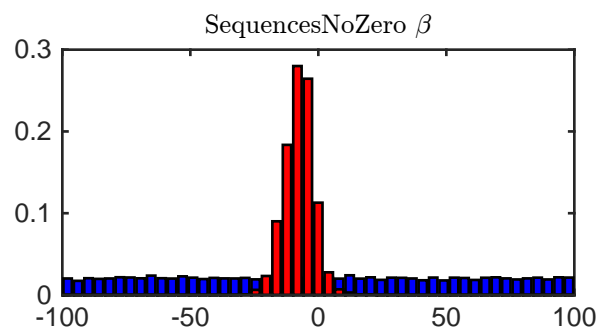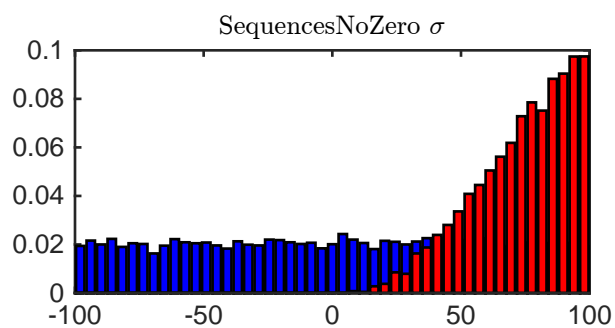

Supplement: Supplementary file 2 [file Scholten.zip › Scholten, Read, and Sanborn. Plots Posteriors. Groups/E31 SequencesNoZero Priors and Posteriors.pdf]

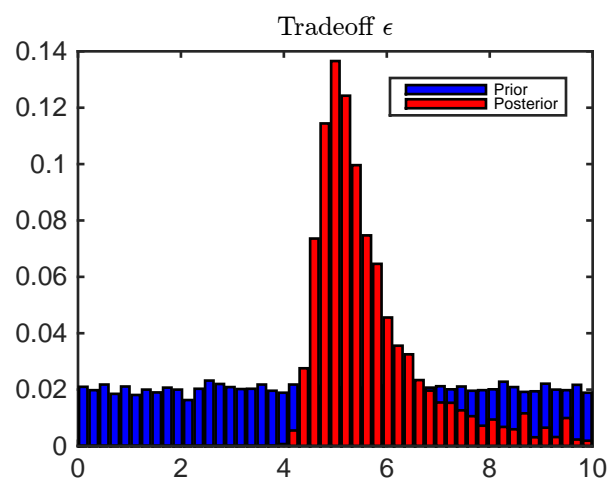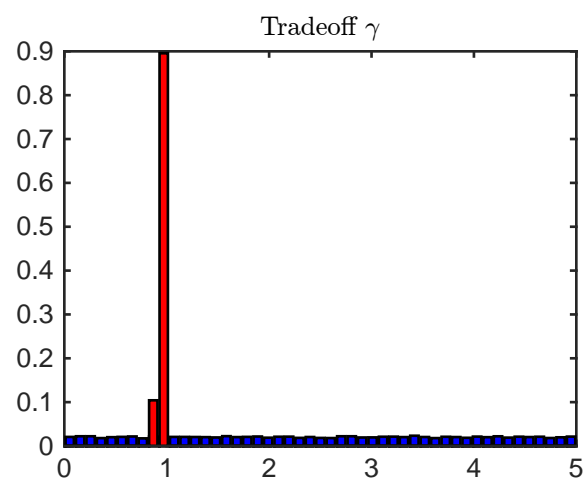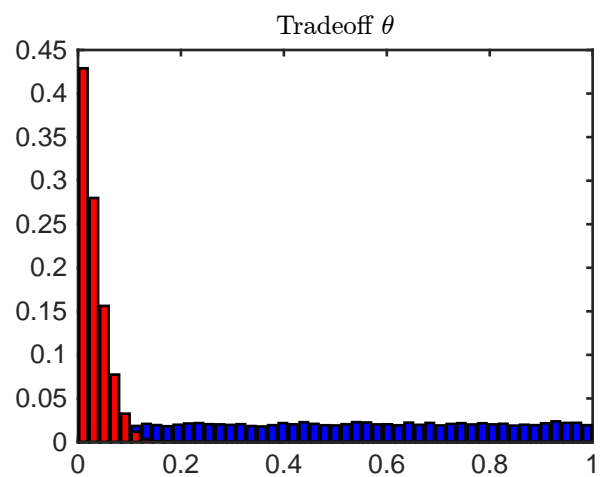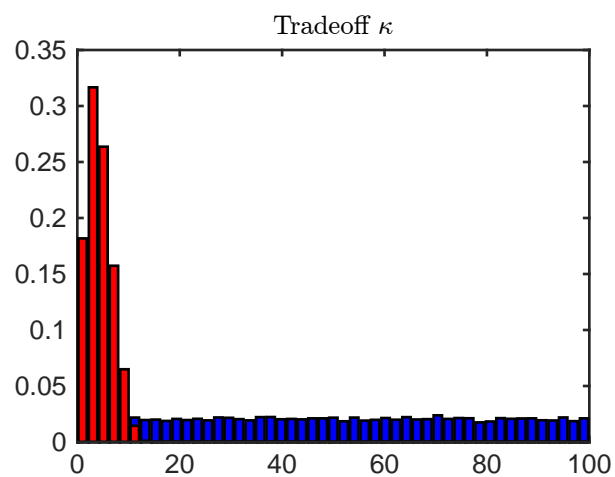

Supplement: Supplementary file 2 [file Scholten.zip › Scholten, Read, and Sanborn. Plots Posteriors. Groups/E31 Tradeoff Priors and Posteriors.pdf]
